# Psychiatric and neuropsychiatric sequelae of COVID-19 within 2 years: a multinational cohort study

**DOI:** 10.1186/s12916-025-03952-z

**Published:** 2025-03-07

**Authors:** Yi Chai, Ivan C. H. Lam, Kenneth K. C. Man, Joseph F. Hayes, Eric Y. F. Wan, Xue Li, Celine S. L. Chui, Wallis C. Y. Lau, Xiaoyu Lin, Can Yin, Min Fan, Esther W. Chan, Ian C. K. Wong, Hao Luo

**Affiliations:** 1 School of Public Health, Shenzhen University Medical School, Shenzhen University, Shenzhen, Guangdong China; 2 Centre for Safe Medication Practice and Research, Department of Pharmacology and Pharmacy, LKS Faculty of Medicine, The University of Hong Kong, Hong Kong SAR, China; 3 The Hong Kong Jockey Club Centre for Suicide Research and Prevention, The University of Hong Kong, Hong Kong SAR, China; 4 Research Department of Practice and Policy, School of Pharmacy, University College London, London, UK; 5 Laboratory of Data Discovery for Health (D24h), Hong Kong Science Park, Hong Kong SAR, China; 6 Division of Psychiatry, University College London, London, UK; 7 Camden and Islington NHS Foundation Trust, London, UK; 8 Department of Family Medicine and Primary Care, LKS Faculty of Medicine, The University of Hong Kong, Hong Kong SAR, China; 9 Department of Medicine, School of Clinical Medicine, LKS Faculty of Medicine, The University of Hong Kong, Hong Kong SAR, China; 10 School of Nursing, LKS Faculty of Medicine, The University of Hong Kong, Hong Kong SAR, China; 11 School of Public Health, LKS Faculty of Medicine, The University of Hong Kong, Hong Kong SAR, China; 12 Real-World Solutions, IQVIA, Durham, NC USA; 13 The University of Hong Kong Shenzhen Institute of Research and Innovation, Shenzhen, Guangdong China; 14 Aston Pharmacy School, Aston University, Birmingham, UK; 15 School of Public Health Sciences, University of Waterloo, Waterloo, ON Canada

**Keywords:** Long COVID, SARS-CoV-2, Psychiatric disorders, Neuropsychiatric disorders, Mental health, OMOP CDM

## Abstract

**Background:**

The long-term psychiatric and neuropsychiatric sequelae of COVID-19 across diverse populations remain not fully understood. This cohort study aims to investigate the short-, medium-, and long-term risks of psychiatric and neuropsychiatric disorders following COVID-19 infection in five countries.

**Methods:**

This population-based multinational network study used electronic medical records from France, Italy, Germany, and the UK and claims data from the USA. The initial target and comparator cohorts were identified using an exact matching approach based on age and sex. Individuals diagnosed with COVID-19 or those with a positive SARS-CoV-2 screening test between December 1, 2019, and December 1, 2020, were included as targets. Up to ten comparators without COVID-19 for each target were selected using the propensity score matching approach. All individuals were followed from the index date until the end of continuous enrolment or the last healthcare encounter. Cox proportional hazard regression models were fitted to estimate the risk of incident diagnosis of depression, anxiety disorders, alcohol misuse or dependence, substance misuse or dependence, bipolar disorders, psychoses, personality disorders, self-harm and suicide, sleep disorders, dementia, and neurodevelopmental disorders within the first 6 months (short-term), 6 months to 1 year (medium-term), and 1 to 2 years (long-term) post-infection.

**Results:**

A total of 303,251 individuals with COVID-19 and 22,108,925 individuals without COVID-19 from five countries were originally included. Within the first 6 months, individuals with COVID-19 had a significantly higher risk of any studied disorders in all databases, with Hazard Ratios (HRs) ranging from 1.14 (95% CI, 1.07–1.22) in Germany to 1.89 (1.64–2.17) in Italy. Increased risks were consistently observed for depression, anxiety disorders, and sleep disorders across almost all countries. During the medium- and long-term periods, higher risks were observed only for depression (medium-term: 1.29, 1.18–1.41; long-term: 1.36, 1.25–1.47), anxiety disorders (medium-term: 1.29, 1.20–1.38; long-term: 1.37, 1.29–1.47), and sleep disorders (medium-term: 1.10, 1.01–1.21; long-term: 1.14, 1.05–1.24) in France, and dementia (medium-term: 1.65, 1.28–2.10) in the UK.

**Conclusions:**

Our study suggests that increased risks of psychiatric and neuropsychiatric outcomes were consistently observed only within, and not after, the 6-month observation period across all databases, except for certain conditions in specific countries.

**Supplementary Information:**

The online version contains supplementary material available at 10.1186/s12916-025-03952-z.

## Background

The COVID-19 pandemic has had unprecedented impacts on global health. In addition to its physical health consequences, concerns have been raised regarding the potential psychiatric and neuropsychiatric complications following infection [1–5]. Studies have indicated that SARS-CoV-2 can directly invade the central nervous system or trigger an immune response that leads to inflammation and subsequent psychiatric and neuropsychiatric manifestations [6–9]. Furthermore, psychosocial stressors associated with the pandemic, such as social isolation, financial insecurity, and fear of illness, may contribute to a higher incidence of psychiatric symptoms [5, 10, 11].

Several studies have examined the association between COVID-19 infection and psychiatric and neuropsychiatric disorders [3, 12–15]. Three USA-based studies using population-based electronic medical records found that COVID-19 infection was associated with an increased risk of psychiatric and neuropsychiatric diagnoses within 6 months post-infection, highlighting a particular vulnerability to mood disorders, anxiety disorders, psychotic disorders, substance use disorders, insomnia, and dementia among individuals with COVID-19 [12, 13, 15]. A recent study included over 400,000 individuals from the UK Biobank database between March 1, 2020, and September 30, 2021, and found that individuals with COVID-19 were more likely to receive subsequent diagnoses of psychotic, mood, anxiety, alcohol use, and sleep disorders than their COVID-19-free counterparts during the 1-year follow-up period [14]. Furthermore, a binational study indicated that during a follow-up period of up to 28 months, COVID-19 infection was associated with a higher risk of psychiatric disorders, including anxiety disorders and post-traumatic stress disorders, in the UK and Hong Kong [16].

While these studies provide valuable insights into the psychiatric and neuropsychiatric sequelae of COVID-19, most have limited follow-up periods, leaving knowledge gaps regarding the post-acute sequelae of SARS-CoV-2. Additionally, the generalizability of findings from specific countries, predominantly the USA and the UK, to other regions is uncertain due to varying healthcare systems and public health responses to the pandemic. Therefore, findings generated from multiple populations and diverse healthcare systems, with large sample sizes, extended follow-up periods, standardized study designs, and a broader range of psychiatric and neuropsychiatric outcomes, are essential to enhance the current understanding of the psychiatric and neuropsychiatric sequelae of COVID-19 [17].

This population-based multinational network study aims to comprehensively investigate the short-, medium-, and long-term psychiatric and neuropsychiatric sequelae of COVID-19 using electronic medical records and claims data from over 25 million individuals across five countries. This investigation sheds light on the potential psychiatric and neurological effects of COVID-19 at various stages, thereby informing the development of effective prevention and management strategies for affected individuals.

## Methods

### Data sources

We used data from five databases. These comprised four electronic medical record databases, the IQVIA Longitudinal Patient Database France (France IQVIA), IQVIA Disease Analyser Germany (Germany IQVIA), Longitudinal Patient Database Italy (Italy IQVIA), and IQVIA Medical Research Data UK (UK IMRD). The fifth database was the IQVIA PharMetrics Plus in the USA (US PharMetrics Plus), a claims-based database. All data were routinely collected and converted to the Observational Medical Outcomes Partnership (OMOP) Common Data Model (CDM), version 5, maintained by the Observational Health Data Sciences and Informatics (OHDSI) network [18]. The standardized structure and content of these databases allow data partners to execute common analytical syntax locally and contribute aggregated results without sharing individual-level data. The databases used in this study are all population-based databases covering patients with diverse socioeconomic characteristics. These databases have been extensively used in previous studies to assess the physical and psychological consequences of the COVID-19 pandemic [19–21]. Detailed descriptions of these databases, including their representativeness and comparability, are presented in Additional file 1: Table S1 and have been previously reported [20–22].

### Study design and participants

This study used data between December 1, 2018, and December 1, 2022. We identified target and comparator cohorts using an exact matching approach based on age and sex. The target cohort consisted of all individuals who received a diagnosis of COVID-19 or a positive SARS-CoV-2 screening test result between December 1, 2019, and December 1, 2020. Targets with any negative SARS-CoV-2 screening test results within 3 days of the index date were excluded to eliminate potential false positives. The diagnostic codes for identifying individuals with COVID-19 were determined through internal and external consultations with epidemiologists, clinicians, and data scientists, and the list was further adjusted during the preliminary testing process. Additional file 1: Table S2 shows the final list of diagnostic codes. The initial comparator cohort included individuals without any diagnosis or positive test results for COVID-19 between December 1, 2019, and December 1, 2022. The earliest date of COVID-19 confirmation was designated as the index date for the targets, and the same dates were assigned as the index date for their corresponding matched comparators.

Individuals were eligible for the study if they had continuous observation for at least 365 days prior to the index date and at least 1 day after the index date. A maximum of ten comparators for each target were selected using the propensity score matching approach, although some targets may have fewer than ten comparator candidates. The standardized difference of mean was used to assess the covariate balance between target and comparator cohorts, with a threshold of 0.2 [23]. The propensity scores were calculated based on a wide range of predefined generic characteristics, including demographics, diagnoses, drug exposures, measurement, medical procedures, and health service use behaviors observed 365 days prior to and on the index date [24, 25]. A large-scale regularized regression was employed for covariate selection and propensity score calculation, which has been widely used in previous research for confounding adjustment [25–28]. All individuals were followed from the index date until the end of continuous enrolment (for UK IMRD and US PharMetrics Plus) or the last healthcare encounter (for France IQVIA, Germany IQVIA, and Italy IQVIA). Our preliminary analysis identified 805,065 targets and 39,754,216 comparators in the US PharMetrics Plus database. Due to computational limitations, we used a stratified random sampling approach to select 20% of individuals from the target and comparator cohorts within each age and sex stratum for the US PharMetrics Plus database.

### Outcomes

The study outcomes included depression, anxiety disorders, alcohol misuse or dependence, substance misuse or dependence, bipolar disorders, psychoses, personality disorders, self-harm and suicide, sleep disorders, dementia, and neurodevelopmental disorders. The corresponding diagnostic codes are shown in Additional file 1: Table S3. Only the first diagnosis of each outcome following the index date was used. We also estimated the risk of any of the 11 outcomes to assess the overall psychiatric and neuropsychiatric sequelae of COVID-19.

### Statistical analysis

The short-, medium-, and long-term periods were defined as 6 months, 6 months to 1 year, and 1 to 2 years since the index date, respectively. For the analysis of each outcome, individuals were excluded if they had the outcome of interest within 365 days prior to the start of the short- (the index date), medium-, and long-term observation to ensure the identification of incident cases. We used the term “incident” broadly to represent a first-ever diagnosis and a potentially prevalent diagnosis that became active after at least 365 days. The exclusion was performed before the propensity score matching step. We tabulated baseline characteristics to evaluate covariate balance before and after propensity score adjustment and report the incidence of outcomes by disease and database. Cox proportional hazards regression models were fitted to quantify the short-, medium-, and long-term associations between each outcome of interest and COVID-19 infection. All parameters are expressed as hazard ratios (HRs) with 95% confidence intervals (95% CIs). Two-sided *P* values of 0.05 or below were considered indicative of statistical significance. We further stratified the analyses by sex and age group (i.e., < 18, 18–24, 25–44, 45–64, and 65 + years) to examine potential differences in associations related to sex and age [29, 30].

All analyses were conducted using statistical software R (version 4.2.0) [31]. The analysis packages were built on the open-source OHDSI *CohortMethod* and *Cyclops* R packages [26, 32, 33]. The study protocol and all statistical analysis packages were prespecified before the analysis. The data are reported following the Strengthening the Reporting of Observational Studies in Epidemiology (STROBE) reporting guideline [34].

### Role of the funding source

The funders of this study had no role in the study design, data collection, data analysis, interpretation, or writing of the report.

## Results

We initially identified 303,251 individuals with COVID-19 and 22,108,925 individuals without COVID-19 across five countries during the study period. Figure S1 in Supplement shows the study cohort selection procedure. After applying inclusion and exclusion criteria and conducting propensity score matching, the sample size varied by follow-up period, outcome, and database (Table 1). In the target cohorts, the majority of individuals included for the 6-month risk analysis were females and aged 45–64 years across all databases. The proportion of females ranged from 56.65% (*N* = 30,181) for substance misuse or dependence in France to 45.76% (1382) for personality disorders in the Germnay. The proportion of individuals aged 45–64 years ranged from 41.11% (3423) for personality disorders in Italy to 31.33% (11,131) for sleep disorders in the UK.

**Table 1:** Sample size, age and sex distribution, follow-up time, the number of psychiatric and neuropsychiatric events, and incidence rate in the short-term observation period by outcome and database

Outcome	Database	Targets/comparators	No. of individuals after propensity score matching	Sex, *N* (%)		Age, *N* (%)					No. of follow-up years	No. of events	Incidence rate per 1000 person years
				**Males**	**Females**	** < 18 years**	**18–24 years**	**25–44 years**	**45–64 years**	**65 + years**			
**Depression**	France IQVIA	Comparators	343,950	151,145 (43.94)	189,065 (54.97)	33,331 (9.69)	30,319 (8.81)	101,733 (29.58)	114,444 (33.27)	54,955 (15.98)	150,206	4163	27.72
		Targets	50,196	21,669 (43.17)	27,861 (55.50)	4486 (8.94)	4149 (8.27)	15,028 (29.94)	16,932 (33.73)	6861 (13.67)	22,669	1045	46.1
	Germany IQVIA	Comparators	124,315	56,551 (45.49)	65,494 (52.68)	13,664 (10.99)	11,294 (9.08)	33,791 (27.18)	38,624 (31.07)	20,252 (16.29)	54,703	3079	56.29
		Targets	18,044	8409 (46.60)	9566 (53.01)	1677 (9.29)	1710 (9.48)	5454 (30.23)	6257 (34.68)	2664 (14.76)	5296	626	75.46
	Italy IQVIA	Comparators	45,797	18,003 (39.31)	23,567 (51.46)	1068 (2.33)	2913 (6.36)	11,710 (25.57)	18,413 (40.21)	11,610 (25.35)	20,704	470	22.7
		Targets	7837	3067 (39.13)	3930 (50.15)	396 (5.05)	484 (6.18)	1968 (25.11)	3198 (40.81)	1767 (22.55)	3468	161	46.41
	UK IMRD	Comparators	306,950	132,653 (43.22)	172,172 (56.09)	35,363 (11.52)	30,667 (9.99)	95,572 (31.14)	95,302 (31.05)	42,316 (13.79)	135,034	1551	11.49
		Targets	35,262	15,344 (43.51)	19,890 (56.41)	3838 (10.88)	3611 (10.24)	10,912 (30.95)	11,094 (31.46)	5697 (16.16)	16,111	210	13.03
	US PharMetrics Plus	Comparators	587,963	280,670 (47.74)	301,241 (51.23)	65,337 (11.11)	72,829 (12.39)	188,202 (32.01)	204,110 (34.71)	38,619 (6.57)	250,800	15,763	62.85
		Targets	125,275	60,265 (48.11)	63,438 (50.64)	10,660 (8.51)	16,420 (13.11)	40,655 (32.45)	44,965 (35.89)	7311 (5.84)	56,516	3989	70.58
**Anxiety disorders**	France IQVIA	Comparators	337,181	148,415 (44.02)	185,012 (54.87)	32,970 (9.78)	29,404 (8.72)	98,591 (29.24)	112,670 (33.42)	54,495 (16.16)	146,417	7262	49.6
		Targets	49,004	21,210 (43.28)	27,133 (55.37)	4417 (9.01)	4008 (8.18)	14,488 (29.56)	16,636 (33.95)	6780 (13.84)	21,836	1867	85.5
	Germany IQVIA	Comparators	125,945	57,116 (45.35)	66,615 (52.89)	13,547 (10.76)	11,229 (8.92)	33,711 (26.77)	39,261 (31.17)	21,481 (17.06)	55,556	2816	50.69
		Targets	18,481	8554 (38.22)	9845 (53.27)	1673 (9.05)	1713 (9.27)	5507 (29.80)	6395 (34.60)	2825 (15.29)	8546	476	55.7
	Italy IQVIA	Comparators	48,516	18,543 (38.22)	25,568 (52.70)	1067 (2.20)	2920 (6.02)	11,984 (24.70)	19,433 (40.05)	12,954 (26.70)	22,046	102	4.63
		Targets	8334	3172 (38.06)	4281 (51.37)	376 (4.51)	484 (5.81)	2017 (24.20)	3394 (40.72)	1988 (23.85)	3733	41	10.98
	UK IMRD	Comparators	306,496	132,735 (43.31)	171,684 (56.02)	35,264 (11.51)	30,653 (10.00)	95,138 (31.04)	95,298 (31.09)	42,379 (13.83)	134,750	1656	12.29
		Targets	35,204	15,354 (43.61)	19,823 (56.31)	3828 (10.87)	3603 (10.23)	10,859 (30.85)	11,093 (31.51)	5714 (16.23)	16,068	265	16.49
	US PharMetrics Plus	Comparators	550,374	267,744 (48.65)	277,405 (50.40)	62,654 (11.38)	67,134 (12.20)	171,792 (31.21)	193,141 (35.09)	38,181 (6.94)	232,441	23,985	103.19
		Targets	117,948	57,768 (42.78)	58,625 (49.70)	10,270 (8.71)	15,263 (12.94)	37,434 (31.74)	42,648 (36.16)	7294 (6.18)	52,256	6802	130.16
**Alcohol misuse or dependence**	France IQVIA	Comparators	367,132	157,063 (42.03)	205,589 (56.00)	33,453 (9.11)	30,960 (8.43)	107,526 (29.29)	124,545 (33.92)	60,126 (16.38)	161,455	285	1.77
		Targets	53,556	22,508 (42.03)	30,321 (56.62)	4498 (8.40)	4230 (7.90)	15,924 (29.73)	18,501 (35.55)	7518 (14.04)	24,518	48	1.96
	Germany IQVIA	Comparators	132,126	59,061 (44.70)	70,785 (53.57)	13,779 (10.43)	11,743 (8.89)	35,608 (26.95)	41,467 (31.38)	22,292 (16.87)	59,066	225	3.81
		Targets	19,320	8832 (45.71)	10,401 (53.84)	1699 (8.79)	1788 (9.25)	5807 (30.06)	6729 (34.83)	2931 (15.17)	9082	18	1.98
	Italy IQVIA	Comparators	49,053	18,715 (38.15)	25,913 (52.83)	1135 (2.31)	2953 (6.02)	12,135 (24.74)	19,696 (40.15)	13,019 (26.54)	22,310	25	1.12
		Targets	8429	3196 (37.92)	4341 (51.50)	378 (4.48)	489 (5.80)	2042 (24.23)	3438 (40.79)	1998 (23.70)	3787	8	2.11
	UK IMRD	Comparators	311,033	133,857 (43.04)	175,097 (56.30)	35,421 (11.39)	31,407 (10.10)	97,301 (31.28)	96,272 (30.95)	42,713 (13.73)	137,067	166	1.21
		Targets	35,731	15,482 (43.33)	20,222 (56.60)	3844 (10.76)	3698 (10.35)	11,112 (31.10)	11,204 (31.36)	5761 (16.12)	16,369	18	1.1
	US PharMetrics Plus	Comparators	650,750	296,118 (45.50)	347,827 (53.45)	68,346 (10.50)	31,025 (4.77)	208,601 (32.06)	225,803 (34.70)	45,719 (7.03)	280,702	2187	7.79
		Targets	138,027	63,304 (45.86)	72,995 (52.88)	11,182 (8.10)	18,050 (13.08)	44,764 (32.43)	49,472 (35.84)	8803 (6.38)	63,223	510	8.07
**Substance misuse or dependence**	France IQVIA	Comparators	365,179	155,996 (42.72)	204,721 (56.06)	33,438 (9.16)	30,851 (8.45)	106,844 (29.26)	123,677 (33.87)	59,884 (16.40)	160,454	790	4.92
		Targets	53,277	22,352 (41.95)	30,181 (56.65)	4496 (8.44)	4220 (7.92)	15,813 (29.68)	18,364 (34.47)	7491 (14.06)	24,363	137	5.62
	Germany IQVIA	Comparators	130,475	58,150 (44.57)	70,055 (53.69)	13,767 (10.55)	11,659 (8.94)	35,071 (26.88)	40,856 (31.31)	22,049 (16.90)	58,168	789	13.56
		Targets	19,146	8730 (45.60)	10,327 (53.94)	1698 (8.87)	1779 (9.29)	5741 (29.99)	6660 (34.78)	2901 (15.15)	8978	96	10.69
	Italy IQVIA	Comparators	48,940	18,651 (37.71)	25,879 (52.88)	1089 (2.23)	2952 (6.03)	12,112 (24.75)	19,672 (40.20)	12,962 (26.49)	22,248	58	2.61
		Targets	8410	3188 (37.91)	4333 (51.52)	387 (4.60)	488 (5.80)	2039 (24.24)	3434 (40.83)	1990 (23.66)	3778	13	3.44
	UK IMRD	Comparators	310,792	133,683 (43.01)	175,049 (56.32)	35,410 (11.39)	31,356 (10.90)	97,241 (31.29)	96,226 (30.96)	42,678 (13.73)	13,6943	271	1.98
		Targets	35,708	15,462 (45.28)	20,219 (56.62)	3843 (10.76)	3693 (10.34)	11,105 (31.10)	11,200 (31.37)	5755 (16.12)	16,356	27	1.65
	US PharMetrics Plus	Comparators	621,083	281,202 (45.28)	333,365 (53.67)	67,865 (10.93)	78,144 (12.58)	198,313 (31.93)	213,416 (34.36)	43,549 (7.01)	266,538	9895	37.12
		Targets	132,328	60,379 (45.63)	70,237 (53.08)	11,107 (8.39)	17,435 (13.18)	42,736 (32.30)	46,994 (35.51)	8369 (6.32)	59,960	2653	44.25
**Bipolar disorders**	France IQVIA	Comparators	367,324	157,450 (45.78)	205,425 (59.73)	33,444 (9.72)	30,951 (9.00)	107,537 (31.27)	124,762 (36.27)	60,126 (17.48)	161,580	113	0.7
		Targets	53,580	22,553 (42.09)	30,299 (56.55)	4497 (8.39)	4231 (7.90)	15,927 (29.73)	18,527 (34.58)	7518 (14.03)	24,538	17	0.69
	Germany IQVIA	Comparators	132,427	59,271 (44.76)	70,871 (53.52)	13,782 (10.41)	11,736 (8.86)	35,712 (26.97)	41,644 (31.45)	22,335 (16.87)	59,244	40	0.68
		Targets	19,352	8856 (45.76)	10,408 (53.78)	1698 (8.77)	1790 (9.25)	5816 (30.05)	6739 (34.82)	2936 (15.17)	9101	< 5	< 0.55
	Italy IQVIA	Comparators	48,983	18,693 (38.16)	25,843 (52.76)	1088 (2.22)	2952 (6.03)	12,077 (24.66)	19,663 (40.14)	13,044 (26.63)	22,273	17	0.76
		Targets	8413	3196 (37.99)	4326 (51.42)	367 (4.36)	488 (5.80)	2035 (24.19)	3435 (40.83)	2000 (23.77)	3782	< 5	< 1.32
	UK IMRD	Comparators	311,440	134,066 (43.05)	175,264 (56.28)	35,421 (11.37)	31,415 (10.09)	97,459 (31.29)	96,449 (30.97)	42,754 (13.763)	137,275	40	0.29
		Targets	35,767	15,502 (43.34)	20,238 (56.58)	3844 (10.75)	3699 (10.34)	11,127 (31.11)	11,220 (31.37)	5765 (16.12)	16,390	8	0.49
	US PharMetrics Plus	Comparators	651,274	298,733 (45.87)	345,851 (53.10)	68,213 (10.47)	80,893 (12.42)	208,434 (32.00)	226,625 (34.80)	45,831 (7.04)	281,063	1382	4.92
		Targets	137,994	63,730 (46.18)	72,543 (52.60)	11,163 (8.09)	18,028 (13.06)	44,766 (32.44)	49,562 (35.92)	8817 (6.39)	63,265	307	4.85
**Psychoses**	France IQVIA	Comparators	367,222	157,237 (42.82)	205,529 (55.97)	33,452 (9.11)	30,949 (8.43)	107,493 (29.27)	124,791 (33.98)	60,072 (16.36)	16,1528	190	1.18
		Targets	53,576	22,531 (42.05)	30,312 (56.58)	4498 (8.40)	4230 (7.90)	15,921 (29.72)	18,529 (34.58)	7513 (14.02)	24,531	27	1.1
	Germany IQVIA	Comparators	132,002	59,113 (44.79)	70,571 (53.46)	13,771 (10.43)	11,723 (8.88)	35,634 (27.00)	41,507 (31.44)	22,116 (16.75)	59,031	172	2.91
		Targets	19,301	8836 (45.78)	10,376 (53.76)	1697 (8.79)	1788 (9.26)	5806 (30.08)	6735 (34.89)	2913 (15.09)	9075	11	1.21
	Italy IQVIA	Comparators	48,921	18,674 (38.17)	25,828 (52.80)	1075 (2.20)	2952 (6.03)	12,117 (24.77)	19,643 (40.15)	12,953 (26.48)	22,244	30	1.35
		Targets	8404	3189 (37.95)	4324 (51.45)	399 (4.75)	487 (5.79)	2038 (24.25)	3433 (40.85)	1988 (23.66)	3775	11	2.91
	UK IMRD	Comparators	311,286	134,004 (43.05)	175,190 (56.28)	35,411 (11.38)	31,403 (10.09)	97,472 (31.31)	96,417 (30.97)	42,695 (13.72)	137,187	111	0.81
		Targets	35,751	15,496 (43.34)	20,227 (56.58)	3842 (10.75)	3697 (10.34)	11,129 (31.13)	11,219 (31.38)	5751 (16.09)	16,384	11	0.67
	US PharMetrics Plus	Comparators	655,719	299,889 (45.73)	349,050 (53.23)	68,363 (10.43)	81,663 (12.45)	210,632 (31.12)	228,218 (34.80)	45,599 (6.95)	283,167	536	1.89
		Targets	138,882	63,981 (46.07)	73,175 (52.69)	11,187 (8.06)	18,163 (13.08)	45,099 (32.47)	49,922 (35.95)	8757 (6.31)	63,715	175	2.75
**Personality disorders**	France IQVIA	Comparators	367,882	157,565 (42.83)	205,832 (55.95)	33,444 (9.09)	30,951 (8.41)	107,663 (29.27)	125,103 (34.01)	60,257 (16.38)	161,848	60	0.37
		Targets	53,662	22,576 (42.07)	30,359 (56.57)	4497 (8.38)	4230 (7.88)	15,943 (29.71)	18,572 (34.61)	7531 (14.03)	24,576	13	0.53
	Germany IQVIA	Comparators	132,010	59,073 (44.75)	70,629 (53.50)	13,753 (10.42)	11,733 (8.89)	35,585 (26.96)	41,464 (31.41)	22,259 (16.86)	59,010	251	4.25
		Targets	19,305	8834 (45.76)	10,382 (45.76)	1697 (8.79)	1788 (9.26)	5800 (30.04)	6727 (34.85)	2930 (15.18)	9071	32	3.53
	Italy IQVIA	Comparators	48,481	18,536 (38.23)	25,590 (52.78)	1118 (2.31)	2952 (6.09)	12,111 (24.98)	19,608 (40.44)	12,589 (25.97)	22,025	152	6.9
		Targets	8327	3164 (38.00)	4284 (51.45)	396 (4.76)	489 (5.87)	2038 (24.47)	3423 (41.11)	1922 (23.08)	3739	39	10.43
	UK IMRD	Comparators	311,272	134,031 (43.06)	175,128 (56.26)	35,411 (11.38)	31,345 (10.07)	97,450 (31.31)	96,375 (30.96)	42,746 (13.73)	137,186	84	0.61
		Targets	35,753	15,500 (43.35)	20,227 (56.57)	3843 (10.75)	3691 (10.32)	11,130 (31.13)	11,214 (31.37)	5764 (16.12)	16,381	15	0.92
	US PharMetrics Plus	Comparators	656,469	300,441 (45.77)	349,260 (53.20)	68,259 (10.40)	81,435 (12.41)	210,520 (32.07)	228,569 (34.82)	46,260 (7.05)	283,419	570	2.01
		Targets	139,016	64,080 (46.10)	73,211 (52.66)	11,173 (8.04)	18,127 (13.04)	45,085 (32.43)	49,961 (35.94)	8907 (6.41)	63,793	78	1.22
**Self-harm and suicide**	France IQVIA	Comparators	367,961	157,640 (42.84)	205,853 (55.94)	33,443 (9.09)	30,958 (8.41)	107,701 (29.27)	125,091 (34.00)	60,280 (16.38)	161,887	44	0.27
		Targets	53,676	22,586 (42.08)	30,359 (56.56)	4496 (8.38)	4232 (7.88)	15,950 (29.72)	18,573 (34.60)	7535 (14.04)	24,584	< 10	< 0.41
	Germany IQVIA	Comparators	132,538	59,319 (44.76)	70,927 (53.51)	13,773 (10.39)	11,749 (8.86)	35,727 (26.96)	41,680 (31.45)	22,376 (16.88)	59,307	< 5	< 0.08
		Targets	19,366	8861 (45.76)	10,417 (53.79)	1698 (8.77)	1790 (9.24)	5819 (30.05)	6753 (34.87)	2940 (15.18)	9108	0	0
	Italy IQVIA	Comparators	49,144	18,755 (38.16)	25,943 (52.79)	1166 (2.37)	2953 (6.01)	12,148 (24.72)	19,721 (40.13)	13,071 (26.60)	22,353	0	0
		Targets	8444	3204 (37.94)	4345 (51.46)	400 (4.74)	489 (5.79)	2044 (24.21)	3446 (40.81)	2004 (23.73)	3795	0	0
	UK IMRD	Comparators	311,149	133,975 (43.06)	175,058 (56.26)	35,342 (11.36)	31,338 (10.07)	97,414 (31.31)	96,417 (30.99)	42,692 (13.72)	137,127	140	1.02
		Targets	35,732	15,493 (43.36)	20,212 (56.57)	3835 (10.73)	3690 (10.33)	11,121 (31.12)	11,218 (31.39)	5756 (14.11)	16,373	14	0.86
	US PharMetrics Plus	Comparators	657,634	300,838 (45.75)	350,011 (53.22)	68,341 (10.39)	81,747 (12.43)	211,021 (32.09)	228,782 (34.79)	46,327 (7.04)	283,995	243	0.86
		Targets	139,239	64,148 (46.07)	73,356 (52.68)	11,182 (8.03)	18,170 (13.05)	45,171 (32.44)	50,013 (35.92)	8928 (6.41)	63,889	69	1.08
**Sleep disorders**	France IQVIA	Comparators	350,031	151,096 (43.17)	195,052 (55.72)	33,106 (9.46)	30,350 (8.67)	104,273 (29.79)	117,993 (33.71)	54,745 (15.64)	152,814	4444	29.08
		Targets	51,025	21,635 (42.40)	28,672 (56.19)	4450 (8.72)	4142 (8.12)	15,385 (30.15)	17,476 (34.25)	6814 (13.35)	23,047	1056	45.82
	Germany IQVIA	Comparators	127,308	56,831 (44.64)	68,233 (53.60)	13,680 (10.75)	11,583 (9.10)	34,710 (27.26)	39,731 (31.21)	20,699 (16.26)	56,322	2182	38.74
		Targets	18,658	8505 (45.58)	10,027 (53.74)	1682 (9.01)	1770 (9.49)	5671 (30.39)	6466 (34.66)	2713 (14.54)	8679	360	41.48
	Italy IQVIA	Comparators	47,108	18,078 (38.38)	24,718 (52.47)	1124 (2.39)	2942 (6.25)	11,962 (25.39)	18,973 (40.28)	12,022 (25.52)	21,334	424	19.57
		Targets	8081	3085 (38.17)	4142 (51.26)	378 (4.68)	488 (6.04)	2011 (24.89)	3293 (40.75)	1850 (22.89)	3596	132	36.7
	UK IMRD	Comparators	309,291	133,171 (43.06)	174,083 (56.28)	35,342 (11.43)	31,294 (10.12)	86,887 (28.09)	95,649 (30.93)	42,224 (13.65)	136,147	902	6.63
		Targets	35,526	15,399 (43.35)	20,102 (56.58)	3837 (10.80)	3682 (10.36)	11,065 (31.15)	11,131 (31.33)	5699 (16.04)	16,254	132	8.12
	US PharMetrics Plus	Comparators	592,830	269,300 (45.43)	317,217 (53.51)	66,595 (11.23)	79,047 (13.33)	195,609 (33.00)	195,813 (33.03)	37,291 (6.29)	252,186	16,962	67.26
		Targets	126,034	57,857 (45.91)	66,575 (52.82)	10,911 (8.66)	17,643 (14.00)	42,013 (33.33)	43,026 (34.14)	7191 (5.71)	56,538	4802	84.87
**Dementia**	France IQVIA	Comparators	367,141	157,365 (42.86)	205,349 (55.93)	33,454 (9.11)	30,962 (8.43)	107,694 (29.33)	125,094 (34.07)	59,533 (16.22)	161,555	87	0.54
		Targets	53,582	22,558 (42.10)	30,295 (56.54)	4498 (8.39)	4233 (7.90)	15,950 (29.77)	18,571 (34.66)	7450 (13.90)	24,544	23	0.94
	Germany IQVIA	Comparators	130,548	58,726 (44.98)	69,559 (53.28)	13,782 (10.56)	11,748 (9.00)	35,711 (27.35)	41,585 (31.85)	20,516 (15.72)	58,364	387	6.63
		Targets	19,132	8794 (45.96)	10,251 (53.58)	1697 (8.87)	1790 (9.36)	5818 (30.41)	6743 (35.24)	2719 (14.21)	8999	50	5.56
	Italy IQVIA	Comparators	48,815	18,660 (38.23)	25,733 (52.72)	1200 (2.46)	2953 (6.05)	12,148 (24.89)	19,720 (40.40)	12,727 (26.07)	22,204	57	2.57
		Targets	8391	3189 (38.00)	4312 (51.39)	399 (4.76)	489 (5.83)	2044 (24.36)	3446 (41.07)	1957 (23.32)	3777	8	2.12
	UK IMRD	Comparators	307,701	132,779 (43.15)	172,882 (56.19)	35,411 (11.51)	31,413 (10.21)	97,502 (31.69)	96,370 (31.32)	39,552 (12.85)	135,856	471	3.47
		Targets	35,263	15,304 (43.40)	19,932 (56.52)	3841 (10.89)	3699 (10.49)	11,133 (31.57)	11,212 (31.80)	5262 (14.92)	16,203	125	7.71
	US PharMetrics Plus	Comparators	649,692	298,235 (45.90)	345,059 (53.11)	68,330 (10.52)	81,713 (12.58)	210,633 (32.42)	229,161 (35.27)	41,290 (6.36)	280,786	1114	3.97
		Targets	137,741	63,632 (46.20)	72,393 (52.56)	11,177 (8.11)	18,166 (13.19)	45,117 (32.75)	49,898 (36.23)	7738 (5.62)	63,243	384	6.07
**Neurodevelopmental disorders**	France IQVIA	Comparators	367,279	157,233 (42.81)	205,574 (55.97)	33,106 (9.01)	30,873 (8.41)	107,624 (29.30)	125,034 (34.04)	60,188 (16.39)	161,542	215	1.33
		Targets	53,581	22,535 (42.06)	30,317 (56.58)	4453 (8.31)	4219 (7.87)	15,938 (29.75)	18,559 (34.64)	7525 (14.04)	24,523	75	3.06
	Germany IQVIA	Comparators	130,731	58,345 (44.63)	70,193 (53.69)	12,802 (9.79)	11,684 (8.94)	35,627 (27.25)	41,574 (31.80)	22,126 (16.92)	58,330	643	11.02
		Targets	19,181	8754 (45.64)	10,340 (53.91)	1578 (8.23)	1775 (9.25)	5807 (30.27)	6740 (35.14)	2914 (15.19)	9003	68	7.55
	Italy IQVIA	Comparators	49,087	18,733 (38.16)	25,918 (52.80)	1176 (2.40)	2950 (6.01)	12,134 (24.72)	19,697 (40.13)	13,068 (26.62)	22,325	22	0.99
		Targets	8424	3195 (37.93)	4337 (51.48)	405 (4.81)	483 (5.73)	2040 (24.22)	3443 (40.87)	2001 (23.75)	3786	6	1.58
	UK IMRD	Comparators	310,702	133,535 (42.98)	175,062 (56.34)	35,124 (11.30)	31,317 (10.08)	97,332 (31.33)	96,350 (31.01)	42,682 (13.74)	136,903	384	2.8
		Targets	35,694	15,447 (43.28)	20,221 (56.65)	3813 (10.68)	3689 (10.34)	11,114 (31.14)	11,212 (31.41)	5754 (16.12)	16,352	34	2.08
	US PharMetrics Plus	Comparators	630,465	287,575 (45.61)	336,474 (53.37)	62,324 (9.89)	75,937 (12.04)	201,789 (32.01)	224,114 (35.55)	45,506 (7.22)	271,166	4825	17.79
		Targets	133,869	61,653 (46.05)	70,521 (52.68)	10,254 (7.66)	16,960 (12.67)	43,330 (32.37)	49,097 (36.68)	8751 (6.54)	61,137	1129	18.47
**Overall**	France IQVIA	Comparators	304,558	136,940 (44.96)	164,901 (54.14)	32,186 (10.57)	28,370 (9.32)	91,590 (30.07)	99,294 (32.60)	45,660 (14.99)	130,911	11,413	87.18
		Targets	44,390	19,614 (44.19)	24,173 (54.46)	4318 (9.73)	3873 (8.72)	13,393 (30.17)	14,600 (32.89)	5685 (12.81)	19,394	2909	149.99
	Germany IQVIA	Comparators	111,888	51,093 (45.66)	58,863 (52.61)	12,460 (11.14)	10,633 (9.50)	31,121 (27.81)	35,003 (31.28)	17,060 (15.25)	48,094	6674	138.77
		Targets	16,528	7752 (46.90)	8708 (52.69)	1527 (9.24)	1618 (9.79)	5059 (30.61)	5754 (34.81)	2277 (13.78)	7408	1233	166.43
	Italy IQVIA	Comparators	42,485	16,783 (39.50)	21,786 (51.28)	1044 (2.46)	2873 (6.76)	11,293 (26.58)	17,334 (40.80)	9876 (23.25)	19,103	995	52.09
		Targets	7252	2864 (39.49)	3630 (50.06)	356 (4.91)	472 (6.51)	1889 (26.05)	2991 (41.24)	1509 (20.81)	3167	325	102.59
	UK IMRD	Comparators	296,306	128,703 (43.44)	165,704 (55.92)	34,785 (11.74)	29,970 (10.11)	92,966 (31.37)	93,187 (31.45)	38,332 (12.94)	130,123	4210	32.35
		Targets	33,989	14,851 (43.69)	19,108 (56.22)	3775 (11.11)	3526 (10.37)	10,618 (31.24)	10,867 (31.97)	5094 (14.99)	15,500	631	40.71
	US PharMetrics Plus	Comparators	453,727	218,987 (48.26)	230,194 (50.73)	56,371 (12.42)	58,638 (12.92)	146,413 (32.27)	152,990 (33.72)	25,942 (5.72)	188,242	36,020	191.35
		Targets	98,366	48,070 (48.87)	48,919 (49.73)	9265 (9.42)	13,538 (13.76)	32,248 (32.78)	34,156 (34.72)	4866 (4.95)	42,281	10,292	243.41

The short-term (6 months) incidence rate was generally high for anxiety disorders, depression, and sleep disorders. The highest incidence of 130.16 per 1000 person-years was observed among individuals with COVID-19 for anxiety disorders in the USA, followed by 85.5 per 1000 person-years for anxiety disorders in France and 84.87 per 1000 person-years for sleep disorders in the USA. No self-harm and suicide cases were observed in Germany and Italy. Additional file 1: Tables S4–S5 show the sample size, sex and age distribution, follow-up time, and number and incidence of psychiatric and neuropsychiatric events for the medium- and long-term observation periods. The incidence of outcomes in the medium- and long-term observation periods was lower than the short-term results. The incidence of any outcome among individuals with COVID-19 for the short-, medium-, and long-term periods ranged from 40.71, 28.73, and 27.67 per 1000 person-years in the UK, to 243.41, 144.11, and 119.95 per 1000 person-years in the USA, respectively.

Table 2 shows selected baseline characteristics before and after propensity score matching for France IQVIA, using short-term depression risk as an example. Before propensity score matching, individuals with COVID-19 were more likely to have acute respiratory disease and use antibacterial, anti-inflammatory, antirheumatic, and opioid products, with standardized difference of mean up to 0.77. After propensity score matching, all standardized differences were less than 0.2, and most were less than 0.1, indicating that the samples of individuals with and without COVID-19 were well-balanced after matching. Additional file 1: Tables S6.1–S6.179 show the baseline characteristics for all outcomes in all databases, which had similar results.

**Table 2:** Selected baseline characteristics for France IQVIA, using short-term depression risk as an example

Characteristic	Before propensity score matching			After propensity score matching		
	**Targets, %**	**Comparators, %**	**Standardized difference**	**Targets, %**	**Comparators, %**	**Standardized difference**
**Age group**						
0–4	0.7	2.9	− 0.17	0.8	1.2	− 0.04
5–9	1.8	4.4	− 0.15	1.9	2.3	− 0.03
10–14	3.2	4.4	− 0.06	3.4	3.7	− 0.01
15–19	5.6	4.3	0.06	5.6	5.9	− 0.01
20–24	7.3	4.2	0.13	7.1	6.5	0.02
25–29	7.3	4.3	0.13	7	6.7	0.01
30–34	8.3	5.1	0.13	8.1	7.7	0.02
35–39	9	5.6	0.13	8.6	7.9	0.03
40–44	9.4	5.9	0.13	9.1	8.3	0.03
45–49	10.1	6.8	0.12	9.9	9.2	0.02
50–54	9.8	7.5	0.08	9.6	9.4	0.01
55–59	8.9	7.8	0.04	8.9	9	0
65–69	4	7.7	− 0.16	4.3	4.7	− 0.02
70–74	3.7	8	− 0.19	4	4.6	− 0.03
75–79	2	5.2	− 0.17	2.2	2.7	− 0.03
80–84	1.3	4	− 0.17	1.5	1.8	− 0.02
85–89	0.9	2.6	− 0.13	1	1.3	− 0.03
90–94	0.4	1.1	− 0.08	0.4	0.6	− 0.03
95–99	0.1	0.2	− 0.03	0.1	0.2	− 0.01
**Sex**						
Female	56.4	57.4	− 0.02	55.9	55	0.02
**Medical history: general**						
Acute respiratory disease	16.7	6.9	0.31	15.7	18.7	− 0.08
Chronic liver disease	0.1	0.1	0.01	0.1	0.1	− 0.01
Chronic obstructive lung disease	0.9	0.7	0.02	0.9	1.3	− 0.04
Crohn’s disease	0.1	0.1	0.01	0.1	0.1	0
Dementia	0.1	0.1	0	0.2	0.2	− 0.02
Depressive disorder	6.1	3.2	0.14	0.2	0.1	0.04
Diabetes mellitus	5.2	4	0.06	5.4	6.8	− 0.06
Gastroesophageal reflux disease	4.3	2	0.13	4.2	5.4	− 0.06
Gastrointestinal hemorrhage	0.5	0.3	0.04	0.4	0.6	− 0.02
Human immunodeficiency virus infection	0.2	0.1	0.03	0.2	0.4	− 0.04
Hyperlipidemia	4	3	0.06	4	4.8	− 0.04
Hypertensive disorder	11.7	9.9	0.06	12.1	15.6	− 0.1
Lesion of liver	0.1	0.1	0	0.1	0.1	− 0.01
Obesity	0.4	0.1	0.05	0.3	0.3	0
Osteoarthritis	3.8	2.2	0.09	3.6	4.5	− 0.05
Pneumonia	0.8	0.3	0.07	0.7	1	− 0.03
Psoriasis	1	0.5	0.05	1	1.1	− 0.02
Renal impairment	0.3	0.2	0.02	0.3	0.4	− 0.02
Rheumatoid arthritis	0.2	0.3	− 0.01	0.3	0.3	− 0.02
Schizophrenia	0.1	0.1	0	0.1	0.1	0
Ulcerative colitis	0.1	0.1	0.01	0.1	0.1	0
Urinary tract infectious disease	1.5	0.7	0.07	1.4	1.9	− 0.03
**Medical history (cardiovascular disease)**						
Atrial fibrillation	0.1	0.3	− 0.03	0.2	0.2	− 0.02
Cerebrovascular disease	0.9	0.8	0.02	1	1.4	− 0.04
Coronary arteriosclerosis	0.4	0.4	0	0.4	0.6	− 0.03
Heart disease	3.3	3.6	− 0.02	3.4	4.7	− 0.07
Heart failure	0.3	0.3	− 0.01	0.3	0.4	− 0.02
Ischemic heart disease	1	0.9	0	1.1	1.5	− 0.03
Peripheral vascular disease	0.2	0.2	0	0.2	0.4	− 0.03
**Medical history (neoplasms)**	0.2	0.1	0.01	0.2	0.3	− 0.02
Venous thrombosis	0.3	0.2	0.03	0.3	0.4	− 0.02
Medical history: Neoplasms						
Malignant neoplastic disease	1.1	0.8	0.03	1.2	1.7	− 0.04
Malignant tumor of breast	0.3	0.2	0.02	0.3	0.4	− 0.02
Malignant tumor of colon	0.2	0.1	0.03	0.2	0.1	0
Primary malignant neoplasm of prostate	0.2	0.1	0.01	0.2	0.3	− 0.03
**Medication use**						
Agents acting on the renin-angiotensin system	10.5	10	0.02	11.1	14.2	− 0.09
Antibacterials for systemic use	36.9	16.7	0.47	34.7	39.4	− 0.1
Antidepressants	7.3	4.7	0.11	3.5	4.3	− 0.04
Antiepileptics	2.4	2	0.03	2.2	3.3	− 0.06
Antiinflammatory and antirheumatic products	35.2	20.3	0.34	35.3	40.1	− 0.1
Antineoplastic agents	1	0.8	0.02	1	1.2	− 0.01
Antipsoriatics	0.5	0.3	0.03	0.5	0.7	− 0.02
Antithrombotic agents	8	7.6	0.01	8.3	10.6	− 0.08
Beta blocking agents	6.3	6.4	0	6.5	8.1	− 0.06
Calcium channel blockers	5.8	5.3	0.02	6.1	7.9	− 0.07
**Medication use**						
Diuretics	5.3	5.5	− 0.01	5.6	7.4	− 0.07
Drugs for acid-related disorders	24.5	14.2	0.26	24.1	28.2	− 0.09
Drugs for obstructive airway diseases	23.1	12.6	0.28	22.7	26.8	− 0.09
Drugs used in diabetes	5.5	4.3	0.05	5.7	7.2	− 0.06
Immunosuppressants	0.3	0.5	− 0.03	0.3	0.4	− 0.01
Opioids	58.5	23.4	0.77	56.1	57.9	− 0.04
Psycholeptics	15	8.7	0.2	12.6	15.2	− 0.07
Psychostimulants, agents used for ADHD and nootropics	4.5	2	0.14	4.2	5	− 0.03

**Fig. 1:**
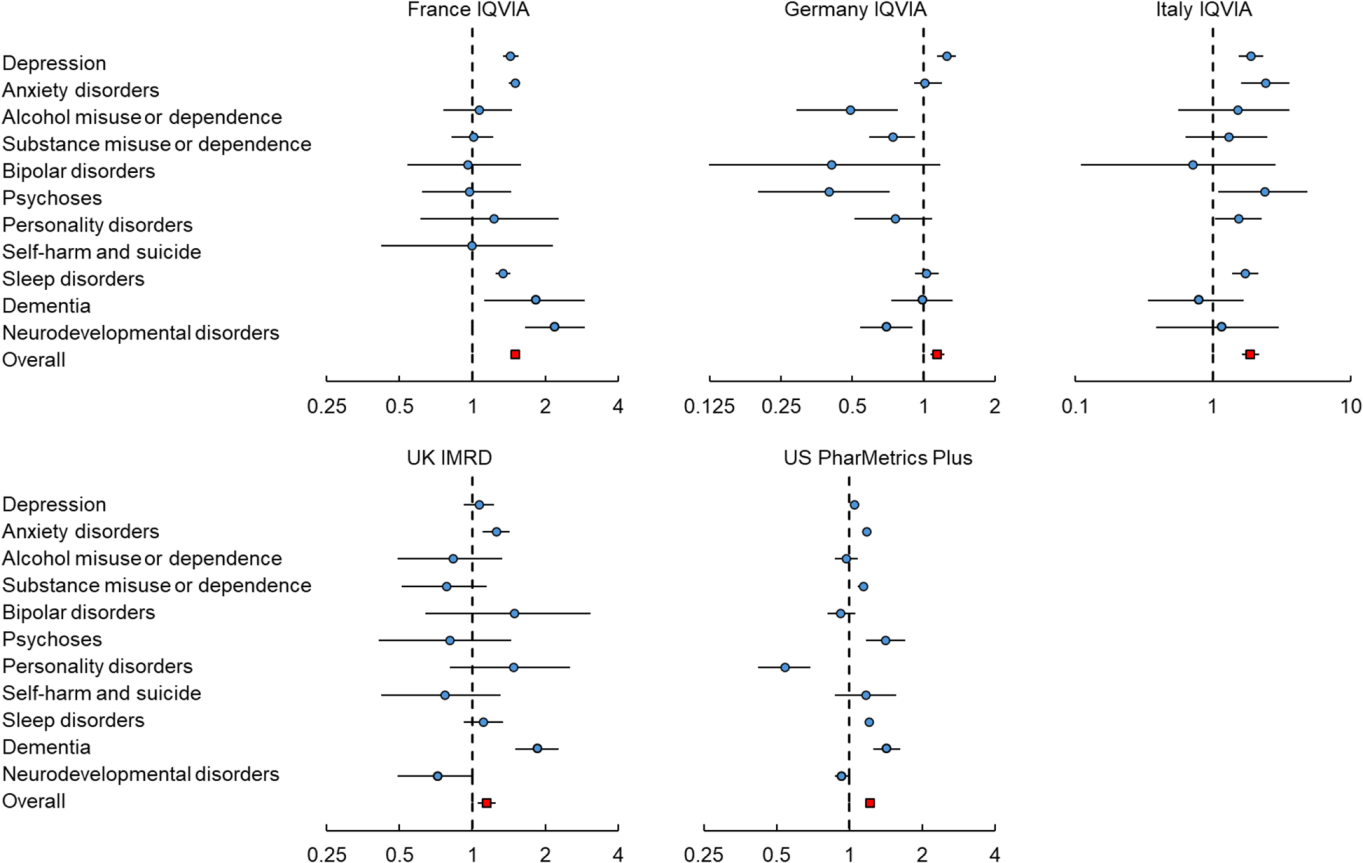
Risk of developing outcome events among individuals with COVID-19 in the short-term observation period (within 6 months)

Figure 1 and Additional file 1: Table S7 show the short-term risks of psychiatric and neuropsychiatric outcomes. During the first 6 months following the index date, individuals with COVID-19 had a significantly higher risk of developing any psychiatric and neuropsychiatric disorder than individuals without COVID-19 across all databases. The HRs ranged from 1.14 (95% CI, 1.07–1.22) in Germany to 1.89 (1.64–2.17) in Italy. Specifically, the risk of depression was higher in individuals with COVID-19 in all databases except for the UK, with HRs ranging from 1.05 (1.01–1.09) in the USA to 1.90 in Italy (1.55–2.31). Significant HRs for anxiety disorders were found in all databases except for Germany, ranging from 1.18 (1.14–1.21) in the USA to 2.43 (1.61–3.61) in Italy. An increased risk of psychoses was observed in Italy (HR 2.39, 95% CI 1.09–4.85) and the USA (1.41, 1.17–1.70). Increased risks of substance misuse or dependence and personality disorders were found among individuals with COVID-19 in the USA (1.14, 1.08–1.19) and Italy (1.55, 1.04–2.25), respectively. Additionally, COVID-19 was associated with an elevated risk of sleep disorders in France (1.34, 1.25–1.44), Italy (1.73, 1.39–2.14), and the USA (1.21, 1.16–1.25). Individuals with COVID-19 had an increased risk of dementia in the UK (1.86, 1.50–2.28), France (1.84, 1.12-2.91), and the USA (1.43, 1.26–1.63). A significant HR for neurodevelopmental disorders was observed only in France (2.20, 1.65–2.91).

**Fig. 2:**
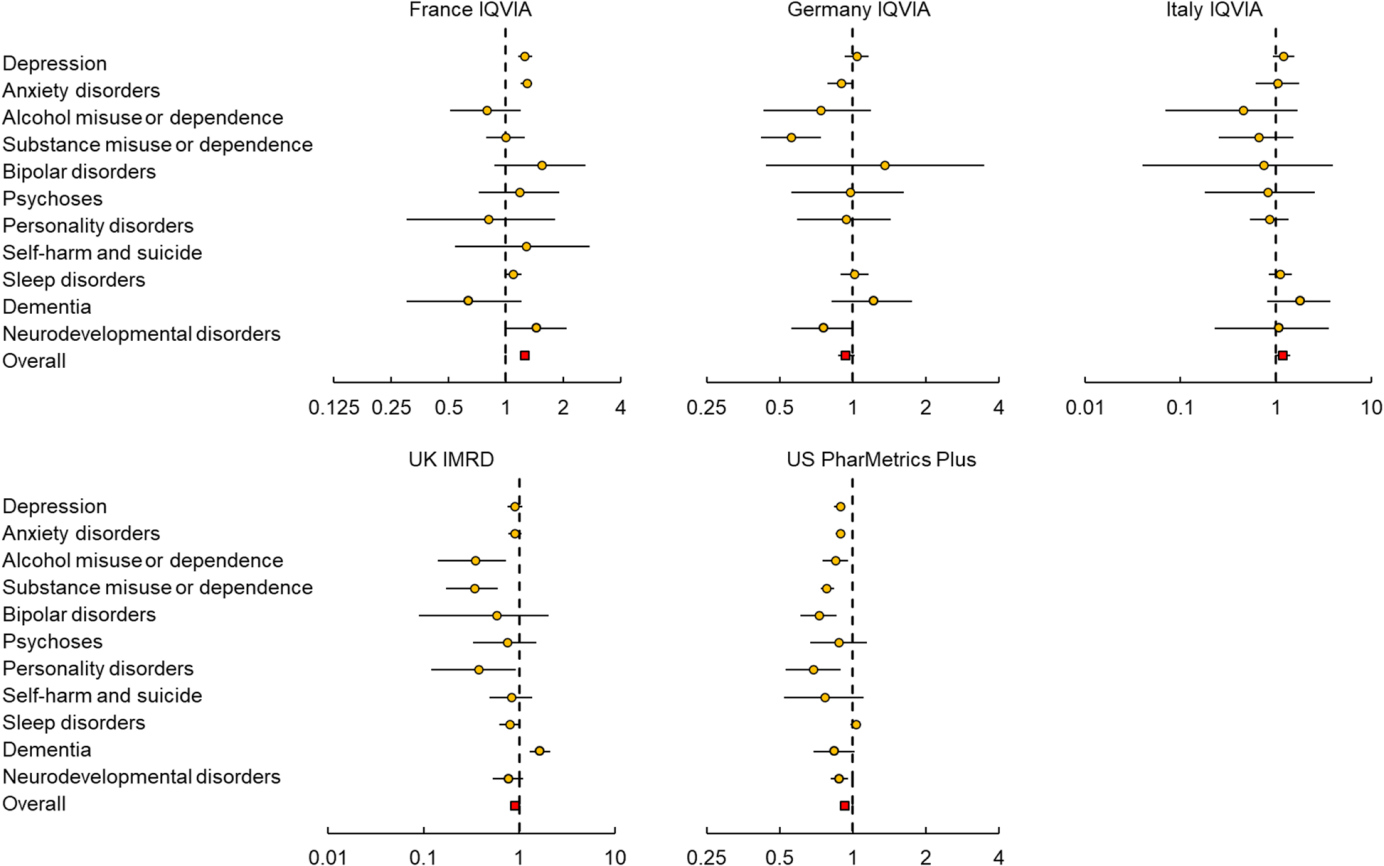
Risk of developing outcome events among individuals with COVID-19 in the medium-term observation period (6 months to 1 year)

**Fig. 3:**
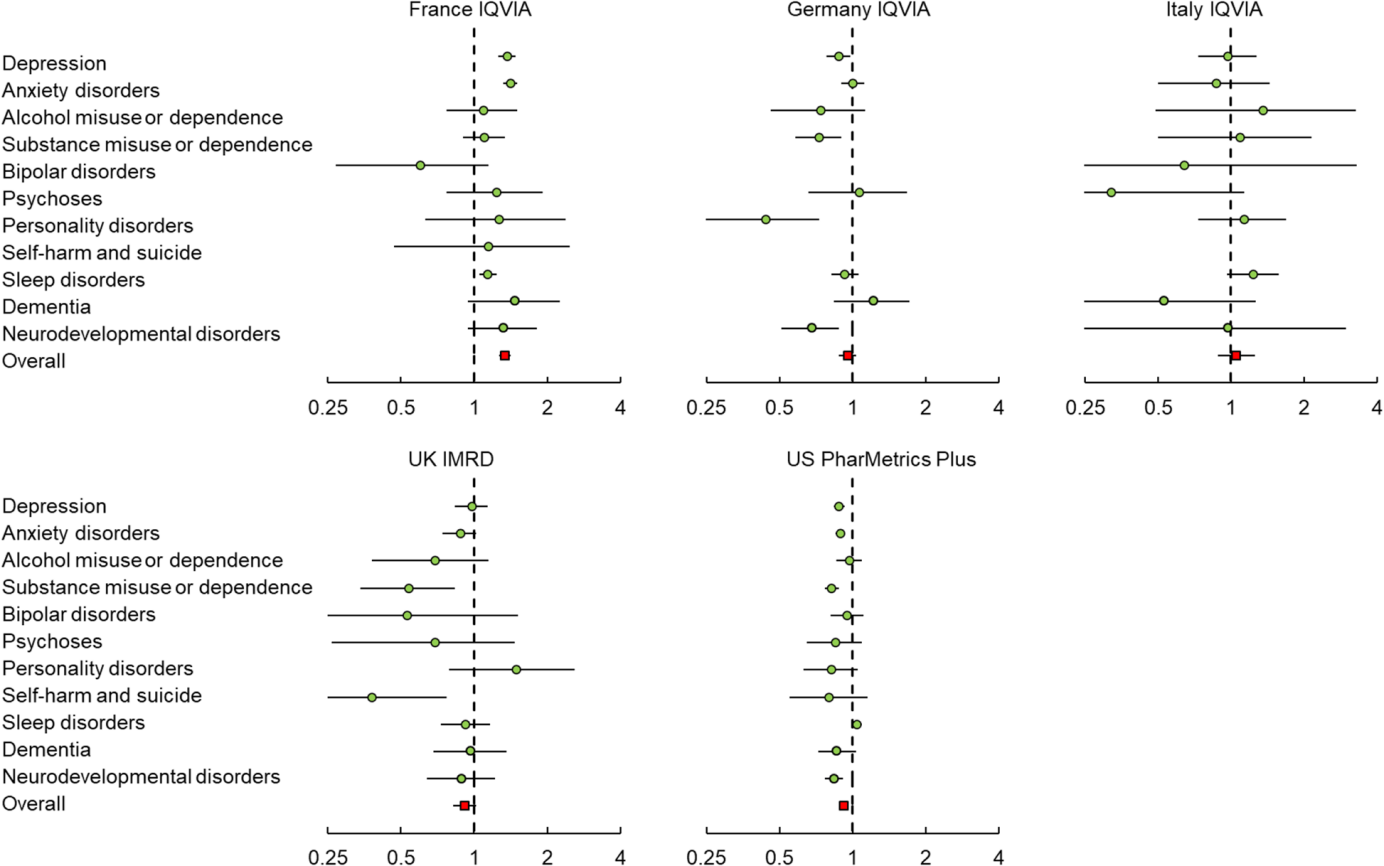
Risk of developing outcome events among individuals with COVID-19 in the long-term observation period (1 year to 2 years)

No significant associations were observed between COVID-19 and any of the psychiatric and neuropsychiatric disorders in the medium-term (6 months to 1 year) or long-term (1 to 2 years) periods, except for France (medium-term: HR 1.26, 95% CI 1.19–1.34; long-term: 1.34, 1.27–1.41) (see Figs. 2 and 3 and Additional file 1: Table S7). In France IQVIA, individuals with COVID-19 had a higher risk of depression (HR 1.29, 95% CI 1.18–1.41), anxiety disorders (1.29, 1.20–1.38), and sleep disorders (1.10, 1.01–1.21) during the medium-term observation, compared to matched comparators. Additionally, in the UK, an elevated risk of dementia (1.65, 1.28–2.10) was observed during the medium-term. In the long-term, elevated risks were only observed for depression (1.36, 1.25–1.47), anxiety disorders (1.37, 1.29–1.47), and sleep disorders (1.14, 1.05–1.24) in France.

Additional file 1: Tables S8–S14 show the results of subgroup analyses. The risk of psychiatric and neuropsychiatric disorders associated with COVID-19 varied by sex and age group. For example, in the UK, there was an increased short-term risk of anxiety disorders (HR 1.59, 95% CI 1.24–2.01) and bipolar disorders (5.15, 1.35–17.09) among males, but not females.

Additionally, a significantly higher short-term risk of substance misuse or dependence (HR 1.26, 1.18–1.34) was observed only among males in the USA. In age group-stratified analyses for short-, medium-, and long-term risks, significant HRs for sleep disorders were only observed among individuals aged 25 years or older in France, Italy, and the USA. An elevated risk of substance misuse or dependence was observed among individuals aged 18–44 years and those aged 65 years or older in Italy and the USA.

## Discussion

In this multinational network study using population-based electronic medical records and claims data from four European countries and the USA, we compared the short-, medium-, and long-term risks of 11 psychiatric and neuropsychiatric disorders of individuals with and without COVID-19. During the first 6 months post-infection, an overall increased risk of psychiatric and neuropsychiatric disorders was evident across all databases, with increased risks consistently observed in depression, anxiety disorders, and sleep disorders. Increased risks were only observed for depression, anxiety disorders, sleep disorders in France, and dementia in the UK, in the medium- and long-term observation periods.

Previous studies have consistently demonstrated that a considerable number of individuals infected with COVID-19 suffer from mental and neurological health issues extending weeks to months beyond the acute phase of the illness. For instance, a meta-analysis of 51 studies involving approximately 19,000 subjects found that 27.4% of COVID-19 survivors experienced sleep disorders, 20.2% had cognitive impairment, 19.1% had anxiety disorders, and 12.9% had depression following the infection [35]. Furthermore, studies from Denmark, Estonia, Iceland, Norway, Sweden, the UK, and the USA indicated an increased risk of psychiatric and neuropsychiatric sequelae in COVID-19 survivors compared to those uninfected by the virus or those suffering from influenza or other respiratory infections [9, 12–14]. However, most of these studies had a follow-up period of less than 1 year, thus not necessarily capturing the potential long-term consequences or accounting for the possibility of delayed help-seeking due to barriers in accessing mental health services. Additionally, the applicability of these findings may be limited by variations in how the index date is determined and the absence of standardized measurements or tools for identifying outcomes [36, 37]. By applying standardized definitions and analytical codes across five population-based databases, our study contributes multinational, large-scale evidence on the psychiatric and neuropsychiatric consequences associated with COVID-19 during a 2-year post-infection timeframe.

Our study found that individuals with COVID-19 showed an increased likelihood of developing psychiatric and neuropsychiatric disorders within the 6 months following infection, consistent with existing research on COVID [9, 12–14], mirroring outcomes observed during past coronaviruses outbreaks [8], and supporting the direct and indirect impact of COVID-19 on mental and neuropsychiatric health. While some studies have suggested that immune dysfunction, including nonspecific neuroinflammation and antineural autoimmune dysregulation, may be associated with the psychiatric and neuropsychiatric sequelae of SARS-CoV-2 during the acute phase of the disease [38–40], the pathophysiological mechanisms underlying these manifestations still remain poorly understood. Additionally, pandemic-related psychosocial stressors, such as unprecedented social isolation due to lockdowns and quarantine, financial insecurity from economic downturns, and grief over the loss of loved ones might have contributed to psychiatric symptoms, although the impact should be similar between individuals with and without COVID-19 [41–45]. It is worth noting that our study focused on individuals infected by the early strain of COVID-19 in 2020, a period associated with greater disease severity [46]. The fear of illness, lack of vaccines, disrupted daily routines, and stigma associated with COVID-19 infection leading to social ostracization, further contributed to the psychological burden and heightened the risk of psychiatric manifestations among individuals with COVID-19 [47, 48].

While our investigation revealed several psychiatric and neuropsychiatric sequelae of COVID-19 in the 6 months following infection, significant differences in risk were observed only for depression, anxiety disorders and sleep disorders in France, and dementia in the UK during the medium- and long-term observation periods. This finding is partly consistent with a previous USA study, which reported that the risks of mood disorders, anxiety disorders, and insomnia associated with COVID-19 decreased 1 to 3 months after the infection, while the risks of dementia and psychotic disorder remained elevated after 2 years [15]. The attenuation of risk can be attributed to several reasons. First, individuals with an incident diagnosis of psychiatric and neuropsychiatric disorder during the first 6 months post-infection were excluded from the mid- and long-term analyses. Therefore, the risks after 6 months might be lower if an individual already “survived” the acute phase of the pandemic. Second, physical status gradually recovers over time, which includes the alleviation of the virus’s direct neurological effects that may have initially led to such disorders [49, 50]. Third, as the pandemic progressed, individuals adapted to the psychosocial stressors triggered by the pandemic. People have found appropriate coping mechanisms and adjusted to changed circumstances, reducing the impact of these stressors on mental health [51, 52]. Fourth, as public health measures control the spread of the virus and vaccines become widely available, the initial fear and uncertainty surrounding COVID-19 and its consequences diminish. Additionally, the implementation of specialized mental health services for COVID-19 survivors helps address and mitigate the long-term psychiatric and neuropsychiatric effects of the virus. However, explaining the considerable heterogeneity in relative risks between countries remains challenging, as the mechanisms underlying the association between COVID-19 infection and psychiatric and neuropsychiatric outcomes are not yet fully understood [38–40]. The significant medium- and long-term increased risks observed in France and the UK may reflect the diverse impacts of differences in COVID-19 containment strategies, health systems, and rates of socioeconomic recovery by country, rather than the pandemic itself. Furthermore, the results may be partially influenced by detection bias, given the severe underdiagnosis and considerable diagnostic delays associated with psychiatric and neuropsychiatric disorders. For instance, the longer effect on dementia observed in the UK could simply result from longer waiting times for dementia assessments. Further research is required to explore the causes of this discrepancy to mitigate potential long-lasting impacts.

Our study has several strengths. By utilizing data from over 25 million individuals across five countries and diverse healthcare settings, we have enabled a comprehensive investigation into the short-, medium-, and long-term psychiatric and neuropsychiatric sequelae of COVID-19. These enhance the precision, representativeness, and generalizability of our findings. To minimize potential confounding, we incorporated a significant amount of covariates into a large-scale regularized regression for covariate selection and propensity score calculation. Furthermore, using the OMOP CDM allowed us to standardize the study design, outcome definition, and analytical syntax within participating data partners. This standardization streamlined the process of generating and sharing results without disclosing individual-level data. It is important to note that our intention is not to compare the incidence or risks among different populations and healthcare systems, as database-specific properties are not comparable. Additionally, we have made the study package publicly available to encourage the reproduction of our findings and to foster collaboration.

This study has limitations. First, phenotype ascertainment may be affected by the inherent measurement issues within our real-world databases. Although we used standardized diagnostic codes to identify outcomes, heterogeneity in diagnostic accuracy and coding processes across different healthcare settings persisted. Moreover, the specific diagnostic codes within the original data sources have not been validated, and their sensitivity and specificity require further exploration [53]. Second, electronic medical record databases from four European countries (France, Italy, Germany, and the UK) were collected from primary care settings, which means inpatient records were not available for this study. Consequently, we could not capture outcomes diagnosed during hospital admissions. Third, several factors associated with the sequelae of COVID-19, such as infection severity, vaccination, and mortality, were not available in our databases, preventing detailed investigations into the effects of these factors on COVID-19 sequelae. Fourth, individuals in the comparator group might have had undiagnosed COVID-19 infection. To minimize the misclassification effect, we included all individuals with a COVID-19 diagnosis in healthcare institutions and positive SARS-CoV-2 screening test results from lab tests and adjusted the coding list for COVID-19 individuals’ ascertainment during the data testing process. Fifth, we only considered incident psychiatric and neuropsychiatric diagnoses. Individuals who sought help repeatedly (with prolonged and more severe symptoms) were excluded from the mid- and long-term analysis, potentially resulting in an underestimation of the results. Sixth, our study only included unvaccinated individuals infected with COVID-19 in 2020, most of whom likely had no prior infection. Caution is warranted when generalizing our findings to other populations, as clinical consequences may differ between primary infections, reinfections, and breakthrough infections. Seventh, due to dataset limitations, we were only able to use a 1-year look-back period to exclude prevalent cases. As a result, some included individuals may not be “true” incident cases. Eighth, this study aimed to provide an overview of the association between psychiatric and neuropsychiatric disorders and COVID-19 across multiple databases, grouping outcomes of interest into 11 relatively broad disease categories. This approach was intended to ensure sufficient statistical power in each database and to simply reporting. However, it is possible that specific conditions within a category may have distinct associations with COVID-19, warranting future investigations. Finally, as with other observational studies, residual confounding was likely present in our study. For instance, individuals without COVID-19 infection were less likely than those with COVID-19 to present at healthcare institutions during the pandemic, reducing the probability of receiving diagnoses for other conditions and potentially overestimating our results.

## Conclusions

In this multinational network cohort study examining the short- (6 months), medium- (6 months to 1 year), and long-term (1 to 2 years) psychiatric and neuropsychiatric sequelae of COVID-19, we consistently observed short-term risks of these conditions associated with COVID-19 across different countries. Notably, there were very few differences in these risks between individuals with and without COVID-19 infection after 6 months. This phenomenon may be attributed to detection bias, the redirection of healthcare resources, diverse pandemic management strategies, and differing rates of socioeconomic recovery rather than the direct impact of COVID-19 itself. These findings underscore the complexity of the pandemic’s indirect effects on mental health. Further research is essential to elucidate the underlying causes of the medium- and long-term elevated risks of psychiatric and neuropsychiatric outcomes observed in France and the UK. Understanding these factors is crucial for developing targeted interventions and healthcare policies to mitigate these long-lasting impacts.

## Supplementary Information

Additional file 1: Fig. S1. Study cohort selection procedure, using short-term depression risk in France IQVIA as an example. Table S1. Descriptions of databases. Table S2. Diagnostic codes for COVID-19 identification. Table S3. Diagnostic codes for outcomes identification. Table S4. Sample size, sex and age distribution, follow-up time, the number of psychiatric and neuropsychiatric events, and incidence rate in the medium-term observation period by outcome and database. Table S5. Sample size, sex and age distribution, follow-up time, the number of psychiatric and neuropsychiatric events, and incidence rate in the long-term observation period by outcome and database. Table S6. Selected baseline characteristics for all outcomes in all databases. Table S7. Risk of developing outcome events among individuals with COVID-19 for the whole study sample. Table S8. Risk of developing outcome events among individuals with COVID-19 for males. Table S9. Risk of developing outcome events among individuals with COVID-19 for females. Table S10. Risk of developing outcome events among individuals with COVID-19 for individuals aged below 18 years. Table S11. Risk of developing outcome events among individuals with COVID-19 for individuals aged between 18 and 24 years. Table S12. Risk of developing outcome events among individuals with COVID-19 for individuals aged between 25 and 44 years. Table S13. Risk of developing outcome events among individuals with COVID-19 for individuals aged between 45 and 64 years. Table S14. Risk of developing outcome events among individuals with COVID-19 for individuals aged between 65 years or older.

## Data Availability

Data are not available as the data custodians have not permitted data sharing due to patient confidentiality and privacy concerns.

## Abbreviations

France IQVIA: IQVIA Longitudinal Patient Database France
Germany IQVIA: IQVIA Disease Analyser Germany
Italy IQVIA: Longitudinal Patient Database Italy
UK IMRD: IQVIA Medical Research Data UK
US PharMetrics Plus: IQVIA PharMetrics Plus in the USA
OMOP CDM: Observational Medical Outcomes Partnership Common Data Model
OHDSI: Observational Health Data Sciences and Informatics
HRs: Hazard Ratios
CIs: Confidence Intervals
