# Supplementary material for: Psychiatric and neuropsychiatric sequelae of COVID-19 within 2 years: a multinational cohort study
Source: BMC Med. 2025 Mar 7;23:144. doi: 10.1186/s12916-025-03952-z (PMC11887073; doi:10.1186/s12916-025-03952-z)
Supplement: Supplementary file 1 — Additional file 1: Fig. S1. Study cohort selection procedure, using short-term depression risk in France IQVIA as an example. Table S1. Descriptions of databases. Table S2. Diagnostic codes for COVID-19 identification. Table S3. Diagnostic codes for outcomes identification. Table S4. Sample size, sex and age distribution, follow-up time, the number of psychiatric and neuropsychiatric events, and incidence rate in the medium-term observation period by outcome and database. Table S5. Sample size, sex and age distribution, follow-up time, the number of psychiatric and neuropsychiatric events, and incidence rate in the long-term observation period by outcome and database. Table S6. Selected baseline characteristics for all outcomes in all databases. Table S7. Risk of developing outcome events among individuals with COVID-19 for the whole study sample. Table S8. Risk of developing outcome events among individuals with COVID-19 for males. Table S9. Risk of developing outcome events among individuals with COVID-19 for females. Table S10. Risk of developing outcome events among individuals with COVID-19 for individuals aged below 18 years. Table S11. Risk of developing outcome events among individuals with COVID-19 for individuals aged between 18 and 24 years. Table S12. Risk of developing outcome events among individuals with COVID-19 for individuals aged between 25 and 44 years. Table S13. Risk of developing outcome events among individuals with COVID-19 for individuals aged between 45 and 64 years. Table S14. Risk of developing outcome events among individuals with COVID-19 for individuals aged between 65 years or older. [file 12916_2025_3952_MOESM1_ESM.pdf]

Contents

Fig. S1. Study cohort selection procedure, using short-term depression risk in France IQVIA as an example. 2

Table S1. Descriptions of databases 3

Table S2. Diagnostic codes for COVID-19 identification.......... 5

Table S3. Diagnostic codes for outcomes identification 6

Table S4. Sample size, sex and age distribution, follow-up time, the number of psychiatric and neuropsychiatric events, and incidence rate in the medium-term observation period by outcome and database..........11

Table S5. Sample size, sex and age distribution, follow-up time, the number of psychiatric and neuropsychiatric events, and incidence rate in the long-term observation period by outcome and database 17

Table S6. Selected baseline characteristics for all outcomes in all databases 23

Table S7. Risk of developing outcome events among individuals with COVID-19 for the whole study sample 740

Table S8. Risk of developing outcome events among individuals with COVID-19 for males 742

Table S9. Risk of developing outcome events among individuals with COVID-19 for females 744

Table S10. Risk of developing outcome events among individuals with COVID-19 for individuals aged below 18 years.......... 746

Table S11. Risk of developing outcome events among individuals with COVID-19 for individuals aged between 18 and 24 years 748

Table S12. Risk of developing outcome events among individuals with COVID-19 for individuals aged between 25 and 44 years 750

Table S13. Risk of developing outcome events among individuals with COVID-19 for individuals aged between 45 and 64 years 752

Table S14. Risk of developing outcome events among individuals with COVID-19 for individuals aged between 65 years or older.......... 754

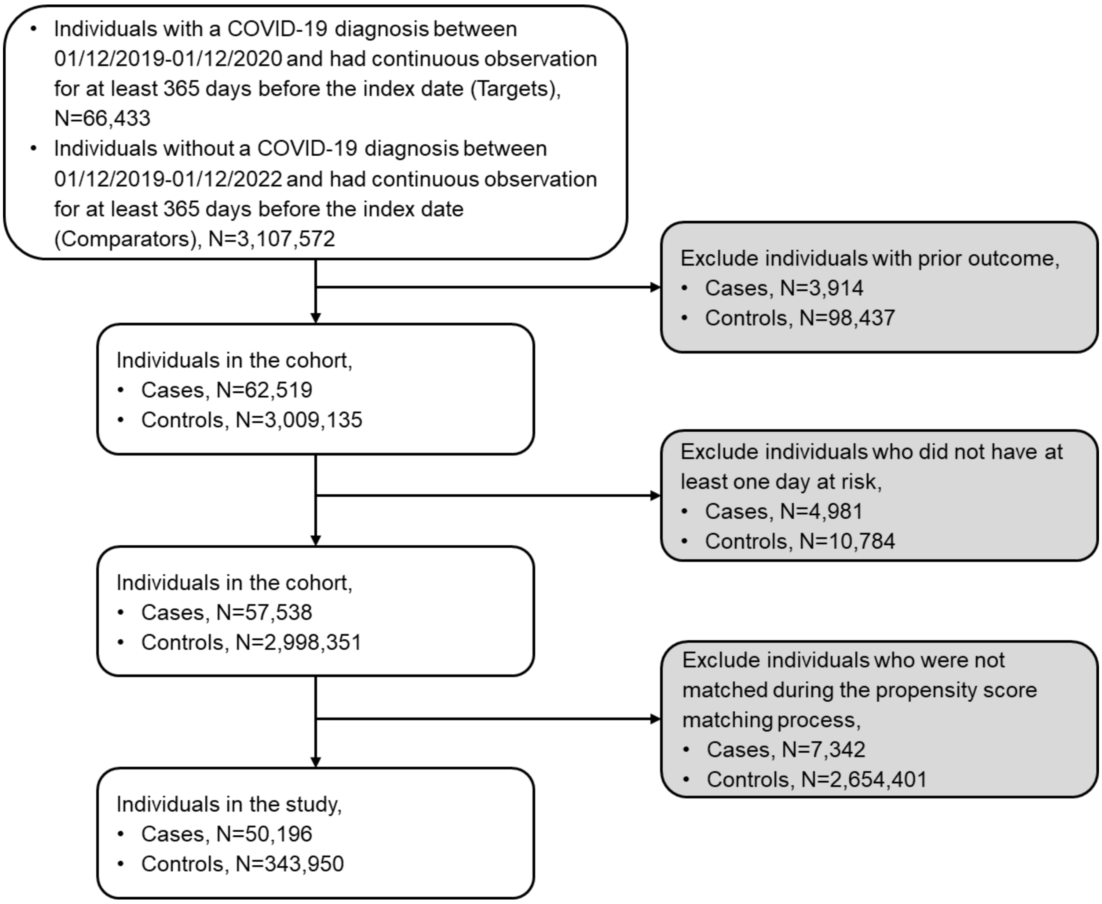

Fig. S1. Study cohort selection procedure, using short-term depression risk in France IQVIA as an example.

Table S1. Descriptions of databases

Database	Short name	Description
IQVIA Longitudinal Patient Database France	France IQVIA	France IQVIA contains anonymized patient records collected from patient management software used by GPs and selected specialists to document patients' clinical records. Currently, 8.7K providers are contributing to the database covering 17.8M cumulative patients in France. Database limited to 10 years history as per CNIL approval. Observation time is defined by the first and last consultation dates.
IQVIA Disease Analyser Germany	Germany IQVIA	Germany IQVIA is comprised of anonymized patient records collected from patient management software used by GPs and selected specialists to document patients' clinical records. Data coverage includes more than 39.8M cumulative patients in the country and collected from 2.8K providers. Patient visiting more than one provider are not cross-identified for data protection reasons and therefore recorded as separate in the system. Dates of service include from 1992 through present. Observation time is defined by the first and last consultation dates.
Longitudinal Patient Database Italy	Italy IQVIA	Italy IQVIA is comprised of anonymized patient records collected from software used by GPs during an office visit to document patients' clinical records. Data coverage includes about 2.3M cumulative patients with at least one visit provided by 1.4K GPs. Dates of service include from 2011 through present. Observation time is defined by the first and last consultation dates.

Table S1. *Continued.* Descriptions of databases

Database	Short name	Description
IQVIA Medical Research Data UK	UK IMRD	UK IMRD is a large database of anonymised electronic medical records collected at Primary Care clinics throughout the UK. Data coverage includes 13.7M cumulative patients from 7.4M providers. Dates of service include from 1994 through present. Quality indicators define the start date for that patient (e.g. each patient's observation period began at the latest of: the patient's registration date, the acceptable mortality recording date of the practice, the Vision date). The end of the observation period is determined by the end date of registration in the database.
IQVIA PharMetrics Plus	US PharMetrics Plus	The IQVIA PharMetrics Plus database is a health plan claims database comprised of fully adjudicated medical and pharmacy claims for more than 210 million unique enrollees since 2006. Data contributors to the database are largely commercial health plans. It is representative of the commercially insured US national population for patients under 65 years of age. It contains a longitudinal view of inpatient and outpatient services, prescription and office/outpatient administered drugs, costs, and detailed enrollment information

Table S2. Diagnostic codes for COVID-19 identification

Phenotype name	Concept ID	Concept name	Link to ATLAS cohort
**COVID-19 diagnosis**	37311059	Exposure to SARS-CoV-2	https://atlas-demo.ohdsi.org/#/cohortdefinition/1779650/conceptsets/
	704994	COVID-19 diagnostic criterion	
	37311061	COVID-19	
**Positive SARS-CoV-2 test results**	37310258	Measurement of severe acute respiratory syndrome coronavirus 2 antibody	
	756055	Measurement of severe acute respiratory syndrome coronavirus 2 (SARS-CoV-2)	

Table S3. Diagnostic codes for outcomes identification

Phenotype name	Concept ID	Concept name	Link to ATLAS cohort
**Depression**	440383	Depressive disorder	https://atlas-demo.ohdsi.org/#/cohortdefinition/1780174
	35625752	Depression care management	
	44788304	Depression-enhanced service completed	
	4114513	Depression-motion	
	4295031	Depression management program	
	44788282	Depression-enhanced services administration	
	2106310	Major depressive disorder, severe without psychotic features (MDD)	
	2106322	Major depressive disorder, severe with psychotic features (MDD)	
	2106305	Major depressive disorder, moderate (MDD)	
	2106304	Major depressive disorder, mild (MDD)	
	40756912	Plan for follow-up care for major depressive disorder, documented (MDD ADOL)	
**Anxiety disorders**	442077	Anxiety disorder	https://atlas-demo.ohdsi.org/#/cohortdefinition/1780177
	4058397	Claustrophobia	
	4322025	Mild anxiety	
	4214746	Severe anxiety	
	440985	Phobia	
	4087190	Performance anxiety	
	4008683	Anxiety neurosis	
	4263429	Moderate anxiety	

Table S3. *Continued.* Diagnostic codes for outcomes identification

Phenotype name	Concept ID	Concept name	Link to ATLAS cohort
**Anxiety disorders**	4338032	Anxiety hysteria	https://atlas-demo.ohdsi.org/#/cohortdefinition/1780177
	4332995	Needle phobia	
	4261239	Anticipatory anxiety	
	4209114	Phonophobia	
	4155074	School phobia	
	4103273	Recurrent anxiety	
	4102977	Disturbance of anxiety and fearfulness in childhood and adolescence	
	4012101	Nosophobia	
**Alcohol misuse or dependence**	433753	Alcohol abuse	https://atlas-demo.ohdsi.org/#/cohortdefinition/1780180
	435243	Alcohol dependence	
	44788279	Alcohol misuse-enhanced services administration	
	44788303	Alcohol misuse-enhanced service completed	
	4218106	Alcoholism	
	439005	Chronic alcoholism in remission	
	436953	Continuous chronic alcoholism	
	4275257	Detoxication psychiatric therapy for alcoholism	
	435532	Episodic chronic alcoholism	
	378726	Dementia associated with alcoholism	
	433735	Acute alcoholic intoxication in alcoholism	
	432609	Acute alcoholic intoxication in remission, in alcoholism	

Table S3. *Continued.* Diagnostic codes for outcomes identification

Phenotype name	Concept ID	Concept name	Link to ATLAS cohort
**Substance misuse or dependence**	4279309	Substance abuse	https://atlas-demo.ohdsi.org/#/cohortdefinition/1780181
	440069	Drug dependence	
	4004672	Psychoactive substance use disorder	
	44786481	Documentation that patient is a current tobacco user	
	4302387	Substance use treatment: drug withdrawal	
	2796056	Substance Abuse Treatment, Pharmacotherapy	
	440787	Drug dependence in mother complicating pregnancy, childbirth AND/OR puerperium	
	434697	Maternal tobacco abuse	
	443274	Psychostimulant dependence	
	4319165	Therapeutic drug dependence	
	37116660	Marijuana user	
	37116661	Cocaine user	
	4269905	Referral to drug abuse counselor	
	4217840	Substance misuse behavior	
	44790195	Delivery of rehabilitation for drug addiction	
	4151569	Drug addiction notification	
	4219382	Persistent substance misuse	
	44787894	Referral to community drug dependency team	
	4149607	Drug addiction therapy-methadone	

Table S3. *Continued.* Diagnostic codes for outcomes identification

Phenotype name	Concept ID	Concept name	Link to ATLAS cohort
**Bipolar disorders**	436665	Bipolar disorder	https://atlas-demo.ohdsi.org/#/cohortdefinition/1780178
	4128935	Bipolar	
**Psychoses**	436073	Psychotic disorder	https://atlas-demo.ohdsi.org/#/cohortdefinition/1780172
	4168389	Borderline schizophrenia	
	432590	Delusional disorder	
	439706	Psychosis with origin in childhood	
**Personality disorders**	441838	Personality disorder	https://atlas-demo.ohdsi.org/#/cohortdefinition/1780179
**Self-harm and suicide**	4219484	Suicide attempt	https://atlas-demo.ohdsi.org/#/cohortdefinition/1780175
	440925	Suicide	
	444362	Suicidal deliberate poisoning	
	4092411	Self-injurious behavior	
	4181216	Self-administered poisoning	
	439235	Self inflicted injury	
	435446	Late effect of self inflicted injury	
	4303690	Intentionally harming self	
**Sleep disorders**	435524	Sleep disorder	https://atlas-demo.ohdsi.org/#/cohortdefinition/1780372

Table S3. *Continued.* Diagnostic codes for outcomes identification

Phenotype name	Concept ID	Concept name	Link to ATLAS cohort
**Dementia**	4182210	Dementia	https://atlas-demo.ohdsi.org/#/cohortdefinition/1780173
	35610623	Dementia advance care plan agreed	
	46284876	Dementia care plan agreed	
	46284877	Dementia care plan reviewed	
	44790944	Dementia monitoring	
	44803643	Dementia monitoring first letter	
	44803915	Dementia monitoring second letter	
	44803843	Dementia monitoring telephone invitation	
	44803781	Dementia monitoring third letter	
	44803706	Dementia monitoring verbal invitation	
	4043378	Frontotemporal dementia	
	42742407	Functional status for dementia assessed and results reviewed (DEM)	
	4059191	H/O: dementia	
	44782763	Lewy body dementia with behavioral disturbance	
**Neurodevelopmental disorders**	45771096	Neurodevelopmental disorder	https://atlas-demo.ohdsi.org/#/cohortdefinition/1780171
	40480225	Adult attention deficit hyperactivity disorder	
	4165912	On learning disability register	
	442335	Dyslexia	
	40483181	History of neurodevelopmental disorder	
	4136053	Learning disabilities health action plan reviewed	
	45765570	Drug therapy for attention deficit hyperactivity disorder	
	4024717	Dyslexia AND/OR speech dysfunction	

Table S4. Sample size, sex and age distribution, follow-up time, the number of psychiatric and neuropsychiatric events, and incidence rate in the medium-term observation period by outcome and database

Outcome	Database	Targets/ Comparators	No. of individuals after propensity score matching	Sex, N (%)		Age, N (%)					No. of follow-up years	No. of events	Incidence rate per 1000 person years
				Males	Females	<18 years	18-24 years	25-44 years	45-64 years	65+ years			
Depression	France IQVIA	Comparators	291192	128093 (43.99)	159231 (54.68)	29110 (10)	25250 (8.67)	82780 (28.43)	98103 (33.69)	47331 (16.25)	136863	3282	23.98
		Targets	42107	18068 (42.91)	23293 (55.32)	3810 (9.05)	3314 (7.87)	12068 (28.66)	14350 (34.08)	5990 (14.23)	20010	684	34.18
	Germany IQVIA	Comparators	105639	48838 (46.23)	55534 (52.57)	12395 (11.73)	9860 (9.33)	29025 (27.48)	33913 (32.1)	16293 (15.42)	50451	2242	44.44
		Targets	15884	7478 (47.08)	8329 (52.44)	1540 (9.7)	1509 (9.5)	4764 (29.99)	5620 (35.38)	2170 (13.66)	4665	389	50.75
	Italy IQVIA	Comparators	38258	14835 (38.78)	19947 (52.14)	927 (2.42)	2433 (6.36)	9457 (24.72)	16002 (41.83)	9456 (24.72)	18004	385	21.38
		Targets	6504	2508 (38.56)	3284 (50.49)	157 (2.41)	398 (6.12)	1578 (24.26)	2733 (42.02)	1407 (21.63)	3111	81	26.03
	UK IMRD	Comparators	259390	111353 (42.93)	146817 (56.6)	31309 (12.07)	26423 (10.19)	82048 (31.63)	83888 (32.34)	30312 (11.69)	122837	1307	10.64
		Targets	30142	12993 (43.11)	17126 (56.82)	3435 (11.4)	3174 (10.53)	9592 (31.82)	9931 (32.95)	3876 (12.86)	14514	149	10.27
	US PharMetrics Plus	Comparators	469098	228731 (48.76)	236723 (50.46)	55799 (11.89)	59668 (12.72)	144832 (30.87)	167585 (35.72)	28212 (6.01)	221609	11123	50.19
		Targets	102617	50244 (48.96)	51285 (49.98)	9274 (9.04)	1370 (1.34)	32268 (31.45)	37385 (36.43)	5292 (5.16)	48893	2295	46.94
Anxiety disorders	France IQVIA	Comparators	281116	124423 (44.26)	153019 (54.43)	28451 (10.12)	24093 (8.57)	78420 (27.9)	95354 (33.92)	46524 (16.55)	131527	5389	40.97
		Targets	40323	17448 (43.27)	22179 (55)	3708 (9.2)	3154 (7.82)	11328 (28.09)	13874 (34.41)	5844 (14.49)	19045	1109	58.23
	Germany IQVIA	Comparators	107474	49433 (46)	56691 (52.75)	12166 (11.32)	9727 (9.05)	28865 (26.86)	34707 (32.29)	17762 (16.53)	51396	2127	41.38
		Targets	16410	7651 (46.62)	8672 (52.85)	1516 (9.24)	1527 (9.31)	4815 (29.34)	5799 (35.34)	2369 (14.44)	7951	324	40.75
	Italy IQVIA	Comparators	41039	15317 (37.32)	22107 (53.87)	925 (2.25)	2450 (5.97)	9679 (23.58)	17144 (41.77)	10764 (26.23)	19399	95	4.9
		Targets	7034	2616 (37.19)	3663 (52.08)	156 (2.22)	403 (5.73)	1631 (23.19)	2958 (42.05)	1641 (23.33)	3383	21	6.21
	UK IMRD	Comparators	258435	111329 (43.08)	145908 (56.46)	31129 (12.05)	26317 (10.18)	81474 (31.53)	83799 (32.43)	30347 (11.74)	122302	1489	12.17
		Targets	30009	12977 (43.24)	17010 (56.68)	3416 (11.38)	3159 (10.53)	9516 (31.71)	9911 (33.03)	3874 (12.91)	14446	169	11.7
	US PharMetrics Plus	Comparators	428761	214854 (50.11)	210979 (49.21)	52588 (12.27)	53649 (12.51)	128563 (29.98)	154676 (36.08)	27262 (6.36)	200993	16839	83.78
		Targets	93934	47269 (50.32)	45620 (48.57)	8767 (9.33)	12416 (13.22)	28672 (30.52)	34576 (36.81)	5166 (5.5)	44392	3493	78.68

Table S4. *Continued.* Sample size, sex and age distribution, follow-up time, the number of psychiatric and neuropsychiatric events, and incidence rate in the medium-term observation period by outcome and database

**Outcome**	**Database**	**Targets/ Comparators**	**No. of individuals after propensity score matching**	**Sex, N (%)**		**Age, N (%)**					**No. of follow-up years**	**No. of events**	**Incidence rate per 1000 person years**
				**Males**	**Females**	**<18 years**	**18-24 years**	**25-44 years**	**45-64 years**	**65+ years**			
**Alcohol misuse or dependence**	France IQVIA	Comparators	317071	135016 (42.58)	177615 (56.02)	29284 (9.24)	26025 (8.21)	89349 (28.18)	109912 (34.66)	52833 (16.66)	149843	198	1.32
		Targets	46047	19121 (41.52)	26139 (56.77)	3833 (8.32)	3428 (7.44)	13106 (28.46)	16234 (35.26)	6699 (14.55)	22069	27	1.22
	Germany IQVIA	Comparators	115714	52185 (45.1)	62213 (53.76)	12594 (10.88)	10426 (9.01)	31549 (27.26)	37898 (32.75)	18731 (16.19)	55845	155	2.78
		Targets	17640	8066 (45.73)	9482 (53.75)	1572 (8.91)	1626 (9.22)	5257 (29.8)	6300 (35.71)	2491 (14.12)	8632	18	2.09
	Italy IQVIA	Comparators	41764	15578 (37.3)	22497 (53.87)	928 (2.22)	2471 (5.92)	9908 (23.72)	17503 (41.91)	10836 (25.95)	19759	23	1.16
		Targets	7147	2647 (37.04)	3733 (52.23)	157 (2.2)	405 (5.67)	1668 (23.34)	3013 (42.16)	1650 (23.09)	3442	<5	<1.45
	UK IMRD	Comparators	264306	112810 (42.68)	150224 (56.84)	31426 (11.89)	27295 (10.33)	84223 (31.87)	85105 (32.2)	30712 (11.62)	125408	141	1.12
		Targets	30710	13153 (42.83)	17530 (57.08)	3450 (11.23)	3280 (10.68)	9840 (32.04)	10070 (32.79)	3929 (12.79)	14830	6	0.4
	US PharMetrics Plus	Comparators	532936	244548 (45.89)	284047 (53.3)	59470 (11.16)	68690 (12.89)	165492 (31.05)	189470 (35.55)	34459 (6.47)	254166	1675	6.59
		Targets	116094	53450 (46.04)	61397 (52.89)	9913 (8.54)	15594 (13.43)	36610 (31.53)	42194 (36.34)	6594 (5.68)	55827	327	5.86
**Substance misuse or dependence**	France IQVIA	Comparators	314853	133784 (42.49)	176672 (56.11)	29270 (9.3)	25910 (8.23)	88591 (28.14)	108910 (34.59)	52595 (16.7)	148712	549	3.69
		Targets	45712	18938 (41.43)	25981 (56.84)	3831 (8.38)	3413 (7.47)	12983 (28.4)	16071 (35.16)	6668 (14.59)	21892	90	4.11
	Germany IQVIA	Comparators	113746	51090 (44.92)	61380 (53.96)	12560 (11.04)	10304 (9.06)	31006 (27.26)	37013 (32.54)	18443 (16.21)	54778	623	11.37
		Targets	17406	7925 (45.53)	9386 (53.92)	1750 (10.05)	1612 (9.26)	5184 (29.78)	6194 (35.59)	2457 (14.12)	8508	57	6.7
	Italy IQVIA	Comparators	41640	15483 (37.18)	22469 (53.96)	928 (2.23)	2463 (5.91)	9868 (23.7)	17475 (41.97)	10789 (25.91)	19694	52	2.64
		Targets	7123	2635 (36.99)	3725 (52.3)	157 (2.2)	403 (5.66)	1660 (23.3)	3006 (42.2)	1641 (23.04)	3431	6	1.75
	UK IMRD	Comparators	246052	112607 (45.77)	150173 (61.03)	31425 (12.77)	27247 (11.07)	84172 (34.21)	85043 (34.56)	30665 (12.46)	125267	234	1.87
		Targets	30683	13131 (42.8)	17526 (57.12)	3449 (11.24)	3275 (10.67)	9832 (32.04)	10064 (32.8)	3922 (12.78)	14818	11	0.74
	US PharMetrics Plus	Comparators	502527	229234 (45.62)	269185 (53.57)	58926 (11.73)	65191 (12.97)	155338 (30.91)	176310 (35.08)	32452 (6.46)	238547	7025	29.45
		Targets	109701	50153 (45.72)	58365 (53.2)	9819 (8.95)	14823 (13.51)	34428 (31.38)	39368 (35.89)	6185 (5.64)	52562	1260	23.97

Table S4. *Continued.* Sample size, sex and age distribution, follow-up time, the number of psychiatric and neuropsychiatric events, and incidence rate in the medium-term observation period by outcome and database

Outcome	Database	Targets/ Comparators	No. of individuals after propensity score matching	Sex, N (%)		Age, N (%)					No. of follow-up years	No. of events	Incidence rate per 1000 person years
				Males	Females	<18 years	18-24 years	25-44 years	45-64 years	65+ years			
**Bipolar disorders**	France IQVIA	Comparators	317512	135541 (42.69)	177535 (55.91)	29275 (9.22)	26017 (8.19)	89401 (28.16)	110254 (34.72)	52842 (16.64)	150079	78	0.52
		Targets	46098	19192 (41.63)	26124 (56.67)	3832 (8.31)	3429 (7.44)	13118 (28.46)	16276 (35.31)	6698 (14.53)	22095	18	0.81
	Germany IQVIA	Comparators	116110	52465 (45.19)	62325 (53.68)	12598 (10.85)	10421 (8.98)	31636 (27.25)	38089 (32.8)	18810 (16.2)	56066	21	0.37
		Targets	17686	8102 (45.81)	9490 (53.66)	1571 (8.88)	1627 (9.2)	5269 (29.79)	6324 (35.76)	2502 (14.15)	8658	6	0.69
	Italy IQVIA	Comparators	41719	15559 (37.29)	22450 (53.81)	928 (2.22)	2470 (5.92)	9865 (23.65)	17464 (41.86)	10881 (26.08)	19737	11	0.56
		Targets	7137	2650 (37.13)	3720 (52.12)	157 (2.2)	404 (5.66)	1664 (23.32)	3008 (42.15)	1654 (23.18)	3437	<5	<1.45
	UK IMRD	Comparators	264769	113101 (42.72)	150408 (56.81)	31426 (11.87)	27306 (10.31)	84403 (31.88)	85351 (32.24)	30754 (11.62)	125671	25	0.2
		Targets	30757	13182 (42.86)	17547 (57.05)	3449 (11.21)	3281 (10.67)	9859 (32.05)	10094 (32.82)	3932 (12.78)	14854	<5	<0.34
	US PharMetrics Plus	Comparators	534188	247169 (46.27)	282589 (52.9)	59351 (11.11)	68638 (12.85)	165567 (30.99)	190608 (35.68)	34642 (6.48)	254914	989	3.88
		Targets	116221	53976 (46.44)	61002 (52.49)	9893 (8.51)	15574 (13.4)	36575 (31.47)	42374 (36.46)	6603 (5.68)	55926	184	3.29
**Psychoses**	France IQVIA	Comparators	317404	135349 (42.64)	177635 (55.96)	29271 (9.22)	26033 (8.2)	89386 (28.16)	110242 (34.73)	52783 (16.63)	150013	122	0.81
		Targets	46090	19175 (41.6)	26131 (56.7)	3832 (8.31)	3430 (7.44)	13115 (28.46)	16274 (35.31)	6694 (14.52)	22091	21	0.95
	Germany IQVIA	Comparators	115606	52278 (45.22)	62047 (53.67)	12578 (10.88)	10412 (9.01)	31551 (27.29)	37936 (32.81)	18581 (16.07)	55807	110	1.97
		Targets	17631	8082 (45.84)	9457 (53.64)	1570 (8.9)	1625 (9.22)	5256 (29.81)	6306 (35.77)	2485 (14.09)	8627	17	1.97
	Italy IQVIA	Comparators	41633	15529 (37.3)	22424 (53.86)	928 (2.23)	2470 (5.93)	9907 (23.8)	17443 (41.9)	10779 (25.89)	19698	21	1.07
		Targets	7125	2640 (37.05)	3719 (52.2)	157 (2.2)	404 (5.67)	1663 (23.34)	3005 (42.18)	1641 (23.03)	3431	<5	<1.46
	UK IMRD	Comparators	264638	113021 (42.71)	150334 (56.81)	31416 (11.87)	27294 (10.31)	84375 (31.88)	85305 (32.23)	30723 (11.61)	125588	74	0.59
		Targets	30745	13174 (42.85)	17543 (57.06)	3448 (11.21)	3279 (10.67)	9860 (32.07)	10093 (32.83)	3923 (12.76)	14847	8	0.54
	US PharMetrics Plus	Comparators	538242	248263 (46.12)	285502 (53.04)	59522 (11.06)	69350 (12.88)	167494 (31.12)	191892 (35.65)	34471 (6.4)	256963	377	1.47
		Targets	117137	54236 (46.3)	61644 (52.63)	9922 (8.47)	15742 (13.44)	36985 (31.57)	42693 (36.45)	6569 (5.61)	56391	75	1.33

Table S4. *Continued.* Sample size, sex and age distribution, follow-up time, the number of psychiatric and neuropsychiatric events, and incidence rate in the medium-term observation period by outcome and database

**Outcome**	**Database**	**Targets/ Comparators**	**No. of individuals after propensity score matching**	**Sex, N (%)**		**Age, N (%)**					**No. of follow-up years**	**No. of events**	**Incidence rate per 1000 person years**
				**Males**	**Females**	**<18 years**	**18-24 years**	**25-44 years**	**45-64 years**	**65+ years**			
**Personality disorders**	France IQVIA	Comparators	318071	135665 (42.65)	177928 (55.94)	29275 (9.2)	26029 (8.18)	89535 (28.15)	110565 (34.76)	52972 (16.65)	150353	46	0.31
		Targets	46178	19215 (41.61)	26183 (56.7)	3832 (8.3)	3429 (7.43)	13134 (28.44)	16322 (35.35)	6713 (14.54)	22136	<10	<0.45
	Germany IQVIA	Comparators	115596	52215 (45.17)	62078 (53.7)	12569 (10.87)	10403 (9)	31506 (27.26)	37869 (32.76)	18708 (16.18)	55803	149	2.67
		Targets	17618	8069 (45.8)	9452 (53.65)	1569 (8.91)	1620 (9.2)	5254 (29.82)	6293 (35.72)	2491 (14.14)	8619	24	2.78
	Italy IQVIA	Comparators	41203	15426 (37.44)	22124 (53.7)	928 (2.25)	2468 (5.99)	9905 (24.04)	17356 (42.12)	10431 (25.32)	19473	151	7.75
		Targets	7049	2626 (37.25)	3671 (52.08)	157 (2.23)	405 (5.75)	1666 (23.63)	2991 (42.43)	1579 (22.4)	3391	24	7.07
	UK IMRD	Comparators	264578	113054 (42.73)	150226 (56.78)	31426 (11.88)	27224 (10.29)	84349 (31.88)	85267 (32.23)	30765 (11.63)	125568	83	0.66
		Targets	30741	13180 (42.87)	17533 (57.03)	3450 (11.22)	3271 (10.64)	9856 (32.06)	10089 (32.82)	3933 (12.79)	14846	<5	<0.34
	US PharMetrics Plus	Comparators	538846	248736 (46.16)	285626 (53.01)	59400 (11.02)	69223 (12.85)	167446 (31.07)	192252 (35.68)	35001 (6.5)	257183	465	1.81
		Targets	117254	54315 (46.32)	61678 (52.6)	9904 (8.45)	15716 (13.4)	36965 (31.53)	42744 (36.45)	6693 (5.71)	56437	77	1.36
**Self-harm and suicide**	France IQVIA	Comparators	318156	135740 (42.66)	177951 (55.93)	29254 (9.19)	26034 (8.18)	89595 (28.16)	110580 (34.76)	53007 (16.66)	150396	34	0.23
		Targets	46191	19227 (41.62)	26187 (56.69)	3828 (8.29)	3428 (7.42)	13144 (28.46)	16326 (35.34)	6716 (14.54)	22142	10	0.45
	Germany IQVIA	Comparators	116237	52523 (45.19)	62402 (53.69)	12589 (10.83)	10435 (8.98)	31659 (27.24)	38136 (32.81)	18863 (16.23)	56132	<5	<0.09
		Targets	17702	8108 (45.8)	9500 (53.67)	1571 (8.87)	1628 (9.2)	5272 (29.78)	6329 (35.75)	2507 (14.16)	8667	<5	<0.58
	Italy IQVIA	Comparators	41864	15616 (37.3)	22539 (53.84)	928 (2.22)	2471 (5.9)	9941 (23.75)	17520 (41.85)	10896 (26.03)	19811	0	0
		Targets	7165	2656 (37.07)	3738 (52.17)	157 (2.19)	405 (5.65)	1672 (23.34)	3020 (42.15)	1656 (23.11)	3450	0	0
	UK IMRD	Comparators	264490	113024 (42.73)	150186 (56.78)	31355 (11.85)	27229 (10.29)	84348 (31.89)	85288 (32.25)	30713 (11.61)	125509	145	1.16
		Targets	30722	13174 (42.88)	17520 (57.03)	3441 (11.2)	3271 (10.65)	9851 (32.06)	10089 (32.84)	3928 (12.79)	14833	18	1.21
	US PharMetrics Plus	Comparators	539914	249092 (46.14)	286295 (53.03)	59439 (11.01)	69508 (12.87)	167905 (31.1)	192468 (35.65)	35046 (6.49)	257773	181	0.7
		Targets	117456	54375 (46.29)	61813 (52.63)	9910 (8.44)	15748 (13.41)	37055 (31.55)	42793 (36.43)	6705 (5.71)	56542	37	0.65

Table S4. *Continued.* Sample size, sex and age distribution, follow-up time, the number of psychiatric and neuropsychiatric events, and incidence rate in the medium-term observation period by outcome and database

Outcome	Database	Targets/ Comparators	No. of individuals after propensity score matching	Sex, N (%)		Age, N (%)					No. of follow-up years	No. of events	Incidence rate per 1000 person years
				Males	Females	<18 years	18-24 years	25-44 years	45-64 years	65+ years			
**Sleep disorders**	France IQVIA	Comparators	296361	127563 (43.04)	165038 (55.69)	28781 (9.71)	25267 (8.53)	85266 (28.77)	101777 (34.34)	46758 (15.78)	139228	3378	24.26
		Targets	42826	17958 (41.93)	24106 (56.29)	3767 (8.8)	3315 (7.74)	12411 (28.98)	14908 (34.81)	5854 (13.67)	20363	639	31.38
	Germany IQVIA	Comparators	109492	49406 (45.12)	58786 (53.69)	12386 (11.31)	10179 (9.3)	30250 (27.63)	35353 (32.29)	16999 (15.53)	52503	1600	30.47
		Targets	16732	7673 (45.86)	8968 (53.6)	1545 (9.23)	1596 (9.54)	5056 (30.22)	5898 (35.25)	2261 (13.51)	8127	268	32.97
	Italy IQVIA	Comparators	39476	14872 (37.67)	2111 (5.35)	926 (2.35)	2445 (6.19)	9664 (24.48)	16575 (41.99)	9781 (24.78)	18593	350	262091
		Targets	6743	2529 (37.51)	3494 (51.82)	156 (2.31)	400 (5.93)	1626 (24.11)	2831 (41.98)	1485 (22.02)	3227	71	30449
	UK IMRD	Comparators	262091	112047 (42.75)	148801 (56.77)	31332 (11.95)	27136 (10.35)	83568 (31.89)	84366 (32.19)	30208 (11.53)	124243	671	5.4
		Targets	30449	13062 (42.9)	17363 (57.02)	3442 (11.3)	3259 (10.7)	9765 (32.07)	9976 (32.76)	3863 (12.69)	14690	71	4.83
	US PharMetrics Plus	Comparators	470654	214967 (45.67)	250952 (53.32)	57233 (12.16)	66324 (14.09)	151008 (32.08)	156350 (33.22)	26598 (5.65)	221882	11848	53.4
		Targets	102612	47321 (46.12)	54210 (52.83)	9573 (9.33)	15091 (14.71)	33413 (32.56)	34831 (33.94)	5072 (4.94)	48761	2689	55.15
**Dementia**	France IQVIA	Comparators	317561	135590 (42.7)	177571 (55.92)	29285 (9.22)	26039 (8.2)	89586 (28.21)	110558 (34.81)	52442 (16.51)	150116	85	0.57
		Targets	46123	19208 (41.65)	26136 (56.67)	3833 (8.31)	3431 (7.44)	13145 (28.5)	16320 (35.38)	6647 (14.41)	22113	10	0.45
	Germany IQVIA	Comparators	114581	52012 (45.39)	61286 (53.49)	12599 (11)	10434 (9.11)	31641 (27.61)	38041 (33.2)	17339 (15.13)	55352	199	3.6
		Targets	17510	8049 (45.97)	9363 (53.47)	1570 (8.97)	1628 (9.3)	5269 (30.09)	6316 (36.07)	2333 (13.32)	8567	32	3.73
	Italy IQVIA	Comparators	41588	15561 (37.42)	22348 (53.74)	928 (2.23)	2471 (5.94)	9941 (23.9)	17508 (42.1)	10647 (25.6)	19680	35	1.78
		Targets	7124	2649 (37.18)	3710 (52.08)	157 (2.2)	405 (5.69)	1672 (23.47)	3019 (42.38)	1617 (22.7)	3432	11	3.21
	UK IMRD	Comparators	262296	112294 (42.81)	148796 (56.73)	31416 (11.98)	27314 (10.41)	84438 (32.19)	85251 (32.5)	28675 (10.93)	124491	387	3.11
		Targets	30429	13068 (42.95)	17328 (56.95)	3447 (11.33)	3282 (10.79)	9865 (32.42)	10084 (33.14)	3620 (11.9)	14689	82	5.58
	US PharMetrics Plus	Comparators	534322	247274 (46.28)	282747 (52.92)	59444 (11.13)	69495 (13.01)	167508 (31.35)	191848 (35.9)	31598 (5.91)	255089	738	2.89
		Targets	116343	54008 (46.42)	61104 (52.52)	9912 (8.52)	15745 (13.53)	36999 (31.8)	42661 (36.67)	5889 (5.06)	56008	136	2.43

Table S4. *Continued.* Sample size, sex and age distribution, follow-up time, the number of psychiatric and neuropsychiatric events, and incidence rate in the medium-term observation period by outcome and database

**Outcome**	**Database**	**Targets/ Comparators**	**No. of individuals after propensity score matching**	**Sex, N (%)**		**Age, N (%)**					**No. of follow-up years**	**No. of events**	**Incidence rate per 1000 person years**
				**Males**	**Females**	**<18 years**	**18-24 years**	**25-44 years**	**45-64 years**	**65+ years**			
**Neurodevelopmental disorders**	France IQVIA	Comparators	317156	135108 (42.6)	177649 (56.01)	28680 (9.04)	25893 (8.16)	89485 (28.21)	110481 (34.83)	52917 (16.68)	149899	164	1.09
		Targets	46044	19134 (41.56)	26128 (56.75)	3763 (8.17)	3410 (7.41)	13125 (28.51)	16303 (35.41)	6705 (14.56)	22066	38	1.72
	Germany IQVIA	Comparators	114148	51427 (45.05)	61544 (53.92)	11415 (10)	10354 (9.07)	31519 (27.61)	38005 (33.29)	18636 (16.33)	55015	465	8.45
		Targets	17466	7973 (45.65)	9398 (53.81)	1425 (8.16)	1612 (9.23)	5255 (30.09)	6301 (36.08)	2479 (14.19)	8536	54	6.33
	Italy IQVIA	Comparators	41797	15593 (37.31)	22506 (53.85)	925 (2.21)	2457 (5.88)	9927 (23.75)	17497 (41.86)	10883 (26.04)	19778	18	0.91
		Targets	7144	2644 (37.01)	3731 (52.23)	155 (2.17)	398 (5.57)	1666 (23.32)	3016 (42.22)	1654 (23.15)	3441	<5	<1.45
	UK IMRD	Comparators	264018	112528 (42.62)	150216 (56.9)	31095 (11.78)	27210 (10.31)	84276 (31.92)	85227 (32.28)	30695 (11.63)	125239	361	2.88
		Targets	30679	13121 (42.77)	17528 (57.13)	3416 (11.13)	3272 (10.67)	9842 (32.08)	10084 (32.87)	3924 (12.79)	14812	31	2.09
	US PharMetrics Plus	Comparators	513064	235744 (45.95)	273064 (53.22)	53071 (10.34)	63423 (12.36)	159183 (31.03)	187871 (36.62)	34311 (6.69)	244076	3700	15.16
		Targets	111835	51784 (46.3)	58841 (52.61)	8899 (7.96)	14445 (12.92)	35192 (31.47)	41838 (37.41)	6544 (5.85)	53637	793	14.78
**Overall**	France IQVIA	Comparators	246658	111579 (45.24)	132227 (53.61)	27292 (11.06)	22832 (9.26)	71025 (28.79)	80836 (32.77)	37651 (15.26)	114507	8322	72.68
		Targets	35228	15567 (44.19)	19006 (53.95)	3566 (10.12)	2976 (8.45)	10187 (28.92)	11632 (33.02)	4705 (13.36)	16470	1667	101.21
	Germany IQVIA	Comparators	91217	42348 (46.43)	47725 (52.32)	10822 (11.86)	8971 (9.83)	25552 (28.01)	29186 (32)	13220 (14.49)	42950	4638	107.98
		Targets	13952	6607 (47.36)	7270 (52.11)	1340 (9.6)	1392 (9.98)	4233 (30.34)	4926 (35.31)	1789 (12.82)	6631	724	109.17
	Italy IQVIA	Comparators	34635	13493 (38.96)	18079 (52.2)	919 (2.65)	2373 (6.85)	8838 (25.52)	14669 (42.35)	7862 (22.7)	16187	819	50.59
		Targets	5891	2295 (38.96)	2972 (50.45)	153 (2.6)	386 (6.55)	1478 (25.09)	2494 (42.34)	1177 (19.98)	2796	166	59.36
	UK IMRD	Comparators	248752	107495 (43.21)	140158 (56.34)	30640 (12.32)	25558 (10.27)	79053 (31.78)	81360 (32.71)	27375 (11)	117328	3474	29.61
		Targets	28869	12532 (43.41)	16315 (56.51)	3363 (11.65)	3069 (10.63)	9234 (31.99)	9639 (33.39)	3444 (11.93)	13854	398	28.73
	US PharMetrics Plus	Comparators	339340	167609 (49.39)	168952 (49.79)	46166 (13.6)	45459 (13.4)	105614 (31.12)	115589 (34.06)	17453 (5.14)	156669	23594	150.6
		Targets	74913	37522 (50.09)	36621 (48.88)	7747 (10.34)	10709 (14.3)	23754 (31.71)	25984 (34.69)	3261 (4.35)	34875	5026	144.11

Table S5. Sample size, sex and age distribution, follow-up time, the number of psychiatric and neuropsychiatric events, and incidence rate in the long-term observation period by outcome and database

Outcome	Database	Targets/ Comparators	No. of individuals after propensity score matching	Sex, N (%)		Age, N (%)					No. of follow-up years	No. of events	Incidence rate per 1000 person years
				Males	Females	<18 years	18-24 years	25-44 years	45-64 years	65+ years			
Depression	France IQVIA	Comparators	252390	112109 (44.42)	139572 (55.3)	25457 (10.09)	21671 (8.59)	70972 (28.12)	86073 (34.1)	41968 (16.63)	174567	4175	23.92
		Targets	36624	15765 (43.05)	20200 (55.16)	3316 (9.05)	2839 (7.75)	10333 (28.21)	12639 (34.51)	5322 (14.53)	25784	890	34.52
	Germany IQVIA	Comparators	93015	43375 (46.63)	49214 (52.91)	11289 (12.14)	8656 (9.31)	25643 (27.57)	30663 (32.97)	14251 (15.32)	62130	2643	42.54
		Targets	14327	6749 (47.11)	7497 (52.33)	1418 (9.9)	1344 (9.38)	4247 (29.64)	5077 (35.44)	1929 (13.46)	9530	406	42.6
	Italy IQVIA	Comparators	33752	12983 (38.47)	17754 (52.6)	861 (2.55)	2026 (6)	8336 (24.7)	14392 (42.64)	8435 (24.99)	22422	448	19.98
		Targets	5689	2183 (38.37)	2906 (51.08)	143 (2.51)	334 (5.87)	1383 (24.31)	2417 (42.49)	1254 (22.04)	3786	74	19.54
	UK IMRD	Comparators	226674	97129 (42.85)	127774 (56.37)	27091 (11.95)	22529 (9.94)	70741 (31.21)	74847 (33.02)	26554 (11.71)	143452	1558	10.86
		Targets	26670	11506 (43.14)	15150 (56.81)	3016 (11.31)	2751 (10.31)	8376 (31.41)	9001 (33.75)	3388 (12.7)	16487	188	11.4
	US PharMetrics Plus	Comparators	411989	203345 (49.36)	205987 (50)	50869 (12.35)	52694 (12.79)	122980 (29.85)	149766 (36.35)	24354 (5.91)	280590	11803	42.06
		Targets	90758	44826 (49.39)	44555 (49.09)	8532 (9.4)	12214 (13.46)	27445 (30.24)	33471 (36.88)	4554 (5.02)	62879	2424	38.55
Anxiety disorders	France IQVIA	Comparators	241152	107933 (44.76)	132711 (55.03)	24690 (10.24)	20356 (8.44)	66472 (27.56)	83033 (34.43)	40984 (17)	165722	6536	39.44
		Targets	34688	15073 (43.45)	19028 (54.85)	3191 (9.2)	2661 (7.67)	9580 (27.62)	12082 (34.83)	5173 (14.91)	24160	1431	59.23
	Germany IQVIA	Comparators	95027	44120 (46.43)	50394 (53.03)	11024 (11.6)	8563 (9.01)	25533 (26.87)	31720 (33.38)	15679 (16.5)	63564	2513	39.53
		Targets	14919	6958 (46.64)	7854 (52.64)	1389 (9.31)	1362 (9.13)	4301 (28.83)	5305 (35.56)	2132 (14.29)	9956	444	44.59
	Italy IQVIA	Comparators	36529	13507 (36.98)	19871 (54.4)	860 (2.35)	2030 (5.56)	8554 (23.42)	15603 (42.71)	9783 (26.78)	24462	112	4.58
		Targets	6221	2289 (36.79)	3287 (52.84)	143 (2.3)	338 (5.43)	1440 (23.15)	2650 (42.6)	1471 (23.65)	4172	21	5.03
	UK IMRD	Comparators	225528	97117 (43.06)	126691 (56.18)	26869 (11.91)	22355 (9.91)	70110 (31.09)	74692 (33.12)	26569 (11.78)	142689	1714	12.01
		Targets	26518	11497 (43.36)	15007 (56.59)	2993 (11.29)	2729 (10.29)	8305 (31.32)	8971 (33.83)	3384 (12.76)	16397	187	11.4
	US PharMetrics Plus	Comparators	370473	188870 (50.98)	179415 (48.43)	47230 (12.75)	46271 (12.49)	106907 (28.86)	136576 (36.87)	23501 (6.34)	249915	17388	69.58
		Targets	81624	41657 (51.04)	38735 (47.46)	7936 (9.72)	10734 (13.15)	23908 (29.29)	30491 (37.36)	4406 (5.4)	55916	3658	65.42

Table 5. *Continued.* Sample size, sex and age distribution, follow-up time, the number of psychiatric and neuropsychiatric events, and incidence rate in the long-term observation period by outcome and database

Outcome	Database	Targets/ Comparators	No. of individuals after propensity score matching	Sex, N (%)		Age, N (%)					No. of follow-up years	No. of events	Incidence rate per 1000 person years
				Males	Females	<18 years	18-24 years	25-44 years	45-64 years	65+ years			
Alcohol misuse or dependence	France IQVIA	Comparators	279233	119332 (42.74)	159015 (56.95)	25663 (9.19)	22589 (8.09)	78218 (28.01)	98304 (35.21)	47404 (16.98)	195313	270	1.61
		Targets	40770	16853 (41.34)	23191 (56.88)	3342 (8.2)	2975 (7.3)	11449 (28.08)	14582 (35.77)	6055 (14.85)	29193	47	1.38
	Germany IQVIA	Comparators	104269	47145 (45.21)	56679 (54.36)	11543 (11.07)	9320 (8.94)	28608 (27.44)	35329 (33.88)	16661 (15.98)	70825	195	2.75
		Targets	16366	7437 (45.44)	8809 (53.83)	1461 (8.93)	1478 (9.03)	4806 (29.37)	5903 (36.07)	2264 (13.83)	11117	25	2.25
	Italy IQVIA	Comparators	37323	13793 (36.96)	20310 (54.42)	862 (2.31)	2067 (5.54)	8822 (23.64)	15990 (42.84)	9845 (26.38)	25026	26	1.04
		Targets	6335	2322 (36.65)	3359 (53.02)	143 (2.26)	341 (5.38)	1482 (23.39)	2708 (42.75)	1478 (23.33)	4266	7	1.64
	UK IMRD	Comparators	231987	98811 (42.59)	131489 (56.68)	27189 (11.72)	23401 (10.09)	73122 (31.52)	76236 (32.86)	26913 (11.6)	147241	180	1.22
		Targets	27306	11696 (42.83)	15597 (57.12)	3027 (11.09)	2860 (10.47)	8666 (31.74)	9172 (33.59)	3434 (12.58)	16938	17	1
	US PharMetrics Plus	Comparators	476387	219277 (46.03)	253894 (53.3)	54964 (11.54)	62421 (13.1)	143656 (30.16)	171625 (36.03)	30420 (6.39)	328508	1907	5.81
		Targets	140527	48112 (34.24)	54822 (39.01)	9258 (6.59)	14187 (10.1)	31807 (22.63)	38337 (27.28)	5761 (4.1)	73120	409	5.59
Substance misuse or dependence	France IQVIA	Comparators	277061	118212 (42.67)	157987 (57.02)	25641 (9.25)	22491 (8.12)	77445 (27.95)	97289 (35.11)	47164 (17.02)	193569	765	3.95
		Targets	40403	16667 (41.25)	23015 (56.96)	3339 (8.26)	2962 (7.33)	11326 (28.03)	14399 (35.64)	6020 (14.9)	28892	134	4.64
	Germany IQVIA	Comparators	102092	45910 (44.97)	55736 (54.59)	11506 (11.27)	9194 (9.01)	27960 (27.39)	34329 (33.63)	16324 (15.99)	69114	816	11.81
		Targets	16109	7282 (45.2)	8705 (54.04)	1457 (9.04)	1464 (9.09)	4724 (29.33)	5783 (35.9)	2228 (13.83)	10910	101	9.26
	Italy IQVIA	Comparators	37173	13677 (36.79)	20275 (54.54)	862 (2.32)	2050 (5.51)	8781 (23.62)	15940 (42.88)	9797 (26.36)	24912	60	2.41
		Targets	6307	2307 (36.58)	3350 (53.12)	143 (2.27)	339 (5.37)	1475 (23.39)	2697 (42.76)	1471 (23.32)	4243	10	2.36
	UK IMRD	Comparators	231754	98621 (42.55)	131446 (56.72)	27188 (11.73)	23346 (10.07)	73054 (31.52)	76191 (32.88)	26861 (11.59)	147054	310	2.11
		Targets	27280	11675 (42.8)	15592 (57.16)	3026 (11.09)	2854 (10.46)	8656 (31.73)	9168 (33.61)	3427 (12.56)	16921	24	1.42
	US PharMetrics Plus	Comparators	445600	203541 (45.68)	238825 (53.6)	54337 (12.19)	58630 (13.16)	133443 (29.95)	158317 (35.53)	28508 (6.4)	305302	7234	23.69
		Targets	98015	44763 (45.67)	51750 (52.8)	9144 (9.33)	13340 (13.61)	29670 (30.27)	35460 (36.18)	5352 (5.46)	68208	1386	20.32

Table S5. *Continued.* Sample size, sex and age distribution, follow-up time, the number of psychiatric and neuropsychiatric events, and incidence rate in the long-term observation period by outcome and database

Outcome	Database	Targets/ Comparators	No. of individuals after propensity score matching	Sex, N (%)		Age, N (%)					No. of follow-up years	No. of events	Incidence rate per 1000 person years
				Males	Females	<18 years	18-24 years	25-44 years	45-64 years	65+ years			
Bipolar disorders	France IQVIA	Comparators	279707	119851 (42.85)	158952 (56.83)	25653 (9.17)	22571 (8.07)	78276 (27.98)	98649 (35.27)	47427 (16.96)	195739	99	0.51
		Targets	40830	16924 (41.45)	23182 (56.78)	3341 (8.18)	2974 (7.28)	11465 (28.08)	14624 (35.82)	6056 (14.83)	29257	<10	<0.34
	Germany IQVIA	Comparators	104722	47461 (45.32)	56807 (54.25)	11545 (11.02)	9315 (8.89)	28734 (27.44)	35568 (33.96)	16790 (16.03)	71186	42	0.59
		Targets	16422	7479 (45.54)	8821 (53.71)	1461 (8.9)	1478 (9)	4821 (29.36)	5933 (36.13)	2276 (13.86)	11170	0	0
	Italy IQVIA	Comparators	37286	13774 (36.94)	20272 (54.37)	862 (2.31)	2068 (5.55)	8779 (23.55)	15965 (42.82)	9880 (26.5)	25001	12	0.48
		Targets	6329	2326 (36.75)	3347 (52.88)	143 (2.26)	341 (5.39)	1478 (23.35)	2705 (42.74)	1482 (23.42)	4260	<5	<1.17
	UK IMRD	Comparators	232458	99084 (42.62)	131667 (56.64)	27189 (11.7)	23412 (10.07)	73295 (31.53)	76434 (32.88)	26963 (11.6)	147610	42	0.28
		Targets	27351	11725 (42.87)	15613 (57.08)	3027 (11.07)	2861 (10.46)	8682 (31.74)	9194 (33.61)	3438 (12.57)	16973	<5	<0.29
	US PharMetrics Plus	Comparators	478284	222421 (46.5)	252632 (52.82)	54815 (11.46)	62477 (13.06)	144000 (30.11)	172990 (36.17)	30681 (6.41)	330126	1117	3.38
		Targets	104813	48728 (46.49)	54491 (51.99)	9230 (8.81)	14190 (13.54)	31825 (30.36)	38598 (36.83)	5786 (5.52)	73406	232	3.16
Psychoses	France IQVIA	Comparators	279561	119726 (42.83)	158998 (56.87)	25642 (9.17)	22604 (8.09)	78284 (28)	98667 (35.29)	47309 (16.92)	195617	147	0.75
		Targets	40820	16910 (41.43)	23186 (56.8)	3339 (8.18)	2977 (7.29)	11465 (28.09)	14624 (35.83)	6044 (14.81)	29243	24	0.82
	Germany IQVIA	Comparators	104222	47245 (45.33)	56514 (54.22)	11534 (11.07)	9299 (8.92)	28616 (27.46)	35421 (33.99)	16569 (15.9)	70806	145	2.05
		Targets	16360	7451 (45.54)	8792 (53.74)	1459 (8.92)	1475 (9.02)	4805 (29.37)	5913 (36.14)	2260 (13.81)	11118	22	1.98
	Italy IQVIA	Comparators	37211	13744 (36.94)	20267 (54.47)	862 (2.32)	2066 (5.55)	8804 (23.66)	15944 (42.85)	9801 (26.34)	24938	38	1.52
		Targets	6319	2315 (36.64)	3348 (52.98)	143 (2.26)	341 (5.4)	1477 (23.37)	2699 (42.71)	1473 (23.31)	4253	<5	<1.18
	UK IMRD	Comparators	232272	98990 (42.62)	131584 (56.65)	27179 (11.7)	23397 (10.07)	73249 (31.54)	76392 (32.89)	26930 (11.59)	147488	81	0.55
		Targets	27337	11716 (42.86)	15608 (57.09)	3026 (11.07)	2859 (10.46)	8683 (31.76)	9191 (33.62)	3432 (12.55)	16964	6	0.35
	US PharMetrics Plus	Comparators	482245	223456 (46.34)	255519 (52.99)	55040 (11.41)	63287 (13.12)	145788 (30.23)	174228 (36.13)	30510 (6.33)	332956	447	1.34
		Targets	105740	48985 (46.33)	55139 (52.15)	9268 (8.76)	14386 (13.61)	32215 (30.47)	38899 (36.79)	5758 (5.45)	74085	88	1.19

Table S5. *Continued.* Sample size, sex and age distribution, follow-up time, the number of psychiatric and neuropsychiatric events, and incidence rate in the long-term observation period by outcome and database

**Outcome**	**Database**	**Targets/ Comparators**	**No. of individuals after propensity score matching**	**Sex, N (%)**		**Age, N (%)**					**No. of follow-up years**	**No. of events**	**Incidence rate per 1000 person years**
				**Males**	**Females**	**<18 years**	**18-24 years**	**25-44 years**	**45-64 years**	**65+ years**			
**Personality disorders**	France IQVIA	Comparators	280291	120023 (42.82)	159381 (56.86)	25653 (9.15)	22602 (8.06)	78417 (27.98)	98977 (35.31)	47558 (16.97)	196170	55	0.28
		Targets	40914	16949 (41.43)	23240 (56.8)	3340 (8.16)	2977 (7.28)	11483 (28.07)	14671 (35.86)	6069 (14.83)	29313	14	0.48
	Germany IQVIA	Comparators	104180	47214 (45.32)	56526 (54.26)	11513 (11.05)	9281 (8.91)	28606 (27.46)	35366 (33.95)	16674 (16)	70769	205	2.9
		Targets	16338	7438 (45.53)	8780 (53.74)	1457 (8.92)	1470 (9)	4804 (29.4)	5897 (36.09)	2263 (13.85)	11105	16	1.44
	Italy IQVIA	Comparators	36695	13635 (37.16)	19889 (54.2)	861 (2.35)	2056 (5.6)	8807 (24)	15828 (43.13)	9381 (25.56)	24518	192	7.83
		Targets	6231	2301 (36.93)	3284 (52.7)	143 (2.29)	341 (5.47)	1479 (23.74)	2684 (43.07)	1407 (22.58)	4181	32	7.65
	UK IMRD	Comparators	232277	99026 (42.63)	131546 (56.63)	27189 (11.71)	23331 (10.04)	73272 (31.55)	76379 (32.88)	26984 (11.62)	147475	89	0.6
		Targets	27337	11721 (42.88)	15604 (57.08)	3027 (11.07)	2852 (10.43)	8682 (31.76)	9189 (33.61)	3441 (12.59)	16961	14	0.83
	US PharMetrics Plus	Comparators	482563	223879 (46.39)	255532 (52.95)	54891 (11.37)	63102 (13.08)	145619 (30.18)	174524 (36.17)	30985 (6.42)	333200	456	1.37
		Targets	105798	49054 (46.37)	55131 (52.11)	9249 (8.74)	14348 (13.56)	32179 (30.42)	38944 (36.81)	5860 (5.54)	74130	87	1.17
**Self-harm and suicide**	France IQVIA	Comparators	280312	120067 (42.83)	159339 (56.84)	25622 (9.14)	22596 (8.06)	78469 (27.99)	98970 (35.31)	47563 (16.97)	196187	38	0.19
		Targets	40924	16958 (41.44)	23239 (56.79)	3337 (8.15)	2974 (7.27)	11490 (28.08)	14675 (35.86)	6068 (14.83)	29326	<10	<0.34
	Germany IQVIA	Comparators	104872	47511 (45.3)	56903 (54.26)	11536 (11)	9329 (8.9)	28754 (27.42)	35629 (33.97)	16840 (16.06)	71310	7	0.1
		Targets	16441	7483 (45.51)	8837 (53.75)	1460 (8.88)	1480 (9)	4824 (29.34)	5938 (36.12)	2284 (13.89)	11182	0	0
	Italy IQVIA	Comparators	6353	13822 (217.57)	20353 (320.37)	862 (13.57)	2068 (32.55)	8846 (139.24)	16010 (252.01)	9895 (155.75)	25097	0	0
		Targets	37416	2332 (6.23)	3364 (8.99)	143 (0.38)	342 (0.91)	1485 (3.97)	2714 (7.25)	1485 (3.97)	4278	0	0
	UK IMRD	Comparators	232064	98954 (42.64)	131426 (56.63)	27065 (11.66)	23343 (10.06)	73227 (31.55)	76368 (32.91)	26925 (11.6)	147346	128	0.87
		Targets	27304	11709 (42.88)	15580 (57.06)	3011 (11.03)	2851 (10.44)	8671 (31.76)	9188 (33.65)	3435 (12.58)	16944	8	0.47
	US PharMetrics Plus	Comparators	483821	224264 (46.35)	256309 (52.98)	54960 (11.36)	63474 (13.12)	146083 (30.19)	174732 (36.12)	31034 (6.41)	344123	221	0.66
		Targets	106003	49118 (46.34)	55277 (52.15)	9248 (8.72)	14388 (13.57)	32272 (30.44)	38994 (36.79)	5871 (5.54)	74287	41	0.55

Table S5. *Continued.* Sample size, sex and age distribution, follow-up time, the number of psychiatric and neuropsychiatric events, and incidence rate in the long-term observation period by outcome and database

**Outcome**	**Database**	**Targets/ Comparators**	**No. of individuals after propensity score matching**	**Sex, N (%)**		**Age, N (%)**					**No. of follow-up years**	**No. of events**	**Incidence rate per 1000 person years**
				**Males**	**Females**	**<18 years**	**18-24 years**	**25-44 years**	**45-64 years**	**65+ years**			
**Sleep disorders**	France IQVIA	Comparators	257246	111536 (43.36)	145112 (56.41)	25098 (9.76)	21758 (8.46)	73735 (28.66)	89337 (34.73)	41208 (16.02)	177915	4128	23.2
		Targets	37294	15634 (41.92)	21008 (56.33)	3268 (8.76)	2847 (7.63)	10690 (28.66)	13124 (35.19)	5180 (13.89)	26281	804	30.59
	Germany IQVIA	Comparators	97256	44013 (45.25)	52816 (54.31)	11292 (11.61)	9016 (9.27)	27110 (27.87)	32316 (33.23)	14957 (15.38)	65270	1963	30.07
		Targets	15270	6959 (45.57)	8203 (53.72)	1425 (9.33)	1431 (9.37)	4562 (29.88)	5406 (35.4)	2020 (13.23)	10227	307	30.02
	Italy IQVIA	Comparators	34750	12954 (37.28)	18726 (53.89)	861 (2.48)	2035 (5.86)	8521 (24.52)	14937 (42.98)	8639 (24.86)	23110	456	19.73
		Targets	5894	2189 (37.14)	3096 (52.53)	142 (2.41)	337 (5.72)	1433 (24.31)	2505 (42.5)	1305 (22.14)	3925	94	23.94
	UK IMRD	Comparators	229547	97990 (42.69)	129957 (56.61)	27074 (11.79)	23239 (10.12)	72430 (31.55)	75376 (32.84)	26398 (11.5)	145543	746	5.13
		Targets	27021	11599 (42.93)	15409 (57.03)	3017 (11.17)	2838 (10.5)	8586 (31.78)	9071 (33.57)	3367 (12.46)	16730	88	5.26
	US PharMetrics Plus	Comparators	410932	188602 (45.9)	219297 (53.37)	52530 (12.78)	59794 (14.55)	128405 (31.25)	136998 (33.34)	22641 (5.51)	278095	12890	46.35
		Targets	90300	41661 (46.14)	47231 (52.3)	8864 (9.82)	13622 (15.09)	28411 (31.46)	30629 (33.92)	4267 (4.73)	62134	3019	48.59
**Dementia**	France IQVIA	Comparators	279867	119955 (42.86)	159049 (56.83)	25664 (9.17)	22612 (8.08)	78465 (28.04)	98982 (35.37)	47091 (16.83)	195839	133	0.68
		Targets	40869	16944 (41.46)	23201 (56.77)	3342 (8.18)	2979 (7.29)	11492 (28.12)	14672 (35.9)	6012 (14.71)	29279	29	0.99
	Germany IQVIA	Comparators	103231	47032 (45.56)	55788 (54.04)	11546 (11.18)	9328 (9.04)	28741 (27.84)	35513 (34.4)	15396 (14.91)	70136	252	3.59
		Targets	16248	7428 (45.72)	8700 (53.55)	1459 (8.98)	1480 (9.11)	4822 (29.68)	5920 (36.44)	2115 (13.02)	11038	40	3.62
	Italy IQVIA	Comparators	37141	13754 (37.03)	20161 (54.28)	862 (2.32)	2068 (5.57)	8846 (23.82)	15997 (43.07)	9628 (25.92)	24884	55	2.21
		Targets	6314	2322 (36.78)	3337 (52.85)	143 (2.26)	342 (5.42)	1485 (23.52)	2713 (42.97)	1447 (22.92)	4249	6	1.41
	UK IMRD	Comparators	230046	98247 (42.71)	130083 (56.55)	27189 (11.82)	23420 (10.18)	73339 (31.88)	76372 (33.2)	24871 (10.81)	146018	304	2.08
		Targets	27027	11605 (42.94)	15403 (56.99)	3025 (11.19)	2861 (10.59)	8689 (32.15)	9187 (33.99)	3124 (11.56)	16758	45	2.69
	US PharMetrics Plus	Comparators	478718	222540 (46.49)	253090 (52.87)	54941 (11.48)	63407 (13.25)	145680 (30.43)	174106 (36.37)	28197 (5.89)	330367	936	2.83
		Targets	105037	48763 (46.42)	54661 (52.04)	9254 (8.81)	14375 (13.69)	32224 (30.68)	38860 (37)	5208 (4.96)	73581	170	2.31

Table S5. *Continued.* Sample size, sex and age distribution, follow-up time, the number of psychiatric and neuropsychiatric events, and incidence rate in the long-term observation period by outcome and database

Outcome	Database	Targets/ Comparators	No. of individuals after propensity score matching	Sex, N (%)		Age, N (%)					No. of follow-up years	No. of events	Incidence rate per 1000 person years
				Males	Females	<18 years	18-24 years	25-44 years	45-64 years	65+ years			
**Neurodevelopmental disorders**	France IQVIA	Comparators	279340	119458 (42.76)	158966 (56.91)	25114 (8.99)	22437 (8.03)	78373 (28.06)	98863 (35.39)	47458 (16.99)	195424	248	1.27
		Targets	40772	16870 (41.38)	23176 (56.84)	3276 (8.03)	2956 (7.25)	11473 (28.14)	14648 (35.93)	6055 (14.85)	29204	51	1.75
	Germany IQVIA	Comparators	102550	46236 (45.09)	55898 (54.51)	10155 (9.9)	9221 (8.99)	28592 (27.88)	35485 (34.6)	16590 (16.18)	69480	606	8.72
		Targets	16163	7323 (45.31)	8725 (53.98)	1285 (7.95)	1459 (9.03)	4804 (29.72)	5907 (36.55)	2252 (13.93)	10961	65	5.93
	Italy IQVIA	Comparators	37341	13797 (36.95)	20310 (54.39)	861 (2.31)	2054 (5.5)	8821 (23.62)	15986 (42.81)	9883 (26.47)	25047	24	0.96
		Targets	6335	2322 (36.65)	3357 (52.99)	143 (2.26)	335 (5.29)	1478 (23.33)	2710 (42.78)	1484 (23.43)	4265	<5	<1.17
	UK IMRD	Comparators	231632	98542 (42.54)	131394 (56.73)	26818 (11.58)	23283 (10.05)	73136 (31.57)	76338 (32.96)	26940 (11.63)	146957	455	3.1
		Targets	27269	11672 (42.8)	15583 (57.15)	2990 (10.96)	2849 (10.45)	8665 (31.78)	9182 (33.67)	3435 (12.6)	16913	45	2.66
	US PharMetrics Plus	Comparators	456510	210863 (46.19)	242468 (53.11)	48166 (10.55)	57061 (12.5)	137577 (30.14)	38006 (8.33)	30367 (6.65)	314206	4506	14.35
		Targets	100151	46461 (46.39)	52163 (52.08)	8174 (8.16)	12979 (12.96)	30439 (30.39)	170160 (169.9)	5715 (5.71)	69894	927	13.26
**Overall**	France IQVIA	Comparators	207629	95420 (45.96)	111778 (53.84)	23590 (11.36)	19018 (9.16)	58999 (28.42)	68606 (33.04)	32574 (15.69)	140469	9822	69.92
		Targets	29658	13231 (44.61)	15893 (53.59)	3053 (10.29)	2467 (8.32)	8410 (28.36)	9891 (33.35)	4064 (13.7)	20214	1991	98.49
	Germany IQVIA	Comparators	78018	36703 (47.04)	41062 (52.63)	9472 (12.14)	7740 (9.92)	21988 (28.18)	25623 (32.84)	11263 (14.44)	50779	5227	103.92
		Targets	12219	5801 (47.48)	6349 (51.96)	1187 (9.71)	1208 (9.89)	3679 (30.11)	4304 (35.22)	1538 (12.59)	7906	840	106.25
	Italy IQVIA	Comparators	29883	11569 (38.71)	15690 (52.5)	856 (2.86)	1921 (6.43)	7653 (25.61)	12981 (43.44)	6758 (22.61)	19526	999	51.16
		Targets	5062	1962 (38.76)	2569 (50.75)	142 (2.81)	317 (6.26)	1279 (25.27)	2164 (42.75)	1022 (20.19)	3310	174	52.57
	UK IMRD	Comparators	215648	93213 (43.22)	120850 (56.04)	26313 (12.2)	21624 (10.03)	67620 (31.36)	72123 (33.44)	23576 (10.93)	135765	3747	27.6
		Targets	25359	11038 (43.53)	14294 (56.37)	2933 (11.57)	2642 (10.42)	8014 (31.6)	8684 (34.24)	2961 (11.68)	15579	431	27.67
	US PharMetrics Plus	Comparators	284248	142259 (50.05)	139645 (49.13)	40717 (14.32)	38098 (13.4)	85283 (30)	98390 (34.61)	14573 (5.13)	187380	23425	125.01
		Targets	63128	31978 (50.66)	30248 (47.92)	6888 (10.91)	9021 (14.29)	19233 (30.47)	22060 (34.94)	2684 (4.25)	42409	5087	119.95

Table S6.1. Selected baseline characteristics for France IQVIA, for the short-term risk of anxiety disorders

Characteristic	Before propensity score matching			After propensity score matching		
	Targets, %	Comparators, %	Standardized difference	Targets, %	Comparators, %	Standardized difference
**Age group (years)**						
0-4	0.7	3	-0.17	0.8	1.3	-0.05
5-9	1.8	4.5	-0.15	2	2.3	-0.02
10-14	3.2	4.5	-0.06	3.4	3.7	-0.02
15-19	5.6	4.3	0.06	5.7	6	-0.01
20-24	7.3	4.2	0.13	6.9	6.4	0.02
25-29	7.3	4.3	0.13	7	6.5	0.02
30-34	8.3	5.1	0.13	8	7.6	0.02
35-39	9	5.6	0.13	8.6	7.7	0.03
40-44	9.4	6	0.13	9	8.1	0.03
45-49	10.1	6.8	0.12	9.8	9.2	0.02
50-54	9.8	7.4	0.09	9.8	9.5	0.01
55-59	8.9	7.9	0.04	9	9.2	-0.01
60-64	6.2	7.9	-0.07	6.4	6.6	-0.01
65-69	4	7.7	-0.16	4.3	4.7	-0.02
70-74	3.7	7.9	-0.18	4.1	4.7	-0.03
75-79	2	5.2	-0.17	2.2	2.7	-0.04
80-84	1.3	4	-0.16	1.5	1.8	-0.02
85-89	0.9	2.6	-0.13	1	1.4	-0.04
90-94	0.4	1.1	-0.08	0.4	0.6	-0.03
95-99	0.1	0.2	-0.03	0.1	0.2	-0.01
**Sex**						
Female	56.4	57.6	-0.02	55.9	55.2	0.01

Table S6.1. *Continued.* Selected baseline characteristics for France IQVIA, for the short-term risk of anxiety disorders

Characteristic	Before propensity score matching			After propensity score matching		
	**Targets, %**	**Comparators, %**	**Standardized difference**	**Targets, %**	**Comparators, %**	**Standardized difference**
**Medical history (general)**						
Acute respiratory disease	16.7	6.8	0.31	15.5	18.6	-0.08
Chronic liver disease	0.1	0.1	0	0.1	0.1	-0.01
Chronic obstructive lung disease	0.9	0.8	0.01	0.9	1.3	-0.03
Crohn’s disease	0.1	0.1	0.01	0.1	0.2	-0.01
Dementia	0.1	0.1	0	0.2	0.3	-0.03
Depressive disorder	6.1	3.2	0.14	4.9	6.1	-0.05
Diabetes mellitus	5.2	3.9	0.06	5.5	7	-0.06
Gastroesophageal reflux disease	4.3	2.1	0.13	4.1	5.1	-0.05
Gastrointestinal hemorrhage	0.5	0.2	0.04	0.4	0.5	-0.01
Human immunodeficiency virus infection	0.2	0.1	0.03	0.2	0.4	-0.04
Hyperlipidemia	4	3	0.06	4	4.8	-0.04
Hypertensive disorder	11.7	10	0.06	12.2	15.6	-0.1
Lesion of liver	0.1	0.1	0	0.1	0.1	-0.01
Obesity	0.4	0.1	0.05	0.3	0.3	0
Osteoarthritis	3.8	2.3	0.09	3.6	4.5	-0.05
Pneumonia	0.8	0.3	0.07	0.8	1	-0.03
Psoriasis	1	0.6	0.05	0.9	1.1	-0.02
Renal impairment	0.3	0.2	0.02	0.3	0.5	-0.02
Rheumatoid arthritis	0.2	0.3	-0.01	0.3	0.3	-0.01
Ulcerative colitis	0.1	0.1	0.01	0.1	0.1	0
Urinary tract infectious disease	1.5	0.7	0.08	1.5	1.8	-0.03

Table S6.1. *Continued.* Selected baseline characteristics for France IQVIA, for the short-term risk of anxiety disorders

Characteristic	Before propensity score matching			After propensity score matching		
	Targets, %	Comparators, %	Standardized difference	Targets, %	Comparators, %	Standardized difference
**Medical history (cardiovascular disease)**						
Atrial fibrillation	0.1	0.3	-0.03	0.2	0.2	-0.01
Cerebrovascular disease	0.9	0.7	0.02	1	1.4	-0.04
Coronary arteriosclerosis	0.4	0.4	0	0.4	0.6	-0.02
Heart disease	3.3	3.6	-0.02	3.5	4.7	-0.06
Heart failure	0.3	0.3	-0.01	0.3	0.4	-0.02
Ischemic heart disease	1	1	0	1.1	1.5	-0.03
Peripheral vascular disease	0.2	0.2	0	0.2	0.4	-0.03
Pulmonary embolism	0.2	0.1	0.01	0.2	0.3	-0.02
Venous thrombosis	0.3	0.2	0.03	0.3	0.4	-0.01
**Medical history (neoplasms)**						
Malignant neoplasm of anorectum	0.1	0.1	0.03	0.1	0.2	0
Malignant neoplastic disease	1.1	0.8	0.03	1.1	1.7	-0.05
Malignant tumor of breast	0.3	0.2	0.01	0.3	0.5	-0.03
Malignant tumor of colon	0.2	0.1	0.03	0.1	0.2	0
Primary malignant neoplasm of prostate	0.2	0.1	0.01	0.2	0.3	-0.03
**Medication use**						
Agents acting on the renin-angiotensin system	10.5	10.1	0.01	11.2	14.2	-0.09
Antibacterials for systemic use	36.9	16.6	0.47	34.5	39.2	-0.1
Antidepressants	7.3	4.7	0.11	5.6	6.9	-0.05
Antiepileptics	2.4	1.9	0.03	2.4	3.3	-0.06
Antiinflammatory and antirheumatic products	35.2	20.2	0.34	35.1	40	-0.1
Antineoplastic agents	1	0.8	0.02	1	1.1	-0.01
Antipsoriatics	0.5	0.3	0.03	0.5	0.6	-0.02
Antithrombotic agents	8	7.7	0.01	8.4	10.8	-0.08
Beta blocking agents	6.3	6.4	-0.01	6.5	8.1	-0.06

Table S6.1. *Continued.* Selected baseline characteristics for France IQVIA, for the short-term risk of anxiety disorders

Characteristic	Before propensity score matching			After propensity score matching		
	**Targets, %**	**Comparators, %**	**Standardized difference**	**Targets, %**	**Comparators, %**	**Standardized difference**
**Medication use**						
Calcium channel blockers	5.8	5.3	0.02	6.1	7.9	-0.07
Diuretics	5.3	5.5	-0.01	5.7	7.4	-0.07
Drugs for acid-related disorders	24.5	14.4	0.26	23.9	27.8	-0.09
Drugs for obstructive airway diseases	23.1	12.7	0.27	22.6	26.4	-0.09
Drugs used in diabetes	5.5	4.3	0.06	5.7	7.3	-0.06
Immunosuppressants	0.3	0.5	-0.03	0.4	0.4	-0.01
Opioids	58.5	23.5	0.76	55.9	57.7	-0.04
Psycholeptics	15	8.6	0.2	11.1	12.8	-0.05
Psychostimulants, agents used for ADHD and nootropics	4.5	2	0.14	4.2	4.9	-0.03

Table S6.2. Selected baseline characteristics for France IQVIA, for the short-term risk of alcohol misuse or dependence

Characteristic	Before propensity score matching			After propensity score matching		
	Targets, %	Comparators, %	Standardized difference	Targets, %	Comparators, %	Standardized difference
**Age group (years)**						
0-4	0.7	2.9	-0.17	0.7	1.2	-0.05
5-9	1.8	4.5	-0.15	1.8	2.1	-0.02
10-14	3.2	4.4	-0.06	3.2	3.5	-0.01
15-19	5.6	4.4	0.06	5.3	5.6	-0.01
20-24	7.3	4.2	0.13	6.8	6.2	0.02
25-29	7.3	4.3	0.13	6.9	6.4	0.02
30-34	8.3	5.1	0.13	8	7.6	0.01
35-39	9	5.6	0.13	8.6	7.8	0.03
40-44	9.4	5.9	0.13	9.2	8.3	0.03
45-49	10.1	6.9	0.12	10	9.4	0.02
50-54	9.8	7.5	0.08	9.9	9.7	0.01
55-59	8.9	7.9	0.03	9.1	9.4	-0.01
60-64	6.2	7.8	-0.06	6.5	6.7	-0.01
65-69	4	7.7	-0.16	4.4	4.7	-0.02
70-74	3.7	7.9	-0.18	4.1	4.8	-0.03
75-79	2	5.2	-0.17	2.2	2.7	-0.03
80-84	1.3	4	-0.16	1.6	1.9	-0.02
85-89	0.9	2.6	-0.13	1	1.4	-0.04
90-94	0.4	1	-0.07	0.4	0.6	-0.03
95-99	0.1	0.2	-0.03	0.1	0.2	-0.01
**Sex**						
Female	56.4	57.4	-0.02	57.1	56.4	0.01

Table S6.2. *Continued.* Selected baseline characteristics for France IQVIA, for the short-term risk of alcohol misuse or dependence

Characteristic	Before propensity score matching			After propensity score matching		
	**Targets, %**	**Comparators, %**	**Standardized difference**	**Targets, %**	**Comparators, %**	**Standardized difference**
**Medical history (general)**						
Acute respiratory disease	16.7	6.8	0.31	16.1	18.8	-0.07
Chronic liver disease	0.1	0.1	0	0.1	0.1	-0.01
Chronic obstructive lung disease	0.9	0.7	0.02	1	1.4	-0.04
Crohn’s disease	0.1	0.1	0.01	0.1	0.2	0
Dementia	0.1	0.1	0	0.2	0.3	-0.02
Depressive disorder	6.1	3.2	0.14	6.4	7.8	-0.06
Diabetes mellitus	5.2	3.9	0.06	5.5	7.1	-0.06
Gastroesophageal reflux disease	4.3	2.1	0.13	4.5	5.6	-0.05
Gastrointestinal hemorrhage	0.5	0.3	0.04	0.5	0.6	-0.02
Human immunodeficiency virus infection	0.2	0.1	0.03	0.2	0.4	-0.04
Hyperlipidemia	4	3	0.06	4.3	5.1	-0.04
Hypertensive disorder	11.7	10	0.06	12.7	16.2	-0.1
Lesion of liver	0.1	0.1	0	0.1	0.1	-0.01
Obesity	0.4	0.1	0.05	0.3	0.4	0
Osteoarthritis	3.8	2.2	0.09	3.9	4.8	-0.04
Pneumonia	0.8	0.3	0.07	0.8	1	-0.03
Psoriasis	1	0.5	0.05	1	1.2	-0.02
Renal impairment	0.3	0.2	0.02	0.3	0.5	-0.03
Rheumatoid arthritis	0.2	0.3	-0.01	0.3	0.4	-0.01
Schizophrenia	0.1	0.1	0	0.1	0.1	0
Urinary tract infectious disease	1.5	0.6	0.08	1.5	1.9	-0.03

Table S6.2. *Continued.* Selected baseline characteristics for France IQVIA, for the short-term risk of alcohol misuse or dependence

Characteristic	Before propensity score matching			After propensity score matching		
	**Targets, %**	**Comparators, %**	**Standardized difference**	**Targets, %**	**Comparators, %**	**Standardized difference**
**Medical history (cardiovascular disease)**						
Atrial fibrillation	0.1	0.2	-0.02	0.2	0.2	-0.01
Cerebrovascular disease	0.9	0.8	0.02	1	1.4	-0.03
Coronary arteriosclerosis	0.4	0.4	0	0.5	0.6	-0.02
Heart disease	3.3	3.6	-0.02	3.6	4.8	-0.06
Heart failure	0.3	0.3	-0.01	0.3	0.4	-0.03
Peripheral vascular disease	0.2	0.2	0	0.2	0.4	-0.03
Pulmonary embolism	0.2	0.1	0.01	0.2	0.4	-0.03
Venous thrombosis	0.3	0.2	0.02	0.4	0.4	-0.01
**Medical history (neoplasms)**						
Malignant neoplastic disease	1.1	0.8	0.03	1.2	1.8	-0.04
Malignant tumor of breast	0.3	0.2	0.02	0.3	0.5	-0.02
Malignant tumor of colon	0.2	0.1	0.03	0.2	0.1	0
Primary malignant neoplasm of prostate	0.2	0.1	0.01	0.2	0.3	-0.03
**Medication use**						
Agents acting on the renin-angiotensin system	10.5	10	0.02	11.4	14.5	-0.09
Antibacterials for systemic use	36.9	16.8	0.47	35.5	39.9	-0.09
Antidepressants	7.3	4.6	0.12	7.8	9.5	-0.06
Antiepileptics	2.4	1.9	0.03	2.5	3.5	-0.06
Antiinflammatory and antirheumatic products	35.2	20.1	0.34	36.2	41.1	-0.1
Antineoplastic agents	1	0.8	0.02	1.1	1.2	-0.01
Antipsoriatics	0.5	0.3	0.03	0.5	0.7	-0.02
Antithrombotic agents	8	7.7	0.01	8.7	10.9	-0.08
Beta blocking agents	6.3	6.4	0	6.9	8.5	-0.06
Calcium channel blockers	5.8	5.3	0.02	6.4	8.1	-0.07
Diuretics	5.3	5.5	-0.01	5.8	7.7	-0.07

Table S6.2. *Continued.* Selected baseline characteristics for France IQVIA, for the short-term risk of alcohol misuse or dependence

Characteristic	Before propensity score matching			After propensity score matching		
	**Targets, %**	**Comparators, %**	**Standardized difference**	**Targets, %**	**Comparators, %**	**Standardized difference**
**Medical history (cardiovascular disease)**						
Drugs for acid-related disorders	24.5	14.3	0.26	25.3	29.5	-0.1
Drugs for obstructive airway diseases	23.1	12.7	0.27	23.4	27.2	-0.09
Drugs used in diabetes	5.5	4.3	0.06	5.9	7.4	-0.06
Immunosuppressants	0.3	0.6	-0.04	0.4	0.4	-0.01
Opioids	58.5	23.5	0.76	56.9	58.5	-0.03
Psycholeptics	15	8.6	0.2	15.7	18.9	-0.09
Psychostimulants, agents used for ADHD and nootropics	4.5	2	0.14	4.5	5.3	-0.04

Table S6.3. Selected baseline characteristics for France IQVIA, for the short-term risk of substance misuse or dependence

Characteristic	Before propensity score matching			After propensity score matching		
	Targets, %	Comparators, %	Standardized difference	Targets, %	Comparators, %	Standardized difference
**Age group (years)**						
0-4	0.7	3	-0.17	0.7	1.1	-0.04
5-9	1.8	4.4	-0.15	1.9	2.2	-0.02
10-14	3.2	4.5	-0.06	3.2	3.5	-0.02
15-19	5.6	4.3	0.06	5.3	5.5	-0.01
20-24	7.3	4.2	0.13	6.8	6.2	0.02
25-29	7.3	4.3	0.13	6.9	6.4	0.02
30-34	8.3	5.1	0.13	8	7.5	0.02
35-39	9	5.7	0.13	8.6	7.7	0.03
40-44	9.4	6	0.13	9.1	8.3	0.03
45-49	10.1	6.9	0.12	10	9.4	0.02
50-54	9.8	7.5	0.08	9.9	9.8	0
55-59	8.9	7.7	0.04	9.1	9.4	-0.01
60-64	6.2	7.8	-0.06	6.5	6.6	-0.01
65-69	4	7.6	-0.16	4.4	4.8	-0.02
70-74	3.7	7.9	-0.18	4.1	4.7	-0.03
75-79	2	5.3	-0.17	2.3	2.7	-0.03
80-84	1.3	3.9	-0.16	1.6	1.9	-0.02
85-89	0.9	2.6	-0.13	1	1.4	-0.04
90-94	0.4	1.1	-0.08	0.4	0.6	-0.03
95-99	0.1	0.2	-0.03	0.1	0.2	-0.01
**Sex**						
Female	56.4	57.3	-0.02	57.2	56.4	0.01

Table S6.3. *Continued.* Selected baseline characteristics for France IQVIA, for the short-term risk of substance misuse or dependence

**Characteristic**	**Before propensity score matching**			**After propensity score matching**		
	**Targets,**	**Comparators,**	**Standardized**	**Targets,**	**Comparators,**	**Standardized**
**Medical history (general)**						
Acute respiratory disease	16.7	6.8	0.31	16.1	18.8	-0.07
Chronic liver disease	0.1	0.1	0	0.1	0.1	0
Chronic obstructive lung disease	0.9	0.7	0.02	0.9	1.3	-0.04
Crohn’s disease	0.1	0.1	0.01	0.1	0.2	-0.01
Dementia	0.1	0.1	0	0.2	0.3	-0.03
Depressive disorder	6.1	3.2	0.14	6.3	7.8	-0.06
Diabetes mellitus	5.2	3.9	0.06	5.5	7	-0.06
Gastroesophageal reflux disease	4.3	2.1	0.13	4.4	5.7	-0.06
Gastrointestinal hemorrhage	0.5	0.3	0.04	0.5	0.6	-0.02
Human immunodeficiency virus	0.2	0.1	0.03	0.2	0.4	-0.04
Hyperlipidemia	4	3	0.05	4.2	5	-0.04
Hypertensive disorder	11.7	10	0.06	12.7	16.2	-0.1
Lesion of liver	0.1	0.1	0	0.1	0.1	-0.01
Obesity	0.4	0.1	0.05	0.3	0.3	0
Osteoarthritis	3.8	2.2	0.09	3.9	4.8	-0.05
Pneumonia	0.8	0.3	0.07	0.8	1	-0.03
Psoriasis	1	0.5	0.05	1	1.2	-0.02
Renal impairment	0.3	0.2	0.02	0.3	0.5	-0.03
Rheumatoid arthritis	0.2	0.3	-0.01	0.3	0.3	-0.01
Ulcerative colitis	0.1	0.1	0.01	0.1	0.1	0
Urinary tract infectious disease	1.5	0.6	0.08	1.5	1.9	-0.03

Table S6.3. *Continued.* Selected baseline characteristics for France IQVIA, for the short-term risk of substance misuse or dependence

**Characteristic**	**Before propensity score matching**			**After propensity score matching**		
	**Targets, %**	**Comparators, %**	**Standardized difference**	**Targets, %**	**Comparators, %**	**Standardized difference**
**Medical history (cardiovascular disease)**						
Atrial fibrillation	0.1	0.2	-0.02	0.2	0.2	-0.01
Cerebrovascular disease	0.9	0.8	0.02	1	1.4	-0.04
Coronary arteriosclerosis	0.4	0.4	0	0.5	0.6	-0.02
Heart disease	3.3	3.6	-0.02	3.6	4.9	-0.06
Heart failure	0.3	0.3	-0.01	0.3	0.4	-0.03
Ischemic heart disease	1	1	0	1.1	1.5	-0.04
Peripheral vascular disease	0.2	0.2	0.01	0.2	0.4	-0.03
Pulmonary embolism	0.2	0.1	0.02	0.2	0.3	-0.03
Venous thrombosis	0.3	0.2	0.03	0.4	0.4	-0.01
**Medical history (neoplasms)**						
Malignant neoplasm of anorectum	0.1	0.1	0.03	0.1	0.1	0
Malignant neoplastic disease	1.1	0.8	0.03	1.2	1.8	-0.04
Malignant tumor of breast	0.3	0.2	0.01	0.3	0.5	-0.02
Primary malignant neoplasm of prostate	0.2	0.1	0.01	0.2	0.3	-0.03
**Medication use**						
Agents acting on the renin-angiotensin system	10.5	10	0.02	11.4	14.5	-0.09
Antibacterials for systemic use	36.9	16.6	0.47	35.5	39.9	-0.09
Antidepressants	7.3	4.6	0.11	7.7	9.4	-0.06
Antiepileptics	2.4	1.9	0.03	2.5	3.6	-0.06
Antiinflammatory and antirheumatic products	35.2	20.1	0.34	36.1	40.9	-0.1
Antineoplastic agents	1	0.8	0.02	1.1	1.1	0
Antipsoriatics	0.5	0.3	0.03	0.5	0.7	-0.02
Antithrombotic agents	8	7.7	0.01	8.7	10.9	-0.08
Beta blocking agents	6.3	6.5	-0.01	6.9	8.5	-0.06
Calcium channel blockers	5.8	5.4	0.02	6.3	8.1	-0.07

Table S6.3. *Continued.* Selected baseline characteristics for France IQVIA, for the short-term risk of substance misuse or dependence

Characteristic	Before propensity score matching			After propensity score matching		
	**Targets, %**	**Comparators, %**	**Standardized difference**	**Targets, %**	**Comparators, %**	**Standardized difference**
**Medication use**						
Diuretics	5.3	5.6	-0.01	5.8	7.7	-0.07
Drugs for acid-related disorders	24.5	14.3	0.26	25.2	29.4	-0.09
Drugs for obstructive airway diseases	23.1	12.7	0.28	23.3	27.1	-0.09
Drugs used in diabetes	5.5	4.3	0.06	5.9	7.3	-0.06
Immunosuppressants	0.3	0.6	-0.04	0.4	0.4	-0.01
Lipid modifying agents	8.2	8.2	0	8.9	11.3	-0.08
Opioids	58.5	23.4	0.76	56.9	58.4	-0.03
Psycholeptics	15	8.6	0.2	15.5	18.6	-0.08
Psychostimulants, agents used for ADHD and nootropics	4.5	2	0.14	4.5	5.2	-0.03

Table S6.4. Selected baseline characteristics for France IQVIA, for the short-term risk of bipolar disorders

**Characteristic**	**Before propensity score matching**			**After propensity score matching**		
	**Targets, %**	**Comparators, %**	**Standardized difference**	**Targets, %**	**Comparators, %**	**Standardized difference**
**Age group (years)**						
0-4	0.7	3	-0.17	0.7	1.2	-0.04
5-9	1.8	4.5	-0.15	1.8	2.1	-0.02
10-14	3.2	4.3	-0.06	3.2	3.5	-0.02
15-19	5.6	4.3	0.06	5.3	5.6	-0.01
20-24	7.3	4.2	0.13	6.8	6.2	0.03
25-29	7.3	4.3	0.13	6.9	6.5	0.02
30-34	8.3	5.1	0.13	8	7.5	0.02
35-39	9	5.7	0.13	8.6	7.8	0.03
40-44	9.4	5.9	0.13	9.2	8.2	0.03
45-49	10.1	6.9	0.12	10	9.4	0.02
50-54	9.8	7.5	0.08	9.9	9.7	0.01
55-59	8.9	7.8	0.04	9.2	9.4	-0.01
60-64	6.2	7.8	-0.07	6.5	6.7	-0.01
65-69	4	7.7	-0.16	4.4	4.8	-0.02
70-74	3.7	7.9	-0.18	4.1	4.7	-0.03
75-79	2	5.2	-0.17	2.3	2.7	-0.03
80-84	1.3	4	-0.16	1.6	1.9	-0.02
85-89	0.9	2.6	-0.13	1	1.4	-0.04
90-94	0.4	1.1	-0.08	0.4	0.6	-0.03
95-99	0.1	0.3	-0.03	0.1	0.2	-0.01
**Sex**						
Female	56.4	57.5	-0.02	57	56.4	0.01

Table S6.4. *Continued.* Selected baseline characteristics for France IQVIA, for the short-term risk of bipolar disorders

Characteristic	Before propensity score matching			After propensity score matching		
	**Targets, %**	**Comparators, %**	**Standardized difference**	**Targets, %**	**Comparators, %**	**Standardized difference**
**Medical history (general)**						
Acute respiratory disease	16.7	6.8	0.31	16.1	18.8	-0.07
Chronic liver disease	0.1	0.1	0.01	0.1	0.1	-0.01
Chronic obstructive lung disease	0.9	0.8	0.02	1	1.4	-0.04
Crohn's disease	0.1	0.1	0.01	0.1	0.2	-0.01
Dementia	0.1	0.1	0	0.2	0.3	-0.03
Depressive disorder	6.1	3.2	0.14	6.4	7.9	-0.06
Diabetes mellitus	5.2	3.9	0.06	5.5	7	-0.06
Gastroesophageal reflux disease	4.3	2	0.13	4.5	5.6	-0.05
Gastrointestinal hemorrhage	0.5	0.3	0.04	0.5	0.6	-0.01
Human immunodeficiency virus infection	0.2	0.1	0.03	0.2	0.4	-0.04
Hyperlipidemia	4	2.9	0.06	4.2	5.1	-0.04
Hypertensive disorder	11.7	9.9	0.06	12.7	16.2	-0.1
Lesion of liver	0.1	0.1	0.01	0.1	0.1	-0.01
Obesity	0.4	0.1	0.05	0.3	0.4	0
Osteoarthritis	3.8	2.2	0.09	3.9	4.8	-0.04
Pneumonia	0.8	0.3	0.07	0.8	1	-0.03
Psoriasis	1	0.5	0.05	1	1.2	-0.02
Renal impairment	0.3	0.2	0.02	0.3	0.5	-0.03
Rheumatoid arthritis	0.2	0.3	-0.01	0.3	0.3	-0.01
Ulcerative colitis	0.1	0.1	0.01	0.1	0.1	0
Urinary tract infectious disease	1.5	0.7	0.08	1.5	1.9	-0.03

Table S6.4. *Continued.* Selected baseline characteristics for France IQVIA, for the short-term risk of bipolar disorders

**Characteristic**	**Before propensity score matching**			**After propensity score matching**		
	**Targets, %**	**Comparators, %**	**Standardized difference**	**Targets, %**	**Comparators, %**	**Standardized difference**
**Medical history (cardiovascular disease)**						
Atrial fibrillation	0.1	0.3	-0.03	0.2	0.2	-0.01
Cerebrovascular disease	0.9	0.8	0.02	1	1.4	-0.04
Coronary arteriosclerosis	0.4	0.4	0.01	0.4	0.7	-0.03
Heart disease	3.3	3.6	-0.02	3.6	4.9	-0.06
Heart failure	0.3	0.3	0	0.3	0.5	-0.03
Ischemic heart disease	1	1	0	1.1	1.5	-0.04
Pulmonary embolism	0.2	0.1	0.02	0.2	0.4	-0.03
Venous thrombosis	0.3	0.2	0.03	0.4	0.4	-0.01
**Medical history (neoplasms)**						
Malignant neoplasm of anorectum	0.1	0.1	0.02	0.1	0.2	0
Malignant neoplastic disease	1.1	0.9	0.03	1.2	1.7	-0.04
Malignant tumor of breast	0.3	0.2	0.02	0.3	0.5	-0.02
Primary malignant neoplasm of prostate	0.2	0.1	0.01	0.2	0.3	-0.03
**Medication use**						
Agents acting on the renin-angiotensin system	10.5	10	0.02	11.5	14.5	-0.09
Antibacterials for systemic use	36.9	16.7	0.47	35.5	40	-0.09
Antidepressants	7.3	4.7	0.11	7.7	9.6	-0.06
Antiepileptics	2.4	2	0.03	2.5	3.5	-0.06
Antiinflammatory and antirheumatic products	35.2	20.1	0.34	36.2	41	-0.1
Antineoplastic agents	1	0.8	0.02	1.1	1.2	-0.01
Antipsoriatics	0.5	0.3	0.03	0.5	0.7	-0.02
Antithrombotic agents	8	7.7	0.01	8.7	11	-0.08
Beta blocking agents	6.3	6.4	-0.01	6.9	8.6	-0.06
Calcium channel blockers	5.8	5.3	0.02	6.4	8.1	-0.07
Diuretics	5.3	5.5	-0.01	5.9	7.6	-0.07

Table S6.4. *Continued.* Selected baseline characteristics for France IQVIA, for the short-term risk of bipolar disorders

**Characteristic**	**Before propensity score matching**			**After propensity score matching**		
	**Targets, %**	**Comparators, %**	**Standardized difference**	**Targets, %**	**Comparators, %**	**Standardized difference**
**Medication use**						
Drugs for acid-related disorders	24.5	14.4	0.26	25.3	29.6	-0.1
Drugs for obstructive airway diseases	23.1	12.7	0.28	23.4	27.2	-0.09
Drugs used in diabetes	5.5	4.3	0.05	5.9	7.4	-0.06
Immunosuppressants	0.3	0.5	-0.03	0.4	0.4	-0.01
Lipid modifying agents	8.2	8.2	0	9	11.4	-0.08
Opioids	58.5	23.4	0.77	57	58.4	-0.03
Psycholeptics	15	8.5	0.2	15.7	18.9	-0.09
Psychostimulants, agents used for ADHD and nootropics	4.5	2	0.14	4.5	5.3	-0.04

Table S6.5. Selected baseline characteristics for France IQVIA, for the short-term risk of psychoses

Characteristic	Before propensity score matching			After propensity score matching		
	Targets, %	Comparators, %	Standardized difference	Targets, %	Comparators, %	Standardized difference
**Age group (years)**						
0-4	0.7	2.9	-0.17	0.7	1.2	-0.04
5-19	1.8	4.4	-0.15	1.8	2.2	-0.02
10-14	3.2	4.4	-0.06	3.2	3.4	-0.01
15-19	5.6	4.3	0.06	5.3	5.5	-0.01
20-24	7.3	4.2	0.13	6.8	6.3	0.02
25-29	7.3	4.4	0.13	6.9	6.4	0.02
30-34	8.3	5	0.13	8	7.6	0.01
35-39	9	5.7	0.13	8.6	7.8	0.03
40-44	9.4	5.9	0.13	9.2	8.2	0.03
45-49	10.1	6.9	0.12	10	9.4	0.02
50-54	9.8	7.4	0.09	9.9	9.7	0.01
55-59	8.9	7.8	0.04	9.2	9.4	-0.01
60-64	6.2	7.9	-0.07	6.5	6.7	-0.01
65-69	4	7.6	-0.16	4.4	4.8	-0.02
70-74	3.7	7.9	-0.18	4.1	4.7	-0.03
75-79	2	5.3	-0.17	2.2	2.7	-0.03
80-84	1.3	4	-0.17	1.6	1.8	-0.02
85-89	0.9	2.6	-0.13	1	1.4	-0.04
90-94	0.4	1.1	-0.08	0.4	0.6	-0.03
95-99	0.1	0.3	-0.03	0.1	0.2	-0.01
**Sex**						
Female	56.4	57.4	-0.02	57.1	56.6	0.01

Table S6.5. *Continued.* Selected baseline characteristics for France IQVIA, for the short-term risk of psychoses

**Characteristic**	**Before propensity score matching**			**After propensity score matching**		
	**Targets, %**	**Comparators, %**	**Standardized difference**	**Targets, %**	**Comparators, %**	**Standardized difference**
**Medical history (general)**						
Acute respiratory disease	16.7	6.8	0.31	16.1	18.8	-0.07
Chronic liver disease	0.1	0.1	0	0.1	0.1	0
Chronic obstructive lung disease	0.9	0.7	0.02	1	1.4	-0.04
Crohn's disease	0.1	0.1	0.01	0.1	0.2	-0.01
Dementia	0.1	0.1	0	0.2	0.3	-0.03
Depressive disorder	6.1	3.2	0.14	6.4	7.9	-0.06
Diabetes mellitus	5.2	4	0.06	5.5	7	-0.06
Gastroesophageal reflux disease	4.3	2.1	0.13	4.5	5.7	-0.06
Gastrointestinal hemorrhage	0.5	0.3	0.04	0.5	0.6	-0.02
Human immunodeficiency virus infection	0.2	0.1	0.04	0.2	0.4	-0.04
Hyperlipidemia	4	2.9	0.06	4.3	5.1	-0.04
Hypertensive disorder	11.7	9.9	0.06	12.7	16.3	-0.1
Obesity	0.4	0.1	0.05	0.3	0.4	0
Osteoarthritis	3.8	2.2	0.09	3.9	4.8	-0.04
Pneumonia	0.8	0.3	0.07	0.8	1	-0.03
Psoriasis	1	0.5	0.05	1	1.2	-0.02
Renal impairment	0.3	0.2	0.02	0.3	0.5	-0.03
Rheumatoid arthritis	0.2	0.3	-0.01	0.3	0.3	-0.01
Ulcerative colitis	0.1	0.1	0.01	0.1	0.1	0
Urinary tract infectious disease	1.5	0.6	0.08	1.5	2	-0.03

Table S6.5. *Continued.* Selected baseline characteristics for France IQVIA, for the short-term risk of psychoses

Characteristic	Before propensity score matching			After propensity score matching		
	Targets, %	Comparators, %	Standardized difference	Targets, %	Comparators, %	Standardized difference
**Medical history (cardiovascular disease)**						
Atrial fibrillation	0.1	0.3	-0.03	0.2	0.2	-0.01
Cerebrovascular disease	0.9	0.8	0.02	1	1.4	-0.03
Coronary arteriosclerosis	0.4	0.4	0	0.5	0.6	-0.03
Heart disease	3.3	3.6	-0.02	3.6	4.9	-0.06
Heart failure	0.3	0.3	-0.01	0.3	0.5	-0.03
Ischemic heart disease	1	1	0	1.1	1.5	-0.04
Peripheral vascular disease	0.2	0.2	0	0.2	0.4	-0.03
Pulmonary embolism	0.2	0.1	0.01	0.2	0.3	-0.02
Venous thrombosis	0.3	0.2	0.03	0.4	0.4	-0.01
**Medical history (neoplasms)**						
Malignant lymphoma	0.1	0.1	0	0.1	0.1	-0.01
Malignant neoplastic disease	1.1	0.9	0.03	1.2	1.7	-0.04
Malignant tumor of breast	0.3	0.2	0.02	0.3	0.5	-0.02
Malignant tumor of colon	0.2	0.1	0.03	0.2	0.1	0
Primary malignant neoplasm of prostate	0.2	0.1	0.01	0.2	0.3	-0.03
**Medication use**						
Agents acting on the renin-angiotensin system	10.5	10	0.02	11.5	14.6	-0.09
Antibacterials for systemic use	36.9	16.6	0.47	35.5	40	-0.09
Antidepressants	7.3	4.6	0.12	7.8	9.5	-0.06
Antiepileptics	2.4	1.9	0.03	2.5	3.5	-0.06
Antiinflammatory and antirheumatic products	35.2	20.1	0.34	36.2	41.2	-0.1
Antineoplastic agents	1	0.8	0.02	1.1	1.1	0
Antipsoriatics	0.5	0.3	0.03	0.5	0.7	-0.02
Antithrombotic agents	8	7.6	0.01	8.7	11	-0.08
Beta blocking agents	6.3	6.5	-0.01	6.9	8.6	-0.06

Table S6.5. *Continued.* Selected baseline characteristics for France IQVIA, for the short-term risk of psychoses

Characteristic	Before propensity score matching			After propensity score matching		
	**Targets, %**	**Comparators, %**	**Standardized difference**	**Targets, %**	**Comparators, %**	**Standardized difference**
**Medication use**						
Calcium channel blockers	5.8	5.4	0.02	6.4	8.2	-0.07
Diuretics	5.3	5.5	-0.01	5.9	7.7	-0.07
Drugs for acid-related disorders	24.5	14.3	0.26	25.3	29.6	-0.1
Drugs for obstructive airway diseases	23.1	12.7	0.28	23.4	27.1	-0.09
Drugs used in diabetes	5.5	4.3	0.05	5.9	7.4	-0.06
Immunosuppressants	0.3	0.5	-0.03	0.4	0.4	-0.01
Opioids	58.5	23.4	0.77	57	58.5	-0.03
Psycholeptics	15	8.6	0.2	15.7	18.9	-0.09
Psychostimulants, agents used for ADHD and nootropics	4.5	2	0.14	4.5	5.3	-0.04

Table S6.6. Selected baseline characteristics for France IQVIA, for the short-term risk of personality disorders

Characteristic	Before propensity score matching			After propensity score matching		
	Targets, %	Comparators, %	Standardized difference	Targets,%	Comparators,%	Standardized difference
**Age group (years)**						
0-4	0.7	2.9	-0.17	0.7	1.1	-0.04
5-9	1.8	4.4	-0.15	1.8	2.1	-0.02
10-14	3.2	4.4	-0.06	3.2	3.4	-0.01
15-19	5.6	4.3	0.06	5.3	5.4	-0.01
20-24	7.3	4.3	0.13	6.8	6.2	0.02
25-29	7.3	4.3	0.13	6.9	6.4	0.02
30-34	8.3	5.1	0.13	8	7.5	0.02
35-39	9	5.6	0.13	8.6	7.8	0.03
40-44	9.4	6	0.13	9.2	8.3	0.03
45-49	10.1	6.8	0.12	10	9.4	0.02
50-54	9.8	7.5	0.08	9.9	9.8	0
55-59	8.9	7.8	0.04	9.2	9.4	-0.01
60-64	6.2	7.8	-0.07	6.5	6.7	-0.01
65-69	4	7.7	-0.16	4.4	4.8	-0.02
70-74	3.7	8	-0.19	4.1	4.7	-0.03
75-79	2	5.2	-0.17	2.3	2.7	-0.03
80-84	1.3	4	-0.16	1.6	1.8	-0.02
85-89	0.9	2.6	-0.13	1	1.4	-0.04
90-94	0.4	1.1	-0.08	0.4	0.6	-0.03
95-99	0.1	0.2	-0.03	0.1	0.2	-0.01
**Sex**						
Female	56.4	57.3	-0.02	57	56.3	0.01

Table S6.6. *Continued.* Selected baseline characteristics for France IQVIA, for the short-term risk of personality disorders

Characteristic	Before propensity score matching			After propensity score matching		
	Targets, %	Comparators, %	Standardized difference	Targets, %	Comparators, %	Standardized difference
**Medical history (general)**						
Acute respiratory disease	16.7	6.8	0.31	16.1	18.9	-0.07
Chronic liver disease	0.1	0.1	0	0.1	0.1	-0.01
Chronic obstructive lung disease	0.9	0.8	0.01	1	1.4	-0.04
Crohn’s disease	0.1	0.1	0.01	0.1	0.2	0
Dementia	0.1	0.1	0	0.2	0.3	-0.02
Depressive disorder	6.1	3.2	0.14	6.4	7.9	-0.06
Diabetes mellitus	5.2	3.9	0.06	5.5	7	-0.06
Gastroesophageal reflux disease	4.3	2.1	0.12	4.5	5.6	-0.05
Gastrointestinal hemorrhage	0.5	0.3	0.04	0.5	0.6	-0.01
Human immunodeficiency virus infection	0.2	0.1	0.03	0.2	0.4	-0.05
Hyperlipidemia	4	3	0.06	4.3	5.1	-0.04
Hypertensive disorder	11.7	10	0.06	12.7	16.2	-0.1
Lesion of liver	0.1	0.1	0	0.1	0.1	-0.01
Obesity	0.4	0.1	0.05	0.3	0.4	0
Osteoarthritis	3.8	2.2	0.09	3.9	4.9	-0.05
Pneumonia	0.8	0.3	0.08	0.8	1	-0.02
Psoriasis	1	0.6	0.04	1	1.3	-0.02
Renal impairment	0.3	0.2	0.02	0.3	0.5	-0.03
Rheumatoid arthritis	0.2	0.3	-0.01	0.3	0.3	-0.01
Ulcerative colitis	0.1	0.1	0.01	0.1	0.1	0
Urinary tract infectious disease	1.5	0.7	0.08	1.5	1.9	-0.03

Table S6.6. *Continued.* Selected baseline characteristics for France IQVIA, for the short-term risk of personality disorders

Characteristic	Before propensity score matching			After propensity score matching		
	Targets, %	Comparators, %	Standardized difference	Targets, %	Comparators, %	Standardized difference
**Medical history (cardiovascular disease)**						
Atrial fibrillation	0.1	0.3	-0.03	0.2	0.2	-0.01
Cerebrovascular disease	0.9	0.8	0.02	1	1.4	-0.03
Coronary arteriosclerosis	0.4	0.4	0	0.5	0.6	-0.02
Heart disease	3.3	3.6	-0.02	3.6	4.8	-0.06
Heart failure	0.3	0.3	-0.01	0.3	0.4	-0.02
Ischemic heart disease	1	1	0	1.1	1.5	-0.04
Pulmonary embolism	0.2	0.1	0.02	0.2	0.3	-0.02
Venous thrombosis	0.3	0.2	0.03	0.4	0.4	-0.01
**Medical history (neoplasms)**						
Malignant neoplasm of anorectum	0.1	0.1	0.02	0.1	0.2	0
Malignant neoplastic disease	1.1	0.9	0.03	1.2	1.8	-0.04
Malignant tumor of breast	0.3	0.2	0.02	0.3	0.5	-0.02
Malignant tumor of colon	0.2	0.1	0.02	0.2	0.2	0
Primary malignant neoplasm of prostate	0.2	0.1	0.01	0.2	0.3	-0.03
**Medication use**						
Agents acting on the renin-angiotensin system	10.5	10.1	0.01	11.5	14.5	-0.09
Antibacterials for systemic use	36.9	16.7	0.47	35.5	39.9	-0.09
Antidepressants	7.3	4.7	0.11	7.8	9.6	-0.06
Antiepileptics	2.4	2	0.03	2.6	3.6	-0.06
Antiinflammatory and antirheumatic products	35.2	20.1	0.34	36.2	41.2	-0.1
Antineoplastic agents	1	0.8	0.03	1.1	1.2	-0.01
Antipsoriatics	0.5	0.3	0.03	0.5	0.7	-0.02
Antithrombotic agents	8	7.6	0.01	8.7	11	-0.08
Beta blocking agents	6.3	6.4	-0.01	6.9	8.5	-0.06
Calcium channel blockers	5.8	5.2	0.03	6.4	8.2	-0.07

Table S6.6. *Continued.* Selected baseline characteristics for France IQVIA, for the short-term risk of personality disorders

**Characteristic**	**Before propensity score matching**			**After propensity score matching**		
	**Targets, %**	**Comparators, %**	**Standardized difference**	**Targets, %**	**Comparators, %**	**Standardized difference**
**Medication use**						
Diuretics	5.3	5.5	-0.01	5.9	7.6	-0.07
Drugs for acid-related disorders	24.5	14.3	0.26	25.4	29.5	-0.09
Drugs for obstructive airway diseases	23.1	12.7	0.28	23.4	27.3	-0.09
Drugs used in diabetes	5.5	4.3	0.06	5.9	7.4	-0.06
Immunosuppressants	0.3	0.5	-0.03	0.4	0.4	-0.01
Opioids	58.5	23.2	0.77	57	58.4	-0.03
Psycholeptics	15	8.6	0.2	15.8	19	-0.09
Psychostimulants, agents used for ADHD and nootropics	4.5	2	0.14	4.5	5.3	-0.04

Table S6.7. Selected baseline characteristics for France IQVIA, for the short-term risk of self-harm and suicide

Characteristic	Before propensity score matching			After propensity score matching		
	Targets, %	Comparators, %	Standardized difference	Targets, %	Comparators, %	Standardized difference
**Age group (years)**						
0-4	0.7	2.9	-0.17	0.7	1.2	-0.04
5-9	1.8	4.4	-0.15	1.8	2.1	-0.02
10-14	3.2	4.4	-0.06	3.2	3.4	-0.01
15-19	5.6	4.4	0.06	5.3	5.5	-0.01
20-24	7.3	4.2	0.13	6.8	6.3	0.02
25-29	7.3	4.4	0.12	6.9	6.5	0.01
30-34	8.3	5	0.13	8	7.4	0.02
35-39	9	5.6	0.13	8.6	7.8	0.03
40-44	9.4	5.9	0.13	9.2	8.2	0.04
45-49	10.1	6.9	0.12	10	9.5	0.02
50-54	9.8	7.4	0.09	9.9	9.7	0.01
55-59	8.9	7.9	0.04	9.2	9.4	-0.01
60-64	6.2	7.8	-0.06	6.5	6.7	-0.01
65-69	4	7.7	-0.16	4.4	4.7	-0.02
70-74	3.7	8	-0.18	4.1	4.8	-0.03
75-79	2	5.1	-0.17	2.2	2.7	-0.03
80-84	1.3	4	-0.16	1.6	1.8	-0.02
85-89	0.9	2.6	-0.13	1	1.4	-0.04
90-94	0.4	1.1	-0.08	0.4	0.6	-0.03
95-99	0.1	0.3	-0.04	0.1	0.2	-0.01
**Sex**						
Female	56.4	57.5	-0.02	57	56.3	0.01

Table S6.7. *Continued.* Selected baseline characteristics for France IQVIA, for the short-term risk of self-harm and suicide

Characteristic	Before propensity score matching			After propensity score matching		
	Targets, %	Comparators, %	Standardized difference	Targets, %	Comparators, %	Standardized difference
**Medical history (general)**						
Acute respiratory disease	16.7	6.8	0.31	16.1	18.7	-0.07
Chronic liver disease	0.1	0.1	0	0.1	0.1	0
Chronic obstructive lung disease	0.9	0.8	0.02	1	1.4	-0.04
Crohn’s disease	0.1	0.1	0.01	0.1	0.2	0
Dementia	0.1	0.1	0	0.2	0.3	-0.02
Depressive disorder	6.1	3.2	0.14	6.4	8	-0.06
Diabetes mellitus	5.2	3.9	0.06	5.5	7.1	-0.06
Gastroesophageal reflux disease	4.3	2	0.13	4.5	5.7	-0.06
Gastrointestinal hemorrhage	0.5	0.3	0.04	0.5	0.6	-0.01
Human immunodeficiency virus infection	0.2	0.1	0.04	0.2	0.4	-0.04
Hyperlipidemia	4	3	0.06	4.3	5	-0.04
Hypertensive disorder	11.7	9.9	0.06	12.7	16.2	-0.1
Lesion of liver	0.1	0.1	0	0.1	0.1	-0.01
Obesity	0.4	0.1	0.05	0.3	0.4	0
Osteoarthritis	3.8	2.2	0.09	3.9	4.9	-0.05
Pneumonia	0.8	0.3	0.07	0.8	1.1	-0.03
Psoriasis	1	0.6	0.05	1	1.2	-0.02
Renal impairment	0.3	0.2	0.02	0.3	0.5	-0.03
Rheumatoid arthritis	0.2	0.3	-0.01	0.3	0.3	-0.01
Ulcerative colitis	0.1	0.1	0.01	0.1	0.1	0
Urinary tract infectious disease	1.5	0.7	0.08	1.5	1.9	-0.03

Table S6.7. *Continued.* Selected baseline characteristics for France IQVIA, for the short-term risk of self-harm and suicide

**Characteristic**	**Before propensity score matching**			**After propensity score matching**		
	**Targets, %**	**Comparators, %**	**Standardized difference**	**Targets, %**	**Comparators, %**	**Standardized difference**
**Medical history (cardiovascular disease)**						
Atrial fibrillation	0.1	0.3	-0.03	0.2	0.2	-0.02
Cerebrovascular disease	0.9	0.8	0.02	1	1.4	-0.03
Coronary arteriosclerosis	0.4	0.4	0	0.5	0.6	-0.02
Heart disease	3.3	3.7	-0.02	3.6	4.9	-0.06
Heart failure	0.3	0.3	-0.01	0.3	0.5	-0.03
Ischemic heart disease	1	1	0	1.1	1.5	-0.04
Peripheral vascular disease	0.2	0.2	0	0.2	0.4	-0.03
Pulmonary embolism	0.2	0.1	0.01	0.2	0.3	-0.02
Venous thrombosis	0.3	0.2	0.03	0.4	0.4	-0.01
**Medical history (neoplasms)**						
Malignant neoplasm of anorectum	0.1	0.1	0.03	0.1	0.2	0
Malignant neoplastic disease	1.1	0.9	0.03	1.2	1.8	-0.04
Malignant tumor of breast	0.3	0.2	0.01	0.3	0.5	-0.02
Malignant tumor of colon	0.2	0.1	0.03	0.2	0.2	0
Primary malignant neoplasm of prostate	0.2	0.1	0.01	0.2	0.3	-0.03
**Medication use**						
Agents acting on the renin-angiotensin system	10.5	9.9	0.02	11.5	14.5	-0.09
Antibacterials for systemic use	36.9	16.6	0.47	35.5	39.9	-0.09
Antidepressants	7.3	4.7	0.11	7.8	9.6	-0.06
Antiepileptics	2.4	1.9	0.03	2.6	3.5	-0.06
Antiinflammatory and antirheumatic products	35.2	20.1	0.34	36.2	41	-0.1
Antineoplastic agents	1	0.8	0.03	1.1	1.1	-0.01
Antipsoriatics	0.5	0.3	0.03	0.5	0.7	-0.02
Antithrombotic agents	8	7.6	0.01	8.7	11	-0.08
Beta blocking agents	6.3	6.5	-0.01	6.9	8.6	-0.06

Table S6.7. *Continued.* Selected baseline characteristics for France IQVIA, for the short-term risk of self-harm and suicide

Characteristic	Before propensity score matching			After propensity score matching		
	Targets, %	Comparators, %	Standardized difference	Targets, %	Comparators, %	Standardized difference
**Medication use**						
Calcium channel blockers	5.8	5.3	0.02	6.4	8.1	-0.07
Diuretics	5.3	5.5	-0.01	5.9	7.7	-0.07
Drugs for acid-related disorders	24.5	14.2	0.26	25.4	29.5	-0.09
Drugs for obstructive airway diseases	23.1	12.7	0.27	23.4	27.1	-0.09
Drugs used in diabetes	5.5	4.3	0.06	5.9	7.5	-0.06
Immunosuppressants	0.3	0.5	-0.03	0.4	0.4	-0.01
Lipid modifying agents	8.2	8.2	0	9	11.5	-0.08
Opioids	58.5	23.5	0.76	57	58.4	-0.03
Psycholeptics	15	8.6	0.2	15.8	19	-0.09
Psychostimulants, agents used for ADHD and nootropics	4.5	2	0.14	4.5	5.3	-0.04

Table S6.8. Selected baseline characteristics for France IQVIA, for the short-term risk of sleep disorders

**Characteristic**	**Before propensity score matching**			**After propensity score matching**		
	**Targets, %**	**Comparators, %**	**Standardized difference**	**Targets,%**	**Comparators,%**	**Standardized difference**
**Age group (years)**						
0-4	0.7	3	-0.17	0.8	1.2	-0.04
5-9	1.8	4.4	-0.15	1.9	2.2	-0.02
10-14	3.2	4.4	-0.06	3.3	3.6	-0.01
15-19	5.6	4.4	0.06	5.5	5.7	-0.01
20-24	7.3	4.2	0.14	6.9	6.4	0.02
25-29	7.3	4.3	0.13	7	6.6	0.02
30-34	8.3	5	0.13	8.2	7.7	0.02
35-39	9	5.7	0.13	8.7	7.9	0.03
40-44	9.4	5.9	0.13	9.2	8.3	0.03
45-49	10.1	6.9	0.12	10.1	9.4	0.02
50-54	9.8	7.5	0.08	9.8	9.5	0.01
55-59	8.9	7.9	0.04	9.1	9.3	-0.01
60-64	6.2	7.8	-0.07	6.4	6.6	-0.01
65-69	4	7.7	-0.16	4.3	4.6	-0.01
70-74	3.7	8	-0.18	3.9	4.6	-0.03
75-79	2	5.2	-0.17	2.1	2.6	-0.04
80-84	1.3	3.9	-0.16	1.4	1.7	-0.02
85-89	0.9	2.6	-0.13	0.9	1.3	-0.04
90-94	0.4	1	-0.07	0.4	0.6	-0.03
95-99	0.1	0.2	-0.03	0.1	0.2	-0.01
**Sex**						
Female	56.4	57.3	-0.02	56.7	56	0.01

Table S6.8. *Continued.* Selected baseline characteristics for France IQVIA, for the short-term risk of sleep disorders

Characteristic	Before propensity score matching			After propensity score matching		
	Targets, %	Comparators, %	Standardized difference	Targets, %	Comparators, %	Standardized difference
**Medical history (general)**						
Acute respiratory disease	16.7	6.9	0.31	15.8	18.8	-0.08
Chronic liver disease	0.1	0.1	0.01	0.1	0.1	-0.01
Chronic obstructive lung disease	0.9	0.8	0.01	0.9	1.2	-0.03
Crohn’s disease	0.1	0.1	0.01	0.1	0.1	0
Dementia	0.1	0.1	0	0.2	0.3	-0.02
Depressive disorder	6.1	3.1	0.14	5.6	7.1	-0.06
Diabetes mellitus	5.2	4	0.06	5.3	6.9	-0.07
Gastroesophageal reflux disease	4.3	2	0.13	4.2	5.3	-0.05
Gastrointestinal hemorrhage	0.5	0.3	0.04	0.5	0.5	-0.01
Human immunodeficiency virus infection	0.2	0.1	0.03	0.2	0.4	-0.04
Hyperlipidemia	4	2.9	0.06	4	4.8	-0.04
Hypertensive disorder	11.7	9.9	0.06	12.1	15.5	-0.1
Lesion of liver	0.1	0.1	0	0.1	0.1	-0.01
Obesity	0.4	0.1	0.05	0.3	0.3	0
Osteoarthritis	3.8	2.2	0.09	3.6	4.5	-0.04
Pneumonia	0.8	0.3	0.07	0.8	1	-0.03
Psoriasis	1	0.5	0.05	0.9	1.1	-0.02
Renal impairment	0.3	0.2	0.02	0.3	0.4	-0.02
Rheumatoid arthritis	0.2	0.3	-0.01	0.2	0.3	-0.02
Ulcerative colitis	0.1	0.1	0.01	0.1	0.1	0
Urinary tract infectious disease	1.5	0.7	0.08	1.5	1.8	-0.03

Table S6.8. *Continued.* Selected baseline characteristics for France IQVIA, for the short-term risk of sleep disorders

Characteristic	Before propensity score matching			After propensity score matching		
	Targets, %	Comparators, %	Standardized difference	Targets, %	Comparators, %	Standardized difference
**Medical history (cardiovascular disease)**						
Atrial fibrillation	0.1	0.2	-0.02	0.1	0.2	-0.01
Cerebrovascular disease	0.9	0.8	0.02	0.9	1.4	-0.04
Coronary arteriosclerosis	0.4	0.4	0	0.4	0.6	-0.02
Heart disease	3.3	3.6	-0.02	3.4	4.6	-0.06
Heart failure	0.3	0.3	-0.01	0.3	0.4	-0.02
Ischemic heart disease	1	1	0	1	1.4	-0.04
Peripheral vascular disease	0.2	0.2	0	0.2	0.3	-0.03
Pulmonary embolism	0.2	0.1	0.02	0.2	0.3	-0.03
Venous thrombosis	0.3	0.2	0.03	0.3	0.4	-0.01
**Medical history (neoplasms)**						
Malignant neoplasm of anorectum	0.1	0.1	0.02	0.1	0.2	0
Malignant neoplastic disease	1.1	0.9	0.03	1.1	1.7	-0.04
Malignant tumor of breast	0.3	0.2	0.02	0.3	0.4	-0.02
Malignant tumor of colon	0.2	0.1	0.03	0.1	0.2	0
Primary malignant neoplasm of prostate	0.2	0.1	0.01	0.2	0.3	-0.03
**Medication use**						
Agents acting on the renin-angiotensin system	10.5	10	0.02	11	13.9	-0.09
Antibacterials for systemic use	36.9	16.9	0.46	34.9	39.4	-0.09
Antidepressants	7.3	4.6	0.11	6.9	8.7	-0.06
Antiepileptics	2.4	1.9	0.03	2.3	3.4	-0.06
Antiinflammatory and antirheumatic products	35.2	20.1	0.34	35.3	40.2	-0.1
Antineoplastic agents	1	0.8	0.02	1	1.1	-0.01
Antipsoriatics	0.5	0.3	0.03	0.5	0.6	-0.01
Antithrombotic agents	8	7.7	0.01	8.2	10.5	-0.08
Beta blocking agents	6.3	6.4	0	6.5	8.2	-0.06

Table S6.8. *Continued.* Selected baseline characteristics for France IQVIA, for the short-term risk of sleep disorders

Characteristic	Before propensity score matching			After propensity score matching		
	**Targets, %**	**Comparators, %**	**Standardized difference**	**Targets, %**	**Comparators, %**	**Standardized difference**
**Medication use**						
Calcium channel blockers	5.8	5.3	0.03	6	7.8	-0.07
Diuretics	5.3	5.4	-0.01	5.5	7.3	-0.07
Drugs for acid-related disorders	24.5	14.3	0.26	24.3	28.3	-0.09
Drugs for obstructive airway diseases	23.1	12.7	0.27	22.8	26.5	-0.09
Drugs used in diabetes	5.5	4.3	0.05	5.6	7.2	-0.07
Immunosuppressants	0.3	0.5	-0.03	0.3	0.4	-0.01
Lipid modifying agents	8.2	8.1	0	8.5	10.9	-0.08
Opioids	58.5	23.5	0.76	56.3	57.9	-0.03
Psycholeptics	15	8.6	0.2	12.9	15.4	-0.07
Psychostimulants, agents used for ADHD and nootropics	4.5	2	0.14	4.3	5.1	-0.04

Table S6.9. Selected baseline characteristics for France IQVIA, for the short-term risk of dementia

Characteristic	Before propensity score matching			After propensity score matching		
	Targets, %	Comparators, %	Standardized difference	Targets, %	Comparators, %	Standardized difference
**Age group (years)**						
0-4	0.7	2.9	-0.17	0.7	1.1	-0.04
5-9	1.8	4.5	-0.15	1.8	2.1	-0.02
10-14	3.2	4.4	-0.06	3.2	3.4	-0.01
15-19	5.6	4.3	0.06	5.3	5.6	-0.01
20-24	7.3	4.2	0.14	6.8	6.3	0.02
25-29	7.3	4.3	0.13	6.9	6.5	0.02
30-34	8.3	5	0.13	8	7.6	0.01
35-39	9	5.7	0.13	8.7	7.8	0.03
40-44	9.4	5.9	0.13	9.2	8.3	0.03
45-49	10.1	6.8	0.12	10.1	9.5	0.02
50-54	9.8	7.5	0.08	10	9.7	0.01
55-59	8.9	7.8	0.04	9.2	9.4	-0.01
60-64	6.2	7.8	-0.06	6.5	6.7	0
65-69	4	7.6	-0.16	4.4	4.7	-0.02
70-74	3.7	8	-0.19	4.1	4.7	-0.03
75-79	2	5.3	-0.17	2.2	2.6	-0.03
80-84	1.3	4	-0.16	1.5	1.8	-0.02
85-89	0.9	2.6	-0.13	0.9	1.4	-0.04
90-94	0.4	1.1	-0.08	0.4	0.6	-0.03
95-99	0.1	0.3	-0.03	0.1	0.2	-0.01
**Sex**						
Female	56.4	57.5	-0.02	57	56.3	0.01

Table S6.9. *Continued.* Selected baseline characteristics for France IQVIA, for the short-term risk of dementia

Characteristic	Before propensity score matching			After propensity score matching		
	Targets, %	Comparators, %	Standardized difference	Targets, %	Comparators, %	Standardized difference
**Medical history (general)**						
Acute respiratory disease	16.7	6.8	0.31	16.1	18.9	-0.07
Chronic liver disease	0.1	0.1	0	0.1	0.1	0
Chronic obstructive lung disease	0.9	0.8	0.01	1	1.3	-0.03
Crohn’s disease	0.1	0.1	0.01	0.1	0.2	0
Depressive disorder	6.1	3.2	0.14	6.4	7.9	-0.06
Diabetes mellitus	5.2	4	0.06	5.5	7	-0.06
Gastroesophageal reflux disease	4.3	2.1	0.12	4.5	5.6	-0.05
Gastrointestinal hemorrhage	0.5	0.3	0.04	0.5	0.6	-0.01
Human immunodeficiency virus infection	0.2	0.1	0.03	0.2	0.4	-0.04
Hyperlipidemia	4	3	0.06	4.2	5	-0.04
Hypertensive disorder	11.7	9.9	0.06	12.7	16	-0.1
Lesion of liver	0.1	0.1	0	0.1	0.1	-0.01
Obesity	0.4	0.1	0.05	0.3	0.4	0
Osteoarthritis	3.8	2.2	0.09	3.9	4.8	-0.05
Pneumonia	0.8	0.3	0.07	0.8	1.1	-0.03
Psoriasis	1	0.5	0.05	1	1.2	-0.02
Renal impairment	0.3	0.2	0.02	0.3	0.5	-0.03
Rheumatoid arthritis	0.2	0.3	-0.01	0.3	0.3	-0.01
Ulcerative colitis	0.1	0.1	0.01	0.1	0.1	0
Urinary tract infectious disease	1.5	0.7	0.08	1.5	1.9	-0.03

Table S6.9. *Continued.* Selected baseline characteristics for France IQVIA, for the short-term risk of dementia

**Characteristic**	**Before propensity score matching**			**After propensity score matching**		
	**Targets, %**	**Comparators, %**	**Standardized difference**	**Targets, %**	**Comparators, %**	**Standardized difference**
**Medical history (cardiovascular disease)**						
Atrial fibrillation	0.1	0.3	-0.03	0.2	0.2	-0.01
Cerebrovascular disease	0.9	0.8	0.02	1	1.4	-0.03
Coronary arteriosclerosis	0.4	0.4	0.01	0.5	0.6	-0.02
Heart disease	3.3	3.7	-0.02	3.6	4.8	-0.06
Heart failure	0.3	0.3	-0.01	0.3	0.4	-0.02
Peripheral vascular disease	0.2	0.2	0	0.2	0.4	-0.03
Pulmonary embolism	0.2	0.1	0.02	0.2	0.3	-0.03
Venous thrombosis	0.3	0.2	0.03	0.4	0.4	-0.01
**Medical history (neoplasms)**						
Malignant neoplasm of anorectum	0.1	0.1	0.02	0.1	0.1	0
Malignant neoplastic disease	1.1	0.9	0.03	1.2	1.7	-0.04
Malignant tumor of breast	0.3	0.2	0.01	0.3	0.5	-0.02
Malignant tumor of colon	0.2	0.1	0.03	0.2	0.1	0
Primary malignant neoplasm of prostate	0.2	0.1	0.01	0.2	0.3	-0.03
**Medication use**						
Agents acting on the renin-angiotensin system	10.5	10	0.02	11.4	14.3	-0.09
Antibacterials for systemic use	36.9	16.8	0.47	35.5	40	-0.09
Antidepressants	7.3	4.8	0.11	7.8	9.5	-0.06
Antiepileptics	2.4	2	0.03	2.5	3.5	-0.06
Antiinflammatory and antirheumatic products	35.2	20.1	0.34	36.2	40.9	-0.1
Antineoplastic agents	1	0.8	0.02	1.1	1.1	-0.01
Antipsoriatics	0.5	0.3	0.03	0.5	0.7	-0.02
Antithrombotic agents	8	7.7	0.01	8.7	10.8	-0.07
Beta blocking agents	6.3	6.4	-0.01	6.9	8.4	-0.06

Table S6.9. *Continued.* Selected baseline characteristics for France IQVIA, for the short-term risk of dementia

Characteristic	Before propensity score matching			After propensity score matching		
	Targets, %	Comparators, %	Standardized difference	Targets, %	Comparators, %	Standardized difference
**Medication use**						
Calcium channel blockers	5.8	5.3	0.02	6.4	8.1	-0.07
Diuretics	5.3	5.5	-0.01	5.8	7.5	-0.07
Drugs for acid-related disorders	24.5	14.3	0.26	25.3	29.4	-0.09
Drugs for obstructive airway diseases	23.1	12.9	0.27	23.4	27.1	-0.09
Drugs used in diabetes	5.5	4.3	0.05	5.9	7.4	-0.06
Immunosuppressants	0.3	0.5	-0.03	0.4	0.4	-0.01
Opioids	58.5	23.4	0.76	57	58.5	-0.03
Psycholeptics	15	8.7	0.2	15.8	18.9	-0.08
Psychostimulants, agents used for ADHD and nootropics	4.5	2	0.14	4.5	5.2	-0.03

Table S6.10. Selected baseline characteristics for France IQVIA, for the short-term risk of neurodevelopmental disorders

**Characteristic**	**Before propensity score matching**			**After propensity score matching**		
	**Targets, %**	**Comparators, %**	**Standardized difference**	**Targets, %**	**Comparators, %**	**Standardized difference**
**Age group (years)**						
0-4	0.7	3	-0.17	0.7	1.1	-0.04
5-9	1.8	4.4	-0.15	1.8	2.2	-0.03
10-14	3.2	4.4	-0.06	3.1	3.3	-0.01
15-19	5.6	4.3	0.06	5.3	5.5	-0.01
20-24	7.3	4.3	0.13	6.8	6.2	0.02
25-29	7.3	4.3	0.13	6.9	6.4	0.02
30-34	8.3	5	0.13	8	7.5	0.02
35-39	9	5.5	0.13	8.6	7.7	0.03
40-44	9.4	5.9	0.13	9.2	8.2	0.03
45-49	10.1	6.9	0.12	10.1	9.5	0.02
50-54	9.8	7.5	0.08	9.9	9.8	0
55-59	8.9	7.9	0.03	9.2	9.4	-0.01
60-64	6.2	7.8	-0.06	6.5	6.8	-0.01
65-69	4	7.7	-0.16	4.4	4.7	-0.02
70-74	3.7	7.9	-0.18	4.1	4.7	-0.03
75-79	2	5.2	-0.17	2.2	2.7	-0.03
80-84	1.3	4	-0.16	1.6	1.8	-0.02
85-89	0.9	2.6	-0.13	1	1.4	-0.04
90-94	0.4	1.1	-0.08	0.4	0.6	-0.03
95-99	0.1	0.2	-0.03	0.1	0.2	-0.01
**Sex**						
Female	56.4	57.4	-0.02	57.1	56.4	0.01

Table S6.10. *Continued.* Selected baseline characteristics for France IQVIA, for the short-term risk of neurodevelopmental disorders

Characteristic	Before propensity score matching			After propensity score matching		
	Targets, %	Comparators, %	Standardized difference	Targets, %	Comparators, %	Standardized difference
**Medical history (general)**						
Acute respiratory disease	16.7	6.9	0.31	16.1	18.9	-0.07
Chronic liver disease	0.1	0.1	0	0.1	0.1	0
Chronic obstructive lung disease	0.9	0.7	0.02	1	1.3	-0.03
Crohn's disease	0.1	0.1	0.01	0.1	0.2	-0.01
Dementia	0.1	0.1	0	0.2	0.3	-0.02
Depressive disorder	6.1	3.2	0.14	6.4	7.9	-0.06
Diabetes mellitus	5.2	3.9	0.06	5.5	7.1	-0.06
Gastroesophageal reflux disease	4.3	2.1	0.13	4.5	5.6	-0.05
Gastrointestinal hemorrhage	0.5	0.3	0.04	0.5	0.6	-0.01
Human immunodeficiency virus infection	0.2	0.1	0.04	0.2	0.4	-0.04
Hyperlipidemia	4	2.9	0.06	4.3	5.1	-0.04
Hypertensive disorder	11.7	9.9	0.06	12.7	16.2	-0.1
Lesion of liver	0.1	0.1	0	0.1	0.1	-0.01
Obesity	0.4	0.1	0.05	0.3	0.4	0
Osteoarthritis	3.8	2.2	0.09	3.9	4.8	-0.04
Pneumonia	0.8	0.3	0.07	0.8	1	-0.03
Psoriasis	1	0.5	0.05	1	1.3	-0.02
Renal impairment	0.3	0.2	0.02	0.3	0.5	-0.03
Rheumatoid arthritis	0.2	0.3	-0.01	0.3	0.3	-0.01
Ulcerative colitis	0.1	0.1	0.01	0.1	0.1	0
Urinary tract infectious disease	1.5	0.7	0.07	1.5	1.9	-0.03

Table S6.10. *Continued.* Selected baseline characteristics for France IQVIA, for the short-term risk of neurodevelopmental disorders

Characteristic	Before propensity score matching			After propensity score matching		
	**Targets, %**	**Comparators, %**	**Standardized difference**	**Targets, %**	**Comparators, %**	**Standardized difference**
**Medical history (cardiovascular disease)**						
Atrial fibrillation	0.1	0.3	-0.03	0.2	0.2	-0.01
Cerebrovascular disease	0.9	0.8	0.02	1	1.4	-0.04
Coronary arteriosclerosis	0.4	0.4	0	0.5	0.6	-0.02
Heart disease	3.3	3.6	-0.02	3.6	4.9	-0.06
Heart failure	0.3	0.3	-0.01	0.3	0.5	-0.03
Ischemic heart disease	1	1	0	1.1	1.5	-0.04
Peripheral vascular disease	0.2	0.2	0	0.2	0.4	-0.03
Pulmonary embolism	0.2	0.1	0.02	0.2	0.3	-0.03
Venous thrombosis	0.3	0.2	0.03	0.4	0.4	-0.01
**Medical history (neoplasms)**						
Malignant neoplasm of anorectum	0.1	0.1	0.03	0.1	0.2	0
Malignant neoplastic disease	1.1	0.8	0.03	1.2	1.7	-0.04
Malignant tumor of breast	0.3	0.2	0.02	0.3	0.5	-0.02
Malignant tumor of colon	0.2	0.1	0.03	0.2	0.2	0
Primary malignant neoplasm of prostate	0.2	0.1	0.01	0.2	0.3	-0.03
**Medication use**						
Agents acting on the renin-angiotensin system	10.5	10	0.02	11.5	14.6	-0.09
Antibacterials for systemic use	36.9	16.8	0.47	35.5	40.2	-0.1
Antidepressants	7.3	4.7	0.11	7.8	9.6	-0.06
Antiepileptics	2.4	1.9	0.03	2.6	3.6	-0.06
Antiinflammatory and antirheumatic products	35.2	20.1	0.34	36.2	41	-0.1
Antineoplastic agents	1	0.8	0.02	1.1	1.2	-0.01
Antipsoriatics	0.5	0.3	0.03	0.5	0.7	-0.02
Antithrombotic agents	8	7.6	0.01	8.7	11	-0.08

Table S6.10. *Continued.* Selected baseline characteristics for France IQVIA, for the short-term risk of neurodevelopmental disorders

Characteristic	Before propensity score matching			After propensity score matching		
	**Targets, %**	**Comparators, %**	**Standardized difference**	**Targets, %**	**Comparators, %**	**Standardized difference**
**Medication use**						
Beta blocking agents	6.3	6.4	-0.01	6.9	8.5	-0.06
Calcium channel blockers	5.8	5.3	0.02	6.4	8.1	-0.07
Diuretics	5.3	5.5	-0.01	5.9	7.7	-0.07
Drugs for acid-related disorders	24.5	14.3	0.26	25.4	29.5	-0.09
Drugs for obstructive airway diseases	23.1	12.7	0.28	23.4	27.4	-0.09
Drugs used in diabetes	5.5	4.3	0.06	5.9	7.5	-0.06
Immunosuppressants	0.3	0.5	-0.03	0.4	0.4	-0.01
Lipid modifying agents	8.2	8.2	0	9	11.5	-0.08
Opioids	58.5	23.5	0.76	57	58.5	-0.03
Psycholeptics	15	8.7	0.2	15.8	19	-0.09
Psychostimulants, agents used for ADHD and nootropics	4.5	2	0.14	4.4	5.3	-0.04

Table S6.11. Selected baseline characteristics for France IQVIA, for the short-term risk of any of psychiatric and neuropsychiatric disorders

Characteristic	Before propensity score matching			After propensity score matching		
	Targets, %	Comparators, %	Standardized difference	Targets, %	Comparators, %	Standardized difference
**Age group (years)**						
0-4	0.7	3	-0.17	0.9	1.3	-0.05
5-9	1.8	4.5	-0.15	2.1	2.6	-0.03
10-14	3.2	4.5	-0.06	3.7	4.1	-0.02
15-19	5.6	4.3	0.06	6	6.4	-0.02
20-24	7.3	4.2	0.13	7.4	6.8	0.02
25-29	7.3	4.3	0.13	7.3	7	0.01
30-34	8.3	5	0.13	8.3	7.9	0.01
35-39	9	5.6	0.13	8.7	7.9	0.03
40-44	9.4	5.9	0.13	9	8.2	0.03
45-49	10.1	6.9	0.12	9.8	8.9	0.03
50-54	9.8	7.4	0.09	9.4	9.1	0.01
55-59	8.9	7.9	0.04	8.6	8.7	0
60-64	6.2	7.8	-0.06	6.2	6.3	0
65-69	4	7.7	-0.16	4.1	4.4	-0.01
70-74	3.7	7.9	-0.18	3.8	4.3	-0.03
75-79	2	5.1	-0.17	1.9	2.5	-0.04
80-84	1.3	4	-0.16	1.3	1.6	-0.02
85-89	0.9	2.6	-0.13	0.9	1.2	-0.03
90-94	0.4	1.1	-0.08	0.4	0.6	-0.03
95-99	0.1	0.3	-0.03	0.1	0.2	-0.01
**Sex**						
Female	56.4	57.5	-0.02	55	54	0.02

Table S6.11. *Continued.* Selected baseline characteristics for France IQVIA, for the short-term risk of any of psychiatric and neuropsychiatric disorders

**Characteristic**	**Before propensity score matching**			**After propensity score matching**		
	**Targets, %**	**Comparators, %**	**Standardized difference**	**Targets, %**	**Comparators, %**	**Standardized difference**
**Medical history (general)**						
Acute respiratory disease	16.7	6.8	0.31	15	18.1	-0.08
Chronic liver disease	0.1	0.1	0.01	0.1	0.1	-0.01
Chronic obstructive lung disease	0.9	0.8	0.01	0.7	1	-0.04
Crohn’s disease	0.1	0.1	0.01	0.1	0.1	-0.01
Depressive disorder	6.1	3.2	0.14	0.2	0.1	0.04
Diabetes mellitus	5.2	3.9	0.06	5	6.6	-0.07
Gastroesophageal reflux disease	4.3	2	0.13	3.7	4.6	-0.04
Gastrointestinal hemorrhage	0.5	0.3	0.03	0.4	0.5	-0.02
Human immunodeficiency virus infection	0.2	0.1	0.04	0.2	0.4	-0.04
Hyperlipidemia	4	2.9	0.06	3.6	4.3	-0.03
Hypertensive disorder	11.7	9.9	0.06	11.2	14.3	-0.09
Lesion of liver	0.1	0.1	0	0.1	0.1	-0.01
Obesity	0.4	0.1	0.05	0.3	0.3	0
Osteoarthritis	3.8	2.2	0.09	3.3	4.1	-0.04
Pneumonia	0.8	0.3	0.07	0.7	1	-0.03
Psoriasis	1	0.6	0.05	0.9	1.1	-0.02
Renal impairment	0.3	0.2	0.03	0.3	0.4	-0.02
Rheumatoid arthritis	0.2	0.3	-0.01	0.2	0.3	-0.02
Ulcerative colitis	0.1	0.1	0.01	0.1	0.1	-0.01
Urinary tract infectious disease	1.5	0.7	0.08	1.4	1.6	-0.02

Table S6.11. *Continued.* Selected baseline characteristics for France IQVIA, for the short-term risk of any of psychiatric and neuropsychiatric disorders

6	Before propensity score matching			After propensity score matching		
	**Targets, %**	**Comparators, %**	**Standardized diff**	**Targets, %**	**Comparators, %**	**Standardized diff**
**Medical history (cardiovascular disease)**						
Atrial fibrillation	0.1	0.3	-0.03	0.1	0.2	-0.01
Cerebrovascular disease	0.9	0.8	0.02	0.8	1.2	-0.04
Coronary arteriosclerosis	0.4	0.4	0	0.4	0.5	-0.02
Heart disease	3.3	3.6	-0.02	3.1	4.3	-0.06
Heart failure	0.3	0.3	-0.01	0.2	0.4	-0.02
Peripheral vascular disease	0.2	0.2	0	0.2	0.3	-0.03
Pulmonary embolism	0.2	0.1	0.02	0.2	0.3	-0.02
Venous thrombosis	0.3	0.2	0.03	0.3	0.4	-0.01
**Medical history (neoplasms)**						
Malignant neoplasm of anorectum	0.1	0.1	0.02	0.1	0.1	0
Malignant neoplastic disease	1.1	0.8	0.03	1	1.4	-0.04
Malignant tumor of breast	0.3	0.2	0.02	0.2	0.4	-0.02
Malignant tumor of colon	0.2	0.1	0.03	0.1	0.1	0
Primary malignant neoplasm of prostate	0.2	0.1	0.01	0.1	0.3	-0.03
**Medication use**						
Agents acting on the renin-angiotensin system	10.5	10	0.02	10.4	13.1	-0.09
Antibacterials for systemic use	36.9	16.7	0.47	33.4	38.3	-0.1
Antidepressants	7.3	4.7	0.11	2	2.3	-0.02
Antiepileptics	2.4	2	0.03	1.9	2.8	-0.06
Antiinflammatory and antirheumatic products	35.2	20.2	0.34	33.7	38.5	-0.1
Antineoplastic agents	1	0.8	0.02	0.9	1	-0.01
Antipsoriatics	0.5	0.3	0.03	0.5	0.6	-0.01
Antithrombotic agents	8	7.6	0.01	7.5	9.7	-0.08
Beta blocking agents	6.3	6.3	0	5.8	7.3	-0.06
Calcium channel blockers	5.8	5.4	0.02	5.6	7.2	-0.06

Table S6.11. *Continued.* Selected baseline characteristics for France IQVIA, for the short-term risk of any of psychiatric and neuropsychiatric disorders

**Characteristic**	**Before propensity score matching**			**After propensity score matching**		
	**Targets, %**	**Comparators, %**	**Standardized difference**	**Targets, %**	**Comparators, %**	**Standardized difference**
**Medication use**						
Diuretics	5.3	5.5	-0.01	5.1	6.9	-0.07
Drugs for acid-related disorders	24.5	14.3	0.26	22	25.6	-0.09
Drugs for obstructive airway diseases	23.1	12.7	0.28	21.6	25.4	-0.09
Drugs used in diabetes	5.5	4.3	0.05	5.3	6.9	-0.07
Immunosuppressants	0.3	0.5	-0.03	0.3	0.4	-0.02
Lipid modifying agents	8.2	8.2	0	7.9	10.1	-0.08
Opioids	58.5	23.3	0.77	54.7	56.6	-0.04
Psycholeptics	15	8.5	0.2	6.4	7	-0.02
Psychostimulants, agents used for ADHD and nootropics	4.5	2	0.14	3.8	4.4	-0.03

Table S6.12. Selected baseline characteristics for France IQVIA, for the medium-term risk of depression

Characteristic	Before propensity score matching			After propensity score matching		
	Targets, %	Comparators, %	Standardized difference	Targets, %	Comparators, %	Standardized difference
**Age group (years)**						
0-4	0.7	3	-0.17	0.8	1.2	-0.04
5-9	1.8	4.4	-0.15	2	2.3	-0.02
10-14	3.2	4.3	-0.06	3.5	3.8	-0.01
15-19	5.6	4.3	0.06	5.6	5.9	-0.01
20-24	7.3	4.3	0.13	6.8	6.1	0.03
25-29	7.3	4.4	0.13	6.5	6.3	0.01
30-34	8.3	5	0.13	7.8	7.2	0.02
35-39	9	5.6	0.13	8.6	7.7	0.03
40-44	9.4	5.9	0.13	9.1	8.3	0.03
45-49	10.1	6.8	0.12	9.9	9.2	0.03
50-54	9.8	7.4	0.09	9.7	9.4	0.01
55-59	8.9	7.9	0.04	9	9.2	0
60-64	6.2	7.8	-0.07	6.5	6.9	-0.01
65-69	4	7.7	-0.16	4.6	4.8	-0.01
70-74	3.7	8	-0.18	4.2	4.9	-0.04
75-79	2	5.2	-0.17	2.3	2.8	-0.03
80-84	1.3	4	-0.16	1.5	1.9	-0.03
85-89	0.9	2.6	-0.13	0.9	1.4	-0.04
90-94	0.4	1	-0.08	0.4	0.6	-0.03
95-99	0.1	0.3	-0.03	0.1	0.2	-0.01
**Sex**						
Female	56.4	57.4	-0.02	56.1	55.5	0.01

Table S6.12. *Continued.* Selected baseline characteristics for France IQVIA, for the medium-term risk of depression

Characteristic	Before propensity score matching			After propensity score matching		
	Targets, %	Comparators, %	Standardized difference	Targets, %	Comparators, %	Standardized difference
**Medical history (general)**						
Acute respiratory disease	16.7	6.8	0.31	16.1	18.8	-0.07
Chronic liver disease	0.1	0.1	0.01	0.1	0.1	-0.01
Chronic obstructive lung disease	0.9	0.8	0.01	0.9	1.4	-0.05
Crohn’s disease	0.1	0.1	0.01	0.1	0.2	-0.01
Dementia	0.1	0.1	0	0.1	0.1	0
Diabetes mellitus	5.2	3.9	0.06	5.7	7.4	-0.07
Gastroesophageal reflux disease	4.3	2	0.13	4.3	5.1	-0.04
Gastrointestinal hemorrhage	0.5	0.3	0.04	0.5	0.6	-0.02
Human immunodeficiency virus infection	0.2	0.1	0.04	0.2	0.4	-0.05
Hyperlipidemia	4	3	0.05	4.3	5.1	-0.04
Hypertensive disorder	11.7	10.1	0.05	12.9	16.5	-0.1
Lesion of liver	0.1	0.1	0	0.1	0.1	-0.01
Obesity	0.4	0.1	0.05	0.3	0.3	0
Osteoarthritis	3.8	2.2	0.09	3.7	4.6	-0.05
Pneumonia	0.8	0.3	0.07	0.8	1	-0.02
Psoriasis	1	0.5	0.05	1	1.1	-0.01
Renal impairment	0.3	0.2	0.02	0.3	0.5	-0.02
Rheumatoid arthritis	0.2	0.3	-0.01	0.2	0.4	-0.03
Ulcerative colitis	0.1	0.1	0.01	0.1	0.2	-0.01
Urinary tract infectious disease	1.5	0.7	0.08	1.4	1.8	-0.03

Table S6.12. *Continued.* Selected baseline characteristics for France IQVIA, for the medium-term risk of depression

Characteristic	Before propensity score matching			After propensity score matching		
	Targets, %	Comparators, %	Standardized difference	Targets, %	Comparators, %	Standardized difference
**Medical history (cardiovascular disease)**						
Atrial fibrillation	0.1	0.3	-0.03	0.2	0.2	-0.02
Cerebrovascular disease	0.9	0.8	0.02	1	1.2	-0.02
Coronary arteriosclerosis	0.4	0.4	0.01	0.5	0.6	-0.01
Heart disease	3.3	3.6	-0.02	3.6	4.9	-0.06
Heart failure	0.3	0.3	-0.01	0.3	0.5	-0.04
Ischemic heart disease	1	1	0	1.2	1.5	-0.03
Peripheral vascular disease	0.2	0.2	0	0.2	0.3	-0.02
Pulmonary embolism	0.2	0.1	0.01	0.2	0.3	-0.01
Venous thrombosis	0.3	0.2	0.03	0.3	0.5	-0.02
**Medical history (neoplasms)**						
Malignant neoplasm of anorectum	0.1	0.1	0.03	0.2	0.2	-0.01
Malignant neoplastic disease	1.1	0.8	0.03	1.2	1.7	-0.04
Malignant tumor of breast	0.3	0.2	0.02	0.3	0.5	-0.02
Malignant tumor of colon	0.2	0.1	0.03	0.2	0.2	0
Primary malignant neoplasm of prostate	0.2	0.1	0.01	0.2	0.2	-0.01
**Medication use**						
Agents acting on the renin-angiotensin system	10.5	10.1	0.01	11.9	14.8	-0.09
Antibacterials for systemic use	36.9	16.6	0.47	35.1	39.4	-0.09
Antidepressants	7.3	4.7	0.11	3.4	4.2	-0.04
Antiepileptics	2.4	1.9	0.03	2.2	3.2	-0.06
Antiinflammatory and antirheumatic products	35.2	20.1	0.34	36.5	41.3	-0.1
Antineoplastic agents	1	0.8	0.02	1	1.2	-0.01
Antipsoriatics	0.5	0.3	0.04	0.6	0.7	-0.01
Antithrombotic agents	8	7.7	0.01	8.7	11	-0.08
Beta blocking agents	6.3	6.4	-0.01	6.9	8.9	-0.07

Table S6.12. *Continued.* Selected baseline characteristics for France IQVIA, for the medium-term risk of depression

Characteristic	Before propensity score matching			After propensity score matching		
	**Targets, %**	**Comparators, %**	**Standardized difference**	**Targets, %**	**Comparators, %**	**Standardized difference**
**Medication use**						
Calcium channel blockers	5.8	5.3	0.02	6.6	8.5	-0.07
Diuretics	5.3	5.5	-0.01	5.9	7.8	-0.08
Drugs for acid-related disorders	24.5	14.3	0.26	24.8	29.4	-0.1
Drugs for obstructive airway diseases	23.1	12.6	0.28	23.5	27	-0.08
Drugs used in diabetes	5.5	4.2	0.06	6	7.8	-0.07
Immunosuppressants	0.3	0.5	-0.03	0.4	0.4	-0.01
Lipid modifying agents	8.2	8.2	0	9.1	11.5	-0.08
Opioids	58.5	23.3	0.77	55.8	57.8	-0.04
Psycholeptics	15	8.6	0.2	12.6	15.3	-0.08
Psychostimulants, agents used for ADHD and nootropics	4.5	2.1	0.13	4.5	5.1	-0.03

Table S6.13. Selected baseline characteristics for France IQVIA, for the medium-term risk of anxiety disorders

**Characteristic**	**Before propensity score matching**			**After propensity score matching**		
	**Targets, %**	**Comparators, %**	**Standardized difference**	**Targets, %**	**Comparators, %**	**Standardized difference**
Age group						
0-4	0.7	2.9	-0.17	0.8	1.2	-0.04
5-9	1.8	4.5	-0.15	2	2.3	-0.02
10-14	3.2	4.5	-0.07	3.5	3.8	-0.01
15-19	5.6	4.3	0.06	5.7	5.9	-0.01
20-24	7.3	4.2	0.13	6.7	6.1	0.03
25-29	7.3	4.4	0.13	6.5	6.1	0.01
30-34	8.3	5	0.13	7.6	7	0.02
35-39	9	5.7	0.13	8.4	7.5	0.03
40-44	9.4	5.9	0.13	8.9	8.3	0.02
45-49	10.1	6.9	0.12	9.9	9.1	0.03
50-54	9.8	7.5	0.08	9.8	9.6	0.01
55-59	8.9	7.8	0.04	9.2	9.3	0
60-64	6.2	7.8	-0.06	6.6	7	-0.02
65-69	4	7.7	-0.16	4.6	4.9	-0.01
70-74	3.7	7.9	-0.18	4.4	5	-0.03
75-79	2	5.2	-0.17	2.3	2.9	-0.03
80-84	1.3	4	-0.16	1.5	2	-0.03
85-89	0.9	2.5	-0.13	0.9	1.3	-0.04
90-94	0.4	1	-0.07	0.4	0.6	-0.03
95-99	0.1	0.2	-0.03	0.1	0.2	-0.01
**Sex**						
Female	56.4	57.3	-0.02	55.8	55.3	0.01

Table S6.13. *Continued.* Selected baseline characteristics for France IQVIA, for the medium-term risk of anxiety disorders

**Characteristic**	**Before propensity score matching**			**After propensity score matching**		
	**Targets, %**	**Comparators, %**	**Standardized difference**	**Targets, %**	**Comparators, %**	**Standardized difference**
**Medical history (general)**						
Acute respiratory disease	16.7	6.7	0.31	15.8	18.4	-0.07
Chronic liver disease	0.1	0.1	0.01	0.1	0.1	-0.01
Chronic obstructive lung disease	0.9	0.7	0.02	0.9	1.4	-0.05
Crohn’s disease	0.1	0.1	0.01	0.1	0.2	-0.01
Dementia	0.1	0.1	0	0.1	0.1	-0.01
Depressive disorder	6.1	3.2	0.14	5	6	-0.04
Diabetes mellitus	5.2	3.9	0.06	5.8	7.6	-0.07
Gastroesophageal reflux disease	4.3	2.1	0.13	4.2	4.8	-0.03
Gastrointestinal hemorrhage	0.5	0.3	0.04	0.4	0.6	-0.02
Human immunodeficiency virus infection	0.2	0.1	0.03	0.2	0.4	-0.05
Hyperlipidemia	4	3	0.06	4.3	5.2	-0.04
Hypertensive disorder	11.7	10	0.06	13	16.6	-0.1
Lesion of liver	0.1	0.1	0	0.1	0.1	-0.01
Obesity	0.4	0.1	0.05	0.3	0.3	0
Osteoarthritis	3.8	2.2	0.09	3.7	4.6	-0.04
Pneumonia	0.8	0.3	0.07	0.8	1	-0.02
Psoriasis	1	0.5	0.05	1	1.1	-0.01
Renal impairment	0.3	0.2	0.02	0.3	0.5	-0.03
Rheumatoid arthritis	0.2	0.3	-0.01	0.3	0.4	-0.02
Ulcerative colitis	0.1	0.1	0.01	0.1	0.2	-0.01
Urinary tract infectious disease	1.5	0.7	0.08	1.4	1.8	-0.03

Table S6.13. *Continued.* Selected baseline characteristics for France IQVIA, for the medium-term risk of anxiety disorders

Characteristic	Before propensity score matching			After propensity score matching		
	Targets, %	Comparators, %	Standardized difference	Targets, %	Comparators, %	Standardized difference
**Medical history (cardiovascular disease)**						
Atrial fibrillation	0.1	0.3	-0.03	0.2	0.2	-0.01
Cerebrovascular disease	0.9	0.7	0.02	1.1	1.2	-0.02
Coronary arteriosclerosis	0.4	0.4	0	0.5	0.6	-0.02
Heart disease	3.3	3.6	-0.02	3.6	4.8	-0.06
Heart failure	0.3	0.3	-0.01	0.3	0.5	-0.03
Peripheral vascular disease	0.2	0.2	0	0.2	0.3	-0.02
Pulmonary embolism	0.2	0.1	0.02	0.2	0.3	-0.01
Venous thrombosis	0.3	0.2	0.03	0.4	0.4	-0.01
**Medical history (neoplasms)**						
Malignant neoplasm of anorectum	0.1	0.1	0.02	0.1	0.2	-0.01
Malignant neoplastic disease	1.1	0.8	0.03	1.2	1.6	-0.04
Malignant tumor of breast	0.3	0.2	0.02	0.3	0.4	-0.02
Malignant tumor of colon	0.2	0.1	0.02	0.1	0.2	-0.01
Primary malignant neoplasm of prostate	0.2	0.1	0.01	0.2	0.3	-0.01
**Medication use**						
Agents acting on the renin-angiotensin system	10.5	10	0.02	12	15	-0.09
Antibacterials for systemic use	36.9	16.7	0.47	34.8	39	-0.09
Antidepressants	7.3	4.6	0.12	5.6	6.9	-0.05
Antiepileptics	2.4	1.9	0.03	2.5	3.4	-0.06
Antiinflammatory and antirheumatic products	35.2	20.1	0.34	36.1	40.8	-0.1
Antineoplastic agents	1	0.8	0.03	1.1	1.1	0
Antipsoriatics	0.5	0.3	0.04	0.5	0.7	-0.02
Antithrombotic agents	8	7.6	0.01	8.9	11.1	-0.07
Beta blocking agents	6.3	6.4	0	6.9	8.9	-0.07
Calcium channel blockers	5.8	5.4	0.02	6.6	8.5	-0.07

Table S6.13. *Continued.* Selected baseline characteristics for France IQVIA, for the medium-term risk of anxiety disorders

Characteristic	Before propensity score matching			After propensity score matching		
	**Targets, %**	**Comparators, %**	**Standardized difference**	**Targets, %**	**Comparators, %**	**Standardized difference**
**Medication use**						
Diuretics	5.3	5.5	-0.01	6.1	7.9	-0.07
Drugs for acid-related disorders	24.5	14.2	0.26	24.6	28.9	-0.1
Drugs for obstructive airway diseases	23.1	12.8	0.27	23.2	26.6	-0.08
Drugs used in diabetes	5.5	4.2	0.06	6.1	7.9	-0.07
Immunosuppressants	0.3	0.5	-0.03	0.4	0.4	-0.01
Lipid modifying agents	8.2	8.1	0	9.3	11.8	-0.08
Opioids	58.5	23.3	0.77	55.4	57.5	-0.04
Psycholeptics	15	8.6	0.2	10.8	12.9	-0.06
Psychostimulants, agents used for ADHD and nootropics	4.5	2	0.14	4.4	5	-0.03

Table S6.14. Selected baseline characteristics for France IQVIA, for the medium-term risk of alcohol misuse or dependence

Characteristic	Before propensity score matching			After propensity score matching		
	Targets, %	Comparators, %	Standardized difference	Targets, %	Comparators, %	Standardized difference
**Age group (years)**						
0-4	0.7	2.9	-0.17	0.7	1.1	-0.04
4-9	1.8	4.5	-0.15	1.8	2	-0.02
10-14	3.2	4.4	-0.06	3.2	3.5	-0.01
15-19	5.6	4.3	0.06	5.3	5.5	-0.01
20-24	7.3	4.2	0.13	6.5	5.8	0.03
25-29	7.3	4.3	0.13	6.3	6.1	0.01
30-34	8.3	5	0.13	7.7	7	0.03
35-39	9	5.6	0.13	8.5	7.6	0.04
40-44	9.4	6	0.13	9.2	8.4	0.03
45-49	10.1	6.8	0.12	10.2	9.6	0.02
50-54	9.8	7.5	0.08	10.1	9.8	0.01
55-59	8.9	7.9	0.04	9.4	9.7	-0.01
60-64	6.2	7.8	-0.06	6.7	7.1	-0.01
65-69	4	7.8	-0.16	4.6	4.8	-0.01
70-74	3.7	7.9	-0.18	4.3	5	-0.03
75-79	2	5.2	-0.17	2.4	2.8	-0.03
80-84	1.3	4	-0.16	1.6	2	-0.03
85-89	0.9	2.5	-0.13	0.9	1.4	-0.04
90-94	0.4	1	-0.07	0.4	0.6	-0.03
95-99	0.1	0.2	-0.03	0.1	0.1	-0.01
**Sex**						
Female	56.4	57.5	-0.02	57.5	57.2	0.01

Table S6.14. *Continued.* Selected baseline characteristics for France IQVIA, for the medium-term risk of alcohol misuse or dependence

**Characteristic**	**Before propensity score matching**			**After propensity score matching**		
	**Targets, %**	**Comparators, %**	**Standardized difference**	**Targets, %**	**Comparators, %**	**Standardized difference**
**Medical history (general)**						
Acute respiratory disease	16.7	6.8	0.31	16.6	19.1	-0.07
Chronic liver disease	0.1	0.1	0	0.1	0.1	-0.01
Chronic obstructive lung disease	0.9	0.8	0.01	1	1.5	-0.04
Crohn's disease	0.1	0.1	0.01	0.1	0.2	-0.01
Dementia	0.1	0.1	0	0.1	0.2	-0.01
Depressive disorder	6.1	3.2	0.14	6.7	8	-0.05
Diabetes mellitus	5.2	4	0.06	5.9	7.5	-0.06
Gastroesophageal reflux disease	4.3	2.1	0.13	4.7	5.6	-0.04
Gastrointestinal hemorrhage	0.5	0.3	0.04	0.5	0.6	-0.01
Human immunodeficiency virus infection	0.2	0.1	0.04	0.2	0.4	-0.05
Hyperlipidemia	4	3	0.06	4.5	5.5	-0.05
Hypertensive disorder	11.7	10	0.06	13.4	17	-0.1
Lesion of liver	0.1	0.1	0	0.1	0.1	-0.01
Obesity	0.4	0.1	0.05	0.3	0.4	0
Osteoarthritis	3.8	2.2	0.09	4	5	-0.05
Pneumonia	0.8	0.3	0.07	0.8	1	-0.02
Psoriasis	1	0.5	0.05	1	1.2	-0.01
Renal impairment	0.3	0.2	0.02	0.3	0.5	-0.03
Rheumatoid arthritis	0.2	0.3	-0.01	0.3	0.4	-0.02
Schizophrenia	0.1	0.1	0	0.1	0.1	0
Ulcerative colitis	0.1	0.1	0.01	0.1	0.2	-0.01
Urinary tract infectious disease	1.5	0.7	0.08	1.5	2	-0.04

Table S6.14. *Continued.* Selected baseline characteristics for France IQVIA, for the medium-term risk of alcohol misuse or dependence

Characteristic	Before propensity score matching			After propensity score matching		
	**Targets, %**	**Comparators, %**	**Standardized difference**	**Targets, %**	**Comparators, %**	**Standardized difference**
**Medical history (cardiovascular disease)**						
Atrial fibrillation	0.1	0.3	-0.03	0.2	0.2	-0.01
Cerebrovascular disease	0.9	0.8	0.02	1.1	1.3	-0.01
Coronary arteriosclerosis	0.4	0.4	0	0.5	0.6	-0.01
Heart disease	3.3	3.6	-0.02	3.8	4.9	-0.06
Heart failure	0.3	0.3	-0.01	0.3	0.5	-0.03
Pulmonary embolism	0.2	0.1	0.02	0.2	0.3	-0.01
Venous thrombosis	0.3	0.2	0.03	0.4	0.5	-0.02
**Medical history: Neoplasms**						
Malignant neoplasm of anorectum	0.1	0.1	0.03	0.1	0.2	0
Malignant neoplastic disease	1.1	0.8	0.03	1.3	1.7	-0.04
Malignant tumor of breast	0.3	0.2	0.01	0.4	0.5	-0.03
Malignant tumor of colon	0.2	0.1	0.03	0.2	0.2	0
Primary malignant neoplasm of prostate	0.2	0.1	0.01	0.2	0.3	-0.01
**Medication use**						
Agents acting on the renin-angiotensin system	10.5	10	0.02	12.2	15.1	-0.08
Antibacterials for systemic use	36.9	16.7	0.47	36	40.2	-0.09
Antidepressants	7.3	4.7	0.11	8	9.7	-0.06
Antiepileptics	2.4	1.9	0.03	2.7	3.6	-0.06
Antiinflammatory and antirheumatic products	35.2	20.2	0.34	37.5	42.3	-0.1
Antineoplastic agents	1	0.8	0.02	1.1	1.2	0
Antipsoriatics	0.5	0.3	0.03	0.5	0.7	-0.02
Antithrombotic agents	8	7.7	0.01	9.1	11.4	-0.07
Beta blocking agents	6.3	6.5	-0.01	7.3	9.3	-0.07
Calcium channel blockers	5.8	5.3	0.02	6.8	8.7	-0.07

Table S6.14. *Continued.* Selected baseline characteristics for France IQVIA, for the medium-term risk of alcohol misuse or dependence

**Characteristic**	**Before propensity score matching**			**After propensity score matching**		
	**Targets, %**	**Comparators, %**	**Standardized difference**	**Targets, %**	**Comparators, %**	**Standardized difference**
**Medication use**						
Diuretics	5.3	5.5	-0.01	6.1	8.1	-0.07
Drugs for acid-related disorders	24.5	14.3	0.26	26.3	30.9	-0.1
Drugs for obstructive airway diseases	23.1	12.7	0.27	24.2	27.7	-0.08
Drugs used in diabetes	5.5	4.3	0.05	6.2	7.9	-0.06
Immunosuppressants	0.3	0.5	-0.03	0.4	0.4	-0.01
Opioids	58.5	23.3	0.77	56.8	58.6	-0.04
Psycholeptics	15	8.6	0.2	16.1	19.4	-0.09
Psychostimulants, agents used for ADHD and nootropics	4.5	2.1	0.14	4.8	5.4	-0.03

Table S6.15. Selected baseline characteristics for France IQVIA, for the medium-term risk of substance misuse or dependence

**Characteristic**	**Before propensity score matching**			**After propensity score matching**		
	**Targets, %**	**Comparators, %**	**Standardized difference**	**Targets, %**	**Comparators, %**	**Standardized difference**
**Age group (years)**						
0-4	0.7	2.9	-0.17	0.7	1.1	-0.04
4-9	1.8	4.4	-0.15	1.8	2.1	-0.02
10-14	3.2	4.5	-0.07	3.2	3.5	-0.01
15-19	5.6	4.4	0.05	5.3	5.5	-0.01
20-24	7.3	4.3	0.13	6.5	5.8	0.03
25-29	7.3	4.3	0.13	6.3	6.1	0.01
30-34	8.3	5.1	0.13	7.7	7.1	0.02
35-39	9	5.5	0.13	8.5	7.6	0.04
40-44	9.4	5.9	0.13	9.2	8.4	0.03
45-49	10.1	6.8	0.12	10.1	9.5	0.02
50-54	9.8	7.5	0.08	10.1	9.8	0.01
55-59	8.9	7.9	0.04	9.4	9.6	-0.01
60-64	6.2	7.8	-0.06	6.7	7.1	-0.01
65-69	4	7.6	-0.16	4.6	4.9	-0.01
70-74	3.7	8	-0.19	4.3	5	-0.03
75-79	2	5.2	-0.17	2.4	2.9	-0.03
80-84	1.3	4	-0.16	1.6	2	-0.03
85-89	0.9	2.6	-0.13	0.9	1.4	-0.04
90-94	0.4	1.1	-0.08	0.4	0.6	-0.03
95-99	0.1	0.3	-0.03	0.1	0.1	-0.01
**Sex**						
Female	56.4	57.3	-0.02	57.6	57.1	0.01

Table S6.15. *Continued.* Selected baseline characteristics for France IQVIA, for the medium-term risk of substance misuse or dependence

Characteristic	Before propensity score matching			After propensity score matching		
	**Targets, %**	**Comparators, %**	**Standardized difference**	**Targets, %**	**Comparators, %**	**Standardized difference**
**Medical history (general)**						
Acute respiratory disease	16.7	6.8	0.31	16.6	19.1	-0.07
Chronic liver disease	0.1	0.1	0.01	0.1	0.1	-0.01
Chronic obstructive lung disease	0.9	0.8	0.01	0.9	1.4	-0.05
Crohn's disease	0.1	0.1	0.01	0.1	0.2	-0.01
Dementia	0.1	0.1	0	0.1	0.2	-0.01
Depressive disorder	6.1	3.1	0.14	6.6	7.9	-0.05
Diabetes mellitus	5.2	3.9	0.06	5.9	7.4	-0.06
Gastroesophageal reflux disease	4.3	2.1	0.13	4.6	5.5	-0.04
Gastrointestinal hemorrhage	0.5	0.3	0.04	0.5	0.6	-0.01
Human immunodeficiency virus infection	0.2	0.1	0.03	0.2	0.4	-0.05
Hyperlipidemia	4	3	0.06	4.5	5.4	-0.04
Hypertensive disorder	11.7	10	0.06	13.4	17.1	-0.1
Lesion of liver	0.1	0.1	0	0.1	0.1	-0.01
Obesity	0.4	0.1	0.05	0.3	0.4	0
Osteoarthritis	3.8	2.2	0.09	4	5	-0.05
Pneumonia	0.8	0.3	0.07	0.8	0.9	-0.01
Psoriasis	1	0.5	0.05	1	1.2	-0.01
Renal impairment	0.3	0.2	0.02	0.3	0.5	-0.03
Rheumatoid arthritis	0.2	0.3	-0.01	0.3	0.4	-0.02
Ulcerative colitis	0.1	0.1	0.01	0.1	0.2	-0.01
Urinary tract infectious disease	1.5	0.7	0.08	1.5	2	-0.04

Table S6.15. *Continued.* Selected baseline characteristics for France IQVIA, for the medium-term risk of substance misuse or dependence

Characteristic	Before propensity score matching			After propensity score matching		
	Targets, %	Comparators, %	Standardized difference	Targets, %	Comparators, %	Standardized difference
**Medical history (cardiovascular disease)**						
Atrial fibrillation	0.1	0.3	-0.03	0.2	0.2	-0.02
Cerebrovascular disease	0.9	0.8	0.02	1.1	1.3	-0.02
Coronary arteriosclerosis	0.4	0.4	0.01	0.5	0.6	-0.01
Heart disease	3.3	3.6	-0.02	3.8	5	-0.06
Heart failure	0.3	0.3	0	0.3	0.5	-0.03
Ischemic heart disease	1	1	0	1.2	1.5	-0.03
Peripheral vascular disease	0.2	0.2	0	0.2	0.3	-0.02
Pulmonary embolism	0.2	0.1	0.02	0.2	0.3	-0.01
Venous thrombosis	0.3	0.2	0.03	0.4	0.5	-0.01
**Medical history (neoplasms)**						
Malignant neoplasm of anorectum	0.1	0.1	0.02	0.1	0.2	-0.01
Malignant neoplastic disease	1.1	0.8	0.03	1.3	1.7	-0.04
Malignant tumor of breast	0.3	0.2	0.02	0.4	0.5	-0.03
Malignant tumor of colon	0.2	0.1	0.03	0.2	0.2	0
Primary malignant neoplasm of prostate	0.2	0.1	0.01	0.2	0.3	-0.02
**Medication use**						
Agents acting on the renin-angiotensin system	10.5	10.1	0.01	12.2	15.2	-0.09
Antibacterials for systemic use	36.9	16.7	0.47	36	40.1	-0.08
Antidepressants	7.3	4.7	0.11	7.9	9.6	-0.06
Antiepileptics	2.4	2	0.03	2.6	3.6	-0.06
Antiinflammatory and antirheumatic products	35.2	20.2	0.34	37.5	42	-0.09
Antineoplastic agents	1	0.8	0.02	1.1	1.2	0
Antipsoriatics	0.5	0.3	0.03	0.5	0.7	-0.02
Antithrombotic agents	8	7.7	0.01	9.1	11.4	-0.07
Beta blocking agents	6.3	6.5	-0.01	7.3	9.3	-0.07

Table S6.15. *Continued.* Selected baseline characteristics for France IQVIA, for the medium-term risk of substance misuse or dependence

Characteristic	Before propensity score matching			After propensity score matching		
	**Targets, %**	**Comparators, %**	**Standardized difference**	**Targets, %**	**Comparators, %**	**Standardized difference**
**Medication use**						
Calcium channel blockers	5.8	5.4	0.02	6.8	8.7	-0.07
Diuretics	5.3	5.5	-0.01	6.1	8.1	-0.08
Drugs for acid-related disorders	24.5	14.4	0.26	26.3	30.8	-0.1
Drugs for obstructive airway diseases	23.1	12.6	0.28	24.1	27.6	-0.08
Drugs used in diabetes	5.5	4.3	0.06	6.2	7.8	-0.06
Immunosuppressants	0.3	0.5	-0.03	0.4	0.4	-0.01
Lipid modifying agents	8.2	8.2	0	9.5	11.9	-0.08
Opioids	58.5	23.5	0.76	56.8	58.5	-0.03
Psycholeptics	15	8.7	0.2	16	19	-0.08
Psychostimulants, agents used for ADHD and nootropics	4.5	2	0.14	4.8	5.4	-0.03

Table S6.16. Selected baseline characteristics for France IQVIA, for the medium-term risk of bipolar disorders

**Characteristic**	**Before propensity score matching**			**After propensity score matching**		
	**Targets, %**	**Comparators, %**	**Standardized difference**	**Targets, %**	**Comparators, %**	**Standardized difference**
**Age group (years)**						
0-4	0.7	3	-0.17	0.7	1.1	-0.04
4-9	1.8	4.4	-0.15	1.8	2	-0.02
10-14	3.2	4.3	-0.06	3.2	3.5	-0.01
15-19	5.6	4.4	0.06	5.3	5.5	-0.01
20-24	7.3	4.3	0.13	6.5	5.8	0.03
25-29	7.3	4.3	0.13	6.3	6	0.01
30-34	8.3	5	0.13	7.6	7.1	0.02
35-39	9	5.6	0.13	8.6	7.5	0.04
40-44	9.4	5.9	0.13	9.2	8.4	0.03
45-49	10.1	6.9	0.12	10.2	9.5	0.02
50-54	9.8	7.6	0.08	10.1	9.8	0.01
55-59	8.9	7.8	0.04	9.4	9.7	-0.01
60-64	6.2	7.8	-0.06	6.8	7.1	-0.01
65-69	4	7.7	-0.16	4.6	4.9	-0.01
70-74	3.7	8	-0.19	4.3	5	-0.03
75-79	2	5.3	-0.17	2.4	2.8	-0.03
80-84	1.3	4	-0.17	1.6	2	-0.04
85-89	0.9	2.5	-0.13	0.9	1.4	-0.04
90-94	0.4	1.1	-0.08	0.4	0.6	-0.03
95-99	0.1	0.2	-0.03	0.1	0.1	-0.01
**Sex**						
Female	56.4	57.4	-0.02	57.4	56.9	0.01

Table S6.16. *Continued.* Selected baseline characteristics for France IQVIA, for the medium-term risk of bipolar disorders

**Characteristic**	**Before propensity score matching**			**After propensity score matching**		
	**Targets, %**	**Comparators, %**	**Standardized difference**	**Targets, %**	**Comparators, %**	**Standardized difference**
**Medical history (general)**						
Acute respiratory disease	16.7	6.8	0.31	16.6	19.1	-0.06
Chronic liver disease	0.1	0.1	0.01	0.1	0.1	-0.01
Chronic obstructive lung disease	0.9	0.7	0.02	1	1.5	-0.05
Crohn’s disease	0.1	0.1	0.01	0.1	0.2	-0.01
Dementia	0.1	0.1	0	0.1	0.2	-0.01
Depressive disorder	6.1	3.2	0.14	6.7	8	-0.05
Diabetes mellitus	5.2	4	0.06	5.9	7.6	-0.07
Gastroesophageal reflux disease	4.3	2.1	0.12	4.7	5.5	-0.04
Gastrointestinal hemorrhage	0.5	0.3	0.04	0.5	0.6	-0.01
Human immunodeficiency virus infection	0.2	0.1	0.04	0.2	0.5	-0.05
Hyperlipidemia	4	2.9	0.06	4.5	5.5	-0.04
Hypertensive disorder	11.7	10	0.06	13.5	17.3	-0.1
Lesion of liver	0.1	0.1	0	0.1	0.1	-0.01
Obesity	0.4	0.1	0.05	0.3	0.4	0
Osteoarthritis	3.8	2.2	0.09	4	5	-0.05
Pneumonia	0.8	0.3	0.07	0.8	1	-0.02
Psoriasis	1	0.6	0.04	1	1.2	-0.01
Renal impairment	0.3	0.2	0.02	0.3	0.5	-0.03
Rheumatoid arthritis	0.2	0.3	-0.01	0.3	0.4	-0.02
Schizophrenia	0.1	0.1	0	0.1	0.1	0
Ulcerative colitis	0.1	0.1	0.01	0.1	0.2	-0.01
Urinary tract infectious disease	1.5	0.7	0.08	1.5	2	-0.03

Table S6.16. *Continued.* Selected baseline characteristics for France IQVIA, for the medium-term risk of bipolar disorders

Characteristic	Before propensity score matching			After propensity score matching		
	**Targets, %**	**Comparators, %**	**Standardized difference**	**Targets, %**	**Comparators, %**	**Standardized difference**
**Medical history (cardiovascular disease)**						
Atrial fibrillation	0.1	0.3	-0.03	0.2	0.2	-0.02
Cerebrovascular disease	0.9	0.8	0.02	1.1	1.3	-0.02
Coronary arteriosclerosis	0.4	0.4	0.01	0.5	0.6	-0.02
Heart disease	3.3	3.6	-0.02	3.8	5	-0.06
Heart failure	0.3	0.3	-0.01	0.3	0.5	-0.03
Ischemic heart disease	1	1	0	1.2	1.5	-0.03
Peripheral vascular disease	0.2	0.2	0	0.2	0.3	-0.02
Pulmonary embolism	0.2	0.2	0.01	0.2	0.3	-0.01
Venous thrombosis	0.3	0.2	0.03	0.4	0.5	-0.01
**Medical history (neoplasms)**						
Malignant neoplasm of anorectum	0.1	0.1	0.02	0.2	0.2	-0.01
Malignant neoplastic disease	1.1	0.9	0.03	1.3	1.7	-0.04
Malignant tumor of breast	0.3	0.2	0.02	0.4	0.5	-0.03
Malignant tumor of colon	0.2	0.1	0.02	0.2	0.2	0
Primary malignant neoplasm of prostate	0.2	0.1	0.01	0.2	0.3	-0.01
**Medication use**						
Agents acting on the renin-angiotensin system	10.5	10	0.02	12.3	15.3	-0.09
Antibacterials for systemic use	36.9	16.7	0.47	36	40.3	-0.09
Antidepressants	7.3	4.7	0.11	8.1	9.7	-0.06
Antiepileptics	2.4	1.9	0.03	2.6	3.6	-0.06
Antiinflammatory and antirheumatic products	35.2	20.2	0.34	37.6	42.4	-0.1
Antineoplastic agents	1	0.8	0.02	1.1	1.2	0
Antipsoriatics	0.5	0.3	0.03	0.6	0.7	-0.02
Antithrombotic agents	8	7.6	0.01	9.1	11.4	-0.08

Table S6.16. *Continued.* Selected baseline characteristics for France IQVIA, for the medium-term risk of bipolar disorders

Characteristic	Before propensity score matching			After propensity score matching		
	**Targets, %**	**Comparators, %**	**Standardized difference**	**Targets, %**	**Comparators, %**	**Standardized difference**
**Medication use**						
Beta blocking agents	6.3	6.5	-0.01	7.3	9.4	-0.07
Calcium channel blockers	5.8	5.4	0.02	6.8	8.8	-0.07
Diuretics	5.3	5.5	-0.01	6.2	8.1	-0.08
Drugs for acid-related disorders	24.5	14.3	0.26	26.4	31	-0.1
Drugs for obstructive airway diseases	23.1	12.7	0.28	24.3	27.7	-0.08
Drugs used in diabetes	5.5	4.4	0.05	6.2	7.9	-0.07
Immunosuppressants	0.3	0.6	-0.04	0.4	0.4	0
Opioids	58.5	23.5	0.76	56.9	58.7	-0.04
Psycholeptics	15	8.7	0.2	16.2	19.4	-0.09
Psychostimulants, agents used for ADHD and nootropics	4.5	2	0.14	4.8	5.4	-0.03

Table S6.17. Selected baseline characteristics for France IQVIA, for the medium-term risk of psychoses

Characteristic	Before propensity score matching			After propensity score matching		
	Targets, %	Comparators, %	Standardized difference	Targets, %	Comparators, %	Standardized difference
**Age group (years)**						
0-4	0.7	3	-0.17	0.7	1.1	-0.04
5-9	1.8	4.5	-0.15	1.8	2	-0.02
10-14	3.2	4.4	-0.06	3.2	3.5	-0.01
15-19	5.6	4.4	0.06	5.3	5.5	-0.01
20-24	7.3	4.2	0.13	6.5	5.9	0.03
25-29	7.3	4.3	0.13	6.3	6.1	0.01
30-34	8.3	5.1	0.13	7.7	7	0.03
35-39	9	5.6	0.13	8.5	7.6	0.04
40-44	9.4	5.8	0.14	9.2	8.5	0.02
45-49	10.1	6.9	0.12	10.2	9.5	0.02
50-54	9.8	7.5	0.08	10.1	9.9	0.01
55-59	8.9	7.8	0.04	9.4	9.6	0
60-64	6.2	7.7	-0.06	6.8	7	-0.01
65-69	4	7.7	-0.16	4.6	4.8	-0.01
70-74	3.7	8	-0.19	4.3	5	-0.03
75-79	2	5.3	-0.18	2.4	2.8	-0.03
80-84	1.3	4	-0.16	1.5	2	-0.04
85-89	0.9	2.6	-0.13	0.9	1.4	-0.04
90-94	0.4	1.1	-0.08	0.4	0.6	-0.03
95-99	0.1	0.2	-0.03	0.1	0.2	-0.01
**Sex**						
Female	56.4	57.5	-0.02	57.5	57	0.01

Table S6.17. *Continued.* Selected baseline characteristics for France IQVIA, for the medium-term risk of psychoses

Characteristic	Before propensity score matching			After propensity score matching		
	Targets, %	Comparators, %	Standardized difference	Targets, %	Comparators, %	Standardized difference
**Medical history (general)**						
Acute respiratory disease	16.7	6.8	0.31	16.6	19.1	-0.07
Chronic liver disease	0.1	0.1	0	0.1	0.1	-0.01
Chronic obstructive lung disease	0.9	0.7	0.02	1	1.5	-0.04
Crohn’s disease	0.1	0.1	0.01	0.1	0.2	-0.01
Dementia	0.1	0.1	0	0.1	0.2	-0.01
Depressive disorder	6.1	3.2	0.14	6.7	8.1	-0.05
Diabetes mellitus	5.2	3.9	0.06	5.9	7.5	-0.07
Gastroesophageal reflux disease	4.3	2	0.13	4.7	5.6	-0.04
Gastrointestinal hemorrhage	0.5	0.3	0.04	0.5	0.6	-0.01
Human immunodeficiency virus infection	0.2	0.1	0.03	0.2	0.4	-0.05
Hyperlipidemia	4	3	0.06	4.5	5.5	-0.04
Hypertensive disorder	11.7	9.9	0.06	13.5	17.1	-0.1
Lesion of liver	0.1	0.1	0	0.1	0.1	-0.01
Obesity	0.4	0.1	0.05	0.3	0.4	0
Osteoarthritis	3.8	2.2	0.1	4	4.9	-0.04
Pneumonia	0.8	0.3	0.07	0.8	1	-0.02
Psoriasis	1	0.5	0.05	1	1.2	-0.01
Renal impairment	0.3	0.2	0.02	0.3	0.5	-0.03
Rheumatoid arthritis	0.2	0.3	-0.01	0.3	0.4	-0.02
Ulcerative colitis	0.1	0.1	0.01	0.1	0.2	-0.01
Urinary tract infectious disease	1.5	0.7	0.07	1.5	2	-0.03

Table S6.17. *Continued.* Selected baseline characteristics for France IQVIA, for the medium-term risk of psychoses

**Characteristic**	**Before propensity score matching**			**After propensity score matching**		
	**Targets, %**	**Comparators, %**	**Standardized difference**	**Targets, %**	**Comparators, %**	**Standardized difference**
**Medical history (cardiovascular disease)**						
Atrial fibrillation	0.1	0.2	-0.02	0.2	0.2	-0.02
Cerebrovascular disease	0.9	0.8	0.02	1.1	1.3	-0.02
Coronary arteriosclerosis	0.4	0.4	0	0.5	0.6	-0.02
Heart disease	3.3	3.6	-0.02	3.8	5	-0.06
Heart failure	0.3	0.3	-0.01	0.3	0.5	-0.03
Peripheral vascular disease	0.2	0.2	0	0.2	0.3	-0.02
Pulmonary embolism	0.2	0.1	0.02	0.2	0.3	-0.01
Venous thrombosis	0.3	0.2	0.03	0.4	0.4	-0.01
**Medical history (neoplasms)**						
Malignant neoplasm of anorectum	0.1	0.1	0.02	0.2	0.2	-0.01
Malignant neoplastic disease	1.1	0.9	0.03	1.3	1.8	-0.04
Malignant tumor of breast	0.3	0.2	0.01	0.4	0.5	-0.03
Malignant tumor of colon	0.2	0.1	0.02	0.2	0.2	-0.01
Primary malignant neoplasm of prostate	0.2	0.1	0.01	0.2	0.3	-0.01
**Medication use**						
Agents acting on the renin-angiotensin system	10.5	9.9	0.02	12.3	15.3	-0.09
Antibacterials for systemic use	36.9	16.7	0.47	36	40.2	-0.09
Antidepressants	7.3	4.7	0.11	8.1	9.8	-0.06
Antiepileptics	2.4	1.9	0.03	2.7	3.7	-0.06
Antiinflammatory and antirheumatic products	35.2	20	0.35	37.6	42.3	-0.1
Antineoplastic agents	1	0.8	0.03	1.1	1.2	0
Antipsoriatics	0.5	0.3	0.04	0.6	0.7	-0.02
Antithrombotic agents	8	7.6	0.01	9.1	11.3	-0.07
Calcium channel blockers	5.8	5.2	0.03	6.8	8.8	-0.07
Diuretics	5.3	5.4	0	6.2	8.1	-0.07

Table S6.17. *Continued.* Selected baseline characteristics for France IQVIA, for the medium-term risk of psychoses

Characteristic	Before propensity score matching			After propensity score matching		
	**Targets, %**	**Comparators, %**	**Standardized difference**	**Targets, %**	**Comparators, %**	**Standardized difference**
**Medication use**						
Drugs for acid-related disorders	24.5	14.3	0.26	26.4	31	-0.1
Drugs for obstructive airway diseases	23.1	12.7	0.28	24.2	27.6	-0.08
Drugs used in diabetes	5.5	4.2	0.06	6.2	7.9	-0.07
Immunosuppressants	0.3	0.5	-0.03	0.4	0.4	-0.01
Lipid modifying agents	8.2	8.1	0	9.5	12	-0.08
Opioids	58.5	23.2	0.77	56.9	58.8	-0.04
Psycholeptics	15	8.5	0.2	16.2	19.3	-0.08
Psychostimulants, agents used for ADHD and nootropics	4.5	2	0.14	4.8	5.5	-0.03

Table S6.18. Selected baseline characteristics for France IQVIA, for the medium-term risk of personality disorders

Characteristic	Before propensity score matching			After propensity score matching		
	Targets, %	Comparators, %	Standardized difference	Targets,%	Comparators,%	Standardized difference
**Age group (years)**						
0-4	0.7	2.9	-0.17	0.7	1	-0.04
5-9	1.8	4.4	-0.15	1.8	2	-0.02
10-14	3.2	4.4	-0.06	3.2	3.5	-0.01
15-19	5.6	4.4	0.06	5.2	5.5	-0.01
20-24	7.3	4.2	0.13	6.5	5.8	0.03
25-29	7.3	4.3	0.13	6.3	6	0.01
30-34	8.3	5	0.13	7.7	7	0.02
35-39	9	5.6	0.13	8.5	7.6	0.04
40-44	9.4	5.9	0.13	9.2	8.5	0.03
45-49	10.1	6.9	0.12	10.2	9.6	0.02
50-54	9.8	7.4	0.08	10.2	9.9	0.01
55-59	8.9	7.9	0.04	9.4	9.7	-0.01
60-64	6.2	7.9	-0.07	6.8	7.1	-0.01
65-69	4	7.7	-0.16	4.6	4.8	-0.01
70-74	3.7	8.1	-0.19	4.3	5	-0.03
75-79	2	5.2	-0.17	2.4	2.8	-0.03
80-84	1.3	4	-0.16	1.6	2	-0.03
85-89	0.9	2.5	-0.13	0.9	1.4	-0.04
90-94	0.4	1	-0.08	0.4	0.6	-0.03
95-99	0.1	0.2	-0.03	0.1	0.2	-0.01
**Sex**						
Female	56.4	57.3	-0.02	57.5	56.9	0.01

Table S6.18. *Continued.* Selected baseline characteristics for France IQVIA, for the medium-term risk of personality disorders

Characteristic	Before propensity score matching			After propensity score matching		
	Targets, %	Comparators, %	Standardized difference	Targets, %	Comparators, %	Standardized difference
**Medical history (general)**						
Acute respiratory disease	16.7	6.9	0.3	16.6	19.1	-0.06
Chronic liver disease	0.1	0.1	0.01	0.1	0.1	-0.01
Chronic obstructive lung disease	0.9	0.7	0.02	1	1.5	-0.04
Crohn’s disease	0.1	0.1	0.01	0.1	0.2	-0.01
Dementia	0.1	0.1	0	0.1	0.2	-0.01
Depressive disorder	6.1	3.2	0.14	6.7	8	-0.05
Diabetes mellitus	5.2	3.9	0.06	5.9	7.5	-0.06
Gastroesophageal reflux disease	4.3	2.1	0.13	4.7	5.5	-0.04
Gastrointestinal hemorrhage	0.5	0.3	0.04	0.5	0.6	-0.01
Human immunodeficiency virus infection	0.2	0.1	0.03	0.2	0.4	-0.05
Hyperlipidemia	4	3	0.05	4.5	5.5	-0.04
Hypertensive disorder	11.7	10	0.06	13.5	17.2	-0.1
Lesion of liver	0.1	0.1	0	0.1	0.1	-0.01
Osteoarthritis	3.8	2.2	0.09	4	5	-0.05
Pneumonia	0.8	0.3	0.07	0.8	1	-0.02
Psoriasis	1	0.6	0.05	1	1.2	-0.01
Renal impairment	0.3	0.2	0.02	0.3	0.5	-0.03
Rheumatoid arthritis	0.2	0.3	-0.01	0.3	0.4	-0.02
Schizophrenia	0.1	0.1	0	0.1	0.1	0
Ulcerative colitis	0.1	0.1	0.01	0.1	0.2	-0.01
Urinary tract infectious disease	1.5	0.7	0.08	1.5	2	-0.03

Table S6.18. *Continued.* Selected baseline characteristics for France IQVIA, for the medium-term risk of personality disorders

Characteristic	Before propensity score matching			After propensity score matching		
	Targets, %	Comparators, %	Standardized difference	Targets, %	Comparators, %	Standardized difference
**Medical history (cardiovascular disease)**						
Atrial fibrillation	0.1	0.3	-0.03	0.2	0.2	-0.02
Cerebrovascular disease	0.9	0.8	0.02	1.1	1.3	-0.01
Coronary arteriosclerosis	0.4	0.4	0	0.5	0.6	-0.02
Heart disease	3.3	3.6	-0.02	3.8	4.9	-0.06
Heart failure	0.3	0.3	-0.01	0.3	0.5	-0.03
Ischemic heart disease	1	1	0	1.2	1.5	-0.03
Pulmonary embolism	0.2	0.1	0.02	0.2	0.3	-0.01
Venous thrombosis	0.3	0.2	0.03	0.4	0.5	-0.02
**Medical history (neoplasms)**						
Malignant neoplasm of anorectum	0.1	0.1	0.03	0.2	0.2	-0.01
Malignant neoplastic disease	1.1	0.8	0.03	1.3	1.7	-0.04
Malignant tumor of breast	0.3	0.2	0.02	0.4	0.5	-0.03
Malignant tumor of colon	0.2	0.1	0.03	0.2	0.2	-0.01
Primary malignant neoplasm of prostate	0.2	0.1	0.01	0.2	0.2	-0.01
**Medication use**						
Agents acting on the renin-angiotensin system	10.5	10	0.02	12.3	15.3	-0.09
Antibacterials for systemic use	36.9	16.8	0.47	36	40.2	-0.09
Antidepressants	7.3	4.6	0.11	8.1	9.8	-0.06
Antiepileptics	2.4	2	0.03	2.7	3.7	-0.06
Antiinflammatory and antirheumatic products	35.2	20.2	0.34	37.6	42.2	-0.1
Antineoplastic agents	1	0.8	0.02	1.1	1.2	-0.01
Antipsoriatics	0.5	0.3	0.03	0.6	0.7	-0.02
Antithrombotic agents	8	7.6	0.01	9.1	11.3	-0.07
Beta blocking agents	6.3	6.4	0	7.3	9.3	-0.07
Calcium channel blockers	5.8	5.3	0.02	6.8	8.7	-0.07

Table S6.18. *Continued.* Selected baseline characteristics for France IQVIA, for the medium-term risk of personality disorders

Characteristic	Before propensity score matching			After propensity score matching		
	**Targets, %**	**Comparators, %**	**Standardized difference**	**Targets, %**	**Comparators, %**	**Standardized difference**
**Medication use**						
Diuretics	5.3	5.5	-0.01	6.2	8.1	-0.08
Drugs for acid-related disorders	24.5	14.3	0.26	26.4	30.9	-0.1
Drugs for obstructive airway diseases	23.1	12.8	0.27	24.2	27.7	-0.08
Drugs used in diabetes	5.5	4.3	0.06	6.2	7.9	-0.07
Immunosuppressants	0.3	0.5	-0.03	0.4	0.4	-0.01
Lipid modifying agents	8.2	8.2	0	9.5	12	-0.08
Opioids	58.5	23.5	0.76	56.9	58.7	-0.04
Psycholeptics	15	8.6	0.2	16.3	19.6	-0.09
Psychostimulants, agents used for ADHD and nootropics	4.5	2	0.14	4.8	5.5	-0.03

Table S6.19. Selected baseline characteristics for France IQVIA, for the medium-term risk of self-harm and suicide

Characteristic	Before propensity score matching			After propensity score matching		
	**Targets, %**	**Comparators, %**	**Standardized difference**	**Targets, %**	**Comparators, %**	**Standardized difference**
**Age group (years)**						
0-4	0.7	2.9	-0.17	0.7	1	-0.04
4-9	1.8	4.4	-0.15	1.8	2.1	-0.02
10-14	3.2	4.4	-0.06	3.2	3.4	-0.01
15-19	5.6	4.4	0.06	5.2	5.5	-0.01
20-24	7.3	4.2	0.13	6.5	5.9	0.02
25-29	7.3	4.3	0.13	6.3	6.1	0.01
30-34	8.3	5	0.13	7.7	7	0.03
35-39	9	5.7	0.12	8.5	7.6	0.04
40-44	9.4	5.9	0.13	9.2	8.4	0.03
45-49	10.1	6.8	0.12	10.2	9.6	0.02
50-54	9.8	7.4	0.08	10.2	9.9	0.01
55-59	8.9	7.8	0.04	9.4	9.6	-0.01
60-64	6.2	7.8	-0.06	6.8	7	-0.01
65-69	4	7.8	-0.16	4.6	4.9	-0.01
70-74	3.7	7.9	-0.18	4.3	4.9	-0.03
75-79	2	5.2	-0.17	2.4	2.9	-0.03
80-84	1.3	4	-0.16	1.6	2	-0.04
85-89	0.9	2.6	-0.14	0.9	1.4	-0.04
90-94	0.4	1.1	-0.08	0.4	0.6	-0.03
95-99	0.1	0.3	-0.03	0.1	0.2	-0.01
**Sex**						
Female	56.4	57.6	-0.02	57.5	57	0.01

Table S6.19. *Continued.* Selected baseline characteristics for France IQVIA, for the medium-term risk of self-harm and suicide

Characteristic	Before propensity score matching			After propensity score matching		
	Targets, %	Comparators, %	Standardized difference	Targets, %	Comparators, %	Standardized difference
**Medical history (general)**						
Acute respiratory disease	16.7	6.8	0.31	16.6	19.2	-0.07
Chronic liver disease	0.1	0.1	0	0.1	0.1	-0.01
Chronic obstructive lung disease	0.9	0.7	0.02	1	1.5	-0.04
Crohn’s disease	0.1	0.1	0.01	0.1	0.2	-0.01
Dementia	0.1	0.1	0	0.1	0.2	-0.01
Depressive disorder	6.1	3.2	0.14	6.7	8	-0.05
Diabetes mellitus	5.2	4	0.06	5.9	7.5	-0.06
Gastroesophageal reflux disease	4.3	2.1	0.13	4.7	5.5	-0.04
Gastrointestinal hemorrhage	0.5	0.3	0.04	0.5	0.6	-0.01
Human immunodeficiency virus infection	0.2	0.1	0.03	0.2	0.4	-0.04
Hyperlipidemia	4	3	0.06	4.5	5.4	-0.04
Hypertensive disorder	11.7	9.9	0.06	13.5	17.1	-0.1
Lesion of liver	0.1	0.1	0	0.1	0.1	-0.01
Osteoarthritis	3.8	2.2	0.09	4	5	-0.04
Pneumonia	0.8	0.3	0.07	0.8	1	-0.02
Psoriasis	1	0.5	0.05	1	1.2	-0.01
Renal impairment	0.3	0.2	0.02	0.3	0.5	-0.03
Rheumatoid arthritis	0.2	0.3	-0.01	0.3	0.4	-0.02
Ulcerative colitis	0.1	0.1	0.01	0.1	0.2	0
Urinary tract infectious disease	1.5	0.7	0.08	1.5	2	-0.03

Table S6.19. *Continued.* Selected baseline characteristics for France IQVIA, for the medium-term risk of self-harm and suicide

**Characteristic**	**Before propensity score matching**			**After propensity score matching**		
	**Targets, %**	**Comparators, %**	**Standardized difference**	**Targets, %**	**Comparators, %**	**Standardized difference**
**Medical history (cardiovascular disease)**						
Atrial fibrillation	0.1	0.3	-0.03	0.2	0.2	-0.02
Cerebrovascular disease	0.9	0.8	0.02	1.1	1.3	-0.01
Coronary arteriosclerosis	0.4	0.4	0	0.5	0.6	-0.02
Heart disease	3.3	3.6	-0.02	3.8	5	-0.06
Heart failure	0.3	0.3	-0.01	0.3	0.5	-0.03
Ischemic heart disease	1	1	0	1.2	1.5	-0.03
Peripheral vascular disease	0.2	0.2	0	0.2	0.3	-0.02
Pulmonary embolism	0.2	0.1	0.02	0.2	0.3	-0.01
Venous thrombosis	0.3	0.2	0.03	0.4	0.5	-0.02
**Medical history (neoplasms)**						
Malignant neoplasm of anorectum	0.1	0.1	0.03	0.2	0.2	0
Malignant neoplastic disease	1.1	0.8	0.03	1.3	1.7	-0.04
Malignant tumor of breast	0.3	0.2	0.02	0.4	0.5	-0.03
Malignant tumor of colon	0.2	0.1	0.03	0.2	0.2	0
Primary malignant neoplasm of prostate	0.2	0.1	0.01	0.2	0.2	-0.01
**Medication use**						
Agents acting on the renin-angiotensin system	10.5	10	0.02	12.3	15.2	-0.09
Antibacterials for systemic use	36.9	16.6	0.47	36	40.2	-0.09
Antidepressants	7.3	4.7	0.11	8.1	9.8	-0.06
Antiepileptics	2.4	1.9	0.03	2.7	3.7	-0.06
Antiinflammatory and antirheumatic products	35.2	20.1	0.34	37.6	42.2	-0.1
Antineoplastic agents	1	0.8	0.02	1.1	1.2	0
Antipsoriatics	0.5	0.3	0.03	0.6	0.7	-0.01
Antithrombotic agents	8	7.6	0.01	9.1	11.4	-0.07
Beta blocking agents	6.3	6.4	0	7.3	9.3	-0.07

Table S6.19. *Continued.* Selected baseline characteristics for France IQVIA, for the medium-term risk of self-harm and suicide

Characteristic	Before propensity score matching			After propensity score matching		
	**Targets, %**	**Comparators, %**	**Standardized difference**	**Targets, %**	**Comparators, %**	**Standardized difference**
**Medication use**						
Calcium channel blockers	5.8	5.3	0.03	6.8	8.7	-0.07
Diuretics	5.3	5.4	-0.01	6.2	8.1	-0.07
Drugs for acid-related disorders	24.5	14.2	0.26	26.4	31	-0.1
Drugs for obstructive airway diseases	23.1	12.7	0.27	24.3	27.6	-0.08
Drugs used in diabetes	5.5	4.3	0.05	6.2	7.9	-0.06
Immunosuppressants	0.3	0.5	-0.03	0.4	0.4	-0.01
Lipid modifying agents	8.2	8.2	0	9.5	12	-0.08
Opioids	58.5	23.3	0.77	56.9	58.7	-0.04
Psycholeptics	15	8.7	0.2	16.3	19.5	-0.08
Psychostimulants, agents used for ADHD and nootropics	4.5	2	0.14	4.8	5.5	-0.03

Table S6.20. Selected baseline characteristics for France IQVIA, for the medium-term risk of sleep disorders

Characteristic	Before propensity score matching			After propensity score matching		
	Targets, %	Comparators, %	Standardized difference	Targets, %	Comparators, %	Standardized difference
**Age group (years)**						
0-4	0.7	3	-0.17	0.8	1.1	-0.04
5-9	1.8	4.5	-0.15	1.9	2.2	-0.02
10-14	3.2	4.4	-0.06	3.4	3.7	-0.02
15-19	5.6	4.3	0.06	5.5	5.7	-0.01
20-24	7.3	4.2	0.14	6.7	6.1	0.02
25-29	7.3	4.3	0.13	6.6	6.2	0.01
30-34	8.3	5.1	0.13	7.9	7.2	0.03
35-39	9	5.6	0.13	8.7	7.8	0.03
40-44	9.4	5.9	0.13	9.3	8.4	0.03
45-49	10.1	6.9	0.12	10.2	9.6	0.02
50-54	9.8	7.4	0.08	10	9.7	0.01
55-59	8.9	7.9	0.04	9.3	9.5	-0.01
60-64	6.2	7.8	-0.06	6.5	6.9	-0.02
65-69	4	7.7	-0.16	4.5	4.7	-0.01
70-74	3.7	8	-0.18	4.1	4.7	-0.03
75-79	2	5.1	-0.17	2.2	2.6	-0.03
80-84	1.3	3.9	-0.16	1.4	1.8	-0.03
85-89	0.9	2.5	-0.13	0.8	1.2	-0.04
90-94	0.4	1.1	-0.08	0.4	0.6	-0.03
95-99	0.1	0.2	-0.03	0.1	0.2	-0.01
**Sex**						
Female	56.4	57.5	-0.02	57.1	56.5	0.01

Table S6.20. *Continued.* Selected baseline characteristics for France IQVIA, for the medium-term risk of sleep disorders

Characteristic	Before propensity score matching			After propensity score matching		
	**Targets, %**	**Comparators, %**	**Standardized difference**	**Targets, %**	**Comparators, %**	**Standardized difference**
**Medical history (general)**						
Acute respiratory disease	16.7	6.8	0.31	16.2	18.8	-0.07
Chronic liver disease	0.1	0.1	0	0.1	0.1	-0.01
Chronic obstructive lung disease	0.9	0.7	0.02	0.9	1.4	-0.04
Crohn's disease	0.1	0.1	0.01	0.1	0.2	-0.01
Dementia	0.1	0.1	0.01	0.1	0.1	-0.01
Depressive disorder	6.1	3.1	0.14	5.8	7	-0.05
Diabetes mellitus	5.2	3.9	0.06	5.6	7.3	-0.07
Gastroesophageal reflux disease	4.3	2	0.13	4.3	5.1	-0.04
Gastrointestinal hemorrhage	0.5	0.3	0.04	0.5	0.6	-0.01
Human immunodeficiency virus infection	0.2	0.1	0.03	0.2	0.4	-0.05
Hyperlipidemia	4	3	0.05	4.2	5.1	-0.04
Hypertensive disorder	11.7	9.9	0.06	12.7	16.2	-0.1
Lesion of liver	0.1	0.1	0	0.1	0.1	-0.01
Obesity	0.4	0.1	0.05	0.3	0.3	-0.01
Osteoarthritis	3.8	2.2	0.09	3.7	4.6	-0.04
Pneumonia	0.8	0.3	0.07	0.7	1	-0.02
Psoriasis	1	0.5	0.05	1	1.1	-0.01
Renal impairment	0.3	0.2	0.03	0.3	0.5	-0.02
Rheumatoid arthritis	0.2	0.3	-0.01	0.2	0.4	-0.02
Ulcerative colitis	0.1	0.1	0.01	0.1	0.2	-0.01
Urinary tract infectious disease	1.5	0.7	0.08	1.5	1.9	-0.03

Table S6.20. *Continued.* Selected baseline characteristics for France IQVIA, for the medium-term risk of sleep disorders

**Characteristic**	**Before propensity score matching**			**After propensity score matching**		
	**Targets, %**	**Comparators, %**	**Standardized difference**	**Targets, %**	**Comparators, %**	**Standardized difference**
**Medical history (cardiovascular disease)**						
Atrial fibrillation	0.1	0.3	-0.03	0.1	0.2	-0.01
Cerebrovascular disease	0.9	0.8	0.02	1	1.2	-0.02
Coronary arteriosclerosis	0.4	0.4	0.01	0.5	0.6	-0.01
Heart disease	3.3	3.6	-0.02	3.5	4.6	-0.06
Heart failure	0.3	0.3	-0.01	0.3	0.4	-0.03
Ischemic heart disease	1	1	0	1.1	1.5	-0.03
Peripheral vascular disease	0.2	0.2	0	0.2	0.3	-0.02
Pulmonary embolism	0.2	0.2	0.01	0.2	0.2	-0.01
Venous thrombosis	0.3	0.2	0.03	0.3	0.5	-0.02
**Medical history (neoplasms)**						
Malignant neoplasm of anorectum	0.1	0.1	0.02	0.1	0.2	-0.01
Malignant neoplastic disease	1.1	0.8	0.03	1.1	1.6	-0.04
Malignant tumor of breast	0.3	0.2	0.02	0.3	0.5	-0.02
Malignant tumor of colon	0.2	0.1	0.02	0.2	0.2	0
Primary malignant neoplasm of prostate	0.2	0.1	0.01	0.2	0.2	-0.01
**Medication use**						
Agents acting on the renin-angiotensin system	10.5	10	0.02	11.6	14.5	-0.08
Antibacterials for systemic use	36.9	16.7	0.47	35.2	39.4	-0.09
Antidepressants	7.3	4.6	0.12	7	8.5	-0.06
Antiepileptics	2.4	1.9	0.03	2.4	3.4	-0.06
Antiinflammatory and antirheumatic products	35.2	20.1	0.34	36.5	41	-0.09
Antineoplastic agents	1	0.9	0.02	1	1.1	-0.01
Antipsoriatics	0.5	0.3	0.03	0.6	0.7	-0.01
Antithrombotic agents	8	7.7	0.01	8.5	10.7	-0.07
Beta blocking agents	6.3	6.4	0	6.9	8.8	-0.07

Table S6.20. *Continued.* Selected baseline characteristics for France IQVIA, for the medium-term risk of sleep disorders

Characteristic	Before propensity score matching			After propensity score matching		
	Targets, %	Comparators, %	Standardized difference	Targets, %	Comparators, %	Standardized difference
**Medication use**						
Calcium channel blockers	5.8	5.4	0.02	6.4	8.3	-0.07
Diuretics	5.3	5.5	-0.01	5.8	7.7	-0.07
Drugs for acid-related disorders	24.5	14.4	0.26	25	29.4	-0.1
Drugs for obstructive airway diseases	23.1	12.7	0.28	23.5	26.8	-0.08
Drugs used in diabetes	5.5	4.3	0.06	6	7.6	-0.07
Immunosuppressants	0.3	0.5	-0.03	0.4s	0.4	-0.01
Lipid modifying agents	8.2	8.3	0	9	11.4	-0.08
Opioids	58.5	23.3	0.77	55.9	57.6	-0.04
Psycholeptics	15	8.5	0.2	12.9	15.2	-0.07
Psychostimulants, agents used for ADHD and nootropics	4.5	2	0.14	4.5	5.1	-0.03

Table S6.21. Selected baseline characteristics for France IQVIA, for the medium-term risk of dementia

Characteristic	Before propensity score matching			After propensity score matching		
	**Targets, %**	**Comparators, %**	**Standardized difference**	**Targets, %**	**Comparators, %**	**Standardized difference**
**Age group (years)**						
0-4	0.7	3	-0.17	0.7	1	-0.04
5-9	1.8	4.4	-0.15	1.8	2	-0.01
10-14	3.2	4.4	-0.06	3.2	3.5	-0.01
15-19	5.6	4.3	0.06	5.3	5.5	-0.01
20-24	7.3	4.3	0.13	6.5	5.9	0.02
25-29	7.3	4.3	0.13	6.3	6.1	0.01
30-34	8.3	5	0.13	7.7	7.1	0.02
35-39	9	5.6	0.13	8.6	7.6	0.04
40-44	9.4	6	0.13	9.2	8.4	0.03
45-49	10.1	6.8	0.12	10.2	9.6	0.02
50-54	9.8	7.5	0.08	10.2	9.9	0.01
55-59	8.9	7.9	0.04	9.4	9.6	-0.01
60-64	6.2	7.8	-0.06	6.8	7	-0.01
65-69	4	7.8	-0.16	4.6	4.9	-0.01
70-74	3.7	8	-0.18	4.3	5	-0.03
75-79	2	5.2	-0.17	2.4	2.8	-0.03
80-84	1.3	4	-0.16	1.5	2	-0.03
85-89	0.9	2.6	-0.13	0.9	1.4	-0.04
90-94	0.4	1.1	-0.08	0.4	0.6	-0.03
95-99	0.1	0.2	-0.03	0.1	0.1	-0.01
**Sex**						
Female	56.4	57.4	-0.02	57.4	57	0.01

Table S6.21. *Continued.* Selected baseline characteristics for France IQVIA, for the medium-term risk of dementia

Characteristic	Before propensity score matching			After propensity score matching		
	Targets, %	Comparators, %	Standardized difference	Targets, %	Comparators, %	Standardized difference
**Medical history (general)**						
Acute respiratory disease	16.7	6.8	0.31	16.6	19.1	-0.06
Chronic liver disease	0.1	0.1	0	0.1	0.1	-0.01
Chronic obstructive lung disease	0.9	0.7	0.02	1	1.5	-0.04
Crohn's disease	0.1	0.1	0.01	0.1	0.2	-0.01
Depressive disorder	6.1	3.2	0.13	6.7	8	-0.05
Diabetes mellitus	5.2	3.9	0.06	5.9	7.5	-0.06
Gastroesophageal reflux disease	4.3	2.1	0.13	4.7	5.5	-0.04
Gastrointestinal hemorrhage	0.5	0.3	0.03	0.5	0.6	-0.01
Human immunodeficiency virus infection	0.2	0.1	0.04	0.2	0.4	-0.04
Hyperlipidemia	4	3	0.06	4.5	5.4	-0.04
Hypertensive disorder	11.7	10	0.06	13.5	17.1	-0.1
Lesion of liver	0.1	0.1	0	0.1	0.1	-0.01
Osteoarthritis	3.8	2.2	0.09	4	5	-0.05
Pneumonia	0.8	0.3	0.07	0.8	0.9	-0.01
Psoriasis	1	0.6	0.04	1	1.2	-0.01
Renal impairment	0.3	0.2	0.02	0.3	0.5	-0.03
Rheumatoid arthritis	0.2	0.3	-0.01	0.3	0.4	-0.02
Ulcerative colitis	0.1	0.1	0	0.1	0.2	-0.01
Urinary tract infectious disease	1.5	0.7	0.08	1.5	2	-0.03

Table S6.21. *Continued.* Selected baseline characteristics for France IQVIA, for the medium-term risk of dementia

Characteristic	Before propensity score matching			After propensity score matching		
	Targets, %	Comparators, %	Standardized difference	Targets, %	Comparators, %	Standardized difference
**Medical history (cardiovascular disease)**						
Atrial fibrillation	0.1	0.3	-0.03	0.2	0.2	-0.01
Cerebrovascular disease	0.9	0.8	0.02	1.1	1.3	-0.02
Coronary arteriosclerosis	0.4	0.4	0	0.5	0.6	-0.01
Heart disease	3.3	3.6	-0.02	3.8	5	-0.06
Heart failure	0.3	0.3	-0.01	0.3	0.5	-0.04
Ischemic heart disease	1	1	0	1.2	1.5	-0.03
Peripheral vascular disease	0.2	0.2	0	0.2	0.3	-0.02
Pulmonary embolism	0.2	0.1	0.01	0.2	0.3	-0.01
Venous thrombosis	0.3	0.2	0.03	0.4	0.5	-0.01
**Medical history (neoplasms)**						
Malignant neoplasm of anorectum	0.1	0.1	0.02	0.2	0.2	-0.01
Malignant neoplastic disease	1.1	0.9	0.03	1.3	1.7	-0.04
Malignant tumor of breast	0.3	0.2	0.01	0.4	0.5	-0.03
Malignant tumor of colon	0.2	0.1	0.03	0.2	0.2	0
Primary malignant neoplasm of prostate	0.2	0.1	0.01	0.2	0.2	-0.01
**Medication use**						
Agents acting on the renin-angiotensin system	10.5	10.1	0.01	12.2	15.2	-0.09
Antibacterials for systemic use	36.9	16.7	0.47	36.1	40.1	-0.08
Antidepressants	7.3	4.7	0.11	8.1	9.7	-0.06
Antiepileptics	2.4	1.9	0.03	2.7	3.7	-0.06
Antiinflammatory and antirheumatic products	35.2	20.2	0.34	37.6	42.2	-0.1
Antineoplastic agents	1	0.8	0.02	1.1	1.2	-0.01
Antipsoriatics	0.5	0.3	0.03	0.6	0.7	-0.02
Antithrombotic agents	8	7.7	0.01	9.1	11.3	-0.07
Beta blocking agents	6.3	6.5	-0.01	7.3	9.3	-0.07

Table S6.21. *Continued.* Selected baseline characteristics for France IQVIA, for the medium-term risk of dementia

**Characteristic**	**Before propensity score matching**			**After propensity score matching**		
	**Targets, %**	**Comparators, %**	**Standardized difference**	**Targets, %**	**Comparators, %**	**Standardized difference**
**Medication use**						
Calcium channel blockers	5.8	5.3	0.02	6.8	8.8	-0.07
Diuretics	5.3	5.5	-0.01	6.2	8.1	-0.07
Drugs for acid-related disorders	24.5	14.3	0.26	26.4	30.9	-0.1
Drugs for obstructive airway diseases	23.1	12.7	0.27	24.3	27.5	-0.07
Drugs used in diabetes	5.5	4.3	0.06	6.2	7.9	-0.06
Immunosuppressants	0.3	0.6	-0.04	0.4	0.4	-0.01
Lipid modifying agents	8.2	8.3	0	9.5	11.9	-0.08
Opioids	58.5	23.4	0.77	56.9	58.7	-0.04
Psycholeptics	15	8.7	0.2	16.3	19.5	-0.09
Psychostimulants, agents used for ADHD and nootropics	4.5	2	0.14	4.8	5.5	-0.03

Table S6.22. Selected baseline characteristics for France IQVIA, for the medium-term risk of neurodevelopmental disorders

Characteristic	Before propensity score matching			After propensity score matching		
	**Targets, %**	**Comparators, %**	**Standardized difference**	**Targets, %**	**Comparators, %**	**Standardized difference**
**Age group (years)**						
0-4	0.7	3	-0.17	0.7	1	-0.04
5-9	1.8	4.5	-0.15	1.8	2.1	-0.02
10-14	3.2	4.4	-0.06	3.1	3.4	-0.01
15-19	5.6	4.3	0.06	5.2	5.5	-0.01
20-24	7.3	4.3	0.13	6.5	5.8	0.03
25-29	7.3	4.4	0.13	6.3	6.1	0.01
30-34	8.3	5.1	0.13	7.7	7.1	0.02
35-39	9	5.6	0.13	8.6	7.6	0.04
40-44	9.4	6	0.13	9.2	8.4	0.03
45-49	10.1	6.8	0.12	10.2	9.6	0.02
50-54	9.8	7.5	0.08	10.2	9.9	0.01
55-59	8.9	7.9	0.04	9.5	9.7	-0.01
60-64	6.2	7.8	-0.06	6.8	7.1	-0.01
65-69	4	7.5	-0.15	4.6	4.9	-0.01
70-74	3.7	7.9	-0.18	4.3	5	-0.03
75-79	2	5.3	-0.17	2.4	2.8	-0.03
80-84	1.3	4	-0.16	1.6	2	-0.04
85-89	0.9	2.6	-0.13	0.9	1.4	-0.04
90-94	0.4	1.1	-0.08	0.4	0.6	-0.03
95-99	0.1	0.2	-0.03	0.1	0.2	-0.01
**Sex**						
Female	56.4	57.5	-0.02	57.5	57.1	0.01

Table S6.22. *Continued.* Selected baseline characteristics for France IQVIA, for the medium-term risk of neurodevelopmental disorders

Characteristic	Before propensity score matching			After propensity score matching		
	**Targets, %**	**Comparators, %**	**Standardized difference**	**Targets, %**	**Comparators, %**	**Standardized difference**
**Medical history (general)**						
Acute respiratory disease	16.7	6.8	0.31	16.6	19.2	-0.07
Chronic liver disease	0.1	0.1	0	0.1	0.1	-0.01
Chronic obstructive lung disease	0.9	0.8	0.02	1	1.5	-0.04
Crohn’s disease	0.1	0.1	0.01	0.1	0.2	-0.01
Dementia	0.1	0.1	0	0.1	0.2	-0.01
Depressive disorder	6.1	3.2	0.14	6.7	8	-0.05
Diabetes mellitus	5.2	3.9	0.06	5.9	7.6	-0.07
Gastroesophageal reflux disease	4.3	2	0.13	4.7	5.5	-0.04
Gastrointestinal hemorrhage	0.5	0.3	0.04	0.5	0.6	-0.01
Human immunodeficiency virus infection	0.2	0.1	0.03	0.2	0.4	-0.05
Hyperlipidemia	4	3	0.06	4.5	5.5	-0.04
Hypertensive disorder	11.7	10	0.06	13.5	17.3	-0.1
Lesion of liver	0.1	0.1	0	0.1	0.1	-0.01
Obesity	0.4	0.1	0.05	0.3	0.4	0
Osteoarthritis	3.8	2.2	0.09	4	5	-0.05
Pneumonia	0.8	0.3	0.07	0.8	1	-0.02
Psoriasis	1	0.5	0.05	1	1.2	-0.01
Renal impairment	0.3	0.2	0.02	0.3	0.5	-0.03
Rheumatoid arthritis	0.2	0.3	-0.01	0.3	0.4	-0.02
Ulcerative colitis	0.1	0.1	0.01	0.1	0.2	0
Urinary tract infectious disease	1.5	0.7	0.08	1.5	2	-0.04

Table S6.22. *Continued.* Selected baseline characteristics for France IQVIA, for the medium-term risk of neurodevelopmental disorders

Characteristic	Before propensity score matching			After propensity score matching		
	Targets, %	Comparators, %	Standardized difference	Targets, %	Comparators, %	Standardized difference
**Medical history (cardiovascular disease)**						
Atrial fibrillation	0.1	0.2	-0.03	0.2	0.2	-0.02
Cerebrovascular disease	0.9	0.8	0.02	1.1	1.3	-0.01
Coronary arteriosclerosis	0.4	0.4	0	0.5	0.6	-0.01
Heart disease	3.3	3.6	-0.02	3.8	5	-0.06
Heart failure	0.3	0.3	0	0.3	0.5	-0.03
Ischemic heart disease	1	1	0	1.2	1.5	-0.03
Peripheral vascular disease	0.2	0.2	0	0.2	0.3	-0.02
Pulmonary embolism	0.2	0.1	0.02	0.2	0.3	-0.01
Venous thrombosis	0.3	0.2	0.03	0.4	0.5	-0.01
**Medical history (neoplasms)**						
Malignant lymphoma	0.1	0.1	0	0.1	0.1	-0.01
Malignant neoplasm of anorectum	0.1	0.1	0.02	0.2	0.2	-0.01
Malignant neoplastic disease	1.1	0.8	0.03	1.3	1.8	-0.04
Malignant tumor of breast	0.3	0.2	0.02	0.4	0.5	-0.03
Malignant tumor of colon	0.2	0.1	0.02	0.2	0.2	-0.01
Primary malignant neoplasm of prostate	0.2	0.1	0.01	0.2	0.3	-0.02
**Medication use**						
Agents acting on the renin-angiotensin system	10.5	10	0.02	12.3	15.3	-0.09
Antibacterials for systemic use	36.9	16.7	0.47	36.1	40.3	-0.09
Antidepressants	7.3	4.7	0.11	8.1	9.8	-0.06
Antiepileptics	2.4	1.9	0.03	2.7	3.7	-0.06
Antiinflammatory and antirheumatic products	35.2	20.1	0.34	37.6	42.3	-0.1
Antineoplastic agents	1	0.8	0.02	1.1	1.2	-0.01
Antipsoriatics	0.5	0.3	0.03	0.6	0.7	-0.02
Antithrombotic agents	8	7.6	0.01	9.2	11.4	-0.07

Table S6.22. *Continued.* Selected baseline characteristics for France IQVIA, for the medium-term risk of neurodevelopmental disorders

**Characteristic**	**Before propensity score matching**			**After propensity score matching**		
	**Targets, %**	**Comparators, %**	**Standardized difference**	**Targets, %**	**Comparators, %**	**Standardized difference**
**Medication use**						
Beta blocking agents	6.3	6.4	0	7.4	9.3	-0.07
Calcium channel blockers	5.8	5.3	0.02	6.8	8.9	-0.07
Diuretics	5.3	5.5	-0.01	6.2	8.1	-0.07
Drugs for acid-related disorders	24.5	14.3	0.26	26.4	31.1	-0.1
Drugs for obstructive airway diseases	23.1	12.6	0.28	24.3	27.7	-0.08
Drugs used in diabetes	5.5	4.3	0.06	6.3	7.9	-0.07
Immunosuppressants	0.3	0.5	-0.03	0.4	0.4	-0.01
Opioids	58.5	23.4	0.76	56.9	58.9	-0.04
Psycholeptics	15	8.6	0.2	16.3	19.5	-0.09
Psychostimulants, agents used for ADHD and nootropics	4.5	2	0.14	4.7	5.4	-0.03

Table S6.23. Selected baseline characteristics for France IQVIA, for the medium-term risk of any of any of psychiatric and neuropsychiatric disorders

Characteristic	Before propensity score matching			After propensity score matching		
	**Targets, %**	**Comparators, %**	**Standardized difference**	**Targets, %**	**Comparators, %**	**Standardized difference**
**Age group (years)**						
0-4	0.7	3	-0.17	0.9	1.4	-0.04
5-9	1.8	4.5	-0.15	2.2	2.6	-0.02
10-14	3.2	4.4	-0.06	3.8	4.2	-0.02
15-19	5.6	4.3	0.06	6.2	6.5	-0.01
20-24	7.3	4.3	0.13	7.2	6.5	0.03
25-29	7.3	4.4	0.13	6.9	6.5	0.01
30-34	8.3	5.1	0.13	8	7.4	0.02
35-39	9	5.6	0.13	8.6	7.7	0.03
40-44	9.4	6	0.13	8.9	8.3	0.02
45-49	10.1	6.8	0.12	9.8	9	0.03
50-54	9.8	7.4	0.08	9.4	9	0.01
55-59	8.9	7.8	0.04	8.8	8.9	0
60-64	6.2	7.8	-0.06	6.3	6.6	-0.01
65-69	4	7.6	-0.16	4.4	4.6	-0.01
70-74	3.7	7.9	-0.18	4	4.7	-0.03
75-79	2	5.2	-0.17	2	2.6	-0.04
80-84	1.3	4	-0.16	1.4	1.7	-0.03
85-89	0.9	2.6	-0.13	0.8	1.2	-0.04
90-94	0.4	1.1	-0.08	0.3	0.5	-0.03
95-99	0.1	0.3	-0.03	0.1	0.2	-0.01
**Sex**						
Female	56.4	57.4	-0.02	54.7	54.3	0.01

Table S6.23. *Continued.* Selected baseline characteristics for France IQVIA, for the medium-term risk of any of any of psychiatric and neuropsychiatric disorders

Characteristic	Before propensity score matching			After propensity score matching		
	**Targets, %**	**Comparators, %**	**Standardized difference**	**Targets, %**	**Comparators, %**	**Standardized difference**
**Medical history (general)**						
Acute respiratory disease	16.7	6.9	0.31	15.1	18.1	-0.08
Chronic liver disease	0.1	0.1	0.01	0.1	0.1	-0.01
Chronic obstructive lung disease	0.9	0.7	0.02	0.7	1.2	-0.05
Crohn’s disease	0.1	0.1	0.01	0.1	0.2	-0.01
Diabetes mellitus	5.2	4	0.06	5.4	7.2	-0.07
Gastroesophageal reflux disease	4.3	2.1	0.13	3.7	4.3	-0.03
Gastrointestinal hemorrhage	0.5	0.3	0.04	0.4	0.5	-0.02
Human immunodeficiency virus infection	0.2	0.1	0.03	0.2	0.4	-0.05
Hyperlipidemia	4	2.9	0.06	3.8	4.6	-0.04
Hypertensive disorder	11.7	9.9	0.06	11.8	15.2	-0.1
Lesion of liver	0.1	0.1	0	0.1	0.1	-0.01
Obesity	0.4	0.1	0.05	0.3	0.3	0
Osteoarthritis	3.8	2.2	0.09	3.3	4.2	-0.05
Pneumonia	0.8	0.3	0.07	0.7	0.9	-0.02
Psoriasis	1	0.5	0.05	0.9	1	-0.01
Renal impairment	0.3	0.2	0.02	0.3	0.4	-0.02
Rheumatoid arthritis	0.2	0.3	-0.01	0.2	0.4	-0.03
Ulcerative colitis	0.1	0.1	0.01	0.1	0.2	0
Urinary tract infectious disease	1.5	0.7	0.08	1.3	1.6	-0.02

Table S6.23. *Continued.* Selected baseline characteristics for France IQVIA, for the medium-term risk of any of any of psychiatric and neuropsychiatric disorders

**Characteristic**	**Before propensity score matching**			**After propensity score matching**		
	**Targets, %**	**Comparators, %**	**Standardized difference**	**Targets, %**	**Comparators, %**	**Standardized difference**
**Medical history (cardiovascular disease)**						
Atrial fibrillation	0.1	0.2	-0.02	0.1	0.2	-0.01
Cerebrovascular disease	0.9	0.8	0.02	0.8	1.1	-0.03
Coronary arteriosclerosis	0.4	0.4	0	0.5	0.5	-0.01
Heart disease	3.3	3.7	-0.02	3.2	4.4	-0.06
Heart failure	0.3	0.3	-0.01	0.2	0.4	-0.04
Ischemic heart disease	1	1	0	1	1.4	-0.03
Peripheral vascular disease	0.2	0.2	0	0.2	0.3	-0.02
Pulmonary embolism	0.2	0.1	0.01	0.2	0.2	-0.01
Venous thrombosis	0.3	0.2	0.03	0.3	0.4	-0.02
**Medical history (neoplasms)**						
Malignant neoplasm of anorectum	0.1	0.1	0.03	0.1	0.2	-0.01
Malignant neoplastic disease	1.1	0.9	0.03	1	1.4	-0.04
Malignant tumor of breast	0.3	0.2	0.01	0.3	0.3	-0.01
Malignant tumor of colon	0.2	0.1	0.03	0.1	0.2	0
Primary malignant neoplasm of prostate	0.2	0.1	0.01	0.2	0.2	-0.01
**Medication use**						
Agents acting on the renin-angiotensin system	10.5	10	0.02	11.1	13.9	-0.09
Antibacterials for systemic use	36.9	16.8	0.47	33.4	38	-0.1
Antidepressants	7.3	4.6	0.11	1.8	2.3	-0.04
Antiepileptics	2.4	1.9	0.03	1.9	2.8	-0.06
Antiinflammatory and antirheumatic products	35.2	20.2	0.34	34.5	39.3	-0.1
Antineoplastic agents	1	0.8	0.02	0.9	1.1	-0.01
Antipsoriatics	0.5	0.3	0.03	0.5	0.6	-0.01

Table S6.23. *Continued.* Selected baseline characteristics for France IQVIA, for the medium-term risk of any of any of psychiatric and neuropsychiatric disorders

Characteristic	Before propensity score matching			After propensity score matching		
	Targets, %	Comparators, %	Standardized difference	Targets, %	Comparators, %	Standardized difference
**Medication use**						
Antithrombotic agents	8	7.6	0.01	7.9	10.2	-0.08
Beta blocking agents	6.3	6.5	-0.01	6.2	8	-0.07
Calcium channel blockers	5.8	5.3	0.02	6	7.8	-0.07
Diuretics	5.3	5.5	-0.01	5.4	7.3	-0.08
Drugs for acid-related disorders	24.5	14.3	0.26	22.3	26.6	-0.1
Drugs for obstructive airway diseases	23.1	12.7	0.27	22.1	25.6	-0.08
Drugs used in diabetes	5.5	4.4	0.05	5.7	7.5	-0.07
Immunosuppressants	0.3	0.5	-0.03	0.3	0.5	-0.02
Lipid modifying agents	8.2	8.2	0	8.4	10.7	-0.08
Opioids	58.5	23.5	0.76	53.9	56.1	-0.04
Psycholeptics	15	8.7	0.2	5.7	6.8	-0.04
Psychostimulants, agents used for ADHD and nootropics	4.5	2	0.14	3.9	4.5	-0.03

Table S6.24. Selected baseline characteristics for France IQVIA, for the long-term risk of depression

Characteristic	Before propensity score matching			After propensity score matching		
	Targets, %	Comparators, %	Standardized difference	Targets, %	Comparators, %	Standardized difference
**Age group (years)**						
0-4	0.7	3	-0.17	0.8	1.3	-0.04
4-9	1.8	4.5	-0.15	2	2.3	-0.02
10-14	3.2	4.3	-0.06	3.5	3.7	-0.01
15-19	5.6	4.3	0.06	5.5	5.8	-0.01
20-24	7.3	4.3	0.13	6.6	6	0.02
25-29	7.3	4.3	0.13	6.3	6.1	0.01
30-34	8.3	5	0.13	7.7	7.2	0.02
35-39	9	5.6	0.13	8.4	7.5	0.03
40-44	9.4	5.9	0.13	9.1	8.3	0.03
45-49	10.1	6.9	0.12	10	9.3	0.03
50-54	9.8	7.5	0.08	9.8	9.4	0.01
55-59	8.9	7.8	0.04	9.2	9.7	-0.02
60-64	6.2	7.8	-0.07	6.7	6.9	-0.01
65-69	4	7.7	-0.16	4.7	5	-0.01
70-74	3.7	8.1	-0.19	4.4	4.9	-0.03
75-79	2	5.2	-0.17	2.4	2.8	-0.02
80-84	1.3	3.9	-0.16	1.5	1.8	-0.03
85-89	0.9	2.6	-0.13	0.9	1.3	-0.03
90-94	0.4	1.1	-0.08	0.3	0.5	-0.02
95-99	0.1	0.3	-0.03	0.1	0.2	-0.01
**Sex**						
Female	56.4	57.5	-0.02	56	55.7	0

Table S6.24. *Continued.* Selected baseline characteristics for France IQVIA, for the long-term risk of depression

Characteristic	Before propensity score matching			After propensity score matching		
	Targets, %	Comparators, %	Standardized difference	Targets, %	Comparators, %	Standardized difference
**Medical history (general)**						
Acute respiratory disease	16.7	6.8	0.31	16.4	19.2	-0.07
Chronic liver disease	0.1	0.1	0	0.1	0.1	-0.01
Chronic obstructive lung disease	0.9	0.8	0.01	0.9	1.3	-0.04
Crohn’s disease	0.1	0.1	0.01	0.1	0.2	-0.01
Dementia	0.1	0.1	0	0.1	0.1	-0.01
Diabetes mellitus	5.2	3.9	0.06	5.8	7.2	-0.06
Gastroesophageal reflux disease	4.3	2.1	0.13	4.3	5.2	-0.04
Gastrointestinal hemorrhage	0.5	0.2	0.04	0.5	0.6	-0.02
Human immunodeficiency virus infection	0.2	0.1	0.03	0.2	0.4	-0.03
Hyperlipidemia	4	3	0.06	4.5	5.2	-0.04
Hypertensive disorder	11.7	9.9	0.06	13.2	16.5	-0.09
Lesion of liver	0.1	0.1	0	0.1	0.1	-0.01
Obesity	0.4	0.1	0.05	0.3	0.3	0.01
Osteoarthritis	3.8	2.2	0.09	3.7	4.7	-0.05
Pneumonia	0.8	0.3	0.07	0.8	1	-0.02
Psoriasis	1	0.5	0.05	1	1.1	-0.01
Renal impairment	0.3	0.2	0.03	0.3	0.5	-0.02
Rheumatoid arthritis	0.2	0.3	-0.01	0.2	0.4	-0.02
Ulcerative colitis	0.1	0.1	0.01	0.1	0.1	0
Urinary tract infectious disease	1.5	0.7	0.08	1.5	1.8	-0.03

Table S6.24. *Continued.* Selected baseline characteristics for France IQVIA, for the long-term risk of depression

**Characteristic**	**Before propensity score matching**			**After propensity score matching**		
	**Targets, %**	**Comparators, %**	**Standardized difference**	**Targets, %**	**Comparators, %**	**Standardized difference**
**Medical history (cardiovascular disease)**						
Atrial fibrillation	0.1	0.2	-0.02	0.2	0.2	-0.01
Cerebrovascular disease	0.9	0.8	0.02	1	1.3	-0.03
Coronary arteriosclerosis	0.4	0.4	0	0.5	0.6	-0.01
Heart disease	3.3	3.6	-0.02	3.7	4.9	-0.06
Heart failure	0.3	0.3	0	0.3	0.5	-0.03
Ischemic heart disease	1	1	0	1.2	1.5	-0.03
Peripheral vascular disease	0.2	0.2	0	0.2	0.3	-0.02
Pulmonary embolism	0.2	0.1	0.01	0.2	0.3	-0.02
Venous thrombosis	0.3	0.2	0.03	0.3	0.5	-0.02
Medical history: Neoplasms						
Malignant neoplasm of anorectum	0.1	0.1	0.03	0.2	0.2	-0.01
Malignant neoplastic disease	1.1	0.9	0.03	1.2	1.7	-0.04
Malignant tumor of breast	0.3	0.2	0.01	0.4	0.4	-0.01
Malignant tumor of colon	0.2	0.1	0.03	0.2	0.2	0
Primary malignant neoplasm of prostate	0.2	0.1	0.01	0.2	0.2	-0.02
Medication use						
Agents acting on the renin-angiotensin system	10.5	10	0.02	12.2	15.2	-0.09
Antibacterials for systemic use	36.9	16.7	0.47	35.6	39.7	-0.08
Antidepressants	7.3	4.7	0.11	3.3	4.1	-0.04
Antiepileptics	2.4	1.9	0.03	2.3	3.1	-0.05
Antiinflammatory and antirheumatic products	35.2	20.1	0.34	37	41.6	-0.1
Antineoplastic agents	1	0.8	0.02	1.1	1.2	-0.02
Antipsoriatics	0.5	0.3	0.03	0.5	0.7	-0.02
Antithrombotic agents	8	7.7	0.01	9	11	-0.07
Beta blocking agents	6.3	6.4	0	7.1	8.8	-0.06

Table S6.24. *Continued.* Selected baseline characteristics for France IQVIA, for the long-term risk of depression

Characteristic	Before propensity score matching			After propensity score matching		
	**Targets, %**	**Comparators, %**	**Standardized difference**	**Targets, %**	**Comparators, %**	**Standardized difference**
**Medication use**						
Calcium channel blockers	5.8	5.3	0.02	6.7	8.6	-0.07
Diuretics	5.3	5.5	-0.01	6.1	7.8	-0.07
Drugs for acid-related disorders	24.5	14.3	0.26	25.2	29.3	-0.09
Drugs for obstructive airway diseases	23.1	12.7	0.28	23.9	27.3	-0.08
Drugs used in diabetes	5.5	4.3	0.06	6.2	7.5	-0.05
Immunosuppressants	0.3	0.5	-0.03	0.4	0.5	-0.02
Lipid modifying agents	8.2	8.2	0	9.4	11.5	-0.07
Opioids	58.5	23.4	0.76	55.9	57.9	-0.04
Psycholeptics	15	8.6	0.2	12.7	15.2	-0.07
Psychostimulants, agents used for ADHD and nootropics	4.5	2.1	0.14	4.5	5.1	-0.03

Table S6.25. Selected baseline characteristics for France IQVIA, for the long-term risk of anxiety disorders

Characteristic	Before propensity score matching			After propensity score matching		
	Targets, %	Comparators, %	Standardized difference	Targets, %	Comparators, %	Standardized difference
**Age group (years)**						
0-4	0.7	2.9	-0.17	0.8	1.3	-0.05
5-9	1.8	4.5	-0.15	2.1	2.3	-0.02
10-14	3.2	4.4	-0.06	3.6	3.8	-0.01
15-19	5.6	4.2	0.06	5.6	5.7	-0.01
20-24	7.3	4.3	0.13	6.5	5.9	0.03
25-29	7.3	4.3	0.13	6.2	5.9	0.01
30-34	8.3	5	0.13	7.4	7	0.02
35-39	9	5.6	0.13	8.2	7.4	0.03
40-44	9.4	5.9	0.13	9	8.3	0.03
45-49	10.1	6.8	0.12	9.9	9.3	0.02
50-54	9.8	7.5	0.08	9.9	9.6	0.01
55-59	8.9	7.8	0.04	9.4	9.8	-0.02
60-64	6.2	7.8	-0.06	6.7	7	-0.01
65-69	4	7.8	-0.16	4.8	5.2	-0.02
70-74	3.7	8	-0.19	4.6	5	-0.02
75-79	2	5.2	-0.17	2.4	2.8	-0.03
80-84	1.3	4	-0.17	1.6	1.8	-0.02
85-89	0.9	2.6	-0.13	1	1.3	-0.03
90-94	0.4	1	-0.08	0.4	0.5	-0.02
95-99	0.1	0.2	-0.03	0.1	0.2	-0.01
**Sex**						
Female	56.4	57.4	-0.02	55.6	55.3	0.01

Table S6.25. *Continued.* Selected baseline characteristics for France IQVIA, for the long-term risk of anxiety disorders

Characteristic	Before propensity score matching			After propensity score matching		
	Targets, %	Comparators, %	Standardized difference	Targets, %	Comparators, %	Standardized difference
**Medical history (general)**						
Acute respiratory disease	16.7	6.8	0.31	16	18.7	-0.07
Chronic liver disease	0.1	0.1	0	0.1	0.1	-0.01
Chronic obstructive lung disease	0.9	0.7	0.02	1	1.4	-0.04
Crohn's disease	0.1	0.1	0.01	0.1	0.2	-0.02
Dementia	0.1	0.1	0.01	0.1	0.1	-0.01
Depressive disorder	6.1	3.2	0.14	5	5.9	-0.04
Diabetes mellitus	5.2	3.9	0.06	6	7.4	-0.06
Gastroesophageal reflux disease	4.3	2.1	0.13	4.2	4.9	-0.03
Gastrointestinal hemorrhage	0.5	0.2	0.04	0.5	0.6	-0.02
Human immunodeficiency virus infection	0.2	0.1	0.04	0.2	0.4	-0.04
Hyperlipidemia	4	3	0.05	4.5	5.3	-0.04
Hypertensive disorder	11.7	9.9	0.06	13.3	16.6	-0.09
Lesion of liver	0.1	0.1	0	0.1	0.1	-0.01
Obesity	0.4	0.1	0.05	0.3	0.3	0.01
Osteoarthritis	3.8	2.2	0.09	3.8	4.7	-0.04
Pneumonia	0.8	0.3	0.07	0.8	1	-0.02
Psoriasis	1	0.6	0.04	1	1.1	-0.01
Renal impairment	0.3	0.2	0.02	0.3	0.5	-0.02
Rheumatoid arthritis	0.2	0.3	-0.01	0.3	0.4	-0.02
Schizophrenia	0.1	0.1	0	0.1	0.1	0
Ulcerative colitis	0.1	0.1	0.01	0.1	0.1	0
Urinary tract infectious disease	1.5	0.7	0.08	1.5	1.8	-0.03

Table S6.25. *Continued.* Selected baseline characteristics for France IQVIA, for the long-term risk of anxiety disorders

Characteristic	Before propensity score matching			After propensity score matching		
	Targets, %	Comparators, %	Standardized difference	Targets, %	Comparators, %	Standardized difference
**Medical history (cardiovascular disease)**						
Atrial fibrillation	0.1	0.3	-0.03	0.2	0.2	-0.01
Cerebrovascular disease	0.9	0.8	0.02	1.1	1.3	-0.03
Coronary arteriosclerosis	0.4	0.4	0.01	0.5	0.6	-0.02
Heart disease	3.3	3.7	-0.02	3.7	4.9	-0.06
Heart failure	0.3	0.3	-0.01	0.3	0.5	-0.03
Peripheral vascular disease	0.2	0.2	0	0.2	0.3	-0.02
Pulmonary embolism	0.2	0.1	0.02	0.2	0.3	-0.02
Venous thrombosis	0.3	0.2	0.03	0.4	0.4	-0.01
**Medical history (neoplasms)**						
Malignant neoplasm of anorectum	0.1	0.1	0.02	0.1	0.2	-0.01
Malignant neoplastic disease	1.1	0.9	0.03	1.2	1.7	-0.03
Malignant tumor of breast	0.3	0.2	0.01	0.4	0.4	-0.01
Malignant tumor of colon	0.2	0.1	0.03	0.1	0.2	-0.01
Primary malignant neoplasm of prostate	0.2	0.1	0.01	0.2	0.3	-0.02
**Medication use**						
Agents acting on the renin-angiotensin system	10.5	10	0.02	12.5	15.3	-0.08
Antibacterials for systemic use	36.9	16.8	0.47	35.2	39.3	-0.08
Antidepressants	7.3	4.7	0.11	5.6	6.8	-0.05
Antiepileptics	2.4	1.9	0.03	2.5	3.3	-0.04
Antiinflammatory and antirheumatic products	35.2	20.2	0.34	36.5	41.2	-0.1
Antineoplastic agents	1	0.8	0.02	1.1	1.2	-0.01
Antipsoriatics	0.5	0.3	0.03	0.5	0.7	-0.02
Antithrombotic agents	8	7.8	0.01	9.2	11.2	-0.06
Beta blocking agents	6.3	6.5	-0.01	7.1	8.7	-0.06

Table S6.25. *Continued.* Selected baseline characteristics for France IQVIA, for the long-term risk of anxiety disorders

Characteristic	Before propensity score matching			After propensity score matching		
	**Targets, %**	**Comparators, %**	**Standardized difference**	**Targets, %**	**Comparators, %**	**Standardized difference**
**Medication use**						
Calcium channel blockers	5.8	5.3	0.02	6.8	8.6	-0.07
Diuretics	5.3	5.5	-0.01	6.3	7.8	-0.06
Drugs for acid-related disorders	24.5	14.4	0.26	25	28.7	-0.08
Drugs for obstructive airway diseases	23.1	12.8	0.27	23.5	27	-0.08
Drugs used in diabetes	5.5	4.2	0.06	6.2	7.6	-0.06
Immunosuppressants	0.3	0.5	-0.03	0.4	0.5	-0.02
Lipid modifying agents	8.2	8.2	0	9.7	11.8	-0.07
Opioids	58.5	23.5	0.76	55.5	57.4	-0.04
Psycholeptics	15	8.6	0.2	10.9	12.7	-0.06
Psychostimulants, agents used for ADHD and nootropics	4.5	2	0.14	4.5	5	-0.02

Table S6.26. Selected baseline characteristics for France IQVIA, for the long-term risk of alcohol misuse or dependence

Characteristic	Before propensity score matching			After propensity score matching		
	Targets, %	Comparators, %	Standardized difference	Targets, %	Comparators, %	Standardized difference
**Age group (years)**						
0-4	0.7	3	-0.17	0.7	1.1	-0.04
5-9	1.8	4.5	-0.15	1.8	2	-0.01
10-14	3.2	4.4	-0.06	3.2	3.4	-0.01
15-19	5.6	4.3	0.06	5.1	5.3	-0.01
20-24	7.3	4.2	0.13	6.2	5.7	0.02
25-29	7.3	4.3	0.13	6.1	5.9	0.01
30-34	8.3	5.1	0.13	7.5	7	0.02
35-39	9	5.6	0.13	8.4	7.5	0.04
40-44	9.4	6	0.13	9.2	8.4	0.03
45-49	10.1	6.9	0.12	10.3	9.7	0.02
50-54	9.8	7.5	0.08	10.2	10	0.01
55-59	8.9	7.9	0.04	9.5	10.1	-0.02
60-64	6.2	7.9	-0.07	6.9	7	0
65-69	4	7.6	-0.16	4.7	5.1	-0.02
70-74	3.7	8	-0.18	4.5	5	-0.03
75-79	2	5.2	-0.17	2.5	2.8	-0.02
80-84	1.3	4	-0.16	1.6	1.9	-0.03
85-89	0.9	2.5	-0.13	1	1.3	-0.03
90-94	0.4	1	-0.08	0.4	0.5	-0.03
95-99	0.1	0.2	-0.03	0.1	0.2	-0.01
**Sex**						
Female	56.4	57.3	-0.02	57.7	57.5	0

Table S6.26. *Continued.* Selected baseline characteristics for France IQVIA, for the long-term risk of alcohol misuse or dependence

Characteristic	Before propensity score matching			After propensity score matching		
	**Targets, %**	**Comparators, %**	**Standardized difference**	**Targets, %**	**Comparators, %**	**Standardized difference**
**Medical history (general)**						
Acute respiratory disease	16.7	6.8	0.31	16.9	19.6	-0.07
Chronic liver disease	0.1	0.1	0.01	0.1	0.1	-0.01
Chronic obstructive lung disease	0.9	0.7	0.02	1.1	1.5	-0.04
Crohn's disease	0.1	0.1	0.01	0.1	0.2	-0.01
Depressive disorder	6.1	3.2	0.14	6.8	8.1	-0.05
Diabetes mellitus	5.2	3.9	0.06	6	7.3	-0.05
Gastroesophageal reflux disease	4.3	2.1	0.13	4.7	5.6	-0.04
Gastrointestinal hemorrhage	0.5	0.2	0.04	0.5	0.6	-0.02
Human immunodeficiency virus infection	0.2	0.1	0.04	0.2	0.4	-0.03
Hyperlipidemia	4	3	0.06	4.7	5.5	-0.04
Hypertensive disorder	11.7	10	0.06	13.8	17.1	-0.09
Lesion of liver	0.1	0.1	0	0.1	0.1	0
Obesity	0.4	0.1	0.05	0.4	0.3	0.01
Osteoarthritis	3.8	2.2	0.09	4.1	5.1	-0.05
Pneumonia	0.8	0.3	0.07	0.8	1	-0.01
Psoriasis	1	0.5	0.05	1.1	1.2	-0.01
Renal impairment	0.3	0.2	0.02	0.3	0.5	-0.02
Rheumatoid arthritis	0.2	0.3	-0.01	0.3	0.4	-0.02
Ulcerative colitis	0.1	0.1	0.01	0.1	0.1	0
Urinary tract infectious disease	1.5	0.7	0.08	1.6	2	-0.04

Table S6.26. *Continued.* Selected baseline characteristics for France IQVIA, for the long-term risk of alcohol misuse or dependence

Characteristic	Before propensity score matching			After propensity score matching		
	Targets, %	Comparators, %	Standardized difference	Targets, %	Comparators, %	Standardized difference
**Medical history (cardiovascular disease)**						
Atrial fibrillation	0.1	0.3	-0.03	0.2	0.2	-0.01
Cerebrovascular disease	0.9	0.8	0.02	1.1	1.4	-0.03
Coronary arteriosclerosis	0.4	0.4	0	0.5	0.6	-0.01
Heart disease	3.3	3.6	-0.02	3.9	5	-0.06
Heart failure	0.3	0.3	0	0.3	0.5	-0.03
Ischemic heart disease	1	1	0	1.2	1.5	-0.03
Peripheral vascular disease	0.2	0.2	0	0.2	0.3	-0.02
Pulmonary embolism	0.2	0.1	0.02	0.2	0.3	-0.02
Venous thrombosis	0.3	0.2	0.03	0.4	0.5	-0.02
**Medical history (neoplasms)**						
Malignant neoplasm of anorectum	0.1	0.1	0.02	0.1	0.2	-0.01
Malignant neoplastic disease	1.1	0.9	0.03	1.3	1.8	-0.04
Malignant tumor of breast	0.3	0.2	0.02	0.4	0.5	-0.02
Malignant tumor of colon	0.2	0.1	0.03	0.2	0.2	-0.01
Primary malignant neoplasm of prostate	0.2	0.1	0.01	0.2	0.3	-0.02
**Medication use**						
Agents acting on the renin-angiotensin system	10.5	10.1	0.01	12.6	15.5	-0.08
Antibacterials for systemic use	36.9	16.7	0.47	36.6	40.5	-0.08
Antidepressants	7.3	4.7	0.11	8.2	9.8	-0.05
Antiepileptics	2.4	2	0.03	2.7	3.5	-0.05
Antiinflammatory and antirheumatic products	35.2	20.1	0.34	38.1	42.8	-0.1
Antineoplastic agents	1	0.8	0.02	1.2	1.3	-0.01
Antipsoriatics	0.5	0.3	0.03	0.5	0.7	-0.02
Antithrombotic agents	8	7.6	0.01	9.4	11.3	-0.06
Beta blocking agents	6.3	6.4	0	7.5	9.1	-0.06

Table S6.26. *Continued.* Selected baseline characteristics for France IQVIA, for the long-term risk of alcohol misuse or dependence

Characteristic	Before propensity score matching			After propensity score matching		
	Targets, %	Comparators, %	Standardized difference	Targets, %	Comparators, %	Standardized difference
**Medication use**						
Calcium channel blockers	5.8	5.3	0.02	6.9	8.8	-0.07
Diuretics	5.3	5.5	-0.01	6.3	8	-0.06
Drugs for acid-related disorders	24.5	14.2	0.26	26.9	31	-0.09
Drugs for obstructive airway diseases	23.1	12.7	0.28	24.6	27.9	-0.08
Drugs used in diabetes	5.5	4.3	0.06	6.3	7.6	-0.05
Immunosuppressants	0.3	0.6	-0.04	0.4	0.5	-0.02
Lipid modifying agents	8.2	8.2	0	9.9	12	-0.07
Opioids	58.5	23.3	0.77	57	58.7	-0.04
Psycholeptics	15	8.5	0.2	16.5	19.5	-0.08
Psychostimulants, agents used for ADHD and nootropics	4.5	2	0.14	4.9	5.4	-0.02

Table S6.27. Selected baseline characteristics for France IQVIA, for the long-term risk of substance misuse or dependence

Characteristic	Before propensity score matching			After propensity score matching		
	Targets, %	Comparators, %	Standardized difference	Targets, %	Comparators, %	Standardized difference
**Age group (years)**						
0-4	0.7	3	-0.17	0.7	1.1	-0.04
5-9	1.8	4.4	-0.15	1.8	2	-0.02
10-14	3.2	4.4	-0.06	3.2	3.4	-0.01
15-19	5.6	4.4	0.06	5.2	5.4	-0.01
20-24	7.3	4.4	0.13	6.3	5.7	0.02
25-29	7.3	4.2	0.13	6.1	6	0.01
30-34	8.3	5.1	0.13	7.5	7	0.02
35-39	9	5.7	0.13	8.4	7.4	0.04
40-44	9.4	5.9	0.13	9.2	8.4	0.03
45-49	10.1	6.9	0.12	10.2	9.6	0.02
50-54	9.8	7.3	0.09	10.2	9.8	0.01
55-59	8.9	7.8	0.04	9.5	10.1	-0.02
60-64	6.2	7.8	-0.06	6.9	7	-0.01
65-69	4	7.7	-0.16	4.7	5.1	-0.02
70-74	3.7	8	-0.18	4.5	5	-0.03
75-79	2	5.3	-0.17	2.5	2.8	-0.02
80-84	1.3	4	-0.16	1.6	1.9	-0.02
85-89	0.9	2.6	-0.13	1	1.3	-0.03
90-94	0.4	1.1	-0.08	0.4	0.5	-0.02
95-99	0.1	0.2	-0.03	0.1	0.2	-0.01
**Sex**						
Female	56.4	57.5	-0.02	57.8	57.5	0

Table S6.27. *Continued.* Selected baseline characteristics for France IQVIA, for the long-term risk of substance misuse or dependence

**Characteristic**	**Before propensity score matching**			**After propensity score matching**		
	**Targets, %**	**Comparators, %**	**Standardized difference**	**Targets, %**	**Comparators, %**	**Standardized difference**
**Medical history (general)**						
Acute respiratory disease	16.7	6.8	0.31	16.8	19.6	-0.07
Chronic liver disease	0.1	0.1	0	0.1	0.1	-0.01
Chronic obstructive lung disease	0.9	0.7	0.02	1	1.4	-0.04
Crohn’s disease	0.1	0.1	0.01	0.1	0.2	-0.01
Dementia	0.1	0.2	0	0.1	0.2	-0.01
Depressive disorder	6.1	3.2	0.14	6.7	8	-0.05
Diabetes mellitus	5.2	4	0.06	6	7.3	-0.05
Gastroesophageal reflux disease	4.3	2	0.13	4.7	5.6	-0.04
Gastrointestinal hemorrhage	0.5	0.3	0.04	0.5	0.6	-0.02
Human immunodeficiency virus infection	0.2	0.1	0.03	0.2	0.4	-0.03
Hyperlipidemia	4	3	0.06	4.7	5.5	-0.04
Hypertensive disorder	11.7	10	0.06	13.8	17.1	-0.09
Lesion of liver	0.1	0.1	0	0.1	0.1	0
Obesity	0.4	0.1	0.05	0.4	0.3	0.01
Osteoarthritis	3.8	2.2	0.09	4.1	5	-0.05
Pneumonia	0.8	0.3	0.07	0.8	1	-0.01
Psoriasis	1	0.5	0.05	1	1.2	-0.02
Renal impairment	0.3	0.2	0.02	0.3	0.5	-0.02
Rheumatoid arthritis	0.2	0.3	-0.01	0.3	0.4	-0.02
Ulcerative colitis	0.1	0.1	0.01	0.1	0.1	0
Urinary tract infectious disease	1.5	0.7	0.08	1.6	2	-0.03

Table S6.27. *Continued.* Selected baseline characteristics for France IQVIA, for the long-term risk of substance misuse or dependence

Characteristic	Before propensity score matching			After propensity score matching		
	Targets, %	Comparators, %	Standardized difference	Targets, %	Comparators, %	Standardized difference
**Medical history (cardiovascular disease)**						
Atrial fibrillation	0.1	0.2	-0.02	0.2	0.2	-0.02
Cerebrovascular disease	0.9	0.7	0.02	1.1	1.4	-0.03
Coronary arteriosclerosis	0.4	0.4	0	0.5	0.6	-0.02
Heart disease	3.3	3.7	-0.02	3.8	5	-0.06
Heart failure	0.3	0.3	-0.01	0.3	0.5	-0.03
Ischemic heart disease	1	1	0	1.2	1.6	-0.03
Pulmonary embolism	0.2	0.1	0.02	0.2	0.3	-0.01
Venous thrombosis	0.3	0.2	0.03	0.4	0.5	-0.02
**Medical history (neoplasms)**						
Malignant neoplasm of anorectum	0.1	0.1	0.02	0.1	0.2	-0.01
Malignant neoplastic disease	1.1	0.8	0.03	1.3	1.8	-0.04
Malignant tumor of breast	0.3	0.2	0.02	0.4	0.5	-0.02
Malignant tumor of colon	0.2	0.1	0.03	0.2	0.2	0
Primary malignant neoplasm of prostate	0.2	0.1	0.01	0.2	0.2	-0.01
**Medication use**						
Agents acting on the renin-angiotensin system	10.5	10.1	0.01	12.6	15.5	-0.08
Antibacterials for systemic use	36.9	16.7	0.47	36.6	40.4	-0.08
Antidepressants	7.3	4.7	0.11	8.1	9.6	-0.06
Antiepileptics	2.4	1.9	0.03	2.7	3.5	-0.05
Antiinflammatory and antirheumatic products	35.2	20.2	0.34	38	42.6	-0.09
Antineoplastic agents	1	0.8	0.02	1.1	1.3	-0.01
Antipsoriatics	0.5	0.3	0.04	0.5	0.7	-0.02
Antithrombotic agents	8	7.7	0.01	9.4	11.3	-0.06
Beta blocking agents	6.3	6.5	-0.01	7.5	9.1	-0.06
Calcium channel blockers	5.8	5.4	0.02	6.9	8.8	-0.07

Table S6.27. *Continued.* Selected baseline characteristics for France IQVIA, for the long-term risk of substance misuse or dependence

Characteristic	Before propensity score matching			After propensity score matching		
	Targets, %	Comparators, %	Standardized difference	Targets, %	Comparators, %	Standardized difference
**Medication use**						
Diuretics	5.3	5.5	-0.01	6.3	8	-0.07
Drugs for acid-related disorders	24.5	14.3	0.26	26.8	30.8	-0.09
Drugs for obstructive airway diseases	23.1	12.6	0.28	24.5	27.8	-0.08
Drugs used in diabetes	5.5	4.4	0.05	6.3	7.5	-0.05
Immunosuppressants	0.3	0.5	-0.03	0.4	0.5	-0.02
Opioids	58.5	23.4	0.76	56.9	58.6	-0.03
Psycholeptics	15	8.7	0.2	16.3	19.2	-0.08
Psychostimulants, agents used for ADHD and nootropics	4.5	2	0.14	4.9	5.4	-0.03

Table S6.28. Selected baseline characteristics for France IQVIA, for the long-term risk of bipolar disorders

Characteristic	Before propensity score matching			After propensity score matching		
	Targets, %	Comparators, %	Standardized difference	Targets, %	Comparators, %	Standardized difference
**Age group (years)**						
0-4	0.7	3	-0.17	0.7	1.1	-0.04
5-9	1.8	4.4	-0.15	1.8	2.1	-0.02
10-14	3.2	4.4	-0.06	3.2	3.4	-0.01
15-19	5.6	4.3	0.06	5.1	5.3	-0.01
20-24	7.3	4.2	0.13	6.2	5.7	0.02
25-29	7.3	4.3	0.13	6.1	5.9	0.01
30-34	8.3	5	0.13	7.5	7	0.02
35-39	9	5.6	0.13	8.4	7.5	0.04
40-44	9.4	6	0.13	9.2	8.5	0.03
45-49	10.1	6.9	0.12	10.3	9.6	0.02
50-54	9.8	7.5	0.08	10.2	10	0.01
55-59	8.9	7.9	0.03	9.6	10.1	-0.02
60-64	6.2	7.8	-0.06	6.9	7	0
65-69	4	7.6	-0.16	4.7	5.1	-0.02
70-74	3.7	8	-0.19	4.4	5	-0.03
75-79	2	5.2	-0.17	2.5	2.8	-0.02
80-84	1.3	4	-0.17	1.6	1.9	-0.03
85-89	0.9	2.6	-0.13	1	1.3	-0.03
90-94	0.4	1.1	-0.08	0.4	0.5	-0.03
95-99	0.1	0.2	-0.03	0.1	0.2	-0.01
**Sex**						
Female	56.4	57.1	-0.01	57.6	57.5	0

Table S6.28. *Continued.* Selected baseline characteristics for France IQVIA, for the long-term risk of bipolar disorders

Characteristic	Before propensity score matching			After propensity score matching		
	**Targets, %**	**Comparators, %**	**Standardized difference**	**Targets, %**	**Comparators, %**	**Standardized difference**
**Medical history (general)**						
Acute respiratory disease	16.7	6.8	0.31	16.9	19.6	-0.07
Chronic liver disease	0.1	0.1	0.01	0.1	0.1	-0.01
Chronic obstructive lung disease	0.9	0.7	0.02	1.1	1.5	-0.04
Crohn’s disease	0.1	0.1	0.01	0.1	0.2	-0.01
Dementia	0.1	0.1	0	0.1	0.2	-0.01
Depressive disorder	6.1	3.2	0.14	6.8	8.2	-0.05
Diabetes mellitus	5.2	3.9	0.06	6	7.3	-0.05
Gastroesophageal reflux disease	4.3	2	0.13	4.7	5.6	-0.04
Gastrointestinal hemorrhage	0.5	0.3	0.04	0.5	0.6	-0.01
Human immunodeficiency virus infection	0.2	0.1	0.03	0.2	0.4	-0.03
Hyperlipidemia	4	3	0.05	4.7	5.5	-0.04
Hypertensive disorder	11.7	10	0.06	13.8	17.2	-0.09
Lesion of liver	0.1	0.1	0	0.1	0.1	-0.01
Obesity	0.4	0.1	0.05	0.4	0.3	0.01
Osteoarthritis	3.8	2.2	0.09	4.1	5.1	-0.05
Pneumonia	0.8	0.3	0.07	0.8	1	-0.02
Psoriasis	1	0.5	0.05	1.1	1.2	-0.02
Renal impairment	0.3	0.2	0.02	0.3	0.5	-0.02
Rheumatoid arthritis	0.2	0.3	-0.01	0.3	0.4	-0.02
Ulcerative colitis	0.1	0.1	0.01	0.1	0.1	0
Urinary tract infectious disease	1.5	0.7	0.08	1.6	2.1	-0.04

Table S6.28. *Continued.* Selected baseline characteristics for France IQVIA, for the long-term risk of bipolar disorders

**Characteristic**	**Before propensity score matching**			**After propensity score matching**		
	**Targets, %**	**Comparators, %**	**Standardized difference**	**Targets, %**	**Comparators, %**	**Standardized difference**
**Medical history (cardiovascular disease)**						
Atrial fibrillation	0.1	0.3	-0.03	0.2	0.2	-0.02
Cerebrovascular disease	0.9	0.8	0.02	1.1	1.4	-0.02
Coronary arteriosclerosis	0.4	0.4	0	0.5	0.6	-0.02
Heart disease	3.3	3.7	-0.03	3.9	5	-0.06
Heart failure	0.3	0.3	-0.01	0.3	0.5	-0.03
Peripheral vascular disease	0.2	0.2	0	0.2	0.3	-0.02
Pulmonary embolism	0.2	0.2	0.01	0.2	0.3	-0.02
Venous thrombosis	0.3	0.2	0.03	0.4	0.5	-0.02
**Medical history (neoplasms)**						
Malignant neoplasm of anorectum	0.1	0.1	0.02	0.1	0.2	-0.01
Malignant neoplastic disease	1.1	0.9	0.03	1.3	1.8	-0.04
Malignant tumor of breast	0.3	0.2	0.02	0.4	0.5	-0.01
Malignant tumor of colon	0.2	0.1	0.03	0.2	0.2	0
Primary malignant neoplasm of prostate	0.2	0.1	0.01	0.2	0.2	-0.01
**Medication use**						
Agents acting on the renin-angiotensin system	10.5	10.1	0.02	12.6	15.5	-0.09
Antibacterials for systemic use	36.9	16.7	0.47	36.7	40.6	-0.08
Antidepressants	7.3	4.7	0.11	8.2	9.8	-0.05
Antiepileptics	2.4	2	0.03	2.6	3.5	-0.05
Antiinflammatory and antirheumatic products	35.2	20.1	0.34	38.1	42.9	-0.1
Antineoplastic agents	1	0.8	0.02	1.1	1.3	-0.01
Antipsoriatics	0.5	0.3	0.03	0.5	0.7	-0.02
Antithrombotic agents	8	7.7	0.01	9.4	11.4	-0.06
Beta blocking agents	6.3	6.4	-0.01	7.5	9.1	-0.06
Calcium channel blockers	5.8	5.3	0.02	6.9	8.8	-0.07

Table S6.28. *Continued.* Selected baseline characteristics for France IQVIA, for the long-term risk of bipolar disorders

Characteristic	Before propensity score matching			After propensity score matching		
	**Targets, %**	**Comparators, %**	**Standardized difference**	**Targets, %**	**Comparators, %**	**Standardized difference**
**Medication use**						
Diuretics	5.3	5.6	-0.01	6.3	8	-0.06
Drugs for acid-related disorders	24.5	14.3	0.26	26.9	31	-0.09
Drugs for obstructive airway diseases	23.1	12.8	0.27	24.7	28.1	-0.08
Drugs used in diabetes	5.5	4.3	0.06	6.4	7.6	-0.05
Immunosuppressants	0.3	0.6	-0.04	0.4	0.5	-0.02
Lipid modifying agents	8.2	8.3	0	9.9	12	-0.06
Opioids	58.5	23.4	0.76	57	58.9	-0.04
Psycholeptics	15	8.6	0.2	16.6	19.5	-0.08
Psychostimulants, agents used for ADHD and nootropics	4.5	2	0.14	4.9	5.5	-0.03

Table S6.29. Selected baseline characteristics for France IQVIA, for the long-term risk of psychoses

Characteristic	Before propensity score matching			After propensity score matching		
	Targets, %	Comparators, %	Standardized difference	Targets, %	Comparators, %	Standardized difference
**Age group (years)**						
0-4	0.7	2.9	-0.17	0.7	1.1	-0.04
5-9	1.8	4.5	-0.15	1.8	2	-0.02
10-14	3.2	4.4	-0.06	3.2	3.4	-0.01
15-19	5.6	4.4	0.06	5.1	5.3	-0.01
20-24	7.3	4.2	0.13	6.2	5.7	0.02
25-29	7.3	4.3	0.13	6.1	5.9	0.01
30-34	8.3	5	0.13	7.5	7	0.02
35-39	9	5.6	0.13	8.4	7.4	0.04
40-44	9.4	5.9	0.13	9.2	8.4	0.03
45-49	10.1	6.9	0.12	10.3	9.7	0.02
50-54	9.8	7.4	0.09	10.2	10	0.01
55-59	8.9	7.8	0.04	9.6	10.2	-0.02
60-64	6.2	7.9	-0.07	6.9	7.1	-0.01
65-69	4	7.6	-0.16	4.7	5.1	-0.02
70-74	3.7	8	-0.19	4.5	5	-0.02
75-79	2	5.1	-0.17	2.5	2.8	-0.02
80-84	1.3	4	-0.16	1.6	1.9	-0.03
85-89	0.9	2.6	-0.13	0.9	1.3	-0.03
90-94	0.4	1.1	-0.08	0.4	0.5	-0.03
95-99	0.1	0.3	-0.04	0.1	0.2	-0.01
**Sex**						
Female	56.4	57.3	-0.02	57.6	57.3	0

Table S6.29. *Continued.* Selected baseline characteristics for France IQVIA, for the long-term risk of psychoses

Characteristic	Before propensity score matching			After propensity score matching		
	Targets, %	Comparators, %	Standardized difference	Targets, %	Comparators, %	Standardized difference
**Medical history (general)**						
Acute respiratory disease	16.7	6.7	0.31	16.9	19.6	-0.07
Chronic liver disease	0.1	0.1	0	0.1	0.1	-0.01
Chronic obstructive lung disease	0.9	0.7	0.02	1.1	1.5	-0.04
Crohn's disease	0.1	0.1	0.01	0.1	0.2	-0.01
Dementia	0.1	0.1	0	0.1	0.2	-0.01
Depressive disorder	6.1	3.2	0.14	6.9	8.1	-0.05
Diabetes mellitus	5.2	3.9	0.06	6	7.3	-0.05
Gastroesophageal reflux disease	4.3	2.1	0.13	4.7	5.7	-0.04
Gastrointestinal hemorrhage	0.5	0.3	0.04	0.5	0.6	-0.01
Human immunodeficiency virus infection	0.2	0.1	0.04	0.2	0.4	-0.03
Hyperlipidemia	4	3	0.05	4.7	5.5	-0.04
Hypertensive disorder	11.7	10	0.06	13.8	17.2	-0.09
Lesion of liver	0.1	0.1	0	0.1	0.1	-0.01
Obesity	0.4	0.1	0.05	0.4	0.3	0.01
Osteoarthritis	3.8	2.2	0.09	4.1	5.1	-0.05
Pneumonia	0.8	0.3	0.07	0.8	1	-0.02
Psoriasis	1	0.5	0.05	1.1	1.2	-0.01
Renal impairment	0.3	0.2	0.02	0.3	0.5	-0.02
Rheumatoid arthritis	0.2	0.3	-0.01	0.3	0.4	-0.02
Schizophrenia	0.1	0.1	0	0.1	0.1	0
Ulcerative colitis	0.1	0.1	0.01	0.1	0.1	0
Urinary tract infectious disease	1.5	0.7	0.08	1.6	2	-0.03

Table S6.29. *Continued.* Selected baseline characteristics for France IQVIA, for the long-term risk of psychoses

Characteristic	Before propensity score matching			After propensity score matching		
	Targets, %	Comparators, %	Standardized difference	Targets, %	Comparators, %	Standardized difference
**Medical history (cardiovascular disease)**						
Atrial fibrillation	0.1	0.3	-0.03	0.2	0.2	-0.02
Cerebrovascular disease	0.9	0.8	0.02	1.1	1.4	-0.02
Coronary arteriosclerosis	0.4	0.4	0.01	0.5	0.6	-0.01
Heart disease	3.3	3.6	-0.02	3.9	5.1	-0.06
Heart failure	0.3	0.3	-0.01	0.3	0.5	-0.03
Peripheral vascular disease	0.2	0.2	0.01	0.2	0.3	-0.01
Pulmonary embolism	0.2	0.1	0.02	0.2	0.3	-0.02
Venous thrombosis	0.3	0.2	0.03	0.4	0.5	-0.02
**Medical history (neoplasms)**						
Malignant neoplasm of anorectum	0.1	0.1	0.02	0.1	0.2	-0.01
Malignant neoplastic disease	1.1	0.9	0.03	1.3	1.8	-0.04
Malignant tumor of breast	0.3	0.2	0.02	0.4	0.5	-0.02
Malignant tumor of colon	0.2	0.1	0.03	0.2	0.2	-0.01
Primary malignant neoplasm of prostate	0.2	0.1	0.01	0.2	0.2	-0.01
**Medication use**						
Agents acting on the renin-angiotensin system	10.5	10	0.02	12.6	15.6	-0.09
Antibacterials for systemic use	36.9	16.5	0.47	36.6	40.6	-0.08
Antidepressants	7.3	4.7	0.11	8.3	9.8	-0.05
Antiepileptics	2.4	2	0.03	2.7	3.5	-0.05
Antiinflammatory and antirheumatic products	35.2	20.1	0.34	38.1	42.8	-0.1
Antineoplastic agents	1	0.8	0.03	1.1	1.3	-0.01
Antipsoriatics	0.5	0.3	0.04	0.5	0.7	-0.02
Antithrombotic agents	8	7.6	0.01	9.4	11.4	-0.06
Beta blocking agents	6.3	6.5	-0.01	7.5	9.1	-0.06
Calcium channel blockers	5.8	5.3	0.02	6.9	8.8	-0.07

Table S6.29. *Continued.* Selected baseline characteristics for France IQVIA, for the long-term risk of psychoses

Characteristic	Before propensity score matching			After propensity score matching		
	**Targets, %**	**Comparators, %**	**Standardized difference**	**Targets, %**	**Comparators, %**	**Standardized difference**
**Medication use**						
Diuretics	5.3	5.6	-0.01	6.4	8.1	-0.07
Drugs for acid-related disorders	24.5	14.1	0.27	26.9	31.1	-0.09
Drugs for obstructive airway diseases	23.1	12.7	0.28	24.7	28.1	-0.08
Drugs used in diabetes	5.5	4.3	0.06	6.4	7.6	-0.05
Immunosuppressants	0.3	0.5	-0.03	0.4	0.5	-0.02
Lipid modifying agents	8.2	8.2	0	9.9	12	-0.06
Opioids	58.5	23.4	0.76	57	58.9	-0.04
Psycholeptics	15	8.5	0.2	16.7	19.6	-0.08
Psychostimulants, agents used for ADHD and nootropics	4.5	2	0.14	4.9	5.5	-0.03

Table S6.30. Selected baseline characteristics for France IQVIA, for the long-term risk of personality disorders

Characteristic	Before propensity score matching			After propensity score matching		
	Targets, %	Comparators, %	Standardized difference	Targets, %	Comparators, %	Standardized difference
**Age group (years)**						
0-4	0.7	2.9	-0.17	0.7	1.1	-0.04
5-9	1.8	4.5	-0.15	1.8	2	-0.02
10-14	3.2	4.4	-0.06	3.2	3.4	-0.01
15-19	5.6	4.4	0.06	5.1	5.3	-0.01
20-24	7.3	4.2	0.13	6.2	5.7	0.02
25-29	7.3	4.3	0.13	6.1	5.9	0.01
30-34	8.3	5	0.13	7.5	7	0.02
35-39	9	5.6	0.13	8.4	7.4	0.04
40-44	9.4	5.9	0.13	9.2	8.4	0.03
45-49	10.1	6.9	0.12	10.3	9.7	0.02
50-54	9.8	7.4	0.09	10.2	10	0.01
55-59	8.9	7.8	0.04	9.6	10.2	-0.02
60-64	6.2	7.9	-0.07	6.9	7.1	-0.01
65-69	4	7.6	-0.16	4.7	5.1	-0.02
70-74	3.7	8	-0.19	4.5	5	-0.02
75-79	2	5.1	-0.17	2.5	2.8	-0.02
80-84	1.3	4	-0.16	1.6	1.9	-0.03
85-89	0.9	2.6	-0.13	0.9	1.3	-0.03
90-94	0.4	1.1	-0.08	0.4	0.5	-0.03
95-99	0.1	0.3	-0.04	0.1	0.2	-0.01
**Sex**						
Female	56.4	57.3	-0.02	57.6	57.3	0

Table S6.30. Continued. Selected baseline characteristics for France IQVIA, for the long-term risk of personality disorders

Characteristic	Before propensity score matching			After propensity score matching		
	Targets, %	Comparators, %	Standardized difference	Targets, %	Comparators, %	Standardized difference
**Medical history (general)**						
Acute respiratory disease	16.7	6.7	0.31	16.9	19.6	-0.07
Chronic liver disease	0.1	0.1	0	0.1	0.1	-0.01
Chronic obstructive lung disease	0.9	0.7	0.02	1.1	1.5	-0.04
Crohn’s disease	0.1	0.1	0.01	0.1	0.2	-0.01
Dementia	0.1	0.1	0	0.1	0.2	-0.01
Depressive disorder	6.1	3.2	0.14	6.9	8.1	-0.05
Diabetes mellitus	5.2	3.9	0.06	6	7.3	-0.05
Gastroesophageal reflux disease	4.3	2.1	0.13	4.7	5.7	-0.04
Gastrointestinal hemorrhage	0.5	0.3	0.04	0.5	0.6	-0.01
Human immunodeficiency virus infection	0.2	0.1	0.04	0.2	0.4	-0.03
Hyperlipidemia	4	3	0.05	4.7	5.5	-0.04
Hypertensive disorder	11.7	10	0.06	13.8	17.2	-0.09
Lesion of liver	0.1	0.1	0	0.1	0.1	-0.01
Obesity	0.4	0.1	0.05	0.4	0.3	0.01
Osteoarthritis	3.8	2.2	0.09	4.1	5.1	-0.05
Pneumonia	0.8	0.3	0.07	0.8	1	-0.02
Psoriasis	1	0.5	0.05	1.1	1.2	-0.01
Renal impairment	0.3	0.2	0.02	0.3	0.5	-0.02
Rheumatoid arthritis	0.2	0.3	-0.01	0.3	0.4	-0.02
Schizophrenia	0.1	0.1	0	0.1	0.1	0
Ulcerative colitis	0.1	0.1	0.01	0.1	0.1	0
Urinary tract infectious disease	1.5	0.7	0.08	1.6	2	-0.03

Table S6.30. Continued. Selected baseline characteristics for France IQVIA, for the long-term risk of personality disorders

Characteristic	Before propensity score matching			After propensity score matching		
	Targets, %	Comparators, %	Standardized difference	Targets, %	Comparators, %	Standardized difference
**Medical history (cardiovascular disease)**						
Atrial fibrillation	0.1	0.3	-0.03	0.2	0.2	-0.02
Cerebrovascular disease	0.9	0.8	0.02	1.1	1.4	-0.02
Coronary arteriosclerosis	0.4	0.4	0.01	0.5	0.6	-0.01
Heart disease	3.3	3.6	-0.02	3.9	5.1	-0.06
Heart failure	0.3	0.3	-0.01	0.3	0.5	-0.03
Peripheral vascular disease	0.2	0.2	0.01	0.2	0.3	-0.01
Pulmonary embolism	0.2	0.1	0.02	0.2	0.3	-0.02
Venous thrombosis	0.3	0.2	0.03	0.4	0.5	-0.02
**Medical history (neoplasms)**						
Malignant neoplasm of anorectum	0.1	0.1	0.02	0.1	0.2	-0.01
Malignant neoplastic disease	1.1	0.9	0.03	1.3	1.8	-0.04
Malignant tumor of breast	0.3	0.2	0.02	0.4	0.5	-0.02
Malignant tumor of colon	0.2	0.1	0.03	0.2	0.2	-0.01
Primary malignant neoplasm of prostate	0.2	0.1	0.01	0.2	0.2	-0.01
**Medication use**						
Agents acting on the renin-angiotensin system	10.5	10	0.02	12.6	15.6	-0.09
Antibacterials for systemic use	36.9	16.5	0.47	36.6	40.6	-0.08
Antidepressants	7.3	4.7	0.11	8.3	9.8	-0.05
Antiepileptics	2.4	2	0.03	2.7	3.5	-0.05
Antiinflammatory and antirheumatic products	35.2	20.1	0.34	38.1	42.8	-0.1
Antineoplastic agents	1	0.8	0.03	1.1	1.3	-0.01
Antipsoriatics	0.5	0.3	0.04	0.5	0.7	-0.02
Antithrombotic agents	8	7.6	0.01	9.4	11.4	-0.06
Beta blocking agents	6.3	6.5	-0.01	7.5	9.1	-0.06
Calcium channel blockers	5.8	5.3	0.02	6.9	8.8	-0.07

Table S6.30. Continued. Selected baseline characteristics for France IQVIA, for the long-term risk of personality disorders

**Characteristic**	**Before propensity score matching**			**After propensity score matching**		
	**Targets, %**	**Comparators, %**	**Standardized difference**	**Targets, %**	**Comparators, %**	**Standardized difference**
**Medication use**						
Diuretics	5.3	5.6	-0.01	6.4	8.1	-0.07
Drugs for acid-related disorders	24.5	14.1	0.27	26.9	31.1	-0.09
Drugs for obstructive airway diseases	23.1	12.7	0.28	24.7	28.1	-0.08
Drugs used in diabetes	5.5	4.3	0.06	6.4	7.6	-0.05
Immunosuppressants	0.3	0.5	-0.03	0.4	0.5	-0.02
Lipid modifying agents	8.2	8.2	0	9.9	12	-0.06
Opioids	58.5	23.4	0.76	57	58.9	-0.04
Psycholeptics	15	8.5	0.2	16.7	19.6	-0.08
Psychostimulants, agents used for ADHD and nootropics	4.5	2	0.14	4.9	5.5	-0.03

Table S6.31. Selected baseline characteristics for France IQVIA, for the long-term risk of self-harm and suicide

**Characteristic**	**Before propensity score matching**			**After propensity score matching**		
	**Targets, %**	**Comparators, %**	**Standardized difference**	**Targets, %**	**Comparators, %**	**Standardized difference**
**Age group (years)**						
0-4	0.7	3	-0.17	0.7	1.1	-0.04
5-9	1.8	4.4	-0.15	1.8	2	-0.01
10-14	3.2	4.4	-0.06	3.2	3.3	-0.01
15-19	5.6	4.3	0.06	5.1	5.3	-0.01
20-24	7.3	4.2	0.13	6.2	5.7	0.02
25-29	7.3	4.3	0.13	6.1	5.8	0.01
30-34	8.3	5	0.13	7.5	7	0.02
35-39	9	5.6	0.13	8.4	7.5	0.04
40-44	9.4	5.9	0.13	9.2	8.5	0.03
45-49	10.1	7	0.11	10.3	9.6	0.02
50-54	9.8	7.5	0.08	10.2	9.9	0.01
55-59	8.9	7.8	0.04	9.6	10.2	-0.02
60-64	6.2	7.8	-0.06	6.9	7	0
65-69	4	7.8	-0.16	4.7	5.2	-0.02
70-74	3.7	7.9	-0.18	4.5	5	-0.03
75-79	2	5.1	-0.17	2.5	2.8	-0.02
80-84	1.3	4	-0.16	1.6	1.9	-0.03
85-89	0.9	2.5	-0.13	0.9	1.3	-0.03
90-94	0.4	1.1	-0.08	0.4	0.5	-0.02
95-99	0.1	0.3	-0.03	0.1	0.2	-0.01
**Sex**						
Female	56.4	57.5	-0.02	57.6	57.4	0

Table S6.31. *Continued.* Selected baseline characteristics for France IQVIA, for the long-term risk of self-harm and suicide

Characteristic	Before propensity score matching			After propensity score matching		
	Targets, %	Comparators, %	Standardized difference	Targets, %	Comparators, %	Standardized difference
**Medical history (general)**						
Acute respiratory disease	16.7	6.8	0.31	16.9	19.6	-0.07
Chronic liver disease	0.1	0.1	0.01	0.1	0.1	-0.01
Chronic obstructive lung disease	0.9	0.7	0.02	1.1	1.5	-0.04
Crohn's disease	0.1	0.1	0.01	0.1	0.2	-0.01
Dementia	0.1	0.1	0	0.1	0.2	-0.01
Depressive disorder	6.1	3.2	0.14	6.9	8.2	-0.05
Diabetes mellitus	5.2	3.9	0.06	6	7.3	-0.05
Gastroesophageal reflux disease	4.3	2	0.13	4.7	5.7	-0.04
Gastrointestinal hemorrhage	0.5	0.2	0.04	0.5	0.6	-0.01
Human immunodeficiency virus infection	0.2	0.1	0.04	0.2	0.4	-0.03
Hyperlipidemia	4	3	0.06	4.7	5.6	-0.04
Hypertensive disorder	11.7	10	0.06	13.8	17.2	-0.09
Lesion of liver	0.1	0.1	0	0.1	0.1	-0.01
Obesity	0.4	0.1	0.05	0.4	0.3	0.01
Osteoarthritis	3.8	2.3	0.09	4.1	5.1	-0.05
Pneumonia	0.8	0.3	0.07	0.8	1	-0.02
Psoriasis	1	0.5	0.05	1.1	1.3	-0.02
Renal impairment	0.3	0.2	0.02	0.3	0.5	-0.02
Rheumatoid arthritis	0.2	0.3	-0.01	0.3	0.4	-0.02
Ulcerative colitis	0.1	0.1	0.01	0.1	0.1	0
Urinary tract infectious disease	1.5	0.7	0.08	1.6	2	-0.04

Table S6.31. *Continued.* Selected baseline characteristics for France IQVIA, for the long-term risk of self-harm and suicide

**Characteristic**	**Before propensity score matching**			**After propensity score matching**		
	**Targets, %**	**Comparators, %**	**Standardized difference**	**Targets, %**	**Comparators, %**	**Standardized difference**
**Medical history (cardiovascular disease)**						
Atrial fibrillation	0.1	0.3	-0.03	0.2	0.2	-0.02
Cerebrovascular disease	0.9	0.8	0.02	1.1	1.4	-0.03
Coronary arteriosclerosis	0.4	0.4	0	0.5	0.6	-0.01
Heart disease	3.3	3.6	-0.02	3.9	5	-0.06
Heart failure	0.3	0.3	-0.01	0.3	0.5	-0.03
Ischemic heart disease	1	1	0	1.2	1.6	-0.03
Pulmonary embolism	0.2	0.1	0.01	0.2	0.3	-0.01
Venous thrombosis	0.3	0.2	0.03	0.4	0.5	-0.02
**Medical history (neoplasms)**						
Malignant neoplasm of anorectum	0.1	0.1	0.02	0.1	0.2	-0.01
Malignant neoplastic disease	1.1	0.9	0.03	1.3	1.8	-0.04
Malignant tumor of breast	0.3	0.2	0.02	0.4	0.5	-0.02
Malignant tumor of colon	0.2	0.1	0.02	0.2	0.2	-0.01
Primary malignant neoplasm of prostate	0.2	0.1	0.01	0.2	0.3	-0.01
**Medication use**						
Agents acting on the renin-angiotensin system	10.5	10	0.02	12.6	15.6	-0.09
Antibacterials for systemic use	36.9	16.6	0.47	36.7	40.6	-0.08
Antidepressants	7.3	4.6	0.11	8.3	9.9	-0.06
Antiepileptics	2.4	1.9	0.03	2.7	3.5	-0.05
Antiinflammatory and antirheumatic products	35.2	20	0.35	38.1	42.9	-0.1
Antineoplastic agents	1	0.8	0.03	1.1	1.3	-0.01
Antipsoriatics	0.5	0.3	0.03	0.5	0.7	-0.02
Antithrombotic agents	8	7.5	0.02	9.4	11.4	-0.06
Beta blocking agents	6.3	6.4	0	7.5	9.2	-0.06
Calcium channel blockers	5.8	5.3	0.02	6.9	8.9	-0.07

Table S6.31. *Continued.* Selected baseline characteristics for France IQVIA, for the long-term risk of self-harm and suicide

**Characteristic**	**Before propensity score matching**			**After propensity score matching**		
	**Targets, %**	**Comparators, %**	**Standardized difference**	**Targets, %**	**Comparators, %**	**Standardized difference**
**Medication use**						
Diuretics	5.3	5.4	-0.01	6.3	8	-0.07
Drugs for acid-related disorders	24.5	14.3	0.26	26.9	31.1	-0.09
Drugs for obstructive airway diseases	23.1	12.7	0.28	24.7	28.2	-0.08
Drugs used in diabetes	5.5	4.3	0.06	6.4	7.6	-0.05
Immunosuppressants	0.3	0.5	-0.03	0.4	0.5	-0.02
Lipid modifying agents	8.2	8.1	0	9.9	12	-0.07
Opioids	58.5	23.4	0.77	57	59	-0.04
Psycholeptics	15	8.6	0.2	16.7	19.7	-0.08
Psychostimulants, agents used for ADHD and nootropics	4.5	2	0.14	4.9	5.4	-0.03

Table S6.32. Selected baseline characteristics for France IQVIA, for the long-term risk of sleep disorders

Characteristic	Before propensity score matching			After propensity score matching		
	Targets, %	Comparators, %	Standardized difference	Targets, %	Comparators, %	Standardized difference
**Age group (years)**						
0-4	0.7	2.9	-0.17	0.8	1.2	-0.04
5-9	1.8	4.5	-0.15	1.9	2.2	-0.02
10-14	3.2	4.3	-0.06	3.4	3.6	-0.01
15-19	5.6	4.3	0.06	5.4	5.6	-0.01
20-24	7.3	4.3	0.13	6.5	6	0.02
25-29	7.3	4.3	0.13	6.4	6.1	0.01
30-34	8.3	5	0.13	7.7	7.3	0.02
35-39	9	5.6	0.13	8.6	7.6	0.04
40-44	9.4	5.9	0.13	9.4	8.6	0.03
45-49	10.1	7	0.11	10.3	9.6	0.02
50-54	9.8	7.5	0.08	10	9.7	0.01
55-59	8.9	7.8	0.04	9.3	9.9	-0.02
60-64	6.2	7.8	-0.06	6.7	6.9	-0.01
65-69	4	7.7	-0.16	4.6	4.9	-0.01
70-74	3.7	8	-0.19	4.2	4.8	-0.03
75-79	2	5.2	-0.17	2.2	2.6	-0.03
80-84	1.3	4.1	-0.17	1.4	1.7	-0.03
85-89	0.9	2.6	-0.13	0.8	1.2	-0.03
90-94	0.4	1	-0.07	0.3	0.5	-0.03
95-99	0.1	0.2	-0.03	0.1	0.2	-0.01
**Sex**						
Female	56.4	57.4	-0.02	57.1	56.8	0.01

Table S6.32. *Continued.* Selected baseline characteristics for France IQVIA, for the long-term risk of sleep disorders

Characteristic	Before propensity score matching			After propensity score matching		
	**Targets, %**	**Comparators, %**	**Standardized difference**	**Targets, %**	**Comparators, %**	**Standardized difference**
**Medical history (general)**						
Acute respiratory disease	16.7	6.8	0.31	16.4	19.2	-0.07
Chronic liver disease	0.1	0.1	0.01	0.1	0.1	-0.01
Chronic obstructive lung disease	0.9	0.7	0.02	1	1.3	-0.03
Crohn’s disease	0.1	0.1	0.01	0.1	0.2	-0.01
Dementia	0.1	0.1	0	0.1	0.1	-0.01
Depressive disorder	6.1	3.2	0.14	5.8	7.1	-0.05
Diabetes mellitus	5.2	4	0.06	5.7	7	-0.05
Gastroesophageal reflux disease	4.3	2.1	0.13	4.3	5.1	-0.04
Gastrointestinal hemorrhage	0.5	0.3	0.04	0.5	0.6	-0.02
Human immunodeficiency virus infection	0.2	0.1	0.04	0.2	0.4	-0.03
Hyperlipidemia	4	2.9	0.06	4.4	5.2	-0.04
Hypertensive disorder	11.7	9.9	0.06	12.9	16.1	-0.09
Lesion of liver	0.1	0.1	0	0.1	0.1	-0.01
Obesity	0.4	0.1	0.05	0.3	0.3	0.01
Osteoarthritis	3.8	2.2	0.09	3.7	4.7	-0.05
Pneumonia	0.8	0.3	0.07	0.8	0.9	-0.02
Psoriasis	1	0.5	0.05	1	1.1	-0.01
Renal impairment	0.3	0.2	0.02	0.3	0.4	-0.02
Rheumatoid arthritis	0.2	0.3	-0.01	0.2	0.4	-0.03
Ulcerative colitis	0.1	0.1	0.01	0.1	0.1	0
Urinary tract infectious disease	1.5	0.7	0.08	1.5	1.9	-0.03

Table S6.32. *Continued.* Selected baseline characteristics for France IQVIA, for the long-term risk of sleep disorders

Characteristic	Before propensity score matching			After propensity score matching		
	Targets, %	Comparators, %	Standardized difference	Targets, %	Comparators, %	Standardized difference
**Medical history (cardiovascular disease)**						
Atrial fibrillation	0.1	0.2	-0.02	0.1	0.2	-0.01
Cerebrovascular disease	0.9	0.8	0.02	1	1.3	-0.03
Coronary arteriosclerosis	0.4	0.4	0	0.5	0.6	-0.01
Heart disease	3.3	3.6	-0.02	3.5	4.7	-0.06
Heart failure	0.3	0.3	0	0.3	0.4	-0.03
Ischemic heart disease	1	1	0	1.1	1.5	-0.03
Peripheral vascular disease	0.2	0.2	0	0.2	0.3	-0.01
Pulmonary embolism	0.2	0.1	0.01	0.2	0.3	-0.01
Venous thrombosis	0.3	0.2	0.03	0.3	0.5	-0.02
**Medical history (neoplasms)**						
Malignant neoplasm of anorectum	0.1	0.1	0.03	0.1	0.2	-0.01
Malignant neoplastic disease	1.1	0.8	0.03	1.2	1.6	-0.04
Malignant tumor of breast	0.3	0.2	0.02	0.4	0.4	-0.01
Malignant tumor of colon	0.2	0.1	0.03	0.1	0.2	-0.01
Primary malignant neoplasm of prostate	0.2	0.1	0.01	0.2	0.2	-0.01
**Medication use**						
Agents acting on the renin-angiotensin system	10.5	10.1	0.01	11.9	14.7	-0.08
Antibacterials for systemic use	36.9	16.6	0.47	35.8	39.7	-0.08
Antidepressants	7.3	4.7	0.11	7	8.5	-0.06
Antiepileptics	2.4	2	0.03	2.4	3.2	-0.05
Antiinflammatory and antirheumatic products	35.2	20.2	0.34	36.8	41.6	-0.1
Antineoplastic agents	1	0.8	0.02	1	1.2	-0.02
Antipsoriatics	0.5	0.3	0.03	0.5	0.7	-0.02
Antithrombotic agents	8	7.7	0.01	8.7	10.7	-0.07
Beta blocking agents	6.3	6.4	0	7	8.6	-0.06

Table S6.32. *Continued.* Selected baseline characteristics for France IQVIA, for the long-term risk of sleep disorders

Characteristic	Before propensity score matching			After propensity score matching		
	**Targets, %**	**Comparators, %**	**Standardized difference**	**Targets, %**	**Comparators, %**	**Standardized difference**
**Medication use**						
Calcium channel blockers	5.8	5.3	0.02	6.5	8.3	-0.07
Diuretics	5.3	5.5	-0.01	5.9	7.5	-0.06
Drugs for acid-related disorders	24.5	14.3	0.26	25.4	29.3	-0.09
Drugs for obstructive airway diseases	23.1	12.7	0.28	23.7	27.1	-0.08
Drugs used in diabetes	5.5	4.3	0.05	6	7.3	-0.05
Immunosuppressants	0.3	0.5	-0.03	0.4	0.5	-0.02
Lipid modifying agents	8.2	8.2	0	9.3	11.4	-0.07
Opioids	58.5	23.4	0.76	56	57.7	-0.03
Psycholeptics	15	8.6	0.2	13.1	15.3	-0.06
Psychostimulants, agents used for ADHD and nootropics	4.5	2	0.14	4.5	5.1	-0.03

Table S6.33. Selected baseline characteristics for France IQVIA, for the long-term risk of dementia

Characteristic	Before propensity score matching			After propensity score matching		
	Targets, %	Comparators, %	Standardized difference	Targets, %	Comparators, %	Standardized difference
**Age group (years)**						
0-4	0.7	3	-0.17	0.7	1.1	-0.04
5-9	1.8	4.4	-0.15	1.8	2	-0.02
10-14	3.2	4.4	-0.06	3.2	3.3	-0.01
15-19	5.6	4.3	0.06	5.1	5.3	-0.01
20-24	7.3	4.3	0.13	6.2	5.7	0.02
25-29	7.3	4.4	0.13	6.1	5.9	0.01
30-34	8.3	5.1	0.13	7.5	7	0.02
35-39	9	5.6	0.13	8.5	7.5	0.04
40-44	9.4	5.9	0.13	9.3	8.5	0.03
45-49	10.1	6.8	0.12	10.3	9.7	0.02
50-54	9.8	7.4	0.09	10.2	10	0.01
55-59	8.9	7.8	0.04	9.6	10.2	-0.02
60-64	6.2	7.8	-0.07	6.9	7	0
65-69	4	7.7	-0.16	4.7	5.1	-0.02
70-74	3.7	8	-0.18	4.5	5	-0.02
75-79	2	5.2	-0.17	2.4	2.8	-0.02
80-84	1.3	4	-0.16	1.6	1.8	-0.02
85-89	0.9	2.6	-0.13	0.9	1.3	-0.03
90-94	0.4	1.1	-0.08	0.3	0.5	-0.02
95-99	0.1	0.2	-0.03	0.1	0.2	-0.02
**Sex**						
Female	56.4	57.5	-0.02	57.6	57.4	0

Table S6.33. *Continued.* Selected baseline characteristics for France IQVIA, for the long-term risk of dementia

Characteristic	Before propensity score matching			After propensity score matching		
	Targets, %	Comparators, %	Standardized difference	Targets, %	Comparators, %	Standardized difference
**Medical history (general)**						
Acute respiratory disease	16.7	6.8	0.31	16.9	19.6	-0.07
Chronic liver disease	0.1	0.1	0.01	0.1	0.1	-0.01
Chronic obstructive lung disease	0.9	0.7	0.02	1.1	1.5	-0.04
Crohn’s disease	0.1	0.1	0.01	0.1	0.2	-0.01
Depressive disorder	6.1	3.2	0.14	6.9	8.2	-0.05
Diabetes mellitus	5.2	4	0.06	6	7.3	-0.05
Gastroesophageal reflux disease	4.3	2.1	0.13	4.7	5.6	-0.04
Gastrointestinal hemorrhage	0.5	0.3	0.04	0.5	0.6	-0.01
Human immunodeficiency virus infection	0.2	0.1	0.03	0.2	0.4	-0.03
Hyperlipidemia	4	2.9	0.06	4.7	5.6	-0.04
Hypertensive disorder	11.7	10	0.06	13.8	17.1	-0.09
Lesion of liver	0.1	0.1	0	0.1	0.1	-0.01
Obesity	0.4	0.1	0.05	0.4	0.3	0.01
Osteoarthritis	3.8	2.1	0.1	4.1	5.1	-0.05
Pneumonia	0.8	0.3	0.07	0.8	1	-0.02
Psoriasis	1	0.5	0.05	1.1	1.3	-0.02
Renal impairment	0.3	0.2	0.02	0.3	0.5	-0.02
Rheumatoid arthritis	0.2	0.3	-0.01	0.3	0.4	-0.02
Ulcerative colitis	0.1	0.1	0.01	0.1	0.1	0
Urinary tract infectious disease	1.5	0.7	0.08	1.6	2	-0.04

Table S6.33. *Continued.* Selected baseline characteristics for France IQVIA, for the long-term risk of dementia

Characteristic	Before propensity score matching			After propensity score matching		
	**Targets, %**	**Comparators, %**	**Standardized difference**	**Targets, %**	**Comparators, %**	**Standardized difference**
**Medical history (cardiovascular disease)**						
Atrial fibrillation	0.1	0.3	-0.03	0.2	0.2	-0.01
Cerebrovascular disease	0.9	0.8	0.02	1.1	1.4	-0.02
Coronary arteriosclerosis	0.4	0.4	0.01	0.5	0.6	-0.01
Heart disease	3.3	3.6	-0.02	3.8	5	-0.06
Heart failure	0.3	0.3	0	0.3	0.5	-0.03
Ischemic heart disease	1	1	0	1.2	1.6	-0.03
Peripheral vascular disease	0.2	0.2	0	0.2	0.3	-0.02
Pulmonary embolism	0.2	0.1	0.02	0.2	0.3	-0.02
Venous thrombosis	0.3	0.2	0.03	0.4	0.5	-0.02
**Medical history (neoplasms)**						
Malignant neoplasm of anorectum	0.1	0.1	0.03	0.1	0.2	-0.01
Malignant neoplastic disease	1.1	0.8	0.03	1.3	1.8	-0.04
Malignant tumor of breast	0.3	0.2	0.02	0.4	0.5	-0.02
Malignant tumor of colon	0.2	0.1	0.03	0.2	0.2	-0.01
Primary malignant neoplasm of prostate	0.2	0.1	0.01	0.2	0.3	-0.02
**Medication use**						
Agents acting on the renin-angiotensin system	10.5	10	0.02	12.6	15.5	-0.09
Antibacterials for systemic use	36.9	16.7	0.47	36.7	40.5	-0.08
Antidepressants	7.3	4.7	0.11	8.3	9.8	-0.05
Antiepileptics	2.4	1.9	0.03	2.7	3.5	-0.05
Antiinflammatory and antirheumatic products	35.2	20.1	0.34	38.1	42.8	-0.1
Antineoplastic agents	1	0.8	0.02	1.1	1.3	-0.01
Antipsoriatics	0.5	0.3	0.03	0.5	0.7	-0.02
Antithrombotic agents	8	7.7	0.01	9.4	11.2	-0.06
Beta blocking agents	6.3	6.5	-0.01	7.5	9.1	-0.06

Table S6.33. *Continued.* Selected baseline characteristics for France IQVIA, for the long-term risk of dementia

**Characteristic**	**Before propensity score matching**			**After propensity score matching**		
	**Targets, %**	**Comparators, %**	**Standardized difference**	**Targets, %**	**Comparators, %**	**Standardized difference**
**Medication use**						
Calcium channel blockers	5.8	5.4	0.02	6.9	8.8	-0.07
Diuretics	5.3	5.6	-0.01	6.3	8	-0.06
Drugs for acid-related disorders	24.5	14.4	0.26	26.9	31	-0.09
Drugs for obstructive airway diseases	23.1	12.7	0.28	24.7	28.1	-0.08
Drugs used in diabetes	5.5	4.3	0.05	6.3	7.6	-0.05
Immunosuppressants	0.3	0.6	-0.04	0.4	0.5	-0.02
Opioids	58.5	23.4	0.76	57.1	58.9	-0.04
Psycholeptics	15	8.6	0.2	16.7	19.7	-0.08
Psychostimulants, agents used for ADHD and nootropics	4.5	2	0.14	4.9	5.5	-0.03

Table S6.34. Selected baseline characteristics for France IQVIA, for the long-term risk of neurodevelopmental disorders

Characteristic	Before propensity score matching			After propensity score matching		
	Targets, %	Comparators, %	Standardized difference	Targets, %	Comparators, %	Standardized difference
**Age group (years)**						
0-4	0.7	2.9	-0.17	0.7	1.1	-0.04
5-9	1.8	4.5	-0.15	1.7	2	-0.01
10-14	3.2	4.3	-0.06	3.1	3.3	-0.01
15-19	5.6	4.3	0.06	5.1	5.3	-0.01
20-24	7.3	4.3	0.13	6.2	5.7	0.02
25-29	7.3	4.3	0.13	6.1	5.9	0.01
30-34	8.3	5.1	0.13	7.5	7.1	0.02
35-39	9	5.6	0.13	8.5	7.5	0.04
40-44	9.4	6	0.13	9.3	8.5	0.03
45-49	10.1	6.8	0.12	10.3	9.7	0.02
50-54	9.8	7.5	0.08	10.2	10	0.01
55-59	8.9	7.9	0.03	9.6	10.2	-0.02
60-64	6.2	7.8	-0.06	6.9	7	0
65-69	4	7.7	-0.16	4.7	5.1	-0.02
70-74	3.7	7.9	-0.18	4.5	5	-0.03
75-79	2	5.2	-0.17	2.5	2.8	-0.02
80-84	1.3	4	-0.17	1.6	1.9	-0.02
85-89	0.9	2.6	-0.13	1	1.3	-0.03
90-94	0.4	1	-0.07	0.4	0.5	-0.02
95-99	0.1	0.2	-0.03	0.1	0.2	-0.01
**Sex**						
Female	56.4	57.4	-0.02	57.6	57.4	0

Table S6.34. *Continued.* Selected baseline characteristics for France IQVIA, for the long-term risk of neurodevelopmental disorders

Characteristic	Before propensity score matching			After propensity score matching		
	Targets, %	Comparators, %	Standardized difference	Targets, %	Comparators, %	Standardized difference
**Medical history (general)**						
Acute respiratory disease	16.7	6.7	0.31	16.9	19.5	-0.07
Chronic liver disease	0.1	0.1	0.01	0.1	0.1	-0.01
Chronic obstructive lung disease	0.9	0.8	0.01	1.1	1.5	-0.04
Crohn's disease	0.1	0.1	0.01	0.1	0.2	-0.01
Dementia	0.1	0.1	0	0.1	0.2	-0.01
Depressive disorder	6.1	3.1	0.14	6.9	8.2	-0.05
Diabetes mellitus	5.2	4	0.06	6	7.3	-0.05
Gastroesophageal reflux disease	4.3	2	0.13	4.7	5.6	-0.04
Gastrointestinal hemorrhage	0.5	0.3	0.04	0.5	0.6	-0.01
Human immunodeficiency virus infection	0.2	0.1	0.03	0.2	0.4	-0.03
Hyperlipidemia	4	3	0.06	4.8	5.5	-0.04
Hypertensive disorder	11.7	9.9	0.06	13.8	17.2	-0.09
Lesion of liver	0.1	0.1	0	0.1	0.1	-0.01
Obesity	0.4	0.1	0.05	0.4	0.3	0.01
Osteoarthritis	3.8	2.2	0.09	4.1	5	-0.05
Pneumonia	0.8	0.3	0.07	0.8	1	-0.02
Psoriasis	1	0.5	0.05	1.1	1.2	-0.01
Renal impairment	0.3	0.2	0.02	0.3	0.5	-0.02
Rheumatoid arthritis	0.2	0.3	-0.01	0.3	0.4	-0.02
Ulcerative colitis	0.1	0.1	0.01	0.1	0.1	0
Urinary tract infectious disease	1.5	0.7	0.08	1.6	2	-0.03

Table S6.34. *Continued.* Selected baseline characteristics for France IQVIA, for the long-term risk of neurodevelopmental disorders

**Characteristic**	**Before propensity score matching**			**After propensity score matching**		
	**Targets, %**	**Comparators, %**	**Standardized difference**	**Targets, %**	**Comparators, %**	**Standardized difference**
**Medical history (cardiovascular disease)**						
Atrial fibrillation	0.1	0.3	-0.03	0.2	0.2	-0.01
Cerebrovascular disease	0.9	0.7	0.02	1.1	1.4	-0.02
Coronary arteriosclerosis	0.4	0.4	0	0.5	0.6	-0.02
Heart disease	3.3	3.7	-0.02	3.9	5.1	-0.06
Heart failure	0.3	0.3	-0.01	0.3	0.5	-0.03
Ischemic heart disease	1	1	0	1.2	1.6	-0.03
Peripheral vascular disease	0.2	0.2	0	0.2	0.3	-0.01
Pulmonary embolism	0.2	0.1	0.01	0.2	0.3	-0.02
Venous thrombosis	0.3	0.2	0.03	0.4	0.5	-0.01
**Medical history (neoplasms)**						
Malignant neoplasm of anorectum	0.1	0.1	0.02	0.1	0.2	-0.01
Malignant neoplastic disease	1.1	0.8	0.03	1.3	1.8	-0.03
Malignant tumor of breast	0.3	0.2	0.02	0.4	0.5	-0.01
Malignant tumor of colon	0.2	0.1	0.03	0.2	0.2	-0.01
Primary malignant neoplasm of prostate	0.2	0.1	0.01	0.2	0.2	-0.01
**Medication use**						
Agents acting on the renin-angiotensin system	10.5	10.1	0.01	12.6	15.5	-0.08
Antibacterials for systemic use	36.9	16.7	0.47	36.7	40.5	-0.08
Antidepressants	7.3	4.7	0.11	8.3	9.8	-0.05
Antiepileptics	2.4	1.9	0.03	2.7	3.5	-0.04
Antiinflammatory and antirheumatic products	35.2	20.3	0.34	38.2	42.8	-0.1
Antineoplastic agents	1	0.8	0.02	1.1	1.3	-0.01
Antipsoriatics	0.5	0.3	0.03	0.5	0.7	-0.02
Antithrombotic agents	8	7.7	0.01	9.4	11.3	-0.06
Beta blocking agents	6.3	6.5	-0.01	7.5	9.1	-0.06

Table S6.34. *Continued.* Selected baseline characteristics for France IQVIA, for the long-term risk of neurodevelopmental disorders

Characteristic	Before propensity score matching			After propensity score matching		
	**Targets, %**	**Comparators, %**	**Standardized difference**	**Targets, %**	**Comparators, %**	**Standardized difference**
**Medication use**						
Calcium channel blockers	5.8	5.3	0.02	7	8.9	-0.07
Diuretics	5.3	5.6	-0.01	6.4	8	-0.06
Drugs for acid-related disorders	24.5	14.4	0.26	27	31	-0.09
Drugs for obstructive airway diseases	23.1	12.7	0.28	24.7	28.1	-0.08
Drugs used in diabetes	5.5	4.4	0.05	6.4	7.6	-0.05
Immunosuppressants	0.3	0.5	-0.03	0.4	0.5	-0.02
Lipid modifying agents	8.2	8.2	0	10	11.9	-0.06
Opioids	58.5	23.3	0.77	57.1	58.7	-0.03
Psycholeptics	15	8.6	0.2	16.7	19.7	-0.08
Psychostimulants, agents used for ADHD and nootropics	4.5	2	0.14	4.8	5.4	-0.03

Table S6.35. Selected baseline characteristics for France IQVIA, for the long-term risk of any of psychiatric and neuropsychiatric disorders

Characteristic	Before propensity score matching			After propensity score matching		
	Targets, %	Comparators, %	Standardized difference	Targets, %	Comparators, %	Standardized difference
0-4	0.7	3	-0.17	0.9	1.5	-0.05
5-9	1.8	4.5	-0.15	2.3	2.6	-0.02
10-14	3.2	4.4	-0.06	4	4.2	-0.01
15-19	5.6	4.4	0.06	6.1	6.4	-0.01
20-24	7.3	4.3	0.13	7	6.3	0.03
25-29	7.3	4.3	0.13	6.6	6.3	0.01
30-34	8.3	5.1	0.13	7.8	7.4	0.02
35-39	9	5.5	0.13	8.3	7.5	0.03
40-44	9.4	5.9	0.13	9.1	8.4	0.03
45-49	10.1	6.9	0.12	9.8	9.1	0.02
50-54	9.8	7.4	0.08	9.4	9	0.02
55-59	8.9	7.9	0.04	8.9	9.3	-0.02
60-64	6.2	7.8	-0.07	6.3	6.6	-0.01
65-69	4	7.7	-0.16	4.6	4.8	-0.01
70-74	3.7	8	-0.19	4.2	4.7	-0.02
75-79	2	5.2	-0.17	2.1	2.6	-0.03
80-84	1.3	4	-0.16	1.3	1.6	-0.02
85-89	0.9	2.5	-0.13	0.8	1.1	-0.03
90-94	0.4	1	-0.07	0.3	0.5	-0.02
95-99	0.1	0.2	-0.03	0.1	0.1	-0.01
**Sex**						
Female	56.4	57.4	-0.02	54.3	54.1	0

Table S6.35. *Continued.* Selected baseline characteristics for France IQVIA, for the long-term risk of any of psychiatric and neuropsychiatric disorders

Characteristic	Before propensity score matching			After propensity score matching		
	**Targets, %**	**Comparators, %**	**Standardized difference**	**Targets, %**	**Comparators, %**	**Standardized difference**
**Medical history (general)**						
Acute respiratory disease	16.7	6.7	0.32	15.3	18.4	-0.08
Chronic liver disease	0.1	0.1	0	0.1	0.1	-0.01
Chronic obstructive lung disease	0.9	0.7	0.02	0.7	1.1	-0.04
Crohn’s disease	0.1	0.1	0.01	0.1	0.2	-0.02
Diabetes mellitus	5.2	4	0.06	5.5	7	-0.06
Gastroesophageal reflux disease	4.3	2.1	0.12	3.6	4.3	-0.03
Gastrointestinal hemorrhage	0.5	0.3	0.04	0.4	0.5	-0.02
Human immunodeficiency virus infection	0.2	0.1	0.03	0.2	0.4	-0.04
Hyperlipidemia	4	2.9	0.06	4	4.7	-0.04
Hypertensive disorder	11.7	9.9	0.06	12.1	15.1	-0.09
Lesion of liver	0.1	0.1	0	0.1	0.1	-0.01
Obesity	0.4	0.1	0.05	0.3	0.2	0.01
Osteoarthritis	3.8	2.2	0.09	3.3	4.3	-0.05
Pneumonia	0.8	0.3	0.08	0.8	0.9	-0.01
Psoriasis	1	0.5	0.05	1	1	0
Renal impairment	0.3	0.2	0.02	0.3	0.4	-0.01
Rheumatoid arthritis	0.2	0.3	-0.01	0.2	0.4	-0.03
Ulcerative colitis	0.1	0.1	0.01	0.1	0.1	0
Urinary tract infectious disease	1.5	0.7	0.08	1.4	1.6	-0.02

Table S6.35. *Continued.* Selected baseline characteristics for France IQVIA, for the long-term risk of any of psychiatric and neuropsychiatric disorders

Characteristic	Before propensity score matching			After propensity score matching		
	Targets, %	Comparators, %	Standardized diff	Targets, %	Comparators, %	Standardized diff
**Medical history (cardiovascular disease)**						
Atrial fibrillation	0.1	0.3	-0.03	0.1	0.2	-0.01
Cerebrovascular disease	0.9	0.8	0.02	0.8	1.2	-0.03
Coronary arteriosclerosis	0.4	0.4	0	0.5	0.6	-0.01
Heart disease	3.3	3.6	-0.02	3.3	4.4	-0.06
Heart failure	0.3	0.3	0	0.2	0.4	-0.03
Ischemic heart disease	1	1	0	1.1	1.4	-0.03
Pulmonary embolism	0.2	0.1	0.01	0.2	0.3	-0.01
Venous thrombosis	0.3	0.2	0.03	0.3	0.4	-0.02
**Medical history (neoplasms)**						
Malignant neoplasm of anorectum	0.1	0.1	0.02	0.1	0.2	-0.01
Malignant neoplastic disease	1.1	0.8	0.03	1.1	1.4	-0.03
Malignant tumor of breast	0.3	0.2	0.02	0.3	0.3	0
Malignant tumor of colon	0.2	0.1	0.02	0.1	0.2	0
Primary malignant neoplasm of prostate	0.2	0.1	0.01	0.2	0.2	-0.01
**Medication use**						
Agents acting on the renin-angiotensin system	10.5	10	0.02	11.5	14.1	-0.08
Antibacterials for systemic use	36.9	16.6	0.47	33.8	38.1	-0.09
Antidepressants	7.3	4.7	0.11	1.6	2.2	-0.04
Antiepileptics	2.4	1.9	0.03	1.9	2.7	-0.05
Antiinflammatory and antirheumatic products	35.2	20.1	0.34	34.7	39.4	-0.1
Antineoplastic agents	1	0.8	0.02	1	1.1	-0.02
Antipsoriatics	0.5	0.3	0.03	0.5	0.6	-0.02
Antithrombotic agents	8	7.7	0.01	8.1	10.1	-0.07
Beta blocking agents	6.3	6.4	0	6.3	7.9	-0.06

Table S6.35. *Continued.* Selected baseline characteristics for France IQVIA, for the long-term risk of any of psychiatric and neuropsychiatric disorders

Characteristic	Before propensity score matching			After propensity score matching		
	**Targets, %**	**Comparators, %**	**Standardized difference**	**Targets, %**	**Comparators, %**	**Standardized difference**
**Medication use**						
Calcium channel blockers	5.8	5.3	0.02	6.2	7.9	-0.07
Diuretics	5.3	5.5	-0.01	5.6	7.2	-0.06
Drugs for acid-related disorders	24.5	14.3	0.26	22.6	26.2	-0.08
Drugs for obstructive airway diseases	23.1	12.8	0.27	22.2	25.7	-0.08
Drugs used in diabetes	5.5	4.3	0.05	5.8	7.2	-0.06
Immunosuppressants	0.3	0.6	-0.04	0.3	0.5	-0.03
Lipid modifying agents	8.2	8.1	0	8.7	10.7	-0.07
Opioids	58.5	23.3	0.77	53.9	55.8	-0.04
Psycholeptics	15	8.6	0.2	5.7	6.5	-0.04
Psychostimulants, agents used for ADHD and nootropics	4.5	2	0.14	4	4.4	-0.02

Table S6.36. Selected baseline characteristics for Germany IQVIA, for the short-term risk of depression

**Characteristic**	**Before propensity score matching**			**After propensity score matching**		
	**Targets, %**	**Comparators, %**	**Standardized difference**	**Targets, %**	**Comparators, %**	**Standardized difference**
**Age group (years)**						
0-4	1.4	2.4	-0.07	1.5	2.9	-0.09
5-9	2.2	3.2	-0.06	2.3	3.5	-0.07
10-14	2.8	3.3	-0.03	2.9	3.6	-0.04
15-19	5	3.6	0.07	5	5.9	-0.04
20-24	7.6	4	0.16	7.6	7.9	-0.01
25-29	7.4	4.3	0.13	7.3	7.2	0
30-34	8	5.2	0.11	7.9	7.7	0.01
35-39	7.6	5.2	0.1	7.6	7.4	0.01
40-44	8.3	5.4	0.12	8.2	7.8	0.02
45-49	8.1	5.9	0.09	8.2	7	0.04
50-54	9.9	8.3	0.05	10	9	0.04
55-59	9.6	9.4	0.01	9.6	8.9	0.03
60-64	7	8.8	-0.07	7.1	6.7	0.01
65-69	3.6	7.8	-0.18	3.7	3.3	0.02
70-74	2.9	6.9	-0.19	2.9	2.6	0.02
75-79	2.5	6.4	-0.19	2.5	2.5	0
80-84	2.7	6.2	-0.17	2.6	2.4	0.01
85-89	2	2.7	-0.05	1.9	2.2	-0.02
90-94	1.1	1	0.01	1	1.3	-0.03
95-99	0.3	0.2	0.04	0.3	0.3	0
**Sex**						
Female	53.8	57.1	-0.07	53.1	52.2	0.02

Table S6.36. *Continued.* Selected baseline characteristics for Germany IQVIA, for the short-term risk of depression

Characteristic	Before propensity score matching			After propensity score matching		
	Targets, %	Comparators, %	Standardized difference	Targets, %	Comparators, %	Standardized difference
**Medical history (general)**						
Acute respiratory disease	59	7.9	1.29	59.2	60.5	-0.03
Chronic liver disease	0.2	0.1	0.03	0.1	0.2	-0.01
Chronic obstructive lung disease	2.8	1.3	0.11	2.5	3.8	-0.08
Crohn’s disease	0.2	0.1	0.02	0.1	0.2	-0.02
Dementia	1.2	0.5	0.07	0.9	1.4	-0.04
Depressive disorder	7.2	2.8	0.2	0.5	0.1	0.08
Diabetes mellitus	4	2.3	0.1	3.7	4.8	-0.05
Gastroesophageal reflux disease	1.4	0.5	0.09	1.3	1.5	-0.01
Gastrointestinal hemorrhage	0.6	0.2	0.06	0.5	0.6	-0.01
Hyperlipidemia	5.5	2.5	0.16	5.1	6	-0.04
Hypertensive disorder	11.7	5.9	0.2	11	13.3	-0.07
Lesion of liver	0.2	0.1	0.01	0.1	0.2	-0.02
Obesity	2.6	1.1	0.11	2.3	2.2	0
Osteoarthritis	5.4	3.4	0.1	4.9	5.6	-0.03
Pneumonia	3	0.4	0.2	3	3.1	-0.01
Psoriasis	0.6	0.5	0.01	0.6	0.6	-0.01
Renal impairment	1.6	0.7	0.08	1.4	2	-0.05
Rheumatoid arthritis	0.6	0.4	0.03	0.6	0.8	-0.03
Schizophrenia	0.1	0.1	0.01	0.1	0.1	-0.01
Ulcerative colitis	0.2	0.1	0.02	0.1	0.3	-0.03
Urinary tract infectious disease	4.1	1.5	0.16	3.9	5.1	-0.06

Table S6.36. *Continued.* Selected baseline characteristics for Germany IQVIA, for the short-term risk of depression

Characteristic	Before propensity score matching			After propensity score matching		
	**Targets, %**	**Comparators, %**	**Standardized difference**	**Targets, %**	**Comparators, %**	**Standardized difference**
**Medical history (cardiovascular disease)**						
Atrial fibrillation	0.8	0.5	0.04	0.7	0.9	-0.03
Cerebrovascular disease	1.2	0.7	0.05	1.1	1.3	-0.02
Coronary arteriosclerosis	1	0.6	0.04	1	1.3	-0.03
Heart disease	7.7	3.9	0.16	7.2	8.5	-0.05
Heart failure	1.8	0.8	0.09	1.6	2	-0.04
Ischemic heart disease	2.4	1.3	0.08	2.2	2.7	-0.03
Peripheral vascular disease	0.9	0.6	0.04	0.8	1	-0.03
Pulmonary embolism	0.3	0.1	0.04	0.3	0.4	-0.03
Venous thrombosis	0.7	0.3	0.06	0.7	1	-0.03
**Medical history (neoplasms)**						
Malignant lymphoma	0.1	0.1	0.02	0.1	0.2	0
Malignant neoplastic disease	1.9	2.1	-0.01	1.8	2.2	-0.03
Malignant tumor of breast	0.3	0.4	-0.02	0.2	0.2	0
Malignant tumor of urinary bladder	0.1	0.1	-0.01	0.1	0.1	-0.01
Primary malignant neoplasm of prostate	0.2	0.3	-0.03	0.1	0.2	-0.01
**Medication use**						
Agents acting on the renin-angiotensin system	18.9	8.6	0.3	19	20.6	-0.04
Antibacterials for systemic use	26.6	9.2	0.47	26.8	32	-0.12
Antidepressants	6.2	3.6	0.12	4.2	5	-0.04
Antiepileptics	2.3	1.5	0.06	2.2	2.7	-0.03
Antiinflammatory and antirheumatic products	32.6	11.6	0.52	32.8	34.8	-0.04
Antineoplastic agents	1	0.9	0.01	1	1.2	-0.02
Antipsoriatics	0.2	0.3	0	0.2	0.3	0
Antithrombotic agents	10	4.6	0.2	9.9	11.8	-0.06
Beta blocking agents	12.1	6	0.21	12	13.4	-0.04

Table S6.36. *Continued.* Selected baseline characteristics for Germany IQVIA, for the short-term risk of depression

**Characteristic**	**Before propensity score matching**			**After propensity score matching**		
	**Targets, %**	**Comparators, %**	**Standardized difference**	**Targets, %**	**Comparators, %**	**Standardized difference**
**Medication use**						
Calcium channel blockers	8.4	3.9	0.19	8.4	9.2	-0.03
Diuretics	11	5.3	0.21	10.8	12.5	-0.05
Drugs for acid-related disorders	20.1	6.8	0.4	19.5	22	-0.06
Drugs for obstructive airway diseases	15.1	7.2	0.25	15.2	19.5	-0.11
Drugs used in diabetes	5.8	3	0.13	5.6	6.2	-0.02
Immunosuppressants	0.6	0.7	-0.02	0.5	0.7	-0.02
Lipid modifying agents	9.4	4.7	0.18	9.3	10.8	-0.05
Opioids	8.8	2.6	0.27	8.6	9.9	-0.04
Psycholeptics	5.1	2.8	0.12	4.5	5.6	-0.05
Psychostimulants, agents used for ADHD and nootropics	0.3	0.2	0.01	0.2	0.3	-0.01

Table S6.37. Selected baseline characteristics for Germany IQVIA, for the short-term risk of anxiety disorders

Characteristic	Before propensity score matching			After propensity score matching		
	**Targets, %**	**Comparators, %**	**Standardized difference**	**Targets, %**	**Comparators, %**	**Standardized difference**
**Age group (years)**						
0-4	1.4	2.4	-0.07	1.5	2.8	-0.09
5-9	2.2	3.3	-0.07	2.3	3.4	-0.07
10-14	2.8	3.2	-0.02	2.8	3.4	-0.04
15-19	5	3.4	0.08	5	5.8	-0.04
20-24	7.6	4	0.15	7.6	7.7	-0.01
25-29	7.4	4.2	0.14	7.3	7.1	0.01
30-34	8	5.2	0.11	7.7	7.6	0
35-39	7.6	5.3	0.1	7.5	7.3	0.01
40-44	8.3	5.5	0.11	8.3	7.7	0.02
45-49	8.1	6	0.08	8.1	7.1	0.04
50-54	9.9	8.3	0.06	9.9	9.1	0.03
55-59	9.6	9.3	0.01	9.6	9	0.02
60-64	7	8.7	-0.06	7.1	6.8	0.01
65-69	3.6	7.9	-0.18	3.7	3.4	0.01
70-74	2.9	6.8	-0.18	2.9	2.7	0.02
75-79	2.5	6.3	-0.18	2.6	2.6	0
80-84	2.7	6.3	-0.17	2.7	2.5	0.01
85-89	2	2.7	-0.05	2	2.3	-0.02
90-94	1.1	1	0.01	1.1	1.4	-0.03
95-99	0.3	0.2	0.03	0.3	0.3	0
**Sex**						
Female	53.8	57.2	-0.07	53.4	52.4	0.02

Table S6.37. *Continued.* Selected baseline characteristics for Germany IQVIA, for the short-term risk of anxiety disorders

Characteristic	Before propensity score matching			After propensity score matching		
	**Targets, %**	**Comparators, %**	**Standardized difference**	**Targets, %**	**Comparators, %**	**Standardized difference**
**Medical history (general)**						
Acute respiratory disease	59	8	1.28	59.2	60.6	-0.03
Chronic liver disease	0.2	0.1	0.02	0.2	0.2	-0.02
Chronic obstructive lung disease	2.8	1.3	0.11	2.9	4.1	-0.07
Crohn’s disease	0.2	0.1	0.02	0.2	0.2	-0.02
Dementia	1.2	0.5	0.08	1.2	1.6	-0.04
Depressive disorder	7.2	2.8	0.2	6.2	6	0.01
Diabetes mellitus	4	2.2	0.1	4.1	5.1	-0.05
Gastroesophageal reflux disease	1.4	0.5	0.1	1.4	1.5	-0.01
Gastrointestinal hemorrhage	0.6	0.2	0.06	0.6	0.6	-0.01
Hyperlipidemia	5.5	2.4	0.16	5.5	6.5	-0.04
Hypertensive disorder	11.7	5.9	0.2	11.7	14.1	-0.07
Lesion of liver	0.2	0.1	0.01	0.2	0.3	-0.02
Obesity	2.6	1.1	0.11	2.5	2.5	0
Osteoarthritis	5.4	3.5	0.09	5.3	5.9	-0.03
Pneumonia	3	0.4	0.2	3	3.2	-0.01
Psoriasis	0.6	0.5	0.02	0.6	0.7	-0.01
Renal impairment	1.6	0.7	0.08	1.6	2.2	-0.04
Rheumatoid arthritis	0.6	0.4	0.03	0.6	0.8	-0.02
Schizophrenia	0.1	0.1	0.01	0.1	0.2	-0.01
Ulcerative colitis	0.2	0.1	0.02	0.1	0.3	-0.03
Urinary tract infectious disease	4.1	1.5	0.16	4.1	5.2	-0.06

Table S6.37. *Continued.* Selected baseline characteristics for Germany IQVIA, for the short-term risk of anxiety disorders

Characteristic	Before propensity score matching			After propensity score matching		
	Targets, %	Comparators, %	Standardized difference	Targets, %	Comparators, %	Standardized difference
**Medical history (cardiovascular disease)**						
Atrial fibrillation	0.8	0.5	0.03	0.8	1	-0.03
Cerebrovascular disease	1.2	0.7	0.05	1.2	1.4	-0.02
Coronary arteriosclerosis	1	0.6	0.04	1	1.4	-0.03
Heart disease	7.7	4	0.16	7.6	9.1	-0.05
Heart failure	1.8	0.9	0.08	1.8	2.3	-0.03
Ischemic heart disease	2.4	1.2	0.09	2.4	2.9	-0.04
Peripheral vascular disease	0.9	0.5	0.04	0.8	1.1	-0.03
Pulmonary embolism	0.3	0.1	0.04	0.3	0.5	-0.03
Venous thrombosis	0.7	0.3	0.06	0.7	1	-0.03
**Medical history (neoplasms)**						
Malignant lymphoma	0.1	0.1	0.02	0.1	0.2	0
Malignant neoplastic disease	1.9	2.1	-0.01	1.9	2.4	-0.03
Malignant tumor of breast	0.3	0.4	-0.02	0.3	0.3	0
Malignant tumor of colon	0.1	0.1	0	0.1	0.1	-0.01
Malignant tumor of urinary bladder	0.1	0.1	-0.01	0.1	0.1	-0.01
Primary malignant neoplasm of prostate	0.2	0.3	-0.03	0.1	0.2	-0.01
**Medication use**						
Agents acting on the renin-angiotensin system	18.9	8.7	0.3	19.3	21.3	-0.05
Antibacterials for systemic use	26.6	9.2	0.47	26.9	32.3	-0.12
Antidepressants	6.2	3.6	0.12	5.5	6.7	-0.05
Antiepileptics	2.3	1.5	0.06	2.3	2.9	-0.04
Antiinflammatory and antirheumatic products	32.6	11.6	0.52	33.5	35.1	-0.03
Antineoplastic agents	1	0.9	0.01	1	1.2	-0.02
Antithrombotic agents	10	4.6	0.21	10.2	12.2	-0.06
Beta blocking agents	12.1	6	0.21	12.4	13.8	-0.04

Table S6.37. *Continued.* Selected baseline characteristics for Germany IQVIA, for the short-term risk of anxiety disorders

Characteristic	Before propensity score matching			After propensity score matching		
	**Targets, %**	**Comparators, %**	**Standardized difference**	**Targets, %**	**Comparators, %**	**Standardized difference**
**Medication use**						
Calcium channel blockers	8.4	3.9	0.19	8.7	9.5	-0.03
Diuretics	11	5.2	0.21	11.3	13.1	-0.06
Drugs for acid-related disorders	20.1	6.9	0.39	20.3	22.8	-0.06
Drugs for obstructive airway diseases	15.1	7.2	0.26	15.4	19.7	-0.11
Drugs used in diabetes	5.8	3	0.14	5.9	6.4	-0.02
Immunosuppressants	0.6	0.7	-0.02	0.6	0.7	-0.02
Lipid modifying agents	9.4	4.7	0.18	9.6	11.2	-0.05
Opioids	8.8	2.6	0.27	9	10.4	-0.04
Psycholeptics	5.1	2.7	0.12	4.8	6	-0.06
Psychostimulants, agents used for ADHD and nootropics	0.3	0.2	0.01	0.3	0.3	0

Table S6.38. Selected baseline characteristics for Germany IQVIA, for the short-term risk of alcohol misuse or dependence

Characteristic	Before propensity score matching			After propensity score matching		
	Targets, %	Comparators, %	Standardized difference	Targets, %	Comparators, %	Standardized difference
**Age group (years)**						
0-4	1.4	2.3	-0.07	1.4	2.7	-0.09
5-9	2.2	3.3	-0.07	2.2	3.2	-0.06
10-14	2.8	3.3	-0.03	2.7	3.3	-0.04
15-19	5	3.5	0.07	4.9	5.7	-0.04
20-24	7.6	4.1	0.15	7.6	7.7	-0.01
25-29	7.4	4.3	0.13	7.3	7.1	0.01
30-34	8	5.1	0.12	7.8	7.6	0.01
35-39	7.6	5.3	0.09	7.5	7.4	0.01
40-44	8.3	5.4	0.11	8.3	7.8	0.02
45-49	8.1	6	0.08	8.2	7.2	0.04
50-54	9.9	8.2	0.06	10	9.2	0.03
55-59	9.6	9.5	0	9.7	9.1	0.02
60-64	7	8.7	-0.06	7.1	6.9	0.01
65-69	3.6	7.9	-0.18	3.6	3.4	0.01
70-74	2.9	6.8	-0.18	2.9	2.6	0.02
75-79	2.5	6.4	-0.19	2.5	2.6	0
80-84	2.7	6.1	-0.17	2.7	2.5	0.01
85-89	2	2.7	-0.05	2	2.3	-0.02
90-94	1.1	1	0.01	1.1	1.4	-0.03
95-99	0.3	0.2	0.04	0.3	0.3	0
**Sex**						
Female	53.8	57	-0.06	53.9	53.2	0.01

Table S6.38. *Continued.* Selected baseline characteristics for Germany IQVIA, for the short-term risk of alcohol misuse or dependence

Characteristic	Before propensity score matching			After propensity score matching		
	Targets, %	Comparators, %	Standardized difference	Targets, %	Comparators, %	Standardized difference
**Medical history (general)**						
Acute respiratory disease	59	7.7	1.29	59.6	60.8	-0.03
Chronic liver disease	0.2	0.1	0.03	0.2	0.2	-0.01
Chronic obstructive lung disease	2.8	1.2	0.11	2.9	4.2	-0.07
Crohn's disease	0.2	0.1	0.02	0.2	0.2	-0.02
Dementia	1.2	0.5	0.08	1.2	1.7	-0.04
Depressive disorder	7.2	2.8	0.2	7.3	7.6	-0.01
Diabetes mellitus	4	2.3	0.1	4.2	5.2	-0.05
Gastroesophageal reflux disease	1.4	0.5	0.1	1.4	1.6	-0.01
Gastrointestinal hemorrhage	0.6	0.2	0.06	0.6	0.7	-0.01
Hyperlipidemia	5.5	2.4	0.16	5.7	6.7	-0.04
Hypertensive disorder	11.7	6	0.2	12	14.5	-0.07
Lesion of liver	0.2	0.1	0.01	0.1	0.3	-0.03
Obesity	2.6	1.1	0.11	2.6	2.6	0
Osteoarthritis	5.4	3.4	0.1	5.5	6.3	-0.03
Pneumonia	3	0.4	0.2	3	3.3	-0.01
Psoriasis	0.6	0.5	0.01	0.6	0.7	0
Renal impairment	1.6	0.7	0.08	1.6	2.2	-0.05
Rheumatoid arthritis	0.6	0.4	0.02	0.6	0.8	-0.02
Schizophrenia	0.1	0.1	0.01	0.1	0.2	-0.01
Ulcerative colitis	0.2	0.1	0.01	0.2	0.3	-0.03
Urinary tract infectious disease	4.1	1.5	0.16	4.2	5.4	-0.06

Table S6.38. *Continued.* Selected baseline characteristics for Germany IQVIA, for the short-term risk of alcohol misuse or dependence

**Characteristic**	**Before propensity score matching**			**After propensity score matching**		
	**Targets, %**	**Comparators, %**	**Standardized difference**	**Targets, %**	**Comparators, %**	**Standardized difference**
**Medical history (cardiovascular disease)**						
Atrial fibrillation	0.8	0.5	0.03	0.8	1	-0.03
Cerebrovascular disease	1.2	0.7	0.05	1.2	1.6	-0.03
Coronary arteriosclerosis	1	0.6	0.04	1.1	1.4	-0.03
Heart disease	7.7	4	0.16	7.9	9.5	-0.06
Heart failure	1.8	0.9	0.08	1.8	2.3	-0.04
Ischemic heart disease	2.4	1.3	0.08	2.5	3	-0.04
Peripheral vascular disease	0.9	0.5	0.04	0.9	1.2	-0.03
Pulmonary embolism	0.3	0.1	0.04	0.3	0.5	-0.03
Venous thrombosis	0.7	0.3	0.06	0.7	1	-0.03
**Medical history (neoplasms)**						
Malignant lymphoma	0.1	0.1	0.02	0.2	0.2	0
Malignant neoplastic disease	1.9	2.1	-0.01	2	2.5	-0.03
Malignant tumor of breast	0.3	0.4	-0.02	0.3	0.3	0
Malignant tumor of urinary bladder	0.1	0.1	-0.01	0.1	0.1	0
Primary malignant neoplasm of prostate	0.2	0.3	-0.03	0.2	0.2	-0.01
**Medication use**						
Agents acting on the renin-angiotensin system	18.9	8.7	0.3	19.6	21.5	-0.05
Antibacterials for systemic use	26.6	9.1	0.47	27.4	32.7	-0.12
Antidepressants	6.2	3.5	0.12	6.4	8	-0.06
Antiepileptics	2.3	1.4	0.06	2.4	3	-0.04
Antiinflammatory and antirheumatic products	32.6	11.6	0.52	33.7	35.3	-0.04
Antineoplastic agents	1	0.9	0.01	1	1.2	-0.02
Antipsoriatics	0.2	0.3	-0.01	0.3	0.3	0
Antithrombotic agents	10	4.6	0.21	10.3	12.4	-0.07
Beta blocking agents	12.1	6	0.21	12.5	14.1	-0.05

Table S6.38. *Continued.* Selected baseline characteristics for Germany IQVIA, for the short-term risk of alcohol misuse or dependence

Characteristic	Before propensity score matching			After propensity score matching		
	**Targets, %**	**Comparators, %**	**Standardized difference**	**Targets, %**	**Comparators, %**	**Standardized difference**
**Medication use**						
Calcium channel blockers	8.4	3.9	0.19	8.7	9.6	-0.03
Diuretics	11	5.2	0.21	11.3	13.2	-0.06
Drugs for acid-related disorders	20.1	6.8	0.4	20.7	23.3	-0.06
Drugs for obstructive airway diseases	15.1	7.2	0.25	15.6	20	-0.11
Drugs used in diabetes	5.8	3	0.14	6	6.5	-0.02
Immunosuppressants	0.6	0.7	-0.02	0.6	0.7	-0.01
Lipid modifying agents	9.4	4.7	0.18	9.7	11.3	-0.05
Opioids	8.8	2.6	0.27	9.1	10.6	-0.05
Psycholeptics	5.1	2.7	0.12	5.3	6.8	-0.06
Psychostimulants, agents used for ADHD and nootropics	0.3	0.2	0.01	0.3	0.3	-0.01

Table S6.39. Selected baseline characteristics for Germany IQVIA, for the short-term risk of substance misuse or dependence

Characteristic	Before propensity score matching			After propensity score matching		
	Targets, %	Comparators, %	Standardized difference	Targets, %	Comparators, %	Standardized difference
**Age group (years)**						
0-4	1.4	2.4	-0.07	1.4	2.7	-0.09
5-9	2.2	3.2	-0.06	2.2	3.3	-0.07
10-14	2.8	3.3	-0.03	2.8	3.4	-0.04
15-19	5	3.5	0.07	4.9	5.7	-0.04
20-24	7.6	4	0.16	7.6	7.8	-0.01
25-29	7.4	4.2	0.13	7.3	7.2	0.01
30-34	8	5.1	0.12	7.9	7.6	0.01
35-39	7.6	5.3	0.09	7.5	7.4	0.01
40-44	8.3	5.5	0.11	8.2	7.8	0.02
45-49	8.1	6	0.08	8.2	7.1	0.04
50-54	9.9	8.3	0.05	10	9.1	0.03
55-59	9.6	9.4	0.01	9.7	9	0.03
60-64	7	8.7	-0.06	7.1	6.8	0.01
65-69	3.6	7.7	-0.18	3.6	3.4	0.01
70-74	2.9	6.9	-0.19	2.9	2.6	0.02
75-79	2.5	6.3	-0.19	2.5	2.6	0
80-84	2.7	6.2	-0.17	2.7	2.5	0.01
85-89	2	2.7	-0.05	2	2.3	-0.02
90-94	1.1	1	0.01	1.1	1.4	-0.03
95-99	0.3	0.2	0.03	0.3	0.3	0
**Sex**						
Female	53.8	57.1	-0.07	54.1	53.4	0.01

Table S6.39. *Continued.* Selected baseline characteristics for Germany IQVIA, for the short-term risk of substance misuse or dependence

Characteristic	Before propensity score matching			After propensity score matching		
	Targets, %	Comparators, %	Standardized difference	Targets, %	Comparators, %	Standardized difference
**Medical history (general)**						
Acute respiratory disease	59	7.9	1.29	59.6	60.8	-0.02
Chronic liver disease	0.2	0.1	0.03	0.1	0.2	-0.01
Chronic obstructive lung disease	2.8	1.3	0.11	2.8	3.9	-0.06
Crohn’s disease	0.2	0.1	0.02	0.2	0.2	-0.01
Dementia	1.2	0.5	0.07	1.2	1.7	-0.04
Depressive disorder	7.2	2.7	0.21	7.2	7.4	-0.01
Diabetes mellitus	4	2.2	0.11	4.1	5.1	-0.05
Gastroesophageal reflux disease	1.4	0.5	0.09	1.4	1.6	-0.02
Gastrointestinal hemorrhage	0.6	0.2	0.06	0.6	0.6	-0.01
Hyperlipidemia	5.5	2.4	0.16	5.5	6.5	-0.04
Hypertensive disorder	11.7	5.9	0.2	11.9	14.3	-0.07
Lesion of liver	0.2	0.1	0.01	0.1	0.2	-0.02
Obesity	2.6	1.1	0.11	2.5	2.5	0
Osteoarthritis	5.4	3.5	0.09	5.5	6.1	-0.03
Pneumonia	3	0.4	0.2	3	3.2	-0.01
Psoriasis	0.6	0.5	0.01	0.6	0.7	0
Renal impairment	1.6	0.7	0.08	1.5	2.2	-0.04
Rheumatoid arthritis	0.6	0.4	0.03	0.6	0.8	-0.02
Schizophrenia	0.1	0.1	0	0.1	0.2	-0.01
Ulcerative colitis	0.2	0.1	0.02	0.2	0.3	-0.03
Urinary tract infectious disease	4.1	1.5	0.16	4.2	5.4	-0.06

Table S6.39. *Continued.* Selected baseline characteristics for Germany IQVIA, for the short-term risk of substance misuse or dependence

Characteristic	Before propensity score matching			After propensity score matching		
	Targets, %	Comparators, %	Standardized difference	Targets, %	Comparators, %	Standardized difference
**Medical history (cardiovascular disease)**						
Atrial fibrillation	0.8	0.5	0.03	0.8	1	-0.03
Cerebrovascular disease	1.2	0.7	0.05	1.2	1.5	-0.03
Coronary arteriosclerosis	1	0.6	0.04	1	1.4	-0.03
Heart disease	7.7	4	0.16	7.8	9.2	-0.05
Heart failure	1.8	0.9	0.08	1.8	2.3	-0.04
Ischemic heart disease	2.4	1.3	0.09	2.4	2.9	-0.03
Peripheral vascular disease	0.9	0.5	0.04	0.8	1.1	-0.03
Pulmonary embolism	0.3	0.1	0.04	0.3	0.5	-0.03
Venous thrombosis	0.7	0.3	0.06	0.7	1	-0.03
**Medical history (neoplasms)**						
Malignant lymphoma	0.1	0.1	0.02	0.2	0.2	0
Malignant neoplastic disease	1.9	2.1	-0.01	2	2.4	-0.03
Malignant tumor of breast	0.3	0.4	-0.02	0.3	0.3	0
Malignant tumor of colon	0.1	0.1	0	0.1	0.1	-0.01
Malignant tumor of urinary bladder	0.1	0.1	0	0.1	0.1	0
Primary malignant neoplasm of prostate	0.2	0.3	-0.03	0.1	0.2	-0.01
**Medication use**						
Agents acting on the renin-angiotensin system	18.9	8.6	0.3	19.6	21.4	-0.05
Antibacterials for systemic use	26.6	9.3	0.46	27.4	32.6	-0.11
Antidepressants	6.2	3.5	0.13	6.3	7.9	-0.06
Antiepileptics	2.3	1.5	0.06	2.3	2.9	-0.04
Antiinflammatory and antirheumatic products	32.6	11.7	0.52	33.6	35.2	-0.03
Antineoplastic agents	1	0.9	0.01	1	1.2	-0.02
Antipsoriatics	0.2	0.3	0	0.3	0.3	0
Antithrombotic agents	10	4.6	0.21	10.3	12.3	-0.06

Table S6.39. *Continued.* Selected baseline characteristics for Germany IQVIA, for the short-term risk of substance misuse or dependence

**Characteristic**	**Before propensity score matching**			**After propensity score matching**		
	**Targets, %**	**Comparators, %**	**Standardized difference**	**Targets, %**	**Comparators, %**	**Standardized difference**
**Medication use**						
Beta blocking agents	12.1	5.9	0.22	12.5	14	-0.04
Calcium channel blockers	8.4	3.8	0.19	8.7	9.6	-0.03
Diuretics	11	5.2	0.21	11.3	13.1	-0.06
Drugs for acid-related disorders	20.1	6.7	0.4	20.6	23.1	-0.06
Drugs for obstructive airway diseases	15.1	7.3	0.25	15.6	19.8	-0.11
Drugs used in diabetes	5.8	3	0.14	5.9	6.4	-0.02
Immunosuppressants	0.6	0.8	-0.03	0.6	0.7	-0.01
Lipid modifying agents	9.4	4.6	0.19	9.7	11.2	-0.05
Opioids	8.8	2.6	0.27	9.1	10.5	-0.05
Psycholeptics	5.1	2.8	0.12	5.2	6.6	-0.06
Psychostimulants, agents used for ADHD and nootropics	0.3	0.2	0.01	0.3	0.3	-0.01

Table S6.40. Selected baseline characteristics for Germany IQVIA, for the short-term risk of bipolar disorders

Characteristic	Before propensity score matching			After propensity score matching		
	Targets, %	Comparators, %	Standardized difference	Targets, %	Comparators, %	Standardized difference
**Age group (years)**						
0-4	1.4	2.4	-0.07	1.4	2.7	-0.09
5-9	2.2	3.3	-0.06	2.2	3.2	-0.06
10-14	2.8	3.2	-0.02	2.7	3.3	-0.04
15-19	5	3.6	0.07	4.9	5.7	-0.04
20-24	7.6	3.9	0.16	7.6	7.7	-0.01
25-29	7.4	4.3	0.13	7.3	7.1	0.01
30-34	8	5.2	0.11	7.8	7.6	0.01
35-39	7.6	5.3	0.1	7.5	7.4	0.01
40-44	8.3	5.5	0.11	8.3	7.8	0.02
45-49	8.1	5.9	0.09	8.2	7.2	0.04
50-54	9.9	8.4	0.05	10	9.2	0.03
55-59	9.6	9.4	0.01	9.7	9.1	0.02
60-64	7	8.6	-0.06	7.2	6.9	0.01
65-69	3.6	7.9	-0.18	3.6	3.4	0.01
70-74	2.9	6.9	-0.19	2.9	2.6	0.02
75-79	2.5	6.3	-0.18	2.6	2.6	0
80-84	2.7	6.2	-0.17	2.7	2.5	0.01
85-89	2	2.7	-0.05	2	2.3	-0.02
90-94	1.1	1	0.01	1.1	1.4	-0.03
95-99	0.3	0.2	0.03	0.3	0.3	0
**Sex**						
Female	53.8	57.1	-0.07	53.9	53.1	0.01

Table S6.40. *Continued.* Selected baseline characteristics for Germany IQVIA, for the short-term risk of bipolar disorders

Characteristic	Before propensity score matching			After propensity score matching		
	Targets, %	Comparators, %	Standardized difference	Targets, %	Comparators, %	Standardized difference
**Medical history (general)**						
Acute respiratory disease	59	7.8	1.29	59.6	60.8	-0.03
Chronic liver disease	0.2	0.1	0.03	0.2	0.2	-0.02
Chronic obstructive lung disease	2.8	1.3	0.11	2.9	4.2	-0.07
Crohn’s disease	0.2	0.1	0.02	0.2	0.2	-0.02
Dementia	1.2	0.5	0.07	1.2	1.8	-0.04
Depressive disorder	7.2	2.8	0.2	7.3	7.7	-0.01
Diabetes mellitus	4	2.3	0.1	4.2	5.3	-0.05
Gastroesophageal reflux disease	1.4	0.5	0.1	1.4	1.6	-0.01
Gastrointestinal hemorrhage	0.6	0.2	0.06	0.6	0.7	-0.01
Hyperlipidemia	5.5	2.4	0.16	5.7	6.7	-0.04
Hypertensive disorder	11.7	5.9	0.2	12.1	14.6	-0.07
Lesion of liver	0.2	0.1	0.01	0.2	0.3	-0.02
Osteoarthritis	5.4	3.4	0.1	5.5	6.3	-0.03
Pneumonia	3	0.4	0.2	3	3.3	-0.01
Psoriasis	0.6	0.5	0.01	0.6	0.7	0
Renal impairment	1.6	0.8	0.08	1.6	2.2	-0.05
Rheumatoid arthritis	0.6	0.4	0.02	0.6	0.8	-0.02
Schizophrenia	0.1	0.1	0.01	0.1	0.2	-0.01
Ulcerative colitis	0.2	0.1	0.02	0.2	0.3	-0.03
Urinary tract infectious disease	4.1	1.5	0.16	4.2	5.4	-0.06

Table S6.40. *Continued.* Selected baseline characteristics for Germany IQVIA, for the short-term risk of bipolar disorders

**Characteristic**	**Before propensity score matching**			**After propensity score matching**		
	**Targets, %**	**Comparators, %**	**Standardized difference**	**Targets, %**	**Comparators, %**	**Standardized difference**
**Medical history (cardiovascular disease)**						
Atrial fibrillation	0.8	0.5	0.03	0.8	1	-0.03
Cerebrovascular disease	1.2	0.7	0.05	1.2	1.6	-0.03
Coronary arteriosclerosis	1	0.6	0.05	1.1	1.4	-0.03
Heart disease	7.7	3.9	0.16	7.9	9.5	-0.06
Heart failure	1.8	0.8	0.09	1.9	2.4	-0.04
Ischemic heart disease	2.4	1.3	0.08	2.5	3	-0.03
Peripheral vascular disease	0.9	0.6	0.03	0.9	1.2	-0.03
Pulmonary embolism	0.3	0.1	0.04	0.3	0.5	-0.03
Venous thrombosis	0.7	0.3	0.06	0.7	1	-0.03
**Medical history (neoplasms)**						
Malignant lymphoma	0.1	0.1	0.02	0.2	0.2	-0.01
Malignant neoplastic disease	1.9	2.1	-0.01	2	2.5	-0.03
Malignant tumor of breast	0.3	0.4	-0.02	0.3	0.3	0
Malignant tumor of colon	0.1	0.1	0	0.1	0.1	-0.01
Malignant tumor of urinary bladder	0.1	0.1	0	0.1	0.1	0
Primary malignant neoplasm of prostate	0.2	0.3	-0.03	0.2	0.2	-0.01
**Medication use**						
Agents acting on the renin-angiotensin system	18.9	8.7	0.3	19.6	21.6	-0.05
Antibacterials for systemic use	26.6	9.3	0.46	27.4	32.7	-0.12
Antidepressants	6.2	3.6	0.12	6.4	8	-0.06
Antiepileptics	2.3	1.5	0.06	2.4	3	-0.04
Antiinflammatory and antirheumatic products	32.6	11.7	0.52	33.7	35.3	-0.04
Antineoplastic agents	1	0.9	0.01	1	1.2	-0.02
Antipsoriatics	0.2	0.3	0	0.3	0.3	0
Antithrombotic agents	10	4.6	0.21	10.3	12.4	-0.07

Table S6.40. *Continued.* Selected baseline characteristics for Germany IQVIA, for the short-term risk of bipolar disorders

**Characteristic**	**Before propensity score matching**			**After propensity score matching**		
	**Targets, %**	**Comparators, %**	**Standardized difference**	**Targets, %**	**Comparators, %**	**Standardized difference**
**Medication use**						
Beta blocking agents	12.1	5.9	0.22	12.5	14.1	-0.05
Calcium channel blockers	8.4	3.9	0.19	8.8	9.7	-0.03
Diuretics	11	5.2	0.21	11.4	13.3	-0.06
Drugs for acid-related disorders	20.1	6.8	0.4	20.8	23.4	-0.06
Drugs for obstructive airway diseases	15.1	7.3	0.25	15.7	20	-0.11
Drugs used in diabetes	5.8	3.1	0.13	6	6.5	-0.02
Immunosuppressants	0.6	0.7	-0.02	0.6	0.7	-0.01
Lipid modifying agents	9.4	4.7	0.18	9.7	11.3	-0.05
Opioids	8.8	2.7	0.27	9.1	10.6	-0.05
Psycholeptics	5.1	2.8	0.12	5.3	6.8	-0.06
Psychostimulants, agents used for ADHD and nootropics	0.3	0.2	0.01	0.3	0.3	0

Table S6.41. Selected baseline characteristics for Germany IQVIA, for the short-term risk of psychoses

Characteristic	Before propensity score matching			After propensity score matching		
	Targets, %	Comparators, %	Standardized difference	Targets, %	Comparators, %	Standardized difference
**Age group (years)**						
0-4	1.4	2.3	-0.07	1.4	2.7	-0.09
5-9	2.2	3.2	-0.06	2.2	3.2	-0.06
10-14	2.8	3.3	-0.03	2.7	3.3	-0.04
15-19	5	3.5	0.07	4.9	5.7	-0.04
20-24	7.6	4	0.16	7.6	7.7	-0.01
25-29	7.4	4.3	0.13	7.3	7.1	0.01
30-34	8	5.2	0.11	7.8	7.6	0.01
35-39	7.6	5.3	0.1	7.6	7.4	0.01
40-44	8.3	5.5	0.11	8.3	7.8	0.02
45-49	8.1	6	0.08	8.2	7.2	0.04
50-54	9.9	8.3	0.05	10	9.2	0.03
55-59	9.6	9.4	0.01	9.7	9.1	0.02
60-64	7	8.7	-0.06	7.1	6.9	0.01
65-69	3.6	7.8	-0.18	3.6	3.4	0.01
70-74	2.9	6.8	-0.18	2.9	2.6	0.02
75-79	2.5	6.3	-0.18	2.5	2.6	0
80-84	2.7	6.2	-0.17	2.7	2.5	0.01
85-89	2	2.7	-0.05	2	2.3	-0.02
90-94	1.1	1	0.02	1.1	1.4	-0.03
95-99	0.3	0.2	0.03	0.3	0.3	0
**Sex**						
Female	53.8	57.1	-0.07	53.9	53.1	0.01

Table S6.41. *Continued.* Selected baseline characteristics for Germany IQVIA, for the short-term risk of psychoses

Characteristic	Before propensity score matching			After propensity score matching		
	**Targets, %**	**Comparators, %**	**Standardized difference**	**Targets, %**	**Comparators, %**	**Standardized difference**
**Medical history (general)**						
Acute respiratory disease	59	7.8	1.29	59.6	60.9	-0.03
Chronic liver disease	0.2	0.1	0.03	0.2	0.2	-0.02
Chronic obstructive lung disease	2.8	1.2	0.11	2.9	4.2	-0.07
Crohn's disease	0.2	0.1	0.02	0.2	0.2	-0.02
Dementia	1.2	0.5	0.08	1.2	1.6	-0.04
Depressive disorder	7.2	2.7	0.2	7.3	7.6	-0.01
Diabetes mellitus	4	2.3	0.1	4.1	5.2	-0.05
Gastroesophageal reflux disease	1.4	0.5	0.1	1.4	1.6	-0.01
Gastrointestinal hemorrhage	0.6	0.2	0.06	0.6	0.7	-0.01
Hyperlipidemia	5.5	2.4	0.16	5.7	6.7	-0.04
Hypertensive disorder	11.7	5.9	0.2	12	14.5	-0.07
Lesion of liver	0.2	0.1	0.01	0.2	0.3	-0.02
Obesity	2.6	1.1	0.11	2.6	2.6	0
Osteoarthritis	5.4	3.4	0.1	5.5	6.2	-0.03
Pneumonia	3	0.4	0.2	3	3.2	-0.01
Psoriasis	0.6	0.5	0.01	0.6	0.7	-0.01
Renal impairment	1.6	0.7	0.08	1.6	2.2	-0.05
Rheumatoid arthritis	0.6	0.5	0.02	0.6	0.8	-0.02
Ulcerative colitis	0.2	0.1	0.02	0.2	0.3	-0.03
Urinary tract infectious disease	4.1	1.5	0.16	4.2	5.4	-0.06

Table S6.41. *Continued.* Selected baseline characteristics for Germany IQVIA, for the short-term risk of psychoses

Characteristic	Before propensity score matching			After propensity score matching		
	Targets, %	Comparators, %	Standardized difference	Targets, %	Comparators, %	Standardized difference
**Medical history (cardiovascular disease)**						
Atrial fibrillation	0.8	0.5	0.04	0.8	1	-0.02
Cerebrovascular disease	1.2	0.7	0.05	1.2	1.5	-0.03
Coronary arteriosclerosis	1	0.7	0.04	1.1	1.4	-0.03
Heart disease	7.7	4	0.16	7.9	9.4	-0.06
Heart failure	1.8	0.9	0.08	1.8	2.3	-0.04
Ischemic heart disease	2.4	1.3	0.09	2.4	3	-0.03
Peripheral vascular disease	0.9	0.6	0.04	0.9	1.2	-0.03
Pulmonary embolism	0.3	0.1	0.04	0.3	0.5	-0.03
Venous thrombosis	0.7	0.3	0.06	0.7	1	-0.03
**Medical history (neoplasms)**						
Malignant lymphoma	0.1	0.1	0.02	0.2	0.2	0
Malignant neoplastic disease	1.9	2.1	-0.01	2	2.5	-0.03
Malignant tumor of breast	0.3	0.4	-0.01	0.3	0.3	0
Malignant tumor of colon	0.1	0.1	0	0.1	0.1	-0.01
Malignant tumor of urinary bladder	0.1	0.1	-0.01	0.1	0.1	0
Primary malignant neoplasm of prostate	0.2	0.3	-0.03	0.2	0.2	-0.01
**Medication use**						
Agents acting on the renin-angiotensin system	18.9	8.6	0.3	19.6	21.5	-0.05
Antibacterials for systemic use	26.6	9.2	0.46	27.4	32.7	-0.12
Antidepressants	6.2	3.5	0.13	6.4	8	-0.06
Antiepileptics	2.3	1.5	0.06	2.4	3	-0.04
Antiinflammatory and antirheumatic products	32.6	11.7	0.52	33.6	35.3	-0.04
Antineoplastic agents	1	0.9	0.01	1	1.2	-0.02
Antipsoriatics	0.2	0.3	-0.01	0.2	0.3	0
Antithrombotic agents	10	4.7	0.2	10.3	12.3	-0.06

Table S6.41. *Continued.* Selected baseline characteristics for Germany IQVIA, for the short-term risk of psychoses

Characteristic	Before propensity score matching			After propensity score matching		
	Targets, %	Comparators, %	Standardized difference	Targets, %	Comparators, %	Standardized difference
**Medication use**						
Beta blocking agents	12.1	5.9	0.22	12.5	14.1	-0.05
Calcium channel blockers	8.4	3.8	0.19	8.8	9.6	-0.03
Diuretics	11	5.2	0.21	11.3	13.2	-0.06
Drugs for acid-related disorders	20.1	6.8	0.4	20.7	23.3	-0.06
Drugs for obstructive airway diseases	15.1	7.2	0.25	15.6	20	-0.11
Drugs used in diabetes	5.8	3.1	0.13	5.9	6.5	-0.02
Immunosuppressants	0.6	0.7	-0.02	0.6	0.7	-0.01
Lipid modifying agents	9.4	4.7	0.18	9.7	11.3	-0.05
Opioids	8.8	2.6	0.27	9.1	10.6	-0.05
Psycholeptics	5.1	2.8	0.12	5.2	6.6	-0.06
Psychostimulants, agents used for ADHD and nootropics	0.3	0.2	0.01	0.3	0.3	0

Table S6.42. Selected baseline characteristics for Germany IQVIA, for the short-term risk of personality disorders

Characteristic	Before propensity score matching			After propensity score matching		
	Targets, %	Comparators, %	Standardized difference	Targets, %	Comparators, %	Standardized difference
**Age group (years)**						
0-4	1.4	2.3	-0.07	1.4	2.7	-0.09
5-9	2.2	3.3	-0.06	2.2	3.2	-0.06
10-14	2.8	3.3	-0.03	2.7	3.3	-0.04
15-19	5	3.5	0.07	4.9	5.7	-0.04
20-24	7.6	3.9	0.16	7.6	7.7	-0.01
25-29	7.4	4.3	0.13	7.3	7.1	0.01
30-34	8	5.1	0.12	7.8	7.6	0.01
35-39	7.6	5.3	0.1	7.6	7.4	0.01
40-44	8.3	5.5	0.11	8.3	7.8	0.02
45-49	8.1	6	0.08	8.2	7.2	0.04
50-54	9.9	8.2	0.06	10	9.2	0.03
55-59	9.6	9.4	0.01	9.7	9.1	0.02
60-64	7	8.7	-0.06	7.1	6.9	0.01
65-69	3.6	7.8	-0.18	3.7	3.4	0.01
70-74	2.9	6.8	-0.18	2.9	2.6	0.02
75-79	2.5	6.3	-0.19	2.5	2.6	0
80-84	2.7	6.2	-0.17	2.7	2.5	0.01
85-89	2	2.7	-0.05	2	2.3	-0.02
90-94	1.1	1	0.01	1.1	1.4	-0.03
95-99	0.3	0.2	0.03	0.3	0.3	0
**Sex**						
Female	53.8	57.1	-0.07	53.9	53.1	0.02

Table S6.42. *Continued.* Selected baseline characteristics for Germany IQVIA, for the short-term risk of personality disorders

Characteristic	Before propensity score matching			After propensity score matching		
	**Targets, %**	**Comparators, %**	**Standardized difference**	**Targets, %**	**Comparators, %**	**Standardized difference**
**Medical history (general)**						
Acute respiratory disease	59	8	1.28	59.6	60.8	-0.03
Chronic liver disease	0.2	0.1	0.02	0.2	0.2	-0.02
Chronic obstructive lung disease	2.8	1.3	0.11	2.9	4.2	-0.07
Crohn's disease	0.2	0.1	0.02	0.2	0.2	-0.02
Dementia	1.2	0.5	0.08	1.2	1.7	-0.04
Depressive disorder	7.2	2.7	0.21	7.2	7.6	-0.01
Diabetes mellitus	4	2.2	0.1	4.1	5.2	-0.05
Gastroesophageal reflux disease	1.4	0.5	0.09	1.4	1.6	-0.01
Gastrointestinal hemorrhage	0.6	0.2	0.06	0.6	0.7	-0.01
Hyperlipidemia	5.5	2.4	0.16	5.7	6.7	-0.04
Hypertensive disorder	11.7	5.9	0.2	12.1	14.5	-0.07
Lesion of liver	0.2	0.1	0.01	0.2	0.3	-0.03
Obesity	2.6	1.1	0.1	2.6	2.6	0
Osteoarthritis	5.4	3.4	0.1	5.5	6.2	-0.03
Pneumonia	3	0.4	0.2	3	3.2	-0.01
Psoriasis	0.6	0.5	0.01	0.6	0.7	-0.01
Renal impairment	1.6	0.7	0.08	1.6	2.2	-0.04
Rheumatoid arthritis	0.6	0.4	0.02	0.6	0.8	-0.02
Schizophrenia	0.1	0.1	0	0.1	0.2	-0.01
Ulcerative colitis	0.2	0.1	0.02	0.2	0.3	-0.03
Urinary tract infectious disease	4.1	1.5	0.16	4.2	5.4	-0.06

Table S6.42. *Continued.* Selected baseline characteristics for Germany IQVIA, for the short-term risk of personality disorders

Characteristic	Before propensity score matching			After propensity score matching		
	**Targets, %**	**Comparators, %**	**Standardized difference**	**Targets, %**	**Comparators, %**	**Standardized difference**
**Medical history (cardiovascular disease)**						
Atrial fibrillation	0.8	0.5	0.03	0.8	1	-0.03
Cerebrovascular disease	1.2	0.7	0.05	1.2	1.5	-0.03
Coronary arteriosclerosis	1	0.6	0.04	1.1	1.4	-0.03
Heart disease	7.7	3.9	0.16	7.9	9.4	-0.05
Heart failure	1.8	0.8	0.09	1.9	2.3	-0.03
Ischemic heart disease	2.4	1.2	0.09	2.5	3	-0.03
Peripheral vascular disease	0.9	0.6	0.04	0.9	1.2	-0.03
Pulmonary embolism	0.3	0.1	0.04	0.3	0.5	-0.03
Venous thrombosis	0.7	0.3	0.06	0.7	1	-0.03
**Medical history (neoplasms)**						
Malignant lymphoma	0.1	0.1	0.02	0.2	0.2	0
Malignant neoplastic disease	1.9	2.1	-0.01	2	2.5	-0.03
Malignant tumor of breast	0.3	0.4	-0.02	0.3	0.3	0
Malignant tumor of colon	0.1	0.1	0	0.1	0.1	-0.01
Malignant tumor of urinary bladder	0.1	0.1	-0.01	0.1	0.1	0
Primary malignant neoplasm of prostate	0.2	0.3	-0.03	0.2	0.2	-0.01
**Medication use**						
Agents acting on the renin-angiotensin system	18.9	8.6	0.3	19.6	21.6	-0.05
Antibacterials for systemic use	26.6	9.2	0.47	27.4	32.7	-0.12
Antidepressants	6.2	3.5	0.13	6.4	7.9	-0.06
Antiepileptics	2.3	1.4	0.06	2.4	3	-0.04
Antiinflammatory and antirheumatic products	32.6	11.7	0.52	33.7	35.3	-0.04
Antineoplastic agents	1	0.9	0.01	1	1.2	-0.02
Antipsoriatics	0.2	0.3	0	0.3	0.3	0
Antithrombotic agents	10	4.6	0.21	10.3	12.4	-0.07

Table S6.42. *Continued.* Selected baseline characteristics for Germany IQVIA, for the short-term risk of personality disorders

Characteristic	Before propensity score matching			After propensity score matching		
	**Targets, %**	**Comparators, %**	**Standardized difference**	**Targets, %**	**Comparators, %**	**Standardized difference**
**Medication use**						
Beta blocking agents	12.1	6	0.22	12.5	14.1	-0.04
Calcium channel blockers	8.4	3.9	0.19	8.8	9.7	-0.03
Diuretics	11	5.1	0.22	11.4	13.2	-0.06
Drugs for acid-related disorders	20.1	6.7	0.4	20.8	23.3	-0.06
Drugs for obstructive airway diseases	15.1	7.2	0.25	15.6	20	-0.11
Drugs used in diabetes	5.8	3	0.14	6	6.5	-0.02
Immunosuppressants	0.6	0.7	-0.02	0.6	0.7	-0.01
Lipid modifying agents	9.4	4.6	0.19	9.7	11.3	-0.05
Opioids	8.8	2.6	0.27	9.1	10.6	-0.05
Psycholeptics	5.1	2.8	0.12	5.3	6.7	-0.06
Psychostimulants, agents used for ADHD and nootropics	0.3	0.2	0.01	0.3	0.3	0

Table S6.43. Selected baseline characteristics for Germany IQVIA, for the short-term risk of self-harm and suicide

**Characteristic**	**Before propensity score matching**			**After propensity score matching**		
	**Targets, %**	**Comparators, %**	**Standardized difference**	**Targets, %**	**Comparators, %**	**Standardized difference**
**Age group (years)**						
0-4	1.4	2.4	-0.07	1.4	2.7	-0.09
5-9	2.2	3.2	-0.06	2.2	3.2	-0.06
10-14	2.8	3.2	-0.02	2.7	3.3	-0.03
15-19	5	3.5	0.07	4.9	5.7	-0.04
20-24	7.6	4	0.16	7.5	7.7	-0.01
25-29	7.4	4.3	0.13	7.3	7.1	0.01
30-34	8	5.1	0.12	7.8	7.6	0.01
35-39	7.6	5.3	0.09	7.5	7.4	0.01
40-44	8.3	5.4	0.12	8.3	7.8	0.02
45-49	8.1	6	0.08	8.2	7.2	0.04
50-54	9.9	8.3	0.06	10	9.2	0.03
55-59	9.6	9.4	0.01	9.7	9.1	0.02
60-64	7	8.6	-0.06	7.1	6.9	0.01
65-69	3.6	7.9	-0.19	3.6	3.4	0.01
70-74	2.9	6.9	-0.19	2.9	2.6	0.02
75-79	2.5	6.4	-0.19	2.6	2.6	0
80-84	2.7	6.2	-0.17	2.7	2.5	0.01
85-89	2	2.7	-0.05	2	2.3	-0.02
90-94	1.1	1	0.01	1.1	1.4	-0.03
95-99	0.3	0.2	0.03	0.3	0.3	0
**Sex**						
Female	53.8	57.3	-0.07	53.9	53.1	0.01

Table S6.43. *Continued.* Selected baseline characteristics for Germany IQVIA, for the short-term risk of self-harm and suicide

**Characteristic**	**Before propensity score matching**			**After propensity score matching**		
	**Targets, %**	**Comparators, %**	**Standardized difference**	**Targets, %**	**Comparators, %**	**Standardized difference**
**Medical history (general)**						
Acute respiratory disease	59	7.8	1.29	59.6	60.9	-0.03
Chronic liver disease	0.2	0.1	0.03	0.2	0.2	-0.02
Chronic obstructive lung disease	2.8	1.2	0.11	2.9	4.2	-0.07
Crohn's disease	0.2	0.1	0.02	0.2	0.2	-0.02
Dementia	1.2	0.5	0.08	1.2	1.8	-0.04
Depressive disorder	7.2	2.7	0.21	7.3	7.8	-0.02
Diabetes mellitus	4	2.2	0.1	4.2	5.3	-0.05
Gastroesophageal reflux disease	1.4	0.5	0.1	1.4	1.6	-0.01
Gastrointestinal hemorrhage	0.6	0.2	0.06	0.6	0.7	-0.01
Hyperlipidemia	5.5	2.4	0.16	5.7	6.7	-0.04
Hypertensive disorder	11.7	5.9	0.2	12.1	14.6	-0.07
Lesion of liver	0.2	0.1	0.01	0.2	0.3	-0.02
Obesity	2.6	1.1	0.11	2.6	2.6	0
Osteoarthritis	5.4	3.4	0.09	5.5	6.3	-0.03
Pneumonia	3	0.4	0.2	3	3.3	-0.01
Psoriasis	0.6	0.5	0.01	0.6	0.7	-0.01
Renal impairment	1.6	0.7	0.08	1.6	2.2	-0.05
Rheumatoid arthritis	0.6	0.4	0.03	0.6	0.8	-0.02
Schizophrenia	0.1	0.1	0.01	0.1	0.2	-0.01
Ulcerative colitis	0.2	0.1	0.01	0.2	0.3	-0.03
Urinary tract infectious disease	4.1	1.5	0.16	4.2	5.4	-0.06

Table S6.43. *Continued.* Selected baseline characteristics for Germany IQVIA, for the short-term risk of self-harm and suicide

**Characteristic**	**Before propensity score matching**			**After propensity score matching**		
	**Targets, %**	**Comparators, %**	**Standardized difference**	**Targets, %**	**Comparators, %**	**Standardized difference**
**Medical history (cardiovascular disease)**						
Atrial fibrillation	0.8	0.5	0.04	0.8	1	-0.03
Cerebrovascular disease	1.2	0.7	0.05	1.2	1.6	-0.03
Coronary arteriosclerosis	1	0.6	0.04	1.1	1.4	-0.03
Heart disease	7.7	3.9	0.16	7.9	9.5	-0.06
Heart failure	1.8	0.9	0.08	1.9	2.4	-0.04
Ischemic heart disease	2.4	1.2	0.09	2.5	3	-0.04
Peripheral vascular disease	0.9	0.6	0.03	0.9	1.2	-0.03
Pulmonary embolism	0.3	0.1	0.04	0.3	0.5	-0.03
Venous thrombosis	0.7	0.3	0.06	0.7	1	-0.03
**Medical history (neoplasms)**						
Malignant lymphoma	0.1	0.1	0.02	0.2	0.2	0
Malignant neoplastic disease	1.9	2.1	-0.01	2	2.5	-0.03
Malignant tumor of breast	0.3	0.4	-0.01	0.3	0.3	0
Malignant tumor of colon	0.1	0.1	0	0.1	0.1	-0.01
Malignant tumor of urinary bladder	0.1	0.1	-0.01	0.1	0.1	0
Primary malignant neoplasm of prostate	0.2	0.3	-0.03	0.2	0.2	-0.01
**Medication use**						
Agents acting on the renin-angiotensin system	18.9	8.7	0.3	19.6	21.6	-0.05
Antibacterials for systemic use	26.6	9.2	0.47	27.4	32.7	-0.12
Antidepressants	6.2	3.5	0.12	6.4	8.1	-0.06
Antiepileptics	2.3	1.5	0.06	2.4	3	-0.04
Antiinflammatory and antirheumatic products	32.6	11.7	0.52	33.7	35.3	-0.04
Antineoplastic agents	1	0.9	0.01	1	1.2	-0.02
Antipsoriatics	0.2	0.2	0	0.3	0.3	0
Antithrombotic agents	10	4.7	0.2	10.3	12.4	-0.07

Table S6.43. *Continued.* Selected baseline characteristics for Germany IQVIA, for the short-term risk of self-harm and suicide

Characteristic	Before propensity score matching			After propensity score matching		
	**Targets, %**	**Comparators, %**	**Standardized difference**	**Targets, %**	**Comparators, %**	**Standardized difference**
**Medication use**						
Beta blocking agents	12.1	5.9	0.22	12.5	14.1	-0.05
Calcium channel blockers	8.4	3.9	0.19	8.8	9.7	-0.03
Diuretics	11	5.3	0.21	11.4	13.3	-0.06
Drugs for acid-related disorders	20.1	6.7	0.4	20.8	23.4	-0.06
Drugs for obstructive airway diseases	15.1	7.2	0.26	15.7	20	-0.11
Drugs used in diabetes	5.8	3	0.14	6	6.5	-0.02
Immunosuppressants	0.6	0.7	-0.02	0.6	0.7	-0.01
Lipid modifying agents	9.4	4.7	0.18	9.7	11.3	-0.05
Opioids	8.8	2.6	0.27	9.1	10.6	-0.05
Psycholeptics	5.1	2.7	0.12	5.3	6.8	-0.06
Psychostimulants, agents used for ADHD and nootropics	0.3	0.2	0	0.3	0.3	0

Table S6.44. Selected baseline characteristics for Germany IQVIA, for the short-term risk of sleep disorders

Characteristic	Before propensity score matching			After propensity score matching		
	Targets, %	Comparators, %	Standardized difference	Targets, %	Comparators, %	Standardized difference
**Age group (years)**						
0-4	1.4	2.4	-0.07	1.5	2.7	-0.09
5-9	2.2	3.3	-0.07	2.3	3.3	-0.06
10-14	2.8	3.3	-0.03	2.8	3.5	-0.04
15-19	5	3.5	0.07	5	5.9	-0.04
20-24	7.6	3.9	0.16	7.7	7.9	-0.01
25-29	7.4	4.2	0.14	7.4	7.2	0.01
30-34	8	5.1	0.12	7.9	7.7	0.01
35-39	7.6	5.3	0.1	7.6	7.5	0
40-44	8.3	5.5	0.11	8.4	7.9	0.02
45-49	8.1	5.9	0.09	8.2	7.1	0.04
50-54	9.9	8.3	0.05	10	9.1	0.03
55-59	9.6	9.5	0	9.6	9	0.02
60-64	7	8.7	-0.06	7	6.7	0.01
65-69	3.6	7.8	-0.18	3.6	3.3	0.01
70-74	2.9	6.9	-0.19	2.9	2.5	0.02
75-79	2.5	6.3	-0.18	2.4	2.5	-0.01
80-84	2.7	6.2	-0.17	2.5	2.4	0.01
85-89	2	2.7	-0.05	1.9	2.2	-0.02
90-94	1.1	1	0.01	1	1.3	-0.03
95-99	0.3	0.2	0.03	0.3	0.3	0
**Sex**						
Female	53.8	57.1	-0.07	53.8	53.2	0.01

Table S6.44. *Continued.* Selected baseline characteristics for Germany IQVIA, for the short-term risk of sleep disorders

Characteristic	Before propensity score matching			After propensity score matching		
	**Targets, %**	**Comparators, %**	**Standardized difference**	**Targets, %**	**Comparators, %**	**Standardized difference**
**Medical history (general)**						
Acute respiratory disease	59	7.9	1.29	59.5	60.9	-0.03
Chronic liver disease	0.2	0.1	0.03	0.2	0.2	-0.02
Chronic obstructive lung disease	2.8	1.3	0.11	2.7	3.9	-0.07
Crohn’s disease	0.2	0.1	0.02	0.2	0.2	-0.02
Dementia	1.2	0.5	0.08	1.1	1.6	-0.04
Depressive disorder	7.2	2.7	0.21	6.6	6.8	-0.01
Diabetes mellitus	4	2.2	0.1	3.9	4.9	-0.05
Gastroesophageal reflux disease	1.4	0.5	0.1	1.4	1.5	-0.01
Gastrointestinal hemorrhage	0.6	0.2	0.06	0.5	0.6	-0.01
Hyperlipidemia	5.5	2.4	0.16	5.2	6.2	-0.04
Hypertensive disorder	11.7	5.8	0.21	11.3	13.6	-0.07
Lesion of liver	0.2	0.1	0.01	0.2	0.3	-0.02
Obesity	2.6	1.1	0.11	2.4	2.4	0
Osteoarthritis	5.4	3.5	0.09	5.1	5.9	-0.03
Pneumonia	3	0.4	0.2	2.9	3.1	-0.01
Psoriasis	0.6	0.5	0.01	0.6	0.7	0
Renal impairment	1.6	0.8	0.08	1.4	2	-0.05
Rheumatoid arthritis	0.6	0.4	0.02	0.6	0.8	-0.03
Schizophrenia	0.1	0.1	0	0.1	0.2	-0.01
Ulcerative colitis	0.2	0.1	0.02	0.1	0.3	-0.03
Urinary tract infectious disease	4.1	1.5	0.16	4	5.2	-0.06

Table S6.44. *Continued.* Selected baseline characteristics for Germany IQVIA, for the short-term risk of sleep disorders

Characteristic	Before propensity score matching			After propensity score matching		
	**Targets, %**	**Comparators, %**	**Standardized difference**	**Targets, %**	**Comparators, %**	**Standardized difference**
**Medical history (cardiovascular disease)**						
Atrial fibrillation	0.8	0.5	0.03	0.7	0.9	-0.02
Cerebrovascular disease	1.2	0.7	0.05	1.1	1.4	-0.03
Coronary arteriosclerosis	1	0.6	0.04	0.9	1.3	-0.03
Heart disease	7.7	3.9	0.16	7.3	8.8	-0.05
Heart failure	1.8	0.9	0.08	1.6	2.1	-0.04
Ischemic heart disease	2.4	1.2	0.09	2.3	2.8	-0.03
Peripheral vascular disease	0.9	0.6	0.04	0.8	1.1	-0.03
Pulmonary embolism	0.3	0.1	0.04	0.3	0.5	-0.03
Venous thrombosis	0.7	0.3	0.06	0.7	1	-0.03
**Medical history (neoplasms)**						
Malignant lymphoma	0.1	0.1	0.02	0.2	0.2	-0.01
Malignant neoplastic disease	1.9	2.1	-0.01	1.8	2.3	-0.04
Malignant tumor of breast	0.3	0.4	-0.02	0.3	0.3	0
Malignant tumor of colon	0.1	0.1	0	0.1	0.1	-0.01
Primary malignant neoplasm of prostate	0.2	0.3	-0.03	0.2	0.2	0
**Medication use**						
Agents acting on the renin-angiotensin system	18.9	8.6	0.3	18.9	20.7	-0.04
Antibacterials for systemic use	26.6	9.1	0.47	26.8	32.4	-0.12
Antidepressants	6.2	3.5	0.13	5.7	7.2	-0.06
Antiepileptics	2.3	1.5	0.06	2.2	2.8	-0.04
Antiinflammatory and antirheumatic products	32.6	11.8	0.52	33.2	35	-0.04
Antineoplastic agents	1	0.8	0.01	1	1.2	-0.02
Antipsoriatics	0.2	0.3	0	0.2	0.3	0
Antithrombotic agents	10	4.6	0.2	9.8	11.8	-0.07
Beta blocking agents	12.1	5.9	0.22	12	13.5	-0.05

Table S6.44. *Continued.* Selected baseline characteristics for Germany IQVIA, for the short-term risk of sleep disorders

Characteristic	Before propensity score matching			After propensity score matching		
	**Targets, %**	**Comparators, %**	**Standardized difference**	**Targets, %**	**Comparators, %**	**Standardized difference**
**Medication use**						
Calcium channel blockers	8.4	3.9	0.19	8.3	9.2	-0.03
Diuretics	11	5.2	0.21	10.8	12.6	-0.06
Drugs for acid-related disorders	20.1	6.9	0.4	20.1	22.6	-0.06
Drugs for obstructive airway diseases	15.1	7.2	0.25	15.3	19.6	-0.11
Drugs used in diabetes	5.8	3	0.14	5.7	6.2	-0.02
Immunosuppressants	0.6	0.7	-0.02	0.6	0.7	-0.02
Lipid modifying agents	9.4	4.6	0.19	9.3	10.9	-0.05
Opioids	8.8	2.7	0.27	8.8	10.1	-0.04
Psycholeptics	5.1	2.7	0.13	4.2	5.4	-0.06
Psychostimulants, agents used for ADHD and nootropics	0.3	0.2	0	0.3	0.3	0

Table S6.45. Selected baseline characteristics for Germany IQVIA, for the short-term risk of dementia

Characteristic	Before propensity score matching			After propensity score matching		
	Targets, %	Comparators, %	Standardized difference	Targets, %	Comparators, %	Standardized difference
**Age group (years)**						
0-4	1.4	2.3	-0.07	1.4	2.7	-0.09
5-9	2.2	3.2	-0.06	2.2	3.3	-0.06
10-14	2.8	3.3	-0.03	2.7	3.4	-0.04
15-19	5	3.5	0.07	4.9	5.8	-0.04
20-24	7.6	4	0.16	7.6	7.8	-0.01
25-29	7.4	4.3	0.13	7.4	7.2	0.01
30-34	8	5.1	0.12	7.9	7.7	0.01
35-39	7.6	5.3	0.1	7.6	7.5	0
40-44	8.3	5.5	0.11	8.4	7.9	0.02
45-49	8.1	5.9	0.09	8.3	7.3	0.04
50-54	9.9	8.3	0.05	10.1	9.3	0.03
55-59	9.6	9.5	0	9.8	9.2	0.02
60-64	7	8.7	-0.06	7.2	7	0.01
65-69	3.6	7.8	-0.18	3.7	3.4	0.01
70-74	2.9	6.9	-0.19	2.9	2.6	0.02
75-79	2.5	6.3	-0.19	2.4	2.5	0
80-84	2.7	6.2	-0.17	2.4	2.2	0.01
85-89	2	2.7	-0.05	1.7	1.9	-0.01
90-94	1.1	1	0.01	0.9	1.1	-0.02
95-99	0.3	0.2	0.03	0.2	0.2	0.01
**Sex**						
Female	53.8	57.1	-0.06	53.7	53	0.01

Table S6.45. *Continued.* Selected baseline characteristics for Germany IQVIA, for the short-term risk of dementia

Characteristic	Before propensity score matching			After propensity score matching		
	**Targets, %**	**Comparators, %**	**Standardized difference**	**Targets, %**	**Comparators, %**	**Standardized difference**
**Medical history (general)**						
Acute respiratory disease	59	7.9	1.29	59.9	61.2	-0.03
Chronic liver disease	0.2	0.1	0.03	0.2	0.2	-0.02
Chronic obstructive lung disease	2.8	1.3	0.11	2.8	4.1	-0.07
Crohn’s disease	0.2	0.1	0.02	0.2	0.2	-0.02
Depressive disorder	7.2	2.7	0.2	7.1	7.4	-0.01
Diabetes mellitus	4	2.3	0.1	3.9	5	-0.05
Gastroesophageal reflux disease	1.4	0.5	0.1	1.4	1.6	-0.01
Gastrointestinal hemorrhage	0.6	0.2	0.06	0.5	0.6	-0.01
Hyperlipidemia	5.5	2.4	0.16	5.5	6.4	-0.04
Hypertensive disorder	11.7	5.9	0.2	11.6	13.9	-0.07
Lesion of liver	0.2	0.1	0.01	0.2	0.3	-0.03
Obesity	2.6	1.1	0.11	2.6	2.6	0
Osteoarthritis	5.4	3.4	0.1	5.4	6	-0.03
Pneumonia	3	0.4	0.2	2.9	3.1	-0.01
Psoriasis	0.6	0.5	0.02	0.6	0.7	-0.01
Renal impairment	1.6	0.7	0.08	1.4	1.9	-0.04
Rheumatoid arthritis	0.6	0.4	0.03	0.6	0.8	-0.03
Schizophrenia	0.1	0.1	0.01	0.1	0.1	-0.01
Ulcerative colitis	0.2	0.1	0.02	0.2	0.3	-0.02
Urinary tract infectious disease	4.1	1.5	0.16	4.1	5.2	-0.05

Table S6.45. *Continued.* Selected baseline characteristics for Germany IQVIA, for the short-term risk of dementia

Characteristic	Before propensity score matching			After propensity score matching		
	Targets, %	Comparators, %	Standardized difference	Targets, %	Comparators, %	Standardized difference
**Medical history (cardiovascular disease)**						
Atrial fibrillation	0.8	0.5	0.03	0.7	0.9	-0.02
Cerebrovascular disease	1.2	0.7	0.05	1.1	1.3	-0.02
Coronary arteriosclerosis	1	0.6	0.04	1	1.3	-0.03
Heart disease	7.7	4	0.16	7.5	8.8	-0.05
Heart failure	1.8	0.9	0.08	1.6	2	-0.03
Ischemic heart disease	2.4	1.3	0.09	2.3	2.8	-0.03
Peripheral vascular disease	0.9	0.6	0.04	0.8	1	-0.03
Pulmonary embolism	0.3	0.1	0.04	0.3	0.5	-0.03
Venous thrombosis	0.7	0.3	0.06	0.7	1	-0.03
**Medical history (neoplasms)**						
Malignant lymphoma	0.1	0.1	0.02	0.2	0.2	0
Malignant neoplastic disease	1.9	2.1	-0.01	1.9	2.4	-0.03
Malignant tumor of breast	0.3	0.4	-0.02	0.3	0.3	0
Malignant tumor of colon	0.1	0.1	0	0.1	0.1	-0.01
Primary malignant neoplasm of prostate	0.2	0.3	-0.03	0.1	0.2	-0.01
**Medication use**						
Agents acting on the renin-angiotensin system	18.9	8.7	0.3	19.2	21	-0.04
Antibacterials for systemic use	26.6	9.2	0.47	27.2	32.5	-0.12
Antidepressants	6.2	3.6	0.12	6.1	7.7	-0.06
Antiepileptics	2.3	1.4	0.06	2.3	2.8	-0.03
Antiinflammatory and antirheumatic products	32.6	11.6	0.52	33.5	35.2	-0.04
Antineoplastic agents	1	0.9	0.01	1	1.2	-0.02
Antipsoriatics	0.2	0.3	0	0.3	0.3	0
Antithrombotic agents	10	4.6	0.21	9.8	11.5	-0.06
Beta blocking agents	12.1	6	0.21	12.1	13.5	-0.04

Table S6.45. *Continued.* Selected baseline characteristics for Germany IQVIA, for the short-term risk of dementia

Characteristic	Before propensity score matching			After propensity score matching		
	**Targets, %**	**Comparators, %**	**Standardized difference**	**Targets, %**	**Comparators, %**	**Standardized difference**
**Medication use**						
Calcium channel blockers	8.4	3.9	0.19	8.5	9.3	-0.03
Diuretics	11	5.2	0.21	10.8	12.4	-0.05
Drugs for acid-related disorders	20.1	6.8	0.4	20.4	22.9	-0.06
Drugs for obstructive airway diseases	15.1	7.3	0.25	15.6	19.9	-0.11
Drugs used in diabetes	5.8	3	0.13	5.7	6.2	-0.02
Immunosuppressants	0.6	0.7	-0.02	0.6	0.7	-0.01
Lipid modifying agents	9.4	4.7	0.18	9.5	10.9	-0.05
Opioids	8.8	2.7	0.27	9	10.3	-0.04
Psycholeptics	5.1	2.8	0.12	4.9	6	-0.05
Psychostimulants, agents used for ADHD and nootropics	0.3	0.2	0	0.2	0.3	-0.01

Table S6.46. Selected baseline characteristics for Germany IQVIA, for the short-term risk of neurodevelopmental disorders

**Characteristic**	**Before propensity score matching**			**After propensity score matching**		
	**Targets, %**	**Comparators, %**	**Standardized difference**	**Targets, %**	**Comparators, %**	**Standardized difference**
**Age group (years)**						
0-4	1.4	2.4	-0.07	1.3	2.4	-0.09
5-9	2.2	3.3	-0.07	2	2.9	-0.06
10-14	2.8	3.3	-0.03	2.6	3.1	-0.03
15-19	5	3.5	0.07	4.8	5.6	-0.04
20-24	7.6	4	0.16	7.6	7.8	-0.01
25-29	7.4	4.2	0.13	7.4	7.2	0.01
30-34	8	5.2	0.11	7.9	7.7	0.01
35-39	7.6	5.3	0.1	7.6	7.5	0.01
40-44	8.3	5.5	0.11	8.4	7.9	0.02
45-49	8.1	6	0.08	8.3	7.2	0.04
50-54	9.9	8.3	0.06	10.1	9.3	0.03
55-59	9.6	9.4	0.01	9.8	9.2	0.02
60-64	7	8.8	-0.07	7.2	7	0.01
65-69	3.6	7.7	-0.18	3.7	3.4	0.01
70-74	2.9	6.8	-0.18	2.9	2.6	0.02
75-79	2.5	6.4	-0.19	2.6	2.6	0
80-84	2.7	6.1	-0.17	2.7	2.5	0.01
85-89	2	2.7	-0.05	2	2.3	-0.02
90-94	1.1	1	0.01	1.1	1.4	-0.03
95-99	0.3	0.2	0.03	0.3	0.3	0
**Sex**						
Female	53.8	57.2	-0.07	54	53.3	0.01

Table S6.46. *Continued.* Selected baseline characteristics for Germany IQVIA, for the short-term risk of neurodevelopmental disorders

Characteristic	Before propensity score matching			After propensity score matching		
	**Targets, %**	**Comparators, %**	**Standardized difference**	**Targets, %**	**Comparators, %**	**Standardized difference**
**Medical history (general)**						
Acute respiratory disease	59	7.9	1.29	59.5	60.8	-0.03
Chronic liver disease	0.2	0.1	0.03	0.2	0.2	-0.02
Chronic obstructive lung disease	2.8	1.3	0.11	2.9	4.2	-0.07
Crohn’s disease	0.2	0.1	0.02	0.2	0.2	-0.02
Dementia	1.2	0.5	0.07	1.2	1.7	-0.04
Depressive disorder	7.2	2.7	0.21	7.3	7.7	-0.01
Diabetes mellitus	4	2.3	0.1	4.2	5.3	-0.05
Gastroesophageal reflux disease	1.4	0.5	0.1	1.4	1.6	-0.02
Gastrointestinal hemorrhage	0.6	0.2	0.06	0.6	0.7	-0.01
Hyperlipidemia	5.5	2.4	0.16	5.7	6.8	-0.04
Hypertensive disorder	11.7	5.9	0.2	12.1	14.7	-0.08
Lesion of liver	0.2	0.1	0.01	0.2	0.3	-0.02
Obesity	2.6	1.1	0.11	2.6	2.5	0
Osteoarthritis	5.4	3.4	0.09	5.5	6.3	-0.03
Pneumonia	3	0.4	0.2	3	3.2	-0.01
Psoriasis	0.6	0.5	0.02	0.7	0.7	0
Renal impairment	1.6	0.7	0.08	1.6	2.2	-0.05
Rheumatoid arthritis	0.6	0.4	0.03	0.6	0.8	-0.02
Schizophrenia	0.1	0.1	0.01	0.1	0.2	-0.01
Ulcerative colitis	0.2	0.1	0.01	0.2	0.3	-0.03
Urinary tract infectious disease	4.1	1.5	0.16	4.2	5.3	-0.05

Table S6.46. *Continued.* Selected baseline characteristics for Germany IQVIA, for the short-term risk of neurodevelopmental disorders

Characteristic	Before propensity score matching			After propensity score matching		
	Targets, %	Comparators, %	Standardized difference	Targets, %	Comparators, %	Standardized difference
**Medical history (cardiovascular disease)**						
Atrial fibrillation	0.8	0.5	0.03	0.8	1	-0.03
Cerebrovascular disease	1.2	0.7	0.05	1.2	1.5	-0.03
Coronary arteriosclerosis	1	0.6	0.04	1.1	1.4	-0.04
Heart disease	7.7	4	0.16	7.9	9.5	-0.06
Heart failure	1.8	0.9	0.08	1.9	2.3	-0.03
Ischemic heart disease	2.4	1.3	0.08	2.5	3	-0.03
Peripheral vascular disease	0.9	0.6	0.04	0.9	1.2	-0.03
Pulmonary embolism	0.3	0.1	0.04	0.3	0.5	-0.03
Venous thrombosis	0.7	0.3	0.05	0.7	1	-0.03
**Medical history (neoplasms)**						
Malignant lymphoma	0.1	0.1	0.02	0.2	0.2	0
Malignant neoplastic disease	1.9	2.1	-0.01	2	2.5	-0.03
Malignant tumor of breast	0.3	0.4	-0.02	0.3	0.3	0
Malignant tumor of colon	0.1	0.1	0	0.1	0.1	-0.01
Malignant tumor of urinary bladder	0.1	0.1	-0.01	0.1	0.1	0
Primary malignant neoplasm of prostate	0.2	0.3	-0.03	0.2	0.2	-0.01
**Medication use**						
Agents acting on the renin-angiotensin system	18.9	8.6	0.3	19.7	21.8	-0.05
Antibacterials for systemic use	26.6	9.3	0.46	27.4	32.6	-0.12
Antidepressants	6.2	3.5	0.13	6.4	8.1	-0.07
Antiepileptics	2.3	1.4	0.06	2.3	2.9	-0.04
Antiinflammatory and antirheumatic products	32.6	11.8	0.52	33.5	35.1	-0.03
Antineoplastic agents	1	0.9	0.01	1	1.2	-0.02
Antipsoriatics	0.2	0.3	0	0.3	0.3	0

Table S6.46. *Continued.* Selected baseline characteristics for Germany IQVIA, for the short-term risk of neurodevelopmental disorders

Characteristic	Before propensity score matching			After propensity score matching		
	**Targets, %**	**Comparators, %**	**Standardized difference**	**Targets, %**	**Comparators, %**	**Standardized difference**
**Medication use**						
Antithrombotic agents	10	4.6	0.21	10.3	12.5	-0.07
Beta blocking agents	12.1	6	0.21	12.5	14.2	-0.05
Calcium channel blockers	8.4	3.9	0.19	8.8	9.8	-0.03
Diuretics	11	5.3	0.21	11.4	13.3	-0.06
Drugs for acid-related disorders	20.1	6.8	0.4	20.8	23.5	-0.06
Drugs for obstructive airway diseases	15.1	7.3	0.25	15.6	19.9	-0.11
Drugs used in diabetes	5.8	3	0.14	6	6.5	-0.02
Immunosuppressants	0.6	0.7	-0.02	0.6	0.7	-0.01
Lipid modifying agents	9.4	4.7	0.18	9.7	11.4	-0.05
Opioids	8.8	2.6	0.27	9.2	10.7	-0.05
Psycholeptics	5.1	2.7	0.12	5.2	6.7	-0.06
Psychostimulants, agents used for ADHD and nootropics	0.3	0.2	0	0.2	0.2	0

Table S6.47. Selected baseline characteristics for Germany IQVIA, for the short-term risk of any of psychiatric and neuropsychiatric disorders

Characteristic	Before propensity score matching			After propensity score matching		
	Targets, %	Comparators, %	Standardized difference	Targets, %	Comparators, %	Standardized difference
**Age group (years)**						
0-4	1.4	2.3	-0.07	1.5	2.9	-0.1
5-9	2.2	3.2	-0.06	2.3	3.4	-0.07
10-14	2.8	3.3	-0.03	2.9	3.7	-0.04
15-19	5	3.5	0.07	5.2	6.2	-0.04
20-24	7.6	4	0.16	7.9	8.2	-0.01
25-29	7.4	4.3	0.13	7.5	7.4	0
35-39	7.6	5.3	0.1	7.7	7.5	0.01
40-44	8.3	5.5	0.11	8.3	7.8	0.02
45-49	8.1	6	0.08	8.3	7.1	0.04
50-54	9.9	8.3	0.06	10.2	9	0.04
55-59	9.6	9.4	0.01	9.6	8.8	0.03
60-64	7	8.7	-0.06	7	6.6	0.01
65-69	3.6	7.8	-0.18	3.6	3.3	0.01
70-74	2.9	6.9	-0.19	2.9	2.6	0.02
75-79	2.5	6.4	-0.19	2.3	2.4	-0.01
80-84	2.7	6.2	-0.17	2.3	2.1	0.01
85-89	2	2.7	-0.05	1.7	1.8	-0.01
90-94	1.1	1	0.02	0.8	1	-0.02
95-99	0.3	0.2	0.03	0.2	0.2	0.01
**Sex**						
Female	53.8	57	-0.06	52.7	52.2	0.01

Table S6.47. *Continued.* Selected baseline characteristics for Germany IQVIA, for the short-term risk of any of psychiatric and neuropsychiatric disorders

Characteristic	Before propensity score matching			After propensity score matching		
	Targets, %	Comparators, %	Standardized difference	Targets, %	Comparators, %	Standardized difference
**Medical history (general)**						
Acute respiratory disease	59	7.9	1.29	59.1	60.5	-0.03
Chronic liver disease	0.2	0.1	0.03	0.1	0.2	-0.02
Chronic obstructive lung disease	2.8	1.3	0.11	2.2	3.2	-0.06
Crohn’s disease	0.2	0.1	0.02	0.1	0.2	-0.02
Depressive disorder	7.2	2.7	0.21	0.5	0.1	0.09
Diabetes mellitus	4	2.2	0.1	3.4	4.2	-0.04
Gastroesophageal reflux disease	1.4	0.5	0.09	1.1	1.3	-0.01
Gastrointestinal hemorrhage	0.6	0.2	0.06	0.5	0.5	-0.01
Hyperlipidemia	5.5	2.4	0.16	4.5	5.3	-0.04
Hypertensive disorder	11.7	5.9	0.2	9.9	11.7	-0.06
Lesion of liver	0.2	0.1	0.01	0.1	0.2	-0.03
Obesity	2.6	1.1	0.11	2	1.9	0
Osteoarthritis	5.4	3.4	0.09	4.4	5	-0.03
Psoriasis	0.6	0.6	0.01	0.6	0.6	0
Renal impairment	1.6	0.7	0.08	1.1	1.6	-0.04
Rheumatoid arthritis	0.6	0.4	0.02	0.5	0.7	-0.02
Ulcerative colitis	0.2	0.1	0.01	0.1	0.2	-0.03
Urinary tract infectious disease	4.1	1.5	0.16	3.6	4.7	-0.06

Table S6.47. *Continued.* Selected baseline characteristics for Germany IQVIA, for the short-term risk of any of psychiatric and neuropsychiatric disorders

Characteristic	Before propensity score matching			After propensity score matching		
	Targets, %	Comparators, %	Standardized difference	Targets, %	Comparators, %	Standardized difference
**Medical history (cardiovascular disease)**						
Atrial fibrillation	0.8	0.5	0.03	0.6	0.7	-0.02
Cerebrovascular disease	1.2	0.7	0.05	0.9	1	-0.01
Coronary arteriosclerosis	1	0.6	0.04	0.8	1.1	-0.03
Heart disease	7.7	4	0.16	6.3	7.3	-0.04
Heart failure	1.8	0.9	0.08	1.3	1.6	-0.03
Ischemic heart disease	2.4	1.3	0.08	1.9	2.2	-0.02
Peripheral vascular disease	0.9	0.6	0.04	0.6	0.8	-0.03
Pulmonary embolism	0.3	0.1	0.04	0.3	0.4	-0.02
Venous thrombosis	0.7	0.3	0.06	0.6	0.9	-0.03
**Medical history (neoplasms)**						
Malignant lymphoma	0.1	0.1	0.02	0.1	0.1	0
Malignant neoplastic disease	1.9	2.1	-0.01	1.6	2	-0.03
Malignant tumor of breast	0.3	0.4	-0.02	0.2	0.2	0
Malignant tumor of colon	0.1	0.1	0	0.1	0.1	-0.01
Primary malignant neoplasm of prostate	0.2	0.3	-0.03	0.1	0.1	-0.01
**Medication use**						
Agents acting on the renin-angiotensin system	18.9	8.7	0.3	18.1	19.3	-0.03
Antibacterials for systemic use	26.6	9.2	0.47	25.7	31.2	-0.12
Antidepressants	6.2	3.5	0.12	3.3	3.7	-0.02
Antiepileptics	2.3	1.5	0.06	1.9	2.2	-0.02
Antiinflammatory and antirheumatic products	32.6	11.8	0.52	31.9	33.7	-0.04
Antineoplastic agents	1	0.9	0.01	1	1.1	-0.02
Antipsoriatics	0.2	0.3	0	0.2	0.2	0

Table S6.47. *Continued.* Selected baseline characteristics for Germany IQVIA, for the short-term risk of any of psychiatric and neuropsychiatric disorders

**Characteristic**	**Before propensity score matching**			**After propensity score matching**		
	**Targets, %**	**Comparators, %**	**Standardized difference**	**Targets, %**	**Comparators, %**	**Standardized difference**
**Medication use**						
Antithrombotic agents	10	4.6	0.21	9	10.5	-0.05
Beta blocking agents	12.1	5.9	0.22	11.1	12.2	-0.03
Calcium channel blockers	8.4	3.9	0.19	7.9	8.5	-0.02
Diuretics	11	5.2	0.21	10	11.2	-0.04
Drugs for acid-related disorders	20.1	6.7	0.4	18.4	20.5	-0.05
Drugs for obstructive airway diseases	15.1	7.3	0.25	14.6	18.8	-0.11
Drugs used in diabetes	5.8	3	0.14	5.3	5.7	-0.02
Immunosuppressants	0.6	0.8	-0.02	0.6	0.7	-0.01
Lipid modifying agents	9.4	4.7	0.18	8.8	10.1	-0.04
Opioids	8.8	2.6	0.27	8.3	9.2	-0.03
Psycholeptics	5.1	2.7	0.12	2.9	3.3	-0.03
Psychostimulants, agents used for ADHD and nootropics	0.3	0.2	0.01	0.1	0.2	-0.01

Table S6.48. Selected baseline characteristics for Germany IQVIA, for the medium-term risk of depression

Characteristic	Before propensity score matching			After propensity score matching		
	Targets, %	Comparators, %	Standardized difference	Targets, %	Comparators, %	Standardized difference
**Age group (years)**						
0-4	1.4	2.4	-0.07	1.6	3.1	-0.1
5-9	2.2	3.2	-0.06	2.5	3.9	-0.08
10-14	2.8	3.3	-0.03	3	3.8	-0.05
15-19	5	3.5	0.07	5.1	5.7	-0.03
25-29	7.4	4.2	0.13	7.3	6.8	0.02
30-34	8	5.2	0.11	7.8	7.4	0.01
35-39	7.6	5.3	0.09	7.6	7.5	0
40-44	8.3	5.5	0.11	8.2	7.9	0.01
45-49	8.1	6	0.08	8.3	7.4	0.03
50-54	9.9	8.3	0.05	10.3	9	0.04
55-59	9.6	9.4	0.01	9.8	9	0.03
60-64	7	8.7	-0.06	7.3	7	0.01
65-69	3.6	7.8	-0.18	3.7	3.5	0.01
70-74	2.9	6.7	-0.18	3	2.7	0.01
75-79	2.5	6.4	-0.19	2.3	2.5	-0.01
80-84	2.7	6.2	-0.17	2.3	2.3	0
85-89	2	2.7	-0.05	1.4	1.7	-0.02
90-94	1.1	1	0.01	0.8	0.9	-0.01
95-99	0.3	0.2	0.03	0.2	0.2	0
**Sex**						
Female	53.8	57.1	-0.07	52.5	52	0.01

Table S6.48. *Continued.* Selected baseline characteristics for Germany IQVIA, for the medium-term risk of depression

Characteristic	Before propensity score matching			After propensity score matching		
	**Targets, %**	**Comparators, %**	**Standardized difference**	**Targets, %**	**Comparators, %**	**Standardized difference**
**Medical history (general)**						
Acute respiratory disease	59	7.9	1.29	59.8	62	-0.04
Chronic liver disease	0.2	0.1	0.03	0.1	0.2	-0.02
Chronic obstructive lung disease	2.8	1.2	0.11	2.5	3.9	-0.08
Crohn's disease	0.2	0.1	0.02	0.2	0.3	-0.03
Dementia	1.2	0.5	0.07	0.7	1	-0.03
Diabetes mellitus	4	2.2	0.1	3.6	4.8	-0.06
Gastroesophageal reflux disease	1.4	0.5	0.1	1.3	1.5	-0.02
Gastrointestinal hemorrhage	0.6	0.2	0.06	0.6	0.5	0.01
Hyperlipidemia	5.5	2.4	0.16	5	6	-0.04
Hypertensive disorder	11.7	6	0.2	11	13.2	-0.07
Lesion of liver	0.2	0.1	0.01	0.1	0.2	-0.03
Obesity	2.6	1.1	0.11	2.2	2.7	-0.03
Osteoarthritis	5.4	3.4	0.1	4.9	5.6	-0.03
Pneumonia	3	0.4	0.2	2.8	3.1	-0.02
Psoriasis	0.6	0.6	0.01	0.6	0.7	-0.01
Renal impairment	1.6	0.7	0.08	1.2	1.7	-0.04
Rheumatoid arthritis	0.6	0.5	0.02	0.6	0.7	-0.02
Schizophrenia	0.1	0.1	0.01	0.1	0.1	-0.01
Ulcerative colitis	0.2	0.1	0.02	0.1	0.2	-0.04
Urinary tract infectious disease	4.1	1.5	0.16	3.8	4.9	-0.06

Table S6.48. *Continued.* Selected baseline characteristics for Germany IQVIA, for the medium-term risk of depression

**Characteristic**	**Before propensity score matching**			**After propensity score matching**		
	**Targets, %**	**Comparators, %**	**Standardized difference**	**Targets, %**	**Comparators, %**	**Standardized difference**
**Medical history (cardiovascular disease)**						
Atrial fibrillation	0.8	0.5	0.03	0.6	0.9	-0.03
Cerebrovascular disease	1.2	0.7	0.05	1	1.2	-0.02
Coronary arteriosclerosis	1	0.6	0.04	0.9	1.2	-0.03
Heart disease	7.7	3.9	0.16	6.8	8.3	-0.06
Heart failure	1.8	0.9	0.08	1.4	2	-0.04
Ischemic heart disease	2.4	1.2	0.09	2	2.7	-0.05
Peripheral vascular disease	0.9	0.6	0.03	0.7	1	-0.03
Pulmonary embolism	0.3	0.1	0.04	0.2	0.4	-0.03
Venous thrombosis	0.7	0.3	0.06	0.7	1	-0.03
Medical history: Neoplasms						
Malignant lymphoma	0.1	0.1	0.02	0.1	0.2	-0.01
Malignant neoplastic disease	1.9	2.1	-0.01	1.7	2.3	-0.04
Malignant tumor of breast	0.3	0.4	-0.02	0.2	0.3	-0.02
Malignant tumor of colon	0.1	0.1	0	0.1	0.1	-0.01
Malignant tumor of urinary bladder	0.1	0.1	-0.01	0.1	0.1	0
Primary malignant neoplasm of prostate	0.2	0.3	-0.04	0.1	0.2	-0.01
Medication use						
Agents acting on the renin-angiotensin system	18.9	8.6	0.3	19.2	20.7	-0.04
Antibacterials for systemic use	26.6	9.3	0.46	27.1	32.5	-0.12
Antidepressants	6.2	3.5	0.12	3.9	4.5	-0.03
Antiepileptics	2.3	1.5	0.06	2	2.5	-0.04
Antiinflammatory and antirheumatic products	32.6	11.7	0.52	33	35.2	-0.05
Antineoplastic agents	1	0.9	0.01	1	1.2	-0.02
Antipsoriatics	0.2	0.3	0	0.2	0.3	-0.01
Antithrombotic agents	10	4.6	0.21	9.4	11.3	-0.06

Table S6.48. *Continued.* Selected baseline characteristics for Germany IQVIA, for the medium-term risk of depression

**Characteristic**	**Before propensity score matching**			**After propensity score matching**		
	**Targets, %**	**Comparators, %**	**Standardized difference**	**Targets, %**	**Comparators, %**	**Standardized difference**
**Medication use**						
Beta blocking agents	12.1	5.9	0.22	11.8	13.4	-0.05
Calcium channel blockers	8.4	3.8	0.19	8.4	9.2	-0.03
Diuretics	11	5.1	0.22	10.4	12.3	-0.06
Drugs for acid-related disorders	20.1	6.8	0.4	19.4	22.1	-0.07
Drugs for obstructive airway diseases	15.1	7.2	0.25	15.4	19.8	-0.12
Drugs used in diabetes	5.8	3	0.14	5.6	6.3	-0.03
Immunosuppressants	0.6	0.7	-0.02	0.6	0.8	-0.03
Lipid modifying agents	9.4	4.6	0.19	9.2	11	-0.06
Opioids	8.8	2.6	0.27	8.4	9.5	-0.04
Psycholeptics	5.1	2.8	0.12	4.1	5	-0.04
Psychostimulants, agents used for ADHD and nootropics	0.3	0.2	0.01	0.2	0.3	-0.02

Table S6.49. Selected baseline characteristics for Germany IQVIA, for the medium-term risk of anxiety disorders

Characteristic	Before propensity score matching			After propensity score matching		
	Targets, %	Comparators, %	Standardized difference	Targets, %	Comparators, %	Standardized difference
**Age group (years)**						
0-4	1.4	2.4	-0.08	1.6	3	-0.09
5-9	2.2	3.3	-0.07	2.4	3.8	-0.08
10-14	2.8	3.3	-0.03	2.8	3.6	-0.04
15-19	5	3.5	0.07	5.1	5.5	-0.02
20-24	7.6	4	0.16	7.6	7.5	0
25-29	7.4	4.3	0.13	7.2	6.7	0.02
30-34	8	5.1	0.12	7.5	7.4	0
40-44	8.3	5.5	0.11	8.3	7.9	0.02
45-49	8.1	5.9	0.09	8.3	7.4	0.03
50-54	9.9	8.2	0.06	10.1	9.2	0.03
55-59	9.6	9.4	0.01	9.7	9.2	0.02
60-64	7	8.7	-0.06	7.4	7.1	0.01
65-69	3.6	7.8	-0.18	3.8	3.6	0.01
70-74	2.9	6.9	-0.19	3	2.8	0.01
75-79	2.5	6.4	-0.19	2.5	2.6	0
80-84	2.7	6.2	-0.17	2.5	2.4	0
85-89	2	2.7	-0.05	1.6	1.8	-0.02
90-94	1.1	1	0.01	0.9	0.9	-0.01
95-99	0.3	0.2	0.03	0.2	0.2	0.01
**Sex**						
Female	53.8	56.9	-0.06	52.9	52.3	0.01

Table S6.49. *Continued.* Selected baseline characteristics for Germany IQVIA, for the medium-term risk of anxiety disorders

Characteristic	Before propensity score matching			After propensity score matching		
	**Targets, %**	**Comparators, %**	**Standardized difference**	**Targets, %**	**Comparators, %**	**Standardized difference**
**Medical history (general)**						
Acute respiratory disease	59	7.8	1.29	59.6	62.1	-0.05
Chronic liver disease	0.2	0.1	0.03	0.1	0.2	-0.02
Chronic obstructive lung disease	2.8	1.3	0.11	3	4.3	-0.07
Crohn’s disease	0.2	0.1	0.02	0.2	0.3	-0.02
Dementia	1.2	0.5	0.07	0.9	1.2	-0.02
Depressive disorder	7.2	2.7	0.21	6.1	5.9	0.01
Diabetes mellitus	4	2.2	0.1	4.1	5.2	-0.05
Gastroesophageal reflux disease	1.4	0.5	0.1	1.4	1.6	-0.02
Gastrointestinal hemorrhage	0.6	0.2	0.06	0.6	0.6	0
Hyperlipidemia	5.5	2.4	0.16	5.5	6.5	-0.04
Hypertensive disorder	11.7	5.9	0.2	11.8	14.1	-0.07
Lesion of liver	0.2	0.1	0.01	0.1	0.3	-0.03
Obesity	2.6	1.1	0.11	2.5	2.9	-0.02
Osteoarthritis	5.4	3.4	0.1	5.3	5.9	-0.03
Pneumonia	3	0.4	0.2	2.9	3.2	-0.02
Psoriasis	0.6	0.5	0.02	0.6	0.7	-0.01
Renal impairment	1.6	0.7	0.08	1.4	1.8	-0.03
Rheumatoid arthritis	0.6	0.4	0.03	0.6	0.8	-0.02
Schizophrenia	0.1	0.1	0.01	0.1	0.1	-0.01
Ulcerative colitis	0.2	0.1	0.02	0.1	0.3	-0.03
Urinary tract infectious disease	4.1	1.4	0.16	4.1	5	-0.04

Table S6.49. *Continued.* Selected baseline characteristics for Germany IQVIA, for the medium-term risk of anxiety disorders

Characteristic	Before propensity score matching			After propensity score matching		
	Targets, %	Comparators, %	Standardized difference	Targets, %	Comparators, %	Standardized difference
**Medical history (cardiovascular disease)**						
Atrial fibrillation	0.8	0.5	0.03	0.8	0.9	-0.02
Cerebrovascular disease	1.2	0.7	0.05	1.2	1.3	-0.01
Coronary arteriosclerosis	1	0.6	0.05	1	1.2	-0.02
Heart disease	7.7	3.9	0.16	7.5	8.9	-0.05
Heart failure	1.8	0.8	0.09	1.7	2.2	-0.04
Ischemic heart disease	2.4	1.3	0.08	2.3	3	-0.04
Peripheral vascular disease	0.9	0.5	0.04	0.8	1.1	-0.03
Pulmonary embolism	0.3	0.1	0.04	0.3	0.4	-0.03
Venous thrombosis	0.7	0.3	0.06	0.7	1	-0.04
**Medical history (neoplasms)**						
Malignant lymphoma	0.1	0.1	0.02	0.1	0.2	-0.01
Malignant neoplastic disease	1.9	2	-0.01	1.8	2.5	-0.04
Malignant tumor of breast	0.3	0.4	-0.02	0.2	0.3	-0.01
Primary malignant neoplasm of prostate	0.2	0.3	-0.03	0.2	0.2	0
**Medication use**						
Agents acting on the renin-angiotensin system	18.9	8.7	0.3	19.7	21.4	-0.04
Antibacterials for systemic use	26.6	9.2	0.47	27.4	32.8	-0.12
Antidepressants	6.2	3.5	0.12	5.4	6.4	-0.04
Antiepileptics	2.3	1.5	0.06	2.2	2.7	-0.03
Antiinflammatory and antirheumatic products	32.6	11.7	0.52	34.1	35.5	-0.03
Antineoplastic agents	1	0.9	0.01	1	1.2	-0.02
Antipsoriatics	0.2	0.2	0	0.2	0.3	-0.01
Antithrombotic agents	10	4.6	0.21	9.8	11.8	-0.07
Beta blocking agents	12.1	5.9	0.22	12.3	13.9	-0.05
Calcium channel blockers	8.4	3.9	0.19	8.8	9.6	-0.03

Table S6.49. *Continued.* Selected baseline characteristics for Germany IQVIA, for the medium-term risk of anxiety disorders

Characteristic	Before propensity score matching			After propensity score matching		
	**Targets, %**	**Comparators, %**	**Standardized difference**	**Targets, %**	**Comparators, %**	**Standardized difference**
**Medication use**						
Diuretics	11	5.1	0.22	11	12.9	-0.06
Drugs for acid-related disorders	20.1	6.8	0.4	20.5	23	-0.06
Drugs for obstructive airway diseases	15.1	7.2	0.26	15.8	20.2	-0.11
Drugs used in diabetes	5.8	3	0.14	5.9	6.6	-0.03
Immunosuppressants	0.6	0.7	-0.02	0.6	0.8	-0.02
Lipid modifying agents	9.4	4.6	0.19	9.7	11.5	-0.06
Opioids	8.8	2.6	0.27	8.9	9.9	-0.04
Psycholeptics	5.1	2.7	0.13	4.4	5.4	-0.04
Psychostimulants, agents used for ADHD and nootropics	0.3	0.2	0.01	0.3	0.3	-0.01

Table S6.50. Selected baseline characteristics for Germany IQVIA, for the medium-term risk of alcohol misuse or dependence

**Characteristic**	**Before propensity score matching**			**After propensity score matching**		
	**Targets, %**	**Comparators, %**	**Standardized difference**	**Targets, %**	**Comparators, %**	**Standardized difference**
**Age group (years)**						
0-4	1.4	2.4	-0.07	1.5	2.8	-0.09
5-9	2.2	3.2	-0.06	2.2	3.5	-0.08
10-14	2.8	3.3	-0.03	2.7	3.5	-0.04
15-19	5	3.5	0.07	5	5.4	-0.02
20-24	7.6	4	0.16	7.6	7.5	0
25-29	7.4	4.3	0.13	7.2	6.8	0.02
30-34	8	5.1	0.11	7.7	7.3	0.01
35-39	7.6	5.2	0.1	7.6	7.6	0
40-44	8.3	5.4	0.11	8.4	8	0.01
45-49	8.1	6	0.08	8.3	7.6	0.03
50-54	9.9	8.4	0.05	10.2	9.4	0.03
55-59	9.6	9.5	0.01	10	9.3	0.02
60-64	7	8.7	-0.06	7.4	7.2	0.01
65-69	3.6	7.8	-0.18	3.7	3.5	0.01
70-74	2.9	6.9	-0.19	3	2.8	0.01
75-79	2.5	6.4	-0.19	2.4	2.5	-0.01
80-84	2.7	6.2	-0.17	2.4	2.4	0
85-89	2	2.7	-0.05	1.5	1.8	-0.02
90-94	1.1	1	0.01	0.8	0.9	-0.01
95-99	0.3	0.2	0.04	0.2	0.2	0
**Sex**						
Female	53.8	57.2	-0.07	53.9	53.4	0.01

Table S6.50. *Continued.* Selected baseline characteristics for Germany IQVIA, for the medium-term risk of alcohol misuse or dependence

Characteristic	Before propensity score matching			After propensity score matching		
	Targets, %	Comparators, %	Standardized difference	Targets, %	Comparators, %	Standardized difference
**Medical history (general)**						
Acute respiratory disease	59	7.8	1.29	60.2	62.5	-0.05
Chronic liver disease	0.2	0.1	0.02	0.1	0.2	-0.02
Chronic obstructive lung disease	2.8	1.3	0.11	2.9	4.3	-0.07
Crohn’s disease	0.2	0.1	0.02	0.2	0.3	-0.02
Dementia	1.2	0.5	0.08	0.9	1.3	-0.03
Depressive disorder	7.2	2.8	0.2	7.3	7.8	-0.02
Diabetes mellitus	4	2.2	0.1	4.2	5.3	-0.05
Gastroesophageal reflux disease	1.4	0.5	0.1	1.5	1.7	-0.02
Gastrointestinal hemorrhage	0.6	0.2	0.06	0.6	0.6	0
Hyperlipidemia	5.5	2.3	0.16	5.7	6.8	-0.04
Hypertensive disorder	11.7	5.9	0.2	12.2	14.6	-0.07
Lesion of liver	0.2	0.1	0.01	0.1	0.3	-0.03
Obesity	2.6	1.1	0.11	2.7	3.1	-0.02
Osteoarthritis	5.4	3.4	0.1	5.7	6.3	-0.03
Pneumonia	3	0.4	0.2	2.9	3.2	-0.02
Psoriasis	0.6	0.5	0.01	0.7	0.8	-0.01
Renal impairment	1.6	0.7	0.08	1.4	1.9	-0.04
Rheumatoid arthritis	0.6	0.4	0.03	0.7	0.8	-0.02
Schizophrenia	0.1	0.1	0.01	0.1	0.2	-0.01
Ulcerative colitis	0.2	0.1	0.02	0.2	0.3	-0.03
Urinary tract infectious disease	4.1	1.4	0.16	4.1	5.3	-0.05

Table S6.50. *Continued.* Selected baseline characteristics for Germany IQVIA, for the medium-term risk of alcohol misuse or dependence

**Characteristic**	**Before propensity score matching**			**After propensity score matching**		
	**Targets, %**	**Comparators, %**	**Standardized difference**	**Targets, %**	**Comparators, %**	**Standardized difference**
**Medical history (cardiovascular disease)**						
Atrial fibrillation	0.8	0.5	0.03	0.8	1	-0.02
Cerebrovascular disease	1.2	0.8	0.04	1.2	1.4	-0.02
Coronary arteriosclerosis	1	0.6	0.04	1	1.3	-0.02
Heart disease	7.7	3.9	0.16	7.8	9.3	-0.05
Heart failure	1.8	0.9	0.08	1.7	2.3	-0.04
Ischemic heart disease	2.4	1.2	0.09	2.4	3.1	-0.04
Peripheral vascular disease	0.9	0.6	0.04	0.8	1.2	-0.04
Pulmonary embolism	0.3	0.1	0.04	0.3	0.4	-0.03
Venous thrombosis	0.7	0.3	0.06	0.7	1.1	-0.04
**Medical history (neoplasms)**						
Malignant lymphoma	0.1	0.1	0.02	0.2	0.2	-0.01
Malignant neoplastic disease	1.9	2.1	-0.01	2	2.6	-0.04
Malignant tumor of breast	0.3	0.4	-0.02	0.3	0.3	-0.01
Malignant tumor of urinary bladder	0.1	0.1	0	0.1	0.1	0
Primary malignant neoplasm of prostate	0.2	0.3	-0.03	0.2	0.2	-0.01
**Medication use**						
Agents acting on the renin-angiotensin system	18.9	8.6	0.3	19.9	21.7	-0.04
Antibacterials for systemic use	26.6	9.1	0.47	27.9	33.3	-0.12
Antidepressants	6.2	3.6	0.12	6.3	7.9	-0.06
Antiepileptics	2.3	1.5	0.06	2.2	2.8	-0.04
Antiinflammatory and antirheumatic products	32.6	11.6	0.52	34.3	35.8	-0.03
Antineoplastic agents	1	0.9	0.01	1	1.2	-0.02
Antipsoriatics	0.2	0.2	0	0.2	0.3	-0.01
Antithrombotic agents	10	4.7	0.2	9.9	11.9	-0.07

Table S6.50. *Continued.* Selected baseline characteristics for Germany IQVIA, for the medium-term risk of alcohol misuse or dependence

**Characteristic**	**Before propensity score matching**			**After propensity score matching**		
	**Targets, %**	**Comparators, %**	**Standardized difference**	**Targets, %**	**Comparators, %**	**Standardized difference**
**Medication use**						
Beta blocking agents	12.1	6	0.22	12.5	14.2	-0.05
Calcium channel blockers	8.4	3.8	0.19	8.8	9.8	-0.03
Diuretics	11	5.3	0.21	11	13	-0.06
Drugs for acid-related disorders	20.1	6.8	0.4	21	23.8	-0.07
Drugs for obstructive airway diseases	15.1	7.2	0.26	16	20.4	-0.12
Drugs used in diabetes	5.8	3	0.14	6	6.7	-0.03
Immunosuppressants	0.6	0.7	-0.02	0.6	0.8	-0.02
Lipid modifying agents	9.4	4.6	0.19	9.8	11.5	-0.06
Opioids	8.8	2.6	0.27	9	10.3	-0.04
Psycholeptics	5.1	2.7	0.12	5	6.2	-0.05
Psychostimulants, agents used for ADHD and nootropics	0.3	0.2	0.01	0.3	0.4	-0.01

Table S6.51. Selected baseline characteristics for Germany IQVIA, for the medium-term risk of substance misuse or dependence

Characteristic	Before propensity score matching			After propensity score matching		
	**Targets, %**	**Comparators, %**	**Standardized difference**	**Targets, %**	**Comparators, %**	**Standardized difference**
**Age group (years)**						
0-4	1.4	2.4	-0.07	1.5	2.8	-0.09
5-9	2.2	3.2	-0.06	2.3	3.6	-0.08
10-14	2.8	3.3	-0.03	2.8	3.6	-0.04
15-19	5	3.5	0.07	5	5.5	-0.02
20-24	7.6	4	0.16	7.6	7.5	0
25-29	7.4	4.3	0.13	7.3	6.8	0.02
30-34	8	5.2	0.11	7.7	7.4	0.01
40-44	8.3	5.4	0.12	8.3	8	0.01
45-49	8.1	5.9	0.09	8.3	7.5	0.03
50-54	9.9	8.3	0.06	10.2	9.3	0.03
55-59	9.6	9.4	0.01	10	9.2	0.03
60-64	7	8.6	-0.06	7.4	7.1	0.01
65-69	3.6	7.8	-0.18	3.7	3.5	0.01
70-74	2.9	6.9	-0.19	2.9	2.8	0.01
75-79	2.5	6.3	-0.19	2.4	2.5	-0.01
80-84	2.7	6.2	-0.17	2.4	2.4	0
85-89	2	2.7	-0.05	1.6	1.8	-0.02
90-94	1.1	1	0.01	0.8	1	-0.01
95-99	0.3	0.1	0.04	0.2	0.2	0
**Sex**						
Female	53.8	57	-0.06	54.1	53.5	0.01

Table S6.51. *Continued.* Selected baseline characteristics for Germany IQVIA, for the medium-term risk of substance misuse or dependence

Characteristic	Before propensity score matching			After propensity score matching		
	Targets, %	Comparators, %	Standardized difference	Targets, %	Comparators, %	Standardized difference
**Medical history (general)**						
Acute respiratory disease	59	7.8	1.29	60.2	62.4	-0.04
Chronic liver disease	0.2	0.1	0.03	0.1	0.2	-0.01
Chronic obstructive lung disease	2.8	1.3	0.11	2.8	4.1	-0.07
Crohn’s disease	0.2	0.1	0.02	0.2	0.3	-0.02
Dementia	1.2	0.5	0.08	0.9	1.3	-0.03
Depressive disorder	7.2	2.8	0.2	7.2	7.5	-0.01
Diabetes mellitus	4	2.3	0.1	4.1	5.2	-0.05
Gastroesophageal reflux disease	1.4	0.5	0.1	1.4	1.6	-0.02
Hyperlipidemia	5.5	2.4	0.16	5.6	6.5	-0.04
Hypertensive disorder	11.7	6	0.2	12.1	14.4	-0.07
Lesion of liver	0.2	0.1	0.01	0.1	0.2	-0.03
Obesity	2.6	1.1	0.11	2.6	2.9	-0.02
Osteoarthritis	5.4	3.4	0.1	5.6	6.2	-0.02
Pneumonia	3	0.4	0.2	2.9	3.2	-0.02
Psoriasis	0.6	0.5	0.02	0.7	0.8	-0.01
Renal impairment	1.6	0.7	0.08	1.4	1.8	-0.04
Rheumatoid arthritis	0.6	0.4	0.03	0.7	0.8	-0.01
Schizophrenia	0.1	0.1	0.01	0.1	0.1	-0.01
Ulcerative colitis	0.2	0.1	0.02	0.2	0.3	-0.03
Urinary tract infectious disease	4.1	1.5	0.16	4.1	5.2	-0.05

Table S6.51. *Continued.* Selected baseline characteristics for Germany IQVIA, for the medium-term risk of substance misuse or dependence

**Characteristic**	**Before propensity score matching**			**After propensity score matching**		
	**Targets, %**	**Comparators, %**	**Standardized difference**	**Targets, %**	**Comparators, %**	**Standardized difference**
**Medical history (cardiovascular disease)**						
Atrial fibrillation	0.8	0.5	0.03	0.7	0.9	-0.02
Cerebrovascular disease	1.2	0.7	0.05	1.1	1.4	-0.02
Coronary arteriosclerosis	1	0.6	0.04	1	1.2	-0.02
Heart disease	7.7	4	0.16	7.6	9.1	-0.05
Heart failure	1.8	0.9	0.08	1.6	2.2	-0.04
Ischemic heart disease	2.4	1.2	0.09	2.3	3	-0.04
Peripheral vascular disease	0.9	0.5	0.04	0.8	1.1	-0.04
Pulmonary embolism	0.3	0.1	0.04	0.3	0.4	-0.03
Venous thrombosis	0.7	0.3	0.06	0.7	1.1	-0.04
**Medical history (neoplasms)**						
Malignant lymphoma	0.1	0.1	0.02	0.1	0.2	-0.01
Malignant neoplastic disease	1.9	2.1	-0.01	1.9	2.5	-0.04
Malignant tumor of breast	0.3	0.4	-0.01	0.3	0.3	-0.01
Malignant tumor of colon	0.1	0.1	0	0.1	0.1	-0.01
Malignant tumor of urinary bladder	0.1	0.1	0	0.1	0.1	0
Primary malignant neoplasm of prostate	0.2	0.3	-0.03	0.2	0.2	-0.01
**Medication use**						
Agents acting on the renin-angiotensin system	18.9	8.7	0.3	19.9	21.5	-0.04
Antibacterials for systemic use	26.6	9.1	0.47	27.8	33.1	-0.11
Antidepressants	6.2	3.5	0.12	6.3	7.7	-0.06
Antiepileptics	2.3	1.5	0.06	2.2	2.8	-0.04
Antiinflammatory and antirheumatic products	32.6	11.8	0.52	34.2	35.6	-0.03
Antineoplastic agents	1	0.9	0.01	1	1.2	-0.02
Antipsoriatics	0.2	0.2	0	0.2	0.3	-0.01
Antithrombotic agents	10	4.6	0.21	9.8	11.8	-0.06

Table S6.51. *Continued.* Selected baseline characteristics for Germany IQVIA, for the medium-term risk of substance misuse or dependence

Characteristic	Before propensity score matching			After propensity score matching		
	**Targets, %**	**Comparators, %**	**Standardized difference**	**Targets, %**	**Comparators, %**	**Standardized difference**
**Medication use**						
Beta blocking agents	12.1	5.9	0.22	12.5	14	-0.05
Calcium channel blockers	8.4	3.9	0.19	8.8	9.7	-0.03
Diuretics	11	5.2	0.21	11	12.9	-0.06
Drugs for acid-related disorders	20.1	6.8	0.4	20.9	23.6	-0.06
Drugs for obstructive airway diseases	15.1	7.2	0.25	16	20.2	-0.11
Drugs used in diabetes	5.8	3	0.14	5.9	6.5	-0.03
Immunosuppressants	0.6	0.7	-0.02	0.6	0.8	-0.02
Lipid modifying agents	9.4	4.7	0.18	9.7	11.3	-0.05
Opioids	8.8	2.6	0.27	9	10.1	-0.04
Psycholeptics	5.1	2.7	0.12	4.9	6.1	-0.05
Psychostimulants, agents used for ADHD and nootropics	0.3	0.2	0	0.3	0.3	-0.01

Table S6.52. Selected baseline characteristics for Germany IQVIA, for the medium-term risk of bipolar disorders

Characteristic	Before propensity score matching			After propensity score matching		
	Targets, %	Comparators, %	Standardized difference	Targets, %	Comparators, %	Standardized difference
**Age group (years)**						
0-4	1.4	2.4	-0.07	1.5	2.7	-0.09
5-9	2.2	3.2	-0.06	2.2	3.5	-0.08
10-14	2.8	3.3	-0.03	2.7	3.5	-0.04
15-19	5	3.5	0.07	5	5.4	-0.02
20-24	7.6	4	0.16	7.6	7.4	0
25-29	7.4	4.3	0.13	7.2	6.7	0.02
30-34	8	5.1	0.11	7.7	7.3	0.01
35-39	7.6	5.4	0.09	7.6	7.6	0
40-44	8.3	5.5	0.11	8.4	8	0.01
45-49	8.1	6	0.08	8.3	7.6	0.03
50-54	9.9	8.3	0.06	10.2	9.4	0.03
55-59	9.6	9.4	0.01	10	9.3	0.02
60-64	7	8.7	-0.06	7.5	7.2	0.01
65-69	3.6	7.8	-0.18	3.7	3.5	0.01
70-74	2.9	6.7	-0.18	3	2.8	0.01
75-79	2.5	6.3	-0.19	2.4	2.5	-0.01
80-84	2.7	6.2	-0.17	2.5	2.4	0.01
85-89	2	2.7	-0.05	1.5	1.8	-0.02
90-94	1.1	1	0.01	0.8	0.9	-0.01
95-99	0.3	0.2	0.03	0.2	0.2	0
**Sex**						
Female	53.8	57.2	-0.07	53.8	53.2	0.01

Table S6.52. *Continued.* Selected baseline characteristics for Germany IQVIA, for the medium-term risk of bipolar disorders

Characteristic	Before propensity score matching			After propensity score matching		
	**Targets, %**	**Comparators, %**	**Standardized difference**	**Targets, %**	**Comparators, %**	**Standardized difference**
**Medical history (general)**						
Acute respiratory disease	59	7.8	1.29	60.2	62.5	-0.05
Chronic liver disease	0.2	0.1	0.03	0.2	0.2	-0.02
Chronic obstructive lung disease	2.8	1.2	0.11	3	4.4	-0.07
Crohn's disease	0.2	0.1	0.02	0.2	0.3	-0.02
Dementia	1.2	0.5	0.07	0.9	1.3	-0.03
Depressive disorder	7.2	2.7	0.21	7.4	7.9	-0.02
Diabetes mellitus	4	2.2	0.1	4.2	5.3	-0.06
Gastroesophageal reflux disease	1.4	0.5	0.09	1.5	1.7	-0.02
Gastrointestinal hemorrhage	0.6	0.2	0.06	0.6	0.6	0
Hyperlipidemia	5.5	2.4	0.16	5.8	6.8	-0.04
Hypertensive disorder	11.7	5.8	0.21	12.3	14.7	-0.07
Lesion of liver	0.2	0.1	0.02	0.2	0.3	-0.03
Obesity	2.6	1.1	0.11	2.7	3.1	-0.03
Osteoarthritis	5.4	3.4	0.1	5.7	6.3	-0.03
Pneumonia	3	0.4	0.2	2.9	3.2	-0.02
Psoriasis	0.6	0.5	0.01	0.7	0.8	-0.01
Renal impairment	1.6	0.7	0.08	1.4	1.9	-0.04
Rheumatoid arthritis	0.6	0.5	0.02	0.7	0.8	-0.01
Schizophrenia	0.1	0.1	0.01	0.1	0.2	-0.01
Ulcerative colitis	0.2	0.1	0.02	0.2	0.3	-0.03
Urinary tract infectious disease	4.1	1.5	0.16	4.1	5.3	-0.05

Table S6.52. *Continued.* Selected baseline characteristics for Germany IQVIA, for the medium-term risk of bipolar disorders

**Characteristic**	**Before propensity score matching**			**After propensity score matching**		
	**Targets, %**	**Comparators, %**	**Standardized difference**	**Targets, %**	**Comparators, %**	**Standardized difference**
**Medical history (cardiovascular disease)**						
Atrial fibrillation	0.8	0.5	0.03	0.8	1	-0.02
Cerebrovascular disease	1.2	0.7	0.05	1.2	1.4	-0.02
Coronary arteriosclerosis	1	0.6	0.04	1	1.3	-0.02
Heart disease	7.7	3.9	0.16	7.8	9.3	-0.05
Heart failure	1.8	0.9	0.08	1.7	2.3	-0.04
Ischemic heart disease	2.4	1.2	0.09	2.4	3.1	-0.04
Peripheral vascular disease	0.9	0.6	0.04	0.8	1.2	-0.04
Pulmonary embolism	0.3	0.1	0.04	0.3	0.4	-0.03
Venous thrombosis	0.7	0.3	0.06	0.7	1.1	-0.04
**Medical history (neoplasms)**						
Malignant lymphoma	0.1	0.1	0.02	0.2	0.2	-0.01
Malignant neoplastic disease	1.9	2.1	-0.01	2	2.6	-0.04
Malignant tumor of breast	0.3	0.3	-0.01	0.3	0.3	-0.01
Malignant tumor of colon	0.1	0.1	0	0.1	0.1	-0.01
Malignant tumor of urinary bladder	0.1	0.1	-0.01	0.1	0.1	0
Primary malignant neoplasm of prostate	0.2	0.3	-0.03	0.2	0.2	0
**Medication use**						
Agents acting on the renin-angiotensin system	18.9	8.6	0.3	20	21.8	-0.04
Antibacterials for systemic use	26.6	9.1	0.47	27.9	33.3	-0.12
Antidepressants	6.2	3.5	0.12	6.4	7.9	-0.06
Antiepileptics	2.3	1.5	0.06	2.3	2.8	-0.04
Antiinflammatory and antirheumatic products	32.6	11.6	0.52	34.3	35.9	-0.03
Antineoplastic agents	1	0.9	0.01	1	1.2	-0.02
Antipsoriatics	0.2	0.3	0	0.2	0.3	-0.01
Antithrombotic agents	10	4.6	0.21	9.9	11.9	-0.06

Table S6.52. *Continued.* Selected baseline characteristics for Germany IQVIA, for the medium-term risk of bipolar disorders

**Characteristic**	**Before propensity score matching**			**After propensity score matching**		
	**Targets, %**	**Comparators, %**	**Standardized difference**	**Targets, %**	**Comparators, %**	**Standardized difference**
**Medication use**						
Beta blocking agents	12.1	5.9	0.22	12.5	14.3	-0.05
Calcium channel blockers	8.4	3.9	0.19	8.8	9.8	-0.03
Diuretics	11	5.2	0.21	11.1	13.1	-0.06
Drugs for acid-related disorders	20.1	6.7	0.4	21.1	23.9	-0.07
Drugs for obstructive airway diseases	15.1	7.2	0.26	16	20.5	-0.12
Drugs used in diabetes	5.8	3	0.14	5.9	6.7	-0.03
Immunosuppressants	0.6	0.8	-0.02	0.6	0.8	-0.02
Lipid modifying agents	9.4	4.6	0.19	9.8	11.5	-0.06
Opioids	8.8	2.6	0.27	9.1	10.3	-0.04
Psycholeptics	5.1	2.8	0.12	5	6.2	-0.05
Psychostimulants, agents used for ADHD and nootropics	0.3	0.2	0.01	0.3	0.4	-0.01

Table S6.53. Selected baseline characteristics for Germany IQVIA, for the medium-term risk of psychoses

Characteristic	Before propensity score matching			After propensity score matching		
	Targets, %	Comparators, %	Standardized difference	Targets, %	Comparators, %	Standardized difference
**Age group (years)**						
0-4	1.4	2.4	-0.07	1.5	2.8	-0.09
5-9	2.2	3.2	-0.06	2.2	3.5	-0.08
10-14	2.8	3.2	-0.02	2.7	3.5	-0.04
15-19	5	3.5	0.07	5	5.4	-0.02
20-24	7.6	4	0.16	7.6	7.4	0
25-29	7.4	4.2	0.13	7.2	6.8	0.02
30-34	8	5.2	0.11	7.7	7.4	0.01
40-44	8.3	5.5	0.11	8.4	8	0.01
45-49	8.1	6	0.08	8.3	7.6	0.03
50-54	9.9	8.2	0.06	10.2	9.4	0.03
55-59	9.6	9.5	0	10	9.3	0.02
60-64	7	8.6	-0.06	7.5	7.2	0.01
65-69	3.6	7.8	-0.18	3.7	3.5	0.01
70-74	2.9	6.8	-0.19	3	2.8	0.01
75-79	2.5	6.3	-0.19	2.4	2.5	-0.01
80-84	2.7	6.1	-0.17	2.4	2.3	0.01
85-89	2	2.8	-0.05	1.5	1.8	-0.02
90-94	1.1	1	0.01	0.8	0.9	-0.01
95-99	0.3	0.2	0.03	0.2	0.2	0
**Sex**						
Female	53.8	57.2	-0.07	53.8	53.2	0.01

Table S6.53. *Continued.* Selected baseline characteristics for Germany IQVIA, for the medium-term risk of psychoses

Characteristic	Before propensity score matching			After propensity score matching		
	Targets, %	Comparators, %	Standardized difference	Targets, %	Comparators, %	Standardized difference
**Medical history (general)**						
Acute respiratory disease	59	7.9	1.29	60.2	62.5	-0.05
Chronic liver disease	0.2	0.1	0.02	0.2	0.2	-0.02
Chronic obstructive lung disease	2.8	1.3	0.11	3	4.3	-0.07
Crohn’s disease	0.2	0.1	0.02	0.2	0.3	-0.02
Dementia	1.2	0.5	0.07	0.9	1.2	-0.03
Depressive disorder	7.2	2.8	0.2	7.3	7.8	-0.02
Diabetes mellitus	4	2.2	0.1	4.1	5.3	-0.06
Gastroesophageal reflux disease	1.4	0.5	0.1	1.5	1.7	-0.02
Gastrointestinal hemorrhage	0.6	0.2	0.06	0.6	0.6	0
Hyperlipidemia	5.5	2.4	0.16	5.7	6.7	-0.04
Hypertensive disorder	11.7	5.9	0.2	12.2	14.6	-0.07
Lesion of liver	0.2	0.1	0.01	0.2	0.3	-0.03
Obesity	2.6	1.1	0.11	2.7	3.1	-0.02
Osteoarthritis	5.4	3.5	0.09	5.7	6.3	-0.03
Pneumonia	3	0.4	0.2	2.9	3.2	-0.02
Psoriasis	0.6	0.5	0.01	0.7	0.8	-0.01
Renal impairment	1.6	0.7	0.08	1.4	1.9	-0.04
Rheumatoid arthritis	0.6	0.4	0.02	0.7	0.8	-0.01
Ulcerative colitis	0.2	0.1	0.02	0.2	0.3	-0.03
Urinary tract infectious disease	4.1	1.5	0.16	4.1	5.2	-0.05

Table S6.53. *Continued.* Selected baseline characteristics for Germany IQVIA, for the medium-term risk of psychoses

Characteristic	Before propensity score matching			After propensity score matching		
	**Targets, %**	**Comparators, %**	**Standardized difference**	**Targets, %**	**Comparators, %**	**Standardized difference**
**Medical history (cardiovascular disease)**						
Atrial fibrillation	0.8	0.5	0.03	0.7	0.9	-0.02
Cerebrovascular disease	1.2	0.7	0.05	1.1	1.4	-0.02
Coronary arteriosclerosis	1	0.6	0.04	1	1.3	-0.03
Heart disease	7.7	4	0.16	7.7	9.2	-0.05
Heart failure	1.8	0.9	0.08	1.7	2.3	-0.04
Ischemic heart disease	2.4	1.3	0.09	2.4	3.1	-0.04
Peripheral vascular disease	0.9	0.5	0.04	0.9	1.2	-0.03
Pulmonary embolism	0.3	0.1	0.04	0.3	0.4	-0.03
Venous thrombosis	0.7	0.3	0.06	0.7	1.1	-0.04
**Medical history (neoplasms)**						
Malignant lymphoma	0.1	0.1	0.02	0.2	0.2	-0.01
Malignant neoplastic disease	1.9	2.1	-0.01	1.9	2.6	-0.04
Malignant tumor of breast	0.3	0.4	-0.02	0.3	0.3	-0.01
Malignant tumor of colon	0.1	0.1	0	0.1	0.1	-0.01
Malignant tumor of urinary bladder	0.1	0.1	-0.01	0.1	0.1	0
Primary malignant neoplasm of prostate	0.2	0.3	-0.03	0.2	0.2	-0.01
**Medication use**						
Agents acting on the renin-angiotensin system	18.9	8.7	0.3	19.9	21.7	-0.04
Antibacterials for systemic use	26.6	9.2	0.47	27.9	33.3	-0.12
Antidepressants	6.2	3.6	0.12	6.3	7.9	-0.06
Antiepileptics	2.3	1.5	0.06	2.3	2.8	-0.04
Antiinflammatory and antirheumatic products	32.6	11.7	0.52	34.2	35.9	-0.03
Antineoplastic agents	1	0.9	0.01	1	1.2	-0.02
Antipsoriatics	0.2	0.2	0	0.2	0.3	-0.01

Table S6.53. *Continued.* Selected baseline characteristics for Germany IQVIA, for the medium-term risk of psychoses

Characteristic	Before propensity score matching			After propensity score matching		
	**Targets, %**	**Comparators, %**	**Standardized difference**	**Targets, %**	**Comparators, %**	**Standardized difference**
**Medication use**						
Antithrombotic agents	10	4.6	0.21	9.9	11.9	-0.06
Beta blocking agents	12.1	6	0.21	12.5	14.2	-0.05
Calcium channel blockers	8.4	3.8	0.19	8.8	9.8	-0.03
Diuretics	11	5.2	0.21	11	13.1	-0.06
Drugs for acid-related disorders	20.1	6.8	0.4	21.1	23.8	-0.07
Drugs for obstructive airway diseases	15.1	7.2	0.25	16	20.5	-0.12
Drugs used in diabetes	5.8	3	0.14	5.9	6.6	-0.03
Immunosuppressants	0.6	0.7	-0.02	0.6	0.8	-0.02
Lipid modifying agents	9.4	4.7	0.19	9.7	11.4	-0.06
Opioids	8.8	2.6	0.27	9	10.2	-0.04
Psycholeptics	5.1	2.8	0.12	4.9	6.1	-0.05
Psychostimulants, agents used for ADHD and nootropics	0.3	0.2	0	0.3	0.4	-0.01

Table S6.54. Selected baseline characteristics for Germany IQVIA, for the medium-term risk of personality disorders

Characteristic	Before propensity score matching			After propensity score matching		
	**Targets, %**	**Comparators, %**	**Standardized difference**	**Targets, %**	**Comparators, %**	**Standardized difference**
**Age group (years)**						
0-4	1.4	2.3	-0.07	1.5	2.7	-0.09
5-9	2.2	3.3	-0.06	2.2	3.5	-0.08
10-14	2.8	3.3	-0.02	2.7	3.5	-0.04
15-19	5	3.5	0.07	5	5.4	-0.02
20-24	7.6	4	0.16	7.5	7.5	0
25-29	7.4	4.3	0.13	7.2	6.7	0.02
30-34	8	5.2	0.11	7.7	7.3	0.01
40-44	8.3	5.4	0.11	8.4	8	0.01
45-49	8.1	5.9	0.09	8.3	7.6	0.03
50-54	9.9	8.4	0.05	10.2	9.4	0.03
55-59	9.6	9.3	0.01	10	9.3	0.02
60-64	7	8.8	-0.07	7.4	7.2	0.01
65-69	3.6	7.8	-0.18	3.8	3.5	0.01
70-74	2.9	6.8	-0.19	3	2.8	0.01
75-79	2.5	6.4	-0.19	2.4	2.5	0
80-84	2.7	6.2	-0.17	2.4	2.4	0
85-89	2	2.7	-0.05	1.5	1.8	-0.02
90-94	1.1	1	0.01	0.8	0.9	-0.01
95-99	0.3	0.2	0.03	0.2	0.2	0
**Sex**						
Female	53.8	57.1	-0.07	53.8	53.2	0.01

Table S6.54. *Continued.* Selected baseline characteristics for Germany IQVIA, for the medium-term risk of personality disorders

Characteristic	Before propensity score matching			After propensity score matching		
	Targets, %	Comparators, %	Standardized difference	Targets, %	Comparators, %	Standardized difference
**Medical history (general)**						
Acute respiratory disease	59	7.8	1.29	60.2	62.5	-0.05
Chronic liver disease	0.2	0.1	0.02	0.2	0.2	-0.02
Chronic obstructive lung disease	2.8	1.2	0.11	3	4.4	-0.07
Crohn’s disease	0.2	0.1	0.02	0.2	0.3	-0.02
Dementia	1.2	0.5	0.08	0.9	1.2	-0.03
Depressive disorder	7.2	2.7	0.21	7.3	7.7	-0.02
Diabetes mellitus	4	2.2	0.11	4.1	5.3	-0.05
Gastroesophageal reflux disease	1.4	0.5	0.1	1.5	1.7	-0.02
Gastrointestinal hemorrhage	0.6	0.2	0.06	0.6	0.6	0
Hyperlipidemia	5.5	2.4	0.16	5.7	6.7	-0.04
Hypertensive disorder	11.7	6	0.2	12.3	14.6	-0.07
Lesion of liver	0.2	0.1	0.01	0.2	0.3	-0.03
Obesity	2.6	1.1	0.11	2.7	3	-0.02
Osteoarthritis	5.4	3.5	0.09	5.7	6.3	-0.02
Pneumonia	3	0.4	0.2	2.9	3.2	-0.02
Psoriasis	0.6	0.5	0.02	0.7	0.8	-0.01
Renal impairment	1.6	0.7	0.08	1.4	1.9	-0.04
Rheumatoid arthritis	0.6	0.4	0.03	0.7	0.8	-0.01
Schizophrenia	0.1	0.1	0	0.1	0.1	-0.01
Ulcerative colitis	0.2	0.1	0.02	0.2	0.3	-0.02
Urinary tract infectious disease	4.1	1.4	0.16	4.1	5.2	-0.05

Table S6.54. *Continued.* Selected baseline characteristics for Germany IQVIA, for the medium-term risk of personality disorders

Characteristic	Before propensity score matching			After propensity score matching		
	**Targets, %**	**Comparators, %**	**Standardized difference**	**Targets, %**	**Comparators, %**	**Standardized difference**
**Medical history (cardiovascular disease)**						
Atrial fibrillation	0.8	0.5	0.03	0.7	1	-0.02
Cerebrovascular disease	1.2	0.7	0.05	1.2	1.4	-0.02
Coronary arteriosclerosis	1	0.6	0.04	1	1.3	-0.02
Heart disease	7.7	4	0.16	7.8	9.3	-0.05
Heart failure	1.8	0.9	0.08	1.7	2.3	-0.04
Ischemic heart disease	2.4	1.3	0.08	2.4	3.1	-0.04
Peripheral vascular disease	0.9	0.5	0.04	0.8	1.2	-0.04
Pulmonary embolism	0.3	0.1	0.04	0.3	0.4	-0.03
Venous thrombosis	0.7	0.3	0.06	0.7	1.1	-0.04
**Medical history (neoplasms)**						
Malignant lymphoma	0.1	0.1	0.02	0.2	0.2	-0.01
Malignant neoplastic disease	1.9	2.1	-0.01	1.9	2.6	-0.04
Malignant tumor of breast	0.3	0.4	-0.02	0.3	0.3	-0.01
Malignant tumor of urinary bladder	0.1	0.1	-0.01	0.1	0.1	0
Primary malignant neoplasm of prostate	0.2	0.3	-0.03	0.2	0.2	0
**Medication use**						
Agents acting on the renin-angiotensin system	18.9	8.7	0.3	20	21.7	-0.04
Antibacterials for systemic use	26.6	9.2	0.47	27.9	33.2	-0.12
Antidepressants	6.2	3.5	0.12	6.3	7.8	-0.06
Antiepileptics	2.3	1.4	0.06	2.2	2.8	-0.04
Antiinflammatory and antirheumatic products	32.6	11.7	0.52	34.3	35.8	-0.03
Antineoplastic agents	1	0.9	0.01	1	1.2	-0.02
Antipsoriatics	0.2	0.3	0	0.2	0.3	-0.01
Antithrombotic agents	10	4.6	0.21	9.9	11.9	-0.07
Beta blocking agents	12.1	5.9	0.22	12.5	14.2	-0.05

Table S6.54. *Continued.* Selected baseline characteristics for Germany IQVIA, for the medium-term risk of personality disorders

**Characteristic**	**Before propensity score matching**			**After propensity score matching**		
	**Targets, %**	**Comparators, %**	**Standardized difference**	**Targets, %**	**Comparators, %**	**Standardized difference**
**Medication use**						
Calcium channel blockers	8.4	3.8	0.19	8.8	9.8	-0.03
Diuretics	11	5.2	0.21	11	13.1	-0.06
Drugs for acid-related disorders	20.1	6.9	0.4	21	23.9	-0.07
Drugs for obstructive airway diseases	15.1	7.2	0.26	16	20.5	-0.12
Drugs used in diabetes	5.8	3	0.14	5.9	6.6	-0.03
Immunosuppressants	0.6	0.7	-0.02	0.6	0.8	-0.02
Lipid modifying agents	9.4	4.7	0.18	9.8	11.5	-0.06
Opioids	8.8	2.6	0.27	9.1	10.2	-0.04
Psycholeptics	5.1	2.8	0.12	5	6.2	-0.05
Psychostimulants, agents used for ADHD and nootropics	0.3	0.2	0	0.3	0.4	-0.01

Table S6.55. Selected baseline characteristics for Germany IQVIA, for the medium-term risk of self-harm and suicide

**Characteristic**	**Before propensity score matching**			**After propensity score matching**		
	**Targets, %**	**Comparators, %**	**Standardized difference**	**Targets, %**	**Comparators, %**	**Standardized difference**
**Age group (years)**						
0-4	1.4	2.4	-0.07	1.5	2.7	-0.09
5-9	2.2	3.1	-0.06	2.2	3.5	-0.08
10-14	2.8	3.2	-0.02	2.7	3.5	-0.04
15-19	5	3.5	0.08	5	5.4	-0.02
20-24	7.6	4	0.15	7.5	7.4	0
25-29	7.4	4.3	0.13	7.2	6.7	0.02
30-34	8	5.1	0.12	7.7	7.3	0.01
35-39	7.6	5.3	0.1	7.6	7.6	0
40-44	8.3	5.6	0.11	8.4	8	0.01
45-49	8.1	5.9	0.09	8.3	7.6	0.03
50-54	9.9	8.4	0.05	10.2	9.4	0.03
55-59	9.6	9.4	0.01	10	9.3	0.02
60-64	7	8.7	-0.06	7.5	7.2	0.01
65-69	3.6	7.9	-0.18	3.7	3.5	0.01
70-74	2.9	6.8	-0.19	3	2.8	0.01
75-79	2.5	6.4	-0.19	2.4	2.5	0
80-84	2.7	6.1	-0.17	2.5	2.4	0
85-89	2	2.7	-0.05	1.5	1.8	-0.02
90-94	1.1	0.9	0.02	0.8	0.9	-0.01
95-99	0.3	0.2	0.03	0.2	0.2	0
**Sex**						
Female	53.8	57.2	-0.07	53.8	53.2	0.01

Table S6.55. *Continued.* Selected baseline characteristics for Germany IQVIA, for the medium-term risk of self-harm and suicide

Characteristic	Before propensity score matching			After propensity score matching		
	Targets, %	Comparators, %	Standardized difference	Targets, %	Comparators, %	Standardized difference
**Medical history (general)**						
Acute respiratory disease	59	7.8	1.29	60.2	62.5	-0.05
Chronic liver disease	0.2	0.1	0.02	0.2	0.2	-0.02
Chronic obstructive lung disease	2.8	1.3	0.11	3	4.4	-0.07
Crohn’s disease	0.2	0.1	0.02	0.2	0.3	-0.02
Dementia	1.2	0.5	0.08	0.9	1.3	-0.03
Depressive disorder	7.2	2.8	0.2	7.4	7.9	-0.02
Diabetes mellitus	4	2.3	0.1	4.2	5.3	-0.06
Gastroesophageal reflux disease	1.4	0.5	0.1	1.5	1.7	-0.02
Gastrointestinal hemorrhage	0.6	0.2	0.06	0.6	0.6	0
Hyperlipidemia	5.5	2.5	0.16	5.8	6.8	-0.04
Hypertensive disorder	11.7	6	0.2	12.3	14.7	-0.07
Lesion of liver	0.2	0.1	0.01	0.2	0.3	-0.03
Obesity	2.6	1.1	0.1	2.7	3.1	-0.02
Osteoarthritis	5.4	3.4	0.09	5.7	6.3	-0.03
Pneumonia	3	0.4	0.2	2.9	3.2	-0.02
Psoriasis	0.6	0.5	0.01	0.7	0.8	-0.01
Renal impairment	1.6	0.7	0.08	1.4	1.9	-0.04
Rheumatoid arthritis	0.6	0.4	0.03	0.7	0.8	-0.02
Schizophrenia	0.1	0.1	0	0.1	0.2	-0.01
Ulcerative colitis	0.2	0.1	0.02	0.2	0.3	-0.03
Urinary tract infectious disease	4.1	1.5	0.16	4.2	5.3	-0.05

Table S6.55. *Continued.* Selected baseline characteristics for Germany IQVIA, for the medium-term risk of self-harm and suicide

**Characteristic**	**Before propensity score matching**			**After propensity score matching**		
	**Targets, %**	**Comparators, %**	**Standardized difference**	**Targets, %**	**Comparators, %**	**Standardized difference**
**Medical history (cardiovascular disease)**						
Atrial fibrillation	0.8	0.5	0.03	0.8	1	-0.02
Cerebrovascular disease	1.2	0.7	0.05	1.2	1.4	-0.02
Coronary arteriosclerosis	1	0.6	0.04	1	1.3	-0.02
Heart disease	7.7	4	0.16	7.8	9.3	-0.05
Heart failure	1.8	0.9	0.08	1.7	2.3	-0.04
Ischemic heart disease	2.4	1.3	0.08	2.4	3.1	-0.04
Peripheral vascular disease	0.9	0.6	0.04	0.9	1.2	-0.04
Pulmonary embolism	0.3	0.1	0.04	0.3	0.4	-0.03
Venous thrombosis	0.7	0.3	0.06	0.7	1.1	-0.04
**Medical history (neoplasms)**						
Malignant lymphoma	0.1	0.1	0.02	0.2	0.2	-0.01
Malignant neoplastic disease	1.9	2.1	-0.01	2	2.6	-0.04
Malignant tumor of breast	0.3	0.4	-0.02	0.3	0.3	-0.01
Malignant tumor of colon	0.1	0.1	0	0.1	0.1	-0.01
Malignant tumor of urinary bladder	0.1	0.1	0	0.1	0.1	0
Primary malignant neoplasm of prostate	0.2	0.3	-0.03	0.2	0.2	-0.01
**Medication use**						
Agents acting on the renin-angiotensin system	18.9	8.8	0.3	20	21.8	-0.04
Antibacterials for systemic use	26.6	9.2	0.46	27.9	33.3	-0.12
Antidepressants	6.2	3.6	0.12	6.4	8	-0.06
Antiepileptics	2.3	1.4	0.06	2.3	2.9	-0.04
Antiinflammatory and antirheumatic products	32.6	11.8	0.52	34.3	35.9	-0.03
Antineoplastic agents	1	0.9	0.01	1	1.2	-0.02
Antipsoriatics	0.2	0.3	0	0.2	0.3	-0.01
Antithrombotic agents	10	4.6	0.21	9.9	12	-0.06

Table S6.55. *Continued.* Selected baseline characteristics for Germany IQVIA, for the medium-term risk of self-harm and suicide

**Characteristic**	**Before propensity score matching**			**After propensity score matching**		
	**Targets, %**	**Comparators, %**	**Standardized difference**	**Targets, %**	**Comparators, %**	**Standardized difference**
**Medication use**						
Beta blocking agents	12.1	6	0.21	12.5	14.3	-0.05
Calcium channel blockers	8.4	3.9	0.19	8.8	9.8	-0.03
Diuretics	11	5.3	0.21	11.1	13.2	-0.06
Drugs for acid-related disorders	20.1	6.8	0.4	21.1	23.9	-0.07
Drugs for obstructive airway diseases	15.1	7.2	0.26	16.1	20.5	-0.12
Drugs used in diabetes	5.8	3.1	0.13	6	6.7	-0.03
Immunosuppressants	0.6	0.7	-0.02	0.6	0.8	-0.02
Lipid modifying agents	9.4	4.7	0.18	9.8	11.5	-0.06
Opioids	8.8	2.6	0.27	9.1	10.3	-0.04
Psycholeptics	5.1	2.7	0.12	5	6.3	-0.06
Psychostimulants, agents used for ADHD and nootropics	0.3	0.2	0	0.3	0.4	-0.01

Table S6.56. Selected baseline characteristics for Germany IQVIA, for the medium-term risk of sleep disorders

Characteristic	Before propensity score matching			After propensity score matching		
	Targets, %	Comparators, %	Standardized difference	Targets, %	Comparators, %	Standardized difference
**Age group (years)**						
0-4	1.4	2.4	-0.07	1.5	2.8	-0.09
5-9	2.2	3.3	-0.07	2.3	3.7	-0.08
10-14	2.8	3.4	-0.03	2.9	3.7	-0.04
15-19	5	3.5	0.07	5.1	5.6	-0.02
20-24	7.6	3.9	0.16	7.8	7.7	0.01
25-29	7.4	4.3	0.13	7.4	6.9	0.02
30-34	8	5.2	0.11	7.8	7.5	0.01
40-44	8.3	5.4	0.12	8.5	8	0.02
45-49	8.1	6	0.08	8.3	7.5	0.03
50-54	9.9	8.3	0.06	10.1	9.3	0.03
55-59	9.6	9.4	0.01	9.8	9.2	0.02
60-64	7	8.6	-0.06	7.2	7	0.01
65-69	3.6	7.8	-0.18	3.6	3.4	0.01
70-74	2.9	6.8	-0.19	2.9	2.7	0.02
75-79	2.5	6.4	-0.19	2.3	2.4	-0.01
80-84	2.7	6.2	-0.17	2.3	2.2	0
85-89	2	2.7	-0.05	1.5	1.7	-0.02
90-94	1.1	1	0.02	0.7	0.9	-0.01
95-99	0.3	0.2	0.03	0.2	0.2	0
**Sex**						
Female	53.8	57.1	-0.07	53.7	53.3	0.01

Table S6.56. *Continued.* Selected baseline characteristics for Germany IQVIA, for the medium-term risk of sleep disorders

Characteristic	Before propensity score matching			After propensity score matching		
	Targets, %	Comparators, %	Standardized difference	Targets, %	Comparators, %	Standardized difference
**Medical history (general)**						
Acute respiratory disease	59	7.8	1.29	60	62.5	-0.05
Chronic liver disease	0.2	0.1	0.03	0.1	0.2	-0.02
Chronic obstructive lung disease	2.8	1.2	0.12	2.7	4	-0.07
Crohn’s disease	0.2	0.1	0.02	0.2	0.3	-0.02
Dementia	1.2	0.5	0.08	0.8	1.1	-0.03
Depressive disorder	7.2	2.7	0.2	6.5	6.8	-0.01
Diabetes mellitus	4	2.3	0.1	3.9	4.9	-0.05
Gastroesophageal reflux disease	1.4	0.5	0.09	1.4	1.5	-0.01
Gastrointestinal hemorrhage	0.6	0.2	0.06	0.5	0.6	-0.01
Hyperlipidemia	5.5	2.4	0.16	5.2	6.2	-0.04
Hypertensive disorder	11.7	5.9	0.21	11.4	13.5	-0.06
Lesion of liver	0.2	0.1	0.01	0.2	0.3	-0.02
Obesity	2.6	1.1	0.11	2.4	2.8	-0.03
Osteoarthritis	5.4	3.4	0.09	5.2	5.9	-0.03
Pneumonia	3	0.4	0.2	2.7	3.1	-0.02
Psoriasis	0.6	0.5	0.01	0.6	0.7	-0.01
Renal impairment	1.6	0.7	0.08	1.2	1.7	-0.04
Rheumatoid arthritis	0.6	0.4	0.03	0.6	0.8	-0.02
Schizophrenia	0.1	0.1	0.01	0.1	0.1	-0.01
Ulcerative colitis	0.2	0.1	0.01	0.1	0.3	-0.03
Urinary tract infectious disease	4.1	1.5	0.16	4	5	-0.05

Table S6.56. *Continued.* Selected baseline characteristics for Germany IQVIA, for the medium-term risk of sleep disorders

Characteristic	Before propensity score matching			After propensity score matching		
	Targets, %	Comparators, %	Standardized difference	Targets, %	Comparators, %	Standardized difference
**Medical history (cardiovascular disease)**						
Atrial fibrillation	0.8	0.5	0.04	0.7	0.8	-0.01
Cerebrovascular disease	1.2	0.7	0.05	1	1.2	-0.02
Coronary arteriosclerosis	1	0.6	0.04	0.9	1.2	-0.02
Heart disease	7.7	3.9	0.16	7.1	8.5	-0.05
Heart failure	1.8	0.9	0.08	1.4	2	-0.04
Ischemic heart disease	2.4	1.3	0.08	2.1	2.8	-0.04
Peripheral vascular disease	0.9	0.6	0.04	0.8	1.1	-0.03
Pulmonary embolism	0.3	0.1	0.05	0.3	0.4	-0.03
Venous thrombosis	0.7	0.3	0.06	0.7	1	-0.04
**Medical history (neoplasms)**						
Malignant lymphoma	0.1	0.1	0.02	0.1	0.2	-0.01
Malignant neoplastic disease	1.9	2.1	-0.01	1.8	2.4	-0.04
Malignant tumor of breast	0.3	0.4	-0.02	0.2	0.3	-0.01
Malignant tumor of urinary bladder	0.1	0.1	0	0.1	0.1	0
Primary malignant neoplasm of prostate	0.2	0.3	-0.03	0.2	0.2	0
**Medication use**						
Agents acting on the renin-angiotensin system	18.9	8.6	0.3	19.1	20.7	-0.04
Antibacterials for systemic use	26.6	9.2	0.47	27.2	32.8	-0.12
Antidepressants	6.2	3.5	0.13	5.6	6.9	-0.05
Antiepileptics	2.3	1.5	0.06	2.1	2.6	-0.04
Antiinflammatory and antirheumatic products	32.6	11.6	0.52	33.7	35.4	-0.04
Antineoplastic agents	1	0.9	0.01	1	1.2	-0.02
Antipsoriatics	0.2	0.3	0	0.2	0.3	-0.01
Antithrombotic agents	10	4.6	0.21	9.3	11.2	-0.06

Table S6.56. *Continued.* Selected baseline characteristics for Germany IQVIA, for the medium-term risk of sleep disorders

Characteristic	Before propensity score matching			After propensity score matching		
	Targets, %	Comparators, %	Standardized difference	Targets, %	Comparators, %	Standardized difference
**Medication use**						
Beta blocking agents	12.1	6	0.21	11.9	13.6	-0.05
Calcium channel blockers	8.4	3.8	0.19	8.3	9.2	-0.03
Diuretics	11	5.2	0.21	10.4	12.2	-0.06
Drugs for acid-related disorders	20.1	6.8	0.4	20.2	22.8	-0.06
Drugs for obstructive airway diseases	15.1	7.1	0.26	15.6	19.9	-0.11
Drugs used in diabetes	5.8	3	0.13	5.6	6.3	-0.03
Immunosuppressants	0.6	0.8	-0.02	0.6	0.8	-0.02
Lipid modifying agents	9.4	4.6	0.19	9.2	10.9	-0.06
Opioids	8.8	2.6	0.27	8.7	9.7	-0.03
Psycholeptics	5.1	2.7	0.12	3.8	4.7	-0.04
Psychostimulants, agents used for ADHD and nootropics	0.3	0.2	0.01	0.3	0.3	-0.01

Table S6.57. Selected baseline characteristics for Germany IQVIA, for the medium-term risk of dementia

Characteristic	Before propensity score matching			After propensity score matching		
	Targets, %	Comparators, %	Standardized difference	Targets, %	Comparators, %	Standardized difference
**Age group (years)**						
0-4	1.4	2.5	-0.08	1.5	2.8	-0.09
5-9	2.2	3.2	-0.06	2.3	3.6	-0.08
10-14	2.8	3.3	-0.03	2.8	3.5	-0.04
15-19	5	3.5	0.07	5	5.5	-0.02
20-24	7.6	4	0.16	7.6	7.6	0
25-29	7.4	4.2	0.13	7.3	6.8	0.02
30-34	8	5.2	0.11	7.8	7.5	0.01
35-39	7.6	5.4	0.09	7.7	7.7	0
40-44	8.3	5.4	0.11	8.5	8.1	0.01
45-49	8.1	6	0.08	8.4	7.7	0.03
50-54	9.9	8.3	0.06	10.3	9.5	0.03
55-59	9.6	9.4	0.01	10.1	9.5	0.02
60-64	7	8.6	-0.06	7.5	7.3	0.01
65-69	3.6	7.8	-0.18	3.7	3.5	0.01
70-74	2.9	6.9	-0.19	2.9	2.7	0.01
80-84	2.7	6.1	-0.17	2.2	2.1	0.01
85-89	2	2.7	-0.05	1.3	1.4	-0.01
90-94	1.1	1	0.01	0.6	0.7	-0.01
95-99	0.3	0.2	0.03	0.2	0.1	0.01
**Sex**						
Female	53.8	57.2	-0.07	53.6	53.1	0.01

Table S6.57. *Continued.* Selected baseline characteristics for Germany IQVIA, for the medium-term risk of dementia

Characteristic	Before propensity score matching			After propensity score matching		
	Targets, %	Comparators, %	Standardized difference	Targets, %	Comparators, %	Standardized difference
**Medical history (general)**						
Acute respiratory disease	59	7.8	1.29	60.4	62.8	-0.05
Chronic liver disease	0.2	0.1	0.03	0.1	0.2	-0.02
Chronic obstructive lung disease	2.8	1.3	0.11	2.9	4.3	-0.07
Crohn’s disease	0.2	0.1	0.02	0.2	0.3	-0.02
Depressive disorder	7.2	2.8	0.2	7.2	7.6	-0.02
Diabetes mellitus	4	2.2	0.1	3.9	5.1	-0.05
Gastroesophageal reflux disease	1.4	0.5	0.09	1.5	1.6	-0.01
Gastrointestinal hemorrhage	0.6	0.2	0.06	0.6	0.6	0
Hyperlipidemia	5.5	2.4	0.16	5.6	6.5	-0.04
Hypertensive disorder	11.7	5.9	0.2	11.9	14.1	-0.07
Lesion of liver	0.2	0.1	0.01	0.1	0.3	-0.03
Obesity	2.6	1.1	0.1	2.7	3.1	-0.02
Osteoarthritis	5.4	3.4	0.1	5.6	6.1	-0.02
Pneumonia	3	0.4	0.2	2.9	3.1	-0.01
Psoriasis	0.6	0.5	0.02	0.7	0.8	-0.01
Renal impairment	1.6	0.7	0.08	1.3	1.7	-0.03
Rheumatoid arthritis	0.6	0.4	0.03	0.6	0.8	-0.02
Schizophrenia	0.1	0.1	0.01	0.1	0.1	-0.01
Ulcerative colitis	0.2	0.1	0.02	0.2	0.3	-0.02
Urinary tract infectious disease	4.1	1.5	0.16	4	5.1	-0.05

Table S6.57. *Continued.* Selected baseline characteristics for Germany IQVIA, for the medium-term risk of dementia

Characteristic	Before propensity score matching			After propensity score matching		
	Targets, %	Comparators, %	Standardized difference	Targets, %	Comparators, %	Standardized difference
**Medical history (cardiovascular disease)**						
Atrial fibrillation	0.8	0.5	0.04	0.7	0.8	-0.02
Cerebrovascular disease	1.2	0.7	0.05	1	1.2	-0.02
Coronary arteriosclerosis	1	0.7	0.04	1	1.2	-0.02
Heart disease	7.7	3.9	0.16	7.4	8.7	-0.05
Heart failure	1.8	0.9	0.08	1.5	2	-0.04
Ischemic heart disease	2.4	1.3	0.09	2.3	2.9	-0.04
Peripheral vascular disease	0.9	0.5	0.04	0.8	1.1	-0.03
Pulmonary embolism	0.3	0.1	0.04	0.3	0.4	-0.02
Venous thrombosis	0.7	0.3	0.06	0.7	1	-0.04
**Medical history (neoplasms)**						
Malignant lymphoma	0.1	0.1	0.02	0.1	0.2	-0.01
Malignant neoplastic disease	1.9	2.1	-0.01	1.8	2.5	-0.04
Malignant tumor of breast	0.3	0.4	-0.02	0.3	0.3	-0.01
Malignant tumor of colon	0.1	0.1	0	0.1	0.1	-0.02
Malignant tumor of urinary bladder	0.1	0.1	-0.01	0.1	0.1	0.01
Primary malignant neoplasm of prostate	0.2	0.3	-0.03	0.2	0.2	0
**Medication use**						
Agents acting on the renin-angiotensin system	18.9	8.6	0.3	19.6	21.2	-0.04
Antibacterials for systemic use	26.6	9.1	0.47	27.8	33.2	-0.12
Antidepressants	6.2	3.5	0.13	6.1	7.7	-0.06
Antiepileptics	2.3	1.5	0.06	2.2	2.7	-0.04
Antiinflammatory and antirheumatic products	32.6	11.7	0.52	34.2	35.7	-0.03
Antineoplastic agents	1	0.9	0.01	1.1	1.2	-0.01
Antipsoriatics	0.2	0.3	0	0.2	0.3	-0.01
Antithrombotic agents	10	4.6	0.21	9.5	11.2	-0.06

Table S6.57. *Continued.* Selected baseline characteristics for Germany IQVIA, for the medium-term risk of dementia

Characteristic	Before propensity score matching			After propensity score matching		
	**Targets, %**	**Comparators, %**	**Standardized difference**	**Targets, %**	**Comparators, %**	**Standardized difference**
**Medication use**						
Beta blocking agents	12.1	6	0.21	12.1	13.7	-0.05
Calcium channel blockers	8.4	3.8	0.19	8.6	9.5	-0.03
Diuretics	11	5.2	0.21	10.6	12.4	-0.06
Drugs for acid-related disorders	20.1	6.8	0.4	20.8	23.4	-0.06
Drugs for obstructive airway diseases	15.1	7.2	0.25	16	20.4	-0.11
Drugs used in diabetes	5.8	3	0.14	5.8	6.4	-0.03
Immunosuppressants	0.6	0.7	-0.02	0.6	0.8	-0.02
Lipid modifying agents	9.4	4.7	0.18	9.6	11.1	-0.05
Opioids	8.8	2.6	0.27	8.9	10	-0.04
Psycholeptics	5.1	2.7	0.12	4.7	5.7	-0.05
Psychostimulants, agents used for ADHD and nootropics	0.3	0.3	0	0.3	0.3	-0.02

Table S6.58. Selected baseline characteristics for Germany IQVIA, for the medium-term risk of neurodevelopmental disorders

Characteristic	Before propensity score matching			After propensity score matching		
	Targets, %	Comparators, %	Standardized difference	Targets, %	Comparators, %	Standardized difference
**Age group (years)**						
0-4	1.4	2.4	-0.07	1.3	2.4	-0.08
45421	2.2	3.2	-0.06	1.9	3.1	-0.07
45579	2.8	3.3	-0.03	2.5	3.2	-0.04
15-19	5	3.5	0.07	4.9	5.3	-0.02
20-24	7.6	4	0.15	7.6	7.5	0
25-29	7.4	4.2	0.13	7.3	6.9	0.02
30-34	8	5.1	0.12	7.8	7.5	0.01
35-39	7.6	5.3	0.09	7.7	7.7	0
40-44	8.3	5.5	0.11	8.5	8.1	0.01
45-49	8.1	5.9	0.09	8.4	7.7	0.03
50-54	9.9	8.3	0.06	10.3	9.6	0.02
55-59	9.6	9.5	0.01	10.1	9.5	0.02
60-64	7	8.7	-0.06	7.5	7.3	0.01
65-69	3.6	7.9	-0.19	3.8	3.6	0.01
70-74	2.9	6.8	-0.18	3	2.8	0.01
75-79	2.5	6.4	-0.19	2.5	2.5	0
80-84	2.7	6.1	-0.17	2.5	2.4	0
85-89	2	2.7	-0.05	1.5	1.8	-0.02
90-94	1.1	1	0.01	0.8	0.9	-0.01
95-99	0.3	0.2	0.04	0.2	0.2	0
**Sex**						
Female	53.8	57	-0.06	53.9	53.5	0.01

Table S6.58. *Continued.* Selected baseline characteristics for Germany IQVIA, for the medium-term risk of neurodevelopmental disorders

Characteristic	Before propensity score matching			After propensity score matching		
	Targets, %	Comparators, %	Standardized difference	Targets, %	Comparators, %	Standardized difference
**Medical history (general)**						
Acute respiratory disease	59	7.7	1.29	60.1	62.3	-0.05
Chronic liver disease	0.2	0.1	0.03	0.2	0.3	-0.02
Chronic obstructive lung disease	2.8	1.2	0.11	2.9	4.3	-0.07
Crohn’s disease	0.2	0.1	0.02	0.2	0.3	-0.02
Dementia	1.2	0.5	0.07	0.9	1.2	-0.04
Depressive disorder	7.2	2.7	0.2	7.4	7.9	-0.02
Diabetes mellitus	4	2.2	0.1	4.2	5.4	-0.06
Gastroesophageal reflux disease	1.4	0.5	0.09	1.5	1.7	-0.02
Gastrointestinal hemorrhage	0.6	0.2	0.06	0.6	0.6	0
Hyperlipidemia	5.5	2.4	0.16	5.8	6.8	-0.04
Hypertensive disorder	11.7	5.8	0.21	12.3	14.9	-0.07
Lesion of liver	0.2	0.1	0.01	0.2	0.3	-0.03
Obesity	2.6	1.1	0.11	2.7	3	-0.02
Osteoarthritis	5.4	3.4	0.1	5.7	6.4	-0.03
Pneumonia	3	0.4	0.2	2.9	3.2	-0.02
Psoriasis	0.6	0.5	0.01	0.7	0.8	-0.01
Renal impairment	1.6	0.7	0.08	1.4	1.9	-0.04
Rheumatoid arthritis	0.6	0.4	0.03	0.7	0.8	-0.01
Schizophrenia	0.1	0.1	0.01	0.1	0.1	-0.01
Ulcerative colitis	0.2	0.1	0.02	0.2	0.3	-0.03
Urinary tract infectious disease	4.1	1.5	0.16	4.2	5.2	-0.05

Table S6.58. *Continued.* Selected baseline characteristics for Germany IQVIA, for the medium-term risk of neurodevelopmental disorders

Characteristic	Before propensity score matching			After propensity score matching		
	Targets, %	Comparators, %	Standardized difference	Targets, %	Comparators, %	Standardized difference
**Medical history (cardiovascular disease)**						
Atrial fibrillation	0.8	0.5	0.04	0.8	1	-0.02
Cerebrovascular disease	1.2	0.7	0.05	1.1	1.4	-0.02
Coronary arteriosclerosis	1	0.6	0.04	1	1.3	-0.03
Heart disease	7.7	4	0.16	7.8	9.3	-0.06
Heart failure	1.8	0.9	0.08	1.7	2.3	-0.04
Ischemic heart disease	2.4	1.3	0.08	2.4	3.1	-0.04
Peripheral vascular disease	0.9	0.5	0.04	0.9	1.2	-0.03
Pulmonary embolism	0.3	0.1	0.04	0.3	0.4	-0.03
Venous thrombosis	0.7	0.3	0.06	0.7	1.1	-0.04
**Medical history (neoplasms)**						
Malignant lymphoma	0.1	0.1	0.02	0.2	0.2	-0.01
Malignant neoplastic disease	1.9	2.1	-0.01	1.9	2.6	-0.04
Malignant tumor of breast	0.3	0.4	-0.02	0.3	0.3	-0.01
Malignant tumor of colon	0.1	0.1	0	0.1	0.1	-0.02
Malignant tumor of urinary bladder	0.1	0.1	-0.01	0.1	0.1	0
Primary malignant neoplasm of prostate	0.2	0.3	-0.03	0.2	0.2	-0.01
**Medication use**						
Agents acting on the renin-angiotensin system	18.9	8.7	0.3	20.1	22	-0.05
Antibacterials for systemic use	26.6	9.2	0.47	27.8	33.1	-0.11
Antidepressants	6.2	3.6	0.12	6.4	8	-0.06
Antiepileptics	2.3	1.5	0.06	2.2	2.8	-0.04
Antiinflammatory and antirheumatic products	32.6	11.7	0.52	34	35.6	-0.03
Antineoplastic agents	1	0.9	0.01	1.1	1.2	-0.02
Antipsoriatics	0.2	0.3	0	0.2	0.3	-0.01
Antithrombotic agents	10	4.6	0.21	9.9	12.1	-0.07

Table S6.58. *Continued.* Selected baseline characteristics for Germany IQVIA, for the medium-term risk of neurodevelopmental disorders

Characteristic	Before propensity score matching			After propensity score matching		
	Targets, %	Comparators, %	Standardized difference	Targets, %	Comparators, %	Standardized difference
**Medication use**						
Beta blocking agents	12.1	6	0.21	12.6	14.4	-0.05
Calcium channel blockers	8.4	3.9	0.19	8.9	9.9	-0.04
Diuretics	11	5.2	0.21	11.1	13.3	-0.07
Drugs for acid-related disorders	20.1	6.8	0.4	21.2	24.1	-0.07
Drugs for obstructive airway diseases	15.1	7.2	0.26	15.9	20.4	-0.12
Drugs used in diabetes	5.8	3	0.14	6	6.8	-0.03
Immunosuppressants	0.6	0.7	-0.02	0.6	0.8	-0.02
Lipid modifying agents	9.4	4.7	0.18	9.8	11.7	-0.06
Opioids	8.8	2.6	0.27	9.1	10.4	-0.04
Psycholeptics	5.1	2.8	0.12	5	6.2	-0.06
Psychostimulants, agents used for ADHD and nootropics	0.3	0.2	0	0.2	0.2	0

Table S6.59. Selected baseline characteristics for Germany IQVIA, for the medium-term risk of any of psychiatric and neuropsychiatric disorders

Characteristic	Before propensity score matching			After propensity score matching		
	Targets, %	Comparators, %	Standardized difference	Targets, %	Comparators, %	Standardized difference
**Age group (years)**						
0-4	1.4	2.4	-0.07	1.6	3	-0.09
5-9	2.2	3.2	-0.06	2.4	3.8	-0.08
10-14	2.8	3.4	-0.03	3	3.9	-0.05
15-19	5	3.5	0.07	5.4	6	-0.02
20-24	7.6	4	0.16	8	8.1	0
25-29	7.4	4.3	0.13	7.6	7	0.02
30-34	8	5.2	0.11	7.8	7.7	0
35-39	7.6	5.3	0.1	7.7	7.6	0
40-44	8.3	5.4	0.11	8.2	7.9	0.01
45-49	8.1	5.9	0.09	8.4	7.4	0.04
50-54	9.9	8.3	0.06	10.3	9.2	0.04
55-59	9.6	9.4	0.01	9.6	8.8	0.03
60-64	7	8.7	-0.06	7.2	6.9	0.01
65-69	3.6	7.8	-0.18	3.6	3.5	0
70-74	2.9	6.7	-0.18	3	2.7	0.02
75-79	2.5	6.3	-0.19	2.2	2.3	-0.01
80-84	2.7	6.3	-0.17	2	2	0
85-89	2	2.8	-0.06	1.3	1.4	-0.01
90-94	1.1	1	0.01	0.6	0.7	-0.01
95-99	0.3	0.2	0.04	0.2	0.1	0.01
**Sex**						
Female	53.8	57.2	-0.07	52.2	52	0

Table S6.59. *Continued.* Selected baseline characteristics for Germany IQVIA, for the medium-term risk of any of psychiatric and neuropsychiatric disorders

Characteristic	Before propensity score matching			After propensity score matching		
	Targets, %	Comparators, %	Standardized difference	Targets, %	Comparators, %	Standardized difference
**Medical history (general)**						
Acute respiratory disease	59	7.9	1.29	59.3	61.8	-0.05
Chronic liver disease	0.2	0.1	0.02	0.1	0.2	-0.03
Chronic obstructive lung disease	2.8	1.3	0.11	2.2	3.3	-0.07
Crohn’s disease	0.2	0.1	0.02	0.1	0.3	-0.03
Diabetes mellitus	4	2.2	0.1	3.2	4.2	-0.05
Gastroesophageal reflux disease	1.4	0.5	0.09	1.1	1.3	-0.01
Gastrointestinal hemorrhage	0.6	0.2	0.06	0.5	0.5	0
Hyperlipidemia	5.5	2.4	0.16	4.3	5.3	-0.05
Hypertensive disorder	11.7	5.9	0.2	9.8	11.7	-0.06
Lesion of liver	0.2	0.1	0.01	0.1	0.2	-0.03
Obesity	2.6	1.1	0.11	1.9	2.2	-0.03
Osteoarthritis	5.4	3.5	0.09	4.3	5	-0.03
Pneumonia	3	0.4	0.2	2.6	2.7	-0.01
Psoriasis	0.6	0.5	0.02	0.6	0.7	-0.01
Renal impairment	1.6	0.7	0.08	1	1.3	-0.03
Rheumatoid arthritis	0.6	0.4	0.03	0.5	0.7	-0.02
Ulcerative colitis	0.2	0.1	0.01	0.1	0.3	-0.04
Urinary tract infectious disease	4.1	1.4	0.16	3.5	4.5	-0.05

Table S6.59. *Continued.* Selected baseline characteristics for Germany IQVIA, for the medium-term risk of any of psychiatric and neuropsychiatric disorders

Characteristic	Before propensity score matching			After propensity score matching		
	Targets, %	Comparators, %	Standardized difference	Targets, %	Comparators, %	Standardized difference
**Medical history (cardiovascular disease)**						
Atrial fibrillation	0.8	0.5	0.03	0.6	0.7	-0.02
Cerebrovascular disease	1.2	0.7	0.05	0.8	0.9	-0.01
Coronary arteriosclerosis	1	0.6	0.04	0.8	1	-0.02
Heart disease	7.7	3.9	0.16	5.9	7	-0.05
Heart failure	1.8	0.9	0.08	1.1	1.5	-0.04
Ischemic heart disease	2.4	1.2	0.09	1.7	2.3	-0.04
Peripheral vascular disease	0.9	0.5	0.04	0.6	0.8	-0.03
Pulmonary embolism	0.3	0.1	0.04	0.2	0.3	-0.01
Venous thrombosis	0.7	0.3	0.06	0.6	0.9	-0.03
**Medical history (neoplasms)**						
Malignant lymphoma	0.1	0.1	0.02	0.1	0.2	0
Malignant neoplastic disease	1.9	2.1	-0.01	1.5	2.1	-0.04
Malignant tumor of breast	0.3	0.4	-0.02	0.2	0.3	-0.02
Malignant tumor of colon	0.1	0.1	0	0.1	0.1	-0.02
Malignant tumor of urinary bladder	0.1	0.1	-0.01	0.1	0.1	0
Primary malignant neoplasm of prostate	0.2	0.3	-0.03	0.1	0.1	-0.01
**Medication use**						
Agents acting on the renin-angiotensin system	18.9	8.7	0.3	18.1	19.3	-0.03
Antibacterials for systemic use	26.6	9.2	0.47	26	31.4	-0.12
Antidepressants	6.2	3.6	0.12	3	3.3	-0.02
Antiepileptics	2.3	1.5	0.06	1.7	2.1	-0.03
Antiinflammatory and antirheumatic products	32.6	11.7	0.52	31.9	33.9	-0.04
Antineoplastic agents	1	0.9	0.01	1	1.2	-0.02

Table S6.59. *Continued.* Selected baseline characteristics for Germany IQVIA, for the medium-term risk of any of psychiatric and neuropsychiatric disorders

**Characteristic**	**Before propensity score matching**			**After propensity score matching**		
	**Targets, %**	**Comparators, %**	**Standardized difference**	**Targets, %**	**Comparators, %**	**Standardized difference**
**Medication use**						
Antipsoriatics	0.2	0.2	0	0.2	0.2	-0.01
Antithrombotic agents	10	4.7	0.2	8.5	10	-0.05
Beta blocking agents	12.1	6	0.21	10.9	12.2	-0.04
Calcium channel blockers	8.4	3.9	0.19	7.8	8.5	-0.03
Diuretics	11	5.2	0.21	9.6	11.1	-0.05
Drugs for acid-related disorders	20.1	6.8	0.4	18.2	20.4	-0.06
Drugs for obstructive airway diseases	15.1	7.3	0.25	14.7	19	-0.11
Drugs used in diabetes	5.8	3	0.13	5.2	5.8	-0.03
Immunosuppressants	0.6	0.7	-0.02	0.6	0.8	-0.02
Lipid modifying agents	9.4	4.6	0.19	8.5	10.2	-0.06
Opioids	8.8	2.6	0.27	8	8.7	-0.03
Psycholeptics	5.1	2.8	0.12	2.4	2.8	-0.02
Psychostimulants, agents used for ADHD and nootropics	0.3	0.2	0.01	0.1	0.2	-0.01

Table S6.60. Selected baseline characteristics for Germany IQVIA, for the long-term risk of depression

Characteristic	Before propensity score matching			After propensity score matching		
	Targets, %	Comparators, %	Standardized difference	Targets, %	Comparators, %	Standardized difference
**Age group (years)**						
0-4	1.4	2.4	-0.07	1.8	3.4	-0.1
5-9	2.2	3.2	-0.06	2.6	3.8	-0.07
10-14	2.8	3.3	-0.03	3	3.7	-0.04
15-19	5	3.5	0.07	5.1	5.7	-0.03
20-24	7.6	4	0.16	7.6	7.8	-0.01
25-29	7.4	4.2	0.14	7.2	6.8	0.01
30-34	8	5.2	0.11	7.6	7.2	0.01
35-39	7.6	5.3	0.09	7.5	7.2	0.01
40-44	8.3	5.4	0.11	8.3	7.9	0.01
45-49	8.1	6	0.08	8.3	7.7	0.02
50-54	9.9	8.4	0.05	10.3	9.3	0.04
55-59	9.6	9.5	0	9.9	9.3	0.02
60-64	7	8.6	-0.06	7.3	6.8	0.02
65-69	3.6	7.8	-0.18	3.8	3.8	0
70-74	2.9	6.8	-0.18	3	2.7	0.02
75-79	2.5	6.3	-0.18	2.2	2.3	-0.01
80-84	2.7	6.3	-0.17	2.3	2.2	0
85-89	2	2.7	-0.05	1.3	1.6	-0.02
90-94	1.1	1	0.01	0.7	0.7	0
95-99	0.3	0.2	0.03	0.2	0.1	0.01
**Sex**						
Female	53.8	57	-0.06	52.4	51.7	0.01

Table S6.60. *Continued.* Selected baseline characteristics for Germany IQVIA, for the long-term risk of depression

Characteristic	Before propensity score matching			After propensity score matching		
	Targets, %	Comparators, %	Standardized difference	Targets, %	Comparators, %	Standardized difference
**Medical history (general)**						
Acute respiratory disease	59	7.9	1.29	60.1	62.9	-0.06
Chronic liver disease	0.2	0.1	0.03	0.1	0.2	-0.01
Chronic obstructive lung disease	2.8	1.2	0.11	2.6	3.7	-0.07
Crohn’s disease	0.2	0.1	0.02	0.1	0.3	-0.03
Dementia	1.2	0.5	0.07	0.6	0.9	-0.04
Diabetes mellitus	4	2.2	0.1	3.6	4.5	-0.04
Gastroesophageal reflux disease	1.4	0.5	0.1	1.3	1.4	-0.01
Gastrointestinal hemorrhage	0.6	0.2	0.06	0.6	0.5	0.01
Hyperlipidemia	5.5	2.4	0.16	5	6	-0.04
Hypertensive disorder	11.7	5.9	0.2	11	13	-0.06
Lesion of liver	0.2	0.1	0.01	0.1	0.2	-0.02
Obesity	2.6	1.1	0.11	2.2	2.2	0
Osteoarthritis	5.4	3.4	0.1	4.9	5.7	-0.04
Pneumonia	3	0.4	0.2	2.9	2.9	0
Psoriasis	0.6	0.5	0.01	0.6	0.7	-0.02
Renal impairment	1.6	0.7	0.08	1.2	1.6	-0.04
Rheumatoid arthritis	0.6	0.4	0.03	0.6	0.7	-0.02
Schizophrenia	0.1	0.1	0.01	0.1	0.1	-0.03
Ulcerative colitis	0.2	0.1	0.01	0.1	0.2	-0.03
Urinary tract infectious disease	4.1	1.5	0.16	3.8	4.9	-0.06

Table S6.60. *Continued.* Selected baseline characteristics for Germany IQVIA, for the long-term risk of depression

Characteristic	Before propensity score matching			After propensity score matching		
	Targets, %	Comparators, %	Standardized difference	Targets, %	Comparators, %	Standardized difference
**Medical history (cardiovascular disease)**						
Atrial fibrillation	0.8	0.5	0.04	0.6	0.8	-0.02
Cerebrovascular disease	1.2	0.7	0.05	0.9	1.2	-0.03
Coronary arteriosclerosis	1	0.6	0.04	0.9	1.3	-0.04
Heart disease	7.7	3.9	0.16	6.7	8	-0.05
Heart failure	1.8	0.9	0.08	1.4	1.8	-0.03
Ischemic heart disease	2.4	1.2	0.09	1.9	2.5	-0.04
Peripheral vascular disease	0.9	0.6	0.04	0.7	1	-0.03
Pulmonary embolism	0.3	0.1	0.04	0.2	0.4	-0.03
Venous thrombosis	0.7	0.3	0.06	0.7	1	-0.03
**Medical history (neoplasms)**						
Malignant lymphoma	0.1	0.1	0.02	0.1	0.2	-0.01
Malignant neoplastic disease	1.9	2.1	-0.01	1.6	2.2	-0.04
Malignant tumor of breast	0.3	0.4	-0.02	0.2	0.3	-0.02
Malignant tumor of colon	0.1	0.1	0	0.1	0.1	-0.02
Primary malignant neoplasm of prostate	0.2	0.3	-0.03	0.1	0.2	-0.01
**Medication use**						
Agents acting on the renin-angiotensin system	18.9	8.7	0.3	19.4	20.6	-0.03
Antibacterials for systemic use	26.6	9.2	0.47	27.4	33	-0.12
Antidepressants	6.2	3.6	0.12	3.6	4.3	-0.04
Antiepileptics	2.3	1.5	0.06	1.8	2.5	-0.04
Antiinflammatory and antirheumatic products	32.6	11.7	0.52	33.2	35.2	-0.04
Antineoplastic agents	1	0.9	0.01	1	1.2	-0.02
Antipsoriatics	0.2	0.2	0	0.2	0.3	-0.01
Antithrombotic agents	10	4.7	0.2	9.2	10.9	-0.06
Beta blocking agents	12.1	6	0.21	11.8	13.4	-0.05

Table S6.60. *Continued.* Selected baseline characteristics for Germany IQVIA, for the long-term risk of depression

Characteristic	Before propensity score matching			After propensity score matching		
	**Targets, %**	**Comparators, %**	**Standardized difference**	**Targets, %**	**Comparators, %**	**Standardized difference**
**Medication use**						
Calcium channel blockers	8.4	3.9	0.19	8.5	9.2	-0.02
Diuretics	11	5.2	0.21	10.4	11.9	-0.05
Drugs for acid-related disorders	20.1	6.8	0.4	19.2	22	-0.07
Drugs for obstructive airway diseases	15.1	7.2	0.25	15.6	19.7	-0.11
Drugs used in diabetes	5.8	3	0.14	5.6	6.1	-0.02
Immunosuppressants	0.6	0.7	-0.02	0.6	0.8	-0.03
Lipid modifying agents	9.4	4.7	0.18	9.2	10.9	-0.06
Opioids	8.8	2.7	0.27	8.2	9.4	-0.04
Psycholeptics	5.1	2.7	0.12	3.8	4.7	-0.04
Psychostimulants, agents used for ADHD and nootropics	0.3	0.2	0.01	0.2	0.3	-0.02

Table S6.61. Selected baseline characteristics for Germany IQVIA, for the long-term risk of anxiety disorders

**Characteristic**	**Before propensity score matching**			**After propensity score matching**		
	**Targets, %**	**Comparators, %**	**Standardized difference**	**Targets, %**	**Comparators, %**	**Standardized difference**
**Age group (years)**						
0-4	1.4	2.4	-0.07	1.6	3.3	-0.1
5-9	2.2	3.2	-0.06	2.4	3.6	-0.07
10-14	2.8	3.2	-0.02	2.8	3.6	-0.04
15-19	5	3.5	0.07	5	5.5	-0.02
20-24	7.6	4	0.16	7.6	7.6	0
25-29	7.4	4.3	0.13	7.1	6.7	0.02
30-34	8	5.1	0.12	7.3	7.1	0.01
35-39	7.6	5.3	0.1	7.4	7.1	0.01
40-44	8.3	5.5	0.11	8.4	7.9	0.01
45-49	8.1	5.9	0.09	8.4	7.7	0.02
50-54	9.9	8.3	0.05	10.3	9.5	0.03
55-59	9.6	9.5	0	9.8	9.5	0.01
60-64	7	8.8	-0.07	7.5	6.9	0.02
65-69	3.6	7.8	-0.18	3.9	3.9	0
70-74	2.9	6.8	-0.18	3.1	2.8	0.01
75-79	2.5	6.4	-0.19	2.5	2.4	0
80-84	2.7	6.1	-0.17	2.4	2.4	0
85-89	2	2.7	-0.05	1.5	1.7	-0.02
90-94	1.1	1	0.01	0.7	0.8	0
95-99	0.3	0.2	0.03	0.2	0.1	0.01
**Sex**						
Female	53.8	57.2	-0.07	52.7	52	0.02

Table S6.61. *Continued.* Selected baseline characteristics for Germany IQVIA, for the long-term risk of anxiety disorders

Characteristic	Before propensity score matching			After propensity score matching		
	**Targets, %**	**Comparators, %**	**Standardized difference**	**Targets, %**	**Comparators, %**	**Standardized difference**
**Medical history (general)**						
Acute respiratory disease	59	7.9	1.29	60	62.9	-0.06
Chronic liver disease	0.2	0.1	0.03	0.1	0.2	-0.02
Chronic obstructive lung disease	2.8	1.3	0.11	3.1	4	-0.05
Crohn's disease	0.2	0.1	0.02	0.2	0.3	-0.02
Dementia	1.2	0.5	0.07	0.9	1.1	-0.03
Depressive disorder	7.2	2.8	0.2	6.1	5.3	0.03
Diabetes mellitus	4	2.3	0.1	4.1	4.8	-0.04
Gastroesophageal reflux disease	1.4	0.5	0.1	1.4	1.5	-0.01
Gastrointestinal hemorrhage	0.6	0.2	0.06	0.6	0.5	0
Hyperlipidemia	5.5	2.4	0.16	5.5	6.5	-0.04
Hypertensive disorder	11.7	5.9	0.2	11.8	13.8	-0.06
Lesion of liver	0.2	0.1	0.01	0.1	0.2	-0.02
Obesity	2.6	1.1	0.1	2.5	2.4	0
Osteoarthritis	5.4	3.4	0.1	5.2	6	-0.04
Pneumonia	3	0.4	0.2	2.8	3	-0.01
Psoriasis	0.6	0.5	0.01	0.6	0.8	-0.02
Renal impairment	1.6	0.7	0.08	1.4	1.8	-0.04
Rheumatoid arthritis	0.6	0.5	0.02	0.6	0.8	-0.02
Schizophrenia	0.1	0.1	0	0.1	0.2	-0.02
Ulcerative colitis	0.2	0.1	0.02	0.1	0.2	-0.03
Urinary tract infectious disease	4.1	1.5	0.16	4.1	5	-0.04

Table S6.61. *Continued.* Selected baseline characteristics for Germany IQVIA, for the long-term risk of anxiety disorders

Characteristic	Before propensity score matching			After propensity score matching		
	Targets, %	Comparators, %	Standardized difference	Targets, %	Comparators, %	Standardized difference
**Medical history (cardiovascular disease)**						
Atrial fibrillation	0.8	0.5	0.03	0.7	0.8	-0.01
Cerebrovascular disease	1.2	0.7	0.05	1.1	1.3	-0.02
Coronary arteriosclerosis	1	0.7	0.04	1	1.4	-0.04
Heart disease	7.7	4	0.16	7.5	8.5	-0.04
Heart failure	1.8	0.9	0.08	1.6	1.9	-0.02
Ischemic heart disease	2.4	1.2	0.09	2.3	2.7	-0.02
Peripheral vascular disease	0.9	0.6	0.03	0.8	1.1	-0.03
Pulmonary embolism	0.3	0.1	0.04	0.3	0.4	-0.03
Venous thrombosis	0.7	0.3	0.06	0.7	1	-0.04
**Medical history (neoplasms)**						
Malignant lymphoma	0.1	0.1	0.02	0.1	0.2	-0.01
Malignant neoplastic disease	1.9	2.1	-0.01	1.8	2.3	-0.04
Malignant tumor of breast	0.3	0.4	-0.02	0.2	0.3	-0.01
Malignant tumor of urinary bladder	0.1	0.1	-0.01	0.1	0.1	-0.01
**Medication use**						
Agents acting on the renin-angiotensin system	18.9	8.6	0.3	20	21.4	-0.03
Antibacterials for systemic use	26.6	9.2	0.47	27.7	33.3	-0.12
Antidepressants	6.2	3.6	0.12	5.3	6.2	-0.04
Antiepileptics	2.3	1.5	0.06	2.2	2.7	-0.04
Antiinflammatory and antirheumatic products	32.6	11.6	0.52	34.6	35.7	-0.02
Antineoplastic agents	1	0.9	0.01	1	1.3	-0.02
Antipsoriatics	0.2	0.3	0	0.2	0.3	0
Antithrombotic agents	10	4.6	0.21	9.7	11.5	-0.06
Beta blocking agents	12.1	6	0.21	12.5	13.8	-0.04
Calcium channel blockers	8.4	3.8	0.19	9	9.5	-0.02

Table S6.61. *Continued.* Selected baseline characteristics for Germany IQVIA, for the long-term risk of anxiety disorders

**Characteristic**	**Before propensity score matching**			**After propensity score matching**		
	**Targets, %**	**Comparators, %**	**Standardized difference**	**Targets, %**	**Comparators, %**	**Standardized difference**
**Medication use**						
Diuretics	11	5.2	0.21	11	12.5	-0.04
Drugs for acid-related disorders	20.1	6.8	0.4	20.5	23.1	-0.06
Drugs for obstructive airway diseases	15.1	7.3	0.25	16.2	20	-0.1
Drugs used in diabetes	5.8	3.1	0.13	6	6.4	-0.02
Immunosuppressants	0.6	0.8	-0.02	0.7	0.8	-0.02
Lipid modifying agents	9.4	4.7	0.18	9.9	11.5	-0.05
Opioids	8.8	2.7	0.27	8.7	9.9	-0.04
Psycholeptics	5.1	2.7	0.12	4.2	5.1	-0.04
Psychostimulants, agents used for ADHD and nootropics	0.3	0.2	0	0.3	0.3	-0.01

Table S6.62. Selected baseline characteristics for Germany IQVIA, for the long-term risk of alcohol misuse or dependence

Characteristic	Before propensity score matching			After propensity score matching		
	Targets, %	Comparators, %	Standardized difference	Targets, %	Comparators, %	Standardized difference
**Age group (years)**						
0-4	1.4	2.4	-0.07	1.5	3	-0.1
5-9	2.2	3.3	-0.06	2.3	3.3	-0.06
10-14	2.8	3.3	-0.03	2.7	3.4	-0.04
15-19	5	3.5	0.07	4.9	5.4	-0.03
20-24	7.6	4	0.16	7.5	7.6	0
25-29	7.4	4.3	0.13	7.1	6.8	0.01
30-34	8	5.2	0.11	7.6	7.1	0.02
35-39	7.6	5.3	0.1	7.6	7.3	0.01
40-44	8.3	5.5	0.11	8.5	8.1	0.02
45-49	8.1	6	0.08	8.4	7.9	0.02
50-54	9.9	8.3	0.05	10.4	9.6	0.03
55-59	9.6	9.4	0.01	10.1	9.7	0.01
60-64	7	8.7	-0.06	7.5	7	0.02
65-69	3.6	7.8	-0.18	3.8	3.8	0
70-74	2.9	6.9	-0.19	3	2.7	0.02
85-89	2	2.7	-0.05	1.4	1.7	-0.02
90-94	1.1	1	0.02	0.7	0.8	-0.01
95-99	0.3	0.2	0.03	0.2	0.1	0.01
**Sex**						
Female	53.8	57.2	-0.07	54	53.4	0.01

Table S6.62. *Continued.* Selected baseline characteristics for Germany IQVIA, for the long-term risk of alcohol misuse or dependence

Characteristic	Before propensity score matching			After propensity score matching		
	**Targets, %**	**Comparators, %**	**Standardized difference**	**Targets, %**	**Comparators, %**	**Standardized difference**
**Medical history (general)**						
Acute respiratory disease	59	7.9	1.29	60.7	63.4	-0.06
Chronic liver disease	0.2	0.1	0.02	0.1	0.2	-0.01
Chronic obstructive lung disease	2.8	1.2	0.11	3	4.1	-0.06
Crohn's disease	0.2	0.1	0.02	0.2	0.3	-0.02
Dementia	1.2	0.5	0.08	0.9	1.2	-0.03
Depressive disorder	7.2	2.7	0.21	7.5	7.2	0.01
Diabetes mellitus	4	2.3	0.1	4.1	5	-0.04
Gastroesophageal reflux disease	1.4	0.5	0.09	1.5	1.6	-0.01
Gastrointestinal hemorrhage	0.6	0.2	0.06	0.6	0.6	0
Hyperlipidemia	5.5	2.4	0.16	5.8	6.8	-0.04
Hypertensive disorder	11.7	6	0.2	12.3	14.4	-0.06
Lesion of liver	0.2	0.1	0.01	0.1	0.2	-0.02
Obesity	2.6	1.1	0.11	2.7	2.6	0
Osteoarthritis	5.4	3.4	0.1	5.7	6.4	-0.03
Pneumonia	3	0.4	0.2	2.9	3	-0.01
Psoriasis	0.6	0.5	0.01	0.7	0.8	-0.02
Renal impairment	1.6	0.7	0.08	1.4	1.8	-0.04
Rheumatoid arthritis	0.6	0.4	0.03	0.7	0.9	-0.02
Schizophrenia	0.1	0.1	0.01	0.1	0.2	-0.01
Ulcerative colitis	0.2	0.1	0.02	0.2	0.3	-0.02
Urinary tract infectious disease	4.1	1.5	0.16	4.2	5.2	-0.05

Table S6.62. *Continued.* Selected baseline characteristics for Germany IQVIA, for the long-term risk of alcohol misuse or dependence

**Characteristic**	**Before propensity score matching**			**After propensity score matching**		
	**Targets, %**	**Comparators, %**	**Standardized difference**	**Targets, %**	**Comparators, %**	**Standardized difference**
**Medical history (cardiovascular disease)**						
Medical history: Cardiovascular disease						
Atrial fibrillation	0.8	0.5	0.04	0.7	0.9	-0.01
Cerebrovascular disease	1.2	0.7	0.05	1.1	1.4	-0.03
Coronary arteriosclerosis	1	0.6	0.04	1	1.4	-0.04
Heart disease	7.7	3.9	0.16	7.7	8.9	-0.04
Heart failure	1.8	0.8	0.09	1.6	2	-0.03
Ischemic heart disease	2.4	1.2	0.09	2.4	2.8	-0.03
Peripheral vascular disease	0.9	0.6	0.04	0.8	1.1	-0.03
Pulmonary embolism	0.3	0.1	0.04	0.3	0.4	-0.03
Venous thrombosis	0.7	0.3	0.06	0.7	1	-0.03
**Medical history (neoplasms)**						
Malignant lymphoma	0.1	0.1	0.02	0.2	0.2	-0.01
Malignant neoplastic disease	1.9	2.1	-0.01	1.9	2.4	-0.04
Malignant tumor of breast	0.3	0.4	-0.01	0.3	0.3	-0.01
Malignant tumor of urinary bladder	0.1	0.1	-0.01	0.1	0.1	0
Primary malignant neoplasm of prostate	0.2	0.3	-0.03	0.2	0.2	0
**Medication use**						
Agents acting on the renin-angiotensin system	18.9	8.7	0.3	20.3	21.7	-0.04
Antibacterials for systemic use	26.6	9.2	0.47	28.4	33.9	-0.12
Antidepressants	6.2	3.5	0.13	6.3	7.8	-0.06
Antiepileptics	2.3	1.4	0.06	2.2	2.8	-0.04
Antiinflammatory and antirheumatic products	32.6	11.7	0.52	34.9	35.9	-0.02
Antineoplastic agents	1	0.9	0.01	1.1	1.3	-0.02
Antipsoriatics	0.2	0.2	0	0.2	0.3	-0.01
Antithrombotic agents	10	4.6	0.21	9.7	11.5	-0.06

Table S6.62. *Continued.* Selected baseline characteristics for Germany IQVIA, for the long-term risk of alcohol misuse or dependence

Characteristic	Before propensity score matching			After propensity score matching		
	Targets, %	Comparators, %	Standardized difference	Targets, %	Comparators, %	Standardized difference
**Medication use**						
Beta blocking agents	12.1	5.9	0.22	12.6	14.2	-0.05
Calcium channel blockers	8.4	3.9	0.19	9	9.6	-0.02
Diuretics	11	5.2	0.21	11	12.6	-0.05
Drugs for acid-related disorders	20.1	6.7	0.4	21.1	23.8	-0.07
Drugs for obstructive airway diseases	15.1	7.2	0.25	16.4	20.4	-0.1
Drugs used in diabetes	5.8	3	0.14	6	6.5	-0.02
Immunosuppressants	0.6	0.7	-0.02	0.6	0.8	-0.02
Lipid modifying agents	9.4	4.7	0.18	9.9	11.5	-0.05
Opioids	8.8	2.6	0.27	9	10.3	-0.04
Psycholeptics	5.1	2.7	0.13	4.7	5.9	-0.05
Psychostimulants, agents used for ADHD and nootropics	0.3	0.2	0.01	0.3	0.3	-0.01

Table S6.63. Selected baseline characteristics for Germany IQVIA, for the long-term risk of substance misuse or dependence

Characteristic	Before propensity score matching			After propensity score matching		
	Targets, %	Comparators, %	Standardized difference	Targets, %	Comparators, %	Standardized difference
**Age group (years)**						
0-4	1.4	2.4	-0.07	1.6	3.1	-0.1
5-9	2.2	3.2	-0.06	2.3	3.4	-0.07
10-14	2.8	3.3	-0.03	2.8	3.5	-0.04
15-19	5	3.5	0.07	4.9	5.5	-0.03
20-24	7.6	4	0.15	7.5	7.7	0
25-29	7.4	4.3	0.13	7.1	6.9	0.01
30-34	8	5.2	0.11	7.6	7.1	0.02
35-39	7.6	5.3	0.1	7.6	7.3	0.01
40-44	8.3	5.5	0.11	8.5	8	0.02
45-49	8.1	6	0.08	8.3	7.8	0.02
50-54	9.9	8.4	0.05	10.4	9.5	0.03
55-59	9.6	9.3	0.01	10.2	9.6	0.02
60-64	7	8.6	-0.06	7.5	6.9	0.02
65-69	3.6	7.8	-0.18	3.8	3.8	0
70-74	2.9	6.8	-0.18	2.9	2.7	0.01
75-79	2.5	6.4	-0.19	2.4	2.4	0
85-89	2	2.7	-0.05	1.4	1.7	-0.02
90-94	1.1	1	0.01	0.7	0.8	-0.01
95-99	0.3	0.2	0.03	0.2	0.1	0.01
**Sex**						
Female	53.8	57.3	-0.07	54.2	53.6	0.01

Table S6.63. *Continued.* Selected baseline characteristics for Germany IQVIA, for the long-term risk of substance misuse or dependence

**Characteristic**	**Before propensity score matching**			**After propensity score matching**		
	**Targets, %**	**Comparators, %**	**Standardized difference**	**Targets, %**	**Comparators, %**	**Standardized difference**
**Medical history (general)**						
Acute respiratory disease	59	7.9	1.29	60.7	63.4	-0.06
Chronic liver disease	0.2	0.1	0.03	0.1	0.2	-0.01
Chronic obstructive lung disease	2.8	1.3	0.11	2.9	3.8	-0.05
Crohn’s disease	0.2	0.1	0.02	0.2	0.3	-0.02
Dementia	1.2	0.5	0.08	0.9	1.2	-0.03
Depressive disorder	7.2	2.8	0.2	7.4	6.9	0.02
Diabetes mellitus	4	2.3	0.1	4.1	4.8	-0.03
Gastroesophageal reflux disease	1.4	0.5	0.1	1.4	1.6	-0.01
Gastrointestinal hemorrhage	0.6	0.2	0.06	0.6	0.6	0
Hyperlipidemia	5.5	2.4	0.16	5.6	6.6	-0.04
Hypertensive disorder	11.7	5.9	0.2	12.2	14	-0.05
Lesion of liver	0.2	0.1	0.01	0.1	0.2	-0.02
Obesity	2.6	1.1	0.11	2.5	2.5	0
Osteoarthritis	5.4	3.4	0.1	5.6	6.3	-0.03
Pneumonia	3	0.4	0.2	2.9	3	0
Psoriasis	0.6	0.5	0.01	0.7	0.8	-0.02
Renal impairment	1.6	0.7	0.08	1.3	1.8	-0.04
Rheumatoid arthritis	0.6	0.4	0.03	0.7	0.8	-0.02
Schizophrenia	0.1	0.1	0.01	0.1	0.1	-0.01
Ulcerative colitis	0.2	0.1	0.02	0.2	0.3	-0.02
Urinary tract infectious disease	4.1	1.5	0.16	4.2	5.2	-0.05

Table S6.63. *Continued.* Selected baseline characteristics for Germany IQVIA, for the long-term risk of substance misuse or dependence

**Characteristic**	**Before propensity score matching**			**After propensity score matching**		
	**Targets, %**	**Comparators, %**	**Standardized difference**	**Targets, %**	**Comparators, %**	**Standardized difference**
**Medical history (cardiovascular disease)**						
Atrial fibrillation	0.8	0.5	0.04	0.7	0.8	-0.02
Cerebrovascular disease	1.2	0.7	0.05	1.1	1.4	-0.03
Coronary arteriosclerosis	1	0.6	0.04	1	1.4	-0.04
Heart disease	7.7	3.9	0.16	7.6	8.7	-0.04
Heart failure	1.8	0.9	0.08	1.5	2	-0.03
Ischemic heart disease	2.4	1.2	0.09	2.3	2.7	-0.03
Peripheral vascular disease	0.9	0.6	0.04	0.8	1.1	-0.03
Pulmonary embolism	0.3	0.1	0.04	0.3	0.4	-0.03
Venous thrombosis	0.7	0.3	0.06	0.7	1	-0.03
**Medical history (neoplasms)**						
Malignant lymphoma	0.1	0.1	0.02	0.1	0.2	-0.01
Malignant neoplastic disease	1.9	2.1	-0.01	1.8	2.3	-0.03
Malignant tumor of breast	0.3	0.4	-0.02	0.3	0.3	-0.01
Malignant tumor of colon	0.1	0.1	0	0.1	0.1	-0.01
Malignant tumor of urinary bladder	0.1	0.1	-0.01	0.1	0.1	0
Primary malignant neoplasm of prostate	0.2	0.3	-0.03	0.2	0.2	0
**Medication use**						
Agents acting on the renin-angiotensin system	18.9	8.6	0.3	20.3	21.5	-0.03
Antibacterials for systemic use	26.6	9.2	0.46	28.3	33.6	-0.12
Antidepressants	6.2	3.5	0.12	6.3	7.6	-0.05
Antiepileptics	2.3	1.4	0.06	2.1	2.7	-0.04
Antiinflammatory and antirheumatic products	32.6	11.7	0.52	34.8	35.6	-0.02
Antineoplastic agents	1	0.8	0.01	1.1	1.3	-0.02
Antipsoriatics	0.2	0.3	-0.01	0.2	0.3	-0.01
Antithrombotic agents	10	4.6	0.21	9.6	11.3	-0.06

Table S6.63. *Continued.* Selected baseline characteristics for Germany IQVIA, for the long-term risk of substance misuse or dependence

Characteristic	Before propensity score matching			After propensity score matching		
	**Targets, %**	**Comparators, %**	**Standardized difference**	**Targets, %**	**Comparators, %**	**Standardized difference**
**Medication use**						
Beta blocking agents	12.1	5.9	0.22	12.5	14	-0.04
Calcium channel blockers	8.4	3.9	0.19	9	9.5	-0.02
Diuretics	11	5.1	0.22	11	12.4	-0.05
Drugs for acid-related disorders	20.1	6.8	0.4	20.9	23.6	-0.06
Drugs for obstructive airway diseases	15.1	7.2	0.25	16.3	20.2	-0.1
Drugs used in diabetes	5.8	3	0.14	6	6.3	-0.01
Immunosuppressants	0.6	0.7	-0.02	0.6	0.8	-0.02
Lipid modifying agents	9.4	4.7	0.18	9.8	11.3	-0.05
Opioids	8.8	2.6	0.27	8.9	10.1	-0.04
Psycholeptics	5.1	2.7	0.12	4.6	5.8	-0.05
Psychostimulants, agents used for ADHD and nootropics	0.3	0.2	0.01	0.3	0.4	-0.01

Table S6.64. Selected baseline characteristics for Germany IQVIA, for the long-term risk of bipolar disorders

Characteristic	Before propensity score matching			After propensity score matching		
	Targets, %	Comparators, %	Standardized difference	Targets, %	Comparators, %	Standardized difference
**Age group (years)**						
0-4	1.4	2.3	-0.07	1.5	3	-0.1
5-9	2.2	3.3	-0.07	2.3	3.3	-0.06
10-14	2.8	3.3	-0.03	2.7	3.3	-0.04
15-19	5	3.5	0.07	4.9	5.4	-0.03
20-24	7.6	4	0.16	7.5	7.6	0
25-29	7.4	4.3	0.13	7.1	6.8	0.01
30-34	8	5.2	0.11	7.5	7.1	0.01
35-39	7.6	5.3	0.09	7.6	7.3	0.01
40-44	8.3	5.4	0.11	8.5	8.1	0.02
45-49	8.1	6	0.08	8.4	7.9	0.02
50-54	9.9	8.4	0.05	10.4	9.7	0.02
55-59	9.6	9.3	0.01	10.2	9.7	0.01
60-64	7	8.6	-0.06	7.6	7	0.02
65-69	3.6	7.8	-0.18	3.8	3.8	0
70-74	2.9	6.8	-0.18	3	2.7	0.02
75-79	2.5	6.3	-0.19	2.4	2.4	0
80-84	2.7	6.2	-0.17	2.4	2.4	0
85-89	2	2.7	-0.05	1.4	1.6	-0.02
90-94	1.1	1	0.01	0.7	0.7	-0.01
95-99	0.3	0.2	0.04	0.2	0.1	0.01
**Sex**						
Female	53.8	57.1	-0.07	53.9	53.2	0.01

Table S6.64. *Continued.* Selected baseline characteristics for Germany IQVIA, for the long-term risk of bipolar disorders

Characteristic	Before propensity score matching			After propensity score matching		
	Targets, %	Comparators, %	Standardized difference	Targets, %	Comparators, %	Standardized difference
**Medical history (general)**						
Acute respiratory disease	59	7.8	1.29	60.7	63.4	-0.06
Chronic liver disease	0.2	0.1	0.03	0.1	0.2	-0.01
Chronic obstructive lung disease	2.8	1.3	0.11	3	4.2	-0.06
Crohn’s disease	0.2	0.1	0.02	0.2	0.3	-0.02
Dementia	1.2	0.5	0.07	0.9	1.2	-0.03
Depressive disorder	7.2	2.8	0.2	7.5	7.3	0.01
Diabetes mellitus	4	2.3	0.1	4.1	5	-0.04
Gastroesophageal reflux disease	1.4	0.5	0.09	1.5	1.6	-0.01
Gastrointestinal hemorrhage	0.6	0.2	0.06	0.6	0.6	0
Hyperlipidemia	5.5	2.4	0.16	5.8	6.8	-0.04
Hypertensive disorder	11.7	5.9	0.2	12.3	14.5	-0.06
Lesion of liver	0.2	0.1	0.01	0.1	0.2	-0.02
Obesity	2.6	1.1	0.11	2.7	2.6	0
Osteoarthritis	5.4	3.5	0.09	5.7	6.5	-0.03
Pneumonia	3	0.4	0.2	2.9	3.1	-0.01
Psoriasis	0.6	0.5	0.01	0.7	0.8	-0.02
Renal impairment	1.6	0.7	0.08	1.4	1.8	-0.04
Rheumatoid arthritis	0.6	0.4	0.03	0.7	0.8	-0.02
Schizophrenia	0.1	0.1	0.01	0.1	0.2	-0.01
Ulcerative colitis	0.2	0.1	0.02	0.2	0.3	-0.02
Urinary tract infectious disease	4.1	1.5	0.16	4.2	5.3	-0.05

Table S6.64. *Continued.* Selected baseline characteristics for Germany IQVIA, for the long-term risk of bipolar disorders

Characteristic	Before propensity score matching			After propensity score matching		
	**Targets, %**	**Comparators, %**	**Standardized difference**	**Targets, %**	**Comparators, %**	**Standardized difference**
**Medical history (cardiovascular disease)**						
Atrial fibrillation	0.8	0.5	0.03	0.7	0.9	-0.01
Cerebrovascular disease	1.2	0.7	0.04	1.1	1.4	-0.03
Coronary arteriosclerosis	1	0.7	0.04	1	1.4	-0.04
Heart disease	7.7	4	0.16	7.8	9	-0.04
Heart failure	1.8	0.9	0.08	1.6	2	-0.03
Ischemic heart disease	2.4	1.3	0.08	2.4	2.8	-0.03
Peripheral vascular disease	0.9	0.5	0.04	0.8	1.1	-0.03
Pulmonary embolism	0.3	0.1	0.05	0.3	0.4	-0.03
Venous thrombosis	0.7	0.3	0.06	0.7	1	-0.03
**Medical history (neoplasms)**						
Malignant lymphoma	0.1	0.1	0.02	0.2	0.2	-0.01
Malignant neoplastic disease	1.9	2.1	-0.01	1.9	2.4	-0.04
Malignant tumor of breast	0.3	0.4	-0.02	0.3	0.3	-0.01
Malignant tumor of colon	0.1	0.1	0	0.1	0.1	-0.01
Malignant tumor of urinary bladder	0.1	0.1	-0.01	0.1	0.1	0
Primary malignant neoplasm of prostate	0.2	0.3	-0.03	0.2	0.2	0
**Medication use**						
Agents acting on the renin-angiotensin system	18.9	8.7	0.3	20.3	21.8	-0.04
Antibacterials for systemic use	26.6	9.1	0.47	28.4	33.8	-0.12
Antidepressants	6.2	3.5	0.12	6.4	7.9	-0.06
Antiepileptics	2.3	1.5	0.06	2.2	2.8	-0.04
Antiinflammatory and antirheumatic products	32.6	11.8	0.52	34.9	35.9	-0.02
Antineoplastic agents	1	0.9	0.01	1.1	1.3	-0.02
Antipsoriatics	0.2	0.3	0	0.2	0.3	-0.01
Antithrombotic agents	10	4.7	0.2	9.8	11.5	-0.06

Table S6.64. *Continued.* Selected baseline characteristics for Germany IQVIA, for the long-term risk of bipolar disorders

Characteristic	Before propensity score matching			After propensity score matching		
	Targets, %	Comparators, %	Standardized difference	Targets, %	Comparators, %	Standardized difference
**Medication use**						
Beta blocking agents	12.1	5.9	0.22	12.6	14.3	-0.05
Calcium channel blockers	8.4	3.9	0.19	9	9.7	-0.02
Diuretics	11	5.2	0.21	11	12.7	-0.05
Drugs for acid-related disorders	20.1	6.8	0.4	21.2	24	-0.07
Drugs for obstructive airway diseases	15.1	7.2	0.25	16.4	20.4	-0.1
Drugs used in diabetes	5.8	3	0.14	6	6.5	-0.02
Immunosuppressants	0.6	0.8	-0.02	0.6	0.8	-0.02
Lipid modifying agents	9.4	4.7	0.18	9.9	11.6	-0.06
Opioids	8.8	2.6	0.27	9	10.3	-0.04
Psycholeptics	5.1	2.8	0.12	4.8	6	-0.05
Psychostimulants, agents used for ADHD and nootropics	0.3	0.2	0.01	0.3	0.4	-0.01

Table S6.65. Selected baseline characteristics for Germany IQVIA, for the long-term risk of psychoses

Characteristic	Before propensity score matching			After propensity score matching		
	Targets, %	Comparators, %	Standardized difference	Targets, %	Comparators, %	Standardized difference
**Age group (years)**						
0-4	1.4	2.4	-0.07	1.5	3	-0.1
5-9	2.2	3.3	-0.07	2.3	3.3	-0.06
10-14	2.8	3.3	-0.03	2.7	3.4	-0.04
15-19	5	3.5	0.07	4.9	5.4	-0.03
20-24	7.6	4	0.16	7.5	7.6	0
25-29	7.4	4.2	0.14	7.1	6.8	0.01
30-34	8	5.2	0.11	7.5	7.1	0.01
35-39	7.6	5.4	0.09	7.6	7.3	0.01
40-44	8.3	5.5	0.11	8.5	8.1	0.02
45-49	8.1	6	0.08	8.4	7.9	0.02
50-54	9.9	8.3	0.06	10.5	9.7	0.03
55-59	9.6	9.4	0.01	10.2	9.7	0.01
60-64	7	8.6	-0.06	7.6	7	0.02
65-69	3.6	7.8	-0.18	3.8	3.8	0
70-74	2.9	6.8	-0.18	3	2.7	0.02
75-79	2.5	6.4	-0.19	2.4	2.3	0
80-84	2.7	6.1	-0.17	2.4	2.3	0
85-89	2	2.7	-0.05	1.4	1.6	-0.02
90-94	1.1	1	0.01	0.7	0.7	-0.01
95-99	0.3	0.1	0.04	0.2	0.1	0.01
**Sex**						
Female	53.8	57.1	-0.07	53.9	53.2	0.01

Table S6.65. *Continued.* Selected baseline characteristics for Germany IQVIA, for the long-term risk of psychoses

Characteristic	Before propensity score matching			After propensity score matching		
	**Targets, %**	**Comparators, %**	**Standardized difference**	**Targets, %**	**Comparators, %**	**Standardized difference**
**Medical history (general)**						
Acute respiratory disease	59	7.8	1.29	60.7	63.5	-0.06
Chronic liver disease	0.2	0.1	0.03	0.1	0.2	-0.01
Chronic obstructive lung disease	2.8	1.2	0.11	3	4.1	-0.06
Crohn’s disease	0.2	0.1	0.02	0.2	0.3	-0.02
Dementia	1.2	0.5	0.07	0.8	1.1	-0.03
Depressive disorder	7.2	2.8	0.2	7.5	7.2	0.01
Diabetes mellitus	4	2.2	0.1	4.1	5	-0.04
Gastroesophageal reflux disease	1.4	0.5	0.1	1.5	1.6	-0.01
Gastrointestinal hemorrhage	0.6	0.2	0.06	0.6	0.6	0
Hyperlipidemia	5.5	2.4	0.16	5.8	6.8	-0.04
Hypertensive disorder	11.7	6	0.2	12.3	14.4	-0.06
Lesion of liver	0.2	0.1	0.01	0.2	0.2	-0.02
Obesity	2.6	1.1	0.11	2.7	2.6	0
Osteoarthritis	5.4	3.4	0.1	5.7	6.4	-0.03
Pneumonia	3	0.4	0.2	2.9	3	-0.01
Psoriasis	0.6	0.5	0.01	0.7	0.8	-0.02
Renal impairment	1.6	0.7	0.08	1.3	1.8	-0.04
Rheumatoid arthritis	0.6	0.5	0.02	0.7	0.8	-0.02
Ulcerative colitis	0.2	0.1	0.01	0.2	0.3	-0.02
Urinary tract infectious disease	4.1	1.5	0.16	4.2	5.2	-0.05

Table S6.65. *Continued.* Selected baseline characteristics for Germany IQVIA, for the long-term risk of psychoses

**Characteristic**	**Before propensity score matching**			**After propensity score matching**		
	**Targets, %**	**Comparators, %**	**Standardized difference**	**Targets, %**	**Comparators, %**	**Standardized difference**
**Medical history (cardiovascular disease)**						
Atrial fibrillation	0.8	0.5	0.04	0.7	0.8	-0.01
Cerebrovascular disease	1.2	0.7	0.05	1.1	1.4	-0.02
Coronary arteriosclerosis	1	0.6	0.04	1	1.4	-0.04
Heart disease	7.7	3.9	0.16	7.7	8.9	-0.04
Heart failure	1.8	0.9	0.08	1.6	2	-0.03
Ischemic heart disease	2.4	1.2	0.09	2.4	2.8	-0.03
Peripheral vascular disease	0.9	0.6	0.04	0.8	1.1	-0.03
Pulmonary embolism	0.3	0.1	0.04	0.3	0.4	-0.03
Venous thrombosis	0.7	0.3	0.06	0.8	1	-0.03
**Medical history (neoplasms)**						
Malignant lymphoma	0.1	0.1	0.02	0.2	0.2	-0.01
Malignant neoplastic disease	1.9	2.1	-0.01	1.9	2.4	-0.04
Malignant tumor of breast	0.3	0.4	-0.02	0.3	0.3	-0.01
Malignant tumor of colon	0.1	0.1	0	0.1	0.1	-0.01
Malignant tumor of urinary bladder	0.1	0.1	-0.01	0.1	0.1	0
Primary malignant neoplasm of prostate	0.2	0.3	-0.03	0.2	0.2	0
**Medication use**						
Agents acting on the renin-angiotensin system	18.9	8.7	0.3	20.3	21.7	-0.04
Antibacterials for systemic use	26.6	9.2	0.47	28.3	33.8	-0.12
Antidepressants	6.2	3.6	0.12	6.3	7.8	-0.06
Antiepileptics	2.3	1.4	0.06	2.2	2.8	-0.04
Antiinflammatory and antirheumatic products	32.6	11.7	0.52	34.8	35.9	-0.02
Antineoplastic agents	1	0.9	0.01	1.1	1.3	-0.02
Antithrombotic agents	10	4.6	0.21	9.7	11.4	-0.06
Beta blocking agents	12.1	6	0.21	12.6	14.2	-0.05

Table S6.65. *Continued.* Selected baseline characteristics for Germany IQVIA, for the long-term risk of psychoses

Characteristic	Before propensity score matching			After propensity score matching		
	Targets, %	Comparators, %	Standardized difference	Targets, %	Comparators, %	Standardized difference
**Medication use**						
Calcium channel blockers	8.4	3.9	0.19	9	9.7	-0.02
Diuretics	11	5.2	0.21	11	12.6	-0.05
Drugs for acid-related disorders	20.1	6.8	0.4	21.1	23.8	-0.06
Drugs for obstructive airway diseases	15.1	7.2	0.25	16.4	20.4	-0.1
Drugs used in diabetes	5.8	3	0.14	6	6.4	-0.02
Immunosuppressants	0.6	0.7	-0.02	0.6	0.8	-0.02
Lipid modifying agents	9.4	4.7	0.18	9.8	11.5	-0.06
Opioids	8.8	2.7	0.27	8.9	10.2	-0.04
Psycholeptics	5.1	2.8	0.12	4.7	5.8	-0.05
Psychostimulants, agents used for ADHD and nootropics	0.3	0.2	0	0.3	0.4	-0.01

Table S6.66. Selected baseline characteristics for Germany IQVIA, for the long-term risk of personality disorders

Characteristic	Before propensity score matching			After propensity score matching		
	Targets, %	Comparators, %	Standardized difference	Targets, %	Comparators, %	Standardized difference
**Age group (years)**						
0-4	1.4	2.3	-0.07	1.5	3	-0.1
5-9	2.2	3.2	-0.06	2.3	3.3	-0.06
10-14	2.8	3.3	-0.03	2.7	3.4	-0.04
15-19	5	3.5	0.07	4.9	5.4	-0.02
20-24	7.6	4	0.15	7.4	7.6	-0.01
25-29	7.4	4.3	0.13	7.1	6.8	0.01
30-34	8	5.2	0.11	7.5	7.1	0.01
35-39	7.6	5.3	0.1	7.6	7.2	0.01
40-44	8.3	5.5	0.11	8.5	8.1	0.02
45-49	8.1	5.9	0.09	8.4	7.9	0.02
50-54	9.9	8.3	0.06	10.4	9.7	0.02
55-59	9.6	9.3	0.01	10.1	9.7	0.01
60-64	7	8.8	-0.07	7.6	7	0.02
65-69	3.6	7.9	-0.18	3.8	3.8	0
70-74	2.9	6.8	-0.18	3	2.7	0.02
75-79	2.5	6.4	-0.19	2.4	2.4	0
85-89	2	2.7	-0.05	1.4	1.6	-0.02
90-94	1.1	1	0.01	0.7	0.7	0
95-99	0.3	0.2	0.04	0.2	0.1	0.01
**Sex**						
Female	53.8	57.1	-0.07	53.9	53.2	0.01

Table S6.66. *Continued.* Selected baseline characteristics for Germany IQVIA, for the long-term risk of personality disorders

Characteristic	Before propensity score matching			After propensity score matching		
	Targets, %	Comparators, %	Standardized difference	Targets, %	Comparators, %	Standardized difference
**Medical history (general)**						
Acute respiratory disease	59	7.8	1.29	60.6	63.4	-0.06
Chronic liver disease	0.2	0.1	0.02	0.1	0.2	-0.01
Chronic obstructive lung disease	2.8	1.3	0.11	3	4.2	-0.06
Crohn’s disease	0.2	0.1	0.02	0.2	0.3	-0.02
Dementia	1.2	0.5	0.07	0.8	1.1	-0.03
Depressive disorder	7.2	2.8	0.2	7.4	7.1	0.01
Diabetes mellitus	4	2.3	0.1	4.1	5	-0.04
Gastroesophageal reflux disease	1.4	0.5	0.1	1.4	1.6	-0.01
Gastrointestinal hemorrhage	0.6	0.2	0.06	0.6	0.6	0
Hyperlipidemia	5.5	2.4	0.16	5.7	6.8	-0.04
Hypertensive disorder	11.7	5.9	0.2	12.3	14.4	-0.06
Lesion of liver	0.2	0.1	0.01	0.1	0.2	-0.02
Obesity	2.6	1.1	0.11	2.7	2.6	0
Osteoarthritis	5.4	3.4	0.1	5.7	6.4	-0.03
Pneumonia	3	0.4	0.2	2.9	3	-0.01
Psoriasis	0.6	0.5	0.01	0.7	0.8	-0.02
Renal impairment	1.6	0.7	0.08	1.4	1.8	-0.04
Rheumatoid arthritis	0.6	0.4	0.03	0.7	0.8	-0.02
Schizophrenia	0.1	0.1	0.01	0.1	0.2	-0.01
Ulcerative colitis	0.2	0.1	0.02	0.2	0.3	-0.02
Urinary tract infectious disease	4.1	1.5	0.16	4.2	5.2	-0.05

Table S6.66. *Continued.* Selected baseline characteristics for Germany IQVIA, for the long-term risk of personality disorders

Characteristic	Before propensity score matching			After propensity score matching		
	Targets, %	Comparators, %	Standardized difference	Targets, %	Comparators, %	Standardized difference
**Medical history (cardiovascular disease)**						
Atrial fibrillation	0.8	0.5	0.04	0.7	0.9	-0.01
Cerebrovascular disease	1.2	0.7	0.05	1.1	1.4	-0.02
Coronary arteriosclerosis	1	0.6	0.04	1	1.4	-0.04
Heart disease	7.7	4	0.16	7.7	8.9	-0.04
Heart failure	1.8	0.9	0.08	1.6	2	-0.03
Ischemic heart disease	2.4	1.3	0.09	2.4	2.8	-0.03
Peripheral vascular disease	0.9	0.6	0.04	0.8	1.1	-0.03
Pulmonary embolism	0.3	0.1	0.04	0.3	0.4	-0.03
Venous thrombosis	0.7	0.3	0.06	0.7	1	-0.03
**Medical history (neoplasms)**						
Malignant lymphoma	0.1	0.1	0.02	0.2	0.2	-0.01
Malignant neoplastic disease	1.9	2.1	-0.01	1.9	2.4	-0.04
Malignant tumor of breast	0.3	0.4	-0.01	0.3	0.3	-0.01
Malignant tumor of colon	0.1	0.1	0	0.1	0.1	-0.01
Malignant tumor of urinary bladder	0.1	0.1	0	0.1	0.1	0
Primary malignant neoplasm of prostate	0.2	0.3	-0.03	0.2	0.2	0
**Medication use**						
Agents acting on the renin-angiotensin system	18.9	8.7	0.3	20.3	21.8	-0.04
Antibacterials for systemic use	26.6	9.2	0.46	28.3	33.8	-0.12
Antidepressants	6.2	3.6	0.12	6.3	7.7	-0.06
Antiepileptics	2.3	1.5	0.06	2.1	2.8	-0.04
Antiinflammatory and antirheumatic products	32.6	11.7	0.52	34.9	35.8	-0.02
Antineoplastic agents	1	0.9	0.01	1.1	1.3	-0.02
Antipsoriatics	0.2	0.3	0	0.2	0.3	-0.01
Antithrombotic agents	10	4.6	0.21	9.7	11.5	-0.06

Table S6.66. *Continued.* Selected baseline characteristics for Germany IQVIA, for the long-term risk of personality disorders

Characteristic	Before propensity score matching			After propensity score matching		
	Targets, %	Comparators, %	Standardized difference	Targets, %	Comparators, %	Standardized difference
**Medication use**						
Beta blocking agents	12.1	6	0.21	12.6	14.3	-0.05
Calcium channel blockers	8.4	3.9	0.19	9	9.7	-0.02
Diuretics	11	5.3	0.21	11	12.6	-0.05
Drugs for acid-related disorders	20.1	6.8	0.4	21.1	23.9	-0.07
Drugs for obstructive airway diseases	15.1	7.2	0.25	16.4	20.3	-0.1
Drugs used in diabetes	5.8	3.1	0.13	6	6.5	-0.02
Immunosuppressants	0.6	0.7	-0.02	0.6	0.8	-0.02
Lipid modifying agents	9.4	4.7	0.18	9.9	11.5	-0.05
Opioids	8.8	2.7	0.27	9	10.3	-0.04
Psycholeptics	5.1	2.7	0.12	4.7	6	-0.06
Psychostimulants, agents used for ADHD and nootropics	0.3	0.3	0	0.3	0.3	-0.01

Table S6.67. Selected baseline characteristics for Germany IQVIA, for the long-term risk of self-harm and suicide

Characteristic	Before propensity score matching			After propensity score matching		
	Targets, %	Comparators, %	Standardized difference	Targets, %	Comparators, %	Standardized difference
**Age group (years)**						
0-4	1.4	2.4	-0.07	1.5	3	-0.1
5-9	2.2	3.3	-0.06	2.3	3.3	-0.06
10-14	2.8	3.3	-0.03	2.7	3.3	-0.04
15-19	5	3.6	0.07	4.9	5.4	-0.03
20-24	7.6	4	0.16	7.5	7.6	0
25-29	7.4	4.3	0.13	7.1	6.8	0.01
30-34	8	5.1	0.11	7.5	7.1	0.01
35-39	7.6	5.3	0.09	7.6	7.3	0.01
40-44	8.3	5.5	0.11	8.5	8.1	0.02
45-49	8.1	5.9	0.09	8.4	7.9	0.02
50-54	9.9	8.3	0.05	10.4	9.7	0.02
55-59	9.6	9.3	0.01	10.2	9.8	0.01
60-64	7	8.7	-0.06	7.6	7	0.02
65-69	3.6	7.8	-0.18	3.8	3.8	0
70-74	2.9	6.8	-0.18	3	2.7	0.02
75-79	2.5	6.4	-0.19	2.4	2.4	0
80-84	2.7	6.2	-0.17	2.4	2.4	0
85-89	2	2.7	-0.05	1.4	1.6	-0.02
90-94	1.1	1	0.01	0.7	0.7	-0.01
95-99	0.3	0.2	0.03	0.2	0.1	0.01
**Sex**						
Female	53.8	56.9	-0.06	53.9	53.2	0.01

Table S6.67. *Continued.* Selected baseline characteristics for Germany IQVIA, for the long-term risk of self-harm and suicide

Characteristic	Before propensity score matching			After propensity score matching		
	**Targets, %**	**Comparators, %**	**Standardized difference**	**Targets, %**	**Comparators, %**	**Standardized difference**
**Medical history (general)**						
Acute respiratory disease	59	7.9	1.29	60.6	63.4	-0.06
Chronic liver disease	0.2	0.1	0.02	0.1	0.2	-0.01
Chronic obstructive lung disease	2.8	1.2	0.11	3	4.2	-0.06
Crohn’s disease	0.2	0.1	0.02	0.2	0.3	-0.02
Dementia	1.2	0.5	0.08	0.9	1.2	-0.03
Depressive disorder	7.2	2.8	0.2	7.6	7.4	0.01
Diabetes mellitus	4	2.2	0.1	4.1	5	-0.04
Gastroesophageal reflux disease	1.4	0.5	0.1	1.5	1.6	-0.01
Gastrointestinal hemorrhage	0.6	0.2	0.06	0.6	0.6	0
Hyperlipidemia	5.5	2.4	0.16	5.8	6.9	-0.04
Hypertensive disorder	11.7	5.9	0.21	12.3	14.5	-0.06
Lesion of liver	0.2	0.1	0.01	0.2	0.2	-0.02
Obesity	2.6	1.1	0.11	2.7	2.7	0
Osteoarthritis	5.4	3.3	0.1	5.7	6.5	-0.03
Pneumonia	3	0.4	0.2	2.9	3	-0.01
Psoriasis	0.6	0.5	0.01	0.7	0.8	-0.02
Renal impairment	1.6	0.7	0.08	1.4	1.8	-0.04
Rheumatoid arthritis	0.6	0.4	0.03	0.7	0.8	-0.02
Schizophrenia	0.1	0.1	0.01	0.1	0.2	-0.01
Ulcerative colitis	0.2	0.1	0.02	0.2	0.3	-0.02
Urinary tract infectious disease	4.1	1.4	0.16	4.2	5.3	-0.05

Table S6.67. *Continued.* Selected baseline characteristics for Germany IQVIA, for the long-term risk of self-harm and suicide

**Characteristic**	**Before propensity score matching**			**After propensity score matching**		
	**Targets, %**	**Comparators, %**	**Standardized difference**	**Targets, %**	**Comparators, %**	**Standardized difference**
**Medical history (cardiovascular disease)**						
Atrial fibrillation	0.8	0.5	0.03	0.7	0.9	-0.01
Cerebrovascular disease	1.2	0.7	0.04	1.1	1.4	-0.03
Coronary arteriosclerosis	1	0.6	0.04	1	1.4	-0.04
Heart disease	7.7	3.9	0.16	7.8	9	-0.04
Heart failure	1.8	0.9	0.08	1.6	2	-0.03
Ischemic heart disease	2.4	1.2	0.09	2.4	2.8	-0.03
Peripheral vascular disease	0.9	0.6	0.03	0.8	1.1	-0.03
Pulmonary embolism	0.3	0.1	0.04	0.3	0.4	-0.03
Venous thrombosis	0.7	0.3	0.06	0.8	1	-0.03
**Medical history (neoplasms)**						
Malignant lymphoma	0.1	0.1	0.02	0.2	0.2	-0.01
Malignant neoplastic disease	1.9	2.1	-0.01	1.9	2.4	-0.04
Malignant tumor of breast	0.3	0.4	-0.02	0.3	0.3	-0.01
Malignant tumor of urinary bladder	0.1	0.1	0	0.1	0.1	0
Primary malignant neoplasm of prostate	0.2	0.3	-0.03	0.2	0.2	0
**Medication use**						
Agents acting on the renin-angiotensin system	18.9	8.6	0.3	20.3	21.8	-0.04
Antibacterials for systemic use	26.6	9.2	0.46	28.3	33.8	-0.12
Antidepressants	6.2	3.6	0.12	6.4	7.9	-0.06
Antiepileptics	2.3	1.4	0.06	2.2	2.8	-0.04
Antiinflammatory and antirheumatic products	32.6	11.7	0.52	34.9	35.9	-0.02
Antineoplastic agents	1	0.9	0.01	1.1	1.3	-0.02
Antipsoriatics	0.2	0.3	0	0.2	0.3	-0.01
Antithrombotic agents	10	4.6	0.21	9.8	11.5	-0.06
Beta blocking agents	12.1	5.9	0.22	12.6	14.3	-0.05

Table S6.67. *Continued.* Selected baseline characteristics for Germany IQVIA, for the long-term risk of self-harm and suicide

Characteristic	Before propensity score matching			After propensity score matching		
	Targets, %	Comparators, %	Standardized difference	Targets, %	Comparators, %	Standardized difference
**Medication use**						
Calcium channel blockers	8.4	3.9	0.19	9	9.7	-0.02
Diuretics	11	5.1	0.22	11	12.7	-0.05
Drugs for acid-related disorders	20.1	6.8	0.4	21.2	23.9	-0.06
Drugs for obstructive airway diseases	15.1	7.2	0.26	16.4	20.4	-0.1
Drugs used in diabetes	5.8	3	0.14	6	6.5	-0.02
Immunosuppressants	0.6	0.7	-0.02	0.6	0.8	-0.02
Lipid modifying agents	9.4	4.7	0.18	9.9	11.6	-0.05
Opioids	8.8	2.6	0.27	9	10.3	-0.04
Psycholeptics	5.1	2.8	0.12	4.8	6.1	-0.06
Psychostimulants, agents used for ADHD and nootropics	0.3	0.2	0	0.3	0.4	-0.01

Table S6.68. Selected baseline characteristics for Germany IQVIA, for the long-term risk of sleep disorders

Characteristic	Before propensity score matching			After propensity score matching		
	Targets, %	Comparators, %	Standardized difference	Targets, %	Comparators, %	Standardized difference
**Age group (years)**						
0-4	1.4	2.4	-0.07	1.6	3.1	-0.1
5-9	2.2	3.2	-0.06	2.4	3.5	-0.07
10-14	2.8	3.3	-0.03	2.9	3.6	-0.04
15-19	5	3.5	0.07	5	5.6	-0.03
20-24	7.6	4	0.16	7.8	7.8	0
25-29	7.4	4.2	0.13	7.3	6.9	0.01
30-34	8	5.2	0.11	7.6	7.3	0.01
35-39	7.6	5.3	0.1	7.7	7.4	0.01
40-44	8.3	5.5	0.11	8.6	8.1	0.02
45-49	8.1	6	0.08	8.4	7.8	0.02
50-54	9.9	8.4	0.05	10.3	9.5	0.02
55-59	9.6	9.3	0.01	9.8	9.5	0.01
60-64	7	8.7	-0.06	7.3	6.8	0.02
65-69	3.6	7.7	-0.18	3.7	3.7	0
70-74	2.9	6.8	-0.18	2.9	2.6	0.02
75-79	2.5	6.3	-0.19	2.2	2.3	0
85-89	2	2.7	-0.05	1.4	1.5	-0.01
90-94	1.1	1	0.01	0.6	0.7	0
95-99	0.3	0.2	0.03	0.2	0.1	0
**Sex**						
Female	53.8	57.3	-0.07	53.8	53.3	0.01

Table S6.68. *Continued.* Selected baseline characteristics for Germany IQVIA, for the long-term risk of sleep disorders

Characteristic	Before propensity score matching			After propensity score matching		
	Targets, %	Comparators, %	Standardized difference	Targets, %	Comparators, %	Standardized difference
**Medical history (general)**						
Acute respiratory disease	59	7.9	1.29	60.4	63.4	-0.06
Chronic liver disease	0.2	0.1	0.03	0.1	0.2	-0.01
Chronic obstructive lung disease	2.8	1.3	0.11	2.8	3.8	-0.05
Crohn’s disease	0.2	0.1	0.02	0.2	0.3	-0.02
Dementia	1.2	0.5	0.07	0.7	1.1	-0.04
Depressive disorder	7.2	2.7	0.21	6.6	6.3	0.01
Diabetes mellitus	4	2.2	0.11	3.9	4.5	-0.03
Gastroesophageal reflux disease	1.4	0.5	0.09	1.3	1.4	-0.01
Gastrointestinal hemorrhage	0.6	0.2	0.06	0.5	0.5	0
Hyperlipidemia	5.5	2.4	0.16	5.2	6.2	-0.04
Hypertensive disorder	11.7	5.9	0.2	11.4	13.2	-0.06
Lesion of liver	0.2	0.1	0.01	0.1	0.2	-0.02
Obesity	2.6	1.1	0.11	2.4	2.3	0
Osteoarthritis	5.4	3.4	0.1	5.1	6	-0.04
Pneumonia	3	0.4	0.2	2.7	2.9	-0.01
Psoriasis	0.6	0.5	0.01	0.6	0.8	-0.01
Renal impairment	1.6	0.7	0.08	1.2	1.6	-0.04
Rheumatoid arthritis	0.6	0.4	0.03	0.6	0.8	-0.02
Schizophrenia	0.1	0.1	0.01	0.1	0.2	-0.01
Ulcerative colitis	0.2	0.1	0.01	0.1	0.3	-0.03
Urinary tract infectious disease	4.1	1.4	0.16	4	5.1	-0.05

Table S6.68. *Continued.* Selected baseline characteristics for Germany IQVIA, for the long-term risk of sleep disorders

Characteristic	Before propensity score matching			After propensity score matching		
	Targets, %	Comparators, %	Standardized difference	Targets, %	Comparators, %	Standardized difference
**Medical history (cardiovascular disease)**						
Atrial fibrillation	0.8	0.5	0.03	0.7	0.7	-0.01
Cerebrovascular disease	1.2	0.7	0.05	1	1.2	-0.02
Coronary arteriosclerosis	1	0.6	0.04	0.9	1.3	-0.04
Heart disease	7.7	4	0.16	7.1	8.1	-0.04
Heart failure	1.8	0.9	0.08	1.3	1.7	-0.03
Ischemic heart disease	2.4	1.3	0.08	2.1	2.5	-0.03
Peripheral vascular disease	0.9	0.5	0.04	0.8	1	-0.03
Pulmonary embolism	0.3	0.1	0.04	0.3	0.4	-0.03
Venous thrombosis	0.7	0.3	0.06	0.7	1	-0.03
**Medical history (neoplasms)**						
Malignant lymphoma	0.1	0.1	0.02	0.2	0.2	-0.01
Malignant neoplastic disease	1.9	2.1	-0.01	1.7	2.2	-0.03
Malignant tumor of breast	0.3	0.4	-0.02	0.2	0.3	-0.02
Primary malignant neoplasm of prostate	0.2	0.3	-0.03	0.2	0.2	0
**Medication use**						
Agents acting on the renin-angiotensin system	18.9	8.6	0.3	19.3	20.6	-0.03
Antibacterials for systemic use	26.6	9.2	0.47	27.6	33.3	-0.12
Antidepressants	6.2	3.5	0.13	5.5	6.8	-0.05
Antiepileptics	2.3	1.5	0.06	2	2.6	-0.04
Antiinflammatory and antirheumatic products	32.6	11.7	0.52	34.1	35.4	-0.03
Antineoplastic agents	1	0.9	0.01	1	1.2	-0.02
Antithrombotic agents	10	4.6	0.21	9.2	10.8	-0.05
Beta blocking agents	12.1	6	0.21	12	13.5	-0.05
Calcium channel blockers	8.4	3.9	0.19	8.4	9.1	-0.02
Diuretics	11	5.2	0.21	10.4	11.7	-0.04

Table S6.68. *Continued.* Selected baseline characteristics for Germany IQVIA, for the long-term risk of sleep disorders

Characteristic	Before propensity score matching			After propensity score matching		
	**Targets, %**	**Comparators, %**	**Standardized difference**	**Targets, %**	**Comparators, %**	**Standardized difference**
**Medication use**						
Drugs for acid-related disorders	20.1	6.8	0.4	20.2	22.8	-0.06
Drugs for obstructive airway diseases	15.1	7.3	0.25	15.9	19.8	-0.1
Drugs used in diabetes	5.8	3	0.14	5.7	6.1	-0.02
Immunosuppressants	0.6	0.7	-0.02	0.6	0.8	-0.02
Lipid modifying agents	9.4	4.6	0.19	9.2	10.9	-0.05
Opioids	8.8	2.6	0.27	8.6	9.6	-0.03
Psycholeptics	5.1	2.8	0.12	3.6	4.4	-0.04
Psychostimulants, agents used for ADHD and nootropics	0.3	0.2	0	0.3	0.3	-0.01

Table S6.69. Selected baseline characteristics for Germany IQVIA, for the long-term risk of dementia

Characteristic	Before propensity score matching			After propensity score matching		
	Targets, %	Comparators, %	Standardized difference	Targets, %	Comparators, %	Standardized difference
**Age group (years)**						
0-4	1.4	2.3	-0.07	1.6	3	-0.1
5-9	2.2	3.3	-0.06	2.3	3.4	-0.06
10-14	2.8	3.2	-0.02	2.7	3.4	-0.04
15-19	5	3.6	0.07	4.9	5.5	-0.03
20-24	7.6	3.9	0.16	7.6	7.7	0
25-29	7.4	4.3	0.13	7.2	6.9	0.01
30-34	8	5.2	0.11	7.6	7.2	0.01
35-39	7.6	5.4	0.09	7.7	7.4	0.01
40-44	8.3	5.5	0.11	8.6	8.2	0.01
45-49	8.1	6	0.08	8.5	8	0.02
50-54	9.9	8.2	0.06	10.5	9.8	0.02
55-59	9.6	9.4	0.01	10.3	9.9	0.01
60-64	7	8.7	-0.06	7.6	7.1	0.02
65-69	3.6	7.8	-0.18	3.8	3.8	0
70-74	2.9	6.8	-0.18	2.9	2.6	0.02
75-79	2.5	6.3	-0.19	2.3	2.2	0.01
80-84	2.7	6.2	-0.17	2.1	2	0.01
85-89	2	2.7	-0.05	1.2	1.3	0
90-94	1.1	1	0.01	0.5	0.5	0
95-99	0.3	0.2	0.03	0.2	0.1	0.01
**Sex**						
Female	53.8	57.1	-0.07	53.7	53	0.01

Table S6.69. *Continued.* Selected baseline characteristics for Germany IQVIA, for the long-term risk dementia

Characteristic	Before propensity score matching			After propensity score matching		
	**Targets, %**	**Comparators, %**	**Standardized difference**	**Targets, %**	**Comparators, %**	**Standardized difference**
**Medical history (general)**						
Acute respiratory disease	59	7.9	1.29	60.9	63.8	-0.06
Chronic liver disease	0.2	0.1	0.02	0.1	0.2	-0.02
Chronic obstructive lung disease	2.8	1.3	0.11	3	4.1	-0.06
Crohn’s disease	0.2	0.1	0.02	0.2	0.3	-0.02
Depressive disorder	7.2	2.7	0.21	7.4	7.1	0.01
Diabetes mellitus	4	2.2	0.11	3.9	4.8	-0.04
Gastroesophageal reflux disease	1.4	0.5	0.1	1.5	1.6	-0.01
Gastrointestinal hemorrhage	0.6	0.2	0.06	0.6	0.6	0
Hyperlipidemia	5.5	2.4	0.16	5.6	6.6	-0.04
Hypertensive disorder	11.7	5.8	0.21	11.9	14	-0.06
Lesion of liver	0.2	0.1	0.01	0.1	0.2	-0.02
Obesity	2.6	1.1	0.11	2.7	2.6	0
Osteoarthritis	5.4	3.4	0.1	5.6	6.3	-0.03
Pneumonia	3	0.4	0.2	2.9	2.9	0
Psoriasis	0.6	0.5	0.02	0.7	0.8	-0.02
Renal impairment	1.6	0.7	0.08	1.3	1.6	-0.03
Rheumatoid arthritis	0.6	0.5	0.02	0.7	0.8	-0.02
Schizophrenia	0.1	0.1	0.01	0.1	0.1	-0.01
Ulcerative colitis	0.2	0.1	0.02	0.2	0.3	-0.02
Urinary tract infectious disease	4.1	1.4	0.16	4.1	5.1	-0.05

Table S6.69. *Continued.* Selected baseline characteristics for Germany IQVIA, for the long-term risk dementia

Characteristic	Before propensity score matching			After propensity score matching		
	Targets, %	Comparators, %	Standardized difference	Targets, %	Comparators, %	Standardized difference
**Medical history (cardiovascular disease)**						
Atrial fibrillation	0.8	0.5	0.03	0.7	0.8	-0.01
Cerebrovascular disease	1.2	0.7	0.04	1	1.2	-0.02
Coronary arteriosclerosis	1	0.6	0.04	1	1.3	-0.03
Heart disease	7.7	3.9	0.16	7.4	8.4	-0.04
Heart failure	1.8	0.8	0.09	1.5	1.8	-0.02
Ischemic heart disease	2.4	1.3	0.09	2.3	2.6	-0.02
Peripheral vascular disease	0.9	0.6	0.04	0.8	1	-0.03
Pulmonary embolism	0.3	0.1	0.04	0.3	0.4	-0.03
Venous thrombosis	0.7	0.3	0.06	0.7	1	-0.03
**Medical history (neoplasms)**						
Malignant lymphoma	0.1	0.1	0.02	0.1	0.2	-0.01
Malignant neoplastic disease	1.9	2.1	-0.01	1.8	2.3	-0.04
Malignant tumor of breast	0.3	0.4	-0.02	0.2	0.3	-0.01
Malignant tumor of colon	0.1	0.1	0	0.1	0.1	-0.01
Malignant tumor of urinary bladder	0.1	0.1	-0.01	0.1	0.1	0
Primary malignant neoplasm of prostate	0.2	0.3	-0.03	0.2	0.2	0
**Medication use**						
Agents acting on the renin-angiotensin system	18.9	8.6	0.3	20	21.2	-0.03
Antibacterials for systemic use	26.6	9.2	0.47	28.2	33.7	-0.12
Antidepressants	6.2	3.5	0.13	6.1	7.6	-0.06
Antiepileptics	2.3	1.5	0.06	2.1	2.7	-0.04
Antiinflammatory and antirheumatic products	32.6	11.7	0.52	34.7	35.7	-0.02
Antineoplastic agents	1	0.9	0.01	1.1	1.2	-0.01
Antipsoriatics	0.2	0.2	0	0.2	0.3	-0.01
Antithrombotic agents	10	4.6	0.2	9.3	10.8	-0.05

Table S6.69. *Continued.* Selected baseline characteristics for Germany IQVIA, for the long-term risk of dementia

Characteristic	Before propensity score matching			After propensity score matching		
	**Targets, %**	**Comparators, %**	**Standardized difference**	**Targets, %**	**Comparators, %**	**Standardized difference**
**Medication use**						
Beta blocking agents	12.1	5.9	0.22	12.2	13.7	-0.04
Calcium channel blockers	8.4	3.8	0.19	8.8	9.4	-0.02
Diuretics	11	5.2	0.22	10.5	11.9	-0.04
Drugs for acid-related disorders	20.1	6.8	0.4	20.9	23.4	-0.06
Drugs for obstructive airway diseases	15.1	7.2	0.25	16.4	20.3	-0.1
Drugs used in diabetes	5.8	3	0.14	5.8	6.2	-0.02
Immunosuppressants	0.6	0.7	-0.02	0.6	0.8	-0.02
Lipid modifying agents	9.4	4.6	0.19	9.7	11.1	-0.05
Opioids	8.8	2.6	0.27	8.9	10	-0.04
Psycholeptics	5.1	2.8	0.12	4.5	5.5	-0.04
Psychostimulants, agents used for ADHD and nootropics	0.3	0.2	0.01	0.3	0.4	-0.02

Table S6.70. Selected baseline characteristics for Germany IQVIA, for the long-term risk of neurodevelopmental disorders

Characteristic	Before propensity score matching			After propensity score matching		
	Targets, %	Comparators, %	Standardized difference	Targets, %	Comparators, %	Standardized difference
**Age group (years)**						
0-4	1.4	2.3	-0.07	1.3	2.5	-0.09
5-9	2.2	3.3	-0.06	1.9	2.8	-0.06
10-14	2.8	3.3	-0.03	2.5	3	-0.03
15-19	5	3.5	0.07	4.8	5.3	-0.02
20-24	7.6	4	0.16	7.5	7.7	-0.01
25-29	7.4	4.2	0.13	7.2	6.9	0.01
30-34	8	5.1	0.11	7.6	7.3	0.01
35-39	7.6	5.3	0.09	7.7	7.4	0.01
40-44	8.3	5.5	0.11	8.6	8.2	0.02
45-49	8.1	6	0.08	8.5	8	0.02
50-54	9.9	8.3	0.06	10.5	9.9	0.02
55-59	9.6	9.4	0.01	10.3	9.9	0.01
60-64	7	8.6	-0.06	7.6	7.2	0.02
65-69	3.6	7.8	-0.18	3.8	3.8	0
70-74	2.9	6.8	-0.18	3	2.7	0.02
75-79	2.5	6.4	-0.19	2.4	2.4	0
85-89	2	2.7	-0.05	1.4	1.6	-0.02
90-94	1.1	1	0.01	0.7	0.7	0
95-99	0.3	0.2	0.03	0.2	0.1	0.01
**Sex**						
Female	53.8	57.1	-0.07	54.1	53.5	0.01

Table S6.70. *Continued.* Selected baseline characteristics for Germany IQVIA, for the long-term risk of neurodevelopmental disorders

Characteristic	Before propensity score matching			After propensity score matching		
	Targets, %	Comparators, %	Standardized difference	Targets, %	Comparators, %	Standardized difference
**Medical history (general)**						
Medical history: General						
Acute respiratory disease	59	7.9	1.29	60.5	63.2	-0.06
Chronic liver disease	0.2	0.1	0.03	0.1	0.2	-0.01
Chronic obstructive lung disease	2.8	1.2	0.11	3	4.1	-0.06
Crohn’s disease	0.2	0.1	0.02	0.2	0.3	-0.02
Dementia	1.2	0.5	0.07	0.8	1.1	-0.03
Depressive disorder	7.2	2.7	0.21	7.5	7.3	0.01
Diabetes mellitus	4	2.3	0.1	4.2	5.1	-0.04
Gastroesophageal reflux disease	1.4	0.5	0.1	1.5	1.6	-0.01
Gastrointestinal hemorrhage	0.6	0.2	0.06	0.6	0.6	0
Hyperlipidemia	5.5	2.4	0.16	5.8	6.9	-0.04
Hypertensive disorder	11.7	5.9	0.2	12.4	14.7	-0.07
Lesion of liver	0.2	0.1	0.01	0.2	0.2	-0.02
Obesity	2.6	1.1	0.11	2.7	2.6	0.01
Osteoarthritis	5.4	3.4	0.09	5.7	6.6	-0.04
Pneumonia	3	0.4	0.2	2.9	3	-0.01
Psoriasis	0.6	0.5	0.02	0.7	0.8	-0.02
Renal impairment	1.6	0.7	0.08	1.3	1.9	-0.04
Rheumatoid arthritis	0.6	0.4	0.03	0.7	0.9	-0.02
Schizophrenia	0.1	0.1	0	0.1	0.2	-0.01
Ulcerative colitis	0.2	0.1	0.02	0.2	0.2	-0.02
Urinary tract infectious disease	4.1	1.5	0.16	4.3	5.2	-0.04

Table S6.70. *Continued.* Selected baseline characteristics for Germany IQVIA, for the long-term risk of neurodevelopmental disorders

**Characteristic**	**Before propensity score matching**			**After propensity score matching**		
	**Targets, %**	**Comparators, %**	**Standardized difference**	**Targets, %**	**Comparators, %**	**Standardized difference**
**Medical history (cardiovascular disease)**						
Atrial fibrillation	0.8	0.5	0.03	0.7	0.9	-0.01
Cerebrovascular disease	1.2	0.7	0.05	1.1	1.4	-0.03
Coronary arteriosclerosis	1	0.6	0.04	1	1.5	-0.04
Heart disease	7.7	4	0.16	7.7	9	-0.05
Heart failure	1.8	0.9	0.08	1.6	2	-0.04
Ischemic heart disease	2.4	1.3	0.08	2.4	2.9	-0.03
Peripheral vascular disease	0.9	0.6	0.04	0.8	1.1	-0.03
Pulmonary embolism	0.3	0.1	0.04	0.3	0.5	-0.03
Venous thrombosis	0.7	0.3	0.06	0.8	1	-0.03
**Medical history (neoplasms)**						
Malignant lymphoma	0.1	0.1	0.02	0.2	0.2	-0.01
Malignant neoplastic disease	1.9	2.1	-0.01	1.8	2.4	-0.04
Malignant tumor of breast	0.3	0.4	-0.01	0.3	0.3	-0.01
Malignant tumor of colon	0.1	0.1	0	0.1	0.1	-0.02
Malignant tumor of urinary bladder	0.1	0.1	-0.01	0.1	0.1	0
Primary malignant neoplasm of prostate	0.2	0.3	-0.03	0.2	0.2	0
**Medication use**						
Agents acting on the renin-angiotensin system	18.9	8.6	0.3	20.5	22.1	-0.04
Antibacterials for systemic use	26.6	9.2	0.47	28.3	33.7	-0.12
Antidepressants	6.2	3.5	0.12	6.4	7.9	-0.06
Antiepileptics	2.3	1.5	0.06	2.1	2.7	-0.04
Antiinflammatory and antirheumatic products	32.6	11.6	0.52	34.5	35.5	-0.02
Antineoplastic agents	1	0.9	0.01	1.1	1.2	-0.02
Antipsoriatics	0.2	0.2	0	0.2	0.3	-0.01
Antithrombotic agents	10	4.6	0.21	9.8	11.6	-0.06

Table S6.70. *Continued.* Selected baseline characteristics for Germany IQVIA, for the long-term risk of neurodevelopmental disorders

Characteristic	Before propensity score matching			After propensity score matching		
	**Targets, %**	**Comparators, %**	**Standardized difference**	**Targets, %**	**Comparators, %**	**Standardized difference**
**Medication use**						
Beta blocking agents	12.1	6	0.21	12.7	14.5	-0.05
Calcium channel blockers	8.4	3.8	0.19	9.1	9.8	-0.03
Diuretics	11	5.2	0.21	11	12.8	-0.06
Drugs for acid-related disorders	20.1	6.7	0.4	21.3	24.2	-0.07
Drugs for obstructive airway diseases	15.1	7.1	0.26	16.3	20.2	-0.1
Drugs used in diabetes	5.8	3	0.14	6.1	6.6	-0.02
Immunosuppressants	0.6	0.7	-0.02	0.6	0.8	-0.02
Lipid modifying agents	9.4	4.7	0.18	9.9	11.7	-0.06
Opioids	8.8	2.6	0.27	9.1	10.4	-0.04
Psycholeptics	5.1	2.8	0.12	4.7	6	-0.05
Psychostimulants, agents used for ADHD and nootropics	0.3	0.2	0.01	0.2	0.2	0

Table S6.71. Selected baseline characteristics for Germany IQVIA, for the long-term risk of any of psychiatric disorders and neuropsychiatric disorders

Characteristic	Before propensity score matching			After propensity score matching		
	Targets, %	Comparators, %	Standardized difference	Targets, %	Comparators, %	Standardized difference
**Age group (years)**						
0-4	1.4	2.3	-0.07	1.6	3.2	-0.1
5-9	2.2	3.3	-0.06	2.5	3.6	-0.07
10-14	2.8	3.3	-0.03	3	3.8	-0.04
15-19	5	3.5	0.07	5.4	6	-0.03
20-24	7.6	4	0.16	8	8.2	-0.01
25-29	7.4	4.3	0.13	7.5	7.1	0.01
30-34	8	5.1	0.12	7.7	7.5	0.01
35-39	7.6	5.3	0.09	7.7	7.3	0.01
40-44	8.3	5.5	0.11	8.3	8	0.01
45-49	8.1	6	0.08	8.5	7.6	0.03
50-54	9.9	8.3	0.06	10.3	9.3	0.03
55-59	9.6	9.3	0.01	9.7	9.1	0.02
60-64	7	8.7	-0.06	7.2	6.6	0.02
65-69	3.6	7.8	-0.18	3.7	3.8	-0.01
70-74	2.9	6.9	-0.19	3	2.7	0.02
75-79	2.5	6.4	-0.19	2.1	2.2	0
85-89	2	2.7	-0.05	1.2	1.3	-0.01
90-94	1.1	1	0.01	0.5	0.5	0
95-99	0.3	0.2	0.03	0.1	0.1	0.01
**Sex**						
Female	53.8	57	-0.06	52	51.7	0.01

Table S6.71. *Continued.* Selected baseline characteristics for Germany IQVIA, for the long-term risk of psychiatric disorders and neuropsychiatric disorders

Characteristic	Before propensity score matching			After propensity score matching		
	Targets, %	Comparators, %	Standardized difference	Targets, %	Comparators, %	Standardized difference
**Medical history (general)**						
Acute respiratory disease	59	7.8	1.29	59.5	62.5	-0.06
Chronic obstructive lung disease	2.8	1.3	0.11	2.2	3.1	-0.05
Crohn’s disease	0.2	0.1	0.02	0.1	0.3	-0.03
Diabetes mellitus	4	2.3	0.1	3.2	3.9	-0.04
Gastroesophageal reflux disease	1.4	0.5	0.09	1.1	1.2	-0.01
Gastrointestinal hemorrhage	0.6	0.2	0.06	0.5	0.5	0
Hyperlipidemia	5.5	2.4	0.16	4.3	5.3	-0.05
Hypertensive disorder	11.7	5.9	0.2	9.7	11.3	-0.05
Lesion of liver	0.2	0.1	0.01	0.1	0.2	-0.03
Obesity	2.6	1.1	0.11	1.8	1.8	0
Osteoarthritis	5.4	3.4	0.1	4.2	5.1	-0.04
Pneumonia	3	0.4	0.2	2.6	2.6	0
Psoriasis	0.6	0.5	0.01	0.5	0.7	-0.02
Renal impairment	1.6	0.8	0.08	0.9	1.3	-0.04
Rheumatoid arthritis	0.6	0.4	0.02	0.5	0.7	-0.02
Ulcerative colitis	0.2	0.1	0.01	0.1	0.2	-0.03
Urinary tract infectious disease	4.1	1.5	0.16	3.6	4.5	-0.05

Table S6.71. *Continued.* Selected baseline characteristics for Germany IQVIA, for the long-term risk of psychiatric disorders and neuropsychiatric disorders

**Characteristic**	**Before propensity score matching**			**After propensity score matching**		
	**Targets, %**	**Comparators, %**	**Standardized difference**	**Targets, %**	**Comparators, %**	**Standardized difference**
**Medical history (cardiovascular disease)**						
Atrial fibrillation	0.8	0.5	0.03	0.5	0.6	-0.01
Cerebrovascular disease	1.2	0.7	0.05	0.9	0.9	-0.01
Coronary arteriosclerosis	1	0.7	0.04	0.8	1.1	-0.04
Heart disease	7.7	4	0.16	5.7	6.7	-0.04
Heart failure	1.8	0.9	0.08	1.1	1.3	-0.02
Ischemic heart disease	2.4	1.3	0.09	1.7	2	-0.03
Peripheral vascular disease	0.9	0.6	0.04	0.6	0.8	-0.02
Pulmonary embolism	0.3	0.1	0.04	0.3	0.4	-0.02
Venous thrombosis	0.7	0.3	0.06	0.6	0.9	-0.03
**Medical history (neoplasms)**						
Malignant lymphoma	0.1	0.1	0.02	0.1	0.2	0
Malignant neoplastic disease	1.9	2.1	-0.01	1.5	1.9	-0.03
Malignant tumor of breast	0.3	0.4	-0.02	0.2	0.3	-0.03
Primary malignant neoplasm of prostate	0.2	0.3	-0.03	0.1	0.1	-0.01
**Medication use**						
Agents acting on the renin-angiotensin system	18.9	8.6	0.3	18.3	19.4	-0.03
Antibacterials for systemic use	26.6	9.2	0.47	26.2	31.8	-0.12
Antidepressants	6.2	3.6	0.12	2.8	3.1	-0.02
Antiepileptics	2.3	1.5	0.06	1.6	2	-0.03
Antiinflammatory and antirheumatic products	32.6	11.7	0.52	31.9	34.1	-0.05
Antineoplastic agents	1	0.9	0.01	0.9	1.1	-0.02
Antipsoriatics	0.2	0.3	0	0.2	0.3	-0.01
Antithrombotic agents	10	4.7	0.2	8.4	9.7	-0.05

Table S6.71. *Continued.* Selected baseline characteristics for Germany IQVIA, for the long-term risk of psychiatric disorders and neuropsychiatric disorders

Characteristic	Before propensity score matching			After propensity score matching		
	Targets, %	Comparators, %	Standardized difference	Targets, %	Comparators, %	Standardized difference
**Medication use**						
Beta blocking agents	12.1	5.9	0.22	10.8	12.1	-0.04
Calcium channel blockers	8.4	3.9	0.19	8	8.4	-0.02
Diuretics	11	5.2	0.21	9.6	10.7	-0.04
Drugs for acid-related disorders	20.1	6.8	0.4	17.9	20.4	-0.06
Drugs for obstructive airway diseases	15.1	7.3	0.25	14.7	18.8	-0.11
Drugs used in diabetes	5.8	3	0.14	5.2	5.6	-0.02
Immunosuppressants	0.6	0.7	-0.02	0.6	0.8	-0.03
Lipid modifying agents	9.4	4.7	0.18	8.6	10.1	-0.05
Opioids	8.8	2.7	0.27	7.8	8.6	-0.03
Psycholeptics	5.1	2.8	0.12	2.2	2.6	-0.02
Psychostimulants, agents used for ADHD and nootropics	0.3	0.2	0	0.1	0.2	-0.01

Table S6.72. Selected baseline characteristics for Italy IQVIA, for the short-term risk of depression

Characteristic	Before propensity score matching			After propensity score matching		
	Targets, %	Comparators, %	Standardized difference	Targets, %	Comparators, %	Standardized difference
**Age group (years)**						
10-14	0.5	0.5	-0.01	0.4	0.8	-0.04
15-19	2.8	3.9	-0.06	3.2	3.5	-0.02
20-24	4.8	4.7	0.01	4.8	5.2	-0.02
25-29	5.6	4.9	0.03	5.2	5.6	-0.02
30-34	5.9	5.1	0.04	6.1	6.1	0
35-39	6.5	5.6	0.04	6.1	6.7	-0.02
40-44	8	6.6	0.05	8.1	8.5	-0.01
45-49	10.4	8.2	0.07	10.4	11	-0.02
55-59	11.7	9.5	0.07	11.7	9.9	0.06
60-64	8.6	8.8	-0.01	8.9	8.3	0.02
65-69	6.2	8.1	-0.07	6.4	6.4	0
70-74	5.8	8.2	-0.1	5.7	5.4	0.01
75-79	4	6.1	-0.1	4.3	3.9	0.02
80-84	4	5.5	-0.07	4.1	3.9	0.01
85-89	2.7	3.4	-0.04	2.2	2.3	-0.01
90-94	1.4	1.5	-0.01	1.1	1.2	-0.01
95-99	0.4	0.2	0.03	0.3	0.2	0.02
**Sex**						
Female	51.9	54.5	-0.05	50.9	51.4	-0.01

Table S6.72. *Continued.* Selected baseline characteristics for Italy IQVIA, for the short-term risk of depression

Characteristic	Before propensity score matching			After propensity score matching		
	Targets, %	Comparators, %	Standardized difference	Targets, %	Comparators, %	Standardized difference
**Medical history (general)**						
Acute respiratory disease	24	13.2	0.28	25.1	30.4	-0.12
Chronic liver disease	1.9	1.8	0.01	1.8	2.1	-0.02
Chronic obstructive lung disease	1.8	1.7	0.01	1.8	1.6	0.02
Crohn's disease	0.3	0.2	0.01	0.3	0.3	-0.01
Dementia	0.8	0.7	0.02	0.6	0.6	0
Diabetes mellitus	7.7	8.5	-0.03	7.4	7.6	-0.01
Gastroesophageal reflux disease	9	7.6	0.05	8.4	8.9	-0.01
Gastrointestinal hemorrhage	0.3	0.3	0	0.3	0.3	-0.01
Human immunodeficiency virus infection	0.1	0.1	0.02	0.1	0.1	0.01
Hyperlipidemia	10.8	11.4	-0.02	10.7	10.3	0.01
Hypertensive disorder	25.4	27.3	-0.04	24.7	23.8	0.02
Lesion of liver	0.3	0.3	-0.01	0.3	0.4	-0.02
Obesity	0.5	0.3	0.03	0.3	0.4	-0.01
Osteoarthritis	7.7	8.2	-0.02	7.4	7.8	-0.01
Pneumonia	9.7	0.9	0.4	4.4	3.6	0.04
Psoriasis	1.7	1.4	0.02	1.5	1.5	0
Renal impairment	2.1	2	0.01	2.2	2.2	0
Rheumatoid arthritis	0.6	0.7	-0.01	0.5	0.5	0.01
Schizophrenia	0.1	0.2	-0.02	0.2	0.2	-0.01
Ulcerative colitis	0.4	0.3	0.02	0.4	0.4	0.01
Urinary tract infectious disease	2.8	2.1	0.04	2.5	3	-0.03
Viral hepatitis C	0.4	0.4	0	0.4	0.5	-0.01

Table S6.72. *Continued.* Selected baseline characteristics for Italy IQVIA, for the short-term risk of depression

Characteristic	Before propensity score matching			After propensity score matching		
	Targets, %	Comparators, %	Standardized difference	Targets, %	Comparators, %	Standardized difference
**Medical history (cardiovascular disease)**						
Atrial fibrillation	3	3	0	2.9	2.8	0.01
Cerebrovascular disease	3.8	3.9	-0.01	3.3	3.2	0
Coronary arteriosclerosis	0.4	0.3	0.02	0.4	0.4	0.01
Heart disease	12.3	12.2	0	11.8	12.1	-0.01
Heart failure	1.2	1.2	0	1.2	1.2	0
Ischemic heart disease	3.8	3.8	0	3.5	3.7	-0.01
Peripheral vascular disease	0.5	0.6	-0.01	0.4	0.5	0
Pulmonary embolism	0.3	0.2	0.02	0.2	0.2	0
Venous thrombosis	0.5	0.4	0.01	0.5	0.6	-0.01
**Medical history (neoplasms)**						
Malignant lymphoma	0.4	0.3	0.01	0.3	0.4	-0.02
Malignant neoplastic disease	6.2	6.1	0	5.9	5.7	0.01
Malignant tumor of breast	1.4	1.5	-0.01	1.3	1.1	0.02
Malignant tumor of colon	0.4	0.5	-0.01	0.4	0.3	0.01
Malignant tumor of urinary bladder	0.4	0.4	0	0.4	0.3	0
Primary malignant neoplasm of prostate	0.8	0.8	0	0.9	0.6	0.03
**Medication use**						
Agents acting on the renin-angiotensin system	20.6	23.7	-0.07	20.4	19.7	0.02
Antibacterials for systemic use	48.3	32.1	0.34	41	44.1	-0.06
Antidepressants	10.7	9.9	0.03	6	6.1	0
Antiepileptics	4.6	4.4	0.01	4	4	0
Antiinflammatory and antirheumatic products	29.3	28.9	0.01	28	30.8	-0.06
Antipsoriatics	1.3	1.3	0.01	1.4	1.3	0.01
Antithrombotic agents	18.6	16.4	0.06	15.1	14.9	0.01

Table S6.72. *Continued.* Selected baseline characteristics for Italy IQVIA, for the short-term risk of depression

Characteristic	Before propensity score matching			After propensity score matching		
	**Targets, %**	**Comparators, %**	**Standardized difference**	**Targets, %**	**Comparators, %**	**Standardized difference**
**Medication use**						
Beta blocking agents	14.6	16.1	-0.04	13.9	13.5	0.01
Calcium channel blockers	9.4	10.5	-0.04	9.5	8.6	0.03
Diuretics	14.1	16.3	-0.06	13.6	13.2	0.01
Drugs for acid-related disorders	26.1	24.3	0.04	23.8	24.6	-0.02
Drugs for obstructive airway diseases	19.6	15.9	0.1	18.4	20.6	-0.06
Immunosuppressants	0.9	0.8	0.01	1	0.7	0.03
Lipid modifying agents	14.2	16.8	-0.07	14	13.6	0.01
Opioids	7.8	6.9	0.04	7.3	8	-0.03
Psycholeptics	12.4	11.6	0.03	10.1	10.4	-0.01
Psychostimulants, agents used for ADHD and nootropics	1	0.8	0.02	0.8	1.2	-0.04

Table S6.73. Selected baseline characteristics for Italy IQVIA, for the short-term risk of anxiety disorders

Characteristic	Before propensity score matching			After propensity score matching		
	**Targets, %**	**Comparators, %**	**Standardized difference**	**Targets, %**	**Comparators, %**	**Standardized difference**
**Age group (years)**						
10-14	0.5	0.5	-0.01	0.4	0.7	-0.04
15-19	2.8	3.8	-0.06	3	3.3	-0.02
20-24	4.8	4.7	0	4.5	4.9	-0.02
25-29	5.6	4.8	0.03	4.9	5.4	-0.02
30-34	5.9	5.1	0.04	5.7	5.9	-0.01
35-39	6.5	5.6	0.04	5.9	6.4	-0.02
40-44	8	6.7	0.05	8.1	8.3	-0.01
45-49	10.4	8.3	0.07	10.2	10.9	-0.02
55-59	11.7	9.6	0.07	11.7	10	0.06
60-64	8.6	8.7	0	8.8	8.6	0.01
65-69	6.2	8.1	-0.07	6.5	6.5	0
70-74	5.8	8.2	-0.09	6.2	5.7	0.02
75-79	4	6	-0.09	4.5	4.3	0.01
80-84	4	5.4	-0.07	4.5	4.3	0.01
85-89	2.7	3.4	-0.04	2.6	2.5	0
90-94	1.4	1.5	0	1.2	1.3	-0.01
95-99	0.4	0.2	0.03	0.3	0.2	0.02
**Sex**						
Female	51.9	54.5	-0.05	52.3	52.5	0

Table S6.73. *Continued.* Selected baseline characteristics for Italy IQVIA, for the short-term risk of anxiety disorders

Characteristic	Before propensity score matching			After propensity score matching		
	Targets, %	Comparators, %	Standardized difference	Targets, %	Comparators, %	Standardized difference
**Medical history (general)**						
Acute respiratory disease	24	13.3	0.28	25.2	30.6	-0.12
Chronic liver disease	1.9	1.8	0.01	2.1	2.2	-0.01
Chronic obstructive lung disease	1.8	1.7	0.01	1.9	1.7	0.01
Crohn’s disease	0.3	0.2	0.01	0.3	0.3	0
Dementia	0.8	0.7	0.02	0.7	0.7	0
Depressive disorder	6.6	6.2	0.02	6.9	7.4	-0.02
Diabetes mellitus	7.7	8.5	-0.03	7.8	8	-0.01
Gastroesophageal reflux disease	9	7.7	0.05	9	9.6	-0.02
Gastrointestinal hemorrhage	0.3	0.2	0	0.3	0.3	-0.01
Human immunodeficiency virus infection	0.1	0.1	0.02	0.1	0.1	0.01
Hyperlipidemia	10.8	11.3	-0.02	11.5	10.9	0.02
Hypertensive disorder	25.4	27.1	-0.04	26	25.1	0.02
Lesion of liver	0.3	0.3	-0.01	0.3	0.4	-0.02
Obesity	0.5	0.3	0.03	0.3	0.4	-0.01
Osteoarthritis	7.7	8.1	-0.02	8	8.5	-0.02
Pneumonia	9.7	0.9	0.4	4.4	3.8	0.03
Psoriasis	1.7	1.4	0.02	1.6	1.6	0
Renal impairment	2.1	1.9	0.02	2.4	2.4	0
Rheumatoid arthritis	0.6	0.7	-0.01	0.6	0.5	0.01
Schizophrenia	0.1	0.2	-0.02	0.2	0.2	-0.01
Ulcerative colitis	0.4	0.3	0.02	0.5	0.4	0.02
Urinary tract infectious disease	2.8	2.1	0.04	2.8	3.2	-0.03
Viral hepatitis C	0.4	0.4	0	0.5	0.4	0

Table S6.73. *Continued.* Selected baseline characteristics for Italy IQVIA, for the short-term risk of anxiety disorders

**Characteristic**	**Before propensity score matching**			**After propensity score matching**		
	**Targets, %**	**Comparators, %**	**Standardized difference**	**Targets, %**	**Comparators, %**	**Standardized difference**
**Medical history (cardiovascular disease)**						
Atrial fibrillation	3	2.9	0	3.1	2.9	0.01
Cerebrovascular disease	3.8	4	-0.01	3.8	3.7	0
Coronary arteriosclerosis	0.4	0.3	0.02	0.4	0.4	0
Heart disease	12.3	12	0.01	12.6	12.8	0
Heart failure	1.2	1.2	0.01	1.3	1.3	-0.01
Ischemic heart disease	3.8	3.8	0	3.8	3.9	-0.01
Peripheral vascular disease	0.5	0.6	-0.01	0.5	0.5	0
Pulmonary embolism	0.3	0.2	0.02	0.3	0.3	0
Venous thrombosis	0.5	0.4	0.01	0.6	0.7	-0.02
**Medical history (neoplasms)**						
Malignant lymphoma	0.4	0.3	0.01	0.3	0.4	-0.01
Malignant neoplasm of anorectum	0.1	0.2	0	0.2	0.2	0
Malignant neoplastic disease	6.2	6.2	0	6.1	6	0
Malignant tumor of breast	1.4	1.5	-0.01	1.3	1.1	0.02
Malignant tumor of colon	0.4	0.5	-0.01	0.4	0.4	0.01
Malignant tumor of urinary bladder	0.4	0.4	0	0.4	0.3	0.01
Primary malignant neoplasm of prostate	0.8	0.7	0	0.8	0.6	0.02
**Medication use**						
Agents acting on the renin-angiotensin system	20.6	23.4	-0.07	21.3	20.8	0.01
Antibacterials for systemic use	48.3	32.2	0.33	41.7	44.9	-0.06
Antidepressants	10.7	9.8	0.03	10.6	10.7	0
Antiepileptics	4.6	4.4	0.01	4.9	5	-0.01
Antiinflammatory and antirheumatic products	29.3	28.9	0.01	29.4	31.8	-0.05
Antineoplastic agents	2.1	2.2	-0.01	2.1	2.2	-0.01
Antipsoriatics	1.3	1.3	0.01	1.4	1.3	0.01

Table S6.73. *Continued.* Selected baseline characteristics for Italy IQVIA, for the short-term risk of anxiety disorders

Characteristic	Before propensity score matching			After propensity score matching		
	Targets, %	Comparators, %	Standardized difference	Targets, %	Comparators, %	Standardized difference
**Medication use**						
Antithrombotic agents	18.6	16.4	0.06	16.6	15.9	0.02
Beta blocking agents	14.6	15.9	-0.04	15	14.4	0.02
Calcium channel blockers	9.4	10.5	-0.04	10	9.1	0.03
Diuretics	14.1	16.2	-0.06	14.6	14.1	0.01
Drugs for acid-related disorders	26.1	24.3	0.04	25.7	26	-0.01
Drugs for obstructive airway diseases	19.6	16	0.09	19	21.2	-0.05
Drugs used in diabetes	6.7	7.6	-0.04	6.9	6.8	0
Immunosuppressants	0.9	0.8	0.01	1	0.8	0.02
Lipid modifying agents	14.2	16.8	-0.07	14.9	14.4	0.01
Opioids	7.8	6.9	0.04	8.1	8.6	-0.02
Psycholeptics	12.4	11.6	0.03	12.7	12.8	0
Psychostimulants, agents used for ADHD and nootropics	1	0.8	0.02	0.9	1.2	-0.04

Table S6.74. Selected baseline characteristics for Italy IQVIA, for the short-term risk of alcohol misuse or dependence

Characteristic	Before propensity score matching			After propensity score matching		
	**Targets, %**	**Comparators, %**	**Standardized difference**	**Targets, %**	**Comparators, %**	**Standardized difference**
**Age group (years)**						
10-14	0.5	0.5	-0.01	0.4	0.7	-0.04
15-19	2.8	3.8	-0.06	3	3.3	-0.02
20-24	4.8	4.7	0.01	4.6	4.8	-0.01
25-29	5.6	4.8	0.03	4.9	5.4	-0.02
30-34	5.9	5.1	0.04	5.7	5.9	-0.01
35-39	6.5	5.6	0.04	5.9	6.4	-0.02
40-44	8	6.7	0.05	8	8.3	-0.01
45-49	10.4	8.1	0.08	10.3	10.9	-0.02
55-59	11.7	9.5	0.07	11.8	10	0.06
60-64	8.6	8.9	-0.01	8.9	8.6	0.01
70-74	5.8	8.2	-0.09	6.2	5.7	0.02
75-79	4	6.2	-0.1	4.5	4.3	0.01
80-84	4	5.5	-0.07	4.4	4.2	0.01
85-89	2.7	3.3	-0.04	2.5	2.5	0
90-94	1.4	1.5	-0.01	1.2	1.3	-0.01
95-99	0.4	0.2	0.03	0.3	0.2	0.02
**Sex**						
Female	51.9	54.4	-0.05	52.4	52.7	-0.01

Table S6.74. *Continued.* Selected baseline characteristics for Italy IQVIA, for the short-term risk of alcohol misuse or dependence

Characteristic	Before propensity score matching			After propensity score matching		
	Targets, %	Comparators, %	Standardized difference	Targets, %	Comparators, %	Standardized difference
**Medical history (general)**						
Acute respiratory disease	24	13.3	0.28	25.4	30.8	-0.12
Chronic liver disease	1.9	1.8	0	2	2.2	-0.01
Chronic obstructive lung disease	1.8	1.7	0.01	1.9	1.7	0.01
Crohn's disease	0.3	0.2	0.01	0.3	0.3	-0.01
Depressive disorder	6.6	6.2	0.02	6.9	7.4	-0.02
Diabetes mellitus	7.7	8.6	-0.03	7.8	7.9	0
Gastroesophageal reflux disease	9	7.6	0.05	9.1	9.7	-0.02
Gastrointestinal hemorrhage	0.3	0.3	0	0.3	0.4	-0.01
Human immunodeficiency virus infection	0.1	0.1	0.02	0.1	0.1	0.01
Hyperlipidemia	10.8	11.3	-0.02	11.5	11	0.01
Hypertensive disorder	25.4	27.3	-0.04	26	25.1	0.02
Lesion of liver	0.3	0.3	-0.01	0.3	0.4	-0.02
Obesity	0.5	0.3	0.03	0.4	0.4	-0.01
Osteoarthritis	7.7	8.2	-0.02	8	8.6	-0.02
Pneumonia	9.7	0.9	0.4	4.4	3.8	0.03
Psoriasis	1.7	1.4	0.02	1.6	1.6	0
Renal impairment	2.1	1.9	0.02	2.4	2.4	0
Rheumatoid arthritis	0.6	0.6	0	0.6	0.5	0.01
Schizophrenia	0.1	0.2	-0.02	0.2	0.2	-0.01
Ulcerative colitis	0.4	0.3	0.02	0.5	0.4	0.01
Urinary tract infectious disease	2.8	2.1	0.04	2.8	3.3	-0.03
Viral hepatitis C	0.4	0.4	0.01	0.5	0.4	0

Table S6.74. *Continued.* Selected baseline characteristics for Italy IQVIA, for the short-term risk of alcohol misuse or dependence

Characteristic	Before propensity score matching			After propensity score matching		
	Targets, %	Comparators, %	Standardized difference	Targets, %	Comparators, %	Standardized difference
**Medical history (cardiovascular disease)**						
Atrial fibrillation	3	3	0	3.1	2.9	0.01
Cerebrovascular disease	3.8	3.9	-0.01	3.7	3.7	0
Heart failure	1.2	1.2	0	1.3	1.3	-0.01
Ischemic heart disease	3.8	3.8	0	3.8	3.9	-0.01
Peripheral vascular disease	0.5	0.6	-0.01	0.5	0.5	0
Pulmonary embolism	0.3	0.2	0.02	0.3	0.3	0
Venous thrombosis	0.5	0.4	0.01	0.6	0.7	-0.02
**Medical history (neoplasms)**						
Malignant lymphoma	0.4	0.3	0.01	0.3	0.4	-0.01
Malignant neoplasm of anorectum	0.1	0.2	0	0.1	0.2	0
Malignant neoplastic disease	6.2	6.1	0	6.1	6	0
Malignant tumor of breast	1.4	1.5	-0.01	1.4	1.1	0.02
Malignant tumor of colon	0.4	0.5	0	0.4	0.4	0.01
Malignant tumor of urinary bladder	0.4	0.4	0	0.4	0.3	0.01
Primary malignant neoplasm of prostate	0.8	0.8	0	0.8	0.6	0.03
**Medication use**						
Agents acting on the renin-angiotensin system	20.6	23.6	-0.07	21.4	20.7	0.02
Antibacterials for systemic use	48.3	32.1	0.33	41.8	44.9	-0.06
Antidepressants	10.7	9.8	0.03	11.2	11.3	0
Antiepileptics	4.6	4.4	0.01	4.9	5.1	-0.01
Antiinflammatory and antirheumatic products	29.3	29.1	0.01	29.4	31.9	-0.05
Antineoplastic agents	2.1	2.2	-0.01	2.1	2.1	-0.01
Antipsoriatics	1.3	1.3	0	1.4	1.3	0.01
Antithrombotic agents	18.6	16.5	0.05	16.6	15.8	0.02
Beta blocking agents	14.6	16.1	-0.04	15.1	14.4	0.02

Table S6.74. *Continued.* Selected baseline characteristics for Italy IQVIA, for the short-term risk of alcohol misuse or dependence

Characteristic	Before propensity score matching			After propensity score matching		
	Targets, %	Comparators, %	Standardized difference	Targets, %	Comparators, %	Standardized difference
**Medication use**						
Calcium channel blockers	9.4	10.6	-0.04	10	9.1	0.03
Diuretics	14.1	16.4	-0.06	14.6	14.1	0.01
Drugs for acid-related disorders	26.1	24.3	0.04	25.7	26.1	-0.01
Drugs for obstructive airway diseases	19.6	16	0.09	19	21.2	-0.06
Drugs used in diabetes	6.7	7.7	-0.04	6.8	6.8	0
Immunosuppressants	0.9	0.8	0.01	1	0.8	0.02
Lipid modifying agents	14.2	16.9	-0.07	14.9	14.4	0.01
Opioids	7.8	7	0.03	8.1	8.7	-0.02
Psycholeptics	12.4	11.5	0.03	13	13.3	-0.01
Psychostimulants, agents used for ADHD and nootropics	1	0.8	0.02	0.9	1.3	-0.04

Table S6.75. Selected baseline characteristics for Italy IQVIA, for the short-term risk of substance misuse or dependence

**Characteristic**	**Before propensity score matching**			**After propensity score matching**		
	**Targets, %**	**Comparators, %**	**Standardized difference**	**Targets, %**	**Comparators, %**	**Standardized difference**
**Age group (years)**						
10-14	0.5	0.5	-0.01	0.4	0.7	-0.04
15-19	2.8	3.9	-0.06	3	3.3	-0.02
20-24	4.8	4.7	0	4.6	4.9	-0.01
25-29	5.6	4.9	0.03	4.9	5.4	-0.02
30-34	5.9	5	0.04	5.7	5.9	-0.01
35-39	6.5	5.6	0.04	5.9	6.4	-0.02
40-44	8	6.6	0.05	8	8.3	-0.01
45-49	10.4	8.2	0.07	10.3	10.9	-0.02
50-54	10.7	9.2	0.05	10.9	10.9	0
55-59	11.7	9.6	0.07	11.8	10	0.06
60-64	8.6	8.8	-0.01	8.9	8.5	0.01
70-74	5.8	8.2	-0.09	6.1	5.7	0.02
75-79	4	6	-0.09	4.5	4.3	0.01
80-84	4	5.5	-0.07	4.4	4.2	0.01
85-89	2.7	3.4	-0.04	2.5	2.5	0
90-94	1.4	1.5	0	1.2	1.3	-0.01
95-99	0.4	0.2	0.03	0.3	0.2	0.02
**Sex**						
Female	51.9	54.4	-0.05	52.4	52.8	-0.01

Table S6.75. *Continued.* Selected baseline characteristics for Italy IQVIA, for the short-term risk of substance misuse or dependence

Characteristic	Before propensity score matching			After propensity score matching		
	Targets, %	Comparators, %	Standardized difference	Targets, %	Comparators, %	Standardized difference
**Medical history (general)**						
Acute respiratory disease	24	13.3	0.28	25.3	30.8	-0.12
Chronic liver disease	1.9	1.8	0	2	2.2	-0.01
Chronic obstructive lung disease	1.8	1.8	0.01	1.9	1.7	0.01
Crohn’s disease	0.3	0.2	0.01	0.3	0.3	-0.01
Depressive disorder	6.6	6.1	0.02	6.9	7.4	-0.02
Diabetes mellitus	7.7	8.5	-0.03	7.8	7.9	0
Gastroesophageal reflux disease	9	7.6	0.05	9.1	9.7	-0.02
Gastrointestinal hemorrhage	0.3	0.3	0	0.3	0.3	-0.01
Human immunodeficiency virus infection	0.1	0.1	0.02	0.1	0.1	0.01
Hyperlipidemia	10.8	11.4	-0.02	11.5	11	0.02
Hypertensive disorder	25.4	27.2	-0.04	26	25.1	0.02
Lesion of liver	0.3	0.3	-0.01	0.3	0.4	-0.02
Obesity	0.5	0.3	0.03	0.3	0.4	-0.01
Osteoarthritis	7.7	8.2	-0.02	8.1	8.5	-0.02
Pneumonia	9.7	0.9	0.4	4.4	3.8	0.03
Psoriasis	1.7	1.4	0.03	1.6	1.6	0
Renal impairment	2.1	1.9	0.02	2.4	2.4	0
Rheumatoid arthritis	0.6	0.6	0	0.6	0.5	0.01
Schizophrenia	0.1	0.2	-0.02	0.2	0.2	-0.01
Ulcerative colitis	0.4	0.3	0.01	0.5	0.4	0.01
Urinary tract infectious disease	2.8	2.1	0.04	2.8	3.3	-0.03
Viral hepatitis C	0.4	0.4	0	0.5	0.4	0.01

Table S6.75. *Continued.* Selected baseline characteristics for Italy IQVIA, for the short-term risk of substance misuse or dependence

Characteristic	Before propensity score matching			After propensity score matching		
	Targets, %	Comparators, %	Standardized difference	Targets, %	Comparators, %	Standardized difference
**Medical history (cardiovascular disease)**						
Cerebrovascular disease	3.8	3.9	-0.01	3.7	3.6	0
Heart disease	12.3	12.2	0	12.6	12.7	0
Heart failure	1.2	1.2	0.01	1.3	1.3	-0.01
Ischemic heart disease	3.8	3.8	0	3.8	3.9	-0.01
Peripheral vascular disease	0.5	0.6	-0.01	0.5	0.5	0
Pulmonary embolism	0.3	0.2	0.02	0.3	0.3	0
Venous thrombosis	0.5	0.5	0.01	0.6	0.7	-0.02
**Medical history (neoplasms)**						
Malignant lymphoma	0.4	0.3	0	0.3	0.4	-0.02
Malignant neoplasm of anorectum	0.1	0.2	0	0.1	0.2	0
Malignant neoplastic disease	6.2	6.1	0	6.1	6	0
Malignant tumor of breast	1.4	1.5	-0.01	1.4	1.1	0.02
Malignant tumor of colon	0.4	0.5	-0.01	0.4	0.4	0.01
Malignant tumor of urinary bladder	0.4	0.4	0	0.4	0.3	0.01
Primary malignant neoplasm of prostate	0.8	0.8	0	0.8	0.6	0.02
**Medication use**						
Agents acting on the renin-angiotensin system	20.6	23.6	-0.07	21.4	20.7	0.02
Antibacterials for systemic use	48.3	32.1	0.33	41.8	44.9	-0.06
Antidepressants	10.7	9.8	0.03	11.1	11.3	0
Antiepileptics	4.6	4.4	0.01	4.9	5	0
Antiinflammatory and antirheumatic products	29.3	28.9	0.01	29.4	31.8	-0.05
Antineoplastic agents	2.1	2.2	-0.01	2.1	2.1	-0.01
Antipsoriatics	1.3	1.3	0.01	1.4	1.3	0.01
Antithrombotic agents	18.6	16.4	0.06	16.6	15.7	0.02
Beta blocking agents	14.6	16.1	-0.04	15.1	14.4	0.02

Table S6.75. *Continued.* Selected baseline characteristics for Italy IQVIA, for the short-term risk of substance misuse or dependence

Characteristic	Before propensity score matching			After propensity score matching		
	Targets, %	Comparators, %	Standardized difference	Targets, %	Comparators, %	Standardized difference
**Medication use**						
Calcium channel blockers	9.4	10.5	-0.04	10	9.1	0.03
Diuretics	14.1	16.3	-0.06	14.6	14.1	0.01
Drugs for acid-related disorders	26.1	24.3	0.04	25.7	26.1	-0.01
Drugs for obstructive airway diseases	19.6	16	0.09	18.9	21.2	-0.06
Drugs used in diabetes	6.7	7.6	-0.04	6.8	6.8	0
Immunosuppressants	0.9	0.8	0.01	1	0.8	0.03
Lipid modifying agents	14.2	16.8	-0.07	14.9	14.4	0.01
Opioids	7.8	6.9	0.04	8.1	8.6	-0.02
Psycholeptics	12.4	11.5	0.03	13	13.3	-0.01
Psychostimulants, agents used for ADHD and nootropics	1	0.8	0.03	0.9	1.3	-0.04

Table S6.76. Selected baseline characteristics for Italy IQVIA, for the short-term risk of bipolar disorders

Characteristic	Before propensity score matching			After propensity score matching		
	**Targets, %**	**Comparators, %**	**Standardized difference**	**Targets, %**	**Comparators, %**	**Standardized difference**
**Age group (years)**						
10-14	0.5	0.5	-0.01	0.4	0.7	-0.04
15-19	2.8	3.9	-0.06	3	3.3	-0.02
20-24	4.8	4.7	0	4.6	4.9	-0.01
25-29	5.6	4.9	0.03	4.9	5.4	-0.02
30-34	5.9	5.1	0.04	5.7	5.9	-0.01
35-39	6.5	5.6	0.04	5.9	6.4	-0.02
40-44	8	6.7	0.05	8	8.3	-0.01
45-49	10.4	8.1	0.08	10.3	10.8	-0.02
50-54	10.7	9.2	0.05	10.8	10.9	0
55-59	11.7	9.6	0.07	11.9	10	0.06
60-64	8.6	8.8	-0.01	8.9	8.6	0.01
65-69	6.2	8.1	-0.08	6.5	6.5	0
70-74	5.8	8.2	-0.09	6.2	5.7	0.02
75-79	4	6	-0.09	4.5	4.3	0.01
80-84	4	5.4	-0.07	4.4	4.3	0.01
85-89	2.7	3.4	-0.04	2.5	2.5	0
90-94	1.4	1.5	-0.01	1.2	1.3	-0.01
95-99	0.4	0.2	0.03	0.3	0.2	0.02
**Sex**						
Female	51.9	54.4	-0.05	52.3	52.6	-0.01

Table S6.76. *Continued.* Selected baseline characteristics for Italy IQVIA, for the short-term risk of bipolar disorders

Characteristic	Before propensity score matching			After propensity score matching		
	Targets, %	Comparators, %	Standardized difference	Targets, %	Comparators, %	Standardized difference
**Medical history (general)**						
Acute respiratory disease	24	13.2	0.28	25.3	30.8	-0.12
Chronic liver disease	1.9	1.8	0.01	2.1	2.2	-0.01
Chronic obstructive lung disease	1.8	1.8	0.01	1.9	1.7	0.01
Crohn’s disease	0.3	0.2	0.01	0.3	0.3	-0.01
Dementia	0.8	0.7	0.02	0.7	0.7	0
Depressive disorder	6.6	6.1	0.02	6.8	7.3	-0.02
Diabetes mellitus	7.7	8.5	-0.03	7.8	7.9	0
Gastroesophageal reflux disease	9	7.6	0.05	9.1	9.7	-0.02
Gastrointestinal hemorrhage	0.3	0.3	0	0.3	0.4	-0.01
Human immunodeficiency virus infection	0.1	0.1	0.02	0.1	0.1	0.01
Hyperlipidemia	10.8	11.3	-0.02	11.5	11	0.01
Hypertensive disorder	25.4	27.1	-0.04	26	25.1	0.02
Lesion of liver	0.3	0.3	0	0.3	0.4	-0.02
Obesity	0.5	0.3	0.03	0.4	0.4	0
Osteoarthritis	7.7	8.2	-0.02	8	8.6	-0.02
Pneumonia	9.7	0.9	0.4	4.4	3.8	0.03
Psoriasis	1.7	1.4	0.02	1.6	1.6	0
Renal impairment	2.1	1.9	0.02	2.4	2.4	0
Rheumatoid arthritis	0.6	0.7	-0.01	0.6	0.5	0.01
Schizophrenia	0.1	0.2	-0.02	0.1	0.2	-0.02
Ulcerative colitis	0.4	0.3	0.02	0.5	0.4	0.01
Urinary tract infectious disease	2.8	2.1	0.04	2.8	3.3	-0.03
Viral hepatitis C	0.4	0.4	0	0.5	0.4	0

Table S6.76. *Continued.* Selected baseline characteristics for Italy IQVIA, for the short-term risk of bipolar disorders

Characteristic	Before propensity score matching			After propensity score matching		
	Targets, %	Comparators, %	Standardized difference	Targets, %	Comparators, %	Standardized difference
**Medical history (cardiovascular disease)**						
Atrial fibrillation	3	3	0	3.1	2.9	0.01
Cerebrovascular disease	3.8	4	-0.01	3.7	3.7	0
Heart disease	12.3	12.2	0	12.6	12.8	0
Heart failure	1.2	1.2	0	1.3	1.3	-0.01
Ischemic heart disease	3.8	3.8	0	3.8	3.9	0
Peripheral vascular disease	0.5	0.6	-0.01	0.5	0.5	-0.01
Pulmonary embolism	0.3	0.2	0.02	0.3	0.3	0
Venous thrombosis	0.5	0.5	0.01	0.6	0.7	-0.02
**Medical history (neoplasms)**						
Malignant lymphoma	0.4	0.3	0	0.3	0.4	-0.02
Malignant neoplasm of anorectum	0.1	0.2	0	0.2	0.2	0
Malignant neoplastic disease	6.2	6.1	0.01	6.1	6	0
Malignant tumor of breast	1.4	1.5	-0.01	1.4	1.1	0.02
Malignant tumor of colon	0.4	0.5	-0.01	0.4	0.4	0.01
Primary malignant neoplasm of prostate	0.8	0.7	0	0.8	0.6	0.02
**Medication use**						
Agents acting on the renin-angiotensin system	20.6	23.5	-0.07	21.4	20.7	0.02
Antibacterials for systemic use	48.3	32.1	0.34	41.7	44.9	-0.06
Antidepressants	10.7	9.7	0.04	11	11.2	0
Antiepileptics	4.6	4.4	0.01	4.8	4.9	0
Antiinflammatory and antirheumatic products	29.3	29	0.01	29.4	31.9	-0.06
Antineoplastic agents	2.1	2.2	-0.01	2.1	2.1	-0.01
Antipsoriatics	1.3	1.3	0	1.4	1.3	0.01
Antithrombotic agents	18.6	16.4	0.06	16.6	15.8	0.02
Beta blocking agents	14.6	16	-0.04	15	14.4	0.02

Table S6.76. *Continued.* Selected baseline characteristics for Italy IQVIA, for the short-term risk of bipolar disorders

Characteristic	Before propensity score matching			After propensity score matching		
	Targets, %	Comparators, %	Standardized difference	Targets, %	Comparators, %	Standardized difference
**Medication use**						
Calcium channel blockers	9.4	10.5	-0.04	10.1	9.1	0.03
Diuretics	14.1	16.2	-0.06	14.6	14.1	0.01
Drugs for acid-related disorders	26.1	24.2	0.04	25.7	26.1	-0.01
Drugs for obstructive airway diseases	19.6	16	0.09	19	21.2	-0.06
Drugs used in diabetes	6.7	7.7	-0.04	6.8	6.8	0
Immunosuppressants	0.9	0.8	0.01	1	0.8	0.02
Lipid modifying agents	14.2	16.8	-0.07	14.9	14.4	0.01
Opioids	7.8	6.8	0.04	8.1	8.7	-0.02
Psycholeptics	12.4	11.4	0.03	12.8	13.1	-0.01
Psychostimulants, agents used for ADHD and nootropics	1	0.8	0.03	0.9	1.3	-0.04

Table S6.77. Selected baseline characteristics for Italy IQVIA, for the short-term risk of psychoses

Characteristic	Before propensity score matching			After propensity score matching		
	Targets, %	Comparators, %	Standardized difference	Targets, %	Comparators, %	Standardized difference
**Age group (years)**						
10-14	0.5	0.5	-0.01	0.4	0.7	-0.04
15-19	2.8	3.8	-0.06	3	3.3	-0.02
20-24	4.8	4.7	0.01	4.6	4.9	-0.01
25-29	5.6	4.8	0.04	4.9	5.4	-0.02
30-34	5.9	5.1	0.04	5.7	6	-0.01
35-39	6.5	5.5	0.04	5.9	6.4	-0.02
40-44	8	6.7	0.05	8	8.3	-0.01
45-49	10.4	8.2	0.07	10.3	10.9	-0.02
50-54	10.7	9.2	0.05	10.9	10.9	0
55-59	11.7	9.6	0.07	11.9	10	0.06
60-64	8.6	8.8	-0.01	8.8	8.6	0.01
65-69	6.2	8.1	-0.07	6.5	6.5	0
70-74	5.8	8.3	-0.1	6.1	5.7	0.02
75-79	4	6.1	-0.1	4.5	4.3	0.01
80-84	4	5.5	-0.07	4.4	4.2	0.01
85-89	2.7	3.4	-0.04	2.5	2.5	0
90-94	1.4	1.5	-0.01	1.2	1.3	-0.01
95-99	0.4	0.2	0.03	0.3	0.2	0.02
**Sex**						
Female	51.9	54.6	-0.05	52.3	52.7	-0.01

Table S6.77. *Continued.* Selected baseline characteristics for Italy IQVIA, for the short-term risk of psychoses

Characteristic	Before propensity score matching			After propensity score matching		
	Targets, %	Comparators, %	Standardized difference	Targets, %	Comparators, %	Standardized difference
**Medical history (general)**						
Acute respiratory disease	24	13.2	0.28	25.4	30.8	-0.12
Chronic liver disease	1.9	1.8	0	2	2.2	-0.01
Chronic obstructive lung disease	1.8	1.8	0.01	1.9	1.7	0.01
Crohn’s disease	0.3	0.2	0.01	0.3	0.3	0
Dementia	0.8	0.7	0.02	0.7	0.6	0
Depressive disorder	6.6	6.2	0.02	6.9	7.4	-0.02
Diabetes mellitus	7.7	8.5	-0.03	7.7	7.9	-0.01
Gastroesophageal reflux disease	9	7.6	0.05	9.1	9.7	-0.02
Gastrointestinal hemorrhage	0.3	0.2	0	0.3	0.4	-0.01
Human immunodeficiency virus infection	0.1	0.1	0.02	0.1	0.1	0.01
Hyperlipidemia	10.8	11.4	-0.02	11.5	10.9	0.02
Hypertensive disorder	25.4	27.1	-0.04	26	25.1	0.02
Lesion of liver	0.3	0.3	-0.01	0.3	0.4	-0.02
Obesity	0.5	0.3	0.03	0.4	0.4	0
Osteoarthritis	7.7	8.1	-0.02	8	8.6	-0.02
Pneumonia	9.7	0.9	0.4	4.4	3.8	0.03
Psoriasis	1.7	1.4	0.03	1.6	1.6	0
Renal impairment	2.1	1.9	0.02	2.3	2.4	0
Rheumatoid arthritis	0.6	0.6	-0.01	0.6	0.5	0.01
Ulcerative colitis	0.4	0.3	0.01	0.5	0.4	0.02
Urinary tract infectious disease	2.8	2.1	0.04	2.8	3.2	-0.03
Viral hepatitis C	0.4	0.4	0	0.5	0.4	0

Table S6.77. *Continued.* Selected baseline characteristics for Italy IQVIA, for the short-term risk of psychoses

**Characteristic**	**Before propensity score matching**			**After propensity score matching**		
	**Targets, %**	**Comparators, %**	**Standardized difference**	**Targets, %**	**Comparators, %**	**Standardized difference**
Medical history: Cardiovascular disease						
Cerebrovascular disease	3.8	3.9	-0.01	3.7	3.7	0
Coronary arteriosclerosis	0.4	0.3	0.02	0.4	0.4	0
Heart disease	12.3	12.2	0	12.6	12.8	0
Heart failure	1.2	1.2	0.01	1.2	1.3	0
Peripheral vascular disease	0.5	0.6	-0.01	0.5	0.5	0
Pulmonary embolism	0.3	0.2	0.02	0.3	0.3	0
Venous thrombosis	0.5	0.5	0.01	0.6	0.7	-0.02
**Medical history (neoplasms)**						
Malignant lymphoma	0.4	0.3	0.01	0.3	0.4	-0.02
Malignant neoplastic disease	6.2	6.1	0	6.1	6	0.01
Malignant tumor of breast	1.4	1.5	-0.01	1.4	1.1	0.02
Malignant tumor of colon	0.4	0.5	-0.01	0.4	0.4	0.01
Malignant tumor of urinary bladder	0.4	0.4	0	0.4	0.3	0.01
Primary malignant neoplasm of prostate	0.8	0.7	0	0.8	0.6	0.03
**Medication use**						
Agents acting on the renin-angiotensin system	20.6	23.6	-0.07	21.4	20.7	0.02
Antibacterials for systemic use	48.3	32.1	0.34	41.8	44.9	-0.06
Antidepressants	10.7	9.8	0.03	11	11.2	-0.01
Antiepileptics	4.6	4.4	0.01	4.8	4.9	-0.01
Antiinflammatory and antirheumatic products	29.3	28.9	0.01	29.4	31.9	-0.06
Antineoplastic agents	2.1	2.2	-0.01	2.1	2.1	0
Antipsoriatics	1.3	1.2	0.01	1.5	1.3	0.02
Antithrombotic agents	18.6	16.5	0.05	16.6	15.7	0.02
Beta blocking agents	14.6	16.1	-0.04	15.1	14.5	0.02
Calcium channel blockers	9.4	10.5	-0.04	10.1	9.1	0.03

Table S6.77. *Continued.* Selected baseline characteristics for Italy IQVIA, for the short-term risk of psychoses

Characteristic	Before propensity score matching			After propensity score matching		
	**Targets, %**	**Comparators, %**	**Standardized difference**	**Targets, %**	**Comparators, %**	**Standardized difference**
**Medication use**						
Diuretics	14.1	16.4	-0.06	14.5	14.1	0.01
Drugs for acid-related disorders	26.1	24.3	0.04	25.8	26	-0.01
Drugs for obstructive airway diseases	19.6	16	0.09	19.1	21.2	-0.05
Drugs used in diabetes	6.7	7.7	-0.04	6.8	6.8	0
Immunosuppressants	0.9	0.8	0.01	1	0.8	0.02
Lipid modifying agents	14.2	16.8	-0.07	14.9	14.4	0.01
Opioids	7.8	6.9	0.03	8.1	8.7	-0.02
Psycholeptics	12.4	11.6	0.03	12.7	13	-0.01
Psychostimulants, agents used for ADHD and nootropics	1	0.8	0.02	0.9	1.3	-0.04

Table S6.78. Selected baseline characteristics for Italy IQVIA, for the short-term risk of personality disorders

Characteristic	Before propensity score matching			After propensity score matching		
	**Targets, %**	**Comparators, %**	**Standardized difference**	**Targets, %**	**Comparators, %**	**Standardized difference**
**Age group (years)**						
10-14	0.5	0.5	-0.01	0.4	0.7	-0.04
15-19	2.8	3.8	-0.06	3	3.3	-0.02
20-24	4.8	4.7	0	4.6	4.9	-0.01
25-29	5.6	4.8	0.03	4.9	5.4	-0.03
30-34	5.9	5.1	0.04	5.7	6	-0.01
35-39	6.5	5.6	0.04	6	6.5	-0.02
40-44	8	6.7	0.05	8.1	8.5	-0.01
45-49	10.4	8.2	0.08	10.4	11	-0.02
50-54	10.7	9.2	0.05	11	11	0
55-59	11.7	9.6	0.07	11.9	10.1	0.06
60-64	8.6	8.8	-0.01	8.9	8.6	0.01
65-69	6.2	8.2	-0.08	6.6	6.5	0
70-74	5.8	8.1	-0.09	6.2	5.6	0.02
75-79	4	6	-0.09	4.4	4.2	0.01
80-84	4	5.5	-0.07	4.2	4	0.01
85-89	2.7	3.4	-0.04	2.4	2.3	0
90-94	1.4	1.5	-0.01	1	1.2	-0.02
95-99	0.4	0.2	0.03	0.3	0.2	0.02
**Sex**						
Female	51.9	54.4	-0.05	52.2	52.6	-0.01

Table S6.78. *Continued.* Selected baseline characteristics for Italy IQVIA, for the short-term risk of personality disorders

Characteristic	Before propensity score matching			After propensity score matching		
	**Targets, %**	**Comparators, %**	**Standardized difference**	**Targets, %**	**Comparators, %**	**Standardized difference**
**Medical history (general)**						
Acute respiratory disease	24	13.2	0.28	25.5	30.8	-0.12
Chronic liver disease	1.9	1.8	0.01	2	2.1	-0.01
Chronic obstructive lung disease	1.8	1.8	0.01	1.8	1.7	0.01
Crohn’s disease	0.3	0.2	0.01	0.3	0.3	-0.01
Dementia	0.8	0.7	0.02	0.6	0.6	0
Depressive disorder	6.6	6.1	0.02	6.7	7.2	-0.02
Diabetes mellitus	7.7	8.6	-0.03	7.6	7.8	-0.01
Gastroesophageal reflux disease	9	7.6	0.05	9	9.5	-0.02
Gastrointestinal hemorrhage	0.3	0.2	0	0.3	0.4	-0.01
Hyperlipidemia	10.8	11.4	-0.02	11.4	10.8	0.02
Hypertensive disorder	25.4	27.3	-0.04	25.6	24.7	0.02
Lesion of liver	0.3	0.3	-0.01	0.3	0.3	-0.02
Obesity	0.5	0.3	0.03	0.4	0.4	-0.01
Osteoarthritis	7.7	8.3	-0.02	7.9	8.3	-0.01
Pneumonia	9.7	0.9	0.4	4.4	3.7	0.03
Psoriasis	1.7	1.4	0.02	1.6	1.6	0
Renal impairment	2.1	1.9	0.02	2.3	2.3	0
Rheumatoid arthritis	0.6	0.7	-0.01	0.6	0.5	0.01
Schizophrenia	0.1	0.2	-0.02	0.2	0.2	-0.01
Ulcerative colitis	0.4	0.3	0.01	0.5	0.4	0.02
Urinary tract infectious disease	2.8	2.1	0.05	2.7	3.2	-0.03
Viral hepatitis C	0.4	0.4	0.01	0.5	0.4	0.01

Table S6.78. *Continued.* Selected baseline characteristics for Italy IQVIA, for the short-term risk of personality disorders

Characteristic	Before propensity score matching			After propensity score matching		
	Targets, %	Comparators, %	Standardized difference	Targets, %	Comparators, %	Standardized difference
**Medical history (cardiovascular disease)**						
Atrial fibrillation	3	2.9	0	3	2.7	0.01
Cerebrovascular disease	3.8	4	-0.01	3.5	3.5	0
Coronary arteriosclerosis	0.4	0.3	0.01	0.3	0.4	0
Heart disease	12.3	12.2	0	12.2	12.4	-0.01
Heart failure	1.2	1.2	0	1.2	1.3	0
Ischemic heart disease	3.8	3.9	0	3.7	3.9	-0.01
Peripheral vascular disease	0.5	0.6	-0.01	0.5	0.5	0
Pulmonary embolism	0.3	0.2	0.02	0.3	0.2	0.01
Venous thrombosis	0.5	0.5	0.01	0.5	0.7	-0.03
**Medical history (neoplasms)**						
Malignant lymphoma	0.4	0.3	0	0.3	0.4	-0.01
Malignant neoplastic disease	6.2	6.2	0	6	5.9	0.01
Malignant tumor of breast	1.4	1.5	-0.01	1.4	1.1	0.02
Malignant tumor of colon	0.4	0.5	-0.01	0.4	0.4	0.01
Malignant tumor of urinary bladder	0.4	0.4	0	0.4	0.3	0.01
Primary malignant neoplasm of prostate	0.8	0.8	0	0.8	0.6	0.02
**Medication use**						
Agents acting on the renin-angiotensin system	20.6	23.7	-0.08	21.1	20.4	0.02
Antibacterials for systemic use	48.3	32	0.34	41.5	44.7	-0.06
Antidepressants	10.7	9.8	0.03	10.8	10.8	0
Antiepileptics	4.6	4.5	0.01	4.8	4.9	0
Antiinflammatory and antirheumatic products	29.3	29.1	0.01	29.2	31.7	-0.05
Antineoplastic agents	2.1	2.2	-0.01	2	2.1	-0.01
Antipsoriatics	1.3	1.3	0	1.4	1.3	0.01
Antithrombotic agents	18.6	16.4	0.06	16.1	15.3	0.02

Table S6.78. *Continued.* Selected baseline characteristics for Italy IQVIA, for the short-term risk of personality disorders

**Characteristic**	**Before propensity score matching**			**After propensity score matching**		
	**Targets, %**	**Comparators, %**	**Standardized difference**	**Targets, %**	**Comparators, %**	**Standardized difference**
**Medication use**						
Beta blocking agents	14.6	16.1	-0.04	14.9	14.2	0.02
Calcium channel blockers	9.4	10.6	-0.04	9.9	8.9	0.03
Diuretics	14.1	16.3	-0.06	14.3	13.8	0.01
Drugs for acid-related disorders	26.1	24.2	0.04	25.4	25.7	-0.01
Drugs for obstructive airway diseases	19.6	15.9	0.1	18.9	21.1	-0.05
Immunosuppressants	0.9	0.8	0.01	1	0.8	0.02
Lipid modifying agents	14.2	16.9	-0.07	14.6	14.1	0.01
Opioids	7.8	6.8	0.04	8	8.5	-0.02
Psycholeptics	12.4	11.4	0.03	12.6	13	-0.01
Psychostimulants, agents used for ADHD and nootropics	1	0.8	0.02	0.9	1.2	-0.04

Table S6.79. Selected baseline characteristics for Italy IQVIA, for the short-term risk of self-harm and suicide

Characteristic	Before propensity score matching			After propensity score matching		
	**Targets, %**	**Comparators, %**	**Standardized difference**	**Targets, %**	**Comparators, %**	**Standardized difference**
**Age group (years)**						
10-14	0.5	0.5	-0.01	0.4	0.7	-0.04
15-19	2.8	3.9	-0.06	3	3.3	-0.02
20-24	4.8	4.7	0	4.6	4.8	-0.01
25-29	5.6	4.8	0.03	4.9	5.4	-0.02
30-34	5.9	5.1	0.04	5.7	5.9	-0.01
35-39	6.5	5.5	0.04	5.9	6.4	-0.02
40-44	8	6.7	0.05	8	8.3	-0.01
45-49	10.4	8.2	0.07	10.3	10.9	-0.02
50-54	10.7	9.1	0.05	10.8	10.9	0
55-59	11.7	9.6	0.07	11.8	10	0.06
60-64	8.6	8.9	-0.01	8.9	8.6	0.01
70-74	5.8	8.2	-0.09	6.2	5.7	0.02
75-79	4	6	-0.09	4.5	4.3	0.01
80-84	4	5.5	-0.07	4.4	4.2	0.01
85-89	2.7	3.4	-0.04	2.5	2.5	0
90-94	1.4	1.5	0	1.2	1.3	-0.01
95-99	0.4	0.2	0.03	0.3	0.2	0.02
**Sex**						
Female	51.9	54.5	-0.05	52.4	52.7	-0.01

Table S6.79. *Continued.* Selected baseline characteristics for Italy IQVIA, for the short-term risk of self-harm and suicide

Characteristic	Before propensity score matching			After propensity score matching		
	**Targets, %**	**Comparators, %**	**Standardized difference**	**Targets, %**	**Comparators, %**	**Standardized difference**
**Medical history (general)**						
Acute respiratory disease	24	13.2	0.28	25.4	30.8	-0.12
Chronic liver disease	1.9	1.8	0.01	2	2.2	-0.01
Chronic obstructive lung disease	1.8	1.8	0.01	1.9	1.7	0.01
Crohn’s disease	0.3	0.2	0.01	0.3	0.3	-0.01
Dementia	0.8	0.7	0.02	0.7	0.7	0
Depressive disorder	6.6	6.2	0.02	6.9	7.4	-0.02
Diabetes mellitus	7.7	8.5	-0.03	7.8	7.9	0
Gastroesophageal reflux disease	9	7.6	0.05	9.1	9.7	-0.02
Gastrointestinal hemorrhage	0.3	0.2	0	0.3	0.4	-0.01
Human immunodeficiency virus infection	0.1	0.1	0.02	0.1	0.1	0.01
Hyperlipidemia	10.8	11.4	-0.02	11.5	11	0.02
Hypertensive disorder	25.4	27.1	-0.04	26	25.1	0.02
Lesion of liver	0.3	0.3	0	0.3	0.4	-0.02
Obesity	0.5	0.3	0.03	0.4	0.4	-0.01
Osteoarthritis	7.7	8.1	-0.02	8	8.6	-0.02
Pneumonia	9.7	0.9	0.4	4.4	3.8	0.03
Psoriasis	1.7	1.4	0.02	1.6	1.6	0
Renal impairment	2.1	1.9	0.02	2.4	2.4	0
Rheumatoid arthritis	0.6	0.7	-0.01	0.6	0.5	0.01
Schizophrenia	0.1	0.2	-0.02	0.2	0.2	-0.01
Ulcerative colitis	0.4	0.3	0.02	0.5	0.4	0.02
Urinary tract infectious disease	2.8	2.1	0.04	2.8	3.3	-0.03
Viral hepatitis C	0.4	0.4	0	0.5	0.4	0

Table S6.79. *Continued.* Selected baseline characteristics for Italy IQVIA, for the short-term risk of self-harm and suicide

Characteristic	Before propensity score matching			After propensity score matching		
	Targets, %	Comparators, %	Standardized difference	Targets, %	Comparators, %	Standardized difference
**Medical history (cardiovascular disease)**						
Atrial fibrillation	3	3	0	3.1	2.9	0.01
Cerebrovascular disease	3.8	3.9	-0.01	3.7	3.7	0
Heart disease	12.3	12.2	0	12.6	12.8	-0.01
Heart failure	1.2	1.2	0	1.3	1.3	-0.01
Ischemic heart disease	3.8	3.8	0	3.8	3.9	-0.01
Peripheral vascular disease	0.5	0.6	-0.01	0.5	0.5	0
Pulmonary embolism	0.3	0.2	0.02	0.3	0.3	0
Venous thrombosis	0.5	0.4	0.01	0.6	0.7	-0.02
**Medical history (neoplasms)**						
Malignant lymphoma	0.4	0.3	0.01	0.3	0.4	-0.02
Malignant neoplastic disease	6.2	6.1	0	6.1	6	0
Malignant tumor of breast	1.4	1.5	-0.01	1.4	1.1	0.02
Malignant tumor of colon	0.4	0.5	-0.01	0.4	0.4	0.01
Malignant tumor of urinary bladder	0.4	0.4	0	0.4	0.3	0.01
Primary malignant neoplasm of prostate	0.8	0.8	0	0.8	0.6	0.03
**Medication use**						
Agents acting on the renin-angiotensin system	20.6	23.6	-0.07	21.4	20.7	0.02
Antibacterials for systemic use	48.3	32	0.34	41.8	44.9	-0.06
Antidepressants	10.7	9.9	0.03	11.2	11.3	0
Antiepileptics	4.6	4.4	0.01	4.9	5.1	-0.01
Antiinflammatory and antirheumatic products	29.3	29	0.01	29.4	31.9	-0.06
Antineoplastic agents	2.1	2.2	-0.01	2.1	2.1	0
Antipsoriatics	1.3	1.3	0	1.4	1.3	0.01
Antithrombotic agents	18.6	16.4	0.06	16.6	15.8	0.02
Beta blocking agents	14.6	16	-0.04	15.1	14.4	0.02

Table S6.79. *Continued.* Selected baseline characteristics for Italy IQVIA, for the short-term risk of self-harm and suicide

**Characteristic**	**Before propensity score matching**			**After propensity score matching**		
	**Targets, %**	**Comparators, %**	**Standardized difference**	**Targets, %**	**Comparators, %**	**Standardized difference**
**Medication use**						
Calcium channel blockers	9.4	10.6	-0.04	10	9.1	0.03
Diuretics	14.1	16.3	-0.06	14.6	14.1	0.01
Drugs for acid-related disorders	26.1	24.3	0.04	25.8	26.1	-0.01
Drugs for obstructive airway diseases	19.6	16	0.1	19	21.2	-0.06
Drugs used in diabetes	6.7	7.7	-0.04	6.8	6.8	0
Immunosuppressants	0.9	0.8	0.01	1	0.8	0.02
Lipid modifying agents	14.2	16.8	-0.07	14.9	14.4	0.01
Opioids	7.8	6.8	0.04	8.1	8.7	-0.02
Psycholeptics	12.4	11.5	0.03	13	13.4	-0.01
Psychostimulants, agents used for ADHD and nootropics	1	0.8	0.03	0.9	1.3	-0.04

Table S6.80. Selected baseline characteristics for Italy IQVIA, for the short-term risk of sleep disorders

Characteristic	Before propensity score matching			After propensity score matching		
	Targets, %	Comparators, %	Standardized difference	Targets, %	Comparators, %	Standardized difference
**Age group (years)**						
10-14	0.5	0.5	-0.01	0.4	0.7	-0.04
15-19	2.8	3.8	-0.06	3	3.4	-0.02
20-24	4.8	4.7	0	4.7	5	-0.02
25-29	5.6	4.9	0.03	5	5.5	-0.02
30-34	5.9	5.1	0.04	5.8	6.2	-0.02
35-39	6.5	5.6	0.04	6.1	6.6	-0.02
40-44	8	6.7	0.05	8.2	8.5	-0.01
45-49	10.4	8.2	0.07	10.3	11	-0.02
50-54	10.7	9.2	0.05	10.9	10.9	0
55-59	11.7	9.6	0.07	11.8	10	0.06
60-64	8.6	8.9	-0.01	8.8	8.5	0.01
65-69	6.2	8.1	-0.07	6.4	6.3	0
70-74	5.8	8.1	-0.09	6.1	5.5	0.02
75-79	4	6	-0.1	4.2	4.1	0
80-84	4	5.5	-0.07	4.3	4	0.01
85-89	2.7	3.3	-0.04	2.5	2.3	0.01
90-94	1.4	1.5	-0.01	1	1.2	-0.01
95-99	0.4	0.2	0.03	0.3	0.2	0.03
**Sex**						
Female	51.9	54.5	-0.05	52.2	52.6	-0.01

Table S6.80. *Continued.* Selected baseline characteristics for Italy IQVIA, for the short-term risk of sleep disorders

Characteristic	Before propensity score matching			After propensity score matching		
	Targets, %	Comparators, %	Standardized difference	Targets, %	Comparators, %	Standardized difference
**Medical history (general)**						
Acute respiratory disease	24	13.3	0.28	24.9	30.4	-0.12
Chronic liver disease	1.9	1.8	0.01	1.9	2.1	-0.01
Chronic obstructive lung disease	1.8	1.8	0.01	1.7	1.6	0.01
Crohn’s disease	0.3	0.2	0.01	0.3	0.3	0
Dementia	0.8	0.7	0.02	0.7	0.6	0
Depressive disorder	6.6	6.1	0.02	6.4	6.9	-0.02
Diabetes mellitus	7.7	8.4	-0.03	7.5	7.6	0
Gastroesophageal reflux disease	9	7.5	0.05	8.7	9.1	-0.01
Gastrointestinal hemorrhage	0.3	0.3	0	0.3	0.3	0
Human immunodeficiency virus infection	0.1	0.1	0.02	0.1	0.1	0.01
Hyperlipidemia	10.8	11.3	-0.02	11.4	10.6	0.03
Hypertensive disorder	25.4	27.2	-0.04	25.2	24.2	0.03
Lesion of liver	0.3	0.3	0	0.2	0.3	-0.02
Obesity	0.5	0.3	0.03	0.3	0.3	0
Osteoarthritis	7.7	8.2	-0.02	7.6	8	-0.01
Pneumonia	9.7	0.9	0.4	4.3	3.7	0.03
Psoriasis	1.7	1.4	0.02	1.5	1.6	-0.01
Renal impairment	2.1	1.9	0.02	2.3	2.2	0
Rheumatoid arthritis	0.6	0.7	-0.01	0.6	0.5	0.01
Schizophrenia	0.1	0.2	-0.02	0.2	0.2	-0.01
Ulcerative colitis	0.4	0.3	0.02	0.5	0.4	0.02
Urinary tract infectious disease	2.8	2.1	0.04	2.6	3	-0.03
Viral hepatitis C	0.4	0.4	0	0.4	0.5	0

Table S6.80. *Continued.* Selected baseline characteristics for Italy IQVIA, for the short-term risk of sleep disorders

**Characteristic**	**Before propensity score matching**			**After propensity score matching**		
	**Targets, %**	**Comparators, %**	**Standardized difference**	**Targets, %**	**Comparators, %**	**Standardized difference**
**Medical history (cardiovascular disease)**						
Atrial fibrillation	3	3	0	2.8	2.7	0.01
Cerebrovascular disease	3.8	4	-0.01	3.7	3.5	0.01
Coronary arteriosclerosis	0.4	0.3	0.02	0.3	0.4	0
Heart disease	12.3	12.2	0	12	12.1	-0.01
Heart failure	1.2	1.2	0	1.1	1.2	0
Peripheral vascular disease	0.5	0.6	-0.01	0.5	0.5	0
Pulmonary embolism	0.3	0.2	0.02	0.3	0.3	0
Venous thrombosis	0.5	0.4	0.01	0.6	0.7	-0.02
**Medical history (neoplasms)**						
Malignant lymphoma	0.4	0.3	0.01	0.3	0.4	-0.02
Malignant neoplasm of anorectum	0.1	0.2	0	0.1	0.1	0
Malignant neoplastic disease	6.2	6.1	0	5.8	5.7	0.01
Malignant tumor of breast	1.4	1.5	-0.01	1.3	1.1	0.02
Malignant tumor of colon	0.4	0.5	-0.01	0.4	0.3	0.01
Malignant tumor of urinary bladder	0.4	0.4	0	0.4	0.3	0
Primary malignant neoplasm of prostate	0.8	0.7	0.01	0.8	0.6	0.03
**Medication use**						
Agents acting on the renin-angiotensin system	20.6	23.6	-0.07	20.7	20.1	0.02
Antibacterials for systemic use	48.3	32	0.34	41.4	44.4	-0.06
Antidepressants	10.7	9.7	0.03	10.3	10.5	-0.01
Antiepileptics	4.6	4.4	0.01	4.7	4.8	-0.01
Antiinflammatory and antirheumatic products	29.3	29.1	0.01	28.7	31.2	-0.05
Antineoplastic agents	2.1	2.2	-0.01	2	2	0
Antipsoriatics	1.3	1.3	0	1.3	1.3	0.01
Antithrombotic agents	18.6	16.4	0.06	16	15.2	0.02

Table S6.80. *Continued.* Selected baseline characteristics for Italy IQVIA, for the short-term risk of sleep disorders

Characteristic	Before propensity score matching			After propensity score matching		
	**Targets, %**	**Comparators, %**	**Standardized difference**	**Targets, %**	**Comparators, %**	**Standardized difference**
**Medication use**						
Beta blocking agents	14.6	16.1	-0.04	14.5	13.9	0.02
Calcium channel blockers	9.4	10.5	-0.04	9.6	8.6	0.04
Diuretics	14.1	16.3	-0.06	14.1	13.4	0.02
Drugs for acid-related disorders	26.1	24.1	0.05	24.9	25	0
Drugs for obstructive airway diseases	19.6	16	0.09	18.7	20.8	-0.05
Drugs used in diabetes	6.7	7.7	-0.04	6.6	6.5	0
Immunosuppressants	0.9	0.8	0.01	0.9	0.8	0.02
Lipid modifying agents	14.2	16.8	-0.07	14.7	14	0.02
Opioids	7.8	6.8	0.04	7.6	8.2	-0.02
Psycholeptics	12.4	11.4	0.03	10.7	11.2	-0.01
Psychostimulants, agents used for ADHD and nootropics	1	0.8	0.02	0.8	1.2	-0.05

Table S6.81. Selected baseline characteristics for Italy IQVIA, for the short-term risk of dementia

Characteristic	Before propensity score matching			After propensity score matching		
	Targets, %	Comparators, %	Standardized difference	Targets, %	Comparators, %	Standardized difference
**Age group (years)**						
10-14	0.5	0.5	-0.01	0.4	0.7	-0.04
15-19	2.8	3.8	-0.06	3	3.3	-0.02
20-24	4.8	4.7	0	4.6	4.9	-0.01
25-29	5.6	4.9	0.03	4.9	5.4	-0.02
30-34	5.9	5.1	0.04	5.7	6	-0.01
35-39	6.5	5.6	0.04	6	6.5	-0.02
40-44	8	6.7	0.05	8.1	8.4	-0.01
45-49	10.4	8.2	0.07	10.4	11	-0.02
50-54	10.7	9.2	0.05	10.9	11	0
55-59	11.7	9.6	0.07	11.9	10	0.06
60-64	8.6	8.8	-0.01	8.9	8.6	0.01
70-74	5.8	8.1	-0.09	6.2	5.7	0.02
75-79	4	6	-0.1	4.5	4.3	0.01
80-84	4	5.4	-0.07	4.3	4.1	0.01
85-89	2.7	3.3	-0.04	2.3	2.3	0.01
90-94	1.4	1.5	-0.01	1	1.2	-0.02
95-99	0.4	0.2	0.03	0.3	0.2	0.02
**Sex**						
Female	51.9	54.5	-0.05	52.2	52.6	-0.01

Table S6.81. *Continued.* Selected baseline characteristics for Italy IQVIA, for the short-term risk of dementia

Characteristic	Before propensity score matching			After propensity score matching		
	**Targets, %**	**Comparators, %**	**Standardized difference**	**Targets, %**	**Comparators, %**	**Standardized difference**
**Medical history (general)**						
Acute respiratory disease	24	13.2	0.28	25.4	30.8	-0.12
Chronic liver disease	1.9	1.8	0.01	2	2.2	-0.01
Chronic obstructive lung disease	1.8	1.7	0.01	1.9	1.7	0.01
Crohn’s disease	0.3	0.2	0.01	0.3	0.3	0
Depressive disorder	6.6	6	0.03	6.8	7.3	-0.02
Diabetes mellitus	7.7	8.4	-0.03	7.7	7.8	0
Gastroesophageal reflux disease	9	7.6	0.05	9	9.6	-0.02
Gastrointestinal hemorrhage	0.3	0.3	0	0.3	0.4	-0.01
Human immunodeficiency virus infection	0.1	0.1	0.02	0.1	0.1	0.01
Hyperlipidemia	10.8	11.3	-0.02	11.4	10.9	0.01
Hypertensive disorder	25.4	27.1	-0.04	25.8	24.8	0.02
Lesion of liver	0.3	0.3	-0.01	0.3	0.4	-0.02
Obesity	0.5	0.3	0.03	0.4	0.4	-0.01
Osteoarthritis	7.7	8.2	-0.02	8	8.5	-0.02
Pneumonia	9.7	0.9	0.4	4.4	3.8	0.03
Psoriasis	1.7	1.4	0.03	1.6	1.6	0
Renal impairment	2.1	1.9	0.02	2.3	2.4	0
Rheumatoid arthritis	0.6	0.7	-0.01	0.6	0.5	0.01
Schizophrenia	0.1	0.2	-0.02	0.2	0.2	-0.01
Ulcerative colitis	0.4	0.3	0.02	0.5	0.4	0.02
Urinary tract infectious disease	2.8	2.1	0.04	2.8	3.2	-0.03
Viral hepatitis C	0.4	0.4	0.01	0.5	0.4	0

Table S6.81. *Continued.* Selected baseline characteristics for Italy IQVIA, for the short-term risk of dementia

Characteristic	Before propensity score matching			After propensity score matching		
	Targets, %	Comparators, %	Standardized difference	Targets, %	Comparators, %	Standardized difference
**Medical history (cardiovascular disease)**						
Cerebrovascular disease	3.8	4	-0.01	3.6	3.6	0
Coronary arteriosclerosis	0.4	0.3	0.02	0.4	0.4	0
Heart disease	12.3	12.1	0	12.5	12.6	0
Heart failure	1.2	1.2	0	1.2	1.3	-0.01
Ischemic heart disease	3.8	3.8	0	3.8	3.9	0
Peripheral vascular disease	0.5	0.6	-0.01	0.5	0.5	0
Pulmonary embolism	0.3	0.2	0.02	0.3	0.3	0
Venous thrombosis	0.5	0.5	0.01	0.6	0.7	-0.02
**Medical history (neoplasms)**						
Malignant lymphoma	0.4	0.3	0.01	0.3	0.4	-0.01
Malignant neoplasm of anorectum	0.1	0.2	0	0.2	0.2	0
Malignant neoplastic disease	6.2	6.1	0	6.1	6	0.01
Malignant tumor of breast	1.4	1.5	-0.01	1.3	1.1	0.02
Malignant tumor of colon	0.4	0.5	-0.01	0.4	0.4	0.01
Malignant tumor of urinary bladder	0.4	0.4	0	0.4	0.3	0.01
Primary malignant neoplasm of prostate	0.8	0.8	0	0.8	0.6	0.03
**Medication use**						
Agents acting on the renin-angiotensin system	20.6	23.6	-0.07	21.3	20.5	0.02
Antibacterials for systemic use	48.3	31.9	0.34	41.7	44.7	-0.06
Antidepressants	10.7	9.7	0.03	11	11	0
Antiepileptics	4.6	4.3	0.01	4.8	5	-0.01
Antiinflammatory and antirheumatic products	29.3	29	0.01	29.4	31.8	-0.05
Antineoplastic agents	2.1	2.2	-0.01	2.1	2.1	0
Antipsoriatics	1.3	1.2	0.01	1.4	1.3	0.01
Antithrombotic agents	18.6	16.4	0.06	16.3	15.4	0.02

Table S6.81. *Continued.* Selected baseline characteristics for Italy IQVIA, for the short-term risk of dementia

Characteristic	Before propensity score matching			After propensity score matching		
	**Targets, %**	**Comparators, %**	**Standardized difference**	**Targets, %**	**Comparators, %**	**Standardized difference**
**Medication use**						
Beta blocking agents	14.6	16	-0.04	15	14.3	0.02
Calcium channel blockers	9.4	10.5	-0.04	10.1	9	0.04
Diuretics	14.1	16.3	-0.06	14.4	13.8	0.02
Drugs for acid-related disorders	26.1	24.1	0.05	25.6	25.9	-0.01
Drugs for obstructive airway diseases	19.6	15.9	0.1	19	21.2	-0.06
Drugs used in diabetes	6.7	7.7	-0.04	6.8	6.7	0
Immunosuppressants	0.9	0.8	0.01	1	0.8	0.02
Lipid modifying agents	14.2	16.7	-0.07	14.8	14.3	0.01
Opioids	7.8	6.8	0.04	8.1	8.6	-0.02
Psycholeptics	12.4	11.4	0.03	12.7	13.1	-0.01
Psychostimulants, agents used for ADHD and nootropics	1	0.8	0.03	0.9	1.3	-0.04

Table S6.82. Selected baseline characteristics for Italy IQVIA, for the short-term risk of neurodevelopmental disorders

**Characteristic**	**Before propensity score matching**			**After propensity score matching**		
	**Targets, %**	**Comparators, %**	**Standardized difference**	**Targets, %**	**Comparators, %**	**Standardized difference**
**Age group (years)**						
10-14	0.5	0.5	-0.01	0.4	0.7	-0.04
15-19	2.8	3.8	-0.06	2.9	3.2	-0.02
20-24	4.8	4.7	0	4.5	4.8	-0.01
25-29	5.6	4.9	0.03	4.9	5.4	-0.02
30-34	5.9	5.1	0.04	5.7	5.9	-0.01
35-39	6.5	5.6	0.04	5.9	6.4	-0.02
40-44	8	6.7	0.05	8	8.3	-0.01
45-49	10.4	8.3	0.07	10.3	10.9	-0.02
50-54	10.7	9.1	0.05	10.8	10.9	0
55-59	11.7	9.5	0.07	11.8	10	0.06
60-64	8.6	8.9	-0.01	8.9	8.6	0.01
65-69	6.2	8	-0.07	6.5	6.5	0
70-74	5.8	8.1	-0.09	6.2	5.7	0.02
75-79	4	6.1	-0.1	4.5	4.3	0.01
80-84	4	5.5	-0.07	4.4	4.2	0.01
85-89	2.7	3.3	-0.04	2.5	2.5	0
90-94	1.4	1.5	-0.01	1.2	1.3	-0.01
95-99	0.4	0.2	0.03	0.3	0.2	0.02
**Sex**						
Female	51.9	54.5	-0.05	52.4	52.7	-0.01

Table S6.82. *Continued.* Selected baseline characteristics for Italy IQVIA, for the short-term risk of neurodevelopmental disorders

Characteristic	Before propensity score matching			After propensity score matching		
	Targets, %	Comparators, %	Standardized difference	Targets, %	Comparators, %	Standardized difference
**Medical history (general)**						
Acute respiratory disease	24	13.2	0.28	25.4	30.8	-0.12
Chronic liver disease	1.9	1.8	0	2.1	2.2	-0.01
Chronic obstructive lung disease	1.8	1.8	0.01	1.9	1.7	0.01
Crohn’s disease	0.3	0.2	0.01	0.3	0.3	-0.01
Dementia	0.8	0.7	0.01	0.7	0.7	0
Depressive disorder	6.6	6.1	0.02	6.9	7.4	-0.02
Diabetes mellitus	7.7	8.5	-0.03	7.8	7.9	0
Gastroesophageal reflux disease	9	7.6	0.05	9.1	9.7	-0.02
Gastrointestinal hemorrhage	0.3	0.2	0	0.3	0.4	-0.01
Human immunodeficiency virus infection	0.1	0.1	0.02	0.1	0.1	0.01
Hyperlipidemia	10.8	11.4	-0.02	11.5	11	0.02
Hypertensive disorder	25.4	27.1	-0.04	26	25.1	0.02
Lesion of liver	0.3	0.3	-0.01	0.3	0.4	-0.02
Obesity	0.5	0.3	0.03	0.4	0.4	-0.01
Osteoarthritis	7.7	8.1	-0.02	8	8.6	-0.02
Pneumonia	9.7	0.9	0.4	4.4	3.8	0.03
Psoriasis	1.7	1.4	0.02	1.6	1.6	0
Renal impairment	2.1	1.9	0.02	2.4	2.4	0
Rheumatoid arthritis	0.6	0.6	-0.01	0.6	0.5	0.01
Schizophrenia	0.1	0.2	-0.02	0.2	0.2	-0.01
Ulcerative colitis	0.4	0.3	0.02	0.5	0.4	0.02
Urinary tract infectious disease	2.8	2.1	0.04	2.8	3.3	-0.03
Viral hepatitis C	0.4	0.4	0	0.5	0.4	0

Table S6.82. *Continued.* Selected baseline characteristics for Italy IQVIA, for the short-term risk of neurodevelopmental disorders

Characteristic	Before propensity score matching			After propensity score matching		
	Targets, %	Comparators, %	Standardized difference	Targets, %	Comparators, %	Standardized difference
**Medical history (cardiovascular disease)**						
Atrial fibrillation	3	2.9	0	3.1	2.9	0.01
Cerebrovascular disease	3.8	4	-0.01	3.7	3.7	0
Heart disease	12.3	12.2	0	12.6	12.8	-0.01
Heart failure	1.2	1.2	0.01	1.3	1.3	-0.01
Peripheral vascular disease	0.5	0.6	-0.01	0.5	0.5	0
Venous thrombosis	0.5	0.5	0.01	0.6	0.7	-0.02
**Medical history (neoplasms)**						
Malignant lymphoma	0.4	0.3	0.01	0.3	0.4	-0.01
Malignant neoplasm of anorectum	0.1	0.2	0	0.2	0.2	0
Malignant neoplastic disease	6.2	6.1	0	6.1	6	0.01
Malignant tumor of breast	1.4	1.5	-0.01	1.4	1.1	0.02
Malignant tumor of colon	0.4	0.5	-0.01	0.4	0.4	0.01
Malignant tumor of urinary bladder	0.4	0.4	0	0.4	0.3	0.01
Primary malignant neoplasm of prostate	0.8	0.8	0	0.8	0.6	0.03
**Medication use**						
Agents acting on the renin-angiotensin system	20.6	23.5	-0.07	21.4	20.7	0.02
Antibacterials for systemic use	48.3	32	0.34	41.8	44.9	-0.06
Antidepressants	10.7	9.8	0.03	11.1	11.3	0
Antiepileptics	4.6	4.4	0.01	4.9	5	0
Antiinflammatory and antirheumatic products	29.3	28.9	0.01	29.4	31.9	-0.06
Antineoplastic agents	2.1	2.2	-0.01	2.1	2.1	-0.01
Antipsoriatics	1.3	1.2	0.01	1.4	1.3	0.01
Antithrombotic agents	18.6	16.4	0.06	16.5	15.8	0.02
Beta blocking agents	14.6	16.1	-0.04	15.1	14.5	0.02
Calcium channel blockers	9.4	10.5	-0.04	10	9.1	0.03

Table S6.82. *Continued.* Selected baseline characteristics for Italy IQVIA, for the short-term risk of neurodevelopmental disorders

**Characteristic**	**Before propensity score matching**			**After propensity score matching**		
	**Targets, %**	**Comparators, %**	**Standardized difference**	**Targets, %**	**Comparators, %**	**Standardized difference**
**Medication use**						
Diuretics	14.1	16.3	-0.06	14.6	14.1	0.01
Drugs for acid-related disorders	26.1	24.3	0.04	25.8	26.1	-0.01
Drugs for obstructive airway diseases	19.6	15.9	0.1	19	21.2	-0.06
Drugs used in diabetes	6.7	7.7	-0.04	6.8	6.8	0
Immunosuppressants	0.9	0.8	0.01	1	0.8	0.02
Lipid modifying agents	14.2	16.8	-0.07	14.9	14.4	0.01
Opioids	7.8	6.9	0.04	8.1	8.7	-0.02
Psycholeptics	12.4	11.5	0.03	12.9	13.3	-0.01
Psychostimulants, agents used for ADHD and nootropics	1	0.8	0.03	0.9	1.3	-0.04

Table S6.83. Selected baseline characteristics for Italy IQVIA, for the short-term risk of any of psychiatric and neuropsychiatric disorders

Characteristic	Before propensity score matching			After propensity score matching		
	Targets, %	Comparators, %	Standardized difference	Targets, %	Comparators, %	Standardized difference
**Age group (years)**						
10-14	0.5	0.5	-0.01	0.5	0.8	-0.04
15-19	2.8	3.9	-0.06	3.3	3.7	-0.02
20-24	4.8	4.8	0	4.9	5.5	-0.03
25-29	5.6	4.8	0.03	5.4	5.9	-0.02
30-34	5.9	5.1	0.04	6.2	6.4	-0.01
35-39	6.5	5.6	0.04	6.4	6.9	-0.02
40-44	8	6.7	0.05	8.4	8.8	-0.01
45-49	10.4	8.2	0.08	10.5	11.1	-0.02
50-54	10.7	9.2	0.05	11.1	11	0
55-59	11.7	9.6	0.07	11.7	10.1	0.05
60-64	8.6	8.8	-0.01	8.9	8.3	0.02
65-69	6.2	8.1	-0.07	6.3	6.3	0
70-74	5.8	8.1	-0.09	5.7	5.1	0.03
75-79	4	6	-0.1	4	3.6	0.02
80-84	4	5.5	-0.07	3.8	3.4	0.02
85-89	2.7	3.3	-0.04	1.8	1.9	-0.01
90-94	1.4	1.5	-0.01	0.8	0.9	-0.01
95-99	0.4	0.2	0.03	0.2	0.2	0.02
**Sex**						
Female	51.9	54.4	-0.05	50.8	51.1	-0.01

Table S6.83. *Continued.* Selected baseline characteristics for Italy IQVIA, for the short-term risk of any of psychiatric and neuropsychiatric disorders

Characteristic	Before propensity score matching			After propensity score matching		
	Targets, %	Comparators, %	Standardized difference	Targets, %	Comparators, %	Standardized difference
**Medical history (general)**						
Acute respiratory disease	24	13.2	0.28	24.8	29.9	-0.11
Chronic liver disease	1.9	1.8	0.01	1.8	1.9	-0.01
Chronic obstructive lung disease	1.8	1.8	0.01	1.6	1.4	0.01
Crohn’s disease	0.3	0.2	0.01	0.3	0.3	-0.01
Diabetes mellitus	7.7	8.4	-0.03	6.9	7.1	-0.01
Gastroesophageal reflux disease	9	7.6	0.05	7.8	8.2	-0.01
Gastrointestinal hemorrhage	0.3	0.3	0	0.3	0.3	0
Human immunodeficiency virus infection	0.1	0.1	0.02	0.1	0.1	0.01
Hyperlipidemia	10.8	11.4	-0.02	10.3	9.7	0.02
Hypertensive disorder	25.4	27.2	-0.04	23.8	22.5	0.03
Lesion of liver	0.3	0.3	-0.01	0.2	0.3	-0.02
Obesity	0.5	0.3	0.03	0.3	0.3	0.01
Osteoarthritis	7.7	8.2	-0.02	6.9	7	0
Pneumonia	9.7	0.9	0.4	4.2	3.4	0.05
Psoriasis	1.7	1.4	0.03	1.4	1.4	0
Renal impairment	2.1	1.9	0.02	2	1.9	0.01
Rheumatoid arthritis	0.6	0.6	-0.01	0.5	0.5	0
Ulcerative colitis	0.4	0.3	0.02	0.4	0.4	0.01
Urinary tract infectious disease	2.8	2.1	0.04	2.2	2.7	-0.03

Table S6.83. *Continued.* Selected baseline characteristics for Italy IQVIA, for the short-term risk of any of psychiatric and neuropsychiatric disorders

Characteristic	Before propensity score matching			After propensity score matching		
	Targets, %	Comparators, %	Standardized difference	Targets, %	Comparators, %	Standardized difference
**Medical history (cardiovascular disease)**						
Atrial fibrillation	3	2.9	0	2.5	2.4	0
Cerebrovascular disease	3.8	3.9	-0.01	3.1	2.8	0.02
Coronary arteriosclerosis	0.4	0.3	0.02	0.4	0.3	0
Heart disease	12.3	12.1	0	10.9	10.9	0
Heart failure	1.2	1.2	0	1	1	0.01
Ischemic heart disease	3.8	3.8	0	3.4	3.5	-0.01
Pulmonary embolism	0.3	0.2	0.02	0.2	0.2	0.01
Venous thrombosis	0.5	0.5	0.01	0.5	0.6	-0.02
**Medical history (neoplasms)**						
Malignant lymphoma	0.4	0.3	0.01	0.3	0.4	-0.02
Malignant neoplastic disease	6.2	6.1	0	5.5	5.4	0.01
Malignant tumor of breast	1.4	1.5	-0.01	1.2	1	0.02
Malignant tumor of colon	0.4	0.5	-0.01	0.4	0.3	0.01
Primary malignant neoplasm of prostate	0.8	0.8	0	0.7	0.6	0.02
**Medication use**						
Agents acting on the renin-angiotensin system	20.6	23.6	-0.07	19.8	18.8	0.03
Antibacterials for systemic use	48.3	31.9	0.34	40.2	43.4	-0.06
Antidepressants	10.7	9.8	0.03	4.3	4.4	0
Antiepileptics	4.6	4.5	0.01	3.3	3.3	0
Antiinflammatory and antirheumatic products	29.3	29	0.01	27.4	29.8	-0.05
Antineoplastic agents	2.1	2.2	-0.01	1.8	1.8	0
Antipsoriatics	1.3	1.3	0.01	1.3	1.2	0.01
Antithrombotic agents	18.6	16.5	0.06	14.1	13.7	0.01

Table S6.83. *Continued.* Selected baseline characteristics for Italy IQVIA, for the short-term risk of any of psychiatric and neuropsychiatric disorders

**Characteristic**	**Before propensity score matching**			**After propensity score matching**		
	**Targets, %**	**Comparators, %**	**Standardized difference**	**Targets, %**	**Comparators, %**	**Standardized difference**
**Medication use**						
Beta blocking agents	14.6	15.9	-0.04	13.2	12.7	0.01
Calcium channel blockers	9.4	10.5	-0.04	8.9	8.1	0.03
Diuretics	14.1	16.3	-0.06	13.1	12.3	0.02
Drugs for acid-related disorders	26.1	24.2	0.04	22.4	23	-0.01
Drugs for obstructive airway diseases	19.6	15.9	0.1	18.1	20.1	-0.05
Immunosuppressants	0.9	0.8	0	0.9	0.7	0.02
Lipid modifying agents	14.2	16.8	-0.07	13.7	13	0.02
Opioids	7.8	6.9	0.03	6.7	7.3	-0.02
Psychostimulants, agents used for ADHD and nootropics	1	0.8	0.02	0.6	1.1	-0.05

Table S6.84. Selected baseline characteristics for Italy IQVIA, for the medium-term risk of depression

Characteristic	Before propensity score matching			After propensity score matching		
	Targets, %	Comparators, %	Standardized difference	Targets, %	Comparators, %	Standardized difference
**Age group (years)**						
10-14	0.5	0.5	-0.01	0.5	0.8	-0.03
15-19	2.8	3.9	-0.06	3.4	3.9	-0.03
20-24	4.8	4.7	0	4.8	5.1	-0.01
25-29	5.6	4.8	0.04	5.2	5.4	0
30-34	5.9	5.1	0.04	5.7	6.1	-0.01
35-39	6.5	5.5	0.04	6.2	6.2	0
40-44	8	6.7	0.05	7.8	8.2	-0.01
45-49	10.4	8.2	0.07	10.2	10.8	-0.02
50-54	10.7	9.3	0.05	10.7	11.3	-0.02
55-59	11.7	9.6	0.07	11.8	10.3	0.05
60-64	8.6	8.7	-0.01	9.5	9	0.02
65-69	6.2	8	-0.07	6.7	7	-0.01
70-74	5.8	8.3	-0.1	6.4	5.4	0.04
75-79	4	6.1	-0.1	4.1	3.8	0.02
80-84	4	5.5	-0.07	3.8	3.7	0
85-89	2.7	3.3	-0.04	2.1	2.2	0
90-94	1.4	1.5	-0.01	0.8	0.9	-0.01
95-99	0.4	0.2	0.03	0.2	0.1	0.01
**Sex**						
Female	51.9	54.3	-0.05	52.2	52	0

Table S6.84. *Continued.* Selected baseline characteristics for Italy IQVIA, for the medium-term risk of depression

Characteristic	Before propensity score matching			After propensity score matching		
	Targets, %	Comparators, %	Standardized difference	Targets, %	Comparators, %	Standardized difference
**Medical history (general)**						
Acute respiratory disease	24	13.4	0.27	25.1	30.1	-0.11
Chronic liver disease	1.9	1.9	0	1.9	2.2	-0.02
Chronic obstructive lung disease	1.8	1.8	0	1.9	1.5	0.02
Crohn's disease	0.3	0.2	0.01	0.3	0.3	-0.01
Diabetes mellitus	7.7	8.5	-0.03	7.8	7.6	0
Gastroesophageal reflux disease	9	7.6	0.05	8.6	8.9	-0.01
Gastrointestinal hemorrhage	0.3	0.2	0.01	0.3	0.3	0
Hyperlipidemia	10.8	11.4	-0.02	10.8	10.7	0
Hypertensive disorder	25.4	27.2	-0.04	25.3	25.1	0.01
Lesion of liver	0.3	0.3	-0.01	0.2	0.3	-0.02
Obesity	0.5	0.3	0.03	0.4	0.4	0
Osteoarthritis	7.7	8.2	-0.02	7.2	7.9	-0.03
Pneumonia	9.7	0.9	0.4	3.9	3.4	0.03
Psoriasis	1.7	1.4	0.02	1.7	1.6	0.01
Renal impairment	2.1	1.9	0.02	2.1	1.9	0.01
Rheumatoid arthritis	0.6	0.6	0	0.5	0.6	-0.01
Schizophrenia	0.1	0.2	-0.02	0.2	0.2	-0.01
Ulcerative colitis	0.4	0.3	0.02	0.4	0.3	0.01
Urinary tract infectious disease	2.8	2.2	0.04	2.4	3	-0.03
Viral hepatitis C	0.4	0.4	0	0.4	0.4	0

Table S6.84. *Continued.* Selected baseline characteristics for Italy IQVIA, for the medium-term risk of depression

Characteristic	Before propensity score matching			After propensity score matching		
	Targets, %	Comparators, %	Standardized difference	Targets, %	Comparators, %	Standardized difference
**Medical history (cardiovascular disease)**						
Atrial fibrillation	3	3	0	2.8	2.8	0
Cerebrovascular disease	3.8	3.9	-0.01	3	3	0
Coronary arteriosclerosis	0.4	0.3	0.01	0.3	0.3	0
Heart failure	1.2	1.2	0	1	0.9	0
Peripheral vascular disease	0.5	0.6	-0.01	0.4	0.5	-0.02
Pulmonary embolism	0.3	0.2	0.02	0.3	0.3	0
Venous thrombosis	0.5	0.5	0.01	0.6	0.7	-0.02
**Medical history (neoplasms)**						
Malignant lymphoma	0.4	0.3	0.01	0.2	0.4	-0.03
Malignant neoplasm of anorectum	0.1	0.2	0	0.2	0.2	-0.01
Malignant neoplastic disease	6.2	6.2	0	5.7	5.9	-0.01
Malignant tumor of breast	1.4	1.5	-0.01	1.3	1.2	0.01
Malignant tumor of urinary bladder	0.4	0.4	0	0.3	0.4	0
Primary malignant neoplasm of prostate	0.8	0.8	0	0.8	0.7	0.01
**Medication use**						
Agents acting on the renin-angiotensin system	20.6	23.6	-0.07	21.2	20.8	0.01
Antibacterials for systemic use	48.3	32.2	0.33	42	44.4	-0.05
Antidepressants	10.7	9.8	0.03	5.9	5.8	0
Antiepileptics	4.6	4.4	0.01	4	4	0
Antiinflammatory and antirheumatic products	29.3	29	0.01	30.3	31.2	-0.02
Antineoplastic agents	2.1	2.2	-0.01	1.9	2	-0.01
Antipsoriatics	1.3	1.3	0	1.5	1.4	0.01
Antithrombotic agents	18.6	16.5	0.06	14.9	14.7	0.01
Beta blocking agents	14.6	16.1	-0.04	13.9	14.1	0
Calcium channel blockers	9.4	10.6	-0.04	9.5	9	0.02

Table S6.84. *Continued.* Selected baseline characteristics for Italy IQVIA, for the medium-term risk of depression

**Characteristic**	**Before propensity score matching**			**After propensity score matching**		
	**Targets, %**	**Comparators, %**	**Standardized difference**	**Targets, %**	**Comparators, %**	**Standardized difference**
**Medication use**						
Diuretics	14.1	16.4	-0.06	13.9	13.4	0.02
Drugs for acid-related disorders	26.1	24.3	0.04	24	24.3	-0.01
Drugs for obstructive airway diseases	19.6	15.9	0.1	18.8	21.6	-0.07
Drugs used in diabetes	6.7	7.8	-0.04	6.7	6.8	0
Immunosuppressants	0.9	0.8	0.01	1	0.8	0.02
Lipid modifying agents	14.2	16.8	-0.07	14.3	13.9	0.01
Opioids	7.8	7	0.03	7.2	7.6	-0.02
Psycholeptics	12.4	11.5	0.03	9.9	10.2	-0.01
Psychostimulants, agents used for ADHD and nootropics	1	0.8	0.03	0.9	1.2	-0.03

Table S6.85. Selected baseline characteristics for Italy IQVIA, for the medium-term risk of anxiety disorders

**Characteristic**	**Before propensity score matching**			**After propensity score matching**		
	**Targets, %**	**Comparators, %**	**Standardized difference**	**Targets, %**	**Comparators, %**	**Standardized difference**
**Age group (years)**						
10-14	0.5	0.5	-0.01	0.4	0.7	-0.03
15-19	2.8	3.8	-0.05	3.2	3.6	-0.03
20-24	4.8	4.6	0.01	4.4	4.7	-0.01
25-29	5.6	4.8	0.03	4.8	5.1	-0.01
30-34	5.9	5.1	0.04	5.4	5.8	-0.02
35-39	6.5	5.5	0.04	6	5.9	0
40-44	8	6.7	0.05	7.7	8	-0.01
45-49	10.4	8.3	0.07	10	10.6	-0.02
50-54	10.7	9.2	0.05	10.6	11.2	-0.02
55-59	11.7	9.5	0.07	12	10.3	0.06
60-64	8.6	8.9	-0.01	9.4	9.3	0
65-69	6.2	8	-0.07	6.8	7.1	-0.01
70-74	5.8	8.3	-0.1	6.7	5.8	0.04
75-79	4	6	-0.09	4.5	4.3	0.01
80-84	4	5.6	-0.07	4.4	4.1	0.01
85-89	2.7	3.4	-0.04	2.5	2.4	0
90-94	1.4	1.5	0	1	1.1	-0.01
95-99	0.4	0.2	0.03	0.2	0.2	0
**Sex**						
Female	51.9	54.5	-0.05	53.9	53.5	0.01

Table S6.85. *Continued.* Selected baseline characteristics for Italy IQVIA, for the medium-term risk of anxiety disorders

Characteristic	Before propensity score matching			After propensity score matching		
	Targets, %	Comparators, %	Standardized difference	Targets, %	Comparators, %	Standardized difference
**Medical history (general)**						
Acute respiratory disease	24	13.2	0.28	25.1	30.4	-0.12
Chronic liver disease	1.9	1.8	0	1.9	2.3	-0.03
Chronic obstructive lung disease	1.8	1.7	0.01	2	1.7	0.02
Crohn’s disease	0.3	0.2	0.01	0.3	0.3	-0.01
Dementia	0.8	0.7	0.02	0.6	0.5	0.01
Depressive disorder	6.6	6.2	0.02	7.2	7.5	-0.01
Diabetes mellitus	7.7	8.5	-0.03	8.1	8.1	0
Gastroesophageal reflux disease	9	7.6	0.05	9.2	9.7	-0.01
Human immunodeficiency virus infection	0.1	0.1	0.02	0.1	0.1	0.02
Hyperlipidemia	10.8	11.4	-0.02	11.5	11.3	0.01
Hypertensive disorder	25.4	27.2	-0.04	26.2	26.5	-0.01
Lesion of liver	0.3	0.3	-0.01	0.2	0.3	-0.03
Obesity	0.5	0.3	0.03	0.4	0.4	-0.01
Osteoarthritis	7.7	8.2	-0.02	7.8	8.7	-0.03
Pneumonia	9.7	0.9	0.4	4.1	3.6	0.03
Psoriasis	1.7	1.3	0.03	1.8	1.8	0
Renal impairment	2.1	1.9	0.02	2.2	2.1	0
Rheumatoid arthritis	0.6	0.6	-0.01	0.6	0.6	0
Schizophrenia	0.1	0.2	-0.02	0.2	0.2	-0.01
Ulcerative colitis	0.4	0.3	0.02	0.4	0.4	0.01
Urinary tract infectious disease	2.8	2.1	0.04	2.7	3.1	-0.02
Viral hepatitis C	0.4	0.4	0	0.5	0.4	0.01

Table S6.85. *Continued.* Selected baseline characteristics for Italy IQVIA, for the medium-term risk of anxiety disorders

Characteristic	Before propensity score matching			After propensity score matching		
	Targets, %	Comparators, %	Standardized difference	Targets, %	Comparators, %	Standardized difference
**Medical history (cardiovascular disease)**						
Atrial fibrillation	3	3	0	3	3	0
Cerebrovascular disease	3.8	4	-0.01	3.6	3.5	0
Coronary arteriosclerosis	0.4	0.3	0.01	0.4	0.3	0.01
Heart disease	12.3	12.2	0	12.7	12.8	0
Heart failure	1.2	1.2	0.01	1	1.1	0
Ischemic heart disease	3.8	3.8	0	3.9	4	-0.01
Peripheral vascular disease	0.5	0.6	-0.01	0.5	0.6	-0.02
Pulmonary embolism	0.3	0.2	0.02	0.3	0.3	0
Venous thrombosis	0.5	0.5	0.01	0.6	0.8	-0.03
**Medical history (neoplasms)**						
Malignant lymphoma	0.4	0.4	0	0.3	0.5	-0.03
Malignant neoplasm of anorectum	0.1	0.2	0	0.2	0.2	-0.01
Malignant neoplastic disease	6.2	6.2	0	6	6.2	-0.01
Malignant tumor of breast	1.4	1.5	-0.01	1.4	1.2	0.02
Malignant tumor of colon	0.4	0.5	-0.01	0.5	0.4	0
Malignant tumor of urinary bladder	0.4	0.4	0	0.4	0.4	0
Primary malignant neoplasm of prostate	0.8	0.7	0	0.7	0.7	0.01
**Medication use**						
Agents acting on the renin-angiotensin system	20.6	23.6	-0.07	22	21.8	0
Antibacterials for systemic use	48.3	32	0.34	42.7	45.1	-0.05
Antidepressants	10.7	9.8	0.03	10.7	10.6	0
Antiepileptics	4.6	4.5	0.01	5.1	5	0
Antiinflammatory and antirheumatic products	29.3	29	0.01	31.6	32.4	-0.02
Antineoplastic agents	2.1	2.2	-0.01	2.2	2.2	0
Antipsoriatics	1.3	1.3	0.01	1.5	1.4	0

Table S6.85. *Continued.* Selected baseline characteristics for Italy IQVIA, for the medium-term risk of anxiety disorders

Characteristic	Before propensity score matching			After propensity score matching		
	Targets, %	Comparators, %	Standardized difference	Targets, %	Comparators, %	Standardized difference
**Medication use**						
Antithrombotic agents	18.6	16.5	0.06	16.7	15.8	0.02
Beta blocking agents	14.6	16.1	-0.04	15.1	15	0
Calcium channel blockers	9.4	10.5	-0.04	10.2	9.5	0.02
Diuretics	14.1	16.4	-0.06	14.8	14.3	0.01
Drugs for acid-related disorders	26.1	24.2	0.04	26.2	25.9	0.01
Drugs for obstructive airway diseases	19.6	15.9	0.1	19.9	22.1	-0.05
Drugs used in diabetes	6.7	7.7	-0.04	7	7.1	0
Immunosuppressants	0.9	0.8	0.01	1	0.9	0.02
Lipid modifying agents	14.2	16.9	-0.07	15.3	14.9	0.01
Opioids	7.8	6.9	0.03	7.9	8.5	-0.02
Psychostimulants, agents used for ADHD and nootropics	1	0.8	0.02	1	1.3	-0.04

Table S6.86. Selected baseline characteristics for Italy IQVIA, for the medium-term risk of alcohol misuse or dependence

Characteristic	Before propensity score matching			After propensity score matching		
	Targets, %	Comparators, %	Standardized difference	Targets, %	Comparators, %	Standardized difference
**Age group (years)**						
10-14	0.5	0.5	-0.01	0.4	0.7	-0.04
15-19	2.8	3.9	-0.06	3.2	3.6	-0.02
20-24	4.8	4.7	0.01	4.4	4.7	-0.01
25-29	5.6	4.8	0.04	4.8	5.1	-0.01
30-34	5.9	5.1	0.04	5.4	5.8	-0.02
35-39	6.5	5.6	0.04	6	5.9	0
40-44	8	6.7	0.05	7.7	8	-0.01
45-49	10.4	8.3	0.07	10.1	10.6	-0.02
50-54	10.7	9.3	0.05	10.7	11.3	-0.02
55-59	11.7	9.6	0.07	12.1	10.3	0.06
60-64	8.6	8.8	-0.01	9.4	9.3	0
65-69	6.2	8.1	-0.07	6.8	7	-0.01
70-74	5.8	8.1	-0.09	6.6	5.8	0.04
75-79	4	6	-0.09	4.4	4.2	0.01
80-84	4	5.5	-0.07	4.3	4.1	0.01
85-89	2.7	3.3	-0.04	2.5	2.4	0.01
90-94	1.4	1.5	0	1	1.1	-0.01
95-99	0.4	0.2	0.03	0.2	0.1	0
**Sex**						
Female	51.9	54.7	-0.06	54.1	53.8	0

Table S6.86. *Continued.* Selected baseline characteristics for Italy IQVIA, for the medium-term risk of alcohol misuse or dependence

Characteristic	Before propensity score matching			After propensity score matching		
	Targets, %	Comparators, %	Standardized difference	Targets, %	Comparators, %	Standardized difference
**Medical history (general)**						
Acute respiratory disease	24	13.2	0.28	25.4	30.6	-0.12
Chronic liver disease	1.9	1.8	0	1.9	2.3	-0.02
Chronic obstructive lung disease	1.8	1.7	0.01	2	1.7	0.02
Crohn's disease	0.3	0.2	0.01	0.3	0.3	-0.01
Dementia	0.8	0.7	0.02	0.6	0.5	0.01
Depressive disorder	6.6	6.2	0.02	7.3	7.5	-0.01
Diabetes mellitus	7.7	8.4	-0.03	8.1	8	0
Gastroesophageal reflux disease	9	7.6	0.05	9.3	9.8	-0.02
Gastrointestinal hemorrhage	0.3	0.2	0	0.3	0.3	-0.01
Human immunodeficiency virus infection	0.1	0.1	0.02	0.1	0.1	0.02
Hyperlipidemia	10.8	11.4	-0.02	11.5	11.4	0
Hypertensive disorder	25.4	27	-0.04	26.3	26.4	0
Lesion of liver	0.3	0.3	-0.01	0.2	0.3	-0.03
Obesity	0.5	0.3	0.03	0.4	0.4	-0.01
Osteoarthritis	7.7	8.1	-0.01	7.9	8.8	-0.03
Pneumonia	9.7	0.9	0.4	4.1	3.6	0.03
Psoriasis	1.7	1.4	0.02	1.8	1.8	0
Renal impairment	2.1	1.9	0.02	2.2	2.1	0
Rheumatoid arthritis	0.6	0.6	0	0.6	0.6	-0.01
Schizophrenia	0.1	0.2	-0.02	0.2	0.2	-0.01
Ulcerative colitis	0.4	0.3	0.02	0.4	0.4	0
Urinary tract infectious disease	2.8	2.1	0.04	2.8	3.2	-0.03
Viral hepatitis C	0.4	0.4	0.01	0.5	0.4	0.01

Table S6.86. *Continued.* Selected baseline characteristics for Italy IQVIA, for the medium-term risk of alcohol misuse or dependence

Characteristic	Before propensity score matching			After propensity score matching		
	Targets, %	Comparators, %	Standardized difference	Targets, %	Comparators, %	Standardized difference
**Medical history (cardiovascular disease)**						
Atrial fibrillation	3	2.9	0	3.1	3	0
Cerebrovascular disease	3.8	3.9	-0.01	3.6	3.5	0
Coronary arteriosclerosis	0.4	0.3	0.02	0.4	0.3	0
Heart disease	12.3	12.1	0	12.7	12.8	0
Heart failure	1.2	1.2	0	1	1.1	0
Ischemic heart disease	3.8	3.7	0	3.9	4	-0.01
Peripheral vascular disease	0.5	0.6	-0.01	0.4	0.6	-0.02
Pulmonary embolism	0.3	0.2	0.02	0.3	0.3	0
Venous thrombosis	0.5	0.5	0.01	0.6	0.8	-0.02
**Medical history (neoplasms)**						
Malignant lymphoma	0.4	0.3	0.01	0.3	0.5	-0.03
Malignant neoplasm of anorectum	0.1	0.2	0	0.1	0.2	-0.01
Malignant neoplastic disease	6.2	6.1	0	6	6.2	-0.01
Malignant tumor of breast	1.4	1.5	-0.01	1.4	1.2	0.02
Malignant tumor of colon	0.4	0.5	-0.01	0.4	0.4	0
Malignant tumor of urinary bladder	0.4	0.4	0	0.3	0.3	0
Primary malignant neoplasm of prostate	0.8	0.7	0.01	0.8	0.7	0.01
**Medication use**						
Agents acting on the renin-angiotensin system	20.6	23.5	-0.07	22	21.7	0.01
Antibacterials for systemic use	48.3	32	0.34	42.8	45.2	-0.05
Antidepressants	10.7	9.8	0.03	11.4	11.1	0.01
Antiepileptics	4.6	4.4	0.01	5.2	5	0.01
Antiinflammatory and antirheumatic products	29.3	28.8	0.01	31.7	32.4	-0.01
Antineoplastic agents	2.1	2.2	-0.01	2.1	2.2	0
Antipsoriatics	1.3	1.3	0.01	1.5	1.4	0

Table S6.86. *Continued.* Selected baseline characteristics for Italy IQVIA, for the medium-term risk of alcohol misuse or dependence

**Characteristic**	**Before propensity score matching**			**After propensity score matching**		
	**Targets, %**	**Comparators, %**	**Standardized difference**	**Targets, %**	**Comparators, %**	**Standardized difference**
**Medication use**						
Antithrombotic agents	18.6	16.3	0.06	16.6	15.7	0.02
Beta blocking agents	14.6	16	-0.04	15.2	15.1	0
Calcium channel blockers	9.4	10.5	-0.04	10.1	9.5	0.02
Diuretics	14.1	16.2	-0.06	14.7	14.2	0.01
Drugs for acid-related disorders	26.1	24.2	0.05	26.2	26	0
Drugs for obstructive airway diseases	19.6	16	0.1	19.8	22.3	-0.06
Drugs used in diabetes	6.7	7.6	-0.04	7	7	0
Immunosuppressants	0.9	0.8	0.01	1	0.9	0.01
Lipid modifying agents	14.2	16.7	-0.07	15.3	14.9	0.01
Opioids	7.8	6.9	0.04	8.2	8.4	-0.01
Psycholeptics	12.4	11.5	0.03	13.1	13.4	-0.01
Psychostimulants, agents used for ADHD and nootropics	1	0.8	0.02	1	1.4	-0.03

Table S6.87. Selected baseline characteristics for Italy IQVIA, for the medium-term risk of substance misuse or dependence

Characteristic	Before propensity score matching			After propensity score matching		
	Targets, %	Comparators, %	Standardized difference	Targets, %	Comparators, %	Standardized difference
**Age group (years)**						
10-14	0.5	0.5	-0.01	0.4	0.7	-0.03
15-19	2.8	3.9	-0.06	3.2	3.6	-0.03
20-24	4.8	4.7	0	4.4	4.7	-0.01
25-29	5.6	4.9	0.03	4.8	5.1	-0.01
30-34	5.9	5.1	0.04	5.4	5.8	-0.02
35-39	6.5	5.6	0.04	6	5.9	0
40-44	8	6.7	0.05	7.7	8	-0.01
45-49	10.4	8.2	0.07	10.1	10.6	-0.02
50-54	10.7	9.2	0.05	10.7	11.2	-0.02
55-59	11.7	9.5	0.07	12.1	10.3	0.06
60-64	8.6	8.8	-0.01	9.4	9.2	0
65-69	6.2	8.1	-0.08	6.8	7	-0.01
70-74	5.8	8.1	-0.09	6.6	5.7	0.04
75-79	4	6.1	-0.1	4.4	4.3	0.01
80-84	4	5.5	-0.07	4.3	4.1	0.01
85-89	2.7	3.3	-0.04	2.5	2.4	0
90-94	1.4	1.5	0	1	1.1	-0.01
95-99	0.4	0.2	0.03	0.1	0.1	0
**Sex**						
Female	51.9	54.5	-0.05	54	53.9	0

Table S6.87. *Continued.* Selected baseline characteristics for Italy IQVIA, for the medium-term risk of substance misuse or dependence

Characteristic	Before propensity score matching			After propensity score matching		
	Targets, %	Comparators, %	Standardized difference	Targets, %	Comparators, %	Standardized difference
**Medical history (general)**						
Acute respiratory disease	24	13.3	0.28	25.4	30.6	-0.12
Chronic liver disease	1.9	1.8	0	1.9	2.3	-0.02
Chronic obstructive lung disease	1.8	1.8	0.01	2	1.6	0.03
Crohn’s disease	0.3	0.2	0.01	0.3	0.3	-0.01
Dementia	0.8	0.7	0.02	0.6	0.5	0.01
Depressive disorder	6.6	6.2	0.02	7.3	7.5	-0.01
Diabetes mellitus	7.7	8.5	-0.03	8.1	8	0
Gastroesophageal reflux disease	9	7.7	0.05	9.3	9.9	-0.02
Gastrointestinal hemorrhage	0.3	0.2	0	0.3	0.3	0
Human immunodeficiency virus infection	0.1	0.1	0.02	0.1	0.1	0.02
Hyperlipidemia	10.8	11.4	-0.02	11.6	11.4	0
Hypertensive disorder	25.4	27.3	-0.04	26.3	26.5	0
Lesion of liver	0.3	0.3	-0.01	0.2	0.3	-0.03
Obesity	0.5	0.3	0.03	0.4	0.4	0
Osteoarthritis	7.7	8.2	-0.02	7.9	8.8	-0.03
Pneumonia	9.7	0.9	0.4	4.1	3.5	0.03
Renal impairment	2.1	1.9	0.02	2.2	2.1	0
Rheumatoid arthritis	0.6	0.6	0	0.6	0.6	-0.01
Schizophrenia	0.1	0.2	-0.02	0.2	0.2	-0.01
Ulcerative colitis	0.4	0.3	0.02	0.4	0.4	0
Urinary tract infectious disease	2.8	2.1	0.04	2.8	3.2	-0.03
Viral hepatitis C	0.4	0.4	0	0.5	0.4	0.01

Table S6.87. *Continued.* Selected baseline characteristics for Italy IQVIA, for the medium-term risk of substance misuse or dependence

Characteristic	Before propensity score matching			After propensity score matching		
	Targets, %	Comparators, %	Standardized difference	Targets, %	Comparators, %	Standardized difference
**Medical history (cardiovascular disease)**						
Atrial fibrillation	3	3	0	3	3	0
Cerebrovascular disease	3.8	3.9	-0.01	3.6	3.5	0.01
Coronary arteriosclerosis	0.4	0.3	0.02	0.4	0.3	0
Heart disease	12.3	12.1	0	12.7	12.7	0
Heart failure	1.2	1.2	0.01	1	1.1	0
Ischemic heart disease	3.8	3.8	0	3.9	4	-0.01
Peripheral vascular disease	0.5	0.6	-0.01	0.4	0.6	-0.02
Pulmonary embolism	0.3	0.2	0.02	0.3	0.3	0
Venous thrombosis	0.5	0.5	0.01	0.6	0.8	-0.02
**Medical history (neoplasms)**						
Malignant lymphoma	0.4	0.3	0	0.3	0.5	-0.03
Malignant neoplasm of anorectum	0.1	0.2	-0.01	0.1	0.2	-0.01
Malignant neoplastic disease	6.2	6.1	0	6	6.2	-0.01
Malignant tumor of breast	1.4	1.5	-0.01	1.4	1.2	0.02
Malignant tumor of colon	0.4	0.5	-0.01	0.4	0.4	0
Malignant tumor of urinary bladder	0.4	0.4	0	0.4	0.3	0
Primary malignant neoplasm of prostate	0.8	0.8	0	0.8	0.7	0.01
**Medication use**						
Agents acting on the renin-angiotensin system	20.6	23.8	-0.08	22	21.7	0.01
Antibacterials for systemic use	48.3	32.1	0.33	42.7	45.2	-0.05
Antidepressants	10.7	9.9	0.03	11.3	11.1	0.01
Antiepileptics	4.6	4.4	0.01	5.2	5	0.01
Antiinflammatory and antirheumatic products	29.3	28.8	0.01	31.8	32.4	-0.01
Antineoplastic agents	2.1	2.2	-0.01	2.1	2.2	0

Table S6.87. *Continued.* Selected baseline characteristics for Italy IQVIA, for the medium-term risk of substance misuse or dependence

Characteristic	Before propensity score matching			After propensity score matching		
	Targets, %	Comparators, %	Standardized difference	Targets, %	Comparators, %	Standardized difference
**Medication use**						
Antipsoriatics	1.3	1.3	0	1.5	1.4	0
Antithrombotic agents	18.6	16.4	0.06	16.6	15.7	0.02
Beta blocking agents	14.6	16	-0.04	15.2	15.1	0
Calcium channel blockers	9.4	10.5	-0.04	10.1	9.5	0.02
Diuretics	14.1	16.3	-0.06	14.7	14.3	0.01
Drugs for acid-related disorders	26.1	24.3	0.04	26.3	26.1	0
Drugs for obstructive airway diseases	19.6	16	0.09	19.8	22.2	-0.06
Drugs used in diabetes	6.7	7.7	-0.04	7	7	0
Immunosuppressants	0.9	0.8	0.01	1	0.9	0.01
Lipid modifying agents	14.2	16.8	-0.07	15.3	14.9	0.01
Opioids	7.8	6.9	0.04	8.2	8.4	-0.01
Psycholeptics	12.4	11.4	0.03	13.1	13.3	-0.01
Psychostimulants, agents used for ADHD and nootropics	1	0.8	0.02	1	1.4	-0.03

Table S6.88. Selected baseline characteristics for Italy IQVIA, for the medium-term risk of bipolar disorders

Characteristic	Before propensity score matching			After propensity score matching		
	Targets, %	Comparators, %	Standardized difference	Targets, %	Comparators, %	Standardized difference
**Age group (years)**						
10-14	0.5	0.6	-0.01	0.5	0.7	-0.03
15-19	2.8	3.8	-0.06	3.2	3.6	-0.03
20-24	4.8	4.7	0	4.4	4.7	-0.02
25-29	5.6	4.9	0.03	4.8	5	-0.01
30-34	5.9	5	0.04	5.4	5.8	-0.02
35-39	6.5	5.5	0.04	6	5.9	0
40-44	8	6.6	0.05	7.7	8	-0.01
45-49	10.4	8.2	0.07	10.1	10.6	-0.02
50-54	10.7	9.2	0.05	10.7	11.2	-0.02
55-59	11.7	9.6	0.07	12.1	10.3	0.06
60-64	8.6	8.8	-0.01	9.3	9.3	0
65-69	6.2	8.2	-0.08	6.8	7	-0.01
70-74	5.8	8.2	-0.09	6.7	5.8	0.04
75-79	4	6	-0.09	4.4	4.3	0.01
80-84	4	5.6	-0.07	4.3	4.1	0.01
85-89	2.7	3.4	-0.04	2.5	2.4	0.01
90-94	1.4	1.5	0	1	1.1	-0.01
95-99	0.4	0.2	0.03	0.2	0.1	0
**Sex**						
Female	51.9	54.4	-0.05	54	53.7	0.01

Table S6.88. *Continued.* Selected baseline characteristics for Italy IQVIA, for the medium-term risk of bipolar disorders

Characteristic	Before propensity score matching			After propensity score matching		
	Targets, %	Comparators, %	Standardized difference	Targets, %	Comparators, %	Standardized difference
**Medical history (general)**						
Acute respiratory disease	24	13.2	0.28	25.4	30.6	-0.12
Chronic liver disease	1.9	1.8	0.01	1.9	2.3	-0.02
Chronic obstructive lung disease	1.8	1.8	0	2	1.7	0.02
Crohn’s disease	0.3	0.2	0.01	0.3	0.3	-0.01
Dementia	0.8	0.7	0.02	0.6	0.5	0.01
Depressive disorder	6.6	6.2	0.02	7.2	7.4	-0.01
Diabetes mellitus	7.7	8.5	-0.03	8.2	8	0.01
Gastroesophageal reflux disease	9	7.6	0.05	9.3	9.8	-0.02
Gastrointestinal hemorrhage	0.3	0.2	0	0.3	0.3	0
Human immunodeficiency virus infection	0.1	0.1	0.02	0.1	0.1	0.02
Hyperlipidemia	10.8	11.5	-0.02	11.5	11.4	0
Hypertensive disorder	25.4	27.2	-0.04	26.3	26.4	0
Lesion of liver	0.3	0.3	-0.01	0.2	0.3	-0.03
Obesity	0.5	0.3	0.03	0.4	0.4	0
Osteoarthritis	7.7	8.3	-0.02	7.9	8.8	-0.03
Pneumonia	9.7	0.9	0.4	4.2	3.6	0.03
Psoriasis	1.7	1.4	0.02	1.8	1.8	0
Renal impairment	2.1	1.9	0.02	2.2	2.1	0
Rheumatoid arthritis	0.6	0.7	-0.01	0.6	0.6	-0.01
Schizophrenia	0.1	0.2	-0.02	0.1	0.2	-0.02
Ulcerative colitis	0.4	0.3	0.02	0.4	0.4	0.01
Urinary tract infectious disease	2.8	2	0.05	2.8	3.2	-0.03
Viral hepatitis C	0.4	0.4	0	0.5	0.4	0.01

Table S6.88. *Continued.* Selected baseline characteristics for Italy IQVIA, for the medium-term risk of bipolar disorders

**Characteristic**	**Before propensity score matching**			**After propensity score matching**		
	**Targets, %**	**Comparators, %**	**Standardized difference**	**Targets, %**	**Comparators, %**	**Standardized difference**
**Medical history (cardiovascular disease)**						
Atrial fibrillation	3	2.9	0	3.1	3	0
Cerebrovascular disease	3.8	4	-0.01	3.6	3.5	0
Coronary arteriosclerosis	0.4	0.3	0.01	0.4	0.3	0.01
Heart disease	12.3	12.2	0	12.7	12.7	0
Heart failure	1.2	1.2	0	1.1	1.1	0
Peripheral vascular disease	0.5	0.6	-0.01	0.4	0.6	-0.02
Pulmonary embolism	0.3	0.2	0.02	0.3	0.3	0
Venous thrombosis	0.5	0.5	0.01	0.6	0.8	-0.02
**Medical history (neoplasms)**						
Malignant lymphoma	0.4	0.3	0.01	0.3	0.5	-0.03
Malignant neoplasm of anorectum	0.1	0.2	0	0.2	0.2	-0.01
Malignant neoplastic disease	6.2	6.1	0	6	6.2	-0.01
Malignant tumor of breast	1.4	1.5	-0.01	1.5	1.2	0.02
Malignant tumor of colon	0.4	0.5	-0.01	0.4	0.4	0
Malignant tumor of urinary bladder	0.4	0.4	-0.01	0.4	0.4	0
Primary malignant neoplasm of prostate	0.8	0.8	0	0.8	0.7	0.01
**Medication use**						
Agents acting on the renin-angiotensin system	20.6	23.7	-0.07	22	21.7	0.01
Antibacterials for systemic use	48.3	32.1	0.34	42.7	45.2	-0.05
Antidepressants	10.7	9.8	0.03	11.2	11	0
Antiepileptics	4.6	4.4	0.01	5	4.8	0.01
Antiinflammatory and antirheumatic products	29.3	29	0.01	31.6	32.5	-0.02
Antineoplastic agents	2.1	2.3	-0.01	2.1	2.2	0
Antipsoriatics	1.3	1.3	0	1.5	1.4	0
Antithrombotic agents	18.6	16.6	0.05	16.6	15.7	0.02

Table S6.88. *Continued.* Selected baseline characteristics for Italy IQVIA, for the medium-term risk of bipolar disorders

Characteristic	Before propensity score matching			After propensity score matching		
	Targets, %	Comparators, %	Standardized difference	Targets, %	Comparators, %	Standardized difference
**Medication use**						
Beta blocking agents	14.6	16.1	-0.04	15.1	15	0
Calcium channel blockers	9.4	10.5	-0.04	10.2	9.5	0.02
Diuretics	14.1	16.4	-0.06	14.7	14.3	0.01
Drugs for acid-related disorders	26.1	24.3	0.04	26.3	26	0.01
Drugs for obstructive airway diseases	19.6	16	0.1	19.8	22.3	-0.06
Drugs used in diabetes	6.7	7.7	-0.04	7	7	0
Immunosuppressants	0.9	0.8	0.01	1	0.9	0.01
Lipid modifying agents	14.2	16.9	-0.08	15.2	14.8	0.01
Opioids	7.8	6.8	0.04	8.1	8.5	-0.01
Psycholeptics	12.4	11.5	0.03	12.9	13.2	-0.01
Psychostimulants, agents used for ADHD and nootropics	1	0.8	0.02	1	1.4	-0.03

Table S6.89. Selected baseline characteristics for Italy IQVIA, for the medium-term risk of psychoses

Characteristic	Before propensity score matching			After propensity score matching		
	Targets, %	Comparators, %	Standardized difference	Targets, %	Comparators, %	Standardized difference
**Age group (years)**						
10-14	0.5	0.5	-0.01	0.4	0.7	-0.04
15-19	2.8	3.9	-0.06	3.2	3.6	-0.02
20-24	4.8	4.7	0	4.4	4.7	-0.02
25-29	5.6	4.8	0.03	4.8	5	-0.01
30-34	5.9	5.1	0.04	5.4	5.9	-0.02
40-44	8	6.6	0.05	7.7	8	-0.01
45-49	10.4	8.2	0.07	10.1	10.6	-0.02
50-54	10.7	9.1	0.05	10.7	11.2	-0.02
55-59	11.7	9.5	0.07	12.1	10.3	0.06
60-64	8.6	8.8	-0.01	9.3	9.2	0
65-69	6.2	8.2	-0.08	6.8	7	-0.01
70-74	5.8	8.2	-0.09	6.7	5.7	0.04
75-79	4	6	-0.09	4.4	4.2	0.01
80-84	4	5.5	-0.07	4.3	4.1	0.01
85-89	2.7	3.4	-0.04	2.5	2.4	0.01
90-94	1.4	1.5	-0.01	1	1	-0.01
95-99	0.4	0.2	0.03	0.1	0.1	0
**Sex**						
Female	51.9	54.6	-0.05	54	53.8	0

Table S6.89. *Continued.* Selected baseline characteristics for Italy IQVIA, for the medium-term risk of psychoses

Characteristic	Before propensity score matching			After propensity score matching		
	**Targets, %**	**Comparators, %**	**Standardized difference**	**Targets, %**	**Comparators, %**	**Standardized difference**
**Medical history (general)**						
Acute respiratory disease	24	13.4	0.28	25.5	30.6	-0.12
Chronic liver disease	1.9	1.9	0	1.9	2.3	-0.02
Chronic obstructive lung disease	1.8	1.8	0	2	1.6	0.03
Crohn's disease	0.3	0.2	0.01	0.3	0.3	-0.01
Dementia	0.8	0.7	0.02	0.6	0.5	0.01
Depressive disorder	6.6	6.2	0.02	7.3	7.5	-0.01
Diabetes mellitus	7.7	8.5	-0.03	8.1	8	0
Gastroesophageal reflux disease	9	7.6	0.05	9.4	9.8	-0.01
Gastrointestinal hemorrhage	0.3	0.2	0.01	0.3	0.3	-0.01
Human immunodeficiency virus infection	0.1	0.1	0.02	0.1	0.1	0.02
Hyperlipidemia	10.8	11.4	-0.02	11.6	11.4	0.01
Hypertensive disorder	25.4	27.2	-0.04	26.3	26.4	0
Lesion of liver	0.3	0.3	-0.01	0.2	0.3	-0.03
Obesity	0.5	0.3	0.03	0.4	0.4	0
Osteoarthritis	7.7	8.2	-0.02	7.9	8.7	-0.03
Pneumonia	9.7	0.9	0.4	4.1	3.6	0.03
Psoriasis	1.7	1.4	0.02	1.8	1.8	0
Renal impairment	2.1	1.9	0.02	2.2	2.1	0
Rheumatoid arthritis	0.6	0.7	-0.01	0.6	0.6	-0.01
Ulcerative colitis	0.4	0.3	0.02	0.4	0.4	0.01
Urinary tract infectious disease	2.8	2.1	0.05	2.8	3.2	-0.03
Viral hepatitis C	0.4	0.4	0	0.5	0.4	0.01

Table S6.89. *Continued.* Selected baseline characteristics for Italy IQVIA, for the medium-term risk of psychoses

Characteristic	Before propensity score matching			After propensity score matching		
	Targets, %	Comparators, %	Standardized difference	Targets, %	Comparators, %	Standardized difference
**Medical history (cardiovascular disease)**						
Medical history: Cardiovascular disease						
Atrial fibrillation	3	2.9	0	3	3	0
Cerebrovascular disease	3.8	4	-0.01	3.6	3.5	0
Coronary arteriosclerosis	0.4	0.3	0.01	0.4	0.3	0.01
Heart disease	12.3	12.2	0	12.7	12.7	0
Heart failure	1.2	1.2	0	1	1	0
Ischemic heart disease	3.8	3.8	0	3.8	4	-0.01
Peripheral vascular disease	0.5	0.6	-0.01	0.4	0.6	-0.02
Pulmonary embolism	0.3	0.2	0.02	0.3	0.3	0
Venous thrombosis	0.5	0.5	0.01	0.6	0.8	-0.02
**Medical history (neoplasms)**						
Malignant lymphoma	0.4	0.4	0	0.3	0.5	-0.03
Malignant neoplastic disease	6.2	6.1	0	6	6.2	-0.01
Malignant tumor of breast	1.4	1.5	-0.01	1.5	1.2	0.02
Malignant tumor of colon	0.4	0.5	-0.01	0.4	0.4	0
Malignant tumor of urinary bladder	0.4	0.4	0	0.4	0.3	0
Primary malignant neoplasm of prostate	0.8	0.7	0.01	0.7	0.7	0.01
**Medication use**						
Agents acting on the renin-angiotensin system	20.6	23.7	-0.07	22	21.7	0
Antibacterials for systemic use	48.3	32.2	0.33	42.8	45.2	-0.05
Antidepressants	10.7	9.8	0.03	11.3	11	0.01
Antiepileptics	4.6	4.4	0.01	5	4.9	0
Antiinflammatory and antirheumatic products	29.3	29	0.01	31.7	32.5	-0.01
Antipsoriatics	1.3	1.3	0	1.5	1.4	0.01
Antithrombotic agents	18.6	16.4	0.06	16.6	15.6	0.03

Table S6.89. *Continued.* Selected baseline characteristics for Italy IQVIA, for the medium-term risk of psychoses

Characteristic	Before propensity score matching			After propensity score matching		
	Targets, %	Comparators, %	Standardized difference	Targets, %	Comparators, %	Standardized difference
**Medication use**						
Beta blocking agents	14.6	16.1	-0.04	15.1	15.1	0
Calcium channel blockers	9.4	10.6	-0.04	10.1	9.4	0.02
Diuretics	14.1	16.3	-0.06	14.6	14.2	0.01
Drugs for acid-related disorders	26.1	24.3	0.04	26.3	26	0.01
Drugs for obstructive airway diseases	19.6	16	0.09	19.9	22.3	-0.06
Drugs used in diabetes	6.7	7.7	-0.04	6.9	7	0
Immunosuppressants	0.9	0.8	0.01	1	0.9	0.01
Lipid modifying agents	14.2	16.9	-0.08	15.3	14.8	0.01
Opioids	7.8	6.9	0.04	8.1	8.4	-0.01
Psycholeptics	12.4	11.5	0.03	12.8	13	-0.01
Psychostimulants, agents used for ADHD and nootropics	1	0.8	0.02	1	1.4	-0.03

Table S6.90. Selected baseline characteristics for Italy IQVIA, for the medium-term risk of personality disorders

Characteristic	Before propensity score matching			After propensity score matching		
	Targets, %	Comparators, %	Standardized difference	Targets, %	Comparators, %	Standardized difference
**Age group (years)**						
10-14	0.5	0.5	-0.01	0.5	0.7	-0.03
15-19	2.8	3.8	-0.05	3.2	3.7	-0.03
20-24	4.8	4.7	0	4.5	4.8	-0.01
25-29	5.6	4.9	0.03	4.8	5.1	-0.01
30-34	5.9	5.1	0.04	5.4	5.9	-0.02
35-39	6.5	5.6	0.04	6	6	0
40-44	8	6.7	0.05	7.8	8.1	-0.01
45-49	10.4	8.3	0.07	10.2	10.8	-0.02
50-54	10.7	9.3	0.05	10.8	11.3	-0.02
55-59	11.7	9.6	0.07	12.2	10.4	0.06
60-64	8.6	8.8	-0.01	9.4	9.3	0
65-69	6.2	8.1	-0.07	6.9	7.1	-0.01
70-74	5.8	8.2	-0.1	6.7	5.6	0.04
75-79	4	5.9	-0.09	4.3	4.1	0.01
80-84	4	5.5	-0.07	4	3.8	0.01
85-89	2.7	3.4	-0.04	2.2	2.2	0
90-94	1.4	1.5	0	0.9	1	-0.01
95-99	0.4	0.2	0.03	0.2	0.1	0
**Sex**						
Female	51.9	54.3	-0.05	53.8	53.6	0

Table S6.90. *Continued.* Selected baseline characteristics for Italy IQVIA, for the medium-term risk of personality disorders

Characteristic	Before propensity score matching			After propensity score matching		
	Targets, %	Comparators, %	Standardized difference	Targets, %	Comparators, %	Standardized difference
**Medical history (general)**						
Acute respiratory disease	24	13.2	0.28	25.5	30.5	-0.11
Chronic liver disease	1.9	1.9	0	1.9	2.2	-0.02
Chronic obstructive lung disease	1.8	1.7	0.01	1.9	1.6	0.02
Crohn's disease	0.3	0.2	0.01	0.3	0.3	-0.01
Dementia	0.8	0.7	0.02	0.5	0.5	0.01
Depressive disorder	6.6	6.1	0.02	7	7.3	-0.01
Diabetes mellitus	7.7	8.5	-0.03	8.1	7.8	0.01
Gastroesophageal reflux disease	9	7.7	0.05	9.2	9.7	-0.01
Gastrointestinal hemorrhage	0.3	0.2	0	0.3	0.3	0
Human immunodeficiency virus infection	0.1	0.1	0.02	0.1	0.1	0.02
Hyperlipidemia	10.8	11.3	-0.02	11.5	11.2	0.01
Hypertensive disorder	25.4	27.2	-0.04	26	26	0
Lesion of liver	0.3	0.3	-0.01	0.2	0.3	-0.02
Obesity	0.5	0.3	0.03	0.4	0.4	-0.01
Osteoarthritis	7.7	8.1	-0.02	7.7	8.5	-0.03
Pneumonia	9.7	0.9	0.4	4.1	3.5	0.03
Psoriasis	1.7	1.4	0.02	1.8	1.8	0
Renal impairment	2.1	1.9	0.01	2.1	2	0.01
Rheumatoid arthritis	0.6	0.7	-0.01	0.6	0.6	-0.01
Schizophrenia	0.1	0.2	-0.02	0.2	0.2	-0.01
Ulcerative colitis	0.4	0.3	0.02	0.4	0.4	0.01
Urinary tract infectious disease	2.8	2.1	0.04	2.6	3.1	-0.03
Viral hepatitis C	0.4	0.4	0.01	0.5	0.4	0.01

Table S6.90. *Continued.* Selected baseline characteristics for Italy IQVIA, for the medium-term risk of personality disorders

Characteristic	Before propensity score matching			After propensity score matching		
	**Targets, %**	**Comparators, %**	**Standardized difference**	**Targets, %**	**Comparators, %**	**Standardized difference**
**Medical history (cardiovascular disease)**						
Cerebrovascular disease	3.8	4	-0.01	3.4	3.3	0.01
Coronary arteriosclerosis	0.4	0.3	0.01	0.4	0.3	0.01
Heart disease	12.3	12.2	0	12.4	12.3	0
Ischemic heart disease	3.8	3.8	0	3.8	3.9	-0.01
Peripheral vascular disease	0.5	0.6	-0.01	0.5	0.6	-0.01
Pulmonary embolism	0.3	0.2	0.02	0.3	0.3	0.01
Venous thrombosis	0.5	0.4	0.01	0.6	0.8	-0.03
**Medical history (neoplasms)**						
Malignant lymphoma	0.4	0.3	0.01	0.3	0.5	-0.03
Malignant neoplastic disease	6.2	6.1	0	6	6.1	0
Malignant tumor of breast	1.4	1.5	-0.01	1.5	1.2	0.02
Malignant tumor of colon	0.4	0.5	-0.01	0.4	0.4	0
Malignant tumor of urinary bladder	0.4	0.4	0	0.3	0.3	0
Primary malignant neoplasm of prostate	0.8	0.7	0	0.7	0.6	0.01
**Medication use**						
Agents acting on the renin-angiotensin system	20.6	23.7	-0.07	21.7	21.4	0.01
Antibacterials for systemic use	48.3	32.1	0.33	42.7	45.1	-0.05
Antidepressants	10.7	9.8	0.03	10.9	10.7	0.01
Antiepileptics	4.6	4.4	0.01	5	4.9	0.01
Antiinflammatory and antirheumatic products	29.3	29.1	0.01	31.4	32.1	-0.01
Antineoplastic agents	2.1	2.2	-0.01	2.1	2.1	0
Antipsoriatics	1.3	1.3	0	1.5	1.4	0.01
Antithrombotic agents	18.6	16.4	0.06	16.1	15.2	0.03
Beta blocking agents	14.6	16.1	-0.04	15	14.8	0.01
Calcium channel blockers	9.4	10.5	-0.04	9.9	9.2	0.02

Table S6.90. *Continued.* Selected baseline characteristics for Italy IQVIA, for the medium-term risk of personality disorders

Characteristic	Before propensity score matching			After propensity score matching		
	**Targets, %**	**Comparators, %**	**Standardized difference**	**Targets, %**	**Comparators, %**	**Standardized difference**
**Medication use**						
Diuretics	14.1	16.3	-0.06	14.4	13.8	0.02
Drugs for acid-related disorders	26.1	24.4	0.04	25.8	25.6	0.01
Drugs for obstructive airway diseases	19.6	16	0.1	19.8	22.1	-0.06
Drugs used in diabetes	6.7	7.8	-0.04	7	6.9	0
Immunosuppressants	0.9	0.8	0.01	1	0.9	0.01
Lipid modifying agents	14.2	16.9	-0.07	15.1	14.6	0.01
Opioids	7.8	6.9	0.03	8	8.2	-0.01
Psycholeptics	12.4	11.5	0.03	12.6	13.1	-0.01
Psychostimulants, agents used for ADHD and nootropics	1	0.8	0.03	1	1.3	-0.03

Table S6.91. Selected baseline characteristics for Italy IQVIA, for the medium-term risk of self-harm and suicide

Characteristic	Before propensity score matching			After propensity score matching		
	**Targets, %**	**Comparators, %**	**Standardized difference**	**Targets, %**	**Comparators, %**	**Standardized difference**
**Age group (years)**						
10-14	0.5	0.5	-0.01	0.5	0.7	-0.03
15-19	2.8	3.8	-0.06	3.2	3.6	-0.03
20-24	4.8	4.7	0	4.4	4.7	-0.01
25-29	5.6	4.8	0.03	4.8	5	-0.01
30-34	5.9	5.1	0.04	5.4	5.8	-0.02
35-39	6.5	5.6	0.04	6	5.9	0
40-44	8	6.8	0.05	7.7	8	-0.01
45-49	10.4	8.4	0.07	10.1	10.7	-0.02
50-54	10.7	9.2	0.05	10.6	11.2	-0.02
55-59	11.7	9.5	0.07	12.1	10.3	0.06
60-64	8.6	8.8	-0.01	9.4	9.3	0
65-69	6.2	8.1	-0.08	6.8	7	-0.01
70-74	5.8	8.1	-0.09	6.7	5.7	0.04
75-79	4	6	-0.09	4.4	4.2	0.01
80-84	4	5.5	-0.07	4.3	4.1	0.01
85-89	2.7	3.3	-0.04	2.5	2.4	0.01
90-94	1.4	1.5	-0.01	1	1.1	-0.01
95-99	0.4	0.2	0.03	0.2	0.1	0
**Sex**						
Female	51.9	54.5	-0.05	54	53.7	0.01

Table S6.91. *Continued.* Selected baseline characteristics for Italy IQVIA, for the medium-term risk of self-harm and suicide

Characteristic	Before propensity score matching			After propensity score matching		
	Targets, %	Comparators, %	Standardized difference	Targets, %	Comparators, %	Standardized difference
**Medical history (general)**						
Acute respiratory disease	24	13.3	0.28	25.4	30.6	-0.12
Chronic liver disease	1.9	1.8	0.01	1.9	2.3	-0.02
Chronic obstructive lung disease	1.8	1.8	0.01	2	1.7	0.02
Crohn's disease	0.3	0.2	0.01	0.3	0.3	-0.01
Dementia	0.8	0.7	0.02	0.6	0.5	0.01
Depressive disorder	6.6	6.1	0.02	7.3	7.5	-0.01
Diabetes mellitus	7.7	8.4	-0.03	8.2	8	0.01
Gastroesophageal reflux disease	9	7.6	0.05	9.3	9.8	-0.02
Gastrointestinal hemorrhage	0.3	0.3	0	0.3	0.3	-0.01
Hyperlipidemia	10.8	11.4	-0.02	11.6	11.4	0.01
Hypertensive disorder	25.4	27.1	-0.04	26.3	26.4	0
Lesion of liver	0.3	0.3	0	0.2	0.3	-0.03
Obesity	0.5	0.3	0.03	0.4	0.4	0
Osteoarthritis	7.7	8.2	-0.02	7.9	8.8	-0.03
Pneumonia	9.7	0.9	0.4	4.1	3.6	0.03
Psoriasis	1.7	1.4	0.02	1.8	1.8	0
Renal impairment	2.1	2	0.01	2.2	2.1	0
Rheumatoid arthritis	0.6	0.6	-0.01	0.6	0.6	-0.01
Schizophrenia	0.1	0.2	-0.02	0.2	0.2	-0.01
Ulcerative colitis	0.4	0.3	0.01	0.4	0.4	0.01
Urinary tract infectious disease	2.8	2.1	0.04	2.8	3.2	-0.03
Viral hepatitis C	0.4	0.4	0	0.5	0.4	0.01

Table S6.91. *Continued.* Selected baseline characteristics for Italy IQVIA, for the medium-term risk of self-harm and suicide

**Characteristic**	**Before propensity score matching**			**After propensity score matching**		
	**Targets, %**	**Comparators, %**	**Standardized difference**	**Targets, %**	**Comparators, %**	**Standardized difference**
**Medical history (cardiovascular disease)**						
Atrial fibrillation	3	3	0	3.1	3	0
Cerebrovascular disease	3.8	4	-0.01	3.6	3.5	0
Coronary arteriosclerosis	0.4	0.3	0.01	0.4	0.3	0.01
Heart disease	12.3	12.2	0	12.7	12.8	0
Heart failure	1.2	1.2	0	1	1.1	0
Ischemic heart disease	3.8	3.8	0	3.9	4	-0.01
Peripheral vascular disease	0.5	0.6	-0.01	0.4	0.6	-0.02
Pulmonary embolism	0.3	0.2	0.02	0.3	0.3	0
Venous thrombosis	0.5	0.5	0.01	0.6	0.8	-0.02
**Medical history (neoplasms)**						
Malignant lymphoma	0.4	0.3	0	0.3	0.5	-0.03
Malignant neoplasm of anorectum	0.1	0.2	0	0.2	0.2	-0.01
Malignant neoplastic disease	6.2	6.1	0	6	6.2	-0.01
Malignant tumor of breast	1.4	1.5	-0.01	1.5	1.2	0.02
Malignant tumor of colon	0.4	0.5	-0.01	0.4	0.4	0
Malignant tumor of urinary bladder	0.4	0.4	0	0.3	0.4	0
Primary malignant neoplasm of prostate	0.8	0.7	0.01	0.8	0.7	0.01
**Medication use**						
Agents acting on the renin-angiotensin system	20.6	23.5	-0.07	22	21.8	0.01
Antibacterials for systemic use	48.3	32.1	0.33	42.8	45.2	-0.05
Antidepressants	10.7	9.8	0.03	11.4	11.2	0.01
Antiepileptics	4.6	4.4	0.01	5.2	5.1	0
Antiinflammatory and antirheumatic products	29.3	29.1	0.01	31.7	32.5	-0.02
Antineoplastic agents	2.1	2.2	-0.01	2.2	2.2	0
Antipsoriatics	1.3	1.3	0	1.5	1.4	0

Table S6.91. *Continued.* Selected baseline characteristics for Italy IQVIA, for the medium-term risk of self-harm and suicide

Characteristic	Before propensity score matching			After propensity score matching		
	**Targets, %**	**Comparators, %**	**Standardized difference**	**Targets, %**	**Comparators, %**	**Standardized difference**
**Medication use**						
Antithrombotic agents	18.6	16.4	0.06	16.6	15.7	0.03
Beta blocking agents	14.6	16	-0.04	15.2	15	0
Calcium channel blockers	9.4	10.6	-0.04	10.1	9.5	0.02
Diuretics	14.1	16.2	-0.06	14.7	14.3	0.01
Drugs for acid-related disorders	26.1	24.3	0.04	26.3	26	0
Drugs for obstructive airway diseases	19.6	16	0.09	19.8	22.3	-0.06
Immunosuppressants	0.9	0.8	0.01	1	0.9	0.01
Lipid modifying agents	14.2	16.8	-0.07	15.3	14.9	0.01
Opioids	7.8	6.9	0.04	8.2	8.4	-0.01
Psycholeptics	12.4	11.5	0.03	13.1	13.4	-0.01
Psychostimulants, agents used for ADHD and nootropics	1	0.8	0.03	1	1.4	-0.03

Table S6.92. Selected baseline characteristics for Italy IQVIA, for the medium-term risk of sleep disorders

Characteristic	Before propensity score matching			After propensity score matching		
	Targets, %	Comparators, %	Standardized difference	Targets, %	Comparators, %	Standardized difference
**Age group (years)**						
10-14	0.5	0.5	-0.01	0.5	0.7	-0.04
15-19	2.8	3.9	-0.06	3.3	3.8	-0.03
20-24	4.8	4.7	0	4.6	4.9	-0.01
25-29	5.6	4.8	0.03	5	5.2	-0.01
30-34	5.9	5	0.04	5.6	6.1	-0.02
35-39	6.5	5.6	0.04	6.2	6	0
40-44	8	6.8	0.05	7.8	8.2	-0.01
45-49	10.4	8.3	0.07	10.2	10.8	-0.02
50-54	10.7	9.2	0.05	10.7	11.2	-0.02
55-59	11.7	9.6	0.07	12.2	10.3	0.06
65-69	6.2	8.2	-0.08	6.6	6.9	-0.01
70-74	5.8	8.1	-0.09	6.6	5.5	0.04
75-79	4	6.1	-0.1	4	3.9	0
80-84	4	5.5	-0.07	4.1	3.8	0.01
85-89	2.7	3.3	-0.04	2.5	2.3	0.01
90-94	1.4	1.5	0	0.9	1	-0.01
95-99	0.4	0.2	0.03	0.1	0.1	0
**Sex**						
Female	51.9	54.5	-0.05	53.8	53.4	0.01

Table S6.92. *Continued.* Selected baseline characteristics for Italy IQVIA, for the medium-term risk of sleep disorders

Characteristic	Before propensity score matching			After propensity score matching		
	Targets, %	Comparators, %	Standardized difference	Targets, %	Comparators, %	Standardized difference
**Medical history (general)**						
Acute respiratory disease	24	13.3	0.28	25.1	30.2	-0.11
Chronic liver disease	1.9	1.8	0	1.8	2.2	-0.03
Chronic obstructive lung disease	1.8	1.8	0	1.8	1.6	0.01
Crohn’s disease	0.3	0.2	0.01	0.3	0.3	-0.01
Dementia	0.8	0.7	0.02	0.6	0.5	0.01
Depressive disorder	6.6	6.1	0.02	6.8	6.9	0
Diabetes mellitus	7.7	8.5	-0.03	7.9	7.7	0.01
Gastroesophageal reflux disease	9	7.6	0.05	8.7	9.1	-0.01
Hyperlipidemia	10.8	11.4	-0.02	11.2	10.8	0.01
Lesion of liver	0.3	0.3	-0.01	0.2	0.3	-0.02
Obesity	0.5	0.3	0.03	0.3	0.4	0
Osteoarthritis	7.7	8.1	-0.02	7.4	8.1	-0.03
Pneumonia	9.7	0.9	0.4	4	3.5	0.03
Psoriasis	1.7	1.4	0.03	1.8	1.7	0
Renal impairment	2.1	1.9	0.02	2	2	0.01
Rheumatoid arthritis	0.6	0.7	-0.01	0.6	0.6	0
Schizophrenia	0.1	0.2	-0.02	0.1	0.2	-0.02
Ulcerative colitis	0.4	0.3	0.02	0.4	0.4	0.01
Urinary tract infectious disease	2.8	2.1	0.04	2.5	2.9	-0.02
Viral hepatitis C	0.4	0.4	0.01	0.4	0.4	0

Table S6.92. *Continued.* Selected baseline characteristics for Italy IQVIA, for the medium-term risk of sleep disorders

**Characteristic**	**Before propensity score matching**			**After propensity score matching**		
	**Targets, %**	**Comparators, %**	**Standardized difference**	**Targets, %**	**Comparators, %**	**Standardized difference**
**Medical history (cardiovascular disease)**						
Cerebrovascular disease	3.8	4	-0.01	3.4	3.3	0
Coronary arteriosclerosis	0.4	0.3	0.02	0.3	0.3	0
Heart disease	12.3	12.2	0	11.9	12.1	-0.01
Heart failure	1.2	1.2	0	0.9	0.9	0
Ischemic heart disease	3.8	3.8	0	3.5	3.9	-0.02
Peripheral vascular disease	0.5	0.6	-0.01	0.5	0.6	-0.02
Pulmonary embolism	0.3	0.2	0.02	0.3	0.3	0
Venous thrombosis	0.5	0.5	0.01	0.6	0.7	-0.01
**Medical history (neoplasms)**						
Malignant lymphoma	0.4	0.4	0	0.3	0.5	-0.03
Malignant neoplasm of anorectum	0.1	0.2	-0.01	0.1	0.2	0
Malignant tumor of breast	1.4	1.6	-0.02	1.4	1.2	0.02
Malignant tumor of colon	0.4	0.5	-0.01	0.4	0.4	0
Malignant tumor of urinary bladder	0.4	0.4	0	0.3	0.3	-0.01
Primary malignant neoplasm of prostate	0.8	0.7	0.01	0.7	0.6	0
**Medication use**						
Agents acting on the renin-angiotensin system	20.6	23.6	-0.07	21.1	21	0
Antibacterials for systemic use	48.3	32.2	0.33	42.2	44.7	-0.05
Antidepressants	10.7	9.8	0.03	10.4	10.3	0.01
Antiepileptics	4.6	4.4	0.01	4.9	4.7	0.01
Antiinflammatory and antirheumatic products	29.3	29.1	0.01	30.7	31.6	-0.02
Antineoplastic agents	2.1	2.2	-0.01	2.2	2.1	0
Antipsoriatics	1.3	1.3	0.01	1.3	1.4	0
Antithrombotic agents	18.6	16.5	0.06	15.9	15	0.02
Beta blocking agents	14.6	16	-0.04	14.7	14.4	0.01

Table S6.92. *Continued.* Selected baseline characteristics for Italy IQVIA, for the medium-term risk of sleep disorders

Characteristic	Before propensity score matching			After propensity score matching		
	**Targets, %**	**Comparators, %**	**Standardized difference**	**Targets, %**	**Comparators, %**	**Standardized difference**
**Medication use**						
Calcium channel blockers	9.4	10.5	-0.04	9.7	9	0.03
Diuretics	14.1	16.3	-0.06	14.2	13.5	0.02
Drugs for acid-related disorders	26.1	24.3	0.04	25.3	24.8	0.01
Drugs for obstructive airway diseases	19.6	16	0.1	19.3	21.9	-0.06
Drugs used in diabetes	6.7	7.7	-0.04	6.8	6.8	0
Immunosuppressants	0.9	0.8	0	1	0.8	0.02
Lipid modifying agents	14.2	16.8	-0.07	14.8	14.3	0.01
Opioids	7.8	6.9	0.04	7.7	7.9	-0.01
Psycholeptics	12.4	11.4	0.03	11	11.2	-0.01
Psychostimulants, agents used for ADHD and nootropics	1	0.8	0.02	0.9	1.3	-0.04

Table S6.93. Selected baseline characteristics for Italy IQVIA, for the medium-term risk of dementia

Characteristic	Before propensity score matching			After propensity score matching		
	Targets, %	Comparators, %	Standardized difference	Targets, %	Comparators, %	Standardized difference
**Age group (years)**						
10-14	0.5	0.5	-0.01	0.4	0.7	-0.03
15-19	2.8	3.8	-0.06	3.2	3.6	-0.03
20-24	4.8	4.8	0	4.4	4.7	-0.02
25-29	5.6	4.9	0.03	4.8	5.1	-0.01
30-34	5.9	5	0.04	5.4	5.9	-0.02
35-39	6.5	5.5	0.04	6	6	0
40-44	8	6.7	0.05	7.8	8	-0.01
45-49	10.4	8.2	0.07	10.1	10.7	-0.02
50-54	10.7	9.2	0.05	10.8	11.3	-0.02
55-59	11.7	9.6	0.07	12.2	10.4	0.06
60-64	8.6	8.8	-0.01	9.4	9.3	0
65-69	6.2	8	-0.07	6.8	7.1	-0.01
70-74	5.8	8.2	-0.09	6.7	5.7	0.04
75-79	4	6.1	-0.1	4.3	4.2	0.01
80-84	4	5.5	-0.07	4.2	3.9	0.01
85-89	2.7	3.4	-0.04	2.4	2.2	0.01
90-94	1.4	1.5	-0.01	0.8	0.9	-0.01
95-99	0.4	0.2	0.04	0.1	0.1	0
**Sex**						
Female	51.9	54.6	-0.05	53.9	53.7	0

Table S6.93. *Continued.* Selected baseline characteristics for Italy IQVIA, for the medium-term risk of dementia

Characteristic	Before propensity score matching			After propensity score matching		
	Targets, %	Comparators, %	Standardized difference	Targets, %	Comparators, %	Standardized difference
**Medical history (general)**						
Acute respiratory disease	24	13.2	0.28	25.5	30.6	-0.12
Chronic liver disease	1.9	1.8	0.01	1.9	2.3	-0.03
Chronic obstructive lung disease	1.8	1.8	0.01	2	1.7	0.02
Crohn’s disease	0.3	0.2	0.01	0.3	0.3	-0.01
Depressive disorder	6.6	6.1	0.02	7.2	7.4	-0.01
Diabetes mellitus	7.7	8.5	-0.03	8.1	7.9	0.01
Gastroesophageal reflux disease	9	7.6	0.05	9.3	9.8	-0.02
Gastrointestinal hemorrhage	0.3	0.3	0	0.3	0.3	-0.01
Hyperlipidemia	10.8	11.4	-0.02	11.5	11.3	0.01
Lesion of liver	0.3	0.3	-0.01	0.2	0.3	-0.03
Obesity	0.5	0.3	0.03	0.4	0.4	0
Osteoarthritis	7.7	8.2	-0.02	7.9	8.7	-0.03
Pneumonia	9.7	0.9	0.4	4.1	3.5	0.03
Psoriasis	1.7	1.4	0.02	1.8	1.8	0
Renal impairment	2.1	1.9	0.02	2.2	2	0.01
Rheumatoid arthritis	0.6	0.7	-0.01	0.6	0.6	-0.01
Schizophrenia	0.1	0.2	-0.02	0.2	0.2	-0.01
Ulcerative colitis	0.4	0.3	0.02	0.4	0.4	0.01
Urinary tract infectious disease	2.8	2.1	0.04	2.7	3.1	-0.03
Viral hepatitis C	0.4	0.4	0.01	0.5	0.4	0.01

Table S6.93. *Continued.* Selected baseline characteristics for Italy IQVIA, for the medium-term risk of dementia

Characteristic	Before propensity score matching			After propensity score matching		
	Targets, %	Comparators, %	Standardized difference	Targets, %	Comparators, %	Standardized difference
**Medical history (cardiovascular disease)**						
Cerebrovascular disease	3.8	3.9	-0.01	3.5	3.4	0.01
Coronary arteriosclerosis	0.4	0.3	0.02	0.4	0.3	0.01
Heart disease	12.3	12.2	0	12.7	12.5	0
Heart failure	1.2	1.2	0.01	1	1	0
Peripheral vascular disease	0.5	0.6	-0.01	0.4	0.6	-0.02
Pulmonary embolism	0.3	0.2	0.03	0.3	0.3	0
Venous thrombosis	0.5	0.5	0.01	0.6	0.8	-0.02
**Medical history (neoplasms)**						
Malignant lymphoma	0.4	0.3	0.01	0.3	0.5	-0.03
Malignant neoplasm of anorectum	0.1	0.2	0	0.2	0.2	-0.01
Malignant neoplastic disease	6.2	6.1	0	6	6.2	-0.01
Malignant tumor of breast	1.4	1.5	-0.01	1.4	1.2	0.02
Malignant tumor of colon	0.4	0.5	-0.01	0.5	0.4	0.01
Malignant tumor of urinary bladder	0.4	0.4	0	0.3	0.4	0
Primary malignant neoplasm of prostate	0.8	0.7	0.01	0.7	0.7	0.01
**Medication use**						
Agents acting on the renin-angiotensin system	20.6	23.6	-0.07	21.9	21.6	0.01
Antibacterials for systemic use	48.3	31.9	0.34	42.7	45.1	-0.05
Antidepressants	10.7	9.8	0.03	11.1	10.9	0.01
Antiepileptics	4.6	4.4	0.01	5.1	5	0
Antiinflammatory and antirheumatic products	29.3	29	0.01	31.7	32.4	-0.01
Antineoplastic agents	2.1	2.3	-0.01	2.1	2.2	0
Antipsoriatics	1.3	1.3	0	1.5	1.4	0.01
Antithrombotic agents	18.6	16.5	0.06	16.3	15.4	0.03

Table S6.93. *Continued.* Selected baseline characteristics for Italy IQVIA, for the medium-term risk of dementia

Characteristic	Before propensity score matching			After propensity score matching		
	Targets, %	Comparators, %	Standardized difference	Targets, %	Comparators, %	Standardized difference
**Medication use**						
Beta blocking agents	14.6	16.1	-0.04	15.2	14.9	0.01
Calcium channel blockers	9.4	10.5	-0.04	10.1	9.4	0.03
Diuretics	14.1	16.3	-0.06	14.6	14	0.02
Drugs for acid-related disorders	26.1	24.2	0.04	26.2	25.8	0.01
Drugs for obstructive airway diseases	19.6	15.8	0.1	19.8	22.2	-0.06
Immunosuppressants	0.9	0.8	0.01	1	0.9	0.02
Lipid modifying agents	14.2	16.9	-0.08	15.3	14.7	0.02
Opioids	7.8	6.9	0.03	8.2	8.4	-0.01
Psycholeptics	12.4	11.6	0.03	12.8	13.1	-0.01
Psychostimulants, agents used for ADHD and nootropics	1	0.8	0.02	1	1.4	-0.03

Table S6.94. Selected baseline characteristics for Italy IQVIA, for the medium-term risk of neurodevelopmental disorders

Characteristic	Before propensity score matching			After propensity score matching		
	Targets, %	Comparators, %	Standardized difference	Targets, %	Comparators, %	Standardized difference
**Age group (years)**						
10-14	0.5	0.5	-0.01	0.4	0.7	-0.03
15-19	2.8	3.9	-0.06	3.1	3.6	-0.03
20-24	4.8	4.7	0	4.4	4.7	-0.01
25-29	5.6	4.8	0.04	4.8	5	-0.01
30-34	5.9	5.1	0.04	5.4	5.8	-0.02
35-39	6.5	5.5	0.04	6	5.9	0
40-44	8	6.8	0.05	7.6	8	-0.01
45-49	10.4	8.2	0.07	10.1	10.7	-0.02
50-54	10.7	9.2	0.05	10.7	11.3	-0.02
55-59	11.7	9.5	0.07	12.2	10.3	0.06
60-64	8.6	8.8	-0.01	9.4	9.3	0
65-69	6.2	8.1	-0.08	6.8	7.1	-0.01
70-74	5.8	8.1	-0.09	6.7	5.8	0.04
75-79	4	6.1	-0.1	4.4	4.3	0.01
80-84	4	5.4	-0.07	4.3	4.1	0.01
85-89	2.7	3.3	-0.04	2.5	2.4	0.01
90-94	1.4	1.5	-0.01	1	1.1	-0.01
95-99	0.4	0.2	0.03	0.2	0.2	0
**Sex**						
Female	51.9	54.5	-0.05	54	53.8	0

Table S6.94. *Continued.* Selected baseline characteristics for Italy IQVIA, for the medium-term risk of neurodevelopmental disorders

Characteristic	Before propensity score matching			After propensity score matching		
	**Targets, %**	**Comparators, %**	**Standardized difference**	**Targets, %**	**Comparators, %**	**Standardized difference**
**Medical history (general)**						
Acute respiratory disease	24	13.3	0.28	25.4	30.5	-0.12
Chronic liver disease	1.9	1.8	0	1.9	2.3	-0.03
Chronic obstructive lung disease	1.8	1.8	0.01	2	1.7	0.02
Crohn’s disease	0.3	0.2	0.01	0.3	0.3	-0.01
Dementia	0.8	0.7	0.02	0.6	0.5	0.01
Depressive disorder	6.6	6.1	0.02	7.3	7.5	-0.01
Diabetes mellitus	7.7	8.5	-0.03	8.2	8	0
Gastroesophageal reflux disease	9	7.6	0.05	9.3	9.8	-0.02
Gastrointestinal hemorrhage	0.3	0.2	0.01	0.3	0.3	0
Human immunodeficiency virus infection	0.1	0.1	0.02	0.1	0.1	0.02
Hyperlipidemia	10.8	11.4	-0.02	11.6	11.4	0.01
Hypertensive disorder	25.4	27.3	-0.04	26.4	26.5	0
Lesion of liver	0.3	0.3	-0.01	0.2	0.3	-0.03
Obesity	0.5	0.3	0.03	0.4	0.4	0
Osteoarthritis	7.7	8.2	-0.02	7.9	8.7	-0.03
Pneumonia	9.7	0.9	0.4	4.1	3.6	0.03
Psoriasis	1.7	1.4	0.02	1.8	1.8	0
Renal impairment	2.1	1.9	0.02	2.2	2.1	0
Rheumatoid arthritis	0.6	0.7	-0.01	0.6	0.6	-0.01
Schizophrenia	0.1	0.2	-0.02	0.2	0.2	-0.01
Ulcerative colitis	0.4	0.3	0.02	0.4	0.4	0.01
Urinary tract infectious disease	2.8	2.1	0.04	2.8	3.2	-0.03
Viral hepatitis C	0.4	0.4	0	0.5	0.4	0.01

Table S6.94. *Continued.* Selected baseline characteristics for Italy IQVIA, for the medium-term risk of neurodevelopmental disorders

Characteristic	Before propensity score matching			After propensity score matching		
	Targets, %	Comparators, %	Standardized difference	Targets, %	Comparators, %	Standardized difference
**Medical history (cardiovascular disease)**						
Atrial fibrillation	3	2.9	0	3.1	3	0
Cerebrovascular disease	3.8	3.9	-0.01	3.6	3.5	0.01
Coronary arteriosclerosis	0.4	0.3	0.01	0.4	0.3	0.01
Heart disease	12.3	12.2	0	12.7	12.8	0
Heart failure	1.2	1.2	0	1	1.1	0
Ischemic heart disease	3.8	3.8	0	3.9	4	-0.01
Peripheral vascular disease	0.5	0.6	-0.01	0.5	0.6	-0.02
Pulmonary embolism	0.3	0.2	0.02	0.3	0.3	0
Venous thrombosis	0.5	0.4	0.01	0.6	0.8	-0.02
**Medical history (neoplasms)**						
Malignant lymphoma	0.4	0.3	0.01	0.3	0.5	-0.03
Malignant neoplasm of anorectum	0.1	0.2	0	0.2	0.2	-0.01
Malignant neoplastic disease	6.2	6.1	0	6.1	6.2	-0.01
Malignant tumor of breast	1.4	1.5	-0.01	1.5	1.2	0.02
Malignant tumor of colon	0.4	0.5	-0.01	0.4	0.4	0
Primary malignant neoplasm of prostate	0.8	0.8	0	0.8	0.7	0.01
**Medication use**						
Agents acting on the renin-angiotensin system	20.6	23.7	-0.07	22.1	21.8	0.01
Antibacterials for systemic use	48.3	32.2	0.33	42.7	45.2	-0.05
Antidepressants	10.7	9.8	0.03	11.4	11.2	0.01
Antiepileptics	4.6	4.4	0.01	5.1	5	0.01
Antiinflammatory and antirheumatic products	29.3	29	0.01	31.8	32.4	-0.01
Antineoplastic agents	2.1	2.2	-0.01	2.2	2.2	0
Antipsoriatics	1.3	1.3	0	1.5	1.4	0.01
Antithrombotic agents	18.6	16.4	0.06	16.6	15.7	0.03

Table S6.94. *Continued.* Selected baseline characteristics for Italy IQVIA, for the medium-term risk of neurodevelopmental disorders

Characteristic	Before propensity score matching			After propensity score matching		
	Targets, %	Comparators, %	Standardized difference	Targets, %	Comparators, %	Standardized difference
**Medication use**						
Beta blocking agents	14.6	16.2	-0.04	15.3	15.1	0.01
Calcium channel blockers	9.4	10.6	-0.04	10.2	9.5	0.02
Diuretics	14.1	16.3	-0.06	14.8	14.3	0.01
Drugs for acid-related disorders	26.1	24.3	0.04	26.3	26	0.01
Drugs for obstructive airway diseases	19.6	16.1	0.09	19.9	22.3	-0.06
Drugs used in diabetes	6.7	7.7	-0.04	7	7	0
Immunosuppressants	0.9	0.8	0.01	1	0.9	0.01
Lipid modifying agents	14.2	16.9	-0.07	15.4	14.9	0.01
Opioids	7.8	6.9	0.04	8.1	8.4	-0.01
Psycholeptics	12.4	11.5	0.03	13.1	13.4	-0.01
Psychostimulants, agents used for ADHD and nootropics	1	0.8	0.02	1	1.4	-0.03

Table S6.95. Selected baseline characteristics for Italy IQVIA, for the medium-term risk of any of psychiatric and neuropsychiatric disorders

Characteristic	Before propensity score matching			After propensity score matching		
	**Targets, %**	**Comparators, %**	**Standardized difference**	**Targets, %**	**Comparators, %**	**Standardized difference**
**Age group (years)**						
10-14	0.5	0.5	-0.01	0.5	0.8	-0.03
15-19	2.8	3.8	-0.06	3.5	4.1	-0.03
20-24	4.8	4.8	0	5.1	5.4	-0.01
25-29	5.6	4.9	0.03	5.4	5.6	-0.01
30-34	5.9	5.1	0.04	6	6.4	-0.02
35-39	6.5	5.5	0.04	6.1	6.4	-0.01
40-44	8	6.7	0.05	8.1	8.5	-0.01
45-49	10.4	8.2	0.07	10.5	10.9	-0.01
50-54	10.7	9.2	0.05	11.1	11.3	-0.01
55-59	11.7	9.5	0.07	11.7	10.3	0.04
60-64	8.6	8.9	-0.01	9.3	8.9	0.01
65-69	6.2	8.2	-0.08	6.8	6.8	0
70-74	5.8	8.2	-0.09	6.2	5.1	0.05
75-79	4	6	-0.09	3.8	3.5	0.01
80-84	4	5.5	-0.07	3.4	3.2	0.01
85-89	2.7	3.4	-0.04	1.8	1.8	0
90-94	1.4	1.5	-0.01	0.6	0.8	-0.03
95-99	0.4	0.2	0.04	0.1	0.1	0
**Sex**						
Female	51.9	54.5	-0.05	51.7	51.9	0

Table S6.95. *Continued.* Selected baseline characteristics for Italy IQVIA, for the medium-term risk of any of psychiatric and neuropsychiatric disorders

Characteristic	Before propensity score matching			After propensity score matching		
	Targets, %	Comparators, %	Standardized difference	Targets, %	Comparators, %	Standardized difference
**Medical history (general)**						
Acute respiratory disease	24	13.1	0.28	24.8	29.7	-0.11
Chronic liver disease	1.9	1.8	0	1.7	1.9	-0.01
Chronic obstructive lung disease	1.8	1.7	0.01	1.6	1.4	0.02
Crohn’s disease	0.3	0.2	0.01	0.3	0.3	0
Diabetes mellitus	7.7	8.5	-0.03	7.2	7.2	0
Gastroesophageal reflux disease	9	7.6	0.05	8	8.1	-0.01
Human immunodeficiency virus infection	0.1	0.1	0.02	0.1	0.1	0.02
Hyperlipidemia	10.8	11.4	-0.02	10.3	10	0.01
Hypertensive disorder	25.4	27.2	-0.04	23.9	23.7	0.01
Lesion of liver	0.3	0.3	-0.01	0.2	0.3	-0.01
Obesity	0.5	0.3	0.03	0.3	0.3	0
Osteoarthritis	7.7	8.2	-0.02	6.6	7.1	-0.02
Pneumonia	9.7	0.9	0.4	3.7	3.3	0.02
Psoriasis	1.7	1.4	0.02	1.6	1.5	0.01
Renal impairment	2.1	1.9	0.02	1.9	1.7	0.01
Rheumatoid arthritis	0.6	0.6	-0.01	0.5	0.6	-0.02
Ulcerative colitis	0.4	0.3	0.02	0.4	0.4	0
Urinary tract infectious disease	2.8	2.1	0.04	2.2	2.6	-0.02
Viral hepatitis C	0.4	0.4	0	0.4	0.4	0

Table S6.95. *Continued.* Selected baseline characteristics for Italy IQVIA, for the medium-term risk of any of psychiatric and neuropsychiatric disorders

Characteristic	Before propensity score matching			After propensity score matching		
	Targets, %	Comparators, %	Standardized difference	Targets, %	Comparators, %	Standardized difference
**Medical history (cardiovascular disease)**						
Atrial fibrillation	3	3	0	2.4	2.5	0
Cerebrovascular disease	3.8	4	-0.01	2.7	2.7	0.01
Coronary arteriosclerosis	0.4	0.3	0.02	0.3	0.3	0
Heart disease	12.3	12.2	0	10.8	11	0
Heart failure	1.2	1.2	0.01	0.8	0.8	0.01
Ischemic heart disease	3.8	3.9	0	3.3	3.5	-0.01
Peripheral vascular disease	0.5	0.6	-0.01	0.4	0.5	-0.01
Pulmonary embolism	0.3	0.2	0.02	0.3	0.2	0.01
Venous thrombosis	0.5	0.4	0.01	0.5	0.7	-0.02
**Medical history (neoplasms)**						
Malignant lymphoma	0.4	0.3	0.01	0.2	0.4	-0.03
Malignant neoplasm of anorectum	0.1	0.2	0	0.1	0.2	-0.01
Malignant neoplastic disease	6.2	6.2	0	5.1	5.6	-0.02
Malignant tumor of breast	1.4	1.5	-0.01	1.2	1.1	0
Malignant tumor of colon	0.4	0.5	-0.01	0.4	0.4	0
Malignant tumor of urinary bladder	0.4	0.4	-0.01	0.3	0.3	-0.01
**Medication use**						
Agents acting on the renin-angiotensin system	20.6	23.5	-0.07	20	19.8	0.01
Antibacterials for systemic use	48.3	32	0.34	41.1	43.9	-0.06
Antidepressants	10.7	9.8	0.03	4	4.2	-0.01
Antiepileptics	4.6	4.4	0.01	3.3	3.2	0
Antiinflammatory and antirheumatic products	29.3	29	0.01	29.1	30	-0.02
Antineoplastic agents	2.1	2.3	-0.01	1.8	1.9	-0.01

Table S6.95. *Continued.* Selected baseline characteristics for Italy IQVIA, for the medium-term risk of any of psychiatric and neuropsychiatric disorders

	Before propensity score matching			After propensity score matching		
	**Targets, %**	**Comparators, %**	**Standardized difference**	**Targets, %**	**Comparators, %**	**Standardized difference**
**Medication use**						
Antipsoriatics	1.3	1.3	0	1.4	1.3	0.01
Antithrombotic agents	18.6	16.5	0.06	13.8	13.6	0
Beta blocking agents	14.6	16	-0.04	13.1	13.3	-0.01
Calcium channel blockers	9.4	10.5	-0.04	9	8.4	0.02
Diuretics	14.1	16.3	-0.06	13	12.5	0.01
Drugs for acid-related disorders	26.1	24.3	0.04	22.8	22.7	0
Drugs for obstructive airway diseases	19.6	16.1	0.09	18.5	21.1	-0.07
Drugs used in diabetes	6.7	7.6	-0.04	6.2	6.4	-0.01
Immunosuppressants	0.9	0.8	0.01	0.9	0.8	0.02
Lipid modifying agents	14.2	16.9	-0.07	13.7	13.4	0.01
Opioids	7.8	6.9	0.04	6.5	7	-0.02
Psycholeptics	12.4	11.5	0.03	6.6	6.8	-0.01
Psychostimulants, agents used for ADHD and nootropics	1	0.8	0.03	0.8	1.1	-0.04

Table S6.96. Selected baseline characteristics for Italy IQVIA, for the long-term risk of depression

Characteristic	Before propensity score matching			After propensity score matching		
	Targets, %	Comparators, %	Standardized difference	Targets, %	Comparators, %	Standardized difference
**Age group (years)**						
10-14	0.5	0.5	-0.01	0.5	0.8	-0.03
15-19	2.8	3.9	-0.06	3.2	3.7	-0.03
20-24	4.8	4.8	0	4.3	5.1	-0.04
25-29	5.6	4.9	0.03	5.3	5.3	0
30-34	5.9	5.1	0.04	5.8	5.9	0
35-39	6.5	5.5	0.04	5.7	6.1	-0.02
40-44	8	6.7	0.05	7.7	8.1	-0.02
45-49	10.4	8.3	0.07	10.5	10.9	-0.01
50-54	10.7	9.1	0.05	11.2	11.6	-0.01
55-59	11.7	9.6	0.07	11.7	10.8	0.03
60-64	8.6	8.9	-0.01	9.8	9.3	0.02
65-69	6.2	8.1	-0.07	7	7.3	-0.01
70-74	5.8	8.1	-0.09	6.2	5.4	0.04
75-79	4	6	-0.09	4.3	3.6	0.03
80-84	4	5.5	-0.07	3.6	3.5	0
85-89	2.7	3.3	-0.04	2.1	1.8	0.02
90-94	1.4	1.5	0	0.8	0.8	0
95-99	0.4	0.2	0.03	0.1	0.1	0
**Sex**						
Female	51.9	54.5	-0.05	51.9	52.6	-0.01

Table S6.96. *Continued.* Selected baseline characteristics for Italy IQVIA, for the long-term risk of depression

**Characteristic**	**Before propensity score matching**			**After propensity score matching**		
	**Targets, %**	**Comparators, %**	**Standardized difference**	**Targets, %**	**Comparators, %**	**Standardized difference**
**Medical history (general)**						
Acute respiratory disease	24	13.3	0.28	25.1	29.9	-0.11
Chronic liver disease	1.9	1.8	0	2	2	0
Chronic obstructive lung disease	1.8	1.7	0.01	1.9	1.6	0.02
Crohn’s disease	0.3	0.2	0.01	0.2	0.4	-0.02
Dementia	0.8	0.7	0.02	0.4	0.4	0
Diabetes mellitus	7.7	8.5	-0.03	7.6	7.2	0.01
Gastroesophageal reflux disease	9	7.6	0.05	8.3	9.1	-0.03
Gastrointestinal hemorrhage	0.3	0.2	0	0.3	0.3	0
Human immunodeficiency virus infection	0.1	0.1	0.02	0.1	0.1	0.02
Hyperlipidemia	10.8	11.4	-0.02	10.9	11	0
Hypertensive disorder	25.4	27.2	-0.04	26.3	25	0.03
Lesion of liver	0.3	0.3	-0.01	0.3	0.3	0
Obesity	0.5	0.3	0.03	0.4	0.4	0
Osteoarthritis	7.7	8.1	-0.01	7.1	7.9	-0.03
Pneumonia	9.7	0.9	0.4	4.3	3.5	0.04
Renal impairment	2.1	1.9	0.02	1.9	1.9	0
Rheumatoid arthritis	0.6	0.6	-0.01	0.5	0.6	0
Ulcerative colitis	0.4	0.3	0.02	0.5	0.4	0.02
Urinary tract infectious disease	2.8	2.1	0.05	2.5	2.8	-0.02
Viral hepatitis C	0.4	0.4	0.01	0.4	0.4	0

Table S6.96. *Continued.* Selected baseline characteristics for Italy IQVIA, for the long-term risk of depression

**Characteristic**	**Before propensity score matching**			**After propensity score matching**		
	**Targets, %**	**Comparators, %**	**Standardized difference**	**Targets, %**	**Comparators, %**	**Standardized difference**
**Medical history (cardiovascular disease)**						
Atrial fibrillation	3	3	0	2.6	2.7	0
Cerebrovascular disease	3.8	4	-0.01	3.2	2.8	0.02
Coronary arteriosclerosis	0.4	0.3	0.02	0.4	0.4	0
Heart disease	12.3	12.1	0	11.9	11.8	0
Heart failure	1.2	1.2	0	0.9	0.9	0
Ischemic heart disease	3.8	3.7	0	3.5	3.4	0
Peripheral vascular disease	0.5	0.6	-0.01	0.4	0.5	-0.01
Pulmonary embolism	0.3	0.2	0.02	0.2	0.2	-0.01
Venous thrombosis	0.5	0.5	0.01	0.5	0.6	-0.01
**Medical history (neoplasms)**						
Malignant lymphoma	0.4	0.3	0	0.3	0.5	-0.03
Malignant neoplasm of anorectum	0.1	0.2	0	0.2	0.2	0
Malignant neoplastic disease	6.2	6.1	0	6.1	5.8	0.01
Malignant tumor of breast	1.4	1.5	-0.01	1.4	1.2	0.01
Malignant tumor of colon	0.4	0.5	-0.01	0.3	0.4	-0.01
Malignant tumor of urinary bladder	0.4	0.4	0	0.4	0.3	0.01
Primary malignant neoplasm of prostate	0.8	0.7	0	0.8	0.6	0.01
**Medication use**						
Agents acting on the renin-angiotensin system	20.6	23.5	-0.07	21.9	20.8	0.03
Antibacterials for systemic use	48.3	32.1	0.33	40.7	44.8	-0.08
Antidepressants	10.7	9.8	0.03	5.7	5.9	-0.01
Antiepileptics	4.6	4.4	0.01	3.8	4	-0.01
Antiinflammatory and antirheumatic products	29.3	28.9	0.01	30.1	31.8	-0.04
Antineoplastic agents	2.1	2.2	-0.01	2	2.1	0
Antithrombotic agents	18.6	16.4	0.06	15	14.4	0.02

Table S6.96. *Continued.* Selected baseline characteristics for Italy IQVIA, for the long-term risk of depression

**Characteristic**	**Before propensity score matching**			**After propensity score matching**		
	**Targets, %**	**Comparators, %**	**Standardized difference**	**Targets, %**	**Comparators, %**	**Standardized difference**
**Medication use**						
Beta blocking agents	14.6	16	-0.04	14	13.8	0.01
Calcium channel blockers	9.4	10.6	-0.04	9.6	8.9	0.03
Diuretics	14.1	16.2	-0.06	14.3	13.3	0.03
Drugs for acid-related disorders	26.1	24.2	0.04	24.1	24.8	-0.02
Drugs for obstructive airway diseases	19.6	15.9	0.1	19.2	21.6	-0.06
Drugs used in diabetes	6.7	7.7	-0.04	6.7	6.4	0.01
Immunosuppressants	0.9	0.8	0.01	1	0.9	0.01
Lipid modifying agents	14.2	16.8	-0.07	14.7	13.8	0.03
Opioids	7.8	6.8	0.04	6.7	7.8	-0.04
Psycholeptics	12.4	11.5	0.03	9.8	10.5	-0.02
Psychostimulants, agents used for ADHD and nootropics	1	0.8	0.03	0.8	1.2	-0.04

Table S6.97. Selected baseline characteristics for Italy IQVIA, for the long-term risk of anxiety disorders

Characteristic	Before propensity score matching			After propensity score matching		
	**Targets, %**	**Comparators, %**	**Standardized difference**	**Targets, %**	**Comparators, %**	**Standardized difference**
**Age group (years)**						
10-14	0.5	0.5	-0.01	0.5	0.7	-0.03
15-19	2.8	3.8	-0.05	3	3.3	-0.02
20-24	4.8	4.6	0.01	4	4.7	-0.04
30-34	5.9	5.1	0.04	5.4	5.6	-0.01
35-39	6.5	5.5	0.04	5.6	5.8	-0.01
40-44	8	6.6	0.05	7.6	7.8	-0.01
45-49	10.4	8.2	0.07	10.4	10.8	-0.01
50-54	10.7	9.2	0.05	11.2	11.5	-0.01
55-59	11.7	9.6	0.07	11.8	10.7	0.03
60-64	8.6	8.9	-0.01	9.6	9.7	0
65-69	6.2	8.2	-0.08	7.1	7.5	-0.01
70-74	5.8	8.2	-0.09	6.6	5.7	0.04
75-79	4	6	-0.09	4.5	4.1	0.02
80-84	4	5.5	-0.07	4.1	4	0.01
85-89	2.7	3.4	-0.04	2.5	2.1	0.03
90-94	1.4	1.5	0	0.9	0.9	0.01
95-99	0.4	0.2	0.03	0.1	0.1	0
**Sex**						
Female	51.9	54.5	-0.05	53.8	54.1	-0.01

Table S6.97. *Continued.* Selected baseline characteristics for Italy IQVIA, for the long-term risk of anxiety disorders

Characteristic	Before propensity score matching			After propensity score matching		
	Targets, %	Comparators, %	Standardized difference	Targets, %	Comparators, %	Standardized difference
**Medical history (general)**						
Acute respiratory disease	24	13.2	0.28	25.3	30	-0.11
Chronic liver disease	1.9	1.8	0.01	2.1	2.1	0
Chronic obstructive lung disease	1.8	1.8	0.01	1.9	1.7	0.02
Crohn’s disease	0.3	0.2	0.01	0.3	0.4	-0.01
Dementia	0.8	0.7	0.02	0.5	0.5	0
Depressive disorder	6.6	6.1	0.02	7	7.7	-0.02
Diabetes mellitus	7.7	8.4	-0.03	7.9	7.7	0
Gastroesophageal reflux disease	9	7.7	0.05	9.1	10.1	-0.04
Gastrointestinal hemorrhage	0.3	0.3	0	0.3	0.3	0
Human immunodeficiency virus infection	0.1	0.1	0.02	0.1	0.1	0.02
Hyperlipidemia	10.8	11.4	-0.02	11.7	11.7	0
Hypertensive disorder	25.4	27.3	-0.04	27.6	26.4	0.03
Lesion of liver	0.3	0.3	0	0.2	0.3	-0.01
Obesity	0.5	0.3	0.03	0.4	0.5	-0.01
Osteoarthritis	7.7	8.2	-0.02	7.7	8.8	-0.04
Pneumonia	9.7	0.9	0.4	4.3	3.7	0.03
Renal impairment	2.1	1.9	0.01	2	2.1	-0.01
Rheumatoid arthritis	0.6	0.7	-0.01	0.7	0.6	0
Schizophrenia	0.1	0.2	-0.02	0.2	0.2	0
Ulcerative colitis	0.4	0.3	0.02	0.5	0.4	0.02
Urinary tract infectious disease	2.8	2.1	0.04	2.8	3.1	-0.02
Viral hepatitis C	0.4	0.4	0	0.5	0.4	0.01

Table S6.97. *Continued.* Selected baseline characteristics for Italy IQVIA, for the long-term risk of anxiety disorders

Characteristic	Before propensity score matching			After propensity score matching		
	Targets, %	Comparators, %	Standardized difference	Targets, %	Comparators, %	Standardized difference
**Medical history (cardiovascular disease)**						
Atrial fibrillation	3	3	0	2.9	2.9	0
Cerebrovascular disease	3.8	4	-0.01	3.6	3.4	0.01
Coronary arteriosclerosis	0.4	0.3	0.01	0.4	0.4	0
Heart disease	12.3	12.3	0	12.7	12.5	0
Heart failure	1.2	1.2	0	1.1	1.1	0
Ischemic heart disease	3.8	3.8	0	3.8	3.7	0
Peripheral vascular disease	0.5	0.6	-0.01	0.5	0.5	0
Pulmonary embolism	0.3	0.2	0.02	0.3	0.3	0
Venous thrombosis	0.5	0.4	0.01	0.6	0.7	-0.01
**Medical history (neoplasms)**						
Malignant lymphoma	0.4	0.3	0.01	0.3	0.5	-0.03
Malignant neoplasm of anorectum	0.1	0.2	0	0.2	0.2	0
Malignant neoplastic disease	6.2	6.1	0	6.3	6.2	0.01
Malignant tumor of breast	1.4	1.5	-0.01	1.4	1.3	0.01
Malignant tumor of colon	0.4	0.5	-0.01	0.3	0.4	-0.01
Malignant tumor of urinary bladder	0.4	0.4	0	0.4	0.3	0.01
Primary malignant neoplasm of prostate	0.8	0.8	0	0.7	0.6	0.01
**Medication use**						
Agents acting on the renin-angiotensin system	20.6	23.6	-0.07	23	21.8	0.03
Antibacterials for systemic use	48.3	32.1	0.33	41.8	45.4	-0.07
Antidepressants	10.7	9.8	0.03	10.5	10.9	-0.01
Antiepileptics	4.6	4.4	0.01	4.7	5	-0.01
Antiinflammatory and antirheumatic products	29.3	29.1	0	31.4	32.9	-0.03
Antineoplastic agents	2.1	2.2	-0.01	2.2	2.3	-0.01
Antipsoriatics	1.3	1.3	0	1.3	1.3	0

Table S6.97. *Continued.* Selected baseline characteristics for Italy IQVIA, for the long-term risk of anxiety disorders

Characteristic	Before propensity score matching			After propensity score matching		
	Targets, %	Comparators, %	Standardized difference	Targets, %	Comparators, %	Standardized difference
**Medication use**						
Antithrombotic agents	18.6	16.5	0.06	16.6	15.6	0.03
Beta blocking agents	14.6	16.1	-0.04	15.2	14.8	0.01
Calcium channel blockers	9.4	10.6	-0.04	10.3	9.4	0.03
Diuretics	14.1	16.3	-0.06	15.2	14.2	0.03
Drugs for acid-related disorders	26.1	24.4	0.04	26.1	26.6	-0.01
Drugs for obstructive airway diseases	19.6	16	0.09	20.4	22	-0.04
Drugs used in diabetes	6.7	7.6	-0.04	6.8	6.8	0
Immunosuppressants	0.9	0.8	0.01	1	0.9	0.01
Lipid modifying agents	14.2	16.9	-0.08	15.4	14.7	0.02
Opioids	7.8	6.8	0.04	7.6	8.7	-0.04
Psycholeptics	12.4	11.5	0.03	12.7	13.3	-0.02
Psychostimulants, agents used for ADHD and nootropics	1	0.8	0.03	1	1.4	-0.03

Table S6.98. Selected baseline characteristics for Italy IQVIA, for the long-term risk of alcohol misuse or dependence

Characteristic	Before propensity score matching			After propensity score matching		
	Targets, %	Comparators, %	Standardized difference	Targets, %	Comparators, %	Standardized difference
**Age group (years)**						
10-14	0.5	0.5	-0.01	0.5	0.7	-0.04
15-19	2.8	3.8	-0.06	3	3.3	-0.02
20-24	4.8	4.6	0.01	3.9	4.7	-0.04
25-29	5.6	4.8	0.04	4.9	4.9	0
30-34	5.9	5	0.04	5.4	5.6	-0.01
35-39	6.5	5.6	0.04	5.6	5.9	-0.01
40-44	8	6.6	0.05	7.7	7.9	-0.01
45-49	10.4	8.2	0.07	10.4	10.8	-0.01
50-54	10.7	9.2	0.05	11.3	11.6	-0.01
55-59	11.7	9.5	0.07	11.9	10.8	0.04
60-64	8.6	8.9	-0.01	9.7	9.6	0
65-69	6.2	8.2	-0.08	7.2	7.4	-0.01
70-74	5.8	8.1	-0.09	6.5	5.7	0.03
75-79	4	6.1	-0.1	4.4	4.1	0.02
80-84	4	5.5	-0.07	4	3.9	0
85-89	2.7	3.4	-0.04	2.5	2.1	0.03
90-94	1.4	1.5	-0.01	0.9	0.9	0
95-99	0.4	0.2	0.03	0.1	0.2	0
**Sex**						
Female	51.9	54.5	-0.05	53.9	54.4	-0.01

Table S6.98. *Continued.* Selected baseline characteristics for Italy IQVIA, for the long-term risk of alcohol misuse or dependence

Characteristic	Before propensity score matching			After propensity score matching		
	Targets, %	Comparators, %	Standardized difference	Targets, %	Comparators, %	Standardized difference
**Medical history (general)**						
Acute respiratory disease	24	13.3	0.28	25.4	30.2	-0.11
Chronic liver disease	1.9	1.8	0.01	2.1	2.1	0
Chronic obstructive lung disease	1.8	1.8	0	1.9	1.7	0.02
Crohn’s disease	0.3	0.2	0.01	0.3	0.4	-0.02
Dementia	0.8	0.7	0.02	0.4	0.5	0
Depressive disorder	6.6	6.1	0.02	7.1	7.6	-0.02
Diabetes mellitus	7.7	8.5	-0.03	7.8	7.7	0
Gastroesophageal reflux disease	9	7.7	0.05	9	10.2	-0.04
Gastrointestinal hemorrhage	0.3	0.2	0.01	0.3	0.3	0
Human immunodeficiency virus infection	0.1	0.1	0.02	0.1	0.1	0.02
Hyperlipidemia	10.8	11.4	-0.02	11.7	11.7	0
Hypertensive disorder	25.4	27.3	-0.04	27.7	26.3	0.03
Lesion of liver	0.3	0.3	-0.01	0.2	0.3	-0.02
Obesity	0.5	0.3	0.03	0.5	0.5	0
Osteoarthritis	7.7	8.2	-0.02	7.7	8.8	-0.04
Pneumonia	9.7	0.9	0.4	4.3	3.7	0.03
Renal impairment	2.1	1.9	0.02	2	2.1	-0.01
Rheumatoid arthritis	0.6	0.7	-0.01	0.7	0.6	0
Schizophrenia	0.1	0.2	-0.02	0.2	0.2	0
Ulcerative colitis	0.4	0.3	0.02	0.5	0.4	0.01
Urinary tract infectious disease	2.8	2.1	0.04	2.8	3.1	-0.02

Table S6.98. *Continued.* Selected baseline characteristics for Italy IQVIA, for the long-term risk of alcohol misuse or dependence

Characteristic	Before propensity score matching			After propensity score matching		
	**Targets, %**	**Comparators, %**	**Standardized difference**	**Targets, %**	**Comparators, %**	**Standardized difference**
**Medical history (cardiovascular disease)**						
Atrial fibrillation	3	3	0	2.8	2.9	-0.01
Cerebrovascular disease	3.8	4	-0.01	3.6	3.3	0.01
Coronary arteriosclerosis	0.4	0.3	0.02	0.3	0.4	0
Heart disease	12.3	12.3	0	12.6	12.5	0
Heart failure	1.2	1.2	0	1	1.1	0
Ischemic heart disease	3.8	3.8	0	3.8	3.7	0
Peripheral vascular disease	0.5	0.6	-0.01	0.5	0.5	0
Pulmonary embolism	0.3	0.2	0.02	0.3	0.3	0
Venous thrombosis	0.5	0.5	0.01	0.6	0.7	-0.01
**Medical history (neoplasms)**						
Malignant lymphoma	0.4	0.3	0	0.3	0.5	-0.03
Malignant neoplasm of anorectum	0.1	0.2	0	0.2	0.2	0
Malignant neoplastic disease	6.2	6.1	0	6.3	6.1	0.01
Malignant tumor of breast	1.4	1.5	-0.01	1.5	1.3	0.01
Malignant tumor of colon	0.4	0.5	-0.01	0.3	0.4	-0.01
Malignant tumor of urinary bladder	0.4	0.4	0	0.4	0.3	0.01
Primary malignant neoplasm of prostate	0.8	0.7	0	0.8	0.6	0.01
**Medication use**						
Agents acting on the renin-angiotensin system	20.6	23.7	-0.07	23	21.8	0.03
Antibacterials for systemic use	48.3	32	0.34	41.9	45.5	-0.07
Antidepressants	10.7	9.8	0.03	11.1	11.5	-0.01
Antiepileptics	4.6	4.5	0.01	4.8	5.1	-0.01
Antiinflammatory and antirheumatic products	29.3	29.1	0.01	31.5	33	-0.03
Antineoplastic agents	2.1	2.2	-0.01	2.1	2.3	-0.01
Antipsoriatics	1.3	1.3	0.01	1.3	1.3	0

Table S6.98. *Continued.* Selected baseline characteristics for Italy IQVIA, for the long-term risk of alcohol misuse or dependence

**Characteristic**	**Before propensity score matching**			**After propensity score matching**		
	**Targets, %**	**Comparators, %**	**Standardized difference**	**Targets, %**	**Comparators, %**	**Standardized difference**
**Medication use**						
Antithrombotic agents	18.6	16.5	0.05	16.4	15.5	0.03
Beta blocking agents	14.6	16.1	-0.04	15.2	14.8	0.01
Calcium channel blockers	9.4	10.6	-0.04	10.3	9.4	0.03
Diuretics	14.1	16.4	-0.06	15.1	14.2	0.03
Drugs for acid-related disorders	26.1	24.3	0.04	26	26.7	-0.02
Drugs for obstructive airway diseases	19.6	15.9	0.1	20.3	22.1	-0.04
Drugs used in diabetes	6.7	7.7	-0.04	6.8	6.7	0
Immunosuppressants	0.9	0.8	0.01	1	0.9	0
Lipid modifying agents	14.2	16.9	-0.07	15.4	14.7	0.02
Opioids	7.8	6.9	0.04	7.7	8.6	-0.04
Psycholeptics	12.4	11.5	0.03	13	13.9	-0.02
Psychostimulants, agents used for ADHD and nootropics	1	0.8	0.02	1.1	1.4	-0.03

Table S6.99. Selected baseline characteristics for Italy IQVIA, for the long-term risk of substance misuse or dependence

Characteristic	Before propensity score matching			After propensity score matching		
	Targets, %	Comparators, %	Standardized difference	Targets, %	Comparators, %	Standardized difference
**Age group (years)**						
10-14	0.5	0.5	-0.01	0.5	0.7	-0.04
15-19	2.8	3.9	-0.06	3	3.4	-0.02
25-29	5.6	4.9	0.03	4.8	4.9	0
30-34	5.9	5.2	0.03	5.4	5.6	-0.01
35-39	6.5	5.5	0.04	5.6	5.9	-0.01
40-44	8	6.6	0.05	7.7	7.9	-0.01
45-49	10.4	8.2	0.08	10.4	10.7	-0.01
50-54	10.7	9.2	0.05	11.3	11.6	-0.01
55-59	11.7	9.6	0.07	11.9	10.8	0.04
60-64	8.6	8.8	-0.01	9.7	9.6	0
65-69	6.2	8.1	-0.07	7.2	7.4	-0.01
70-74	5.8	8.2	-0.09	6.5	5.7	0.03
75-79	4	6	-0.09	4.4	4.1	0.02
80-84	4	5.5	-0.07	4	3.9	0
85-89	2.7	3.3	-0.04	2.5	2.1	0.03
90-94	1.4	1.5	-0.01	0.9	0.9	0
95-99	0.4	0.2	0.03	0.1	0.2	-0.01
**Sex**						
Female	51.9	54.4	-0.05	54	54.5	-0.01

Table S6.99. *Continued.* Selected baseline characteristics for Italy IQVIA, for the long-term risk of substance misuse or dependence

Characteristic	Before propensity score matching			After propensity score matching		
	Targets, %	Comparators, %	Standardized difference	Targets, %	Comparators, %	Standardized difference
**Medical history (general)**						
Acute respiratory disease	24	13.2	0.28	25.4	30.2	-0.11
Chronic liver disease	1.9	1.8	0.01	2.1	2.1	0
Chronic obstructive lung disease	1.8	1.8	0	1.9	1.7	0.02
Crohn's disease	0.3	0.2	0.01	0.3	0.4	-0.02
Dementia	0.8	0.7	0.02	0.4	0.5	0
Depressive disorder	6.6	6.2	0.02	7.1	7.6	-0.02
Diabetes mellitus	7.7	8.5	-0.03	7.8	7.6	0.01
Gastroesophageal reflux disease	9	7.6	0.05	9.1	10.2	-0.04
Gastrointestinal hemorrhage	0.3	0.3	0	0.3	0.3	0
Human immunodeficiency virus infection	0.1	0.1	0.02	0.1	0.1	0.02
Hyperlipidemia	10.8	11.4	-0.02	11.8	11.7	0
Hypertensive disorder	25.4	27.2	-0.04	27.7	26.3	0.03
Lesion of liver	0.3	0.3	-0.01	0.2	0.3	-0.02
Obesity	0.5	0.3	0.03	0.5	0.5	0
Osteoarthritis	7.7	8.2	-0.02	7.7	8.8	-0.04
Pneumonia	9.7	0.9	0.4	4.3	3.7	0.03
Renal impairment	2.1	1.9	0.02	2	2.1	-0.01
Rheumatoid arthritis	0.6	0.6	-0.01	0.7	0.6	0.01
Schizophrenia	0.1	0.2	-0.02	0.2	0.2	0
Ulcerative colitis	0.4	0.3	0.02	0.5	0.4	0.01
Urinary tract infectious disease	2.8	2.1	0.04	2.8	3.1	-0.02
Viral hepatitis C	0.4	0.4	0	0.4	0.4	0

Table S6.99. *Continued.* Selected baseline characteristics for Italy IQVIA, for the long-term risk of substance misuse or dependence

Characteristic	Before propensity score matching			After propensity score matching		
	Targets, %	Comparators, %	Standardized difference	Targets, %	Comparators, %	Standardized difference
**Medical history (cardiovascular disease)**						
Cerebrovascular disease	3.8	3.9	-0.01	3.6	3.3	0.02
Coronary arteriosclerosis	0.4	0.3	0.02	0.3	0.4	0
Heart disease	12.3	12.2	0	12.6	12.5	0
Heart failure	1.2	1.2	0.01	1	1.1	0
Ischemic heart disease	3.8	3.8	0	3.8	3.7	0
Peripheral vascular disease	0.5	0.6	-0.01	0.5	0.5	0
Pulmonary embolism	0.3	0.2	0.02	0.3	0.3	0
Venous thrombosis	0.5	0.5	0.01	0.6	0.7	-0.01
**Medical history (neoplasms)**						
Malignant lymphoma	0.4	0.3	0.01	0.3	0.5	-0.03
Malignant neoplasm of anorectum	0.1	0.2	0	0.2	0.2	0
Malignant neoplastic disease	6.2	6.2	0	6.3	6.1	0.01
Malignant tumor of breast	1.4	1.5	-0.01	1.5	1.3	0.01
Malignant tumor of colon	0.4	0.5	-0.01	0.3	0.4	-0.01
Malignant tumor of urinary bladder	0.4	0.4	0	0.4	0.3	0.01
Primary malignant neoplasm of prostate	0.8	0.7	0	0.8	0.6	0.01
**Medication use**						
Agents acting on the renin-angiotensin system	20.6	23.6	-0.07	23	21.7	0.03
Antibacterials for systemic use	48.3	32.1	0.33	41.9	45.5	-0.07
Antidepressants	10.7	9.8	0.03	11.1	11.4	-0.01
Antiepileptics	4.6	4.4	0.01	4.8	5	-0.01
Antiinflammatory and antirheumatic products	29.3	29	0.01	31.5	33	-0.03
Antineoplastic agents	2.1	2.3	-0.01	2.2	2.3	-0.01
Antipsoriatics	1.3	1.3	0	1.3	1.3	0
Antithrombotic agents	18.6	16.5	0.06	16.4	15.4	0.03

Table S6.99. *Continued.* Selected baseline characteristics for Italy IQVIA, for the long-term risk of substance misuse or dependence

**Characteristic**	**Before propensity score matching**			**After propensity score matching**		
	**Targets, %**	**Comparators, %**	**Standardized difference**	**Targets, %**	**Comparators, %**	**Standardized difference**
**Medication use**						
Beta blocking agents	14.6	16.1	-0.04	15.2	14.9	0.01
Calcium channel blockers	9.4	10.5	-0.04	10.3	9.4	0.03
Diuretics	14.1	16.2	-0.06	15.1	14.2	0.03
Drugs for acid-related disorders	26.1	24.3	0.04	26.1	26.7	-0.01
Drugs for obstructive airway diseases	19.6	16.1	0.09	20.3	22.1	-0.04
Drugs used in diabetes	6.7	7.7	-0.04	6.8	6.7	0
Immunosuppressants	0.9	0.8	0.01	1	0.9	0
Lipid modifying agents	14.2	17	-0.08	15.4	14.7	0.02
Opioids	7.8	6.9	0.04	7.7	8.6	-0.04
Psycholeptics	12.4	11.6	0.03	13	13.8	-0.02
Psychostimulants, agents used for ADHD and nootropics	1	0.8	0.02	1.1	1.4	-0.03

Table S6.100. Selected baseline characteristics for Italy IQVIA, for the long-term risk of bipolar disorders

Characteristic	Before propensity score matching			After propensity score matching		
	Targets, %	Comparators, %	Standardized difference	Targets, %	Comparators, %	Standardized difference
**Age group (years)**						
10-14	0.5	0.5	-0.01	0.5	0.7	-0.04
15-19	2.8	3.9	-0.06	3	3.3	-0.02
20-24	4.8	4.7	0	4	4.7	-0.04
25-29	5.6	4.9	0.03	4.9	4.9	0
30-34	5.9	5	0.04	5.4	5.6	-0.01
35-39	6.5	5.6	0.04	5.6	5.9	-0.01
40-44	8	6.7	0.05	7.7	7.9	-0.01
45-49	10.4	8.2	0.08	10.4	10.7	-0.01
50-54	10.7	9.3	0.05	11.3	11.6	-0.01
55-59	11.7	9.5	0.07	11.9	10.7	0.04
60-64	8.6	8.7	-0.01	9.7	9.6	0
65-69	6.2	8.1	-0.08	7.1	7.4	-0.01
70-74	5.8	8.2	-0.1	6.5	5.7	0.04
75-79	4	6	-0.1	4.4	4.1	0.02
80-84	4	5.4	-0.07	4	3.9	0
85-89	2.7	3.3	-0.04	2.5	2.1	0.03
90-94	1.4	1.5	-0.01	0.9	0.9	0
95-99	0.4	0.2	0.03	0.1	0.2	0
**Sex**						
Female	51.9	54.5	-0.05	53.9	54.3	-0.01

Table S6.100. *Continued.* Selected baseline characteristics for Italy IQVIA, for the long-term risk of bipolar disorders

Characteristic	Before propensity score matching			After propensity score matching		
	Targets, %	Comparators, %	Standardized difference	Targets, %	Comparators, %	Standardized difference
**Medical history (general)**						
Acute respiratory disease	24	13.4	0.28	25.5	30.2	-0.1
Chronic liver disease	1.9	1.8	0.01	2.1	2.2	0
Chronic obstructive lung disease	1.8	1.8	0.01	1.9	1.7	0.02
Crohn's disease	0.3	0.2	0.01	0.3	0.4	-0.02
Dementia	0.8	0.7	0.01	0.4	0.5	0
Depressive disorder	6.6	6.1	0.02	7	7.5	-0.02
Diabetes mellitus	7.7	8.6	-0.03	7.9	7.6	0.01
Gastroesophageal reflux disease	9	7.6	0.05	9.1	10.2	-0.04
Human immunodeficiency virus infection	0.1	0.1	0.02	0.1	0.1	0.02
Hypertensive disorder	25.4	27.1	-0.04	27.8	26.3	0.03
Lesion of liver	0.3	0.3	-0.01	0.2	0.3	-0.02
Obesity	0.5	0.3	0.03	0.5	0.4	0
Osteoarthritis	7.7	8.2	-0.02	7.8	8.8	-0.04
Pneumonia	9.7	0.9	0.4	4.3	3.8	0.03
Psoriasis	1.7	1.4	0.02	1.8	1.8	0
Renal impairment	2.1	1.9	0.01	2	2.1	0
Rheumatoid arthritis	0.6	0.7	-0.01	0.7	0.6	0
Schizophrenia	0.1	0.2	-0.02	0.1	0.2	-0.01
Ulcerative colitis	0.4	0.3	0.02	0.5	0.4	0.01
Urinary tract infectious disease	2.8	2.1	0.04	2.8	3.1	-0.02

Table S6.100. *Continued.* Selected baseline characteristics for Italy IQVIA, for the long-term risk of bipolar disorders

**Characteristic**	**Before propensity score matching**			**After propensity score matching**		
	**Targets, %**	**Comparators, %**	**Standardized difference**	**Targets, %**	**Comparators, %**	**Standardized difference**
Medical history: Cardiovascular disease						
Atrial fibrillation	3	3	0	2.8	2.9	0
Cerebrovascular disease	3.8	3.9	-0.01	3.6	3.4	0.01
Coronary arteriosclerosis	0.4	0.3	0.01	0.3	0.4	0
Heart disease	12.3	12.2	0	12.6	12.5	0
Heart failure	1.2	1.2	0	1	1.1	0
Ischemic heart disease	3.8	3.8	0	3.7	3.7	0
Peripheral vascular disease	0.5	0.6	-0.01	0.5	0.5	0
Pulmonary embolism	0.3	0.2	0.02	0.3	0.3	0
Venous thrombosis	0.5	0.5	0.01	0.6	0.7	-0.01
**Medical history (neoplasms)**						
Malignant lymphoma	0.4	0.3	0	0.3	0.5	-0.03
Malignant neoplasm of anorectum	0.1	0.2	0	0.2	0.2	0
Malignant neoplastic disease	6.2	6.1	0	6.4	6.1	0.01
Malignant tumor of breast	1.4	1.5	-0.01	1.5	1.3	0.01
Malignant tumor of colon	0.4	0.5	-0.01	0.3	0.4	-0.01
Malignant tumor of urinary bladder	0.4	0.4	0	0.4	0.3	0.01
Primary malignant neoplasm of prostate	0.8	0.8	0	0.8	0.7	0.01
**Medication use**						
Agents acting on the renin-angiotensin system	20.6	23.5	-0.07	22.9	21.7	0.03
Antibacterials for systemic use	48.3	32.2	0.33	41.8	45.5	-0.07
Antidepressants	10.7	9.7	0.03	11	11.3	-0.01
Antiepileptics	4.6	4.4	0.01	4.6	4.8	-0.01
Antiinflammatory and antirheumatic products	29.3	28.9	0.01	31.4	33.1	-0.04
Antineoplastic agents	2.1	2.2	-0.01	2.2	2.3	-0.01
Antipsoriatics	1.3	1.3	0	1.3	1.3	0

Table S6.100. *Continued.* Selected baseline characteristics for Italy IQVIA, for the long-term risk of bipolar disorders

	**Before propensity score matching**			**After propensity score matching**		
	**Targets, %**	**Comparators, %**	**Standardized difference**	**Targets, %**	**Comparators, %**	**Standardized difference**
**Medication use**						
Antithrombotic agents	18.6	16.5	0.06	16.4	15.5	0.03
Beta blocking agents	14.6	16	-0.04	15.2	14.8	0.01
Calcium channel blockers	9.4	10.6	-0.04	10.3	9.4	0.03
Diuretics	14.1	16.2	-0.06	15.2	14.2	0.03
Drugs for acid-related disorders	26.1	24.2	0.04	26	26.7	-0.01
Drugs for obstructive airway diseases	19.6	16	0.1	20.2	22.1	-0.05
Drugs used in diabetes	6.7	7.8	-0.04	6.8	6.7	0
Immunosuppressants	0.9	0.8	0.01	1	1	0
Lipid modifying agents	14.2	16.7	-0.07	15.3	14.7	0.02
Opioids	7.8	6.7	0.04	7.6	8.6	-0.04
Psycholeptics	12.4	11.4	0.03	12.9	13.7	-0.02
Psychostimulants, agents used for ADHD and nootropics	1	0.8	0.03	1.1	1.4	-0.03

Table S6.101. Selected baseline characteristics for Italy IQVIA, for the long-term risk of psychoses

Characteristic	Before propensity score matching			After propensity score matching		
	Targets, %	Comparators, %	Standardized difference	Targets, %	Comparators, %	Standardized difference
**Age group (years)**						
10-14	0.5	0.6	-0.01	0.5	0.7	-0.04
15-19	2.8	3.9	-0.06	3	3.3	-0.02
20-24	4.8	4.7	0.01	3.9	4.7	-0.04
25-29	5.6	4.7	0.04	4.8	4.9	0
30-34	5.9	5.1	0.04	5.4	5.6	-0.01
35-39	6.5	5.6	0.04	5.6	5.9	-0.01
40-44	8	6.8	0.05	7.8	7.9	-0.01
45-49	10.4	8.2	0.07	10.4	10.8	-0.01
50-54	10.7	9.1	0.05	11.3	11.6	-0.01
55-59	11.7	9.5	0.07	11.9	10.8	0.04
60-64	8.6	8.9	-0.01	9.7	9.6	0
65-69	6.2	8.1	-0.07	7.1	7.4	-0.01
70-74	5.8	8.2	-0.1	6.5	5.7	0.04
75-79	4	6	-0.09	4.5	4	0.02
80-84	4	5.4	-0.07	4	3.9	0.01
85-89	2.7	3.4	-0.04	2.5	2	0.03
90-94	1.4	1.5	-0.01	0.9	0.9	0
95-99	0.4	0.2	0.03	0.1	0.1	-0.01
**Sex**						
Female	51.9	54.3	-0.05	53.8	54.4	-0.01

Table S6.101. *Continued.* Selected baseline characteristics for Italy IQVIA, for the long-term risk of psychoses

Characteristic	Before propensity score matching			After propensity score matching		
	Targets, %	Comparators, %	Standardized difference	Targets, %	Comparators, %	Standardized difference
**Medical history (general)**						
Acute respiratory disease	24	13.3	0.28	25.5	30.2	-0.1
Chronic liver disease	1.9	1.8	0	2.1	2.1	0
Chronic obstructive lung disease	1.8	1.7	0.01	1.9	1.7	0.02
Crohn’s disease	0.3	0.2	0.01	0.3	0.3	-0.01
Dementia	0.8	0.7	0.02	0.4	0.4	0
Depressive disorder	6.6	6.2	0.02	7.1	7.6	-0.02
Diabetes mellitus	7.7	8.5	-0.03	7.8	7.6	0
Gastroesophageal reflux disease	9	7.6	0.05	9.1	10.2	-0.04
Gastrointestinal hemorrhage	0.3	0.3	0	0.3	0.3	0
Human immunodeficiency virus infection	0.1	0.1	0.02	0.1	0.1	0.02
Hyperlipidemia	10.8	11.4	-0.02	11.8	11.7	0
Hypertensive disorder	25.4	27.2	-0.04	27.7	26.3	0.03
Lesion of liver	0.3	0.3	-0.01	0.2	0.3	-0.01
Obesity	0.5	0.3	0.03	0.5	0.4	0
Osteoarthritis	7.7	8.2	-0.02	7.8	8.8	-0.04
Pneumonia	9.7	0.9	0.4	4.3	3.7	0.03
Psoriasis	1.7	1.4	0.02	1.8	1.8	0
Renal impairment	2.1	1.9	0.01	2	2.1	0
Rheumatoid arthritis	0.6	0.6	-0.01	0.7	0.6	0
Ulcerative colitis	0.4	0.3	0.01	0.5	0.4	0.02
Urinary tract infectious disease	2.8	2.1	0.04	2.8	3.1	-0.02
Viral hepatitis C	0.4	0.4	0.01	0.4	0.4	0.01

Table S6.101. *Continued.* Selected baseline characteristics for Italy IQVIA, for the long-term risk of psychoses

	**Before propensity score matching**			**After propensity score matching**		
	**Targets, %**	**Comparators, %**	**Standardized difference**	**Targets, %**	**Comparators, %**	**Standardized difference**
**Medical history (cardiovascular disease)**						
Atrial fibrillation	3	3	0	2.8	2.9	-0.01
Cerebrovascular disease	3.8	4	-0.01	3.6	3.3	0.01
Coronary arteriosclerosis	0.4	0.3	0.01	0.4	0.4	0
Heart disease	12.3	12.2	0	12.5	12.5	0
Heart failure	1.2	1.2	0	1	1.1	-0.01
Ischemic heart disease	3.8	3.8	0	3.7	3.7	0
Peripheral vascular disease	0.5	0.6	-0.01	0.5	0.5	0
Pulmonary embolism	0.3	0.2	0.02	0.3	0.3	0
Venous thrombosis	0.5	0.5	0.01	0.6	0.6	-0.01
**Medical history (neoplasms)**						
Malignant lymphoma	0.4	0.3	0	0.3	0.5	-0.03
Malignant neoplasm of anorectum	0.1	0.2	0	0.2	0.2	0
Malignant tumor of breast	1.4	1.5	-0.01	1.5	1.3	0.01
Malignant tumor of colon	0.4	0.5	-0.01	0.3	0.4	-0.01
Malignant tumor of urinary bladder	0.4	0.4	0	0.4	0.3	0.01
Primary malignant neoplasm of prostate	0.8	0.8	0	0.7	0.6	0.01
**Medication use**						
Agents acting on the renin-angiotensin system	20.6	23.7	-0.07	23	21.7	0.03
Antibacterials for systemic use	48.3	32	0.34	41.9	45.5	-0.07
Antidepressants	10.7	9.9	0.03	11	11.4	-0.01
Antiepileptics	4.6	4.4	0.01	4.6	4.9	-0.01
Antiinflammatory and antirheumatic products	29.3	28.9	0.01	31.5	33.1	-0.03
Antineoplastic agents	2.1	2.2	-0.01	2.2	2.3	-0.01
Antipsoriatics	1.3	1.3	0	1.3	1.3	0
Antithrombotic agents	18.6	16.6	0.05	16.4	15.4	0.03

Table S6.101. *Continued.* Selected baseline characteristics for Italy IQVIA, for the long-term risk of psychoses

**Characteristic**	**Before propensity score matching**			**After propensity score matching**		
	**Targets, %**	**Comparators, %**	**Standardized difference**	**Targets, %**	**Comparators, %**	**Standardized difference**
**Medication use**						
Beta blocking agents	14.6	16.1	-0.04	15	14.9	0
Calcium channel blockers	9.4	10.6	-0.04	10.3	9.3	0.03
Diuretics	14.1	16.4	-0.06	15.1	14.2	0.03
Drugs for acid-related disorders	26.1	24.3	0.04	26.1	26.6	-0.01
Drugs for obstructive airway diseases	19.6	16	0.09	20.4	22.1	-0.04
Drugs used in diabetes	6.7	7.7	-0.04	6.8	6.7	0
Immunosuppressants	0.9	0.8	0	1	0.9	0
Lipid modifying agents	14.2	16.9	-0.08	15.4	14.7	0.02
Opioids	7.8	6.9	0.04	7.7	8.7	-0.03
Psycholeptics	12.4	11.5	0.03	12.7	13.6	-0.03
Psychostimulants, agents used for ADHD and nootropics	1	0.8	0.02	1.1	1.4	-0.03

Table S6.102. Selected baseline characteristics for Italy IQVIA, for the long-term risk of personality disorders

Characteristic	Before propensity score matching			After propensity score matching		
	Targets, %	Comparators, %	Standardized difference	Targets, %	Comparators, %	Standardized difference
**Age group (years)**						
10-14	0.5	0.5	-0.01	0.4	0.7	-0.04
15-19	2.8	3.8	-0.06	3	3.4	-0.02
20-24	4.8	4.7	0	4	4.7	-0.04
25-29	5.6	4.8	0.03	4.9	5	0
30-34	5.9	5.1	0.04	5.5	5.7	-0.01
35-39	6.5	5.6	0.04	5.7	5.9	-0.01
40-44	8	6.6	0.05	7.8	8	-0.01
45-49	10.4	8.3	0.07	10.6	10.9	-0.01
50-54	10.7	9.4	0.04	11.4	11.7	-0.01
55-59	11.7	9.5	0.07	12	10.8	0.04
60-64	8.6	8.8	-0.01	9.8	9.7	0.01
65-69	6.2	8	-0.07	7.2	7.4	-0.01
70-74	5.8	8.2	-0.09	6.5	5.6	0.04
75-79	4	6.1	-0.1	4.3	3.8	0.02
80-84	4	5.5	-0.07	3.6	3.6	0
85-89	2.7	3.3	-0.04	2.2	1.9	0.02
90-94	1.4	1.5	0	0.8	0.8	0
95-99	0.4	0.2	0.03	0.1	0.1	0
**Sex**						
Female	51.9	54.5	-0.05	53.6	54.1	-0.01

Table S6.102. *Continued.* Selected baseline characteristics for Italy IQVIA, for the long-term risk of personality disorders

Characteristic	Before propensity score matching			After propensity score matching		
	Targets, %	Comparators, %	Standardized difference	Targets, %	Comparators, %	Standardized difference
**Medical history (general)**						
Acute respiratory disease	24	13.2	0.28	25.5	30.2	-0.1
Chronic liver disease	1.9	1.8	0.01	2.1	2.1	0
Chronic obstructive lung disease	1.8	1.7	0.01	1.9	1.7	0.02
Crohn's disease	0.3	0.2	0.01	0.3	0.4	-0.01
Dementia	0.8	0.7	0.02	0.4	0.4	0
Depressive disorder	6.6	6.2	0.02	6.8	7.4	-0.03
Diabetes mellitus	7.7	8.5	-0.03	7.7	7.5	0.01
Gastroesophageal reflux disease	9	7.6	0.05	9	10.1	-0.04
Gastrointestinal hemorrhage	0.3	0.3	0	0.3	0.3	0
Human immunodeficiency virus infection	0.1	0.1	0.02	0.1	0.1	0.02
Hyperlipidemia	10.8	11.4	-0.02	11.7	11.5	0.01
Hypertensive disorder	25.4	27.1	-0.04	27.3	25.9	0.03
Lesion of liver	0.3	0.3	-0.01	0.2	0.3	-0.01
Obesity	0.5	0.3	0.03	0.4	0.5	0
Osteoarthritis	7.7	8.2	-0.02	7.6	8.5	-0.03
Pneumonia	9.7	0.9	0.4	4.2	3.7	0.03
Psoriasis	1.7	1.4	0.02	1.8	1.8	0
Renal impairment	2.1	1.9	0.02	1.9	2	-0.01
Rheumatoid arthritis	0.6	0.6	-0.01	0.6	0.6	0
Schizophrenia	0.1	0.2	-0.02	0.2	0.2	0
Ulcerative colitis	0.4	0.3	0.02	0.5	0.4	0.02
Urinary tract infectious disease	2.8	2.1	0.05	2.6	3.1	-0.03
Viral hepatitis C	0.4	0.4	0.01	0.4	0.4	0

Table S6.102. *Continued.* Selected baseline characteristics for Italy IQVIA, for the long-term risk of personality disorders

Characteristic	Before propensity score matching			After propensity score matching		
	Targets, %	Comparators, %	Standardized difference	Targets, %	Comparators, %	Standardized difference
**Medical history (cardiovascular disease)**						
Atrial fibrillation	3	3	0	2.6	2.8	-0.01
Cerebrovascular disease	3.8	3.9	-0.01	3.4	3.1	0.01
Coronary arteriosclerosis	0.4	0.3	0.01	0.3	0.4	-0.01
Heart disease	12.3	12.2	0	12.2	12.1	0
Heart failure	1.2	1.2	0	1	1	0
Ischemic heart disease	3.8	3.8	0	3.6	3.6	0
Peripheral vascular disease	0.5	0.6	-0.01	0.5	0.5	0
Pulmonary embolism	0.3	0.2	0.03	0.3	0.2	0
Venous thrombosis	0.5	0.5	0.01	0.5	0.6	-0.02
**Medical history (neoplasms)**						
Malignant lymphoma	0.4	0.3	0.01	0.3	0.5	-0.03
Malignant neoplasm of anorectum	0.1	0.2	0	0.2	0.2	0
Malignant tumor of breast	1.4	1.5	-0.01	1.5	1.4	0.01
Malignant tumor of colon	0.4	0.5	-0.01	0.3	0.4	-0.01
Malignant tumor of urinary bladder	0.4	0.4	0	0.4	0.3	0.01
Primary malignant neoplasm of prostate	0.8	0.8	0	0.7	0.6	0.01
**Medication use**						
Agents acting on the renin-angiotensin system	20.6	23.5	-0.07	22.5	21.4	0.03
Antibacterials for systemic use	48.3	32.2	0.33	41.8	45.4	-0.07
Antidepressants	10.7	9.8	0.03	10.6	11.1	-0.01
Antiepileptics	4.6	4.4	0.01	4.7	4.9	-0.01
Antiinflammatory and antirheumatic products	29.3	28.9	0.01	31.1	32.7	-0.03
Antineoplastic agents	2.1	2.2	-0.01	2	2.2	-0.01
Antipsoriatics	1.3	1.3	0.01	1.3	1.3	0
Antithrombotic agents	18.6	16.5	0.05	15.9	15	0.02

Table S6.102. *Continued.* Selected baseline characteristics for Italy IQVIA, for the long-term risk of personality disorders

Characteristic	Before propensity score matching			After propensity score matching		
	Targets, %	Comparators, %	Standardized difference	Targets, %	Comparators, %	Standardized difference
**Medication use**						
Beta blocking agents	14.6	16.1	-0.04	14.8	14.6	0
Calcium channel blockers	9.4	10.5	-0.04	10.1	9.1	0.03
Diuretics	14.1	16.3	-0.06	14.9	13.8	0.03
Drugs for acid-related disorders	26.1	24.4	0.04	25.6	26.3	-0.01
Drugs for obstructive airway diseases	19.6	16	0.1	20.2	22	-0.04
Drugs used in diabetes	6.7	7.7	-0.04	6.8	6.6	0
Immunosuppressants	0.9	0.8	0.01	0.9	0.9	0
Lipid modifying agents	14.2	16.8	-0.07	15.1	14.5	0.02
Opioids	7.8	6.9	0.04	7.5	8.4	-0.03
Psycholeptics	12.4	11.6	0.03	12.6	13.5	-0.03
Psychostimulants, agents used for ADHD and nootropics	1	0.8	0.02	1	1.3	-0.03

Table S6.103. Selected baseline characteristics for Italy IQVIA, for the long-term risk of self-harm and suicide

Characteristic	Before propensity score matching			After propensity score matching		
	**Targets, %**	**Comparators, %**	**Standardized difference**	**Targets, %**	**Comparators, %**	**Standardized difference**
**Age group (years)**						
10-14	0.5	0.5	-0.01	0.5	0.7	-0.03
15-19	2.8	3.8	-0.06	3	3.3	-0.02
20-24	4.8	4.7	0	3.9	4.7	-0.04
25-29	5.6	4.9	0.03	4.8	4.9	0
30-34	5.9	5.1	0.04	5.4	5.6	-0.01
35-39	6.5	5.5	0.04	5.6	5.9	-0.01
40-44	8	6.7	0.05	7.7	7.9	-0.01
45-49	10.4	8.2	0.07	10.4	10.8	-0.01
50-54	10.7	9.2	0.05	11.3	11.6	-0.01
55-59	11.7	9.5	0.07	11.9	10.8	0.03
60-64	8.6	8.8	-0.01	9.7	9.6	0
65-69	6.2	8.1	-0.07	7.1	7.4	-0.01
70-74	5.8	8.2	-0.09	6.5	5.7	0.04
75-79	4	6	-0.09	4.4	4	0.02
80-84	4	5.5	-0.07	4	3.9	0
85-89	2.7	3.4	-0.04	2.5	2.1	0.03
90-94	1.4	1.5	-0.01	0.9	0.9	0
95-99	0.4	0.2	0.03	0.1	0.2	0
**Sex**						
Female	51.9	54.5	-0.05	53.9	54.4	-0.01

Table S6.103. *Continued.* Selected baseline characteristics for Italy IQVIA, for the long-term risk of self-harm and suicide

Characteristic	Before propensity score matching			After propensity score matching		
	**Targets, %**	**Comparators, %**	**Standardized difference**	**Targets, %**	**Comparators, %**	**Standardized difference**
**Medical history (general)**						
Acute respiratory disease	24	13.2	0.28	25.4	30.2	-0.11
Chronic liver disease	1.9	1.8	0.01	2.1	2.2	0
Chronic obstructive lung disease	1.8	1.7	0.01	1.9	1.7	0.02
Crohn's disease	0.3	0.2	0.01	0.3	0.4	-0.02
Dementia	0.8	0.7	0.01	0.4	0.5	0
Depressive disorder	6.6	6.1	0.02	7.1	7.7	-0.02
Diabetes mellitus	7.7	8.4	-0.03	7.9	7.7	0.01
Gastroesophageal reflux disease	9	7.6	0.05	9.1	10.2	-0.04
Gastrointestinal hemorrhage	0.3	0.2	0.01	0.3	0.3	0
Hyperlipidemia	10.8	11.3	-0.02	11.7	11.7	0
Hypertensive disorder	25.4	27.1	-0.04	27.8	26.3	0.03
Lesion of liver	0.3	0.3	-0.01	0.2	0.3	-0.02
Osteoarthritis	7.7	8.2	-0.02	7.7	8.8	-0.04
Pneumonia	9.7	0.9	0.4	4.3	3.7	0.03
Renal impairment	2.1	1.9	0.01	2	2.1	-0.01
Rheumatoid arthritis	0.6	0.6	-0.01	0.7	0.6	0
Schizophrenia	0.1	0.2	-0.02	0.2	0.2	0
Ulcerative colitis	0.4	0.3	0.02	0.5	0.4	0.02
Urinary tract infectious disease	2.8	2	0.05	2.8	3.1	-0.02

Table S6.103. *Continued.* Selected baseline characteristics for Italy IQVIA, for the long-term risk of self-harm and suicide

**Characteristic**	**Before propensity score matching**			**After propensity score matching**		
	**Targets, %**	**Comparators, %**	**Standardized difference**	**Targets, %**	**Comparators, %**	**Standardized difference**
**Medical history (cardiovascular disease)**						
Atrial fibrillation	3	3	0	2.8	2.9	-0.01
Cerebrovascular disease	3.8	4	-0.01	3.6	3.4	0.01
Coronary arteriosclerosis	0.4	0.3	0.02	0.3	0.4	0
Heart disease	12.3	12.2	0	12.6	12.5	0
Heart failure	1.2	1.2	0	1	1.1	0
Peripheral vascular disease	0.5	0.6	-0.01	0.5	0.5	0
Pulmonary embolism	0.3	0.2	0.02	0.3	0.3	0
Venous thrombosis	0.5	0.5	0.01	0.6	0.7	-0.01
Medical history: Neoplasms						
Malignant lymphoma	0.4	0.3	0.01	0.3	0.5	-0.03
Malignant neoplasm of anorectum	0.1	0.2	0	0.2	0.2	0
Malignant neoplastic disease	6.2	6.2	0	6.3	6.1	0.01
Malignant tumor of breast	1.4	1.5	-0.01	1.5	1.3	0.01
Malignant tumor of colon	0.4	0.5	-0.01	0.3	0.4	-0.01
Malignant tumor of urinary bladder	0.4	0.4	0	0.4	0.3	0.01
Primary malignant neoplasm of prostate	0.8	0.7	0.01	0.8	0.6	0.01
**Medication use**						
Agents acting on the renin-angiotensin system	20.6	23.5	-0.07	23	21.7	0.03
Antibacterials for systemic use	48.3	32	0.34	41.8	45.5	-0.07
Antidepressants	10.7	9.8	0.03	11.1	11.5	-0.01
Antiepileptics	4.6	4.4	0.01	4.8	5.1	-0.01
Antiinflammatory and antirheumatic products	29.3	29	0.01	31.4	33	-0.03
Antineoplastic agents	2.1	2.2	-0.01	2.2	2.3	-0.01
Antipsoriatics	1.3	1.3	0.01	1.3	1.3	0
Antithrombotic agents	18.6	16.4	0.06	16.4	15.5	0.03

Table S6.103. *Continued.* Selected baseline characteristics for Italy IQVIA, for the long-term risk of self-harm and suicide

Characteristic	Before propensity score matching			After propensity score matching		
	Targets, %	Comparators, %	Standardized difference	Targets, %	Comparators, %	Standardized difference
**Medication use**						
Beta blocking agents	14.6	16.2	-0.04	15.1	14.8	0.01
Calcium channel blockers	9.4	10.5	-0.04	10.3	9.3	0.03
Diuretics	14.1	16.2	-0.06	15.2	14.2	0.03
Drugs for acid-related disorders	26.1	24.1	0.05	26	26.7	-0.02
Drugs for obstructive airway diseases	19.6	16	0.1	20.2	22.1	-0.05
Drugs used in diabetes	6.7	7.6	-0.04	6.8	6.7	0
Immunosuppressants	0.9	0.8	0.01	1	0.9	0
Lipid modifying agents	14.2	16.8	-0.07	15.4	14.7	0.02
Opioids	7.8	6.8	0.04	7.7	8.6	-0.04
Psycholeptics	12.4	11.5	0.03	13	13.9	-0.03
Psychostimulants, agents used for ADHD and nootropics	1	0.8	0.02	1.1	1.4	-0.03

Table S6.104. Selected baseline characteristics for Italy IQVIA, for the long-term risk of sleep disorders

Characteristic	Before propensity score matching			After propensity score matching		
	**Targets, %**	**Comparators, %**	**Standardized difference**	**Targets, %**	**Comparators, %**	**Standardized difference**
**Age group (years)**						
10-14	0.5	0.5	-0.01	0.5	0.8	-0.03
15-19	2.8	3.9	-0.06	3.2	3.5	-0.02
20-24	4.8	4.7	0	4.1	4.9	-0.04
25-29	5.6	4.8	0.03	5.1	5.1	0
30-34	5.9	5	0.04	5.6	5.9	-0.01
35-39	6.5	5.5	0.04	5.8	6.1	-0.01
40-44	8	6.6	0.05	7.8	8.2	-0.01
45-49	10.4	8.2	0.07	10.6	10.9	-0.01
50-54	10.7	9.2	0.05	11.1	11.5	-0.01
55-59	11.7	9.5	0.07	11.9	10.8	0.04
60-64	8.6	8.9	-0.01	9.5	9.3	0
65-69	6.2	8.1	-0.08	7	7.2	-0.01
70-74	5.8	8.2	-0.09	6.6	5.4	0.05
75-79	4	6.1	-0.1	4.1	3.8	0.02
80-84	4	5.5	-0.07	3.7	3.7	0
85-89	2.7	3.4	-0.04	2.4	1.9	0.04
90-94	1.4	1.5	0	0.8	0.8	0
95-99	0.4	0.2	0.03	0.2	0.1	0.01
**Sex**						
Female	51.9	54.5	-0.05	53.5	54.1	-0.01

Table S6.104. *Continued.* Selected baseline characteristics for Italy IQVIA, for the long-term risk of sleep disorders

Characteristic	Before propensity score matching			After propensity score matching		
	**Targets, %**	**Comparators, %**	**Standardized difference**	**Targets, %**	**Comparators, %**	**Standardized difference**
**Medical history (general)**						
Acute respiratory disease	24	13.3	0.28	25.1	29.8	-0.11
Chronic liver disease	1.9	1.8	0	1.9	2.1	-0.01
Chronic obstructive lung disease	1.8	1.7	0.01	1.8	1.6	0.01
Crohn's disease	0.3	0.2	0.01	0.3	0.3	-0.01
Dementia	0.8	0.7	0.02	0.5	0.5	0
Depressive disorder	6.6	6.1	0.02	6.5	7	-0.02
Diabetes mellitus	7.7	8.5	-0.03	7.6	7.4	0.01
Gastroesophageal reflux disease	9	7.6	0.05	8.7	9.4	-0.03
Gastrointestinal hemorrhage	0.3	0.3	0	0.3	0.3	0.01
Hyperlipidemia	10.8	11.4	-0.02	11.4	11	0.01
Hypertensive disorder	25.4	27.2	-0.04	26.8	25	0.04
Lesion of liver	0.3	0.3	0	0.2	0.3	-0.02
Obesity	0.5	0.3	0.03	0.4	0.4	0
Osteoarthritis	7.7	8.1	-0.02	7.1	8.1	-0.04
Pneumonia	9.7	0.9	0.4	4.1	3.6	0.03
Psoriasis	1.7	1.4	0.02	1.8	1.7	0.01
Renal impairment	2.1	1.9	0.02	1.8	1.9	0
Schizophrenia	0.1	0.2	-0.02	0.2	0.2	0
Ulcerative colitis	0.4	0.3	0.02	0.5	0.4	0.02
Urinary tract infectious disease	2.8	2.1	0.04	2.6	2.9	-0.01
Viral hepatitis C	0.4	0.4	0	0.4	0.4	-0.01

Table S6.104. *Continued.* Selected baseline characteristics for Italy IQVIA, for the long-term risk of sleep disorders

Characteristic	Before propensity score matching			After propensity score matching		
	Targets, %	Comparators, %	Standardized difference	Targets, %	Comparators, %	Standardized difference
**Medical history (cardiovascular disease)**						
Atrial fibrillation	3	3	0	2.5	2.7	-0.01
Cerebrovascular disease	3.8	3.9	-0.01	3.4	3.1	0.02
Coronary arteriosclerosis	0.4	0.3	0.01	0.4	0.4	0
Heart disease	12.3	12.1	0	12	11.9	0
Ischemic heart disease	3.8	3.8	0	3.6	3.6	0
Peripheral vascular disease	0.5	0.6	-0.01	0.5	0.4	0
Pulmonary embolism	0.3	0.2	0.02	0.3	0.3	0
Venous thrombosis	0.5	0.5	0.01	0.6	0.6	-0.01
**Medical history (neoplasms)**						
Malignant lymphoma	0.4	0.3	0.01	0.3	0.5	-0.03
Malignant neoplasm of anorectum	0.1	0.2	0	0.2	0.2	0
Malignant neoplastic disease	6.2	6.1	0	6.1	5.9	0.01
Malignant tumor of breast	1.4	1.5	-0.01	1.4	1.3	0.01
Malignant tumor of colon	0.4	0.5	-0.01	0.3	0.4	-0.01
Malignant tumor of urinary bladder	0.4	0.4	0	0.4	0.3	0.01
Primary malignant neoplasm of prostate	0.8	0.7	0.01	0.7	0.6	0.01
**Medication use**						
Agents acting on the renin-angiotensin system	20.6	23.6	-0.07	22.2	20.8	0.04
Antibacterials for systemic use	48.3	31.9	0.34	41.3	45.1	-0.08
Antidepressants	10.7	9.7	0.03	10.2	10.6	-0.01
Antiepileptics	4.6	4.4	0.01	4.7	4.8	0
Antiinflammatory and antirheumatic products	29.3	29	0.01	30.2	32	-0.04
Antineoplastic agents	2.1	2.2	-0.01	2.1	2.2	-0.01
Antipsoriatics	1.3	1.3	0	1.2	1.2	-0.01
Antithrombotic agents	18.6	16.4	0.06	15.7	14.7	0.03

Table S6.104. *Continued.* Selected baseline characteristics for Italy IQVIA, for the long-term risk of sleep disorders

Characteristic	Before propensity score matching			After propensity score matching		
	Targets, %	Comparators, %	Standardized difference	Targets, %	Comparators, %	Standardized difference
**Medication use**						
Beta blocking agents	14.6	16	-0.04	14.4	14.2	0
Calcium channel blockers	9.4	10.5	-0.04	9.8	8.8	0.03
Diuretics	14.1	16.3	-0.06	14.3	13.3	0.03
Drugs for acid-related disorders	26.1	24.3	0.04	24.9	25.4	-0.01
Drugs for obstructive airway diseases	19.6	16	0.09	20	21.6	-0.04
Drugs used in diabetes	6.7	7.7	-0.04	6.7	6.5	0.01
Immunosuppressants	0.9	0.8	0.01	0.9	0.9	0
Lipid modifying agents	14.2	16.9	-0.07	15.1	14	0.03
Opioids	7.8	6.8	0.04	7	8.2	-0.04
Psycholeptics	12.4	11.5	0.03	10.7	11.6	-0.03
Psychostimulants, agents used for ADHD and nootropics	1	0.8	0.02	0.9	1.4	-0.04

Table S6.105. Selected baseline characteristics for Italy IQVIA, for the long-term risk of dementia

Characteristic	Before propensity score matching			After propensity score matching		
	**Targets, %**	**Comparators, %**	**Standardized difference**	**Targets, %**	**Comparators, %**	**Standardized difference**
**Age group (years)**						
10-14	0.5	0.5	-0.01	0.5	0.7	-0.04
15-19	2.8	3.8	-0.06	3	3.4	-0.02
20-24	4.8	4.7	0	3.9	4.7	-0.04
25-29	5.6	4.8	0.03	4.9	4.9	0
30-34	5.9	5.1	0.04	5.4	5.6	-0.01
35-39	6.5	5.5	0.04	5.6	5.9	-0.01
40-44	8	6.6	0.05	7.8	8	-0.01
45-49	10.4	8.2	0.07	10.5	10.8	-0.01
50-54	10.7	9.3	0.05	11.4	11.7	-0.01
55-59	11.7	9.7	0.07	11.9	10.8	0.04
60-64	8.6	8.8	-0.01	9.8	9.7	0
65-69	6.2	8.1	-0.07	7.2	7.5	-0.01
70-74	5.8	8.1	-0.09	6.5	5.7	0.04
75-79	4	6	-0.09	4.4	4	0.02
80-84	4	5.5	-0.07	3.9	3.8	0
85-89	2.7	3.4	-0.04	2.4	1.9	0.03
90-94	1.4	1.5	0	0.8	0.8	0
95-99	0.4	0.2	0.03	0.1	0.1	-0.01
**Sex**						
Female	51.9	54.5	-0.05	53.7	54.3	-0.01

Table S6.105. *Continued.* Selected baseline characteristics for Italy IQVIA, for the long-term risk of dementia

Characteristic	Before propensity score matching			After propensity score matching		
	Targets, %	Comparators, %	Standardized difference	Targets, %	Comparators, %	Standardized difference
**Medical history (general)**						
Acute respiratory disease	24	13.3	0.28	25.5	30.2	-0.1
Chronic liver disease	1.9	1.8	0	2.1	2.2	-0.01
Chronic obstructive lung disease	1.8	1.7	0.01	1.9	1.7	0.02
Crohn's disease	0.3	0.2	0.01	0.3	0.4	-0.02
Depressive disorder	6.6	6.1	0.02	6.9	7.6	-0.03
Diabetes mellitus	7.7	8.4	-0.03	7.8	7.6	0.01
Gastroesophageal reflux disease	9	7.7	0.05	9	10.1	-0.04
Gastrointestinal hemorrhage	0.3	0.3	0	0.3	0.3	0
Human immunodeficiency virus infection	0.1	0.1	0.02	0.1	0.1	0.02
Hyperlipidemia	10.8	11.4	-0.02	11.6	11.7	0
Hypertensive disorder	25.4	27	-0.04	27.7	26.1	0.04
Lesion of liver	0.3	0.3	-0.01	0.2	0.3	-0.02
Obesity	0.5	0.3	0.03	0.4	0.5	0
Osteoarthritis	7.7	8.2	-0.02	7.7	8.7	-0.04
Pneumonia	9.7	0.9	0.4	4.3	3.7	0.03
Psoriasis	1.7	1.4	0.03	1.8	1.8	0
Renal impairment	2.1	1.9	0.02	1.9	2	-0.01
Rheumatoid arthritis	0.6	0.7	-0.01	0.6	0.6	0
Schizophrenia	0.1	0.2	-0.02	0.2	0.2	0
Ulcerative colitis	0.4	0.3	0.02	0.5	0.4	0.02
Urinary tract infectious disease	2.8	2.1	0.05	2.8	3.1	-0.02
Viral hepatitis C	0.4	0.4	0	0.4	0.4	0

Table S6.105. *Continued.* Selected baseline characteristics for Italy IQVIA, for the long-term risk of dementia

**Characteristic**	**Before propensity score matching**			**After propensity score matching**		
	**Targets, %**	**Comparators, %**	**Standardized difference**	**Targets, %**	**Comparators, %**	**Standardized difference**
**Medical history (cardiovascular disease)**						
Atrial fibrillation	3	2.9	0	2.8	2.9	0
Cerebrovascular disease	3.8	4	-0.01	3.5	3.2	0.02
Coronary arteriosclerosis	0.4	0.3	0.01	0.3	0.4	0
Heart disease	12.3	12.2	0	12.5	12.3	0
Heart failure	1.2	1.1	0.01	1	1	0
Peripheral vascular disease	0.5	0.6	-0.01	0.5	0.5	0
Pulmonary embolism	0.3	0.2	0.02	0.3	0.3	0
Venous thrombosis	0.5	0.5	0.01	0.6	0.6	-0.01
**Medical history (neoplasms)**						
Malignant lymphoma	0.4	0.3	0	0.3	0.5	-0.03
Malignant neoplasm of anorectum	0.1	0.2	0	0.2	0.2	0
Malignant neoplastic disease	6.2	6.2	0	6.3	6.1	0.01
Malignant tumor of breast	1.4	1.5	-0.01	1.5	1.3	0.01
Malignant tumor of colon	0.4	0.5	-0.01	0.3	0.4	-0.01
Malignant tumor of urinary bladder	0.4	0.4	0	0.4	0.3	0.01
Primary malignant neoplasm of prostate	0.8	0.8	0	0.8	0.6	0.01
**Medication use**						
Agents acting on the renin-angiotensin system	20.6	23.5	-0.07	22.9	21.5	0.03
Antibacterials for systemic use	48.3	32	0.34	41.8	45.5	-0.07
Antidepressants	10.7	9.8	0.03	10.9	11.3	-0.01
Antiepileptics	4.6	4.5	0.01	4.7	5	-0.02
Antiinflammatory and antirheumatic products	29.3	29	0.01	31.5	32.9	-0.03
Antineoplastic agents	2.1	2.2	-0.01	2.1	2.3	-0.01
Antipsoriatics	1.3	1.3	0.01	1.3	1.3	0
Antithrombotic agents	18.6	16.5	0.06	16.1	15.2	0.03

Table S6.105. *Continued.* Selected baseline characteristics for Italy IQVIA, for the long-term risk of dementia

**Characteristic**	**Before propensity score matching**			**After propensity score matching**		
	**Targets, %**	**Comparators, %**	**Standardized difference**	**Targets, %**	**Comparators, %**	**Standardized difference**
**Medication use**						
Beta blocking agents	14.6	16.1	-0.04	15	14.7	0.01
Calcium channel blockers	9.4	10.5	-0.04	10.2	9.3	0.03
Diuretics	14.1	16.2	-0.06	15	14	0.03
Drugs for acid-related disorders	26.1	24.3	0.04	25.8	26.5	-0.01
Drugs for obstructive airway diseases	19.6	15.9	0.1	20.3	22.1	-0.04
Drugs used in diabetes	6.7	7.6	-0.04	6.8	6.7	0.01
Immunosuppressants	0.9	0.8	0.01	1	0.9	0
Lipid modifying agents	14.2	16.9	-0.07	15.3	14.6	0.02
Opioids	7.8	6.9	0.04	7.6	8.6	-0.04
Psycholeptics	12.4	11.5	0.03	12.8	13.7	-0.03
Psychostimulants, agents used for ADHD and nootropics	1	0.8	0.02	1	1.4	-0.03

Table S6.106. Selected baseline characteristics for Italy IQVIA, for the long-term risk of neurodevelopmental disorders

Characteristic	Before propensity score matching			After propensity score matching		
	Targets, %	Comparators, %	Standardized difference	Targets, %	Comparators, %	Standardized difference
**Age group (years)**						
10-14	0.5	0.5	-0.01	0.5	0.7	-0.03
15-19	2.8	3.9	-0.06	3	3.3	-0.02
20-24	4.8	4.7	0	3.9	4.6	-0.04
25-29	5.6	4.9	0.03	4.8	4.9	0
30-34	5.9	5.1	0.04	5.4	5.6	-0.01
35-39	6.5	5.5	0.04	5.6	5.9	-0.01
40-44	8	6.7	0.05	7.7	7.9	-0.01
45-49	10.4	8.2	0.07	10.4	10.8	-0.01
50-54	10.7	9.2	0.05	11.3	11.6	-0.01
55-59	11.7	9.6	0.07	11.9	10.8	0.03
60-64	8.6	8.7	-0.01	9.7	9.6	0
65-69	6.2	8.3	-0.08	7.2	7.4	-0.01
70-74	5.8	8.1	-0.09	6.6	5.7	0.04
75-79	4	6	-0.1	4.4	4.1	0.02
80-84	4	5.4	-0.07	4	3.9	0
85-89	2.7	3.4	-0.04	2.5	2.1	0.03
90-94	1.4	1.5	0	0.9	0.9	0
95-99	0.4	0.2	0.03	0.1	0.2	0
**Sex**						
Female	51.9	54.4	-0.05	53.9	54.4	-0.01

Table S6.106. *Continued.* Selected baseline characteristics for Italy IQVIA, for the long-term risk of neurodevelopmental disorders

Characteristic	Before propensity score matching			After propensity score matching		
	Targets, %	Comparators, %	Standardized difference	Targets, %	Comparators, %	Standardized difference
**Medical history (general)**						
Acute respiratory disease	24	13.2	0.28	25.4	30.1	-0.1
Chronic liver disease	1.9	1.8	0	2.1	2.2	0
Chronic obstructive lung disease	1.8	1.8	0	1.9	1.7	0.02
Crohn’s disease	0.3	0.2	0.01	0.3	0.4	-0.02
Dementia	0.8	0.7	0.02	0.4	0.5	0
Depressive disorder	6.6	6.3	0.02	7.1	7.7	-0.02
Diabetes mellitus	7.7	8.5	-0.03	7.9	7.7	0.01
Gastroesophageal reflux disease	9	7.6	0.05	9.1	10.2	-0.04
Human immunodeficiency virus infection	0.1	0.1	0.02	0.1	0.1	0.02
Hyperlipidemia	10.8	11.4	-0.02	11.8	11.7	0
Hypertensive disorder	25.4	27.2	-0.04	27.8	26.3	0.03
Lesion of liver	0.3	0.3	0	0.2	0.3	-0.02
Osteoarthritis	7.7	8.2	-0.02	7.7	8.8	-0.04
Pneumonia	9.7	0.9	0.4	4.3	3.7	0.03
Psoriasis	1.7	1.4	0.02	1.8	1.8	0
Renal impairment	2.1	1.9	0.02	2	2.1	0
Rheumatoid arthritis	0.6	0.6	-0.01	0.7	0.6	0.01
Schizophrenia	0.1	0.2	-0.02	0.2	0.2	0
Ulcerative colitis	0.4	0.3	0.02	0.5	0.4	0.02
Urinary tract infectious disease	2.8	2.1	0.04	2.8	3.2	-0.02
Viral hepatitis C	0.4	0.4	0	0.4	0.4	0

Table S6.106. *Continued.* Selected baseline characteristics for Italy IQVIA, for the long-term risk of neurodevelopmental disorders

Characteristic	Before propensity score matching			After propensity score matching		
	**Targets, %**	**Comparators, %**	**Standardized difference**	**Targets, %**	**Comparators, %**	**Standardized difference**
**Medical history (cardiovascular disease)**						
Atrial fibrillation	3	3	0	2.8	2.9	0
Cerebrovascular disease	3.8	3.9	-0.01	3.6	3.4	0.02
Coronary arteriosclerosis	0.4	0.3	0.02	0.4	0.4	0
Heart disease	12.3	12.2	0	12.6	12.6	0
Heart failure	1.2	1.2	0	1	1.1	0
Ischemic heart disease	3.8	3.8	0	3.8	3.7	0
Peripheral vascular disease	0.5	0.6	-0.01	0.5	0.5	0
Pulmonary embolism	0.3	0.2	0.02	0.3	0.3	0
Venous thrombosis	0.5	0.5	0.01	0.6	0.7	-0.01
**Medical history (neoplasms)**						
Malignant lymphoma	0.4	0.3	0	0.3	0.5	-0.03
Malignant neoplastic disease	6.2	6.2	0	6.4	6.1	0.01
Malignant tumor of breast	1.4	1.5	-0.01	1.5	1.3	0.01
Malignant tumor of colon	0.4	0.5	-0.01	0.3	0.4	-0.01
Malignant tumor of urinary bladder	0.4	0.4	0	0.4	0.3	0.01
Primary malignant neoplasm of prostate	0.8	0.7	0	0.8	0.6	0.01
**Medication use**						
Agents acting on the renin-angiotensin system	20.6	23.7	-0.07	23	21.7	0.03
Antibacterials for systemic use	48.3	32.1	0.34	41.8	45.5	-0.07
Antidepressants	10.7	9.8	0.03	11.1	11.5	-0.01
Antiepileptics	4.6	4.4	0.01	4.8	5	-0.01
Antiinflammatory and antirheumatic products	29.3	29	0.01	31.5	33	-0.03
Antineoplastic agents	2.1	2.2	-0.01	2.1	2.3	-0.01
Antipsoriatics	1.3	1.2	0.01	1.3	1.3	0
Antithrombotic agents	18.6	16.5	0.06	16.5	15.5	0.03

Table S6.106. *Continued.* Selected baseline characteristics for Italy IQVIA, for the long-term risk of neurodevelopmental disorders

Characteristic	Before propensity score matching			After propensity score matching		
	Targets, %	Comparators, %	Standardized difference	Targets, %	Comparators, %	Standardized difference
**Medication use**						
Beta blocking agents	14.6	16	-0.04	15.2	14.9	0.01
Calcium channel blockers	9.4	10.6	-0.04	10.3	9.4	0.03
Diuretics	14.1	16.3	-0.06	15.2	14.2	0.03
Drugs for acid-related disorders	26.1	24.3	0.04	26.1	26.7	-0.01
Drugs for obstructive airway diseases	19.6	16	0.09	20.3	22.1	-0.04
Drugs used in diabetes	6.7	7.7	-0.04	6.9	6.8	0
Immunosuppressants	0.9	0.8	0.01	1	0.9	0
Lipid modifying agents	14.2	16.9	-0.08	15.5	14.7	0.02
Opioids	7.8	6.9	0.04	7.7	8.7	-0.04
Psycholeptics	12.4	11.6	0.02	13	13.9	-0.03
Psychostimulants, agents used for ADHD and nootropics	1	0.8	0.02	1.1	1.4	-0.03

Table S6.107. Selected baseline characteristics for Italy IQVIA, for the long-term risk of any of psychiatric and neuropsychiatric disorders

Characteristic	Before propensity score matching			After propensity score matching		
	Targets, %	Comparators, %	Standardized difference	Targets, %	Comparators, %	Standardized difference
**Age group (years)**						
10-14	0.5	0.5	-0.01	0.6	0.8	-0.03
15-19	2.8	3.8	-0.06	3.6	3.8	-0.01
20-24	4.8	4.8	0	4.5	5.4	-0.04
25-29	5.6	4.8	0.03	5.6	5.6	0
30-34	5.9	5.1	0.04	6	6.2	-0.01
35-39	6.5	5.5	0.04	5.9	6.4	-0.02
40-44	8	6.7	0.05	7.8	8.4	-0.02
45-49	10.4	8.3	0.07	11	11.1	0
50-54	10.7	9.1	0.05	11.2	11.5	-0.01
55-59	11.7	9.6	0.07	11.9	10.8	0.03
60-64	8.6	8.7	0	9.7	9.2	0.02
65-69	6.2	8.1	-0.07	6.7	7	-0.01
70-74	5.8	8.2	-0.09	6.2	5.1	0.05
75-79	4	6.1	-0.1	3.8	3.2	0.03
80-84	4	5.4	-0.07	2.9	3.1	-0.01
85-89	2.7	3.4	-0.04	1.6	1.5	0.01
90-94	1.4	1.5	0	0.6	0.7	-0.01
95-99	0.4	0.2	0.03	<0.1	0.1	0
**Sex**						
Female	51.9	54.4	-0.05	51.6	52.2	-0.01

Table S6.107. *Continued.* Selected baseline characteristics for Italy IQVIA, for the long-term risk of any of psychiatric and neuropsychiatric disorders

Characteristic	Before propensity score matching			After propensity score matching		
	Targets, %	Comparators, %	Standardized difference	Targets, %	Comparators, %	Standardized difference
**Medical history (general)**						
Acute respiratory disease	24	13.3	0.28	25.1	29.5	-0.1
Chronic obstructive lung disease	1.8	1.8	0	1.6	1.3	0.02
Crohn’s disease	0.3	0.2	0.01	0.3	0.3	-0.01
Diabetes mellitus	7.7	8.5	-0.03	7	6.8	0.01
Gastroesophageal reflux disease	9	7.7	0.05	7.9	8.3	-0.01
Gastrointestinal hemorrhage	0.3	0.2	0	0.3	0.3	0
Hyperlipidemia	10.8	11.4	-0.02	10.4	10.2	0.01
Hypertensive disorder	25.4	27.2	-0.04	24.9	23.5	0.03
Lesion of liver	0.3	0.3	-0.01	0.2	0.3	-0.01
Obesity	0.5	0.3	0.03	0.3	0.3	-0.01
Osteoarthritis	7.7	8.2	-0.02	6.4	7	-0.02
Pneumonia	9.7	0.9	0.4	4	3.3	0.04
Psoriasis	1.7	1.4	0.02	1.6	1.5	0.01
Renal impairment	2.1	1.9	0.02	1.5	1.7	-0.01
Rheumatoid arthritis	0.6	0.6	-0.01	0.5	0.6	-0.02
Ulcerative colitis	0.4	0.3	0.02	0.5	0.4	0.02
Urinary tract infectious disease	2.8	2.1	0.04	2.3	2.5	-0.01
Viral hepatitis C	0.4	0.4	0	0.4	0.4	0

Table S6.107. *Continued.* Selected baseline characteristics for Italy IQVIA, for the long-term risk of any of psychiatric and neuropsychiatric disorders

**Characteristic**	**Before propensity score matching**			**After propensity score matching**		
	**Targets, %**	**Comparators, %**	**Standardized difference**	**Targets, %**	**Comparators, %**	**Standardized difference**
**Medical history (cardiovascular disease)**						
Atrial fibrillation	3	3	0	2.2	2.4	-0.02
Cerebrovascular disease	3.8	4	-0.01	2.8	2.4	0.03
Coronary arteriosclerosis	0.4	0.3	0.02	0.4	0.4	0
Heart failure	1.2	1.2	0.01	0.8	0.7	0
Peripheral vascular disease	0.5	0.6	-0.01	0.4	0.4	0
Pulmonary embolism	0.3	0.2	0.02	0.3	0.2	0.02
Venous thrombosis	0.5	0.4	0.01	0.5	0.6	-0.02
Medical history: Neoplasms						
Malignant lymphoma	0.4	0.3	0.01	0.3	0.5	-0.03
Malignant neoplasm of anorectum	0.1	0.2	0	0.2	0.1	0
Malignant neoplastic disease	6.2	6.2	0	5.7	5.5	0.01
Malignant tumor of breast	1.4	1.5	-0.01	1.4	1.2	0.02
Malignant tumor of colon	0.4	0.5	-0.01	0.3	0.4	-0.01
Malignant tumor of urinary bladder	0.4	0.4	0	0.4	0.3	0.01
Primary malignant neoplasm of prostate	0.8	0.8	0	0.7	0.6	0.01
Medication use						
Agents acting on the renin-angiotensin system	20.6	23.6	-0.07	20.7	19.7	0.03
Antibacterials for systemic use	48.3	32.1	0.33	40.4	44.3	-0.08
Antidepressants	10.7	9.9	0.03	3.8	4.2	-0.02
Antiepileptics	4.6	4.4	0.01	3.3	3.2	0
Antiinflammatory and antirheumatic products	29.3	29.2	0	28.7	30.3	-0.03
Antineoplastic agents	2.1	2.2	-0.01	1.8	2	-0.01
Antipsoriatics	1.3	1.3	0	1.1	1.2	-0.01

Table S6.107. *Continued.* Selected baseline characteristics for Italy IQVIA, for the long-term risk of any of psychiatric and neuropsychiatric disorders

**Characteristic**	**Before propensity score matching**			**After propensity score matching**		
	**Targets, %**	**Comparators, %**	**Standardized difference**	**Targets, %**	**Comparators, %**	**Standardized difference**
**Medication use**						
Antithrombotic agents	18.6	16.5	0.06	13.9	13.3	0.02
Beta blocking agents	14.6	16	-0.04	12.9	13.2	-0.01
Calcium channel blockers	9.4	10.6	-0.04	8.9	8.1	0.03
Diuretics	14.1	16.3	-0.06	13.1	12.3	0.02
Drugs for acid-related disorders	26.1	24.3	0.04	22.8	23.1	-0.01
Drugs for obstructive airway diseases	19.6	16.1	0.09	19.3	20.9	-0.04
Drugs used in diabetes	6.7	7.7	-0.04	6.3	6	0.01
Immunosuppressants	0.9	0.8	0.01	0.9	0.8	0.01
Lipid modifying agents	14.2	16.9	-0.08	14	13	0.03
Opioids	7.8	6.9	0.04	6	7.2	-0.05
Psycholeptics	12.4	11.5	0.03	6.4	6.9	-0.02
Psychostimulants, agents used for ADHD and nootropics	1	0.8	0.02	0.7	1.1	-0.05

Table S6.108. Selected baseline characteristics for UK IMRD, for the short-term risk of depression

Characteristic	Before propensity score matching			After propensity score matching		
	**Targets, %**	**Comparators, %**	**Standardized difference**	**Targets, %**	**Comparators, %**	**Standardized difference**
**Age group (years)**						
0-4	1.7	3.9	-0.14	1.7	1.7	0
5-9	2.3	5.2	-0.15	2.4	2.2	0.01
10-14	3.8	5.2	-0.07	3.9	3.8	0.01
20-24	7.3	5.2	0.09	7.3	7.7	-0.02
25-29	7.6	6.2	0.06	7.5	7.8	-0.01
30-34	7.7	6.9	0.03	7.7	7.6	0
35-39	8.1	7	0.04	8.1	8	0
40-44	7.6	6.6	0.04	7.6	7.5	0.01
45-49	8.1	6.6	0.06	8.1	8.2	0
55-59	8.4	7.3	0.04	8.4	8.5	0
60-64	6	6.5	-0.02	6.1	6	0
65-69	3.6	5.6	-0.1	3.6	3.5	0
70-74	3.2	5.6	-0.12	3.2	3	0.01
75-79	2.8	4.1	-0.07	2.8	2.7	0.01
85-89	2.1	1.8	0.02	2.1	2.2	-0.01
90-94	1.4	0.8	0.06	1.4	1.5	-0.01
95-99	0.6	0.2	0.06	0.6	0.6	0
**Sex**						
Female	56.7	49.4	0.15	56.5	58.4	-0.04

Table S6.108. *Continued.* Selected baseline characteristics for UK IMRD, for the short-term risk of depression

Characteristic	Before propensity score matching			After propensity score matching		
	Targets, %	Comparators, %	Standardized difference	Targets, %	Comparators, %	Standardized difference
**Race**						
race = Asian	0.8	0.7	0.01	0.8	0.7	0.01
race = Black or African American	0.2	0.3	-0.02	0.2	0.2	0
race = White	4.5	6.6	-0.09	4.5	4.3	0.01
race = Asian Indian	0.9	0.9	0	0.9	0.9	0
race = Bangladeshi	0.2	0.1	0.02	0.2	0.1	0.01
race = Chinese	0.1	0.3	-0.06	0.1	0.1	0
race = Pakistani	1.7	0.6	0.11	1.7	1.8	-0.01
race = African	0.7	0.9	-0.02	0.7	0.8	-0.01
race = West Indian	0.1	0.1	0	0.1	0.1	-0.01
race = European	36.7	36.3	0.01	36.6	37.2	-0.01
race = Middle Eastern or North African	0.1	0.1	0	0.1	0.1	-0.01
race = Arab	0.1	0.1	0	0.1	0.1	0
**Medical history (general)**						
Acute respiratory disease	4.4	2.6	0.1	4.3	4.7	-0.02
Attention deficit hyperactivity disorder	0.1	0.1	0.01	0.1	0.1	-0.01
Chronic obstructive lung disease	0.7	0.6	0.02	0.7	0.9	-0.02
Dementia	0.4	0.2	0.04	0.4	0.5	-0.01
Diabetes mellitus	0.7	0.6	0.01	0.7	0.8	-0.01
Gastroesophageal reflux disease	0.3	0.2	0.02	0.3	0.4	-0.01
Gastrointestinal hemorrhage	0.5	0.4	0.02	0.5	0.6	-0.01
Hyperlipidemia	0.2	0.2	0	0.2	0.2	0
Hypertensive disorder	0.9	0.8	0.01	0.9	1	-0.01
Lesion of liver	0.1	0.1	0.01	0.1	0.2	-0.01
Obesity	0.2	0.1	0.02	0.2	0.2	0
Osteoarthritis	0.8	0.8	0	0.8	0.8	0

Table S6.108. *Continued.* Selected baseline characteristics for UK IMRD, for the short-term risk of depression

Characteristic	Before propensity score matching			After propensity score matching		
	Targets, %	Comparators, %	Standardized difference	Targets, %	Comparators, %	Standardized difference
**Medical history (general)**						
Pneumonia	1.1	0.2	0.11	1	1.1	-0.01
Psoriasis	0.4	0.3	0.02	0.3	0.4	-0.01
Renal impairment	1.1	0.5	0.06	1.1	1.2	-0.01
Rheumatoid arthritis	0.1	0.1	0.01	0.1	0.1	0
Urinary tract infectious disease	2.1	1	0.09	2.1	2.3	-0.01
**Medical history (cardiovascular disease)**						
Atrial fibrillation	0.6	0.4	0.04	0.6	0.7	-0.01
Cerebrovascular disease	0.4	0.3	0.03	0.4	0.5	-0.01
Coronary arteriosclerosis	0.1	0.1	0	0.1	0.1	0
Heart disease	1.7	1.1	0.06	1.7	2	-0.02
Heart failure	0.4	0.2	0.04	0.4	0.5	-0.01
Ischemic heart disease	0.4	0.3	0.03	0.4	0.5	-0.01
Peripheral vascular disease	0.1	0.1	0.01	0.1	0.1	0
Pulmonary embolism	0.2	0.1	0.03	0.2	0.2	0
Venous thrombosis	0.3	0.2	0.01	0.3	0.4	-0.02
Medical history: Neoplasms						
Malignant neoplastic disease	1	0.8	0.02	1	1.2	-0.02
Malignant tumor of breast	0.1	0.1	0	0.1	0.1	-0.01
Malignant tumor of lung	0.1	0.1	0.01	0.1	0.1	-0.01

Table S6.108. *Continued.* Selected baseline characteristics for UK IMRD, for the short-term risk of depression

Characteristic	Before propensity score matching			After propensity score matching		
	**Targets, %**	**Comparators, %**	**Standardized difference**	**Targets, %**	**Comparators, %**	**Standardized difference**
**Medication use**						
Agents acting on the renin-angiotensin system	12.6	12.2	0.01	12.6	12.8	-0.01
Antibacterials for systemic use	38.4	27.7	0.23	38.2	40.7	-0.05
Antidepressants	22.2	16	0.16	21.1	23	-0.05
Antiepileptics	5.9	4.4	0.07	5.9	6.6	-0.03
Antiinflammatory and antirheumatic products	20.5	16.6	0.1	20.4	21.6	-0.03
Antineoplastic agents	1	1	0	1	1	0
Antipsoriatics	4.5	3	0.08	4.5	4.9	-0.02
Antithrombotic agents	10.1	9.2	0.03	10.1	10.5	-0.01
Beta blocking agents	10	8.9	0.04	9.8	10.3	-0.02
Calcium channel blockers	8.3	8.3	0	8.2	8.3	0
Diuretics	6.9	6.1	0.03	6.9	7	-0.01
Drugs for acid-related disorders	25.8	20.8	0.12	25.6	27.7	-0.05
Drugs for obstructive airway diseases	23.7	19.1	0.11	23.6	24.9	-0.03
Drugs used in diabetes	6.5	4.9	0.07	6.4	6.7	-0.01
Immunosuppressants	1	0.9	0.01	1	1	0
Lipid modifying agents	13.9	14.2	-0.01	13.9	14.1	-0.01
Opioids	17.6	13	0.13	17.5	18.8	-0.04
Psycholeptics	10.3	7.7	0.09	10	11.2	-0.04
Psychostimulants, agents used for ADHD and nootropics	0.2	0.3	-0.01	0.2	0.3	-0.01

Table S6.109. Selected baseline characteristics for UK IMRD, for the short-term risk of anxiety disorders

Characteristic	Before propensity score matching			After propensity score matching		
	Targets, %	Comparators, %	Standardized difference	Targets, %	Comparators, %	Standardized difference
**Age group (years)**						
5-9	2.3	5.2	-0.15	2.4	2.2	0.01
10-14	3.8	5.3	-0.07	3.9	3.8	0
15-19	6	4.9	0.05	6	6	0
20-24	7.3	5.2	0.09	7.3	7.7	-0.02
25-29	7.6	6.3	0.05	7.5	7.8	-0.01
30-34	7.7	7	0.03	7.7	7.6	0
35-39	8.1	6.9	0.05	8.1	8	0
40-44	7.6	6.5	0.05	7.6	7.5	0
45-49	8.1	6.7	0.06	8.2	8.2	0
50-54	8.9	7.4	0.06	9	9.1	0
55-59	8.4	7.4	0.04	8.4	8.5	0
60-64	6	6.4	-0.02	6.1	6	0
65-69	3.6	5.6	-0.1	3.6	3.5	0
70-74	3.2	5.6	-0.12	3.2	3	0.01
75-79	2.8	4.1	-0.07	2.8	2.7	0.01
80-84	2.5	2.8	-0.02	2.5	2.4	0
85-89	2.1	1.7	0.03	2.1	2.2	-0.01
90-94	1.4	0.8	0.06	1.4	1.5	-0.01
95-99	0.6	0.2	0.06	0.6	0.6	0
**Sex**						
Female	56.7	49.4	0.14	56.4	58.2	-0.04

Table S6.109. *Continued.* Selected baseline characteristics for UK IMRD, for the short-term risk of anxiety disorders

Characteristic	Before propensity score matching			After propensity score matching		
	Targets, %	Comparators, %	Standardized difference	Targets, %	Comparators, %	Standardized difference
**Race**						
race = Asian	0.8	0.6	0.02	0.8	0.7	0.01
race = Black or African American	0.2	0.3	-0.02	0.2	0.2	0
race = White	4.5	6.7	-0.1	4.5	4.2	0.01
race = Asian Indian	0.9	0.8	0.01	0.9	0.9	0
race = Bangladeshi	0.2	0.1	0.01	0.2	0.2	0.01
race = Chinese	0.1	0.3	-0.06	0.1	0.1	0
race = Pakistani	1.7	0.6	0.1	1.7	1.8	0
race = African	0.7	0.9	-0.02	0.7	0.7	-0.01
race = West Indian	0.1	0.1	-0.01	0.1	0.1	-0.01
race = European	36.7	36.2	0.01	36.6	37.2	-0.01
race = Middle Eastern or North African	0.1	0.1	0	0.1	0.1	-0.01
**Medical history (general)**						
Acute respiratory disease	4.4	2.6	0.1	4.3	4.7	-0.02
Attention deficit hyperactivity disorder	0.1	0.1	0	0.1	0.1	0
Chronic obstructive lung disease	0.7	0.6	0.02	0.7	0.9	-0.02
Dementia	0.4	0.2	0.04	0.4	0.5	-0.01
Depressive disorder	1.4	1.1	0.04	0.8	0.9	0
Diabetes mellitus	0.7	0.6	0.01	0.7	0.8	-0.01
Gastroesophageal reflux disease	0.3	0.2	0.02	0.3	0.4	-0.01
Gastrointestinal hemorrhage	0.5	0.4	0.02	0.5	0.6	-0.01
Hypertensive disorder	0.9	0.8	0.01	0.9	1	-0.01
Lesion of liver	0.1	0.1	0.01	0.1	0.2	-0.01
Obesity	0.2	0.1	0.02	0.2	0.2	0

Table S6.109. *Continued.* Selected baseline characteristics for UK IMRD, for the short-term risk of anxiety disorders

Characteristic	Before propensity score matching			After propensity score matching		
	**Targets, %**	**Comparators, %**	**Standardized difference**	**Targets, %**	**Comparators, %**	**Standardized difference**
**Medical history (general)**						
Osteoarthritis	0.8	0.8	0.01	0.8	0.8	0
Pneumonia	1.1	0.2	0.11	1	1.1	-0.01
Psoriasis	0.4	0.3	0.02	0.4	0.4	0
Renal impairment	1.1	0.5	0.06	1.1	1.2	-0.01
Rheumatoid arthritis	0.1	0.1	0.01	0.1	0.1	0
Urinary tract infectious disease	2.1	1	0.09	2.1	2.3	-0.01
**Medical history (cardiovascular disease)**						
Atrial fibrillation	0.6	0.3	0.04	0.6	0.7	-0.01
Cerebrovascular disease	0.4	0.3	0.03	0.4	0.5	-0.02
Coronary arteriosclerosis	0.1	0.1	0.01	0.1	0.1	0
Heart disease	1.7	1.1	0.05	1.7	2	-0.02
Heart failure	0.4	0.2	0.04	0.4	0.5	-0.01
Ischemic heart disease	0.4	0.3	0.02	0.4	0.5	-0.01
Peripheral vascular disease	0.1	0.1	0.01	0.1	0.1	0
Pulmonary embolism	0.2	0.1	0.03	0.2	0.2	0
Venous thrombosis	0.3	0.2	0.02	0.3	0.4	-0.02
**Medical history (neoplasms)**						
Malignant neoplastic disease	1	0.8	0.02	1	1.2	-0.02
Malignant tumor of breast	0.1	0.1	0	0.1	0.1	0
Malignant tumor of lung	0.1	0.1	0.01	0.1	0.1	-0.01

Table S6.109. *Continued.* Selected baseline characteristics for UK IMRD, for the short-term risk of anxiety disorders

Characteristic	Before propensity score matching			After propensity score matching		
	Targets, %	Comparators, %	Standardized difference	Targets, %	Comparators, %	Standardized difference
**Medication use**						
Agents acting on the renin-angiotensin system	12.6	12.2	0.01	12.6	12.8	-0.01
Antibacterials for systemic use	38.4	27.8	0.23	38.2	40.7	-0.05
Antidepressants	22.2	16.1	0.16	21.2	23.1	-0.05
Antiepileptics	5.9	4.3	0.07	5.9	6.5	-0.03
Antiinflammatory and antirheumatic products	20.5	16.2	0.11	20.4	21.5	-0.03
Antineoplastic agents	1	1	0	1	1	0
Antipsoriatics	4.5	3	0.08	4.5	4.9	-0.02
Antithrombotic agents	10.1	9.1	0.04	10.1	10.6	-0.01
Beta blocking agents	10	8.9	0.04	9.6	10.2	-0.02
Calcium channel blockers	8.3	8.3	0	8.2	8.3	0
Diuretics	6.9	6.1	0.03	6.9	7.1	-0.01
Drugs for acid-related disorders	25.8	20.7	0.12	25.6	27.7	-0.05
Drugs for obstructive airway diseases	23.7	19.1	0.11	23.5	25	-0.04
Drugs used in diabetes	6.5	4.9	0.07	6.4	6.8	-0.01
Immunosuppressants	1	0.9	0.01	1	1	0
Lipid modifying agents	13.9	14.2	-0.01	13.9	14.2	-0.01
Opioids	17.6	12.9	0.13	17.4	18.9	-0.04
Psycholeptics	10.3	7.7	0.09	9.9	11	-0.04
Psychostimulants, agents used for ADHD and nootropics	0.2	0.3	-0.01	0.2	0.3	-0.01

Table S6.110. Selected baseline characteristics for UK IMRD, for the short-term risk of alcohol misuse or dependence

Characteristic	Before propensity score matching			After propensity score matching		
	Targets, %	Comparators, %	Standardized difference	Targets, %	Comparators, %	Standardized difference
**Age group (years)**						
0-4	1.7	3.8	-0.13	1.7	1.7	0
5-9	2.3	5.2	-0.15	2.3	2.2	0.01
10-14	3.8	5.2	-0.07	3.8	3.8	0
20-24	7.3	5.2	0.09	7.4	7.8	-0.01
25-29	7.6	6.2	0.06	7.6	7.9	-0.01
30-34	7.7	7	0.03	7.7	7.7	0
35-39	8.1	7	0.04	8.1	8	0.01
40-44	7.6	6.5	0.04	7.7	7.5	0.01
45-49	8.1	6.8	0.05	8.1	8.2	0
50-54	8.9	7.4	0.06	8.9	9	0
55-59	8.4	7.3	0.04	8.4	8.5	0
60-64	6	6.5	-0.02	6	6	0
65-69	3.6	5.5	-0.1	3.6	3.5	0
70-74	3.2	5.6	-0.12	3.2	3	0.01
75-79	2.8	4.1	-0.07	2.8	2.7	0.01
80-84	2.5	2.9	-0.02	2.5	2.5	0
85-89	2.1	1.8	0.02	2.1	2.2	-0.01
90-94	1.4	0.8	0.06	1.4	1.5	-0.01
95-99	0.6	0.2	0.06	0.6	0.6	0
**Sex**						
Female	56.7	49.4	0.15	56.7	58.6	-0.04

Table S6.110. *Continued.* Selected baseline characteristics for UK IMRD, for the short-term risk of alcohol misuse or dependence

Characteristic	Before propensity score matching			After propensity score matching		
	Targets, %	Comparators, %	Standardized difference	Targets, %	Comparators, %	Standardized difference
**Race**						
race = Asian	0.8	0.7	0.01	0.8	0.7	0.01
race = Black or African American	0.2	0.3	-0.02	0.2	0.2	0
race = White	4.5	6.7	-0.1	4.5	4.2	0.01
race = Bangladeshi	0.2	0.1	0.01	0.2	0.2	0.01
race = Chinese	0.1	0.3	-0.06	0.1	0.1	0
race = Pakistani	1.7	0.6	0.11	1.7	1.8	0
race = African	0.7	0.9	-0.02	0.7	0.7	-0.01
race = West Indian	0.1	0.1	-0.01	0.1	0.1	0
race = European	36.7	36.1	0.01	36.7	37.3	-0.01
race = Middle Eastern or North African	0.1	0.1	0	0.1	0.1	-0.01
race = Arab	0.1	0.1	0	0.1	0.1	0
**Medical history (general)**						
Acute respiratory disease	4.4	2.6	0.1	4.4	4.8	-0.02
Attention deficit hyperactivity disorder	0.1	0.1	0	0.1	0.1	0
Chronic obstructive lung disease	0.7	0.6	0.02	0.7	0.9	-0.02
Dementia	0.4	0.2	0.04	0.4	0.5	-0.02
Depressive disorder	1.4	1	0.04	1.4	1.6	-0.01
Diabetes mellitus	0.7	0.6	0.02	0.7	0.8	-0.01
Gastroesophageal reflux disease	0.3	0.2	0.02	0.3	0.4	-0.01
Gastrointestinal hemorrhage	0.5	0.4	0.02	0.5	0.6	-0.01
Hypertensive disorder	0.9	0.8	0.01	0.9	1	-0.01
Lesion of liver	0.1	0.1	0.01	0.1	0.2	-0.01
Obesity	0.2	0.1	0.02	0.2	0.2	0

Table S6.110. *Continued.* Selected baseline characteristics for UK IMRD, for the short-term risk of alcohol misuse or dependence

Characteristic	Before propensity score matching			After propensity score matching		
	**Targets, %**	**Comparators, %**	**Standardized difference**	**Targets, %**	**Comparators, %**	**Standardized difference**
**Medical history (general)**						
Osteoarthritis	0.8	0.7	0.01	0.8	0.8	0
Pneumonia	1.1	0.2	0.11	1	1.1	0
Psoriasis	0.4	0.2	0.02	0.4	0.4	-0.01
Renal impairment	1.1	0.5	0.07	1.1	1.2	-0.01
Rheumatoid arthritis	0.1	0.1	0.01	0.1	0.1	0
Urinary tract infectious disease	2.1	1	0.09	2.1	2.4	-0.02
**Medical history (cardiovascular disease)**						
Atrial fibrillation	0.6	0.4	0.04	0.6	0.7	-0.01
Cerebrovascular disease	0.4	0.3	0.03	0.4	0.5	-0.01
Coronary arteriosclerosis	0.1	0.1	0	0.1	0.1	0
Heart disease	1.7	1.1	0.06	1.7	2	-0.02
Heart failure	0.4	0.2	0.04	0.4	0.5	-0.01
Ischemic heart disease	0.4	0.3	0.03	0.4	0.5	-0.01
Peripheral vascular disease	0.1	0.1	0.01	0.1	0.1	0
Pulmonary embolism	0.2	0.1	0.03	0.2	0.2	0
Venous thrombosis	0.3	0.2	0.02	0.3	0.4	-0.02
**Medical history (neoplasms)**						
Malignant neoplastic disease	1	0.8	0.02	1	1.2	-0.02
Malignant tumor of breast	0.1	0.1	0.01	0.1	0.1	0
Malignant tumor of lung	0.1	0.1	0.01	0.1	0.1	-0.01

Table S6.110. *Continued.* Selected baseline characteristics for UK IMRD, for the short-term risk of alcohol misuse or dependence

Characteristic	Before propensity score matching			After propensity score matching		
	Targets, %	Comparators, %	Standardized difference	Targets, %	Comparators, %	Standardized difference
**Medication use**						
Agents acting on the renin-angiotensin system	12.6	12.2	0.01	12.6	12.8	-0.01
Antibacterials for systemic use	38.4	27.5	0.23	38.4	40.9	-0.05
Antidepressants	22.2	16	0.16	22.1	24.1	-0.05
Antiepileptics	5.9	4.3	0.07	5.9	6.6	-0.03
Antiinflammatory and antirheumatic products	20.5	16.4	0.11	20.5	21.6	-0.03
Antineoplastic agents	1	1	0	1	1	0
Antipsoriatics	4.5	3	0.08	4.5	4.9	-0.02
Antithrombotic agents	10.1	9.2	0.03	10.1	10.5	-0.01
Beta blocking agents	10	8.8	0.04	9.9	10.4	-0.02
Calcium channel blockers	8.3	8.4	0	8.2	8.3	0
Diuretics	6.9	6.1	0.03	6.8	7	-0.01
Drugs for acid-related disorders	25.8	20.7	0.12	25.7	27.8	-0.05
Drugs for obstructive airway diseases	23.7	19.1	0.11	23.7	25	-0.03
Drugs used in diabetes	6.5	4.8	0.07	6.4	6.7	-0.01
Immunosuppressants	1	0.9	0.01	1	1	0
Lipid modifying agents	13.9	14.3	-0.01	13.9	14.2	-0.01
Opioids	17.6	12.8	0.13	17.5	19.1	-0.04
Psycholeptics	10.3	7.7	0.09	10.2	11.5	-0.04
Psychostimulants, agents used for ADHD and nootropics	0.2	0.3	-0.01	0.2	0.3	-0.01

Table S6.111. Selected baseline characteristics for UK IMRD, for the short-term risk of substance misuse or dependence

Characteristic	Before propensity score matching			After propensity score matching		
	Targets, %	Comparators, %	Standardized difference	Targets, %	Comparators, %	Standardized difference
**Age group (years)**						
0-4	1.7	3.9	-0.13	1.7	1.7	0
5-9	2.3	5.3	-0.16	2.3	2.2	0.01
10-14	3.8	5.3	-0.07	3.8	3.7	0
20-24	7.3	5.3	0.09	7.4	7.8	-0.01
25-29	7.6	6.2	0.06	7.6	7.8	-0.01
30-34	7.7	6.9	0.03	7.7	7.6	0
35-39	8.1	7	0.04	8.1	8	0
40-44	7.6	6.4	0.05	7.7	7.5	0.01
45-49	8.1	6.7	0.05	8.1	8.2	0
50-54	8.9	7.4	0.05	8.9	9	0
55-59	8.4	7.4	0.04	8.4	8.5	-0.01
60-64	6	6.5	-0.02	6	6	0
65-69	3.6	5.6	-0.1	3.6	3.5	0
70-74	3.2	5.7	-0.12	3.2	3	0.01
75-79	2.8	4	-0.07	2.8	2.7	0.01
80-84	2.5	2.8	-0.02	2.5	2.5	0
85-89	2.1	1.8	0.02	2.1	2.2	-0.01
90-94	1.4	0.7	0.07	1.4	1.5	-0.01
95-99	0.6	0.2	0.06	0.6	0.6	0
**Sex**						
Female	56.7	49.4	0.15	56.7	58.6	-0.04

Table S6.111. *Continued.* Selected baseline characteristics for UK IMRD, for the short-term risk of substance misuse or dependence

Characteristic	Before propensity score matching			After propensity score matching		
	Targets, %	Comparators, %	Standardized difference	Targets, %	Comparators, %	Standardized difference
**Race**						
race = Asian	0.8	0.6	0.02	0.8	0.7	0.01
race = Black or African American	0.2	0.3	-0.02	0.2	0.2	0
race = White	4.5	6.6	-0.09	4.5	4.2	0.01
race = Asian Indian	0.9	0.9	0	0.9	0.9	0
race = Bangladeshi	0.2	0.1	0.01	0.2	0.1	0.01
race = Chinese	0.1	0.4	-0.06	0.1	0.1	0
race = Pakistani	1.7	0.6	0.1	1.7	1.8	0
race = African	0.7	0.8	-0.01	0.7	0.8	-0.01
race = West Indian	0.1	0.1	0	0.1	0.1	0
race = European	36.7	36.3	0.01	36.7	37.3	-0.01
race = Middle Eastern or North African	0.1	0.1	0	0.1	0.1	-0.01
race = Arab	0.1	0.1	0	0.1	0.1	0
**Medical history (general)**						
Acute respiratory disease	4.4	2.6	0.1	4.4	4.8	-0.02
Attention deficit hyperactivity disorder	0.1	0.1	0	0.1	0.1	0
Chronic obstructive lung disease	0.7	0.6	0.02	0.7	0.9	-0.02
Dementia	0.4	0.2	0.04	0.4	0.5	-0.01
Depressive disorder	1.4	1.1	0.04	1.4	1.6	-0.01
Diabetes mellitus	0.7	0.6	0.01	0.7	0.8	-0.01
Gastroesophageal reflux disease	0.3	0.2	0.03	0.3	0.4	-0.01
Gastrointestinal hemorrhage	0.5	0.4	0.02	0.5	0.6	-0.01
Hypertensive disorder	0.9	0.8	0.01	0.9	1	-0.01
Lesion of liver	0.1	0.1	0.01	0.1	0.1	-0.01

Table S6.111. *Continued.* Selected baseline characteristics for UK IMRD, for the short-term risk of substance misuse or dependence

Characteristic	Before propensity score matching			After propensity score matching		
	Targets, %	Comparators, %	Standardized difference	Targets, %	Comparators, %	Standardized difference
**Medical history (general)**						
Obesity	0.2	0.1	0.02	0.2	0.2	0
Osteoarthritis	0.8	0.7	0.01	0.8	0.8	0
Pneumonia	1.1	0.2	0.11	1	1.1	-0.01
Psoriasis	0.4	0.3	0.02	0.4	0.4	0
Renal impairment	1.1	0.5	0.07	1.1	1.2	-0.01
Rheumatoid arthritis	0.1	0.1	0	0.1	0.1	0
Urinary tract infectious disease	2.1	1	0.1	2.1	2.3	-0.02
**Medical history (cardiovascular disease)**						
Atrial fibrillation	0.6	0.4	0.04	0.6	0.7	-0.01
Cerebrovascular disease	0.4	0.3	0.03	0.4	0.5	-0.02
Coronary arteriosclerosis	0.1	0.1	0	0.1	0.1	0
Heart disease	1.7	1.1	0.05	1.7	2	-0.02
Heart failure	0.4	0.2	0.04	0.4	0.5	-0.01
Ischemic heart disease	0.4	0.3	0.02	0.4	0.5	-0.01
Peripheral vascular disease	0.1	0.1	0.01	0.1	0.1	0
Pulmonary embolism	0.2	0.1	0.03	0.2	0.2	0
Venous thrombosis	0.3	0.2	0.01	0.3	0.4	-0.02
**Medical history (neoplasms)**						
Malignant neoplastic disease	1	0.7	0.03	1	1.2	-0.02
Malignant tumor of breast	0.1	0.1	0	0.1	0.1	-0.01
Malignant tumor of lung	0.1	0.1	0.01	0.1	0.1	-0.01

Table S6.111. *Continued.* Selected baseline characteristics for UK IMRD, for the short-term risk of substance misuse or dependence

Characteristic	Before propensity score matching			After propensity score matching		
	Targets, %	Comparators, %	Standardized difference	Targets, %	Comparators, %	Standardized difference
**Medication use**						
Agents acting on the renin-angiotensin system	12.6	12.2	0.01	12.6	12.7	0
Antibacterials for systemic use	38.4	27.7	0.23	38.4	40.8	-0.05
Antidepressants	22.2	15.9	0.16	22	24	-0.05
Antiepileptics	5.9	4.3	0.08	5.9	6.6	-0.03
Antiinflammatory and antirheumatic products	20.5	16.5	0.1	20.5	21.6	-0.03
Antineoplastic agents	1	1	0	1	1	0
Antipsoriatics	4.5	2.9	0.08	4.5	4.9	-0.02
Antithrombotic agents	10.1	9.2	0.03	10.1	10.5	-0.01
Beta blocking agents	10	8.9	0.04	9.9	10.4	-0.02
Diuretics	6.9	6.1	0.03	6.8	7	-0.01
Drugs for acid-related disorders	25.8	20.6	0.12	25.7	27.8	-0.05
Drugs for obstructive airway diseases	23.7	19.1	0.11	23.7	25	-0.03
Drugs used in diabetes	6.5	4.9	0.07	6.4	6.7	-0.01
Immunosuppressants	1	0.9	0.01	1	1	0
Lipid modifying agents	13.9	14.3	-0.01	13.9	14.1	-0.01
Opioids	17.6	12.9	0.13	17.5	18.8	-0.04
Psycholeptics	10.3	7.6	0.09	10.2	11.4	-0.04
Psychostimulants, agents used for ADHD and nootropics	0.2	0.3	-0.01	0.2	0.3	-0.01

Table S6.112. Selected baseline characteristics for UK IMRD, for the short-term risk of bipolar disorders

Characteristic	Before propensity score matching			After propensity score matching		
	**Targets, %**	**Comparators, %**	**Standardized difference**	**Targets, %**	**Comparators, %**	**Standardized difference**
**Age group (years)**						
0-4	1.7	3.9	-0.14	1.7	1.6	0
5-9	2.3	5.2	-0.15	2.3	2.2	0.01
10-14	3.8	5.2	-0.07	3.8	3.8	0
15-19	6	4.9	0.05	6	5.9	0
20-24	7.3	5.1	0.09	7.4	7.7	-0.01
25-29	7.6	6.3	0.05	7.6	7.8	-0.01
30-34	7.7	6.9	0.03	7.7	7.7	0
35-39	8.1	7	0.04	8.1	8	0
40-44	7.6	6.5	0.04	7.7	7.5	0
45-49	8.1	6.8	0.05	8.1	8.2	0
50-54	8.9	7.3	0.06	8.9	9	0
55-59	8.4	7.4	0.04	8.4	8.5	0
60-64	6	6.5	-0.02	6	6	0
65-69	3.6	5.7	-0.1	3.6	3.5	0
70-74	3.2	5.6	-0.12	3.2	2.9	0.01
75-79	2.8	4	-0.07	2.8	2.7	0.01
85-89	2.1	1.7	0.03	2.1	2.2	-0.01
90-94	1.4	0.8	0.06	1.4	1.5	-0.01
95-99	0.6	0.2	0.06	0.6	0.6	0
**Sex**						
Female	56.7	49.3	0.15	56.7	58.5	-0.04

Table S6.112. *Continued.* Selected baseline characteristics for UK IMRD, for the short-term risk of bipolar disorders

Characteristic	Before propensity score matching			After propensity score matching		
	Targets, %	Comparators, %	Standardized difference	Targets, %	Comparators, %	Standardized difference
**Race**						
race = Asian	0.8	0.6	0.02	0.8	0.7	0.01
race = Black or African American	0.2	0.3	-0.02	0.2	0.2	0
race = White	4.5	6.7	-0.1	4.5	4.2	0.01
race = Asian Indian	0.9	0.9	0.01	0.9	0.9	0
race = Bangladeshi	0.2	0.1	0.02	0.2	0.2	0.01
race = Chinese	0.1	0.3	-0.06	0.1	0.1	0
race = Pakistani	1.7	0.6	0.11	1.7	1.8	0
race = African	0.7	0.9	-0.02	0.7	0.7	0
race = West Indian	0.1	0.1	-0.01	0.1	0.1	0
race = European	36.7	36.2	0.01	36.7	37.4	-0.01
race = Middle Eastern or North African	0.1	0.1	0	0.1	0.1	-0.01
race = Arab	0.1	0.1	0	0.1	0.1	0
**Medical history (general)**						
Acute respiratory disease	4.4	2.6	0.1	4.4	4.8	-0.02
Attention deficit hyperactivity disorder	0.1	0.1	0	0.1	0.1	0
Chronic obstructive lung disease	0.7	0.6	0.02	0.7	0.9	-0.02
Dementia	0.4	0.2	0.04	0.4	0.5	-0.01
Depressive disorder	1.4	1.1	0.04	1.4	1.6	-0.01
Diabetes mellitus	0.7	0.6	0.01	0.7	0.8	-0.01
Gastroesophageal reflux disease	0.3	0.2	0.02	0.3	0.4	-0.01
Gastrointestinal hemorrhage	0.5	0.4	0.02	0.5	0.6	-0.01
Hyperlipidemia	0.2	0.2	0	0.2	0.2	0
Hypertensive disorder	0.9	0.8	0.01	0.9	1	-0.01

Table S6.112. *Continued.* Selected baseline characteristics for UK IMRD, for the short-term risk of bipolar disorders

Characteristic	Before propensity score matching			After propensity score matching		
	Targets, %	Comparators, %	Standardized difference	Targets, %	Comparators, %	Standardized difference
**Medical history (general)**						
Lesion of liver	0.1	0.1	0.01	0.1	0.2	-0.01
Obesity	0.2	0.1	0.02	0.2	0.2	0
Osteoarthritis	0.8	0.7	0.01	0.8	0.9	0
Pneumonia	1.1	0.2	0.11	1	1.1	-0.01
Psoriasis	0.4	0.3	0.02	0.4	0.4	-0.01
Renal impairment	1.1	0.5	0.07	1.1	1.2	-0.01
Rheumatoid arthritis	0.1	0.1	0.01	0.1	0.1	0
Urinary tract infectious disease	2.1	1	0.09	2.1	2.4	-0.02
**Medical history (cardiovascular disease)**						
Atrial fibrillation	0.6	0.3	0.04	0.6	0.7	-0.02
Cerebrovascular disease	0.4	0.3	0.03	0.4	0.5	-0.02
Coronary arteriosclerosis	0.1	0.1	0	0.1	0.1	0
Heart disease	1.7	1.1	0.06	1.7	2	-0.02
Heart failure	0.4	0.2	0.04	0.4	0.5	-0.01
Ischemic heart disease	0.4	0.3	0.02	0.4	0.5	-0.01
Peripheral vascular disease	0.1	0.1	0.01	0.1	0.1	0
Pulmonary embolism	0.2	0.1	0.03	0.2	0.2	0
Venous thrombosis	0.3	0.2	0.02	0.3	0.4	-0.02
**Medical history (neoplasms)**						
Malignant neoplastic disease	1	0.8	0.02	1	1.2	-0.02
Malignant tumor of breast	0.1	0.1	0	0.1	0.1	-0.01

Table S6.112. *Continued.* Selected baseline characteristics for UK IMRD, for the short-term risk of bipolar disorders

Characteristic	Before propensity score matching			After propensity score matching		
	Targets, %	Comparators, %	Standardized difference	Targets, %	Comparators, %	Standardized difference
**Medication use**						
Agents acting on the renin-angiotensin system	12.6	12.1	0.01	12.6	12.8	-0.01
Antibacterials for systemic use	38.4	27.6	0.23	38.4	40.8	-0.05
Antidepressants	22.2	15.9	0.16	22.1	24	-0.05
Antiepileptics	5.9	4.3	0.07	5.9	6.6	-0.03
Antiinflammatory and antirheumatic products	20.5	16.3	0.11	20.5	21.6	-0.03
Antineoplastic agents	1	1	0	1	1	0
Antipsoriatics	4.5	3	0.08	4.5	4.9	-0.02
Antithrombotic agents	10.1	9	0.04	10.1	10.6	-0.02
Beta blocking agents	10	8.8	0.04	9.9	10.4	-0.02
Calcium channel blockers	8.3	8.2	0	8.2	8.2	0
Diuretics	6.9	6	0.03	6.8	7	-0.01
Drugs for acid-related disorders	25.8	20.7	0.12	25.7	27.8	-0.05
Drugs for obstructive airway diseases	23.7	19.1	0.11	23.7	25	-0.03
Drugs used in diabetes	6.5	4.9	0.07	6.4	6.7	-0.01
Immunosuppressants	1	0.9	0.01	1	1	0
Lipid modifying agents	13.9	14.2	-0.01	13.9	14.2	-0.01
Opioids	17.6	12.8	0.13	17.5	18.9	-0.04
Psycholeptics	10.3	7.7	0.09	10.2	11.4	-0.04
Psychostimulants, agents used for ADHD and nootropics	0.2	0.3	-0.02	0.2	0.3	-0.01

Table S6.113. Selected baseline characteristics for UK IMRD, for the short-term risk of psychoses

	**Before propensity score matching**			**After propensity score matching**		
	**Targets, %**	**Comparators, %**	**Standardized difference**	**Targets, %**	**Comparators, %**	**Standardized difference**
**Age group (years)**						
0-4	1.7	3.9	-0.14	1.7	1.7	0
5-9	2.3	5.2	-0.15	2.3	2.2	0.01
10-14	3.8	5.2	-0.07	3.8	3.7	0
20-24	7.3	5.2	0.09	7.4	7.7	-0.01
25-29	7.6	6.3	0.05	7.6	7.9	-0.01
30-34	7.7	7	0.03	7.8	7.7	0
35-39	8.1	6.9	0.05	8.2	8.1	0
40-44	7.6	6.5	0.04	7.7	7.5	0
50-54	8.9	7.4	0.05	8.9	9	0
55-59	8.4	7.4	0.04	8.4	8.5	0
60-64	6	6.4	-0.02	6	6	0
65-69	3.6	5.8	-0.1	3.6	3.5	0
70-74	3.2	5.7	-0.12	3.2	3	0.01
75-79	2.8	4	-0.06	2.8	2.7	0.01
80-84	2.5	2.8	-0.02	2.5	2.4	0
85-89	2.1	1.7	0.03	2.1	2.2	-0.01
90-94	1.4	0.7	0.06	1.4	1.5	-0.01
95-99	0.6	0.2	0.06	0.6	0.6	0
**Sex**						
Female	56.7	49.3	0.15	56.7	58.4	-0.04

Table S6.113. *Continued.* Selected baseline characteristics for UK IMRD, for the short-term risk of psychoses

Characteristic	Before propensity score matching			After propensity score matching		
	Targets, %	Comparators, %	Standardized difference	Targets, %	Comparators, %	Standardized difference
**Race**						
race = Asian	0.8	0.6	0.02	0.8	0.7	0.01
race = Black or African American	0.2	0.3	-0.01	0.2	0.2	0
race = White	4.5	6.7	-0.1	4.5	4.2	0.01
race = Asian Indian	0.9	0.9	0.01	0.9	0.9	0
race = Bangladeshi	0.2	0.1	0.01	0.2	0.2	0.01
race = Pakistani	1.7	0.6	0.11	1.7	1.8	0
race = African	0.7	0.9	-0.02	0.7	0.7	-0.01
race = West Indian	0.1	0.1	0	0.1	0.1	0
race = European	36.7	36.1	0.01	36.7	37.3	-0.01
race = Middle Eastern or North African	0.1	0.1	0	0.1	0.1	-0.01
race = Arab	0.1	0.1	0	0.1	0.1	0
**Medical history (general)**						
Acute respiratory disease	4.4	2.7	0.09	4.4	4.8	-0.02
Attention deficit hyperactivity disorder	0.1	0.1	0	0.1	0.1	0
Chronic obstructive lung disease	0.7	0.6	0.01	0.7	0.9	-0.02
Dementia	0.4	0.2	0.04	0.4	0.5	-0.01
Depressive disorder	1.4	1	0.04	1.4	1.6	-0.01
Diabetes mellitus	0.7	0.6	0.01	0.7	0.8	-0.01
Gastroesophageal reflux disease	0.3	0.2	0.03	0.3	0.4	-0.01
Gastrointestinal hemorrhage	0.5	0.4	0.02	0.5	0.6	-0.01
Hyperlipidemia	0.2	0.2	0	0.2	0.2	0
Hypertensive disorder	0.9	0.8	0.01	0.9	1	-0.01
Lesion of liver	0.1	0.1	0.01	0.1	0.2	-0.02
Obesity	0.2	0.1	0.02	0.2	0.2	0

Table S6.113. *Continued.* Selected baseline characteristics for UK IMRD, for the short-term risk of psychoses

Characteristic	Before propensity score matching			After propensity score matching		
	**Targets, %**	**Comparators, %**	**Standardized difference**	**Targets, %**	**Comparators, %**	**Standardized difference**
**Medical history (general)**						
Osteoarthritis	0.8	0.8	0.01	0.8	0.8	0
Pneumonia	1.1	0.2	0.11	1	1.1	-0.01
Psoriasis	0.4	0.3	0.02	0.4	0.4	-0.01
Renal impairment	1.1	0.5	0.07	1.1	1.2	-0.01
Rheumatoid arthritis	0.1	0.1	0	0.1	0.1	0
Urinary tract infectious disease	2.1	1	0.09	2.1	2.3	-0.02
**Medical history (cardiovascular disease)**						
Atrial fibrillation	0.6	0.3	0.04	0.6	0.7	-0.01
Cerebrovascular disease	0.4	0.2	0.03	0.4	0.5	-0.02
Heart disease	1.7	1	0.06	1.7	2	-0.02
Heart failure	0.4	0.2	0.04	0.4	0.5	-0.01
Ischemic heart disease	0.4	0.3	0.02	0.4	0.5	-0.01
Peripheral vascular disease	0.1	0.1	0.01	0.1	0.1	0
Pulmonary embolism	0.2	0.1	0.03	0.2	0.2	0
Venous thrombosis	0.3	0.2	0.02	0.3	0.4	-0.02
**Medical history (neoplasms)**						
Malignant neoplastic disease	1	0.8	0.02	1	1.2	-0.02
Malignant tumor of breast	0.1	0.1	0	0.1	0.1	-0.01
Malignant tumor of lung	0.1	0.1	0.01	0.1	0.1	-0.01

Table S6.113. *Continued.* Selected baseline characteristics for UK IMRD, for the short-term risk of psychoses

Characteristic	Before propensity score matching			After propensity score matching		
	**Targets, %**	**Comparators, %**	**Standardized difference**	**Targets, %**	**Comparators, %**	**Standardized difference**
**Medication use**						
Agents acting on the renin-angiotensin system	12.6	12.3	0.01	12.6	12.8	-0.01
Antibacterials for systemic use	38.4	27.8	0.23	38.3	40.8	-0.05
Antidepressants	22.2	15.9	0.16	22.1	24	-0.04
Antiepileptics	5.9	4.2	0.08	5.9	6.5	-0.02
Antiinflammatory and antirheumatic products	20.5	16.3	0.11	20.5	21.6	-0.03
Antineoplastic agents	1	1	0	1	1	0
Antipsoriatics	4.5	3	0.08	4.5	4.9	-0.02
Antithrombotic agents	10.1	9.1	0.04	10.1	10.5	-0.01
Beta blocking agents	10	8.8	0.04	9.9	10.4	-0.02
Calcium channel blockers	8.3	8.4	0	8.2	8.3	0
Diuretics	6.9	6.1	0.03	6.8	7	-0.01
Drugs for acid-related disorders	25.8	20.6	0.12	25.7	27.8	-0.05
Drugs for obstructive airway diseases	23.7	19	0.11	23.7	24.9	-0.03
Drugs used in diabetes	6.5	4.9	0.07	6.4	6.7	-0.01
Immunosuppressants	1	0.9	0.01	1	1	0
Lipid modifying agents	13.9	14.3	-0.01	13.8	14.2	-0.01
Opioids	17.6	12.8	0.13	17.5	19	-0.04
Psycholeptics	10.3	7.6	0.09	10.2	11.3	-0.04
Psychostimulants, agents used for ADHD and nootropics	0.2	0.3	-0.01	0.2	0.3	-0.01

Table S6.114. Selected baseline characteristics for UK IMRD, for the short-term risk of personality disorders

Characteristic	Before propensity score matching			After propensity score matching		
	Targets, %	Comparators, %	Standardized difference	Targets, %	Comparators, %	Standardized difference
**Age group (years)**						
0-4	1.7	3.9	-0.14	1.7	1.7	0
5-9	2.3	5.2	-0.15	2.3	2.2	0.01
10-14	3.8	5.2	-0.07	3.8	3.8	0
15-19	6	4.9	0.05	6	5.9	0
20-24	7.3	5.1	0.09	7.3	7.8	-0.02
25-29	7.6	6.2	0.06	7.6	7.8	-0.01
30-34	7.7	7	0.03	7.8	7.7	0
35-39	8.1	6.9	0.05	8.2	8	0
40-44	7.6	6.5	0.04	7.7	7.5	0.01
45-49	8.1	6.8	0.05	8.1	8.3	-0.01
55-59	8.4	7.4	0.04	8.4	8.5	0
60-64	6	6.5	-0.02	6	6	0
65-69	3.6	5.6	-0.1	3.6	3.5	0
70-74	3.2	5.6	-0.12	3.2	2.9	0.01
75-79	2.8	4	-0.06	2.8	2.7	0.01
80-84	2.5	2.9	-0.02	2.5	2.4	0
85-89	2.1	1.8	0.02	2.1	2.2	-0.01
90-94	1.4	0.7	0.07	1.4	1.5	-0.01
95-99	0.6	0.2	0.06	0.6	0.6	0
**Sex**						
Female	56.7	49.4	0.14	56.6	58.5	-0.04

Table S6.114. *Continued.* Selected baseline characteristics for UK IMRD, for the short-term risk of personality disorders

Characteristic	Before propensity score matching			After propensity score matching		
	Targets, %	Comparators, %	Standardized difference	Targets, %	Comparators, %	Standardized difference
**Race**						
race = Asian	0.8	0.7	0.01	0.8	0.7	0.01
race = Black or African American	0.2	0.3	-0.02	0.2	0.2	0
race = White	4.5	6.6	-0.09	4.5	4.2	0.01
race = Asian Indian	0.9	0.9	0	0.9	0.9	0
race = Bangladeshi	0.2	0.1	0.02	0.2	0.1	0.01
race = Chinese	0.1	0.3	-0.06	0.1	0.1	0
race = Pakistani	1.7	0.6	0.11	1.7	1.8	0
race = African	0.7	0.9	-0.02	0.7	0.7	-0.01
race = West Indian	0.1	0.1	-0.01	0.1	0.1	0
race = European	36.7	36.3	0.01	36.7	37.4	-0.01
race = Arab	0.1	0.1	0	0.1	0.1	0
**Medical history (general)**						
Acute respiratory disease	4.4	2.6	0.09	4.4	4.8	-0.02
Attention deficit hyperactivity disorder	0.1	0.1	0.01	0.1	0.1	0
Chronic obstructive lung disease	0.7	0.6	0.02	0.7	0.9	-0.02
Dementia	0.4	0.2	0.04	0.4	0.5	-0.02
Depressive disorder	1.4	1.1	0.04	1.4	1.6	-0.01
Diabetes mellitus	0.7	0.6	0.01	0.7	0.8	-0.01
Gastroesophageal reflux disease	0.3	0.2	0.02	0.3	0.4	-0.01
Gastrointestinal hemorrhage	0.5	0.4	0.02	0.5	0.6	-0.01
Hypertensive disorder	0.9	0.8	0.01	0.9	1	-0.01
Lesion of liver	0.1	0.1	0.01	0.1	0.2	-0.01
Obesity	0.2	0.1	0.02	0.2	0.2	0

Table S6.114. *Continued.* Selected baseline characteristics for UK IMRD, for the short-term risk of personality disorders

Characteristic	Before propensity score matching			After propensity score matching		
	Targets, %	Comparators, %	Standardized difference	Targets, %	Comparators, %	Standardized difference
**Medical history (general)**						
Osteoarthritis	0.8	0.7	0.01	0.8	0.8	0
Pneumonia	1.1	0.2	0.11	1	1.1	-0.01
Psoriasis	0.4	0.3	0.01	0.4	0.4	-0.01
Renal impairment	1.1	0.5	0.06	1.1	1.2	-0.01
Rheumatoid arthritis	0.1	0.1	0	0.1	0.1	0
Urinary tract infectious disease	2.1	1	0.1	2.1	2.3	-0.01
**Medical history (cardiovascular disease)**						
Atrial fibrillation	0.6	0.3	0.04	0.6	0.7	-0.01
Cerebrovascular disease	0.4	0.3	0.03	0.4	0.5	-0.02
Coronary arteriosclerosis	0.1	0.1	0	0.1	0.1	0
Heart disease	1.7	1.1	0.06	1.7	2	-0.02
Heart failure	0.4	0.2	0.04	0.4	0.5	-0.01
Ischemic heart disease	0.4	0.3	0.02	0.4	0.5	-0.01
Peripheral vascular disease	0.1	0.1	0.01	0.1	0.1	0
Pulmonary embolism	0.2	0.1	0.03	0.2	0.2	0
Venous thrombosis	0.3	0.2	0.02	0.3	0.4	-0.02
**Medical history (neoplasms)**						
Malignant neoplastic disease	1	0.8	0.02	1	1.2	-0.02
Malignant tumor of breast	0.1	0.1	0	0.1	0.1	-0.01
Malignant tumor of lung	0.1	0.1	0.01	0.1	0.1	-0.01

Table S6.114. *Continued.* Selected baseline characteristics for UK IMRD, for the short-term risk of personality disorders

Characteristic	Before propensity score matching			After propensity score matching		
	**Targets, %**	**Comparators, %**	**Standardized difference**	**Targets, %**	**Comparators, %**	**Standardized difference**
**Medication use**						
Agents acting on the renin-angiotensin system	12.6	12.2	0.01	12.6	12.8	-0.01
Antibacterials for systemic use	38.4	27.6	0.23	38.3	40.8	-0.05
Antidepressants	22.2	15.8	0.16	22.1	24.1	-0.05
Antiepileptics	5.9	4.3	0.07	5.9	6.6	-0.03
Antiinflammatory and antirheumatic products	20.5	16.4	0.11	20.5	21.6	-0.03
Antineoplastic agents	1	1	-0.01	1	1	0
Antipsoriatics	4.5	3	0.08	4.5	4.9	-0.02
Antithrombotic agents	10.1	9.1	0.03	10.1	10.6	-0.01
Beta blocking agents	10	8.8	0.04	9.9	10.4	-0.02
Diuretics	6.9	6.1	0.03	6.8	7.1	-0.01
Drugs for acid-related disorders	25.8	20.5	0.12	25.7	27.8	-0.05
Drugs for obstructive airway diseases	23.7	19	0.12	23.7	25	-0.03
Drugs used in diabetes	6.5	4.8	0.07	6.4	6.7	-0.01
Immunosuppressants	1	0.9	0.01	1	1	0
Lipid modifying agents	13.9	14.2	-0.01	13.9	14.1	-0.01
Opioids	17.6	12.7	0.14	17.6	19	-0.04
Psycholeptics	10.3	7.7	0.09	10.2	11.4	-0.04
Psychostimulants, agents used for ADHD and nootropics	0.2	0.3	-0.01	0.2	0.3	-0.01

Table S6.115. Selected baseline characteristics for UK IMRD, for the short-term risk of self-harm and suicide

Characteristic	Before propensity score matching			After propensity score matching		
	Targets, %	Comparators, %	Standardized difference	Targets, %	Comparators, %	Standardized difference
**Age group (years)**						
0-4	1.7	3.9	-0.14	1.7	1.7	0
5-9	2.3	5.3	-0.16	2.3	2.2	0.01
10-14	3.8	5.3	-0.07	3.8	3.8	0
15-19	6	4.9	0.05	6	5.9	0
20-24	7.3	5.2	0.09	7.4	7.7	-0.01
25-29	7.6	6.2	0.06	7.6	7.9	-0.01
30-34	7.7	7	0.03	7.7	7.7	0
35-39	8.1	7	0.04	8.1	8.1	0
40-44	7.6	6.5	0.04	7.7	7.5	0
45-49	8.1	6.7	0.05	8.2	8.2	0
50-54	8.9	7.3	0.06	8.9	9	0
55-59	8.4	7.4	0.04	8.4	8.5	0
60-64	6	6.5	-0.02	6	6	0
65-69	3.6	5.6	-0.1	3.6	3.5	0
70-74	3.2	5.6	-0.12	3.2	2.9	0.01
75-79	2.8	4.1	-0.07	2.8	2.7	0.01
80-84	2.5	2.9	-0.02	2.5	2.5	0
85-89	2.1	1.7	0.03	2.1	2.1	0
90-94	1.4	0.8	0.06	1.4	1.5	-0.01
**Sex**						
Female	56.7	49.4	0.14	56.6	58.4	-0.04

Table S6.115. *Continued.* Selected baseline characteristics for UK IMRD, for the short-term risk of self-harm and suicide

Characteristic	Before propensity score matching			After propensity score matching		
	Targets, %	Comparators, %	Standardized difference	Targets, %	Comparators, %	Standardized difference
**Race**						
race = Asian	0.8	0.6	0.02	0.8	0.7	0.01
race = Black or African American	0.2	0.3	-0.02	0.2	0.2	0
race = White	4.5	6.7	-0.1	4.5	4.2	0.01
race = Asian Indian	0.9	0.9	0.01	0.9	0.9	0
race = Bangladeshi	0.2	0.1	0.01	0.2	0.1	0.01
race = Chinese	0.1	0.4	-0.06	0.1	0.1	0
race = Pakistani	1.7	0.6	0.11	1.7	1.8	0
race = African	0.7	0.9	-0.02	0.7	0.8	-0.01
race = West Indian	0.1	0.1	0	0.1	0.1	0
race = European	36.7	36.2	0.01	36.7	37.4	-0.01
race = Middle Eastern or North African	0.1	0.1	0	0.1	0.1	0
race = Arab	0.1	0.1	0	0.1	0.1	0
**Medical history (general)**						
Acute respiratory disease	4.4	2.7	0.09	4.4	4.8	-0.02
Attention deficit hyperactivity disorder	0.1	0.1	0	0.1	0.1	0
Chronic obstructive lung disease	0.7	0.6	0.02	0.7	0.9	-0.02
Dementia	0.4	0.2	0.04	0.4	0.5	-0.01
Depressive disorder	1.4	1	0.04	1.4	1.6	-0.01
Diabetes mellitus	0.7	0.6	0.02	0.7	0.8	-0.01
Gastroesophageal reflux disease	0.3	0.2	0.03	0.3	0.4	0
Gastrointestinal hemorrhage	0.5	0.4	0.02	0.5	0.6	-0.01
Hyperlipidemia	0.2	0.2	0	0.2	0.2	0
Hypertensive disorder	0.9	0.7	0.01	0.9	1	-0.01

Table S6.115. *Continued.* Selected baseline characteristics for UK IMRD, for the short-term risk of self-harm and suicide

Characteristic	Before propensity score matching			After propensity score matching		
	Targets, %	Comparators, %	Standardized difference	Targets, %	Comparators, %	Standardized difference
**Medical history (general)**						
Lesion of liver	0.1	0.1	0.01	0.1	0.2	-0.01
Obesity	0.2	0.1	0.02	0.2	0.2	0
Osteoarthritis	0.8	0.7	0.01	0.8	0.8	0
Pneumonia	1.1	0.2	0.11	1	1.1	-0.01
Psoriasis	0.4	0.3	0.01	0.4	0.4	0
Renal impairment	1.1	0.5	0.07	1.1	1.2	-0.01
Rheumatoid arthritis	0.1	0.1	0.01	0.1	0.1	0
Urinary tract infectious disease	2.1	1	0.09	2.1	2.3	-0.01
**Medical history (cardiovascular disease)**						
Atrial fibrillation	0.6	0.4	0.04	0.6	0.7	-0.01
Cerebrovascular disease	0.4	0.2	0.03	0.4	0.5	-0.02
Heart disease	1.7	1.1	0.06	1.7	2	-0.02
Heart failure	0.4	0.2	0.04	0.4	0.5	-0.01
Ischemic heart disease	0.4	0.3	0.03	0.4	0.5	-0.01
Peripheral vascular disease	0.1	0.1	0.01	0.1	0.1	0
Pulmonary embolism	0.2	0.1	0.03	0.2	0.2	0
Venous thrombosis	0.3	0.2	0.02	0.3	0.4	-0.02
**Medical history (neoplasms)**						
Malignant neoplastic disease	1	0.8	0.02	1	1.2	-0.02
Malignant tumor of breast	0.1	0.1	0	0.1	0.1	-0.01
Malignant tumor of lung	0.1	0.1	0.01	0.1	0.1	-0.01

Table S6.115. *Continued.* Selected baseline characteristics for UK IMRD, for the short-term risk of self-harm and suicide

Characteristic	Before propensity score matching			After propensity score matching		
	Targets, %	Comparators, %	Standardized difference	Targets, %	Comparators, %	Standardized difference
**Medication use**						
Agents acting on the renin-angiotensin system	12.6	12.1	0.01	12.6	12.9	-0.01
Antibacterials for systemic use	38.4	27.7	0.23	38.3	40.8	-0.05
Antidepressants	22.2	15.9	0.16	22.1	24.1	-0.05
Antiepileptics	5.9	4.3	0.08	5.9	6.6	-0.03
Antiinflammatory and antirheumatic products	20.5	16.4	0.11	20.5	21.6	-0.03
Antineoplastic agents	1	1	0	1	1	0
Antipsoriatics	4.5	3	0.08	4.5	4.9	-0.02
Antithrombotic agents	10.1	9.1	0.04	10.1	10.5	-0.01
Beta blocking agents	10	8.8	0.04	9.9	10.4	-0.02
Calcium channel blockers	8.3	8.4	0	8.2	8.3	0
Diuretics	6.9	6	0.04	6.8	7	-0.01
Drugs for acid-related disorders	25.8	20.7	0.12	25.7	27.9	-0.05
Drugs for obstructive airway diseases	23.7	19.1	0.11	23.7	25	-0.03
Drugs used in diabetes	6.5	4.9	0.07	6.4	6.7	-0.01
Immunosuppressants	1	0.9	0.01	1	1	0
Lipid modifying agents	13.9	14.3	-0.01	13.9	14.1	-0.01
Opioids	17.6	12.8	0.13	17.5	19	-0.04
Psycholeptics	10.3	7.6	0.09	10.2	11.4	-0.04
Psychostimulants, agents used for ADHD and nootropics	0.2	0.3	-0.01	0.2	0.3	-0.01

Table S6.116. Selected baseline characteristics for UK IMRD, for the short-term risk of sleep disorders

Characteristic	Before propensity score matching			After propensity score matching		
	**Targets, %**	**Comparators, %**	**Standardized difference**	**Targets, %**	**Comparators, %**	**Standardized difference**
**Age group (years)**						
0-4	1.7	3.9	-0.14	1.7	1.7	0
5-9	2.3	5.2	-0.15	2.3	2.2	0.01
10-14	6	5	0.04	6	6	0
20-24	7.3	5.1	0.09	7.4	7.8	-0.02
25-29	7.6	6.2	0.05	7.6	7.9	-0.01
30-34	7.7	7	0.03	7.7	7.7	0
35-39	8.1	7	0.04	8.2	8.1	0
40-44	7.6	6.5	0.04	7.7	7.5	0
45-49	8.1	6.7	0.05	8.1	8.2	0
50-54	8.9	7.4	0.05	8.9	8.9	0
55-59	8.4	7.3	0.04	8.4	8.5	0
60-64	6	6.5	-0.02	6	6	0
65-69	3.6	5.6	-0.1	3.6	3.5	0
70-74	3.2	5.6	-0.12	3.2	2.9	0.01
75-79	2.8	4	-0.07	2.8	2.6	0.01
80-84	2.5	2.9	-0.02	2.5	2.4	0
85-89	2.1	1.7	0.03	2.1	2.2	-0.01
90-94	1.4	0.8	0.06	1.4	1.5	-0.01
95-99	0.6	0.2	0.06	0.6	0.6	0
**Sex**						
Female	56.7	49.7	0.14	56.6	58.5	-0.04

Table S6.116. *Continued.* Selected baseline characteristics for UK IMRD, for the short-term risk of sleep disorders

Characteristic	Before propensity score matching			After propensity score matching		
	Targets, %	Comparators, %	Standardized difference	Targets, %	Comparators, %	Standardized difference
**Race**						
race = Asian	0.8	0.7	0.01	0.8	0.7	0.01
race = Black or African American	0.2	0.3	-0.02	0.2	0.2	0
race = White	4.5	6.6	-0.09	4.5	4.2	0.01
race = Asian Indian	0.9	0.9	0.01	0.9	1	0
race = Bangladeshi	0.2	0.1	0.02	0.2	0.1	0.01
race = Chinese	0.1	0.4	-0.06	0.1	0.1	0
race = Pakistani	1.7	0.6	0.1	1.7	1.8	0
race = African	0.7	0.8	-0.02	0.7	0.7	-0.01
race = West Indian	0.1	0.1	-0.01	0.1	0.1	-0.01
race = European	36.7	36.3	0.01	36.7	37.4	-0.01
race = Middle Eastern or North African	0.1	0.1	0	0.1	0.1	-0.01
**Medical history (general)**						
Acute respiratory disease	4.4	2.6	0.1	4.3	4.7	-0.02
Attention deficit hyperactivity disorder	0.1	0.1	0	0.1	0.1	0
Chronic obstructive lung disease	0.7	0.6	0.02	0.7	0.9	-0.02
Dementia	0.4	0.2	0.04	0.4	0.5	-0.01
Depressive disorder	1.4	1	0.04	1.4	1.6	-0.01
Diabetes mellitus	0.7	0.6	0.02	0.7	0.8	-0.01
Gastroesophageal reflux disease	0.3	0.2	0.03	0.3	0.4	-0.01
Gastrointestinal hemorrhage	0.5	0.4	0.02	0.5	0.6	-0.01
Hyperlipidemia	0.2	0.2	0	0.2	0.2	0
Hypertensive disorder	0.9	0.8	0.01	0.9	1	-0.01
Lesion of liver	0.1	0.1	0.01	0.1	0.2	-0.01

Table S6.116. *Continued.* Selected baseline characteristics for UK IMRD, for the short-term risk of sleep disorders

Characteristic	Before propensity score matching			After propensity score matching		
	Targets, %	Comparators, %	Standardized difference	Targets, %	Comparators, %	Standardized difference
**Medical history (general)**						
Obesity	0.2	0.1	0.02	0.2	0.2	0
Osteoarthritis	0.8	0.7	0.01	0.8	0.8	0
Pneumonia	1.1	0.2	0.11	1	1.1	0
Psoriasis	0.4	0.3	0.02	0.4	0.4	-0.01
Renal impairment	1.1	0.5	0.07	1.1	1.2	-0.01
Rheumatoid arthritis	0.1	0.1	0.01	0.1	0.1	0
Urinary tract infectious disease	2.1	1	0.09	2.1	2.3	-0.01
**Medical history (cardiovascular disease)**						
Atrial fibrillation	0.6	0.3	0.04	0.6	0.7	-0.02
Cerebrovascular disease	0.4	0.3	0.03	0.4	0.5	-0.01
Coronary arteriosclerosis	0.1	0.1	0	0.1	0.1	0
Heart disease	1.7	1.1	0.06	1.7	2	-0.02
Heart failure	0.4	0.2	0.04	0.4	0.4	-0.01
Ischemic heart disease	0.4	0.3	0.02	0.4	0.5	-0.01
Peripheral vascular disease	0.1	0.1	0.01	0.1	0.1	0
Pulmonary embolism	0.2	0.1	0.03	0.2	0.2	0
Venous thrombosis	0.3	0.2	0.02	0.3	0.4	-0.02
**Medical history (neoplasms)**						
Malignant neoplastic disease	1	0.8	0.02	1	1.2	-0.02
Malignant tumor of breast	0.1	0.1	0	0.1	0.1	-0.01
Malignant tumor of lung	0.1	0.1	0.01	0.1	0.1	-0.01

Table S6.116. *Continued.* Selected baseline characteristics for UK IMRD, for the short-term risk of sleep disorders

Characteristic	Before propensity score matching			After propensity score matching		
	Targets, %	Comparators, %	Standardized difference	Targets, %	Comparators, %	Standardized difference
**Medication use**						
Agents acting on the renin-angiotensin system	12.6	12.3	0.01	12.5	12.8	-0.01
Antibacterials for systemic use	38.4	27.7	0.23	38.3	40.7	-0.05
Antidepressants	22.2	15.9	0.16	21.9	23.8	-0.04
Antiepileptics	5.9	4.4	0.07	5.9	6.5	-0.03
Antiinflammatory and antirheumatic products	20.5	16.3	0.11	20.4	21.5	-0.03
Antineoplastic agents	1	0.9	0	1	1	0
Antipsoriatics	4.5	3	0.08	4.5	4.8	-0.02
Antithrombotic agents	10.1	9.1	0.03	10	10.4	-0.01
Beta blocking agents	10	8.9	0.04	9.8	10.3	-0.02
Calcium channel blockers	8.3	8.3	0	8.2	8.2	0
Diuretics	6.9	6.2	0.03	6.8	7	-0.01
Drugs for acid-related disorders	25.8	20.6	0.12	25.6	27.6	-0.05
Drugs for obstructive airway diseases	23.7	19.1	0.11	23.5	24.9	-0.03
Drugs used in diabetes	6.5	4.9	0.07	6.4	6.6	-0.01
Immunosuppressants	1	0.9	0.01	1	1	0
Lipid modifying agents	13.9	14.2	-0.01	13.8	14	-0.01
Opioids	17.6	12.9	0.13	17.4	18.8	-0.04
Psycholeptics	10.3	7.8	0.09	10	11.1	-0.04
Psychostimulants, agents used for ADHD and nootropics	0.2	0.3	-0.01	0.2	0.3	-0.01

Table S6.117. *S*elected baseline characteristics for UK IMRD, for the short-term risk of dementia

Characteristic	Before propensity score matching			After propensity score matching		
	Targets, %	Comparators, %	Standardized difference	Targets, %	Comparators, %	Standardized difference
**Age group (years)**						
0-4	1.7	3.9	-0.14	1.7	1.7	0
5-9	2.3	5.2	-0.15	2.4	2.3	0.01
10-14	3.8	5.3	-0.07	3.9	3.8	0
15-19	6	4.9	0.04	6.1	6	0
20-24	7.3	5.2	0.09	7.5	7.8	-0.01
25-29	7.6	6.3	0.05	7.7	8	-0.01
30-34	7.7	6.9	0.03	7.9	7.8	0
35-39	8.1	6.9	0.05	8.3	8.1	0
40-44	7.6	6.5	0.04	7.8	7.7	0
45-49	8.1	6.7	0.05	8.3	8.3	0
50-54	8.9	7.4	0.06	9.1	9.1	0
55-59	8.4	7.4	0.04	8.5	8.6	0
60-64	6	6.5	-0.02	6.1	6	0
65-69	3.6	5.6	-0.1	3.6	3.5	0
70-74	3.2	5.7	-0.12	3.1	2.9	0.01
75-79	2.8	4.1	-0.07	2.6	2.5	0.01
80-84	2.5	2.8	-0.02	2.2	2.1	0
85-89	2.1	1.7	0.03	1.8	1.8	0
90-94	1.4	0.7	0.06	1.2	1.2	0
95-99	0.6	0.2	0.06	0.5	0.5	0
**Sex**						
Female	56.7	49.2	0.15	56.6	58.5	-0.04

Table S6.117. *Continued.* Selected baseline characteristics for UK IMRD, for the short-term risk of dementia

Characteristic	Before propensity score matching			After propensity score matching		
	Targets, %	Comparators, %	Standardized difference	Targets, %	Comparators, %	Standardized difference
**Race**						
race = Asian	0.8	0.6	0.02	0.8	0.7	0.01
race = Black or African American	0.2	0.3	-0.02	0.2	0.2	0
race = White	4.5	6.7	-0.1	4.5	4.2	0.01
race = Asian Indian	0.9	0.9	0.01	1	0.9	0
race = Bangladeshi	0.2	0.1	0.02	0.2	0.1	0.01
race = Chinese	0.1	0.3	-0.06	0.1	0.1	0
race = Pakistani	1.7	0.6	0.11	1.7	1.8	0
race = African	0.7	0.9	-0.02	0.7	0.8	-0.01
race = West Indian	0.1	0.1	-0.01	0.1	0.1	-0.01
race = European	36.7	36.2	0.01	36.7	37.3	-0.01
race = Middle Eastern or North African	0.1	0.1	0	0.1	0.1	-0.01
**Medical history (general)**						
Acute respiratory disease	4.4	2.6	0.1	4.3	4.8	-0.02
Attention deficit hyperactivity disorder	0.1	0.1	0	0.1	0.1	0
Chronic obstructive lung disease	0.7	0.6	0.01	0.7	0.9	-0.02
Depressive disorder	1.4	1	0.04	1.4	1.6	-0.01
Diabetes mellitus	0.7	0.6	0.01	0.7	0.8	-0.01
Gastroesophageal reflux disease	0.3	0.2	0.03	0.3	0.4	-0.01
Gastrointestinal hemorrhage	0.5	0.4	0.02	0.5	0.6	-0.01
Hypertensive disorder	0.9	0.8	0.01	0.9	1	-0.01
Lesion of liver	0.1	0.1	0.01	0.1	0.2	-0.01
Osteoarthritis	0.8	0.7	0.01	0.8	0.8	0

Table S6.117. *Continued.* Selected baseline characteristics for UK IMRD, for the short-term risk of dementia

Characteristic	Before propensity score matching			After propensity score matching		
	Targets, %	Comparators, %	Standardized difference	Targets, %	Comparators, %	Standardized difference
**Medical history (general)**						
Pneumonia	1.1	0.2	0.1	0.9	1	-0.01
Psoriasis	0.4	0.3	0.02	0.4	0.4	-0.01
Renal impairment	1.1	0.5	0.07	1	1.1	-0.01
Rheumatoid arthritis	0.1	0.1	0.01	0.1	0.1	0
Urinary tract infectious disease	2.1	1	0.1	2	2.2	-0.02
**Medical history (cardiovascular disease)**						
Atrial fibrillation	0.6	0.3	0.04	0.6	0.7	-0.01
Cerebrovascular disease	0.4	0.3	0.03	0.4	0.5	-0.01
Coronary arteriosclerosis	0.1	0.1	0	0.1	0.1	0
Heart disease	1.7	1	0.06	1.6	1.9	-0.02
Heart failure	0.4	0.2	0.04	0.4	0.4	-0.01
Ischemic heart disease	0.4	0.3	0.03	0.4	0.5	-0.01
Pulmonary embolism	0.2	0.1	0.03	0.2	0.2	0
Venous thrombosis	0.3	0.2	0.02	0.3	0.4	-0.02
**Medical history (neoplasms)**						
Malignant neoplastic disease	1	0.8	0.02	0.9	1.1	-0.02
Malignant tumor of breast	0.1	0.1	0.01	0.1	0.1	0
**Medication use**						
Agents acting on the renin-angiotensin system	12.6	12.2	0.01	12.4	12.5	0
Antibacterials for systemic use	38.4	27.6	0.23	37.8	40.2	-0.05
Antidepressants	22.2	15.9	0.16	21.8	23.7	-0.05
Antiepileptics	5.9	4.3	0.07	5.8	6.4	-0.03

Table S6.117. *Continued.* Selected baseline characteristics for UK IMRD, for the short-term risk of dementia

Characteristic	Before propensity score matching			After propensity score matching		
	Targets, %	Comparators, %	Standardized difference	Targets, %	Comparators, %	Standardized difference
**Medication use**						
Antiinflammatory and antirheumatic products	20.5	16.3	0.11	20.2	21.3	-0.03
Antineoplastic agents	1	1	0	1	1	0
Antipsoriatics	4.5	3	0.08	4.4	4.8	-0.02
Antithrombotic agents	10.1	9	0.04	9.5	9.9	-0.01
Beta blocking agents	10	8.7	0.04	9.7	10.2	-0.02
Calcium channel blockers	8.3	8.4	0	8	8.1	0
Diuretics	6.9	6	0.04	6.5	6.7	0
Drugs for acid-related disorders	25.8	20.6	0.12	25.2	27.2	-0.05
Drugs for obstructive airway diseases	23.7	19.1	0.11	23.6	24.9	-0.03
Drugs used in diabetes	6.5	4.9	0.07	6.3	6.6	-0.01
Immunosuppressants	1	0.9	0.01	1	1	0
Lipid modifying agents	13.9	14.1	0	13.4	13.6	-0.01
Opioids	17.6	12.9	0.13	17.3	18.6	-0.04
Psycholeptics	10.3	7.7	0.09	9.7	10.9	-0.04
Psychostimulants, agents used for ADHD and nootropics	0.2	0.3	-0.01	0.2	0.3	-0.01

Table S6.118. Selected baseline characteristics for UK IMRD, for the short-term risk of neurodevelopmental disorders

Characteristic	Before propensity score matching			After propensity score matching		
	**Targets, %**	**Comparators, %**	**Standardized difference**	**Targets, %**	**Comparators, %**	**Standardized difference**
**Age group (years)**						
5-9	2.3	5.3	-0.16	2.3	2.2	0.01
10-14	3.8	5.3	-0.07	3.8	3.7	0
20-24	7.3	5.2	0.09	7.4	7.7	-0.01
25-29	7.6	6.1	0.06	7.6	7.8	-0.01
30-34	7.7	6.9	0.03	7.8	7.7	0
35-39	8.1	7	0.04	8.2	8	0
40-44	7.6	6.4	0.05	7.7	7.6	0
45-49	8.1	6.7	0.05	8.2	8.3	0
50-54	8.9	7.4	0.05	8.9	9	0
55-59	8.4	7.3	0.04	8.4	8.5	0
60-64	6	6.6	-0.02	6	6	0
65-69	3.6	5.7	-0.1	3.6	3.5	0
70-74	3.2	5.7	-0.12	3.2	3	0.01
75-79	2.8	4.1	-0.07	2.8	2.6	0.01
80-84	2.5	2.8	-0.02	2.5	2.5	0
85-89	2.1	1.7	0.03	2.1	2.2	-0.01
90-94	1.4	0.8	0.06	1.4	1.5	-0.01
95-99	0.6	0.2	0.06	0.6	0.6	0
**Sex**						
Female	56.7	49.3	0.15	56.7	58.5	-0.04

Table S6.118. *Continued.* Selected baseline characteristics for UK IMRD, for the short-term risk of neurodevelopmental disorders

Characteristic	Before propensity score matching			After propensity score matching		
	Targets, %	Comparators, %	Standardized difference	Targets, %	Comparators, %	Standardized difference
**Race**						
race = Asian	0.8	0.6	0.02	0.8	0.7	0.01
race = White	4.5	6.7	-0.09	4.5	4.2	0.01
race = Bangladeshi	0.2	0.1	0.01	0.2	0.1	0.01
race = Chinese	0.1	0.4	-0.06	0.1	0.1	0
race = Pakistani	1.7	0.6	0.11	1.7	1.8	0
race = African	0.7	0.8	-0.02	0.7	0.8	-0.01
race = West Indian	0.1	0.1	0	0.1	0.1	0
race = European	36.7	36.4	0	36.7	37.4	-0.01
**Medical history (general)**						
Acute respiratory disease	4.4	2.6	0.1	4.4	4.8	-0.02
Chronic obstructive lung disease	0.7	0.6	0.02	0.7	0.9	-0.02
Dementia	0.4	0.2	0.04	0.4	0.5	-0.01
Depressive disorder	1.4	1	0.04	1.4	1.6	-0.01
Diabetes mellitus	0.7	0.6	0.01	0.7	0.8	-0.01
Gastroesophageal reflux disease	0.3	0.2	0.02	0.3	0.4	0
Gastrointestinal hemorrhage	0.5	0.4	0.02	0.5	0.6	-0.01
Hyperlipidemia	0.2	0.2	0	0.2	0.2	0
Hypertensive disorder	0.9	0.8	0.01	0.9	1	-0.01
Lesion of liver	0.1	0.1	0.01	0.1	0.2	-0.01
Obesity	0.2	0.1	0.02	0.2	0.2	0
Osteoarthritis	0.8	0.7	0.01	0.8	0.9	0
Pneumonia	1.1	0.2	0.11	1	1.1	-0.01
Psoriasis	0.4	0.3	0.02	0.4	0.4	-0.01
Renal impairment	1.1	0.5	0.07	1.1	1.2	-0.01

Table S6.118. *Continued.* Selected baseline characteristics for UK IMRD, for the short-term risk of neurodevelopmental disorders

Characteristic	Before propensity score matching			After propensity score matching		
	Targets, %	Comparators, %	Standardized difference	Targets, %	Comparators, %	Standardized difference
**Medical history (general)**						
Rheumatoid arthritis	0.1	0.1	0.01	0.1	0.1	0
Urinary tract infectious disease	2.1	1	0.09	2.1	2.4	-0.02
**Medical history (cardiovascular disease)**						
Atrial fibrillation	0.6	0.3	0.04	0.6	0.7	-0.01
Cerebrovascular disease	0.4	0.3	0.03	0.4	0.5	-0.02
Coronary arteriosclerosis	0.1	0.1	0	0.1	0.1	0
Heart disease	1.7	1.1	0.06	1.7	2	-0.02
Heart failure	0.4	0.2	0.05	0.4	0.4	-0.01
Ischemic heart disease	0.4	0.3	0.02	0.4	0.5	-0.01
Peripheral vascular disease	0.1	0.1	0.01	0.1	0.1	0
Pulmonary embolism	0.2	0.1	0.03	0.2	0.2	0
Venous thrombosis	0.3	0.2	0.02	0.3	0.4	-0.02
**Medical history (neoplasms)**						
Malignant neoplastic disease	1	0.8	0.02	1	1.2	-0.02
Malignant tumor of lung	0.1	0.1	0.01	0.1	0.1	-0.02
**Medication use**						
Agents acting on the renin-angiotensin system	12.6	12.3	0.01	12.6	12.8	-0.01
Antibacterials for systemic use	38.4	27.7	0.23	38.3	40.8	-0.05
Antidepressants	22.2	15.9	0.16	22.1	24	-0.04
Antiepileptics	5.9	4.3	0.07	5.9	6.6	-0.03
Antiinflammatory and antirheumatic products	20.5	16.5	0.1	20.5	21.6	-0.03
Antipsoriatics	4.5	3	0.08	4.5	4.9	-0.02

Table S6.118. *Continued.* Selected baseline characteristics for UK IMRD, for the short-term risk of neurodevelopmental disorders

Characteristic	Before propensity score matching			After propensity score matching		
	**Targets, %**	**Comparators, %**	**Standardized difference**	**Targets, %**	**Comparators, %**	**Standardized difference**
**Medication use**						
Antithrombotic agents	10.1	9.2	0.03	10.1	10.5	-0.01
Beta blocking agents	10	8.8	0.04	9.9	10.4	-0.02
Calcium channel blockers	8.3	8.4	0	8.2	8.3	0
Diuretics	6.9	6	0.04	6.8	7	-0.01
Drugs for acid-related disorders	25.8	20.7	0.12	25.7	27.8	-0.05
Drugs for obstructive airway diseases	23.7	19.1	0.11	23.7	24.9	-0.03
Drugs used in diabetes	6.5	5	0.06	6.4	6.7	-0.01
Immunosuppressants	1	0.9	0.01	1	1	0
Lipid modifying agents	13.9	14.3	-0.01	13.9	14.1	-0.01
Opioids	17.6	12.9	0.13	17.6	18.9	-0.04
Psycholeptics	10.3	7.7	0.09	10.2	11.3	-0.04
Psychostimulants, agents used for ADHD and nootropics	0.2	0.3	-0.01	0.2	0.2	-0.01

Table S6.119. Selected baseline characteristics for UK IMRD, for the short-term risk of any of psychiatric and neuropsychiatric disorders

Characteristic	Before propensity score matching			After propensity score matching		
	Targets, %	Comparators, %	Standardized difference	Targets, %	Comparators, %	Standardized difference
**Age group (years)**						
0-4	1.7	3.9	-0.14	1.7	1.7	0
5-9	2.3	5.2	-0.15	2.4	2.3	0.01
10-14	3.8	5.3	-0.07	3.9	3.8	0
15-19	6	5	0.04	6.1	6	0
20-24	7.3	5.2	0.09	7.4	7.7	-0.01
25-29	7.6	6.3	0.05	7.6	7.9	-0.01
30-34	7.7	7	0.03	7.8	7.7	0
35-39	8.1	7	0.04	8.2	8.1	0
40-44	7.6	6.5	0.05	7.7	7.6	0
45-49	8.1	6.7	0.05	8.3	8.3	0
50-54	8.9	7.3	0.06	9.1	9.1	0
55-59	8.4	7.4	0.04	8.6	8.7	0
60-64	6	6.5	-0.02	6.1	6.1	0
65-69	3.6	5.6	-0.1	3.6	3.6	0
70-74	3.2	5.6	-0.12	3.2	2.9	0.01
75-79	2.8	4.1	-0.07	2.6	2.5	0.01
80-84	2.5	2.9	-0.02	2.2	2.2	0
85-89	2.1	1.7	0.03	1.8	1.8	0
90-94	1.4	0.7	0.07	1.2	1.2	-0.01
95-99	0.6	0.2	0.06	0.5	0.5	0
**Sex**						
Female	56.7	49.3	0.15	56.3	58.2	-0.04

Table S6.119. *Continued.* Selected baseline characteristics for UK IMRD, for the short-term risk of any of psychiatric and neuropsychiatric disorders

Characteristic	Before propensity score matching			After propensity score matching		
	Targets, %	Comparators, %	Standardized difference	Targets, %	Comparators, %	Standardized difference
**Race**						
race = Asian	0.8	0.7	0.01	0.8	0.7	0.01
race = White	4.5	6.7	-0.1	4.5	4.2	0.01
race = Asian Indian	0.9	0.9	0.01	1	0.9	0
race = Bangladeshi	0.2	0.1	0.02	0.2	0.1	0.01
race = Chinese	0.1	0.4	-0.06	0.1	0.1	0
race = Pakistani	1.7	0.6	0.11	1.8	1.8	-0.01
race = African	0.7	0.9	-0.03	0.7	0.8	-0.01
race = West Indian	0.1	0.1	-0.01	0.1	0.1	-0.01
race = European	36.7	36.1	0.01	36.6	37.2	-0.01
race = Arab	0.1	0.1	0	0.1	0.1	0
**Medical history (general)**						
Acute respiratory disease	4.4	2.7	0.09	4.2	4.6	-0.02
Chronic obstructive lung disease	0.7	0.6	0.02	0.7	0.8	-0.02
Diabetes mellitus	0.7	0.6	0.01	0.7	0.7	-0.01
Gastroesophageal reflux disease	0.3	0.2	0.02	0.3	0.4	-0.01
Gastrointestinal hemorrhage	0.5	0.4	0.02	0.5	0.6	-0.01
Hyperlipidemia	0.2	0.2	0	0.2	0.2	0
Hypertensive disorder	0.9	0.8	0.01	0.9	0.9	-0.01
Lesion of liver	0.1	0.1	0.01	0.1	0.1	-0.01
Obesity	0.2	0.1	0.02	0.2	0.2	0
Osteoarthritis	0.8	0.7	0.01	0.8	0.8	0
Pneumonia	1.1	0.2	0.11	0.9	1	-0.01

Table S6.119. *Continued.* Selected baseline characteristics for UK IMRD, for the short-term risk of any of psychiatric and neuropsychiatric disorders

Characteristic	Before propensity score matching			After propensity score matching		
	Targets, %	Comparators, %	Standardized difference	Targets, %	Comparators, %	Standardized difference
**Medical history (general)**						
Psoriasis	0.4	0.3	0.01	0.3	0.4	-0.01
Renal impairment	1.1	0.5	0.07	1	1.1	-0.01
Rheumatoid arthritis	0.1	0.1	0.01	0.1	0.1	0
Urinary tract infectious disease	2.1	1	0.09	1.9	2.1	-0.01
**Medical history (cardiovascular disease)**						
Atrial fibrillation	0.6	0.3	0.04	0.6	0.7	-0.02
Cerebrovascular disease	0.4	0.3	0.03	0.4	0.4	-0.01
Heart disease	1.7	1.1	0.06	1.6	1.9	-0.03
Heart failure	0.4	0.2	0.04	0.4	0.4	-0.01
Ischemic heart disease	0.4	0.3	0.03	0.4	0.5	-0.02
Peripheral vascular disease	0.1	0.1	0.01	0.1	0.1	0
Pulmonary embolism	0.2	0.1	0.03	0.2	0.2	0
Venous thrombosis	0.3	0.2	0.02	0.2	0.4	-0.03
**Medical history (neoplasms)**						
Malignant neoplastic disease	1	0.8	0.02	0.9	1.2	-0.02
Malignant tumor of breast	0.1	0.1	0.01	0.1	0.1	-0.01
**Medication use**						
Agents acting on the renin-angiotensin system	12.6	12.2	0.01	12.4	12.6	0
Antibacterials for systemic use	38.4	27.6	0.23	37.3	39.9	-0.05
Antidepressants	22.2	16	0.16	19.9	21.7	-0.04
Antiepileptics	5.9	4.3	0.07	5.6	6.2	-0.03
Antiinflammatory and antirheumatic products	20.5	16.3	0.11	19.9	21.1	-0.03

Table S6.119. *Continued.* Selected baseline characteristics for UK IMRD, for the short-term risk of any of psychiatric and neuropsychiatric disorders

Characteristic	Before propensity score matching			After propensity score matching		
	**Targets, %**	**Comparators, %**	**Standardized difference**	**Targets, %**	**Comparators, %**	**Standardized difference**
**Medication use**						
Antineoplastic agents	1	1	0	1	1	0
Antipsoriatics	4.5	3	0.08	4.3	4.7	-0.02
Antithrombotic agents	10.1	9.1	0.03	9.4	9.9	-0.02
Beta blocking agents	10	8.8	0.04	9.2	9.7	-0.02
Calcium channel blockers	8.3	8.3	0	8	8.1	0
Diuretics	6.9	6.1	0.03	6.5	6.7	-0.01
Drugs for acid-related disorders	25.8	20.6	0.12	24.8	26.9	-0.05
Drugs for obstructive airway diseases	23.7	19	0.11	23.3	24.6	-0.03
Drugs used in diabetes	6.5	4.9	0.07	6.3	6.6	-0.01
Immunosuppressants	1	0.9	0.01	1	1	0
Lipid modifying agents	13.9	14.2	-0.01	13.4	13.7	-0.01
Opioids	17.6	12.8	0.13	16.9	18.4	-0.04
Psycholeptics	10.3	7.7	0.09	8.8	9.9	-0.04
Psychostimulants, agents used for ADHD and nootropics	0.2	0.3	-0.01	0.2	0.2	-0.01

Table S6.120. Selected baseline characteristics for UK IMRD, for the medium-term risk of depression

Characteristic	Before propensity score matching			After propensity score matching		
	**Targets, %**	**Comparators, %**	**Standardized difference**	**Targets, %**	**Comparators, %**	**Standardized difference**
**Age group (years)**						
0-4	1.7	3.9	-0.14	1.7	1.8	0
5-9	2.3	5.2	-0.15	2.5	2.4	0
10-14	3.8	5.2	-0.07	4	3.8	0.01
15-19	6	4.9	0.05	6.2	6.4	0
20-24	7.3	5.1	0.09	7.5	8	-0.02
25-29	7.6	6.3	0.05	7.7	7.8	0
35-39	8.1	6.9	0.05	8.4	8.3	0
40-44	7.6	6.5	0.05	7.9	8	0
45-49	8.1	6.8	0.05	8.6	8.4	0.01
50-54	8.9	7.4	0.05	9.3	9.5	-0.01
60-64	6	6.5	-0.02	6.3	6.3	0
65-69	3.6	5.6	-0.1	3.6	3.5	0
70-74	3.2	5.6	-0.12	2.9	2.6	0.02
75-79	2.8	4.1	-0.07	2.3	2.1	0.01
80-84	2.5	2.9	-0.02	1.8	1.7	0
85-89	2.1	1.8	0.02	1.3	1.3	0
90-94	1.4	0.7	0.07	0.8	0.9	-0.01
95-99	0.6	0.2	0.06	0.3	0.3	0
**Sex**						
Female	56.7	49.6	0.14	56.9	58.7	-0.04

Table S6.120. *Continued.* Selected baseline characteristics for UK IMRD, for the medium-term risk of depression

Characteristic	Before propensity score matching			After propensity score matching		
	Targets, %	Comparators, %	Standardized difference	Targets, %	Comparators, %	Standardized difference
**Race**						
race = Asian	0.8	0.6	0.02	0.8	0.8	0
race = Black or African American	0.2	0.3	-0.02	0.2	0.2	-0.01
race = White	4.5	6.7	-0.1	4.5	4.2	0.02
race = Asian Indian	0.9	0.9	0.01	0.8	0.8	0
race = Bangladeshi	0.2	0.1	0.01	0.2	0.1	0.01
race = Chinese	0.1	0.4	-0.06	0.1	0.1	0
race = Pakistani	1.7	0.6	0.1	1.9	1.9	0
race = African	0.7	0.9	-0.02	0.6	0.6	0
race = European	36.7	36.3	0.01	36.3	37.3	-0.02
**Medical history (general)**						
Acute respiratory disease	4.4	2.6	0.1	4.3	4.7	-0.02
Attention deficit hyperactivity disorder	0.1	0.1	0	0.1	0.1	0
Chronic obstructive lung disease	0.7	0.6	0.02	0.6	0.6	0
Dementia	0.4	0.2	0.04	0.2	0.3	-0.01
Diabetes mellitus	0.7	0.6	0.01	0.7	0.7	-0.01
Gastroesophageal reflux disease	0.3	0.2	0.02	0.3	0.3	0
Gastrointestinal hemorrhage	0.5	0.4	0.02	0.5	0.5	-0.01
Hypertensive disorder	0.9	0.8	0.01	0.9	0.9	0
Lesion of liver	0.1	0.1	0.01	0.1	0.1	-0.01
Obesity	0.2	0.1	0.02	0.2	0.2	0
Osteoarthritis	0.8	0.7	0.01	0.7	0.8	0
Pneumonia	1.1	0.2	0.11	0.7	0.8	-0.01
Psoriasis	0.4	0.3	0.02	0.3	0.4	0
Renal impairment	1.1	0.5	0.06	0.8	0.8	0
Rheumatoid arthritis	0.1	0.1	0	0.1	0.1	0
Urinary tract infectious disease	2.1	1	0.09	1.8	1.9	-0.01

Table S6.120. *Continued.* Selected baseline characteristics for UK IMRD, for the medium-term risk of depression

Characteristic	Before propensity score matching			After propensity score matching		
	**Targets, %**	**Comparators, %**	**Standardized difference**	**Targets, %**	**Comparators, %**	**Standardized difference**
**Medical history (cardiovascular disease)**						
Atrial fibrillation	0.6	0.4	0.04	0.4	0.5	-0.01
Cerebrovascular disease	0.4	0.3	0.03	0.3	0.4	-0.01
Coronary arteriosclerosis	0.1	0.1	0	0.1	0.1	0.01
Heart disease	1.7	1	0.06	1.3	1.5	-0.01
Heart failure	0.4	0.2	0.04	0.3	0.3	-0.01
Ischemic heart disease	0.4	0.3	0.03	0.4	0.4	-0.01
Peripheral vascular disease	0.1	0.1	0.01	0.1	0.1	0.01
Pulmonary embolism	0.2	0.1	0.03	0.2	0.1	0
Venous thrombosis	0.3	0.2	0.02	0.2	0.3	-0.02
**Medical history (neoplasms)**						
Malignant neoplastic disease	1	0.8	0.02	0.7	0.7	-0.01
Malignant tumor of breast	0.1	0.1	0	0.1	0.1	0
**Medication use**						
Agents acting on the renin-angiotensin system	12.6	12.1	0.01	11.9	12	-0.01
Antibacterials for systemic use	38.4	27.8	0.23	37.1	39.3	-0.04
Antidepressants	22.2	15.9	0.16	20.6	22.1	-0.04
Antiepileptics	5.9	4.3	0.07	5.3	5.9	-0.02
Antiinflammatory and antirheumatic products	20.5	16.4	0.11	20	20.9	-0.02
Antineoplastic agents	1	1	0	0.9	1	0
Antipsoriatics	4.5	3	0.08	4.2	4.5	-0.01
Antithrombotic agents	10.1	9.1	0.03	8.3	8.5	0
Beta blocking agents	10	8.8	0.04	8.9	9.2	-0.01
Calcium channel blockers	8.3	8.2	0	7.7	7.6	0

Table S6.120. *Continued.* Selected baseline characteristics for UK IMRD, for the medium-term risk of depression

Characteristic	Before propensity score matching			After propensity score matching		
	**Targets, %**	**Comparators, %**	**Standardized difference**	**Targets, %**	**Comparators, %**	**Standardized difference**
**Medication use**						
Diuretics	6.9	6.1	0.03	5.6	5.7	0
Drugs for acid-related disorders	25.8	20.6	0.12	24.4	25.9	-0.04
Drugs for obstructive airway diseases	23.7	19.2	0.11	23.4	24.5	-0.03
Drugs used in diabetes	6.5	4.9	0.07	5.8	6	-0.01
Immunosuppressants	1	0.9	0.01	0.9	1	-0.01
Lipid modifying agents	13.9	14.2	-0.01	12.7	12.8	0
Opioids	17.6	12.9	0.13	16.8	17.7	-0.02
Psycholeptics	10.3	7.8	0.09	9	9.7	-0.02
Psychostimulants, agents used for ADHD and nootropics	0.2	0.3	-0.01	0.2	0.3	-0.01

Table S6.121. Selected baseline characteristics for UK IMRD, for the medium-term risk of anxiety disorders

Characteristic	Before propensity score matching			After propensity score matching		
	Targets, %	Comparators, %	Standardized difference	Targets, %	Comparators, %	Standardized difference
**Age group (years)**						
0-4	1.7	3.9	-0.14	1.8	1.8	0
5-9	2.3	5.3	-0.15	2.5	2.4	0
10-14	3.8	5.3	-0.07	4	3.8	0.01
15-19	6	4.8	0.05	6.2	6.3	0
20-24	7.3	5.2	0.09	7.5	8	-0.02
25-29	7.6	6.2	0.06	7.7	7.8	0
40-44	7.6	6.5	0.04	7.9	8	0
45-49	8.1	6.7	0.05	8.6	8.4	0.01
50-54	8.9	7.4	0.05	9.3	9.6	-0.01
55-59	8.4	7.4	0.04	8.9	8.8	0
60-64	6	6.5	-0.02	6.4	6.3	0
65-69	3.6	5.6	-0.1	3.6	3.5	0
70-74	3.2	5.6	-0.12	2.9	2.6	0.02
75-79	2.8	4.1	-0.07	2.3	2.1	0.01
80-84	2.5	2.9	-0.02	1.8	1.7	0
85-89	2.1	1.8	0.03	1.3	1.4	0
90-94	1.4	0.8	0.06	0.8	0.9	-0.01
95-99	0.6	0.2	0.06	0.3	0.3	0
**Sex**						
Female	56.7	49.4	0.15	56.7	58.6	-0.04

Table S6.121. *Continued.* Selected baseline characteristics for UK IMRD, for the medium-term risk of anxiety disorders

Characteristic	Before propensity score matching			After propensity score matching		
	Targets, %	Comparators, %	Standardized difference	Targets, %	Comparators, %	Standardized difference
**Race**						
race = Black or African American	0.2	0.3	-0.02	0.2	0.2	-0.01
race = White	4.5	6.7	-0.1	4.5	4.2	0.01
race = Asian Indian	0.9	0.8	0.01	0.8	0.8	0
race = Bangladeshi	0.2	0.1	0.01	0.2	0.1	0.01
race = Chinese	0.1	0.4	-0.06	0.1	0.1	0
race = Pakistani	1.7	0.6	0.11	1.9	1.9	0
race = African	0.7	0.9	-0.02	0.6	0.6	0
race = European	36.7	36.3	0.01	36.3	37.3	-0.02
**Medical history (general)**						
Acute respiratory disease	4.4	2.7	0.09	4.3	4.7	-0.02
Attention deficit hyperactivity disorder	0.1	0.1	0	0.1	0.1	0
Chronic obstructive lung disease	0.7	0.6	0.02	0.6	0.6	0
Dementia	0.4	0.2	0.04	0.2	0.3	-0.01
Depressive disorder	1.4	1	0.04	0.8	0.8	-0.01
Diabetes mellitus	0.7	0.6	0.01	0.7	0.7	-0.01
Gastroesophageal reflux disease	0.3	0.2	0.03	0.3	0.3	0
Gastrointestinal hemorrhage	0.5	0.4	0.02	0.5	0.5	0
Hyperlipidemia	0.2	0.2	0	0.2	0.2	-0.01
Hypertensive disorder	0.9	0.8	0.01	0.9	0.9	0
Lesion of liver	0.1	0.1	0.01	0.1	0.1	-0.01
Obesity	0.2	0.1	0.02	0.2	0.2	0
Osteoarthritis	0.8	0.7	0.01	0.8	0.8	0
Pneumonia	1.1	0.2	0.11	0.7	0.8	-0.01
Psoriasis	0.4	0.2	0.02	0.3	0.4	0

Table S6.121. *Continued.* Selected baseline characteristics for UK IMRD, for the medium-term risk of anxiety disorders

Characteristic	Before propensity score matching			After propensity score matching		
	Targets, %	Comparators, %	Standardized difference	Targets, %	Comparators, %	Standardized difference
**Medical history (general)**						
Renal impairment	1.1	0.5	0.07	0.8	0.8	0
Rheumatoid arthritis	0.1	0.1	0.01	0.1	0.1	0
Urinary tract infectious disease	2.1	1	0.09	1.8	1.9	-0.01
**Medical history (cardiovascular disease)**						
Atrial fibrillation	0.6	0.3	0.04	0.4	0.5	-0.01
Cerebrovascular disease	0.4	0.3	0.03	0.3	0.4	-0.01
Heart disease	1.7	1.1	0.06	1.3	1.5	-0.02
Heart failure	0.4	0.2	0.04	0.2	0.3	-0.01
Ischemic heart disease	0.4	0.3	0.02	0.4	0.4	-0.01
Peripheral vascular disease	0.1	0.1	0.01	0.1	0.1	0.01
Pulmonary embolism	0.2	0.1	0.03	0.2	0.1	0
Venous thrombosis	0.3	0.2	0.01	0.3	0.3	-0.01
**Medical history (neoplasms)**						
Malignant neoplastic disease	1	0.8	0.02	0.7	0.7	-0.01
Malignant tumor of breast	0.1	0.1	0	0.1	0.1	0
**Medication use**						
Agents acting on the renin-angiotensin system	12.6	12.1	0.02	11.9	12.1	-0.01
Antibacterials for systemic use	38.4	27.6	0.23	36.9	39.3	-0.05
Antidepressants	22.2	15.8	0.16	20.6	22.2	-0.04
Antiepileptics	5.9	4.3	0.07	5.3	5.9	-0.03
Antiinflammatory and antirheumatic products	20.5	16.3	0.11	19.9	20.9	-0.02
Antineoplastic agents	1	1	0	0.9	1	0

Table S6.121. *Continued.* Selected baseline characteristics for UK IMRD, for the medium-term risk of anxiety disorders

Characteristic	Before propensity score matching			After propensity score matching		
	**Targets, %**	**Comparators, %**	**Standardized difference**	**Targets, %**	**Comparators, %**	**Standardized difference**
**Medication use**						
Antipsoriatics	4.5	3	0.08	4.2	4.5	-0.01
Antithrombotic agents	10.1	9.1	0.04	8.3	8.5	-0.01
Beta blocking agents	10	8.7	0.04	8.7	9.1	-0.01
Calcium channel blockers	8.3	8.2	0	7.7	7.6	0
Diuretics	6.9	6.1	0.03	5.6	5.8	-0.01
Drugs for acid-related disorders	25.8	20.7	0.12	24.2	25.8	-0.04
Drugs for obstructive airway diseases	23.7	19.1	0.11	23.2	24.5	-0.03
Drugs used in diabetes	6.5	4.9	0.07	5.8	6	-0.01
Immunosuppressants	1	0.9	0.01	0.9	1	-0.01
Lipid modifying agents	13.9	14.2	-0.01	12.7	12.9	0
Opioids	17.6	12.8	0.13	16.7	17.7	-0.03
Psycholeptics	10.3	7.6	0.09	8.8	9.5	-0.02
Psychostimulants, agents used for ADHD and nootropics	0.2	0.3	-0.01	0.2	0.3	-0.01

Table S6.122. Selected baseline characteristics for UK IMRD, for the medium-term risk of alcohol misuse or dependence

Characteristic	Before propensity score matching			After propensity score matching		
	Targets, %	Comparators, %	Standardized difference	Targets, %	Comparators, %	Standardized difference
**Age group (years)**						
0-4	1.7	3.9	-0.14	1.7	1.8	0
5-9	2.3	5.2	-0.15	2.4	2.4	0
10-14	3.8	5.2	-0.07	4	3.7	0.01
15-19	6	4.9	0.04	6.3	6.4	0
20-24	7.3	5.2	0.09	7.7	8.1	-0.02
25-29	7.6	6.2	0.06	7.8	7.9	0
30-34	7.7	7	0.03	8	8	0
35-39	8.1	6.9	0.05	8.4	8.4	0
40-44	7.6	6.5	0.04	8	8	0
45-49	8.1	6.8	0.05	8.6	8.4	0.01
50-54	8.9	7.4	0.06	9.3	9.5	-0.01
55-59	8.4	7.4	0.04	8.8	8.8	0
60-64	6	6.4	-0.02	6.3	6.2	0
65-69	3.6	5.6	-0.1	3.6	3.5	0
70-74	3.2	5.7	-0.12	2.9	2.6	0.02
75-79	2.8	4	-0.06	2.3	2.1	0.01
80-84	2.5	2.9	-0.02	1.7	1.7	0
85-89	2.1	1.8	0.02	1.3	1.3	0
90-94	1.4	0.7	0.07	0.8	0.8	-0.01
95-99	0.6	0.2	0.06	0.3	0.3	0
**Sex**						
Female	56.7	49.5	0.14	57.1	59.1	-0.04

Table S6.122. *Continued.* Selected baseline characteristics for UK IMRD, for the medium-term risk of alcohol misuse or dependence

Characteristic	Before propensity score matching			After propensity score matching		
	Targets, %	Comparators, %	Standardized difference	Targets, %	Comparators, %	Standardized difference
**Race**						
race = Black or African American	0.2	0.3	-0.02	0.2	0.2	-0.01
race = White	4.5	6.7	-0.1	4.5	4.2	0.01
race = Asian Indian	0.9	0.9	0.01	0.8	0.8	0
race = Bangladeshi	0.2	0.1	0.02	0.2	0.1	0.01
race = Chinese	0.1	0.3	-0.06	0.1	0.1	0
race = Pakistani	1.7	0.6	0.1	1.9	1.9	0
race = African	0.7	0.9	-0.02	0.6	0.6	0
race = European	36.7	36.1	0.01	36.4	37.3	-0.02
race = Middle Eastern or North African	0.1	0.1	0	0.1	0.1	0
**Medical history (general)**						
Acute respiratory disease	4.4	2.7	0.09	4.3	4.8	-0.02
Chronic obstructive lung disease	0.7	0.6	0.02	0.6	0.6	0
Dementia	0.4	0.2	0.04	0.2	0.3	-0.01
Depressive disorder	1.4	1.1	0.04	1.4	1.6	-0.01
Diabetes mellitus	0.7	0.6	0.02	0.7	0.7	-0.01
Gastroesophageal reflux disease	0.3	0.2	0.02	0.3	0.4	0
Gastrointestinal hemorrhage	0.5	0.4	0.02	0.5	0.5	-0.01
Hyperlipidemia	0.2	0.2	0	0.2	0.2	-0.01
Hypertensive disorder	0.9	0.8	0.01	0.9	0.9	0
Lesion of liver	0.1	0.1	0.01	0.1	0.1	-0.01
Obesity	0.2	0.1	0.02	0.2	0.2	0
Osteoarthritis	0.8	0.7	0.01	0.8	0.8	0

Table S6.122. *Continued.* Selected baseline characteristics for UK IMRD, for the medium-term risk of alcohol misuse or dependence

Characteristic	Before propensity score matching			After propensity score matching		
	Targets, %	Comparators, %	Standardized difference	Targets, %	Comparators, %	Standardized difference
**Medical history (general)**						
Pneumonia	1.1	0.2	0.11	0.8	0.8	-0.01
Psoriasis	0.4	0.3	0.01	0.3	0.4	0
Renal impairment	1.1	0.5	0.07	0.8	0.8	0
Rheumatoid arthritis	0.1	0.1	0.01	0.1	0.1	-0.01
Urinary tract infectious disease	2.1	1	0.09	1.8	2	-0.01
**Medical history (cardiovascular disease)**						
Atrial fibrillation	0.6	0.4	0.04	0.4	0.5	-0.01
Cerebrovascular disease	0.4	0.3	0.03	0.3	0.4	-0.01
Coronary arteriosclerosis	0.1	0.1	0.01	0.1	0.1	0.01
Heart disease	1.7	1.1	0.06	1.3	1.5	-0.01
Heart failure	0.4	0.2	0.04	0.3	0.3	-0.01
Ischemic heart disease	0.4	0.3	0.02	0.4	0.4	-0.01
Peripheral vascular disease	0.1	0.1	0.01	0.1	0.1	0.01
Pulmonary embolism	0.2	0.1	0.04	0.2	0.1	0
Venous thrombosis	0.3	0.2	0.02	0.3	0.3	-0.01
**Medical history (neoplasms)**						
Malignant neoplastic disease	1	0.8	0.02	0.7	0.7	-0.01
Malignant tumor of breast	0.1	0.1	0	0.1	0.1	0
**Medication use**						
Agents acting on the renin-angiotensin system	12.6	12.2	0.01	11.8	12	-0.01
Antibacterials for systemic use	38.4	27.6	0.23	37.3	39.6	-0.05
Antidepressants	22.2	15.9	0.16	21.6	23.3	-0.04

Table S6.122. *Continued.* Selected baseline characteristics for UK IMRD, for the medium-term risk of alcohol misuse or dependence

Characteristic	Before propensity score matching			After propensity score matching		
	Targets, %	Comparators, %	Standardized difference	Targets, %	Comparators, %	Standardized difference
**Medication use**						
Antiepileptics	5.9	4.3	0.07	5.4	5.9	-0.02
Antiinflammatory and antirheumatic products	20.5	16.3	0.11	20	21	-0.02
Antineoplastic agents	1	1	0	0.9	1	0
Antipsoriatics	4.5	3	0.08	4.2	4.5	-0.02
Antithrombotic agents	10.1	9.1	0.04	8.3	8.4	0
Beta blocking agents	10	8.8	0.04	9	9.4	-0.01
Calcium channel blockers	8.3	8.3	0	7.7	7.6	0
Diuretics	6.9	6.1	0.03	5.6	5.7	0
Drugs for acid-related disorders	25.8	20.7	0.12	24.5	26	-0.04
Drugs for obstructive airway diseases	23.7	18.9	0.12	23.5	24.7	-0.03
Drugs used in diabetes	6.5	4.9	0.07	5.8	6	-0.01
Immunosuppressants	1	0.9	0.01	0.9	1	-0.01
Lipid modifying agents	13.9	14.2	-0.01	12.7	12.8	0
Opioids	17.6	12.8	0.13	16.9	17.9	-0.03
Psycholeptics	10.3	7.7	0.09	9.2	9.9	-0.02
Psychostimulants, agents used for ADHD and nootropics	0.2	0.3	-0.01	0.2	0.3	-0.01

Table S6.123. Selected baseline characteristics for UK IMRD, for the medium-term risk of substance misuse or dependence

Characteristic	Before propensity score matching			After propensity score matching		
	Targets, %	Comparators, %	Standardized difference	Targets, %	Comparators, %	Standardized difference
**Age group (years)**						
0-4	1.7	3.9	-0.14	1.7	1.8	0
5-9	2.3	5.2	-0.15	2.4	2.4	0
10-14	3.8	5.2	-0.07	4	3.8	0.01
15-19	6	4.9	0.04	6.3	6.4	0
20-24	7.3	5.2	0.09	7.6	8.1	-0.02
25-29	7.6	6.2	0.06	7.8	7.9	0
30-34	7.7	6.9	0.03	8	8	0
35-39	8.1	6.9	0.05	8.4	8.4	0
40-44	7.6	6.4	0.05	8	8	0
45-49	8.1	6.7	0.05	8.6	8.4	0.01
50-54	8.9	7.5	0.05	9.3	9.5	-0.01
55-59	8.4	7.4	0.04	8.8	8.8	0
60-64	6	6.5	-0.02	6.3	6.2	0
65-69	3.6	5.6	-0.1	3.6	3.4	0.01
70-74	3.2	5.7	-0.12	2.9	2.6	0.02
75-79	2.8	4.1	-0.07	2.3	2.1	0.01
80-84	2.5	2.9	-0.02	1.7	1.7	0.01
85-89	2.1	1.8	0.02	1.3	1.3	0
90-94	1.4	0.8	0.06	0.8	0.9	-0.01
95-99	0.6	0.2	0.06	0.3	0.3	0
**Sex**						
Female	56.7	49.4	0.15	57.2	59	-0.04

Table S6.123. *Continued.* Selected baseline characteristics for UK IMRD, for the medium-term risk of substance misuse or dependence

Characteristic	Before propensity score matching			After propensity score matching		
	Targets, %	Comparators, %	Standardized difference	Targets, %	Comparators, %	Standardized difference
**Race**						
race = Black or African American	0.2	0.3	-0.01	0.2	0.2	-0.01
race = White	4.5	6.7	-0.1	4.5	4.2	0.01
race = Asian Indian	0.9	0.9	0.01	0.8	0.8	0
race = Bangladeshi	0.2	0.1	0.02	0.2	0.1	0.01
race = Chinese	0.1	0.3	-0.06	0.1	0.1	0
race = Pakistani	1.7	0.6	0.11	1.9	1.9	0
race = African	0.7	0.8	-0.02	0.6	0.6	0
race = European	36.7	36.1	0.01	36.4	37.3	-0.02
race = Middle Eastern or North African	0.1	0.1	0	0.1	0.1	0
race = Arab	0.1	0.1	0	0.1	0.1	0
**Medical history (general)**						
Acute respiratory disease	4.4	2.6	0.1	4.3	4.8	-0.02
Attention deficit hyperactivity disorder	0.1	0.1	0	0.1	0.1	0
Chronic obstructive lung disease	0.7	0.6	0.02	0.6	0.6	0
Dementia	0.4	0.2	0.04	0.2	0.3	-0.01
Depressive disorder	1.4	1.1	0.03	1.4	1.6	-0.01
Diabetes mellitus	0.7	0.6	0.01	0.7	0.7	-0.01
Gastroesophageal reflux disease	0.3	0.2	0.02	0.3	0.4	0
Gastrointestinal hemorrhage	0.5	0.4	0.02	0.5	0.5	-0.01
Hyperlipidemia	0.2	0.2	0	0.2	0.2	-0.01
Hypertensive disorder	0.9	0.8	0.01	0.9	0.9	0
Lesion of liver	0.1	0.1	0.01	0.1	0.1	-0.01

Table S6.123. *Continued.* Selected baseline characteristics for UK IMRD, for the medium-term risk of substance misuse or dependence

Characteristic	Before propensity score matching			After propensity score matching		
	Targets, %	Comparators, %	Standardized difference	Targets, %	Comparators, %	Standardized difference
**Medical history (general)**						
Obesity	0.2	0.1	0.02	0.2	0.2	0
Osteoarthritis	0.8	0.7	0.01	0.8	0.8	0
Pneumonia	1.1	0.2	0.1	0.8	0.8	0
Psoriasis	0.4	0.3	0.02	0.3	0.4	0
Renal impairment	1.1	0.5	0.06	0.8	0.8	0
Rheumatoid arthritis	0.1	0.1	0	0.1	0.1	-0.01
Urinary tract infectious disease	2.1	1	0.09	1.8	2	-0.01
**Medical history (cardiovascular disease)**						
Atrial fibrillation	0.6	0.3	0.04	0.4	0.5	-0.01
Cerebrovascular disease	0.4	0.3	0.02	0.3	0.4	-0.01
Coronary arteriosclerosis	0.1	0.1	0	0.1	0.1	0.01
Heart disease	1.7	1.1	0.06	1.3	1.5	-0.02
Heart failure	0.4	0.2	0.04	0.3	0.3	0
Ischemic heart disease	0.4	0.3	0.02	0.4	0.4	-0.01
Peripheral vascular disease	0.1	0.1	0.01	0.1	0.1	0.01
Pulmonary embolism	0.2	0.1	0.03	0.2	0.1	0
Venous thrombosis	0.3	0.2	0.02	0.3	0.3	-0.01
**Medical history (neoplasms)**						
Malignant neoplastic disease	1	0.8	0.02	0.6	0.7	-0.01
Malignant tumor of breast	0.1	0.1	0	0.1	0.1	0

Table S6.123. *Continued.* Selected baseline characteristics for UK IMRD, for the medium-term risk of substance misuse or dependence

Characteristic	Before propensity score matching			After propensity score matching		
	Targets, %	Comparators, %	Standardized difference	Targets, %	Comparators, %	Standardized difference
**Medication use**						
Agents acting on the renin-angiotensin system	12.6	12.3	0.01	11.8	12	-0.01
Antibacterials for systemic use	38.4	27.7	0.23	37.3	39.6	-0.05
Antidepressants	22.2	15.9	0.16	21.6	23.2	-0.04
Antiepileptics	5.9	4.2	0.08	5.4	5.9	-0.02
Antiinflammatory and antirheumatic products	20.5	16.3	0.11	20	20.9	-0.02
Antineoplastic agents	1	1	0	0.9	1	0
Antipsoriatics	4.5	3	0.08	4.2	4.5	-0.02
Antithrombotic agents	10.1	9.2	0.03	8.3	8.5	0
Beta blocking agents	10	8.8	0.04	9	9.4	-0.01
Calcium channel blockers	8.3	8.3	0	7.7	7.5	0
Diuretics	6.9	6	0.03	5.6	5.7	0
Drugs for acid-related disorders	25.8	20.7	0.12	24.5	25.9	-0.03
Drugs for obstructive airway diseases	23.7	19.1	0.11	23.5	24.6	-0.03
Drugs used in diabetes	6.5	4.9	0.07	5.8	6	-0.01
Immunosuppressants	1	0.9	0.01	0.9	1	0
Lipid modifying agents	13.9	14.4	-0.01	12.6	12.8	0
Opioids	17.6	12.9	0.13	16.9	17.8	-0.02
Psycholeptics	10.3	7.6	0.09	9.2	9.9	-0.02
Psychostimulants, agents used for ADHD and nootropics	0.2	0.3	-0.01	0.2	0.3	-0.01

Table S6.124. Selected baseline characteristics for UK IMRD, for the medium-term risk of bipolar disorders

Characteristic	Before propensity score matching			After propensity score matching		
	Targets, %	Comparators, %	Standardized difference	Targets, %	Comparators, %	Standardized difference
**Age group (years)**						
0-4	1.7	3.9	-0.14	1.7	1.8	0
5-9	2.3	5.3	-0.16	2.4	2.4	0
10-14	3.8	5.2	-0.07	4	3.7	0.01
15-19	6	4.9	0.05	6.2	6.3	0
20-24	7.3	5.2	0.09	7.6	8.1	-0.02
25-29	7.6	6.3	0.05	7.8	7.9	0
30-34	7.7	7	0.03	8	8.1	0
35-39	8.1	6.9	0.05	8.4	8.3	0
40-44	7.6	6.5	0.05	7.9	8	0
45-49	8.1	6.8	0.05	8.6	8.4	0.01
50-54	8.9	7.4	0.05	9.3	9.5	-0.01
60-64	6	6.5	-0.02	6.3	6.3	0
65-69	3.6	5.7	-0.1	3.6	3.5	0
70-74	3.2	5.6	-0.12	2.9	2.6	0.02
75-79	2.8	4	-0.06	2.3	2.1	0.01
80-84	2.5	2.9	-0.02	1.7	1.7	0
85-89	2.1	1.7	0.03	1.3	1.3	0
90-94	1.4	0.7	0.06	0.8	0.8	-0.01
95-99	0.6	0.2	0.06	0.3	0.3	0
**Sex**						
Female	56.7	49.4	0.15	57.1	58.9	-0.04

Table S6.124. *Continued.* Selected baseline characteristics for UK IMRD, for the medium-term risk of bipolar disorders

Characteristic	Before propensity score matching			After propensity score matching		
	Targets, %	Comparators, %	Standardized difference	Targets, %	Comparators, %	Standardized difference
**Race**						
race = Asian	0.8	0.6	0.02	0.8	0.8	0
race = Black or African American	0.2	0.3	-0.02	0.2	0.2	-0.01
race = White	4.5	6.7	-0.1	4.5	4.2	0.01
race = Asian Indian	0.9	0.9	0	0.8	0.8	0
race = Bangladeshi	0.2	0.1	0.02	0.2	0.1	0.01
race = Chinese	0.1	0.3	-0.06	0.1	0.1	0
race = Pakistani	1.7	0.6	0.11	1.9	1.9	0
race = African	0.7	0.9	-0.02	0.6	0.6	0
race = European	36.7	36.2	0.01	36.4	37.3	-0.02
race = Middle Eastern or North African	0.1	0.1	0	0.1	0.1	0
**Medical history (general)**						
Acute respiratory disease	4.4	2.7	0.09	4.3	4.8	-0.02
Attention deficit hyperactivity disorder	0.1	0.1	0	0.1	0.1	0
Chronic obstructive lung disease	0.7	0.6	0.02	0.6	0.6	0
Dementia	0.4	0.2	0.04	0.2	0.3	-0.01
Depressive disorder	1.4	1.1	0.04	1.4	1.6	-0.01
Diabetes mellitus	0.7	0.6	0.01	0.7	0.7	-0.01
Gastroesophageal reflux disease	0.3	0.2	0.02	0.3	0.4	0
Gastrointestinal hemorrhage	0.5	0.4	0.02	0.5	0.5	-0.01
Hyperlipidemia	0.2	0.2	0	0.2	0.2	-0.01
Hypertensive disorder	0.9	0.8	0.01	0.9	0.9	0
Lesion of liver	0.1	0.1	0.01	0.1	0.1	-0.01
Obesity	0.2	0.1	0.02	0.2	0.2	0

Table S6.124. *Continued.* Selected baseline characteristics for UK IMRD, for the medium-term risk of bipolar disorders

**Characteristic**	**Before propensity score matching**			**After propensity score matching**		
	**Targets, %**	**Comparators, %**	**Standardized difference**	**Targets, %**	**Comparators, %**	**Standardized difference**
**Medical history (general)**						
Osteoarthritis	0.8	0.8	0.01	0.8	0.8	0
Pneumonia	1.1	0.2	0.11	0.8	0.8	-0.01
Psoriasis	0.4	0.3	0.01	0.3	0.4	0
Renal impairment	1.1	0.5	0.06	0.8	0.8	0
Rheumatoid arthritis	0.1	0.1	0.01	0.1	0.1	0
Urinary tract infectious disease	2.1	1	0.09	1.8	2	-0.01
**Medical history (cardiovascular disease)**						
Atrial fibrillation	0.6	0.3	0.04	0.4	0.5	-0.01
Cerebrovascular disease	0.4	0.2	0.03	0.3	0.4	-0.01
Coronary arteriosclerosis	0.1	0.1	0	0.1	0.1	0.01
Heart disease	1.7	1	0.06	1.3	1.5	-0.02
Heart failure	0.4	0.2	0.04	0.3	0.3	0
Ischemic heart disease	0.4	0.3	0.03	0.4	0.5	-0.01
Peripheral vascular disease	0.1	0.1	0.01	0.1	0.1	0.01
Pulmonary embolism	0.2	0.1	0.03	0.2	0.1	0
Venous thrombosis	0.3	0.2	0.01	0.3	0.3	-0.01
**Medical history (neoplasms)**						
Malignant neoplastic disease	1	0.7	0.03	0.7	0.7	-0.01
Malignant tumor of breast	0.1	0.1	0	0.1	0.1	0

Table S6.124. *Continued.* Selected baseline characteristics for UK IMRD, for the medium-term risk of bipolar disorders

Characteristic	Before propensity score matching			After propensity score matching		
	Targets, %	Comparators, %	Standardized difference	Targets, %	Comparators, %	Standardized difference
**Medication use**						
Agents acting on the renin-angiotensin system	12.6	12.1	0.01	11.8	12.1	-0.01
Antibacterials for systemic use	38.4	27.6	0.23	37.3	39.6	-0.05
Antidepressants	22.2	15.9	0.16	21.7	23.4	-0.04
Antiepileptics	5.9	4.2	0.08	5.4	5.9	-0.02
Antiinflammatory and antirheumatic products	20.5	16.4	0.11	20	21	-0.03
Antipsoriatics	4.5	3	0.08	4.2	4.5	-0.01
Antithrombotic agents	10.1	9	0.04	8.3	8.5	-0.01
Beta blocking agents	10	8.8	0.04	9.1	9.4	-0.01
Calcium channel blockers	8.3	8.2	0	7.7	7.6	0
Diuretics	6.9	6.1	0.03	5.6	5.7	-0.01
Drugs for acid-related disorders	25.8	20.6	0.12	24.5	26.1	-0.04
Drugs for obstructive airway diseases	23.7	19.1	0.11	23.5	24.7	-0.03
Drugs used in diabetes	6.5	4.9	0.07	5.8	6	-0.01
Immunosuppressants	1	0.9	0.01	0.9	1	0
Lipid modifying agents	13.9	14.1	0	12.7	12.8	0
Opioids	17.6	12.9	0.13	16.9	17.9	-0.03
Psycholeptics	10.3	7.8	0.09	9.3	9.9	-0.02
Psychostimulants, agents used for ADHD and nootropics	0.2	0.3	-0.01	0.2	0.3	-0.01

Table S6.125. Selected baseline characteristics for UK IMRD, for the medium-term risk of psychoses

Characteristic	Before propensity score matching			After propensity score matching		
	Targets, %	Comparators, %	Standardized difference	Targets, %	Comparators, %	Standardized difference
**Age group (years)**						
0-4	1.7	4	-0.14	1.7	1.8	0
5-9	2.3	5.2	-0.15	2.4	2.4	0
10-14	3.8	5.2	-0.07	4	3.8	0.01
15-19	6	5	0.04	6.2	6.3	0
20-24	7.3	5.2	0.09	7.6	8.1	-0.02
25-29	7.6	6.2	0.06	7.8	7.9	0
30-34	7.7	7	0.03	8	8.1	0
40-44	7.6	6.5	0.05	8	8	0
45-49	8.1	6.7	0.05	8.6	8.4	0.01
50-54	8.9	7.5	0.05	9.3	9.5	-0.01
60-64	6	6.5	-0.02	6.3	6.2	0
65-69	3.6	5.6	-0.1	3.6	3.5	0.01
70-74	3.2	5.6	-0.12	2.9	2.6	0.02
75-79	2.8	4.1	-0.07	2.3	2.1	0.01
80-84	2.5	2.8	-0.02	1.7	1.7	0
85-89	2.1	1.7	0.03	1.3	1.3	0
90-94	1.4	0.8	0.06	0.8	0.8	-0.01
95-99	0.6	0.2	0.06	0.3	0.3	0
**Sex**						
Female	56.7	49.4	0.14	57.1	58.9	-0.04

Table S6.125. *Continued.* Selected baseline characteristics for UK IMRD, for the medium-term risk of psychoses

Characteristic	Before propensity score matching			After propensity score matching		
	Targets, %	Comparators, %	Standardized difference	Targets, %	Comparators, %	Standardized difference
**Race**						
race = Asian	0.8	0.7	0.01	0.8	0.8	0
race = Black or African American	0.2	0.3	-0.02	0.2	0.2	-0.01
race = White	4.5	6.7	-0.1	4.5	4.2	0.01
race = Asian Indian	0.9	0.9	0.01	0.8	0.8	0
race = Bangladeshi	0.2	0.1	0.02	0.2	0.1	0.01
race = Chinese	0.1	0.4	-0.06	0.1	0.1	0
race = Pakistani	1.7	0.6	0.1	1.9	1.9	0
race = African	0.7	0.9	-0.02	0.6	0.6	0
race = European	36.7	36.1	0.01	36.4	37.4	-0.02
race = Middle Eastern or North African	0.1	0.1	0	0.1	0.1	0
**Medical history (general)**						
Acute respiratory disease	4.4	2.6	0.1	4.3	4.8	-0.02
Attention deficit hyperactivity disorder	0.1	0.1	0	0.1	0.1	0
Chronic obstructive lung disease	0.7	0.6	0.02	0.6	0.6	0
Dementia	0.4	0.2	0.04	0.2	0.3	-0.01
Depressive disorder	1.4	1	0.04	1.4	1.6	-0.01
Diabetes mellitus	0.7	0.6	0.02	0.7	0.7	-0.01
Gastroesophageal reflux disease	0.3	0.2	0.02	0.3	0.4	0
Gastrointestinal hemorrhage	0.5	0.4	0.02	0.5	0.5	-0.01
Hyperlipidemia	0.2	0.2	0	0.2	0.2	-0.01
Hypertensive disorder	0.9	0.8	0.01	0.9	0.9	0
Lesion of liver	0.1	0.1	0.01	0.1	0.1	-0.01
Obesity	0.2	0.1	0.02	0.2	0.2	0
Osteoarthritis	0.8	0.8	0.01	0.8	0.8	0

Table S6.125. *Continued.* Selected baseline characteristics for UK IMRD, for the medium-term risk of psychoses

Characteristic	Before propensity score matching			After propensity score matching		
	Targets, %	Comparators, %	Standardized difference	Targets, %	Comparators, %	Standardized difference
**Medical history (general)**						
Pneumonia	1.1	0.2	0.11	0.8	0.8	-0.01
Psoriasis	0.4	0.3	0.01	0.4	0.4	0
Renal impairment	1.1	0.5	0.06	0.8	0.8	0
Rheumatoid arthritis	0.1	0.1	0.01	0.1	0.1	0
Urinary tract infectious disease	2.1	1	0.09	1.8	1.9	-0.01
**Medical history (cardiovascular disease)**						
Atrial fibrillation	0.6	0.3	0.04	0.4	0.5	-0.01
Cerebrovascular disease	0.4	0.2	0.03	0.3	0.4	-0.01
Coronary arteriosclerosis	0.1	0.1	0	0.1	0.1	0.01
Heart disease	1.7	1.1	0.06	1.3	1.5	-0.02
Heart failure	0.4	0.2	0.04	0.3	0.3	-0.01
Ischemic heart disease	0.4	0.3	0.03	0.4	0.4	-0.01
Peripheral vascular disease	0.1	0.1	0.01	0.1	0.1	0.01
Pulmonary embolism	0.2	0.1	0.03	0.2	0.1	0
Venous thrombosis	0.3	0.2	0.02	0.3	0.3	-0.02
**Medical history (neoplasms)**						
Malignant neoplastic disease	1	0.8	0.02	0.7	0.7	-0.01
Malignant tumor of breast	0.1	0.1	0	0.1	0.1	0

Table S6.125. *Continued.* Selected baseline characteristics for UK IMRD, for the medium-term risk of psychoses

Characteristic	Before propensity score matching			After propensity score matching		
	Targets, %	Comparators, %	Standardized difference	Targets, %	Comparators, %	Standardized difference
**Medication use**						
Agents acting on the renin-angiotensin system	12.6	12.2	0.01	11.8	12	-0.01
Antibacterials for systemic use	38.4	27.8	0.23	37.3	39.5	-0.05
Antidepressants	22.2	15.8	0.16	21.7	23.3	-0.04
Antiepileptics	5.9	4.3	0.08	5.4	5.9	-0.02
Antiinflammatory and antirheumatic products	20.5	16.3	0.11	20	20.9	-0.02
Antineoplastic agents	1	1	0	0.9	1	0
Antipsoriatics	4.5	2.9	0.08	4.2	4.5	-0.01
Antithrombotic agents	10.1	9.2	0.03	8.3	8.5	0
Beta blocking agents	10	8.9	0.04	9.1	9.4	-0.01
Calcium channel blockers	8.3	8.3	0	7.7	7.6	0
Diuretics	6.9	6.1	0.03	5.6	5.7	0
Drugs for acid-related disorders	25.8	20.6	0.12	24.5	26	-0.03
Drugs for obstructive airway diseases	23.7	19.1	0.11	23.5	24.6	-0.03
Drugs used in diabetes	6.5	4.9	0.07	5.8	6	-0.01
Immunosuppressants	1	0.9	0.01	0.9	1	0
Lipid modifying agents	13.9	14.2	-0.01	12.6	12.7	0
Opioids	17.6	12.9	0.13	16.9	17.9	-0.02
Psycholeptics	10.3	7.7	0.09	9.3	9.9	-0.02
Psychostimulants, agents used for ADHD and nootropics	0.2	0.3	-0.01	0.2	0.3	-0.01

Table S6.126. Selected baseline characteristics for UK IMRD, for the medium-term risk of personality disorders

Characteristic	Before propensity score matching			After propensity score matching		
	**Targets, %**	**Comparators, %**	**Standardized difference**	**Targets, %**	**Comparators, %**	**Standardized difference**
**Age group (years)**						
0-4	1.7	3.9	-0.14	1.7	1.8	0
5-9	2.3	5.3	-0.15	2.4	2.4	0
10-14	3.8	5.3	-0.07	4	3.8	0.01
15-19	6	5	0.04	6.3	6.3	0
20-24	7.3	5.2	0.09	7.6	8.1	-0.02
25-29	7.6	6.2	0.06	7.8	7.9	0
30-34	7.7	6.9	0.03	8	8.1	0
35-39	8.1	6.9	0.05	8.4	8.4	0
40-44	7.6	6.4	0.05	8	8	0
45-49	8.1	6.7	0.05	8.6	8.4	0.01
50-54	8.9	7.4	0.06	9.3	9.5	-0.01
60-64	6	6.5	-0.02	6.3	6.2	0
65-69	3.6	5.7	-0.1	3.6	3.5	0.01
70-74	3.2	5.7	-0.12	2.9	2.6	0.02
75-79	2.8	4	-0.07	2.3	2.1	0.01
80-84	2.5	2.8	-0.02	1.7	1.7	0
85-89	2.1	1.7	0.03	1.3	1.3	0
90-94	1.4	0.8	0.06	0.8	0.9	-0.01
95-99	0.6	0.2	0.06	0.3	0.3	0
**Sex**						
Female	56.7	49.5	0.14	57.1	59	-0.04

Table S6.126. *Continued.* Selected baseline characteristics for UK IMRD, for the medium-term risk of personality disorders

Characteristic	Before propensity score matching			After propensity score matching		
	Targets, %	Comparators, %	Standardized difference	Targets, %	Comparators, %	Standardized difference
**Race**						
race = Black or African American	0.2	0.3	-0.02	0.2	0.2	-0.01
race = White	4.5	6.6	-0.09	4.5	4.2	0.01
race = Asian Indian	0.9	0.9	0.01	0.7	0.8	0
race = Bangladeshi	0.2	0.1	0.02	0.2	0.1	0.01
race = Chinese	0.1	0.3	-0.06	0.1	0.1	0
race = Pakistani	1.7	0.6	0.11	1.9	1.9	0
race = African	0.7	0.9	-0.02	0.6	0.6	0
race = European	36.7	36.2	0.01	36.4	37.3	-0.02
race = Arab	0.1	0.1	0	0.1	0.1	0
**Medical history (general)**						
Acute respiratory disease	4.4	2.6	0.1	4.3	4.8	-0.02
Attention deficit hyperactivity disorder	0.1	0.1	0	0.1	0.1	0
Chronic obstructive lung disease	0.7	0.6	0.01	0.6	0.6	0
Dementia	0.4	0.2	0.04	0.2	0.3	-0.01
Depressive disorder	1.4	1.1	0.04	1.4	1.6	-0.01
Diabetes mellitus	0.7	0.6	0.01	0.7	0.7	-0.01
Gastroesophageal reflux disease	0.3	0.2	0.03	0.3	0.4	0
Gastrointestinal hemorrhage	0.5	0.4	0.02	0.5	0.5	-0.01
Hyperlipidemia	0.2	0.2	0	0.2	0.2	-0.01
Hypertensive disorder	0.9	0.8	0.01	0.9	0.9	0
Lesion of liver	0.1	0.1	0.01	0.1	0.1	-0.01
Obesity	0.2	0.1	0.02	0.2	0.2	0
Osteoarthritis	0.8	0.8	0	0.8	0.8	0

Table S6.126. *Continued.* Selected baseline characteristics for UK IMRD, for the medium-term risk of personality disorders

Characteristic	Before propensity score matching			After propensity score matching		
	Targets, %	Comparators, %	Standardized difference	Targets, %	Comparators, %	Standardized difference
**Medical history (general)**						
Pneumonia	1.1	0.2	0.11	0.8	0.8	-0.01
Psoriasis	0.4	0.3	0.01	0.4	0.4	0
Renal impairment	1.1	0.5	0.07	0.8	0.8	0
Rheumatoid arthritis	0.1	0.1	0.01	0.1	0.1	0
Urinary tract infectious disease	2.1	1	0.1	1.8	2	-0.01
**Medical history (cardiovascular disease)**						
Atrial fibrillation	0.6	0.4	0.04	0.4	0.5	-0.01
Cerebrovascular disease	0.4	0.3	0.03	0.3	0.4	-0.01
Coronary arteriosclerosis	0.1	0.1	0	0.1	0.1	0.01
Heart disease	1.7	1.1	0.06	1.3	1.5	-0.02
Heart failure	0.4	0.2	0.04	0.3	0.3	-0.01
Ischemic heart disease	0.4	0.3	0.02	0.4	0.4	-0.01
Peripheral vascular disease	0.1	0.1	0.01	0.1	0.1	0.01
Pulmonary embolism	0.2	0.1	0.03	0.2	0.1	0
Venous thrombosis	0.3	0.2	0.02	0.3	0.3	-0.01
**Medical history (neoplasms)**						
Malignant neoplastic disease	1	0.7	0.03	0.7	0.7	-0.01
Malignant tumor of breast	0.1	0.1	0	0.1	0.1	0

Table S6.126. *Continued.* Selected baseline characteristics for UK IMRD, for the medium-term risk of personality disorders

Characteristic	Before propensity score matching			After propensity score matching		
	Targets, %	Comparators, %	Standardized difference	Targets, %	Comparators, %	Standardized difference
**Medication use**						
Agents acting on the renin-angiotensin system	12.6	12.2	0.01	11.8	12	-0.01
Antibacterials for systemic use	38.4	27.6	0.23	37.3	39.6	-0.05
Antidepressants	22.2	15.9	0.16	21.7	23.3	-0.04
Antiepileptics	5.9	4.3	0.07	5.4	5.9	-0.02
Antiinflammatory and antirheumatic products	20.5	16.4	0.11	20	20.9	-0.02
Antineoplastic agents	1	1	0	0.9	1	0
Antipsoriatics	4.5	3	0.08	4.2	4.5	-0.02
Antithrombotic agents	10.1	9.1	0.04	8.3	8.5	0
Beta blocking agents	10	8.8	0.04	9.1	9.4	-0.01
Diuretics	6.9	6.1	0.03	5.6	5.7	0
Drugs for acid-related disorders	25.8	20.6	0.12	24.5	26	-0.03
Drugs for obstructive airway diseases	23.7	19	0.11	23.5	24.7	-0.03
Drugs used in diabetes	6.5	4.9	0.07	5.8	5.9	-0.01
Immunosuppressants	1	0.9	0.01	0.9	1	0
Lipid modifying agents	13.9	14.2	-0.01	12.7	12.8	0
Opioids	17.6	13	0.13	16.9	17.8	-0.02
Psycholeptics	10.3	7.7	0.09	9.2	9.9	-0.02
Psychostimulants, agents used for ADHD and nootropics	0.2	0.3	-0.01	0.2	0.3	-0.01

Table S6.127. Selected baseline characteristics for UK IMRD, for the medium-term risk of self-harm and suicide

Characteristic	Before propensity score matching			After propensity score matching		
	Targets, %	Comparators, %	Standardized difference	Targets, %	Comparators, %	Standardized difference
**Age group (years)**						
0-4	1.7	3.9	-0.14	1.7	1.8	0
5-0	2.3	5.2	-0.15	2.4	2.4	0
10-14	3.8	5.2	-0.07	3.9	3.7	0.01
15-19	6	5	0.04	6.2	6.3	0
20-24	7.3	5.1	0.09	7.6	8.1	-0.02
25-29	7.6	6.2	0.06	7.8	7.9	0
30-34	7.7	7	0.03	8	8.1	0
40-44	7.6	6.5	0.04	8	8	0
45-49	8.1	6.7	0.06	8.6	8.4	0.01
50-54	8.9	7.4	0.05	9.3	9.5	-0.01
60-64	6	6.5	-0.02	6.3	6.3	0
65-69	3.6	5.6	-0.1	3.6	3.5	0.01
70-74	3.2	5.7	-0.12	2.9	2.6	0.02
75-79	2.8	4.1	-0.07	2.3	2.1	0.01
80-84	2.5	2.9	-0.02	1.7	1.7	0
85-89	2.1	1.8	0.02	1.3	1.3	0
90-94	1.4	0.7	0.07	0.8	0.9	-0.01
95-99	0.6	0.2	0.06	0.3	0.3	0
**Sex**						
Female	56.7	49.3	0.15	57.1	59	-0.04

Table S6.127. *Continued.* Selected baseline characteristics for UK IMRD, for the medium-term risk of self-harm and suicide

Characteristic	Before propensity score matching			After propensity score matching		
	Targets, %	Comparators, %	Standardized difference	Targets, %	Comparators, %	Standardized difference
**Race**						
race = Asian	0.8	0.7	0.01	0.8	0.8	0
race = Black or African American	0.2	0.3	-0.02	0.2	0.2	-0.01
race = White	4.5	6.6	-0.09	4.5	4.2	0.02
race = Asian Indian	0.9	0.9	0.01	0.8	0.8	0
race = Bangladeshi	0.2	0.1	0.01	0.2	0.1	0.01
race = Chinese	0.1	0.4	-0.06	0.1	0.1	0
race = Pakistani	1.7	0.6	0.11	1.9	1.9	0
race = African	0.7	0.9	-0.02	0.6	0.6	0
race = European	36.7	36.3	0.01	36.4	37.4	-0.02
**Medical history (general)**						
Acute respiratory disease	4.4	2.6	0.1	4.3	4.8	-0.02
Attention deficit hyperactivity disorder	0.1	0.1	0	0.1	0.1	0
Chronic obstructive lung disease	0.7	0.6	0.02	0.6	0.6	0
Dementia	0.4	0.2	0.04	0.2	0.3	-0.01
Depressive disorder	1.4	1.1	0.03	1.4	1.6	-0.01
Diabetes mellitus	0.7	0.6	0.01	0.7	0.7	-0.01
Gastroesophageal reflux disease	0.3	0.2	0.03	0.3	0.4	0
Gastrointestinal hemorrhage	0.5	0.4	0.02	0.5	0.5	-0.01
Hyperlipidemia	0.2	0.2	0	0.2	0.2	-0.01
Hypertensive disorder	0.9	0.8	0.01	0.9	0.9	0
Lesion of liver	0.1	0.1	0.01	0.1	0.1	-0.01
Obesity	0.2	0.1	0.01	0.2	0.2	0
Osteoarthritis	0.8	0.7	0.01	0.8	0.8	0
Pneumonia	1.1	0.2	0.11	0.7	0.8	-0.01

Table S6.127. *Continued.* Selected baseline characteristics for UK IMRD, for the medium-term risk of self-harm and suicide

Characteristic	Before propensity score matching			After propensity score matching		
	**Targets, %**	**Comparators, %**	**Standardized difference**	**Targets, %**	**Comparators, %**	**Standardized difference**
**Medical history (general)**						
Psoriasis	0.4	0.3	0.01	0.4	0.4	0
Renal impairment	1.1	0.5	0.07	0.8	0.8	0
Rheumatoid arthritis	0.1	0.1	0.01	0.1	0.1	-0.01
Urinary tract infectious disease	2.1	1	0.09	1.8	2	-0.01
**Medical history (cardiovascular disease)**						
Atrial fibrillation	0.6	0.3	0.04	0.4	0.5	-0.01
Cerebrovascular disease	0.4	0.3	0.03	0.3	0.4	-0.01
Heart disease	1.7	1.1	0.06	1.3	1.5	-0.01
Heart failure	0.4	0.2	0.04	0.3	0.3	-0.01
Ischemic heart disease	0.4	0.3	0.02	0.4	0.4	-0.01
Peripheral vascular disease	0.1	0.1	0.01	0.1	0.1	0.01
Pulmonary embolism	0.2	0.1	0.03	0.2	0.1	0
Venous thrombosis	0.3	0.2	0.02	0.3	0.3	-0.01
**Medical history (neoplasms)**						
Malignant neoplastic disease	1	0.8	0.02	0.6	0.7	-0.01
Malignant tumor of breast	0.1	0.1	0	0.1	0.1	0
**Medication use**						
Agents acting on the renin-angiotensin system	12.6	12.3	0.01	11.8	12	-0.01
Antibacterials for systemic use	38.4	27.7	0.23	37.3	39.5	-0.05
Antidepressants	22.2	15.9	0.16	21.7	23.3	-0.04
Antiepileptics	5.9	4.3	0.07	5.4	5.9	-0.03
Antiinflammatory and antirheumatic products	20.5	16.4	0.11	20	21	-0.02

Table S6.127. *Continued.* Selected baseline characteristics for UK IMRD, for the medium-term risk of self-harm and suicide

Characteristic	Before propensity score matching			After propensity score matching		
	Targets, %	Comparators, %	Standardized difference	Targets, %	Comparators, %	Standardized difference
**Medication use**						
Antineoplastic agents	1	1	0	0.9	1	0
Antipsoriatics	4.5	3	0.08	4.2	4.5	-0.02
Antithrombotic agents	10.1	9.2	0.03	8.3	8.4	0
Beta blocking agents	10	8.9	0.04	9	9.4	-0.01
Calcium channel blockers	8.3	8.4	0	7.7	7.6	0
Diuretics	6.9	6.1	0.03	5.6	5.7	0
Drugs for acid-related disorders	25.8	20.6	0.12	24.5	26	-0.03
Drugs for obstructive airway diseases	23.7	19.1	0.11	23.5	24.6	-0.03
Drugs used in diabetes	6.5	4.9	0.07	5.8	5.9	-0.01
Immunosuppressants	1	0.9	0.01	0.9	1	-0.01
Lipid modifying agents	13.9	14.4	-0.01	12.7	12.7	0
Opioids	17.6	12.9	0.13	16.9	17.9	-0.03
Psycholeptics	10.3	7.7	0.09	9.2	9.9	-0.02
Psychostimulants, agents used for ADHD and nootropics	0.2	0.3	-0.01	0.2	0.3	-0.01

Table S6.128. Selected baseline characteristics for UK IMRD, for the medium-term risk of sleep disorders

Characteristic	Before propensity score matching			After propensity score matching		
	Targets, %	Comparators, %	Standardized difference	Targets, %	Comparators, %	Standardized difference
**Age group (years)**						
0-4	1.7	3.8	-0.13	1.7	1.8	0
5-9	2.3	5.3	-0.16	2.4	2.4	0.01
10-14	3.8	5.2	-0.07	4	3.8	0.01
15-19	6	5	0.04	6.3	6.4	0
20-24	7.3	5.2	0.09	7.7	8.1	-0.02
25-29	7.6	6.1	0.06	7.8	7.9	0
30-34	7.7	6.9	0.03	8	8.1	0
35-39	8.1	6.9	0.04	8.4	8.4	0
40-44	7.6	6.5	0.04	8	8	0
45-49	8.1	6.8	0.05	8.6	8.4	0.01
50-54	8.9	7.4	0.06	9.2	9.5	-0.01
55-59	8.4	7.4	0.04	8.8	8.7	0
60-64	6	6.5	-0.02	6.3	6.2	0
65-69	3.6	5.6	-0.1	3.6	3.5	0
70-74	3.2	5.7	-0.12	2.9	2.6	0.02
75-79	2.8	4.1	-0.07	2.3	2.1	0.01
80-84	2.5	2.9	-0.02	1.7	1.7	0
85-89	2.1	1.7	0.03	1.3	1.3	0
90-94	1.4	0.8	0.06	0.8	0.8	-0.01
95-99	0.6	0.2	0.06	0.3	0.3	0
**Sex**						
Female	56.7	49.5	0.14	57.1	58.9	-0.04

Table S6.128. *Continued.* Selected baseline characteristics for UK IMRD, for the medium-term risk of sleep disorders

Characteristic	Before propensity score matching			After propensity score matching		
	Targets, %	Comparators, %	Standardized difference	Targets, %	Comparators, %	Standardized difference
**Race**						
race = Asian	0.8	0.6	0.02	0.8	0.8	0
race = Black or African American	0.2	0.3	-0.02	0.2	0.2	-0.01
race = White	4.5	6.8	-0.1	4.5	4.2	0.01
race = Asian Indian	0.9	0.9	0.01	0.8	0.8	0
race = Bangladeshi	0.2	0.1	0.02	0.2	0.1	0.01
race = Chinese	0.1	0.3	-0.06	0.1	0.1	0
race = Pakistani	1.7	0.6	0.11	1.9	1.9	0
race = African	0.7	0.8	-0.02	0.6	0.6	0
race = European	36.7	36.2	0.01	36.4	37.4	-0.02
race = Middle Eastern or North African	0.1	0.1	0	0.1	0.1	0
**Medical history (general)**						
Acute respiratory disease	4.4	2.7	0.09	4.3	4.7	-0.02
Attention deficit hyperactivity disorder	0.1	0.1	0	0.1	0.1	0
Chronic obstructive lung disease	0.7	0.6	0.01	0.6	0.6	-0.01
Dementia	0.4	0.2	0.04	0.2	0.3	-0.01
Depressive disorder	1.4	1	0.04	1.4	1.6	-0.01
Diabetes mellitus	0.7	0.6	0.01	0.7	0.7	-0.01
Gastroesophageal reflux disease	0.3	0.2	0.02	0.3	0.3	0
Gastrointestinal hemorrhage	0.5	0.4	0.02	0.5	0.5	-0.01
Hyperlipidemia	0.2	0.2	0	0.2	0.2	-0.01
Hypertensive disorder	0.9	0.8	0.01	0.9	0.9	0
Lesion of liver	0.1	0.1	0.01	0.1	0.1	-0.01

Table S6.128. *Continued.* Selected baseline characteristics for UK IMRD, for the medium-term risk of sleep disorders

Characteristic	Before propensity score matching			After propensity score matching		
	Targets, %	Comparators, %	Standardized difference	Targets, %	Comparators, %	Standardized difference
**Medical history (general)**						
Obesity	0.2	0.1	0.02	0.2	0.2	0
Osteoarthritis	0.8	0.7	0.01	0.7	0.8	0
Pneumonia	1.1	0.2	0.11	0.7	0.8	-0.01
Psoriasis	0.4	0.3	0.02	0.3	0.4	0
Renal impairment	1.1	0.5	0.06	0.8	0.8	0
Rheumatoid arthritis	0.1	0.1	0	0.1	0.1	0
Urinary tract infectious disease	2.1	1	0.09	1.8	1.9	-0.01
**Medical history (cardiovascular disease)**						
Atrial fibrillation	0.6	0.3	0.04	0.4	0.5	-0.01
Cerebrovascular disease	0.4	0.3	0.02	0.3	0.4	-0.01
Heart disease	1.7	1.1	0.06	1.3	1.5	-0.02
Heart failure	0.4	0.2	0.04	0.2	0.3	-0.01
Ischemic heart disease	0.4	0.3	0.02	0.4	0.4	-0.01
Peripheral vascular disease	0.1	0.1	0.01	0.1	0.1	0.01
Pulmonary embolism	0.2	0.1	0.03	0.2	0.1	0
Venous thrombosis	0.3	0.2	0.01	0.3	0.3	-0.02
**Medical history (neoplasms)**						
Malignant neoplastic disease	1	0.8	0.02	0.6	0.7	-0.01
Malignant tumor of breast	0.1	0.1	0	0.1	0.1	0

Table S6.128. *Continued.* Selected baseline characteristics for UK IMRD, for the medium-term risk of sleep disorders

Characteristic	Before propensity score matching			After propensity score matching		
	**Targets, %**	**Comparators, %**	**Standardized difference**	**Targets, %**	**Comparators, %**	**Standardized difference**
**Medication use**						
Agents acting on the renin-angiotensin system	12.6	12.3	0.01	11.7	12	-0.01
Antibacterials for systemic use	38.4	27.7	0.23	37.1	39.4	-0.05
Antidepressants	22.2	15.9	0.16	21.4	23.1	-0.04
Antiepileptics	5.9	4.3	0.07	5.3	5.8	-0.02
Antiinflammatory and antirheumatic products	20.5	16.4	0.11	19.9	20.8	-0.02
Antineoplastic agents	1	1	-0.01	0.9	0.9	0
Antipsoriatics	4.5	3	0.08	4.2	4.5	-0.01
Antithrombotic agents	10.1	9.2	0.03	8.2	8.3	0
Beta blocking agents	10	8.8	0.04	8.9	9.3	-0.01
Calcium channel blockers	8.3	8.3	0	7.6	7.5	0
Diuretics	6.9	6.1	0.03	5.5	5.6	0
Drugs for acid-related disorders	25.8	20.7	0.12	24.3	25.7	-0.03
Drugs for obstructive airway diseases	23.7	19.2	0.11	23.3	24.5	-0.03
Drugs used in diabetes	6.5	4.9	0.07	5.7	5.9	-0.01
Immunosuppressants	1	0.9	0.01	1	1	0
Lipid modifying agents	13.9	14.3	-0.01	12.5	12.7	0
Opioids	17.6	12.9	0.13	16.7	17.6	-0.02
Psycholeptics	10.3	7.7	0.09	9	9.6	-0.02
Psychostimulants, agents used for ADHD and nootropics	0.2	0.3	-0.01	0.2	0.3	-0.01

Table S6.129. Selected baseline characteristics for UK IMRD, for the medium-term risk of dementia

Characteristic	Before propensity score matching			After propensity score matching		
	Targets, %	Comparators, %	Standardized difference	Targets, %	Comparators, %	Standardized difference
**Age group (years)**						
0-4	1.7	3.8	-0.13	1.7	1.8	0
5-9	2.3	5.2	-0.15	2.5	2.4	0
10-14	3.8	5.3	-0.07	4	3.8	0.01
15-19	6	5	0.04	6.3	6.4	0
20-24	7.3	5.3	0.08	7.7	8.2	-0.02
25-29	7.6	6.3	0.05	7.9	8	0
30-34	7.7	6.8	0.03	8.1	8.1	0
35-39	8.1	7	0.04	8.5	8.4	0
40-44	7.6	6.4	0.05	8	8.1	0
45-49	8.1	6.8	0.05	8.7	8.5	0.01
50-54	8.9	7.3	0.06	9.4	9.6	-0.01
55-59	8.4	7.4	0.04	8.9	8.9	0
60-64	6	6.5	-0.02	6.4	6.3	0
65-69	3.6	5.7	-0.1	3.6	3.5	0.01
70-74	3.2	5.6	-0.12	2.9	2.6	0.02
75-79	2.8	4	-0.07	2.1	2	0.01
80-84	2.5	2.9	-0.02	1.5	1.5	0
85-89	2.1	1.8	0.02	1.1	1.1	-0.01
90-94	1.4	0.8	0.06	0.6	0.7	-0.01
95-99	0.6	0.2	0.06	0.2	0.2	0
**Sex**						
Female	56.7	49.3	0.15	57	58.9	-0.04

Table S6.129. *Continued.* Selected baseline characteristics for UK IMRD, for the medium-term risk of dementia

Characteristic	Before propensity score matching			After propensity score matching		
	Targets, %	Comparators, %	Standardized difference	Targets, %	Comparators, %	Standardized difference
**Race**						
race = Asian	0.8	0.6	0.02	0.8	0.8	0
race = Black or African American	0.2	0.3	-0.02	0.2	0.2	-0.01
race = White	4.5	6.7	-0.1	4.5	4.2	0.01
race = Asian Indian	0.9	0.9	0	0.8	0.8	0
race = Bangladeshi	0.2	0.1	0.01	0.2	0.1	0.01
race = Chinese	0.1	0.4	-0.06	0.1	0.1	0
race = Pakistani	1.7	0.6	0.11	1.9	1.9	0
race = African	0.7	0.9	-0.02	0.6	0.6	0
race = European	36.7	36.1	0.01	36.4	37.4	-0.02
race = Arab	0.1	0.1	0	0.1	0.1	0
**Medical history (general)**						
Acute respiratory disease	4.4	2.6	0.1	4.3	4.8	-0.02
Attention deficit hyperactivity disorder	0.1	0.1	0.01	0.1	0.1	0
Chronic obstructive lung disease	0.7	0.6	0.02	0.6	0.6	-0.01
Depressive disorder	1.4	1.1	0.04	1.4	1.6	-0.01
Diabetes mellitus	0.7	0.6	0.01	0.7	0.7	-0.01
Gastroesophageal reflux disease	0.3	0.2	0.03	0.3	0.4	0
Gastrointestinal hemorrhage	0.5	0.4	0.02	0.5	0.5	-0.01
Hyperlipidemia	0.2	0.2	0	0.2	0.2	0
Hypertensive disorder	0.9	0.8	0.01	0.9	0.9	0
Lesion of liver	0.1	0.1	0.01	0.1	0.1	-0.01
Obesity	0.2	0.1	0.02	0.2	0.2	0

Table S6.129. *Continued.* Selected baseline characteristics for UK IMRD, for the medium-term risk of dementia

Characteristic	Before propensity score matching			After propensity score matching		
	Targets, %	Comparators, %	Standardized difference	Targets, %	Comparators, %	Standardized difference
**Medical history (general)**						
Osteoarthritis	0.8	0.8	0	0.8	0.8	0
Pneumonia	1.1	0.2	0.1	0.7	0.8	0
Psoriasis	0.4	0.3	0.02	0.4	0.4	0
Renal impairment	1.1	0.5	0.07	0.8	0.7	0
Rheumatoid arthritis	0.1	0.1	0.01	0.1	0.1	0
Urinary tract infectious disease	2.1	1	0.09	1.7	1.9	-0.01
**Medical history (cardiovascular disease)**						
Atrial fibrillation	0.6	0.4	0.04	0.4	0.5	0
Cerebrovascular disease	0.4	0.2	0.03	0.3	0.4	-0.01
Heart disease	1.7	1.1	0.05	1.3	1.4	-0.01
Heart failure	0.4	0.2	0.04	0.2	0.3	0
Ischemic heart disease	0.4	0.3	0.03	0.4	0.4	-0.01
Peripheral vascular disease	0.1	0.1	0.01	0.1	0.1	0.01
Pulmonary embolism	0.2	0.1	0.03	0.2	0.1	0
Venous thrombosis	0.3	0.2	0.02	0.2	0.3	-0.02
**Medical history (neoplasms)**						
Malignant neoplastic disease	1	0.8	0.02	0.6	0.7	-0.01
Malignant tumor of breast	0.1	0.1	0	0.1	0.1	0

Table S6.129. *Continued.* Selected baseline characteristics for UK IMRD, for the medium-term risk of dementia

Characteristic	Before propensity score matching			After propensity score matching		
	Targets, %	Comparators, %	Standardized difference	Targets, %	Comparators, %	Standardized difference
**Medication use**						
Agents acting on the renin-angiotensin system	12.6	12.2	0.01	11.7	11.9	-0.01
Antibacterials for systemic use	38.4	27.7	0.23	36.9	39.2	-0.05
Antidepressants	22.2	15.9	0.16	21.5	23.1	-0.04
Antiepileptics	5.9	4.3	0.07	5.3	5.8	-0.03
Antiinflammatory and antirheumatic products	20.5	16.2	0.11	19.9	20.8	-0.02
Antineoplastic agents	1	1	0	0.9	1	0
Antipsoriatics	4.5	3	0.08	4.1	4.4	-0.02
Antithrombotic agents	10.1	9.1	0.04	7.9	8.1	-0.01
Beta blocking agents	10	8.8	0.04	8.9	9.2	-0.01
Diuretics	6.9	6.1	0.03	5.4	5.5	0
Drugs for acid-related disorders	25.8	20.5	0.12	24.1	25.6	-0.04
Drugs for obstructive airway diseases	23.7	19.1	0.11	23.5	24.6	-0.03
Drugs used in diabetes	6.5	4.9	0.07	5.7	5.9	-0.01
Immunosuppressants	1	0.9	0.01	0.9	1	-0.01
Lipid modifying agents	13.9	14.3	-0.01	12.3	12.4	0
Opioids	17.6	12.8	0.13	16.8	17.7	-0.03
Psycholeptics	10.3	7.8	0.09	8.9	9.7	-0.03
Psychostimulants, agents used for ADHD and nootropics	0.2	0.3	-0.01	0.2	0.3	-0.01

Table S6.130. Selected baseline characteristics for UK IMRD, for the medium-term risk of neurodevelopmental disorders

Characteristic	Before propensity score matching			After propensity score matching		
	Targets, %	Comparators, %	Standardized difference	Targets, %	Comparators, %	Standardized difference
**Age group (years)**						
0-4	1.7	3.9	-0.14	1.7	1.8	0
5-9	2.3	5.2	-0.15	2.4	2.3	0.01
10-14	3.8	5.2	-0.07	3.9	3.7	0.01
15-19	6	5	0.04	6.2	6.3	0
20-24	7.3	5.2	0.09	7.6	8.1	-0.02
25-29	7.6	6.2	0.06	7.8	7.9	0
30-34	7.7	7	0.03	8	8.1	0
35-39	8.1	6.9	0.05	8.4	8.3	0
40-44	7.6	6.5	0.04	8	8.1	0
45-49	8.1	6.7	0.05	8.6	8.4	0.01
50-54	8.9	7.4	0.06	9.3	9.5	-0.01
60-64	6	6.4	-0.01	6.3	6.3	0
65-69	3.6	5.6	-0.1	3.6	3.5	0.01
70-74	3.2	5.7	-0.12	2.9	2.6	0.02
75-79	2.8	4	-0.07	2.3	2.1	0.01
80-84	2.5	2.9	-0.03	1.7	1.7	0.01
85-89	2.1	1.7	0.03	1.3	1.3	0
90-94	1.4	0.8	0.06	0.8	0.8	-0.01
95-99	0.6	0.2	0.06	0.3	0.3	0
**Sex**						
Female	56.7	49.3	0.15	57.2	59	-0.04

Table S6.130. *Continued.* Selected baseline characteristics for UK IMRD, for the medium-term risk of neurodevelopmental disorders

Characteristic	Before propensity score matching			After propensity score matching		
	Targets, %	Comparators, %	Standardized difference	Targets, %	Comparators, %	Standardized difference
**Race**						
race = Asian	0.8	0.7	0.01	0.8	0.8	0
race = Black or African American	0.2	0.4	-0.02	0.2	0.2	-0.01
race = White	4.5	6.6	-0.09	4.5	4.2	0.01
race = Bangladeshi	0.2	0.1	0.01	0.2	0.1	0.01
race = Chinese	0.1	0.4	-0.06	0.1	0.1	0
race = Pakistani	1.7	0.6	0.11	1.9	1.9	0
race = African	0.7	0.9	-0.02	0.6	0.6	0
race = European	36.7	36.2	0.01	36.4	37.3	-0.02
**Medical history (general)**						
Acute respiratory disease	4.4	2.6	0.1	4.3	4.8	-0.02
Chronic obstructive lung disease	0.7	0.6	0.02	0.6	0.6	0
Dementia	0.4	0.2	0.04	0.2	0.3	-0.01
Depressive disorder	1.4	1.1	0.03	1.4	1.6	-0.01
Diabetes mellitus	0.7	0.6	0.01	0.7	0.7	-0.01
Gastroesophageal reflux disease	0.3	0.2	0.02	0.3	0.4	0
Gastrointestinal hemorrhage	0.5	0.4	0.02	0.5	0.5	-0.01
Hyperlipidemia	0.2	0.2	0	0.2	0.2	-0.01
Hypertensive disorder	0.9	0.8	0.01	0.9	0.9	0
Lesion of liver	0.1	0.1	0.01	0.1	0.1	-0.01
Obesity	0.2	0.1	0.01	0.2	0.2	0
Osteoarthritis	0.8	0.8	0.01	0.8	0.8	0
Pneumonia	1.1	0.2	0.1	0.7	0.8	-0.01
Psoriasis	0.4	0.3	0.02	0.4	0.4	0

Table S6.130. *Continued.* Selected baseline characteristics for UK IMRD, for the medium-term risk of neurodevelopmental disorders

Characteristic	Before propensity score matching			After propensity score matching		
	Targets, %	Comparators, %	Standardized difference	Targets, %	Comparators, %	Standardized difference
**Medical history (general)**						
Renal impairment	1.1	0.5	0.07	0.8	0.8	0
Rheumatoid arthritis	0.1	0.1	0.01	0.1	0.1	0
Urinary tract infectious disease	2.1	1	0.09	1.8	1.9	-0.01
**Medical history (cardiovascular disease)**						
Atrial fibrillation	0.6	0.3	0.04	0.4	0.5	-0.01
Cerebrovascular disease	0.4	0.3	0.03	0.3	0.4	-0.01
Coronary arteriosclerosis	0.1	0.1	0	0.1	0.1	0.01
Heart disease	1.7	1.1	0.06	1.3	1.5	-0.02
Heart failure	0.4	0.2	0.04	0.3	0.3	-0.01
Ischemic heart disease	0.4	0.3	0.03	0.4	0.4	-0.01
Peripheral vascular disease	0.1	0.1	0.01	0.1	0.1	0.01
Pulmonary embolism	0.2	0.1	0.03	0.2	0.1	0
Venous thrombosis	0.3	0.2	0.01	0.3	0.3	-0.01
**Medical history (neoplasms)**						
Malignant neoplastic disease	1	0.8	0.02	0.7	0.7	-0.01
Malignant tumor of breast	0.1	0.1	0	0.1	0.1	0
**Medication use**						
Agents acting on the renin-angiotensin system	12.6	12.2	0.01	11.8	12.1	-0.01
Antibacterials for systemic use	38.4	27.7	0.23	37.2	39.5	-0.05
Antidepressants	22.2	15.9	0.16	21.7	23.3	-0.04
Antiepileptics	5.9	4.3	0.07	5.4	5.9	-0.02
Antiinflammatory and antirheumatic products	20.5	16.4	0.11	20	21	-0.02

Table S6.130. *Continued.* Selected baseline characteristics for UK IMRD, for the medium-term risk of neurodevelopmental disorders

Characteristic	Before propensity score matching			After propensity score matching		
	Targets, %	Comparators, %	Standardized difference	Targets, %	Comparators, %	Standardized difference
**Medication use**						
Antineoplastic agents	1	1	0	0.9	1	0
Antipsoriatics	4.5	3	0.08	4.2	4.5	-0.02
Antithrombotic agents	10.1	9.1	0.03	8.3	8.4	0
Beta blocking agents	10	8.9	0.04	9.1	9.4	-0.01
Calcium channel blockers	8.3	8.3	0	7.7	7.6	0
Diuretics	6.9	6.1	0.03	5.6	5.7	0
Drugs for acid-related disorders	25.8	20.7	0.12	24.5	26	-0.03
Drugs for obstructive airway diseases	23.7	19.2	0.11	23.5	24.6	-0.03
Drugs used in diabetes	6.5	4.9	0.07	5.8	6	-0.01
Immunosuppressants	1	0.9	0.01	0.9	1	-0.01
Lipid modifying agents	13.9	14.3	-0.01	12.7	12.8	0
Opioids	17.6	13	0.13	16.9	17.9	-0.03
Psycholeptics	10.3	7.7	0.09	9.2	9.9	-0.02
Psychostimulants, agents used for ADHD and nootropics	0.2	0.3	-0.01	0.2	0.2	-0.01

Table S6.131. Selected baseline characteristics for UK IMRD, for the medium-term risk of any of psychiatric and neuropsychiatric disorders

Characteristic	Before propensity score matching			After propensity score matching		
	Targets, %	Comparators, %	Standardized difference	Targets, %	Comparators, %	Standardized difference
**Age group (years)**						
0-4	1.7	3.9	-0.14	1.8	1.8	0
5-9	2.3	5.2	-0.15	2.5	2.5	0
10-14	3.8	5.2	-0.07	4.1	3.9	0.01
15-19	6	4.9	0.05	6.3	6.4	0
20-24	7.3	5.2	0.09	7.6	8	-0.02
25-29	7.6	6.2	0.06	7.7	7.8	0
35-39	8.1	6.9	0.05	8.4	8.3	0
40-44	7.6	6.5	0.04	7.9	8	0
45-49	8.1	6.8	0.05	8.7	8.5	0.01
50-54	8.9	7.4	0.06	9.4	9.6	-0.01
55-59	8.4	7.3	0.04	9	8.9	0
60-64	6	6.4	-0.01	6.4	6.4	0
65-69	3.6	5.6	-0.1	3.6	3.5	0
70-74	3.2	5.7	-0.12	2.9	2.6	0.02
75-79	2.8	4.1	-0.07	2.1	2	0.01
85-89	2.1	1.7	0.03	1.1	1.1	-0.01
90-94	1.4	0.8	0.06	0.6	0.7	-0.01
95-99	0.6	0.2	0.06	0.2	0.3	0
**Sex**						
Female	56.7	49.5	0.14	56.6	58.6	-0.04

Table S6.131. *Continued.* Selected baseline characteristics for UK IMRD, for the medium-term risk of any of psychiatric and neuropsychiatric disorders

Characteristic	Before propensity score matching			After propensity score matching		
	Targets, %	Comparators, %	Standardized difference	Targets, %	Comparators, %	Standardized difference
**Race**						
race = Asian	0.8	0.6	0.02	0.8	0.8	0
race = Black or African American	0.2	0.3	-0.01	0.2	0.2	-0.01
race = White	4.5	6.7	-0.1	4.5	4.2	0.01
race = Asian Indian	0.9	0.9	0.01	0.8	0.8	0
race = Bangladeshi	0.2	0.1	0.01	0.2	0.1	0.01
race = Chinese	0.1	0.4	-0.06	0.1	0.1	0
race = Pakistani	1.7	0.6	0.11	1.9	2	0
race = African	0.7	0.9	-0.02	0.6	0.6	0
race = European	36.7	36.2	0.01	36.3	37.3	-0.02
race = Middle Eastern or North African	0.1	0.1	0	0.1	0.1	0
**Medical history (general)**						
Acute respiratory disease	4.4	2.6	0.1	4.2	4.6	-0.02
Chronic obstructive lung disease	0.7	0.6	0.02	0.5	0.6	0
Diabetes mellitus	0.7	0.7	0.01	0.6	0.7	-0.01
Gastroesophageal reflux disease	0.3	0.2	0.02	0.3	0.3	0
Gastrointestinal hemorrhage	0.5	0.4	0.02	0.5	0.5	0
Hyperlipidemia	0.2	0.2	0	0.2	0.2	-0.01
Hypertensive disorder	0.9	0.8	0.01	0.9	0.9	0
Lesion of liver	0.1	0.1	0.01	0.1	0.1	-0.01
Obesity	0.2	0.1	0.02	0.2	0.2	0
Osteoarthritis	0.8	0.8	0	0.7	0.8	0
Pneumonia	1.1	0.2	0.1	0.7	0.7	0
Psoriasis	0.4	0.3	0.02	0.3	0.4	0
Renal impairment	1.1	0.5	0.07	0.7	0.7	0

Table S6.131. *Continued.* Selected baseline characteristics for UK IMRD, for the medium-term risk of any of psychiatric and neuropsychiatric disorders

Characteristic	Before propensity score matching			After propensity score matching		
	Targets, %	Comparators, %	Standardized difference	Targets, %	Comparators, %	Standardized difference
**Medical history (general)**						
Rheumatoid arthritis	0.1	0.1	0.01	0.1	0.1	0
Urinary tract infectious disease	2.1	1	0.1	1.6	1.8	-0.01
**Medical history (cardiovascular disease)**						
Atrial fibrillation	0.6	0.4	0.04	0.4	0.5	-0.01
Cerebrovascular disease	0.4	0.3	0.03	0.3	0.3	-0.01
Heart disease	1.7	1.1	0.05	1.2	1.4	-0.01
Heart failure	0.4	0.2	0.04	0.2	0.3	-0.01
Ischemic heart disease	0.4	0.3	0.02	0.4	0.4	-0.01
Peripheral vascular disease	0.1	0.1	0.01	0.1	0.1	0.01
Pulmonary embolism	0.2	0.1	0.03	0.2	0.1	0.01
Venous thrombosis	0.3	0.2	0.02	0.2	0.3	-0.02
**Medical history (neoplasms)**						
Malignant neoplastic disease	1	0.8	0.02	0.6	0.7	-0.01
Malignant tumor of breast	0.1	0.1	0	0.1	0.1	0
**Medication use**						
Agents acting on the renin-angiotensin system	12.6	12.2	0.01	11.7	11.9	-0.01
Antibacterials for systemic use	38.4	27.7	0.23	36.1	38.5	-0.05
Antidepressants	22.2	16	0.16	19.2	20.7	-0.04
Antiepileptics	5.9	4.3	0.08	5	5.5	-0.03
Antiinflammatory and antirheumatic products	20.5	16.5	0.1	19.5	20.3	-0.02
Antineoplastic agents	1	1	0	0.9	1	0
Antipsoriatics	4.5	3	0.08	4	4.3	-0.01

Table S6.131. *Continued.* Selected baseline characteristics for UK IMRD, for the medium-term risk of any of psychiatric and neuropsychiatric disorders

Characteristic	Before propensity score matching			After propensity score matching		
	Targets, %	Comparators, %	Standardized difference	Targets, %	Comparators, %	Standardized difference
**Medication use**						
Antithrombotic agents	10.1	9.2	0.03	7.7	7.9	-0.01
Beta blocking agents	10	8.9	0.04	8.3	8.7	-0.01
Calcium channel blockers	8.3	8.3	0	7.5	7.4	0
Diuretics	6.9	6.1	0.03	5.4	5.4	0
Drugs for acid-related disorders	25.8	20.8	0.12	23.5	25	-0.03
Drugs for obstructive airway diseases	23.7	19.2	0.11	23	24.1	-0.03
Drugs used in diabetes	6.5	4.9	0.07	5.6	5.8	-0.01
Immunosuppressants	1	0.9	0.01	0.9	1	-0.01
Lipid modifying agents	13.9	14.3	-0.01	12.2	12.4	-0.01
Opioids	17.6	12.9	0.13	16.2	17.1	-0.02
Psycholeptics	10.3	7.7	0.09	7.8	8.4	-0.02
Psychostimulants, agents used for ADHD and nootropics	0.2	0.3	-0.01	0.2	0.2	-0.01

Table S6.132. Selected baseline characteristics for UK IMRD, for the long-term risk of depression

Characteristic	Before propensity score matching			After propensity score matching		
	Targets, %	Comparators, %	Standardized difference	Targets, %	Comparators, %	Standardized difference
**Age group (years)**						
0-4	1.7	4	-0.14	1.8	1.9	-0.01
5-9	2.3	5.2	-0.15	2.4	2.4	0
10-14	3.8	5.2	-0.07	3.9	3.6	0.02
15-19	6	4.9	0.05	6.2	6.2	0
20-24	7.3	5.2	0.09	7.4	7.8	-0.01
25-29	7.6	6.2	0.06	7.5	7.6	0
30-34	7.7	6.9	0.03	7.8	7.9	0
35-39	8.1	7	0.04	8.2	8.1	0
40-44	7.6	6.5	0.04	8	7.9	0
45-49	8.1	6.7	0.05	8.8	8.7	0
50-54	8.9	7.4	0.05	9.5	9.7	-0.01
55-59	8.4	7.3	0.04	9.1	9.2	0
60-64	6	6.5	-0.02	6.6	6.6	0
65-69	3.6	5.7	-0.1	3.7	3.7	0
70-74	3.2	5.7	-0.12	3	2.8	0.02
75-79	2.8	4.1	-0.07	2.3	2.1	0.01
80-84	2.5	2.8	-0.02	1.7	1.7	0.01
85-89	2.1	1.8	0.02	1.1	1.2	0
90-94	1.4	0.8	0.06	0.6	0.7	-0.01
95-99	0.6	0.2	0.06	0.2	0.2	0
**Sex**						
Female	56.7	49.3	0.15	56.8	58.8	-0.04

Table S6.132. *Continued.* Selected baseline characteristics for UK IMRD, for the long-term risk of depression

Characteristic	Before propensity score matching			After propensity score matching		
	Targets, %	Comparators, %	Standardized diff	Targets, %	Comparators, %	Standardized diff
**Race**						
race = Asian	0.8	0.6	0.02	0.7	0.6	0.01
race = Black or African American	0.2	0.3	-0.02	0.1	0.2	0
race = White	4.5	6.7	-0.1	4.4	3.9	0.03
race = Asian Indian	0.9	0.9	0	0.6	0.6	0
race = Bangladeshi	0.2	0.1	0.02	0.2	0.1	0.02
race = Pakistani	1.7	0.6	0.11	1.9	2.1	-0.01
race = African	0.7	0.9	-0.02	0.4	0.4	-0.01
race = European	36.7	36.2	0.01	36.1	37.1	-0.02
**Medical history (general)**						
Acute respiratory disease	4.4	2.6	0.1	4.2	4.6	-0.02
Attention deficit hyperactivity disorder	0.1	0.1	0	0.1	0.1	0
Chronic obstructive lung disease	0.7	0.6	0.02	0.6	0.6	-0.01
Dementia	0.4	0.2	0.04	0.2	0.3	-0.01
Diabetes mellitus	0.7	0.6	0.01	0.7	0.8	-0.01
Gastroesophageal reflux disease	0.3	0.2	0.03	0.3	0.4	-0.01
Gastrointestinal hemorrhage	0.5	0.4	0.02	0.5	0.5	-0.01
Hyperlipidemia	0.2	0.2	0	0.2	0.2	0
Hypertensive disorder	0.9	0.8	0.01	0.9	1	-0.01
Lesion of liver	0.1	0.1	0.01	0.1	0.1	-0.01
Obesity	0.2	0.1	0.02	0.2	0.2	0
Osteoarthritis	0.8	0.7	0.01	0.8	0.8	0
Pneumonia	1.1	0.2	0.1	0.7	0.7	0
Psoriasis	0.4	0.3	0.01	0.3	0.3	0
Renal impairment	1.1	0.5	0.07	0.7	0.8	0
Rheumatoid arthritis	0.1	0.1	0.01	0.1	0.1	0
Urinary tract infectious disease	2.1	1	0.09	1.7	1.9	-0.01

Table S6.132. *Continued.* Selected baseline characteristics for UK IMRD, for the long-term risk of depression

Characteristic	Before propensity score matching			After propensity score matching		
	Targets, %	Comparators, %	Standardized difference	Targets, %	Comparators, %	Standardized difference
**Medical history (cardiovascular disease)**						
Atrial fibrillation	0.6	0.4	0.04	0.4	0.5	-0.01
Cerebrovascular disease	0.4	0.3	0.03	0.3	0.4	-0.01
Coronary arteriosclerosis	0.1	0.1	0	0.1	0.1	0.01
Heart disease	1.7	1.1	0.05	1.3	1.5	-0.01
Heart failure	0.4	0.2	0.04	0.2	0.3	0
Ischemic heart disease	0.4	0.3	0.02	0.4	0.4	-0.01
Pulmonary embolism	0.2	0.1	0.03	0.2	0.1	0.01
Venous thrombosis	0.3	0.2	0.01	0.3	0.3	-0.01
**Medical history (neoplasms)**						
Malignant neoplastic disease	1	0.8	0.02	0.6	0.7	-0.01
Malignant tumor of breast	0.1	0.1	0	0.1	0.1	-0.01
**Medication use**						
Agents acting on the renin-angiotensin system	12.6	12.2	0.01	12.1	12.5	-0.01
Antibacterials for systemic use	38.4	27.8	0.23	37.1	39.7	-0.05
Antidepressants	22.2	15.9	0.16	20.8	22.5	-0.04
Antiepileptics	5.9	4.3	0.08	5.3	5.7	-0.02
Antiinflammatory and antirheumatic products	20.5	16.4	0.11	20.4	21.4	-0.02
Antipsoriatics	4.5	3	0.08	4.2	4.5	-0.01
Antithrombotic agents	10.1	9.2	0.03	8.2	8.4	-0.01
Beta blocking agents	10	8.8	0.04	8.9	9.5	-0.02
Calcium channel blockers	8.3	8.4	0	7.7	7.9	-0.01
Diuretics	6.9	6.1	0.03	5.6	5.8	-0.01
Drugs for acid-related disorders	25.8	20.7	0.12	24.8	26.8	-0.05

Table S6.132. *Continued.* Selected baseline characteristics for UK IMRD, for the long-term risk of depression

Characteristic	Before propensity score matching			After propensity score matching		
	**Targets, %**	**Comparators, %**	**Standardized difference**	**Targets, %**	**Comparators, %**	**Standardized difference**
**Medication use**						
Drugs for obstructive airway diseases	23.7	19.1	0.11	23.7	24.9	-0.03
Drugs used in diabetes	6.5	4.9	0.07	5.7	6.1	-0.02
Immunosuppressants	1	0.9	0.01	0.9	1	0
Lipid modifying agents	13.9	14.3	-0.01	12.7	12.9	-0.01
Opioids	17.6	12.9	0.13	17.2	18.3	-0.03
Psycholeptics	10.3	7.7	0.09	9	9.7	-0.03
Psychostimulants, agents used for ADHD and nootropics	0.2	0.3	-0.01	0.2	0.3	-0.01

Table S6.133. Selected baseline characteristics for UK IMRD, for the long-term risk of anxiety disorders

Characteristic	Before propensity score matching			After propensity score matching		
	Targets, %	Comparators, %	Standardized difference	Targets, %	Comparators, %	Standardized difference
**Age group (years)**						
0-4	1.7	3.9	-0.13	1.8	1.9	-0.01
5-9	2.3	5.3	-0.16	2.4	2.4	0.01
10-14	3.8	5.2	-0.07	3.9	3.6	0.02
15-19	6	4.9	0.05	6.2	6.2	0
20-24	7.3	5.2	0.09	7.4	7.8	-0.02
25-29	7.6	6.2	0.06	7.5	7.6	0
30-34	7.7	7	0.03	7.8	7.9	0
35-39	8.1	6.9	0.05	8.2	8.1	0
40-44	7.6	6.4	0.05	7.9	7.8	0
45-49	8.1	6.7	0.06	8.8	8.7	0
50-54	8.9	7.5	0.05	9.5	9.7	-0.01
55-59	8.4	7.4	0.04	9.1	9.2	0
65-69	3.6	5.6	-0.1	3.7	3.7	0
70-74	3.2	5.7	-0.12	3	2.8	0.01
75-79	2.8	4.1	-0.07	2.3	2.1	0.01
80-84	2.5	2.9	-0.02	1.7	1.7	0
85-89	2.1	1.7	0.03	1.1	1.2	0
90-94	1.4	0.7	0.06	0.6	0.7	-0.01
95-99	0.6	0.2	0.06	0.2	0.2	0
**Sex**						
Female	56.7	49.3	0.15	56.6	58.7	-0.04

Table S6.133. *Continued.* Selected baseline characteristics for UK IMRD, for the long-term risk of anxiety disorders

Characteristic	Before propensity score matching			After propensity score matching		
	Targets, %	Comparators, %	Standardized difference	Targets, %	Comparators, %	Standardized difference
**Race**						
race = Asian	0.8	0.7	0.01	0.7	0.6	0.01
race = Black or African American	0.2	0.4	-0.02	0.1	0.2	-0.01
race = White	4.5	6.6	-0.09	4.4	3.9	0.02
race = Asian Indian	0.9	0.9	0.01	0.6	0.6	0
race = Bangladeshi	0.2	0.1	0.01	0.2	0.1	0.02
race = Pakistani	1.7	0.6	0.1	2	2.1	-0.01
race = African	0.7	0.9	-0.02	0.4	0.4	0
race = European	36.7	36.2	0.01	36.2	37.1	-0.02
**Medical history (general)**						
Acute respiratory disease	4.4	2.6	0.1	4.2	4.6	-0.02
Chronic obstructive lung disease	0.7	0.6	0.02	0.5	0.7	-0.01
Dementia	0.4	0.2	0.04	0.2	0.3	-0.01
Depressive disorder	1.4	1.1	0.04	0.7	0.8	-0.01
Diabetes mellitus	0.7	0.6	0.02	0.7	0.8	-0.01
Gastroesophageal reflux disease	0.3	0.2	0.03	0.3	0.4	-0.01
Gastrointestinal hemorrhage	0.5	0.4	0.02	0.5	0.5	-0.01
Hyperlipidemia	0.2	0.2	0	0.2	0.2	0
Hypertensive disorder	0.9	0.8	0.01	0.9	1	-0.01
Lesion of liver	0.1	0.1	0.01	0.1	0.1	-0.01
Obesity	0.2	0.1	0.02	0.2	0.2	0
Pneumonia	1.1	0.2	0.11	0.7	0.7	0
Psoriasis	0.4	0.3	0.01	0.3	0.3	0
Renal impairment	1.1	0.5	0.07	0.8	0.8	0
Urinary tract infectious disease	2.1	1	0.09	1.7	1.9	-0.01

Table S6.133. *Continued.* Selected baseline characteristics for UK IMRD, for the long-term risk of anxiety disorders

Characteristic	Before propensity score matching			After propensity score matching		
	Targets, %	Comparators, %	Standardized difference	Targets, %	Comparators, %	Standardized difference
**Medical history (cardiovascular disease)**						
Medical history: Cardiovascular disease						
Atrial fibrillation	0.6	0.3	0.04	0.4	0.5	-0.01
Cerebrovascular disease	0.4	0.3	0.03	0.3	0.4	-0.01
Coronary arteriosclerosis	0.1	0.1	0	0.1	0.1	0.01
Heart disease	1.7	1	0.06	1.3	1.5	-0.02
Heart failure	0.4	0.2	0.04	0.2	0.3	-0.01
Ischemic heart disease	0.4	0.3	0.02	0.4	0.4	-0.01
Pulmonary embolism	0.2	0.1	0.03	0.2	0.1	0.01
Venous thrombosis	0.3	0.2	0.01	0.3	0.3	-0.01
**Medical history (neoplasms)**						
Malignant neoplastic disease	1	0.7	0.03	0.6	0.7	-0.01
Malignant tumor of breast	0.1	0.1	0	0.1	0.1	-0.01
**Medication use**						
Agents acting on the renin-angiotensin system	12.6	12.3	0.01	12	12.5	-0.01
Antibacterials for systemic use	38.4	27.7	0.23	36.9	39.6	-0.06
Antidepressants	22.2	15.9	0.16	20.8	22.5	-0.04
Antiepileptics	5.9	4.3	0.07	5.2	5.7	-0.02
Antiinflammatory and antirheumatic products	20.5	16.5	0.1	20.3	21.4	-0.03
Antineoplastic agents	1	1	0	0.9	0.9	0
Antipsoriatics	4.5	3	0.08	4.2	4.5	-0.01
Antithrombotic agents	10.1	9.2	0.03	8.2	8.4	-0.01
Beta blocking agents	10	8.9	0.04	8.7	9.3	-0.02
Calcium channel blockers	8.3	8.3	0	7.7	7.9	-0.01

Table S6.133. *Continued.* Selected baseline characteristics for UK IMRD, for the long-term risk of anxiety disorders

Characteristic	Before propensity score matching			After propensity score matching		
	**Targets, %**	**Comparators, %**	**Standardized difference**	**Targets, %**	**Comparators, %**	**Standardized difference**
**Medication use**						
Diuretics	6.9	6.1	0.03	5.6	5.9	-0.01
Drugs for acid-related disorders	25.8	20.7	0.12	24.6	26.8	-0.05
Drugs for obstructive airway diseases	23.7	19.1	0.11	23.6	24.9	-0.03
Drugs used in diabetes	6.5	4.9	0.07	5.7	6.1	-0.02
Immunosuppressants	1	0.9	0.01	0.9	1	0
Lipid modifying agents	13.9	14.4	-0.01	12.7	13	-0.01
Opioids	17.6	12.9	0.13	17	18.3	-0.03
Psycholeptics	10.3	7.7	0.09	8.8	9.5	-0.03
Psychostimulants, agents used for ADHD and nootropics	0.2	0.3	-0.01	0.2	0.2	-0.01

Table S6.134. Selected baseline characteristics for UK IMRD, for the long-term risk of alcohol misuse or dependence

Characteristic	Before propensity score matching			After propensity score matching		
	Targets, %	Comparators, %	Standardized difference	Targets, %	Comparators, %	Standardized difference
**Age group (years)**						
0-4	1.7	3.9	-0.14	1.7	1.8	-0.01
5-9	2.3	5.3	-0.16	2.4	2.3	0.01
10-14	3.8	5.2	-0.07	3.9	3.5	0.02
15-19	6	4.9	0.04	6.2	6.2	0
20-24	7.3	5.2	0.09	7.6	8	-0.02
25-29	7.6	6.2	0.06	7.6	7.7	0
30-34	7.7	6.9	0.03	7.9	8	0
35-39	8.1	6.9	0.05	8.3	8.2	0
40-44	7.6	6.5	0.05	8	7.8	0
45-49	8.1	6.8	0.05	8.8	8.8	0
50-54	8.9	7.4	0.05	9.4	9.7	-0.01
55-59	8.4	7.4	0.04	9	9.1	0
60-64	6	6.4	-0.01	6.6	6.5	0
65-69	3.6	5.7	-0.1	3.7	3.6	0
70-74	3.2	5.7	-0.12	3	2.7	0.01
75-79	2.8	4.1	-0.07	2.3	2.1	0.01
80-84	2.5	2.9	-0.02	1.7	1.6	0.01
85-89	2.1	1.7	0.03	1.1	1.2	0
90-94	1.4	0.7	0.06	0.6	0.7	-0.01
95-99	0.6	0.2	0.06	0.2	0.2	0
**Sex**						
Female	56.7	49.4	0.15	57.1	59.1	-0.04

Table S6.134. *Continued.* Selected baseline characteristics for UK IMRD, for the long-term risk of alcohol misuse or dependence

Characteristic	Before propensity score matching			After propensity score matching		
	Targets, %	Comparators, %	Standardized difference	Targets, %	Comparators, %	Standardized difference
**Race**						
race = Asian	0.8	0.7	0.01	0.7	0.6	0.01
race = Black or African American	0.2	0.3	-0.01	0.1	0.2	-0.01
race = White	4.5	6.7	-0.1	4.4	3.9	0.02
race = Asian Indian	0.9	0.9	0.01	0.6	0.6	0
race = Bangladeshi	0.2	0.1	0.01	0.2	0.1	0.02
race = Pakistani	1.7	0.6	0.11	1.9	2.1	-0.01
race = African	0.7	0.8	-0.02	0.4	0.4	-0.01
race = European	36.7	36.3	0.01	36.2	37.2	-0.02
**Medical history (general)**						
Acute respiratory disease	4.4	2.6	0.1	4.3	4.7	-0.02
Chronic obstructive lung disease	0.7	0.6	0.02	0.6	0.7	-0.01
Dementia	0.4	0.2	0.04	0.2	0.3	-0.01
Depressive disorder	1.4	1.1	0.03	1.4	1.5	-0.01
Diabetes mellitus	0.7	0.6	0.01	0.7	0.8	-0.01
Gastroesophageal reflux disease	0.3	0.2	0.03	0.4	0.4	0
Gastrointestinal hemorrhage	0.5	0.4	0.02	0.5	0.5	-0.01
Hyperlipidemia	0.2	0.2	0	0.2	0.2	0
Hypertensive disorder	0.9	0.8	0.01	0.9	1	-0.01
Lesion of liver	0.1	0.1	0.01	0.1	0.1	-0.01
Obesity	0.2	0.1	0.02	0.2	0.2	0
Osteoarthritis	0.8	0.8	0.01	0.8	0.8	0
Pneumonia	1.1	0.2	0.11	0.7	0.7	0

Table S6.134. *Continued.* Selected baseline characteristics for UK IMRD, for the long-term risk of alcohol misuse or dependence

Characteristic	Before propensity score matching			After propensity score matching		
	Targets, %	Comparators, %	Standardized difference	Targets, %	Comparators, %	Standardized difference
**Medical history (general)**						
Psoriasis	0.4	0.3	0.01	0.3	0.3	0
Renal impairment	1.1	0.5	0.07	0.8	0.8	0
Rheumatoid arthritis	0.1	0.1	0.01	0.1	0.1	0
Urinary tract infectious disease	2.1	1	0.09	1.8	2	-0.02
**Medical history (cardiovascular disease)**						
Atrial fibrillation	0.6	0.3	0.04	0.4	0.5	-0.01
Cerebrovascular disease	0.4	0.3	0.03	0.3	0.4	-0.01
Coronary arteriosclerosis	0.1	0.1	0	0.1	0.1	0.01
Heart disease	1.7	1.1	0.05	1.3	1.5	-0.02
Heart failure	0.4	0.2	0.04	0.2	0.3	0
Ischemic heart disease	0.4	0.3	0.02	0.4	0.4	-0.01
Pulmonary embolism	0.2	0.1	0.03	0.2	0.1	0.01
Venous thrombosis	0.3	0.2	0.02	0.3	0.3	-0.01
**Medical history (neoplasms)**						
Malignant neoplastic disease	1	0.8	0.02	0.6	0.7	-0.01
Malignant tumor of breast	0.1	0.1	0	0.1	0.1	-0.01
**Medication use**						
Agents acting on the renin-angiotensin system	12.6	12.3	0.01	12	12.4	-0.01
Antibacterials for systemic use	38.4	27.7	0.23	37.3	39.9	-0.05
Antidepressants	22.2	16	0.16	22	23.8	-0.04
Antiepileptics	5.9	4.3	0.08	5.3	5.8	-0.02
Antiinflammatory and antirheumatic products	20.5	16.4	0.11	20.5	21.5	-0.03

Table S6.134. *Continued.* Selected baseline characteristics for UK IMRD, for the long-term risk of alcohol misuse or dependence

Characteristic	Before propensity score matching			After propensity score matching		
	Targets, %	Comparators, %	Standardized difference	Targets, %	Comparators, %	Standardized difference
**Medication use**						
Antipsoriatics	4.5	3	0.08	4.2	4.6	-0.02
Antithrombotic agents	10.1	9.1	0.03	8.1	8.4	-0.01
Beta blocking agents	10	8.8	0.04	9.1	9.6	-0.02
Calcium channel blockers	8.3	8.4	0	7.7	7.8	-0.01
Diuretics	6.9	6.2	0.03	5.6	5.8	-0.01
Drugs for acid-related disorders	25.8	20.6	0.12	24.9	26.9	-0.05
Drugs for obstructive airway diseases	23.7	19.1	0.11	23.9	25	-0.03
Drugs used in diabetes	6.5	4.9	0.07	5.7	6.1	-0.02
Immunosuppressants	1	0.9	0.01	0.9	0.9	0
Lipid modifying agents	13.9	14.3	-0.01	12.6	12.8	-0.01
Opioids	17.6	13	0.13	17.3	18.5	-0.03
Psycholeptics	10.3	7.7	0.09	9.2	10	-0.03
Psychostimulants, agents used for ADHD and nootropics	0.2	0.3	-0.01	0.2	0.3	-0.01

Table S6.135. Selected baseline characteristics for UK IMRD, for the long-term risk of substance misuse or dependence

Characteristic	Before propensity score matching			After propensity score matching		
	**Targets, %**	**Comparators, %**	**Standardized difference**	**Targets, %**	**Comparators, %**	**Standardized difference**
**Age group (years)**						
0-4	1.7	3.9	-0.14	1.7	1.8	-0.01
5-9	2.3	5.3	-0.16	2.4	2.3	0.01
10-14	3.8	5.3	-0.07	3.9	3.5	0.02
15-19	6	4.9	0.04	6.2	6.2	0
20-24	7.3	5.2	0.09	7.5	8	-0.02
25-29	7.6	6.2	0.06	7.7	7.7	0
30-34	7.7	6.8	0.04	7.9	8	0
35-39	8.1	6.9	0.05	8.3	8.2	0
40-44	7.6	6.4	0.05	8	7.8	0.01
45-49	8.1	6.7	0.05	8.8	8.8	0
50-54	8.9	7.4	0.06	9.4	9.7	-0.01
55-59	8.4	7.4	0.04	9	9.2	0
60-64	6	6.5	-0.02	6.6	6.5	0
65-69	3.6	5.7	-0.1	3.7	3.6	0
70-74	3.2	5.7	-0.12	3	2.7	0.01
75-79	2.8	4.1	-0.07	2.3	2.1	0.01
80-84	2.5	2.9	-0.02	1.7	1.6	0.01
85-89	2.1	1.7	0.03	1.1	1.2	-0.01
90-94	1.4	0.8	0.06	0.6	0.7	-0.01
95-99	0.6	0.2	0.06	0.2	0.2	0
**Sex**						
Female	56.7	49.7	0.14	57.2	59.2	-0.04

Table S6.135. *Continued.* Selected baseline characteristics for UK IMRD, for the long-term risk of substance misuse or dependence

Characteristic	Before propensity score matching			After propensity score matching		
	Targets, %	Comparators, %	Standardized difference	Targets, %	Comparators, %	Standardized difference
**Race**						
race = Asian	0.8	0.6	0.02	0.7	0.6	0.01
race = Black or African American	0.2	0.3	-0.02	0.1	0.2	-0.01
race = White	4.5	6.6	-0.09	4.4	3.9	0.02
race = Asian Indian	0.9	0.9	0.01	0.6	0.6	0
race = Bangladeshi	0.2	0.1	0.02	0.2	0.1	0.02
race = Pakistani	1.7	0.6	0.11	1.9	2.1	-0.01
race = African	0.7	0.9	-0.02	0.4	0.4	-0.01
race = European	36.7	36.3	0.01	36.2	37.2	-0.02
**Medical history (general)**						
Acute respiratory disease	4.4	2.7	0.09	4.3	4.7	-0.02
Attention deficit hyperactivity disorder	0.1	0.1	0	0.1	0.1	0
Chronic obstructive lung disease	0.7	0.6	0.02	0.6	0.7	-0.01
Dementia	0.4	0.2	0.04	0.2	0.3	-0.01
Depressive disorder	1.4	1	0.04	1.4	1.5	-0.01
Diabetes mellitus	0.7	0.6	0.01	0.7	0.8	-0.01
Gastroesophageal reflux disease	0.3	0.2	0.03	0.4	0.4	0
Gastrointestinal hemorrhage	0.5	0.4	0.02	0.5	0.5	-0.01
Hypertensive disorder	0.9	0.8	0.01	0.9	1	-0.01
Lesion of liver	0.1	0.1	0.01	0.1	0.1	-0.01
Obesity	0.2	0.1	0.02	0.2	0.2	0
Osteoarthritis	0.8	0.7	0.01	0.8	0.8	0
Pneumonia	1.1	0.2	0.1	0.7	0.7	0

Table S6.135. *Continued.* Selected baseline characteristics for UK IMRD, for the long-term risk of substance misuse or dependence

Characteristic	Before propensity score matching			After propensity score matching		
	Targets, %	Comparators, %	Standardized difference	Targets, %	Comparators, %	Standardized difference
**Medical history (general)**						
Psoriasis	0.4	0.3	0.01	0.3	0.3	0
Renal impairment	1.1	0.5	0.07	0.7	0.8	0
Rheumatoid arthritis	0.1	0.1	0.01	0.1	0.1	0
Urinary tract infectious disease	2.1	1	0.09	1.8	2	-0.02
**Medical history (cardiovascular disease)**						
Atrial fibrillation	0.6	0.3	0.04	0.4	0.5	-0.01
Cerebrovascular disease	0.4	0.3	0.03	0.3	0.4	-0.01
Coronary arteriosclerosis	0.1	0.1	0.01	0.1	0.1	0.01
Heart disease	1.7	1.1	0.06	1.3	1.5	-0.02
Heart failure	0.4	0.2	0.04	0.2	0.3	0
Ischemic heart disease	0.4	0.3	0.02	0.4	0.4	-0.01
Pulmonary embolism	0.2	0.1	0.04	0.2	0.1	0.01
Venous thrombosis	0.3	0.2	0.02	0.3	0.3	-0.01
**Medical history (neoplasms)**						
Malignant neoplastic disease	1	0.8	0.02	0.6	0.7	-0.01
Malignant tumor of breast	0.1	0.1	0	0.1	0.1	-0.01
**Medication use**						
Agents acting on the renin-angiotensin system	12.6	12.3	0.01	11.9	12.4	-0.01
Antibacterials for systemic use	38.4	27.8	0.23	37.3	39.9	-0.05
Antidepressants	22.2	16	0.16	22	23.7	-0.04
Antiepileptics	5.9	4.4	0.07	5.3	5.8	-0.02
Antiinflammatory and antirheumatic products	20.5	16.4	0.11	20.5	21.5	-0.03

Table S6.135. *Continued.* Selected baseline characteristics for UK IMRD, for the long-term risk of substance misuse or dependence

Characteristic	Before propensity score matching			After propensity score matching		
	Targets, %	Comparators, %	Standardized difference	Targets, %	Comparators, %	Standardized difference
**Medication use**						
Antipsoriatics	4.5	3	0.08	4.2	4.6	-0.02
Antithrombotic agents	10.1	9.2	0.03	8.1	8.4	-0.01
Beta blocking agents	10	8.9	0.04	9.1	9.6	-0.02
Calcium channel blockers	8.3	8.4	0	7.7	7.8	-0.01
Diuretics	6.9	6.1	0.03	5.6	5.8	-0.01
Drugs for acid-related disorders	25.8	20.7	0.12	24.9	26.9	-0.05
Drugs for obstructive airway diseases	23.7	19.2	0.11	23.9	25	-0.03
Drugs used in diabetes	6.5	4.9	0.07	5.7	6	-0.02
Immunosuppressants	1	0.9	0.01	0.9	0.9	0
Lipid modifying agents	13.9	14.3	-0.01	12.6	12.8	-0.01
Opioids	17.6	12.9	0.13	17.2	18.5	-0.03
Psycholeptics	10.3	7.7	0.09	9.2	10	-0.03
Psychostimulants, agents used for ADHD and nootropics	0.2	0.3	-0.01	0.2	0.3	-0.01

Table S6.136. Selected baseline characteristics for UK IMRD, for the long-term risk of bipolar disorders

Characteristic	Before propensity score matching			After propensity score matching		
	Targets, %	Comparators, %	Standardized difference	Targets, %	Comparators, %	Standardized difference
**Age group (years)**						
0-4	1.7	3.9	-0.14	1.7	1.8	-0.01
5-9	2.3	5.3	-0.16	2.4	2.3	0.01
10-14	3.8	5.2	-0.07	3.8	3.5	0.02
15-19	6	5	0.04	6.1	6.2	0
20-24	7.3	5.2	0.09	7.5	8	-0.02
25-29	7.6	6.1	0.06	7.6	7.7	0
30-34	7.7	6.9	0.03	7.9	8	0
35-39	8.1	6.9	0.05	8.3	8.2	0
40-44	7.6	6.5	0.04	8	7.9	0
45-49	8.1	6.8	0.05	8.8	8.8	0
50-54	8.9	7.4	0.05	9.4	9.7	-0.01
55-59	8.4	7.4	0.04	9	9.1	0
60-64	6	6.4	-0.02	6.6	6.5	0
65-69	3.6	5.7	-0.1	3.7	3.6	0
70-74	3.2	5.7	-0.12	3	2.7	0.01
75-79	2.8	4.1	-0.07	2.3	2.1	0.01
80-84	2.5	2.9	-0.02	1.7	1.6	0.01
85-89	2.1	1.7	0.03	1.1	1.2	0
90-94	1.4	0.8	0.06	0.6	0.7	-0.01
95-99	0.6	0.2	0.06	0.2	0.2	0
**Sex**						
Female	56.7	49.3	0.15	57.1	59.1	-0.04

Table S6.136. *Continued.* Selected baseline characteristics for UK IMRD, for the long-term risk of bipolar disorders

Characteristic	Before propensity score matching			After propensity score matching		
	Targets, %	Comparators, %	Standardized difference	Targets, %	Comparators, %	Standardized difference
**Race**						
race = Asian	0.8	0.6	0.02	0.7	0.6	0.01
race = Black or African American	0.2	0.3	-0.02	0.1	0.2	-0.01
race = White	4.5	6.8	-0.1	4.4	3.9	0.03
race = Asian Indian	0.9	0.9	0	0.6	0.6	0
race = Bangladeshi	0.2	0.1	0.02	0.2	0.1	0.02
race = Pakistani	1.7	0.6	0.11	1.9	2.1	-0.01
race = African	0.7	0.9	-0.02	0.4	0.4	-0.01
race = European	36.7	36.1	0.01	36.2	37.2	-0.02
**Medical history (general)**						
Acute respiratory disease	4.4	2.6	0.1	4.3	4.7	-0.02
Attention deficit hyperactivity disorder	0.1	0.1	0	0.1	0.1	0
Chronic obstructive lung disease	0.7	0.6	0.02	0.6	0.7	-0.01
Dementia	0.4	0.2	0.04	0.2	0.3	-0.01
Depressive disorder	1.4	1	0.04	1.4	1.5	-0.01
Diabetes mellitus	0.7	0.6	0.01	0.7	0.8	-0.01
Gastroesophageal reflux disease	0.3	0.2	0.02	0.4	0.4	0
Gastrointestinal hemorrhage	0.5	0.4	0.02	0.5	0.5	-0.01
Hyperlipidemia	0.2	0.2	0	0.2	0.2	0
Hypertensive disorder	0.9	0.8	0.01	0.9	1	-0.01
Lesion of liver	0.1	0.1	0.01	0.1	0.1	-0.01
Obesity	0.2	0.1	0.02	0.2	0.2	0
Osteoarthritis	0.8	0.7	0.01	0.8	0.8	0

Table S6.136. *Continued.* Selected baseline characteristics for UK IMRD, for the long-term risk of bipolar disorders

Characteristic	Before propensity score matching			After propensity score matching		
	**Targets, %**	**Comparators, %**	**Standardized difference**	**Targets, %**	**Comparators, %**	**Standardized difference**
**Medical history (general)**						
Pneumonia	1.1	0.2	0.1	0.7	0.7	0
Psoriasis	0.4	0.3	0.02	0.3	0.3	0
Renal impairment	1.1	0.5	0.07	0.8	0.8	0
Rheumatoid arthritis	0.1	0.1	0.01	0.1	0.1	0
Urinary tract infectious disease	2.1	1	0.09	1.8	2	-0.01
**Medical history (cardiovascular disease)**						
Atrial fibrillation	0.6	0.3	0.04	0.4	0.5	-0.01
Cerebrovascular disease	0.4	0.3	0.03	0.3	0.4	-0.01
Coronary arteriosclerosis	0.1	0.1	0	0.1	0.1	0.01
Heart disease	1.7	1.1	0.06	1.3	1.5	-0.02
Heart failure	0.4	0.1	0.05	0.2	0.2	0
Ischemic heart disease	0.4	0.3	0.02	0.4	0.4	-0.01
Pulmonary embolism	0.2	0.1	0.03	0.2	0.1	0.01
Venous thrombosis	0.3	0.2	0.02	0.3	0.3	-0.01
**Medical history (neoplasms)**						
Malignant neoplastic disease	1	0.8	0.02	0.6	0.7	-0.01
Malignant tumor of breast	0.1	0.1	0	0.1	0.1	-0.01
**Medication use**						
Agents acting on the renin-angiotensin system	12.6	12.2	0.01	12	12.4	-0.01
Antibacterials for systemic use	38.4	27.6	0.23	37.3	39.9	-0.05
Antidepressants	22.2	15.8	0.16	22.1	23.9	-0.04
Antiepileptics	5.9	4.3	0.07	5.3	5.8	-0.02

Table S6.136. *Continued.* Selected baseline characteristics for UK IMRD, for the long-term risk of bipolar disorders

**Characteristic**	**Before propensity score matching**			**After propensity score matching**		
	**Targets, %**	**Comparators, %**	**Standardized difference**	**Targets, %**	**Comparators, %**	**Standardized difference**
**Medication use**						
Antiinflammatory and antirheumatic products	20.5	16.4	0.11	20.5	21.5	-0.03
Antineoplastic agents	1	1	0	0.9	0.9	0
Antipsoriatics	4.5	2.9	0.08	4.2	4.5	-0.02
Antithrombotic agents	10.1	9.2	0.03	8.2	8.4	-0.01
Beta blocking agents	10	8.9	0.04	9.1	9.7	-0.02
Calcium channel blockers	8.3	8.2	0	7.7	7.8	-0.01
Diuretics	6.9	6	0.04	5.6	5.8	-0.01
Drugs for acid-related disorders	25.8	20.6	0.12	25	27	-0.05
Drugs for obstructive airway diseases	23.7	19.1	0.11	23.9	25	-0.03
Drugs used in diabetes	6.5	4.8	0.07	5.7	6.1	-0.02
Immunosuppressants	1	0.9	0.01	0.9	1	0
Lipid modifying agents	13.9	14.3	-0.01	12.6	12.8	-0.01
Opioids	17.6	13	0.13	17.3	18.6	-0.03
Psycholeptics	10.3	7.7	0.09	9.3	10.1	-0.03
Psychostimulants, agents used for ADHD and nootropics	0.2	0.3	-0.01	0.2	0.3	-0.01

Table S6.137. Selected baseline characteristics for UK IMRD, for the long-term risk of psychoses

Characteristic	Before propensity score matching			After propensity score matching		
	Targets, %	Comparators, %	Standardized difference	Targets, %	Comparators, %	Standardized difference
**Age group (years)**						
0-4	1.7	3.9	-0.14	1.7	1.8	-0.01
5-9	2.3	5.2	-0.15	2.4	2.3	0.01
10-14	3.8	5.3	-0.07	3.8	3.5	0.02
15-19	6	5	0.04	6.1	6.2	0
20-24	7.3	5.2	0.09	7.5	8	-0.02
25-29	7.6	6.2	0.06	7.6	7.7	0
30-34	7.7	7	0.03	7.9	8	0
35-39	8.1	7	0.04	8.3	8.2	0
40-44	7.6	6.5	0.04	8	7.9	0
45-49	8.1	6.9	0.05	8.8	8.8	0
50-54	8.9	7.4	0.06	9.4	9.7	-0.01
55-59	8.4	7.4	0.04	9	9.1	0
60-64	6	6.5	-0.02	6.6	6.5	0
65-69	3.6	5.6	-0.1	3.7	3.6	0
70-74	3.2	5.7	-0.12	3	2.7	0.01
75-79	2.8	4	-0.07	2.3	2.1	0.01
80-84	2.5	2.9	-0.02	1.7	1.6	0.01
85-89	2.1	1.7	0.03	1.1	1.2	0
90-94	1.4	0.7	0.07	0.6	0.7	-0.01
95-99	0.6	0.2	0.06	0.2	0.2	0
**Sex**						
Female	56.7	49.4	0.14	57.1	59.1	-0.04

Table S6.137. *Continued.* Selected baseline characteristics for UK IMRD, for the long-term risk of psychoses

Characteristic	Before propensity score matching			After propensity score matching		
	Targets, %	Comparators, %	Standardized difference	Targets, %	Comparators, %	Standardized difference
**Race**						
race = Asian	0.8	0.7	0.01	0.7	0.6	0.01
race = Black or African American	0.2	0.3	-0.02	0.1	0.2	-0.01
race = White	4.5	6.7	-0.1	4.4	3.9	0.03
race = Asian Indian	0.9	0.9	0.01	0.6	0.6	0
race = Bangladeshi	0.2	0.1	0.01	0.2	0.1	0.02
race = Pakistani	1.7	0.6	0.1	1.9	2.1	-0.01
race = African	0.7	0.8	-0.02	0.4	0.4	0
race = European	36.7	36.1	0.01	36.2	37.2	-0.02
**Medical history (general)**						
Acute respiratory disease	4.4	2.7	0.09	4.3	4.7	-0.02
Attention deficit hyperactivity disorder	0.1	0.1	0	0.1	0.1	0
Chronic obstructive lung disease	0.7	0.6	0.02	0.6	0.7	-0.01
Dementia	0.4	0.2	0.04	0.2	0.3	-0.01
Depressive disorder	1.4	1	0.04	1.4	1.5	-0.01
Diabetes mellitus	0.7	0.6	0.01	0.7	0.8	-0.01
Gastroesophageal reflux disease	0.3	0.2	0.02	0.4	0.4	-0.01
Gastrointestinal hemorrhage	0.5	0.4	0.02	0.5	0.5	-0.01
Hyperlipidemia	0.2	0.2	0	0.2	0.2	0
Hypertensive disorder	0.9	0.8	0.01	0.9	1	-0.01
Lesion of liver	0.1	0.1	0.01	0.1	0.1	-0.01
Obesity	0.2	0.1	0.02	0.2	0.2	0
Osteoarthritis	0.8	0.8	0.01	0.8	0.8	0

Table S6.137. *Continued.* Selected baseline characteristics for UK IMRD, for the long-term risk of psychoses

Characteristic	Before propensity score matching			After propensity score matching		
	Targets, %	Comparators, %	Standardized difference	Targets, %	Comparators, %	Standardized difference
**Medical history (general)**						
Pneumonia	1.1	0.2	0.11	0.7	0.7	0
Renal impairment	1.1	0.5	0.07	0.7	0.8	0
Rheumatoid arthritis	0.1	0.1	0.01	0.1	0.1	0
Urinary tract infectious disease	2.1	1	0.09	1.8	2	-0.02
**Medical history (cardiovascular disease)**						
Atrial fibrillation	0.6	0.3	0.04	0.4	0.5	-0.01
Cerebrovascular disease	0.4	0.2	0.03	0.3	0.4	-0.01
Coronary arteriosclerosis	0.1	0.1	0.01	0.1	0.1	0.01
Heart disease	1.7	1.1	0.06	1.3	1.5	-0.02
Heart failure	0.4	0.1	0.05	0.2	0.2	0
Ischemic heart disease	0.4	0.3	0.02	0.4	0.4	-0.01
Pulmonary embolism	0.2	0.1	0.04	0.2	0.1	0.01
Venous thrombosis	0.3	0.2	0.02	0.3	0.3	-0.01
**Medical history (neoplasms)**						
Malignant neoplastic disease	1	0.8	0.02	0.6	0.7	-0.01
Malignant tumor of breast	0.1	0.1	0	0.1	0.1	-0.01
**Medication use**						
Agents acting on the renin-angiotensin system	12.6	12.2	0.01	12	12.4	-0.01
Antibacterials for systemic use	38.4	27.7	0.23	37.3	39.9	-0.05
Antidepressants	22.2	15.9	0.16	22.1	23.9	-0.04
Antiepileptics	5.9	4.4	0.07	5.3	5.8	-0.02
Antiinflammatory and antirheumatic products	20.5	16.3	0.11	20.5	21.5	-0.03

Table S6.137. *Continued.* Selected baseline characteristics for UK IMRD, for the long-term risk of psychoses

Characteristic	Before propensity score matching			After propensity score matching		
	Targets, %	Comparators, %	Standardized difference	Targets, %	Comparators, %	Standardized difference
**Medication use**						
Antipsoriatics	4.5	3	0.08	4.2	4.5	-0.02
Antithrombotic agents	10.1	9	0.04	8.1	8.3	-0.01
Beta blocking agents	10	8.7	0.04	9.1	9.7	-0.02
Diuretics	6.9	6	0.03	5.6	5.8	-0.01
Drugs for acid-related disorders	25.8	20.6	0.12	24.9	27	-0.05
Drugs for obstructive airway diseases	23.7	19.1	0.11	23.9	25	-0.03
Drugs used in diabetes	6.5	4.9	0.07	5.7	6.1	-0.02
Immunosuppressants	1	0.9	0.01	0.9	1	0
Lipid modifying agents	13.9	14.2	-0.01	12.6	12.8	-0.01
Opioids	17.6	12.8	0.14	17.3	18.6	-0.03
Psycholeptics	10.3	7.6	0.1	9.2	10	-0.03
Psychostimulants, agents used for ADHD and nootropics	0.2	0.3	-0.01	0.2	0.3	-0.01

Table S6.138. Selected baseline characteristics for UK IMRD, for the long-term risk of personality disorders

Characteristic	Before propensity score matching			After propensity score matching		
	Targets, %	Comparators, %	Standardized difference	Targets, %	Comparators, %	Standardized difference
**Age group (years)**						
0-4	1.7	3.9	-0.13	1.7	1.8	-0.01
5-9	2.3	5.2	-0.15	2.4	2.3	0.01
10-14	3.8	5.3	-0.07	3.8	3.5	0.02
15-19	6	4.9	0.05	6.1	6.2	0
20-24	7.3	5.2	0.09	7.5	7.9	-0.02
25-29	7.6	6.2	0.06	7.6	7.7	0
30-34	7.7	6.9	0.03	7.9	8	0
35-39	8.1	6.9	0.05	8.3	8.2	0
40-44	7.6	6.5	0.04	8	7.9	0
45-49	8.1	6.7	0.06	8.8	8.8	0
50-54	8.9	7.4	0.06	9.4	9.7	-0.01
55-59	8.4	7.4	0.04	9	9.2	0
60-64	6	6.5	-0.02	6.6	6.5	0
65-69	3.6	5.6	-0.1	3.7	3.6	0
70-74	3.2	5.7	-0.12	3	2.7	0.01
75-79	2.8	4.1	-0.07	2.3	2.1	0.01
80-84	2.5	2.8	-0.02	1.7	1.6	0.01
85-89	2.1	1.8	0.02	1.1	1.2	0
90-94	1.4	0.8	0.06	0.6	0.7	-0.01
95-99	0.6	0.2	0.06	0.2	0.2	0
**Sex**						
Female	56.7	49.4	0.15	57.1	59.1	-0.04

Table S6.138. *Continued.* Selected baseline characteristics for UK IMRD, for the long-term risk of personality disorders

	Before propensity score matching			After propensity score matching		
	**Targets, %**	**Comparators, %**	**Standardized difference**	**Targets, %**	**Comparators, %**	**Standardized difference**
**Race**						
race = Asian	0.8	0.6	0.02	0.7	0.6	0.01
race = Black or African American	0.2	0.3	-0.01	0.1	0.2	-0.01
race = White	4.5	6.6	-0.09	4.4	3.9	0.03
race = Asian Indian	0.9	0.8	0.01	0.6	0.6	0
race = Bangladeshi	0.2	0.1	0.02	0.2	0.1	0.02
race = Pakistani	1.7	0.6	0.1	1.9	2.1	-0.01
race = African	0.7	0.9	-0.02	0.4	0.4	-0.01
race = European	36.7	36.1	0.01	36.2	37.2	-0.02
**Medical history (general)**						
Acute respiratory disease	4.4	2.6	0.1	4.3	4.7	-0.02
Attention deficit hyperactivity disorder	0.1	0.1	0	0.1	0.1	0
Chronic obstructive lung disease	0.7	0.6	0.02	0.6	0.7	-0.01
Dementia	0.4	0.2	0.04	0.2	0.3	-0.01
Depressive disorder	1.4	1.1	0.03	1.4	1.5	-0.01
Diabetes mellitus	0.7	0.6	0.01	0.7	0.8	-0.01
Gastroesophageal reflux disease	0.3	0.2	0.02	0.4	0.4	-0.01
Gastrointestinal hemorrhage	0.5	0.4	0.02	0.5	0.5	-0.01
Hyperlipidemia	0.2	0.2	0	0.2	0.2	0
Hypertensive disorder	0.9	0.8	0.01	0.9	1	-0.01
Lesion of liver	0.1	0.1	0.01	0.1	0.1	-0.01
Obesity	0.2	0.1	0.02	0.2	0.2	0
Osteoarthritis	0.8	0.7	0.01	0.8	0.8	0

Table S6.138. *Continued.* Selected baseline characteristics for UK IMRD, for the long-term risk of personality disorders

Characteristic	Before propensity score matching			After propensity score matching		
	**Targets, %**	**Comparators, %**	**Standardized difference**	**Targets, %**	**Comparators, %**	**Standardized difference**
**Medical history (general)**						
Pneumonia	1.1	0.2	0.11	0.7	0.7	0
Renal impairment	1.1	0.5	0.06	0.8	0.8	0
Rheumatoid arthritis	0.1	0.1	0.01	0.1	0.1	0
Urinary tract infectious disease	2.1	1	0.09	1.8	2	-0.01
**Medical history (cardiovascular disease)**						
Atrial fibrillation	0.6	0.4	0.04	0.4	0.5	-0.01
Cerebrovascular disease	0.4	0.3	0.03	0.3	0.4	-0.01
Heart disease	1.7	1.1	0.05	1.3	1.5	-0.02
Heart failure	0.4	0.2	0.04	0.2	0.2	0
Ischemic heart disease	0.4	0.3	0.02	0.4	0.4	-0.01
Pulmonary embolism	0.2	0.1	0.04	0.2	0.1	0.01
Venous thrombosis	0.3	0.2	0.02	0.3	0.3	-0.01
**Medical history (neoplasms)**						
Malignant neoplastic disease	1	0.8	0.02	0.6	0.7	-0.01
Malignant tumor of breast	0.1	0.1	0	0.1	0.1	-0.01
**Medication use**						
Agents acting on the renin-angiotensin system	12.6	12.2	0.01	12	12.4	-0.01
Antibacterials for systemic use	38.4	27.6	0.23	37.3	39.9	-0.05
Antidepressants	22.2	16	0.16	22	23.8	-0.04
Antiepileptics	5.9	4.3	0.07	5.3	5.8	-0.02
Antiinflammatory and antirheumatic products	20.5	16.2	0.11	20.5	21.5	-0.03
Antipsoriatics	4.5	3.1	0.08	4.2	4.5	-0.02

Table S6.138. *Continued.* Selected baseline characteristics for UK IMRD, for the long-term risk of personality disorders

Characteristic	Before propensity score matching			After propensity score matching		
	**Targets, %**	**Comparators, %**	**Standardized difference**	**Targets, %**	**Comparators, %**	**Standardized difference**
**Medication use**						
Antithrombotic agents	10.1	9.2	0.03	8.2	8.4	-0.01
Beta blocking agents	10	8.8	0.04	9.1	9.6	-0.02
Calcium channel blockers	8.3	8.3	0	7.7	7.8	-0.01
Diuretics	6.9	6.1	0.03	5.6	5.8	-0.01
Drugs for acid-related disorders	25.8	20.6	0.12	24.9	27	-0.05
Drugs for obstructive airway diseases	23.7	19.2	0.11	23.9	25	-0.03
Drugs used in diabetes	6.5	4.9	0.07	5.7	6.1	-0.02
Immunosuppressants	1	0.9	0.01	0.9	1	0
Lipid modifying agents	13.9	14.3	-0.01	12.6	12.8	-0.01
Opioids	17.6	12.9	0.13	17.3	18.6	-0.03
Psycholeptics	10.3	7.7	0.09	9.2	10	-0.03
Psychostimulants, agents used for ADHD and nootropics	0.2	0.3	-0.01	0.2	0.3	-0.01

Table S6.139. Selected baseline characteristics for UK IMRD, for the long-term risk of self-harm and suicide

Characteristic	Before propensity score matching			After propensity score matching		
	Targets, %	Comparators, %	Standardized difference	Targets, %	Comparators, %	Standardized difference
**Age group (years)**						
0-4	1.7	3.9	-0.13	1.7	1.8	-0.01
5-9	2.3	5.3	-0.16	2.4	2.3	0.01
10-14	3.8	5.3	-0.07	3.8	3.5	0.02
15-19	6	4.8	0.05	6.1	6.2	0
20-24	7.3	5.2	0.09	7.5	8	-0.02
25-29	7.6	6.3	0.05	7.7	7.7	0
30-34	7.7	6.9	0.03	7.9	8	0
35-39	8.1	6.9	0.04	8.3	8.2	0
40-44	7.6	6.5	0.04	8	7.9	0
45-49	8.1	6.7	0.05	8.8	8.8	0
50-54	8.9	7.5	0.05	9.4	9.7	-0.01
55-59	8.4	7.3	0.04	9	9.2	0
60-64	6	6.4	-0.01	6.6	6.5	0
65-69	3.6	5.6	-0.1	3.7	3.6	0
70-74	3.2	5.7	-0.12	3	2.7	0.01
75-79	2.8	4.1	-0.07	2.3	2.1	0.01
80-84	2.5	2.9	-0.03	1.7	1.6	0.01
85-89	2.1	1.8	0.02	1.1	1.2	0
90-94	1.4	0.7	0.06	0.6	0.7	-0.01
95-99	0.6	0.2	0.06	0.2	0.2	0
**Sex**						
Female	56.7	49.5	0.14	57.1	59.1	-0.04

Table S6.139. *Continued.* Selected baseline characteristics for UK IMRD, for the long-term risk of self-harm and suicide

Characteristic	Before propensity score matching			After propensity score matching		
	Targets, %	Comparators, %	Standardized difference	Targets, %	Comparators, %	Standardized difference
**Medical history (general)**						
race = Asian	0.8	0.6	0.02	0.7	0.6	0.01
race = Black or African American	0.2	0.3	-0.02	0.1	0.2	-0.01
race = White	4.5	6.7	-0.09	4.4	3.9	0.03
race = Asian Indian	0.9	0.9	0.01	0.6	0.6	0
race = Bangladeshi	0.2	0.1	0.02	0.2	0.1	0.02
race = Pakistani	1.7	0.6	0.11	1.9	2.1	-0.01
race = African	0.7	0.9	-0.02	0.4	0.4	0
race = European	36.7	36.2	0.01	36.2	37.2	-0.02
Medical history: General						
Acute respiratory disease	4.4	2.6	0.1	4.3	4.7	-0.02
Attention deficit hyperactivity disorder	0.1	0.1	0	0.1	0.1	0
Chronic obstructive lung disease	0.7	0.6	0.02	0.6	0.7	-0.01
Dementia	0.4	0.2	0.04	0.2	0.3	-0.01
Depressive disorder	1.4	1.1	0.04	1.4	1.5	-0.01
Diabetes mellitus	0.7	0.6	0.01	0.7	0.8	-0.01
Gastroesophageal reflux disease	0.3	0.2	0.02	0.3	0.4	-0.01
Gastrointestinal hemorrhage	0.5	0.4	0.02	0.5	0.6	-0.01
Hyperlipidemia	0.2	0.2	0	0.2	0.2	0
Hypertensive disorder	0.9	0.8	0.01	0.9	1	-0.01
Lesion of liver	0.1	0.1	0.01	0.1	0.1	-0.01
Obesity	0.2	0.1	0.02	0.2	0.2	0
Osteoarthritis	0.8	0.8	0.01	0.8	0.8	0

Table S6.139. *Continued.* Selected baseline characteristics for UK IMRD, for the long-term risk of self-harm and suicide

Characteristic	Before propensity score matching			After propensity score matching		
	**Targets, %**	**Comparators, %**	**Standardized difference**	**Targets, %**	**Comparators, %**	**Standardized difference**
**Medical history (cardiovascular disease)**						
Pneumonia	1.1	0.2	0.11	0.7	0.7	0
Renal impairment	1.1	0.5	0.07	0.8	0.8	0
Rheumatoid arthritis	0.1	0.1	0.01	0.1	0.1	0
Urinary tract infectious disease	2.1	1	0.09	1.8	2	-0.01
Medical history: Cardiovascular disease						
Atrial fibrillation	0.6	0.4	0.04	0.4	0.5	-0.01
Cerebrovascular disease	0.4	0.2	0.03	0.3	0.4	-0.01
Coronary arteriosclerosis	0.1	0.1	0	0.1	0.1	0.01
Heart disease	1.7	1.1	0.06	1.3	1.5	-0.02
Heart failure	0.4	0.2	0.05	0.2	0.2	0
Ischemic heart disease	0.4	0.3	0.03	0.4	0.4	-0.01
Pulmonary embolism	0.2	0.1	0.03	0.2	0.1	0.01
Venous thrombosis	0.3	0.2	0.02	0.3	0.3	-0.01
**Medical history (neoplasms)**						
Malignant neoplastic disease	1	0.7	0.03	0.6	0.7	-0.01
Malignant tumor of breast	0.1	0.1	0	0.1	0.1	-0.01
**Medication use**						
Agents acting on the renin-angiotensin system	12.6	12.2	0.01	12	12.5	-0.01
Antibacterials for systemic use	38.4	27.7	0.23	37.3	39.9	-0.05
Antidepressants	22.2	16	0.16	22	23.8	-0.04
Antiepileptics	5.9	4.3	0.07	5.3	5.8	-0.02
Antiinflammatory and antirheumatic products	20.5	16.5	0.1	20.5	21.6	-0.03

Table S6.139. *Continued.* Selected baseline characteristics for UK IMRD, for the long-term risk of self-harm and suicide

Characteristic	Before propensity score matching			After propensity score matching		
	**Targets, %**	**Comparators, %**	**Standardized difference**	**Targets, %**	**Comparators, %**	**Standardized difference**
**Medical history (cardiovascular disease)**						
Antineoplastic agents	1	1	0	0.9	0.9	0
Antipsoriatics	4.5	2.9	0.08	4.2	4.5	-0.02
Antithrombotic agents	10.1	9.1	0.04	8.2	8.4	-0.01
Beta blocking agents	10	8.8	0.04	9.1	9.7	-0.02
Calcium channel blockers	8.3	8.2	0	7.7	7.8	-0.01
Diuretics	6.9	6	0.03	5.6	5.8	-0.01
Drugs for acid-related disorders	25.8	20.6	0.12	24.9	27	-0.05
Drugs for obstructive airway diseases	23.7	19.2	0.11	23.9	25	-0.03
Drugs used in diabetes	6.5	4.8	0.07	5.7	6.1	-0.02
Immunosuppressants	1	0.9	0.01	0.9	1	0
Lipid modifying agents	13.9	14.3	-0.01	12.6	12.8	-0.01
Opioids	17.6	12.9	0.13	17.3	18.6	-0.03
Psycholeptics	10.3	7.7	0.09	9.2	10	-0.03
Psychostimulants, agents used for ADHD and nootropics	0.2	0.3	-0.01	0.2	0.3	-0.01

Table S6.140. Selected baseline characteristics for UK IMRD, for the long-term risk of sleep disorders

Characteristic	Before propensity score matching			After propensity score matching		
	Targets, %	Comparators, %	Standardized difference	Targets, %	Comparators, %	Standardized difference
**Age group (years)**						
0-4	1.7	3.9	-0.14	1.8	1.8	0
5-9	2.3	5.3	-0.16	2.4	2.3	0.01
10-14	3.8	5.3	-0.07	3.9	3.5	0.02
15-19	6	4.9	0.04	6.2	6.3	0
20-24	7.3	5.2	0.09	7.6	8	-0.02
25-29	7.6	6.2	0.06	7.7	7.8	0
30-34	7.7	7	0.03	7.9	8	0
35-39	8.1	7	0.04	8.3	8.2	0
40-44	7.6	6.4	0.05	8	7.9	0
45-49	8.1	6.7	0.06	8.8	8.7	0
50-54	8.9	7.4	0.06	9.4	9.7	-0.01
55-59	8.4	7.3	0.04	9	9.1	0
60-64	6	6.4	-0.01	6.5	6.5	0
65-69	3.6	5.6	-0.1	3.6	3.6	0
70-74	3.2	5.6	-0.12	3	2.7	0.01
75-79	2.8	4	-0.06	2.3	2.1	0.01
80-84	2.5	2.9	-0.02	1.7	1.6	0.01
85-89	2.1	1.8	0.02	1.1	1.2	-0.01
90-94	1.4	0.8	0.06	0.6	0.7	-0.01
95-99	0.6	0.2	0.06	0.2	0.2	0
**Sex**						
Female	56.7	49.4	0.14	57	59.1	-0.04

Table S6.140. *Continued.* Selected baseline characteristics for UK IMRD, for the long-term risk of sleep disorders

Characteristic	Before propensity score matching			After propensity score matching		
	Targets, %	Comparators, %	Standardized difference	Targets, %	Comparators, %	Standardized difference
**Race**						
race = Asian	0.8	0.6	0.02	0.7	0.6	0.01
race = Black or African American	0.2	0.3	-0.01	0.1	0.2	-0.01
race = White	4.5	6.7	-0.1	4.4	3.9	0.03
race = Asian Indian	0.9	0.9	0.01	0.6	0.6	0
race = Bangladeshi	0.2	0.1	0.02	0.2	0.1	0.02
race = Pakistani	1.7	0.6	0.11	1.9	2.1	-0.01
race = African	0.7	0.9	-0.02	0.4	0.4	-0.01
race = European	36.7	36.5	0	36.2	37.2	-0.02
**Medical history (general)**						
Acute respiratory disease	4.4	2.6	0.1	4.2	4.6	-0.02
Attention deficit hyperactivity disorder	0.1	0.1	0	0.1	0.1	0
Chronic obstructive lung disease	0.7	0.6	0.02	0.5	0.7	-0.01
Dementia	0.4	0.2	0.04	0.2	0.3	-0.01
Depressive disorder	1.4	1.1	0.04	1.3	1.5	-0.01
Diabetes mellitus	0.7	0.6	0.01	0.7	0.8	-0.01
Gastroesophageal reflux disease	0.3	0.2	0.02	0.3	0.4	0
Gastrointestinal hemorrhage	0.5	0.4	0.02	0.5	0.5	-0.01
Hypertensive disorder	0.9	0.8	0.01	0.9	1	-0.01
Lesion of liver	0.1	0.1	0.01	0.1	0.1	-0.01
Obesity	0.2	0.1	0.02	0.2	0.2	0
Osteoarthritis	0.8	0.7	0.01	0.8	0.8	0
Pneumonia	1.1	0.2	0.11	0.7	0.7	0

Table S6.140. *Continued.* Selected baseline characteristics for UK IMRD, for the long-term risk of sleep disorders

Characteristic	Before propensity score matching			After propensity score matching		
	Targets, %	Comparators, %	Standardized difference	Targets, %	Comparators, %	Standardized difference
**Medical history (general)**						
Psoriasis	0.4	0.3	0.02	0.3	0.3	0
Rheumatoid arthritis	0.1	0.1	0.01	0.1	0.1	0
Urinary tract infectious disease	2.1	1	0.1	1.8	1.9	-0.01
**Medical history (cardiovascular disease)**						
Atrial fibrillation	0.6	0.3	0.04	0.4	0.5	-0.01
Cerebrovascular disease	0.4	0.3	0.03	0.3	0.4	-0.01
Heart disease	1.7	1.1	0.06	1.2	1.4	-0.02
Heart failure	0.4	0.2	0.04	0.2	0.2	0
Ischemic heart disease	0.4	0.3	0.02	0.3	0.4	-0.01
Pulmonary embolism	0.2	0.1	0.03	0.2	0.1	0.01
Venous thrombosis	0.3	0.2	0.02	0.3	0.3	-0.01
**Medical history (neoplasms)**						
Malignant neoplastic disease	1	0.8	0.02	0.6	0.7	-0.01
Malignant tumor of breast	0.1	0.1	0	0.1	0.1	-0.01
**Medication use**						
Agents acting on the renin-angiotensin system	12.6	12.2	0.01	11.8	12.3	-0.01
Antibacterials for systemic use	38.4	27.6	0.23	37.1	39.7	-0.05
Antidepressants	22.2	15.9	0.16	21.7	23.4	-0.04
Antiepileptics	5.9	4.3	0.07	5.2	5.7	-0.02
Antiinflammatory and antirheumatic products	20.5	16.4	0.11	20.3	21.3	-0.03
Antineoplastic agents	1	0.9	0	0.9	0.9	0
Antipsoriatics	4.5	2.9	0.09	4.2	4.5	-0.02

Table S6.140. *Continued.* Selected baseline characteristics for UK IMRD, for the long-term risk of sleep disorders

Characteristic	Before propensity score matching			After propensity score matching		
	**Targets, %**	**Comparators, %**	**Standardized difference**	**Targets, %**	**Comparators, %**	**Standardized difference**
**Medication use**						
Antithrombotic agents	10.1	9.1	0.03	8	8.2	-0.01
Beta blocking agents	10	8.8	0.04	8.9	9.5	-0.02
Calcium channel blockers	8.3	8.3	0	7.6	7.8	-0.01
Diuretics	6.9	6.1	0.03	5.4	5.7	-0.01
Drugs for acid-related disorders	25.8	20.6	0.12	24.7	26.7	-0.05
Drugs for obstructive airway diseases	23.7	19.2	0.11	23.7	24.8	-0.03
Drugs used in diabetes	6.5	4.9	0.07	5.6	6	-0.02
Immunosuppressants	1	0.9	0.01	0.9	0.9	0
Lipid modifying agents	13.9	14.2	-0.01	12.5	12.7	-0.01
Opioids	17.6	12.9	0.13	17	18.3	-0.03
Psycholeptics	10.3	7.7	0.09	8.9	9.7	-0.03
Psychostimulants, agents used for ADHD and nootropics	0.2	0.3	-0.01	0.2	0.3	-0.01

Table S6.141. Selected baseline characteristics for UK IMRD, for the long-term risk of dementia

Characteristic	Before propensity score matching			After propensity score matching		
	Targets, %	Comparators, %	Standardized difference	Targets, %	Comparators, %	Standardized difference
**Age group (years)**						
0-4	1.7	3.9	-0.14	1.8	1.8	-0.01
5-9	2.3	5.2	-0.15	2.4	2.3	0.01
10-14	3.8	5.2	-0.07	3.9	3.6	0.02
15-19	6	5	0.04	6.2	6.3	0
20-24	7.3	5.2	0.09	7.6	8	-0.01
25-29	7.6	6.2	0.05	7.8	7.8	0
30-34	7.7	6.9	0.03	8	8.1	0
35-39	8.1	7	0.04	8.4	8.3	0
40-44	7.6	6.4	0.05	8.1	8	0
45-49	8.1	6.8	0.05	8.9	8.8	0
50-54	8.9	7.4	0.06	9.5	9.8	-0.01
55-59	8.4	7.3	0.04	9.1	9.2	0
60-64	6	6.5	-0.02	6.6	6.6	0
65-69	3.6	5.6	-0.1	3.7	3.6	0
70-74	3.2	5.7	-0.12	2.9	2.7	0.01
75-79	2.8	4	-0.07	2.1	1.9	0.01
80-84	2.5	2.9	-0.02	1.4	1.4	0
85-89	2.1	1.7	0.03	0.9	1	-0.01
90-94	1.4	0.8	0.06	0.4	0.5	-0.01
95-99	0.6	0.2	0.06	0.2	0.2	-0.01
**Sex**						
Female	56.7	49.5	0.14	57	59	-0.04

Table S6.141. *Continued.* Selected baseline characteristics for UK IMRD, for the long-term risk of dementia

Characteristic	Before propensity score matching			After propensity score matching		
	Targets, %	Comparators, %	Standardized difference	Targets, %	Comparators, %	Standardized difference
**Race**						
race = Asian	0.8	0.6	0.02	0.7	0.6	0.01
race = Black or African American	0.2	0.3	-0.02	0.1	0.2	-0.01
race = White	4.5	6.7	-0.1	4.4	3.9	0.03
race = Asian Indian	0.9	0.8	0.01	0.6	0.6	0
race = Bangladeshi	0.2	0.1	0.01	0.2	0.1	0.02
race = Pakistani	1.7	0.6	0.11	1.9	2.1	-0.01
race = African	0.7	0.9	-0.02	0.4	0.4	-0.01
race = European	36.7	36.3	0.01	36.2	37.2	-0.02
**Medical history (general)**						
Acute respiratory disease	4.4	2.6	0.1	4.3	4.7	-0.02
Attention deficit hyperactivity disorder	0.1	0.1	0.01	0.1	0.1	0
Chronic obstructive lung disease	0.7	0.6	0.02	0.6	0.7	-0.01
Depressive disorder	1.4	1	0.04	1.4	1.5	-0.01
Diabetes mellitus	0.7	0.6	0.01	0.7	0.8	-0.02
Gastroesophageal reflux disease	0.3	0.2	0.02	0.4	0.4	-0.01
Gastrointestinal hemorrhage	0.5	0.4	0.02	0.5	0.5	-0.01
Hyperlipidemia	0.2	0.2	0	0.2	0.2	0
Hypertensive disorder	0.9	0.8	0	0.9	1	-0.01
Lesion of liver	0.1	0.1	0.01	0.1	0.1	-0.01
Obesity	0.2	0.1	0.02	0.2	0.2	0
Osteoarthritis	0.8	0.8	0.01	0.8	0.8	0
Pneumonia	1.1	0.2	0.11	0.7	0.6	0

Table S6.141. *Continued.* Selected baseline characteristics for UK IMRD, for the long-term risk of dementia

Characteristic	Before propensity score matching			After propensity score matching		
	Targets, %	Comparators, %	Standardized difference	Targets, %	Comparators, %	Standardized difference
**Medical history (general)**						
Renal impairment	1.1	0.5	0.07	0.7	0.7	0
Rheumatoid arthritis	0.1	0.1	0.01	0.1	0.1	0
Urinary tract infectious disease	2.1	1	0.09	1.7	1.9	-0.02
**Medical history (cardiovascular disease)**						
Atrial fibrillation	0.6	0.3	0.04	0.4	0.5	-0.01
Cerebrovascular disease	0.4	0.3	0.03	0.3	0.3	-0.01
Coronary arteriosclerosis	0.1	0.1	0	0.1	0.1	0.01
Heart disease	1.7	1.1	0.06	1.2	1.4	-0.01
Heart failure	0.4	0.2	0.04	0.2	0.2	0
Ischemic heart disease	0.4	0.3	0.02	0.4	0.4	-0.01
Pulmonary embolism	0.2	0.1	0.03	0.2	0.1	0.01
Venous thrombosis	0.3	0.2	0.02	0.3	0.3	-0.01
**Medical history (neoplasms)**						
Malignant neoplastic disease	1	0.8	0.02	0.6	0.7	-0.01
Malignant tumor of breast	0.1	0.1	0	0.1	0.1	-0.01
**Medication use**						
Agents acting on the renin-angiotensin system	12.6	12.2	0.01	11.8	12.3	-0.01
Antibacterials for systemic use	38.4	27.6	0.23	36.9	39.6	-0.06
Antidepressants	22.2	16	0.16	21.8	23.6	-0.04
Antiepileptics	5.9	4.3	0.07	5.2	5.7	-0.02
Antiinflammatory and antirheumatic products	20.5	16.3	0.11	20.3	21.4	-0.03
Antineoplastic agents	1	1	0	0.9	0.9	0

Table S6.141. *Continued.* Selected baseline characteristics for UK IMRD, for the long-term risk of dementia

Characteristic	Before propensity score matching			After propensity score matching		
	Targets, %	Comparators, %	Standardized difference	Targets, %	Comparators, %	Standardized difference
**Medication use**						
Antipsoriatics	4.5	2.9	0.08	4.1	4.5	-0.02
Antithrombotic agents	10.1	9.2	0.03	7.6	7.9	-0.01
Beta blocking agents	10	8.8	0.04	8.9	9.5	-0.02
Calcium channel blockers	8.3	8.3	0	7.5	7.7	-0.01
Diuretics	6.9	6.1	0.03	5.3	5.6	-0.01
Drugs for acid-related disorders	25.8	20.6	0.12	24.5	26.6	-0.05
Drugs for obstructive airway diseases	23.7	19	0.12	23.8	25	-0.03
Drugs used in diabetes	6.5	4.9	0.07	5.6	6	-0.02
Immunosuppressants	1	0.9	0.01	0.9	0.9	0
Lipid modifying agents	13.9	14.3	-0.01	12.2	12.4	-0.01
Opioids	17.6	13	0.13	17.1	18.4	-0.03
Psycholeptics	10.3	7.8	0.09	8.9	9.8	-0.03
Psychostimulants, agents used for ADHD and nootropics	0.2	0.3	-0.01	0.2	0.3	-0.01

Table S6.142. Selected baseline characteristics for UK IMRD, for the long-term risk of neurodevelopmental disorders

Characteristic	Before propensity score matching			After propensity score matching		
	Targets, %	Comparators, %	Standardized difference	Targets, %	Comparators, %	Standardized difference
**Age group (years)**						
0-4	1.7	3.9	-0.14	1.7	1.8	-0.01
5-9	2.3	5.2	-0.15	2.4	2.3	0.01
10-14	3.8	5.2	-0.07	3.8	3.5	0.02
15-19	6	4.9	0.04	6.1	6.2	0
20-24	7.3	5.2	0.09	7.5	8	-0.02
25-29	7.6	6.2	0.06	7.6	7.7	0
30-34	7.7	6.9	0.03	7.9	8	0
35-39	8.1	7	0.04	8.3	8.2	0
40-44	7.6	6.5	0.04	8	7.9	0
45-49	8.1	6.7	0.06	8.8	8.8	0
50-54	8.9	7.5	0.05	9.4	9.7	-0.01
55-59	8.4	7.3	0.04	9.1	9.2	0
60-64	6	6.5	-0.02	6.6	6.5	0
65-69	3.6	5.6	-0.1	3.7	3.6	0
70-74	3.2	5.6	-0.12	3	2.7	0.01
75-79	2.8	4.1	-0.07	2.3	2.1	0.01
80-84	2.5	2.9	-0.02	1.7	1.6	0.01
85-89	2.1	1.8	0.02	1.1	1.2	0
90-94	1.4	0.8	0.06	0.6	0.7	-0.01
95-99	0.6	0.2	0.06	0.2	0.2	0
**Sex**						
Female	56.7	49.5	0.14	57.1	59.1	-0.04

Table S6.142. *Continued.* Selected baseline characteristics for UK IMRD, for the long-term risk of neurodevelopmental disorders

Characteristic	Before propensity score matching			After propensity score matching		
	Targets, %	Comparators, %	Standardized difference	Targets, %	Comparators, %	Standardized difference
**Race**						
race = Asian	0.8	0.7	0.01	0.7	0.6	0.01
race = Black or African American	0.2	0.3	-0.02	0.1	0.2	-0.01
race = White	4.5	6.6	-0.09	4.4	3.9	0.03
race = Asian Indian	0.9	0.9	0.01	0.6	0.6	0
race = Bangladeshi	0.2	0.1	0.02	0.2	0.1	0.02
race = Pakistani	1.7	0.6	0.11	1.9	2.1	-0.01
race = African	0.7	0.9	-0.03	0.4	0.4	-0.01
race = European	36.7	36.3	0.01	36.2	37.1	-0.02
**Medical history (general)**						
Acute respiratory disease	4.4	2.7	0.09	4.3	4.7	-0.02
Chronic obstructive lung disease	0.7	0.6	0.02	0.6	0.7	-0.01
Dementia	0.4	0.2	0.04	0.2	0.3	-0.01
Depressive disorder	1.4	1	0.04	1.4	1.5	-0.01
Diabetes mellitus	0.7	0.6	0.01	0.7	0.8	-0.01
Gastroesophageal reflux disease	0.3	0.2	0.02	0.4	0.4	-0.01
Gastrointestinal hemorrhage	0.5	0.4	0.02	0.5	0.6	-0.01
Hyperlipidemia	0.2	0.2	-0.01	0.2	0.2	0
Hypertensive disorder	0.9	0.8	0.01	0.9	1	-0.01
Lesion of liver	0.1	0.1	0.01	0.1	0.1	-0.01
Obesity	0.2	0.1	0.02	0.2	0.2	0
Osteoarthritis	0.8	0.7	0.01	0.8	0.8	0
Pneumonia	1.1	0.2	0.1	0.7	0.7	0

Table S6.142. *Continued.* Selected baseline characteristics for UK IMRD, for the long-term risk of neurodevelopmental disorders

Characteristic	Before propensity score matching			After propensity score matching		
	**Targets, %**	**Comparators, %**	**Standardized difference**	**Targets, %**	**Comparators, %**	**Standardized difference**
**Medical history (general)**						
Renal impairment	1.1	0.5	0.07	0.8	0.8	0
Rheumatoid arthritis	0.1	0.1	0	0.1	0.1	0
Urinary tract infectious disease	2.1	1	0.09	1.8	2	-0.01
**Medical history (cardiovascular disease)**						
Atrial fibrillation	0.6	0.4	0.04	0.4	0.5	-0.01
Cerebrovascular disease	0.4	0.3	0.03	0.3	0.4	-0.01
Coronary arteriosclerosis	0.1	0.1	0	0.1	0.1	0.01
Heart disease	1.7	1.1	0.06	1.3	1.5	-0.02
Heart failure	0.4	0.2	0.04	0.2	0.2	0
Ischemic heart disease	0.4	0.3	0.02	0.4	0.4	-0.01
Pulmonary embolism	0.2	0.1	0.03	0.2	0.1	0.01
Venous thrombosis	0.3	0.2	0.02	0.3	0.3	-0.01
**Medical history (neoplasms)**						
Malignant neoplastic disease	1	0.8	0.02	0.6	0.7	-0.01
Malignant tumor of breast	0.1	0.1	0	0.1	0.1	-0.01
**Medication use**						
Agents acting on the renin-angiotensin system	12.6	12.2	0.01	12	12.5	-0.01
Antibacterials for systemic use	38.4	27.7	0.23	37.3	39.9	-0.05
Antidepressants	22.2	15.9	0.16	22.1	23.8	-0.04
Antiepileptics	5.9	4.3	0.07	5.3	5.8	-0.02
Antiinflammatory and antirheumatic products	20.5	16.3	0.11	20.5	21.6	-0.03
Antipsoriatics	4.5	3	0.08	4.2	4.6	-0.02

Table S6.142. *Continued.* Selected baseline characteristics for UK IMRD, for the long-term risk of neurodevelopmental disorders

Characteristic	Before propensity score matching			After propensity score matching		
	**Targets, %**	**Comparators, %**	**Standardized difference**	**Targets, %**	**Comparators, %**	**Standardized difference**
**Medication use**						
Antithrombotic agents	10.1	9.2	0.03	8.1	8.4	-0.01
Beta blocking agents	10	8.8	0.04	9.1	9.7	-0.02
Calcium channel blockers	8.3	8.3	0	7.7	7.9	-0.01
Diuretics	6.9	6.1	0.03	5.6	5.8	-0.01
Drugs for acid-related disorders	25.8	20.6	0.12	24.9	27	-0.05
Drugs for obstructive airway diseases	23.7	19.1	0.11	23.9	25	-0.03
Drugs used in diabetes	6.5	4.9	0.07	5.7	6.1	-0.02
Immunosuppressants	1	0.9	0.01	0.9	1	0
Lipid modifying agents	13.9	14.3	-0.01	12.6	12.9	-0.01
Opioids	17.6	12.8	0.13	17.3	18.6	-0.03
Psycholeptics	10.3	7.7	0.09	9.2	10	-0.03
Psychostimulants, agents used for ADHD and nootropics	0.2	0.3	-0.01	0.2	0.2	-0.01

Table S6.143. elected baseline characteristics for UK IMRD, for the long-term risk of any of psychiatric and neuropsychiatric disorders

Characteristic	Before propensity score matching			After propensity score matching		
	Targets, %	Comparators, %	Standardized difference	Targets, %	Comparators, %	Standardized difference
**Age group (years)**						
0-4	1.7	3.9	-0.14	1.8	1.9	-0.01
5-9	2.3	5.2	-0.15	2.5	2.4	0.01
10-14	3.8	5.2	-0.07	4	3.7	0.02
15-19	6	4.9	0.05	6.3	6.3	0
20-24	7.3	5.3	0.09	7.5	7.9	-0.02
25-29	7.6	6.2	0.06	7.6	7.6	0
30-34	7.7	7	0.03	7.9	8	0
35-39	8.1	7	0.04	8.3	8.1	0
40-44	7.6	6.5	0.05	8	7.9	0
45-49	8.1	6.7	0.05	8.9	8.8	0
50-54	8.9	7.5	0.05	9.6	9.8	-0.01
55-59	8.4	7.3	0.04	9.3	9.3	0
60-64	6	6.5	-0.02	6.6	6.7	0
65-69	3.6	5.6	-0.1	3.7	3.7	0
70-74	3.2	5.7	-0.12	3	2.7	0.02
75-79	2.8	4	-0.06	2.1	2	0.01
80-84	2.5	2.8	-0.02	1.4	1.4	0
85-89	2.1	1.8	0.02	0.9	1	-0.01
90-94	1.4	0.8	0.06	0.4	0.6	-0.02
95-99	0.6	0.2	0.06	0.2	0.2	-0.01
**Sex**						
Female	56.7	49.3	0.15	56.4	58.5	-0.04

Table S6.143. *Continued.* Selected baseline characteristics for UK IMRD, for the long-term risk of any of psychiatric and neuropsychiatric disorders

Characteristic	Before propensity score matching			After propensity score matching		
	Targets,	Comparators,	Standardized	Targets,	Comparators,	Standardized
**Race**						
race = Asian	0.8	0.7	0.01	0.7	0.7	0.01
race = Black or African	0.2	0.3	-0.02	0.2	0.2	0
race = White	4.5	6.8	-0.1	4.4	3.9	0.02
race = Asian Indian	0.9	0.8	0.01	0.6	0.6	0
race = Bangladeshi	0.2	0.1	0.02	0.2	0.1	0.02
race = Pakistani	1.7	0.6	0.11	2	2.1	-0.01
race = African	0.7	0.9	-0.02	0.4	0.5	0
race = European	36.7	36	0.01	36.2	37.1	-0.02
**Medical history (general)**						
Acute respiratory disease	4.4	2.6	0.1	4.1	4.4	-0.02
Chronic obstructive lung disease	0.7	0.6	0.02	0.5	0.6	-0.01
Diabetes mellitus	0.7	0.6	0.01	0.7	0.7	-0.01
Gastroesophageal reflux disease	0.3	0.2	0.02	0.3	0.3	0
Gastrointestinal hemorrhage	0.5	0.4	0.02	0.4	0.5	-0.01
Hyperlipidemia	0.2	0.2	0	0.2	0.2	0
Hypertensive disorder	0.9	0.8	0.01	0.9	1	-0.01
Lesion of liver	0.1	0.1	0.01	0.1	0.1	-0.01
Pneumonia	1.1	0.2	0.11	0.6	0.6	0
Psoriasis	0.4	0.3	0.01	0.3	0.3	0
Renal impairment	1.1	0.5	0.07	0.7	0.7	0
Rheumatoid arthritis	0.1	0.1	0.01	0.1	0.1	0
Urinary tract infectious disease	2.1	1	0.09	1.6	1.8	-0.02

Table S6.143. *Continued.* Selected baseline characteristics for UK IMRD, for the long-term risk of any of psychiatric and neuropsychiatric disorders

Characteristic	Before propensity score matching			After propensity score matching		
	**Targets, %**	**Comparators, %**	**Standardized difference**	**Targets, %**	**Comparators, %**	**Standardized difference**
**Medical history (cardiovascular disease)**						
Atrial fibrillation	0.6	0.3	0.04	0.4	0.5	-0.01
Cerebrovascular disease	0.4	0.3	0.02	0.2	0.3	-0.01
Heart disease	1.7	1.1	0.06	1.2	1.4	-0.02
Heart failure	0.4	0.2	0.04	0.2	0.2	-0.01
Ischemic heart disease	0.4	0.3	0.03	0.3	0.4	-0.01
Pulmonary embolism	0.2	0.1	0.03	0.2	0.1	0.01
Venous thrombosis	0.3	0.2	0.02	0.2	0.3	-0.01
**Medical history (neoplasms)**						
Malignant neoplastic disease	1	0.7	0.02	0.6	0.7	-0.01
Malignant tumor of breast	0.1	0.1	0.01	0.1	0.1	-0.01
**Medication use**						
Agents acting on the renin-angiotensin system	12.6	12.2	0.01	11.8	12.2	-0.01
Antibacterials for systemic use	38.4	27.6	0.23	36.1	38.8	-0.06
Antidepressants	22.2	15.9	0.16	19.3	20.9	-0.04
Antiepileptics	5.9	4.2	0.08	4.9	5.3	-0.02
Antiinflammatory and antirheumatic products	20.5	16.4	0.11	19.9	20.8	-0.02
Antipsoriatics	4.5	3.1	0.08	4	4.3	-0.01
Antithrombotic agents	10.1	9.2	0.03	7.5	7.8	-0.01
Beta blocking agents	10	8.8	0.04	8.2	8.9	-0.02
Calcium channel blockers	8.3	8.3	0	7.4	7.7	-0.01
Diuretics	6.9	6.1	0.03	5.3	5.5	-0.01
Drugs for acid-related disorders	25.8	20.6	0.12	23.8	25.9	-0.05

Table S6.143. *Continued.* Selected baseline characteristics for UK IMRD, for the long-term risk of any of psychiatric and neuropsychiatric disorders

Characteristic	Before propensity score matching			After propensity score matching		
	Targets, %	Comparators, %	Standardized difference	Targets, %	Comparators, %	Standardized difference
**Medication use**						
Drugs for obstructive airway diseases	23.7	19.1	0.11	23.2	24.4	-0.03
Drugs used in diabetes	6.5	4.9	0.07	5.5	5.9	-0.02
Immunosuppressants	1	0.9	0.01	0.9	0.9	0
Lipid modifying agents	13.9	14.3	-0.01	12.1	12.4	-0.01
Opioids	17.6	12.9	0.13	16.5	17.6	-0.03
Psycholeptics	10.3	7.6	0.09	7.7	8.4	-0.03
Psychostimulants, agents used for ADHD and nootropics	0.2	0.3	-0.01	0.2	0.2	-0.01

Table S6.144. Selected baseline characteristics for US PharMetrics Plus for the short-term risk of depression

Characteristic	Before propensity score matching			After propensity score matching		
	Targets, %	Comparators, %	Standardized difference	Targets, %	Comparators, %	Standardized difference
**Age group (years)**						
0-4	0.7	3.7	-0.2	0.9	2.3	-0.11
5-9	1.2	5.5	-0.24	1.5	2.9	-0.1
10-14	2.3	6.1	-0.19	2.7	3.8	-0.06
15-19	6.1	6.6	-0.02	6.7	7.3	-0.03
20-24	9.1	7.5	0.06	9.8	10	-0.01
25-29	7.2	7	0.01	7.5	8.4	-0.03
30-34	7.7	7.2	0.02	8	8.5	-0.02
35-39	8.2	7.4	0.03	8.4	8.2	0.01
40-44	8.8	7.3	0.06	8.8	8.4	0.01
45-49	9.9	7.4	0.09	9.6	8.3	0.04
50-54	10.8	8	0.1	10.5	9.1	0.05
55-59	10.7	8.5	0.07	10.2	8.7	0.05
60-64	9.1	8.4	0.02	8.6	7.6	0.04
65-69	3.2	4.2	-0.05	2.9	2.5	0.02
70-74	1.6	1.9	-0.03	1.3	1.3	0
75-79	1.1	1.3	-0.02	0.8	0.8	0.01
80-84	2.4	1.9	0.03	1.7	1.9	-0.01
**Sex**						
Female	52.5	50.2	0.04	50.9	52.9	-0.04

Table S6.144. *Continued.* Selected baseline characteristics for US PharMetrics Plus for the short-term risk of depression

Characteristic	Before propensity score matching			After propensity score matching		
	Targets, %	Comparators, %	Standardized difference	Targets, %	Comparators, %	Standardized difference
**Medical history (general)**						
Acute respiratory disease	40.9	18.5	0.5	36.1	41.3	-0.11
Attention deficit hyperactivity disorder	3.2	2.9	0.02	2.7	3.3	-0.04
Chronic liver disease	0.4	0.2	0.03	0.3	0.3	0
Chronic obstructive lung disease	2.3	1.2	0.08	1.6	1.9	-0.02
Crohn’s disease	0.3	0.2	0.01	0.3	0.3	-0.01
Dementia	1.4	0.4	0.11	0.7	1.1	-0.04
Depressive disorder	10.3	6.9	0.12	0.4	0.1	0.07
Diabetes mellitus	12.2	5.9	0.22	10	8.6	0.05
Gastroesophageal reflux disease	11	5.8	0.19	9	9.3	-0.01
Gastrointestinal hemorrhage	1.6	0.8	0.07	1.3	1.4	-0.01
Human immunodeficiency virus infection	0.3	0.2	0.03	0.3	0.3	0
Hyperlipidemia	22.7	14.7	0.2	19.9	18.1	0.05
Hypertensive disorder	27.4	16.4	0.27	23.6	21.6	0.05
Lesion of liver	0.5	0.3	0.03	0.4	0.4	-0.01
Obesity	12.4	5.7	0.23	9.9	9.1	0.03
Osteoarthritis	11.5	7.1	0.15	9.7	9.3	0.01
Pneumonia	10.6	1.2	0.41	4.6	3.1	0.08
Psoriasis	1.1	0.8	0.03	1	1	0
Renal impairment	5	1.9	0.17	3.4	3.3	0
Rheumatoid arthritis	1.1	0.6	0.06	0.9	0.9	0
Ulcerative colitis	0.3	0.2	0.02	0.3	0.3	-0.01
Urinary tract infectious disease	5.5	2.5	0.16	4.4	4.7	-0.01
Viral hepatitis C	0.2	0.1	0.01	0.1	0.1	0

Table S6.144. *Continued.* Selected baseline characteristics for US PharMetrics Plus for the short-term risk of depression

Characteristic	Before propensity score matching			After propensity score matching		
	Targets, %	Comparators, %	Standardized difference	Targets, %	Comparators, %	Standardized difference
**Medical history (cardiovascular disease)**						
Atrial fibrillation	2.4	1.2	0.09	1.8	1.8	0
Cerebrovascular disease	1.2	0.7	0.05	0.9	1	-0.01
Coronary arteriosclerosis	4	2.3	0.1	3.2	3.1	0
Heart failure	2.4	0.9	0.12	1.7	1.8	-0.01
Ischemic heart disease	2.5	1.1	0.1	1.8	1.8	0
Peripheral vascular disease	2.1	0.9	0.1	1.5	1.7	-0.01
Venous thrombosis	0.8	0.4	0.06	0.6	0.6	0
**Medical history (neoplasms)**						
Malignant lymphoma	0.3	0.2	0.03	0.3	0.3	-0.01
Malignant neoplasm of anorectum	0.1	0.1	0.01	0.1	0.1	-0.01
Malignant neoplastic disease	3.9	3.1	0.04	3.4	3.7	-0.02
Malignant tumor of breast	0.7	0.6	0.01	0.5	0.7	-0.01
Malignant tumor of colon	0.2	0.1	0.01	0.1	0.2	0
Malignant tumor of lung	0.2	0.1	0.02	0.2	0.2	-0.01
Malignant tumor of urinary bladder	0.1	0.1	0.01	0.1	0.1	-0.01
Primary malignant neoplasm of prostate	0.5	0.4	0.02	0.4	0.4	0.01
**Medication use**						
Agents acting on the renin-angiotensin system	11.9	8	0.13	10.5	9.2	0.04
Antibacterials for systemic use	39.7	25.2	0.31	35.7	37.6	-0.04
Antidepressants	12.7	9	0.12	8.4	9.3	-0.03
Antiepileptics	6.1	3.8	0.1	4.4	4.8	-0.02
Antiinflammatory and antirheumatic products	19.8	10.8	0.25	17.3	16.8	0.01
Antineoplastic agents	2.8	1.9	0.06	2.5	2.8	-0.02
Antipsoriatics	1.8	1.3	0.05	1.8	1.9	-0.01
Antithrombotic agents	4.9	2.5	0.13	3.8	3.9	0

Table S6.144. *Continued.* Selected baseline characteristics for US PharMetrics Plus for the short-term risk of depression

Characteristic	Before propensity score matching			After propensity score matching		
	Targets, %	Comparators, %	Standardized difference	Targets, %	Comparators, %	Standardized difference
**Medication use**						
Beta blocking agents	7	4.5	0.11	5.9	5.5	0.01
Calcium channel blockers	5.3	3.5	0.09	4.6	4.3	0.02
Diuretics	8.9	5.5	0.13	7.6	7	0.03
Drugs for acid-related disorders	12.9	7.5	0.18	10.9	11.3	-0.01
Drugs for obstructive airway diseases	24.6	14.2	0.26	21.5	22.5	-0.02
Drugs used in diabetes	7.1	3.8	0.15	5.9	5	0.04
Immunosuppressants	1.7	1.1	0.05	1.4	1.4	0
Lipid modifying agents	11.4	7.9	0.12	9.9	8.8	0.04
Opioids	13.1	7.8	0.18	11.1	11.6	-0.02
Psycholeptics	13.3	8.6	0.15	10.6	11.5	-0.03
Psychostimulants, agents used for ADHD and nootropics	3.6	2.7	0.05	3	3.6	-0.03

Table S6.145. Selected baseline characteristics for US PharMetrics Plus for the short-term risk of anxiety disorders

Characteristic	Before propensity score matching			After propensity score matching		
	Targets, %	Comparators, %	Standardized difference	Targets, %	Comparators, %	Standardized difference
**Age group (years)**						
0-4	0.7	3.7	-0.2	1	2.4	-0.11
5-9	1.2	5.5	-0.24	1.5	3	-0.1
10-14	2.3	6.1	-0.19	2.7	3.8	-0.06
15-19	6.1	6.6	-0.02	6.7	7.3	-0.02
20-24	9.1	7.5	0.06	9.6	9.8	-0.01
25-29	7.2	7	0.01	7.5	8.2	-0.03
30-34	7.7	7.2	0.02	7.8	8.3	-0.02
35-39	8.2	7.4	0.03	8.2	8.1	0
40-44	8.8	7.2	0.06	8.5	8.1	0.02
45-49	9.9	7.4	0.09	9.6	8.3	0.04
50-54	10.8	8	0.1	10.5	9	0.05
55-59	10.7	8.6	0.07	10.4	8.8	0.05
60-64	9.1	8.4	0.02	8.8	7.6	0.04
65-69	3.2	4.1	-0.05	3	2.7	0.02
70-74	1.6	2	-0.03	1.4	1.4	0
75-79	1.1	1.3	-0.02	0.9	0.8	0.01
80-84	2.4	1.9	0.03	1.9	2.2	-0.02
**Sex**						
Female	52.5	50.1	0.05	50	51.8	-0.04

Table S6.145. *Continued.* Selected baseline characteristics for US PharMetrics Plus for the short-term risk of anxiety disorders

Characteristic	Before propensity score matching			After propensity score matching		
	Targets, %	Comparators, %	Standardized difference	Targets, %	Comparators, %	Standardized difference
**Medical history (general)**						
Acute respiratory disease	40.9	18.5	0.51	35.4	40.6	-0.11
Attention deficit hyperactivity disorder	3.2	2.9	0.02	2.3	2.7	-0.03
Chronic liver disease	0.4	0.2	0.03	0.3	0.4	-0.01
Chronic obstructive lung disease	2.3	1.2	0.08	1.6	1.9	-0.02
Crohn’s disease	0.3	0.2	0.01	0.3	0.4	-0.02
Dementia	1.4	0.4	0.1	0.9	1.3	-0.04
Depressive disorder	10.3	7	0.12	5.1	5.5	-0.02
Diabetes mellitus	12.2	6	0.22	10.3	9.1	0.04
Gastroesophageal reflux disease	11	5.8	0.19	8.5	8.8	-0.01
Gastrointestinal hemorrhage	1.6	0.8	0.06	1.2	1.4	-0.01
Human immunodeficiency virus infection	0.3	0.2	0.03	0.3	0.4	-0.01
Hyperlipidemia	22.7	14.8	0.2	20	18.2	0.04
Hypertensive disorder	27.4	16.5	0.27	23.6	21.5	0.05
Lesion of liver	0.5	0.2	0.04	0.4	0.4	-0.01
Obesity	12.4	5.8	0.23	9.8	9	0.03
Osteoarthritis	11.5	6.9	0.16	9.7	9.3	0.01
Pneumonia	10.6	1.2	0.41	4.7	3.1	0.08
Psoriasis	1.1	0.8	0.03	1	1	0
Renal impairment	5	1.9	0.17	3.6	3.5	0
Rheumatoid arthritis	1.1	0.6	0.06	0.9	0.9	0
Ulcerative colitis	0.3	0.2	0.02	0.3	0.3	0
Urinary tract infectious disease	5.5	2.5	0.15	4.4	4.7	-0.01
Viral hepatitis C	0.2	0.1	0.02	0.1	0.2	-0.01

Table S6.145. *Continued.* Selected baseline characteristics for US PharMetrics Plus for the short-term risk of anxiety disorders

Characteristic	Before propensity score matching			After propensity score matching		
	Targets, %	Comparators, %	Standardized difference	Targets, %	Comparators, %	Standardized difference
**Medical history (cardiovascular disease)**						
Cerebrovascular disease	1.2	0.7	0.05	0.9	1.1	-0.01
Coronary arteriosclerosis	4	2.3	0.1	3.3	3.3	0
Heart disease	10.3	5.7	0.17	8.3	8.4	0
Heart failure	2.4	0.9	0.12	1.8	1.9	-0.01
Ischemic heart disease	2.5	1.1	0.1	1.9	1.9	0
Peripheral vascular disease	2.1	0.9	0.1	1.6	1.8	-0.02
Pulmonary embolism	0.6	0.2	0.06	0.4	0.4	0
Venous thrombosis	0.8	0.4	0.06	0.6	0.7	-0.01
**Medical history (neoplasms)**						
Malignant lymphoma	0.3	0.2	0.02	0.3	0.3	0
Malignant neoplasm of anorectum	0.1	0.1	0	0.1	0.1	-0.01
Malignant neoplastic disease	3.9	3.1	0.04	3.4	3.7	-0.01
Malignant tumor of breast	0.7	0.6	0.01	0.6	0.6	-0.01
Malignant tumor of colon	0.2	0.1	0.01	0.1	0.2	-0.01
Malignant tumor of lung	0.2	0.1	0.02	0.2	0.2	-0.01
Malignant tumor of urinary bladder	0.1	0.1	0.01	0.1	0.1	-0.01
Primary malignant neoplasm of prostate	0.5	0.4	0.01	0.5	0.4	0.01
**Medication use**						
Agents acting on the renin-angiotensin system	11.9	8	0.13	10.5	9.2	0.04
Antibacterials for systemic use	39.7	25	0.32	35	36.8	-0.04
Antidepressants	12.7	9.1	0.11	7.4	8	-0.03
Antiepileptics	6.1	3.8	0.1	4.1	4.3	-0.01
Antiinflammatory and antirheumatic products	19.8	10.7	0.25	16.8	16.1	0.02
Antineoplastic agents	2.8	1.9	0.06	2.4	2.7	-0.02
Antipsoriatics	1.8	1.2	0.05	1.7	1.9	-0.01

Table S6.145. *Continued.* Selected baseline characteristics for US PharMetrics Plus for the short-term risk of anxiety disorders

Characteristic	Before propensity score matching			After propensity score matching		
	**Targets, %**	**Comparators, %**	**Standardized difference**	**Targets, %**	**Comparators, %**	**Standardized difference**
**Medication use**						
Antithrombotic agents	4.9	2.5	0.13	3.8	3.9	0
Beta blocking agents	7	4.6	0.1	5.7	5.3	0.02
Calcium channel blockers	5.3	3.4	0.09	4.5	4.3	0.01
Diuretics	8.9	5.4	0.13	7.6	6.9	0.03
Drugs for acid-related disorders	12.9	7.4	0.18	10.5	10.7	-0.01
Drugs for obstructive airway diseases	24.6	14.1	0.27	21	22	-0.03
Drugs used in diabetes	7.1	3.8	0.14	6.1	5.2	0.04
Immunosuppressants	1.7	1.1	0.05	1.4	1.4	0
Lipid modifying agents	11.4	7.9	0.12	10.1	9	0.04
Opioids	13.1	7.7	0.18	10.8	11.4	-0.02
Psycholeptics	13.3	8.5	0.15	9	9.4	-0.02
Psychostimulants, agents used for ADHD and nootropics	3.6	2.7	0.05	2.7	3.1	-0.03

Table S6.146. Selected baseline characteristics for US PharMetrics Plus for the short-term risk of alcohol misuse or dependence

Characteristic	Before propensity score matching			After propensity score matching		
	Targets, %	Comparators, %	Standardized difference	Targets, %	Comparators, %	Standardized difference
**Age group (years)**						
0-4	0.7	3.7	-0.2	0.8	2.1	-0.1
5-9	1.2	5.6	-0.24	1.4	2.7	-0.09
10-14	2.3	6.1	-0.19	2.6	3.6	-0.06
15-19	6.1	6.6	-0.02	6.6	7.1	-0.02
20-24	9.1	7.6	0.06	9.7	10.1	-0.01
25-29	7.2	7	0.01	7.5	8.3	-0.03
30-34	7.7	7.1	0.02	8	8.7	-0.03
35-39	8.2	7.4	0.03	8.4	8.4	0
40-44	8.8	7.3	0.06	8.8	8.3	0.02
45-49	9.9	7.3	0.09	9.6	8.3	0.05
50-54	10.8	8.1	0.1	10.5	9	0.05
55-59	10.7	8.6	0.07	10.2	8.7	0.05
60-64	9.1	8.5	0.02	8.5	7.5	0.04
65-69	3.2	4.1	-0.05	2.9	2.7	0.02
70-74	1.6	2	-0.03	1.4	1.4	0
75-79	1.1	1.3	-0.02	0.9	0.8	0.01
80-84	2.4	1.9	0.03	2.1	2.3	-0.01
**Sex**						
Female	52.5	50.1	0.05	53.1	55.4	-0.05

Table S6.146. *Continued.* Selected baseline characteristics for US PharMetrics Plus for the short-term risk of alcohol misuse or dependence

Characteristic	Before propensity score matching			After propensity score matching		
	Targets, %	Comparators, %	Standardized difference	Targets, %	Comparators, %	Standardized difference
**Medical history (general)**						
Acute respiratory disease	40.9	18.5	0.5	37	42.2	-0.11
Attention deficit hyperactivity disorder	3.2	2.9	0.02	3.3	4	-0.04
Chronic liver disease	0.4	0.2	0.03	0.3	0.3	0
Chronic obstructive lung disease	2.3	1.2	0.09	1.9	2.3	-0.03
Crohn’s disease	0.3	0.2	0.01	0.3	0.4	-0.02
Dementia	1.4	0.4	0.11	1.2	1.6	-0.04
Depressive disorder	10.3	6.8	0.12	9.8	11.4	-0.05
Diabetes mellitus	12.2	6	0.22	10.4	9.1	0.04
Gastroesophageal reflux disease	11	5.8	0.19	10.1	10.6	-0.02
Gastrointestinal hemorrhage	1.6	0.9	0.06	1.4	1.6	-0.02
Human immunodeficiency virus infection	0.3	0.2	0.03	0.3	0.3	-0.01
Hyperlipidemia	22.7	14.9	0.2	20.8	19.1	0.04
Hypertensive disorder	27.4	16.4	0.27	24.6	22.8	0.04
Lesion of liver	0.5	0.2	0.04	0.3	0.4	-0.01
Obesity	12.4	5.9	0.23	10.8	10.1	0.02
Osteoarthritis	11.5	7	0.16	10.7	10.4	0.01
Pneumonia	10.6	1.1	0.41	4.9	3.5	0.07
Psoriasis	1.1	0.8	0.03	1	1.1	-0.01
Renal impairment	5	1.9	0.17	3.7	3.8	0
Rheumatoid arthritis	1.1	0.6	0.06	1	1	0
Ulcerative colitis	0.3	0.2	0.02	0.3	0.4	-0.01
Urinary tract infectious disease	5.5	2.5	0.15	5	5.4	-0.01
Viral hepatitis C	0.2	0.1	0.01	0.1	0.2	-0.01

Table S6.146. *Continued.* Selected baseline characteristics for US PharMetrics Plus for the short-term risk of alcohol misuse or dependence

Characteristic	Before propensity score matching			After propensity score matching		
	Targets, %	Comparators, %	Standardized difference	Targets, %	Comparators, %	Standardized difference
**Medical history (cardiovascular disease)**						
Cerebrovascular disease	1.2	0.7	0.06	1.1	1.2	-0.01
Coronary arteriosclerosis	4	2.3	0.1	3.5	3.4	0
Heart disease	10.3	5.7	0.17	8.9	9.1	0
Heart failure	2.4	0.9	0.12	2	2.1	-0.01
Ischemic heart disease	2.5	1.1	0.1	2	2.1	0
Peripheral vascular disease	2.1	0.9	0.1	1.8	1.9	-0.01
Pulmonary embolism	0.6	0.2	0.06	0.4	0.5	-0.01
Venous thrombosis	0.8	0.4	0.06	0.7	0.7	-0.01
**Medical history (neoplasms)**						
Malignant lymphoma	0.3	0.2	0.02	0.3	0.3	-0.01
Malignant neoplasm of anorectum	0.1	0.1	0.01	0.1	0.1	-0.01
Malignant neoplastic disease	3.9	3.1	0.04	3.6	4	-0.02
Malignant tumor of breast	0.7	0.6	0.01	0.6	0.7	-0.01
Malignant tumor of lung	0.2	0.1	0.02	0.2	0.2	-0.02
Malignant tumor of urinary bladder	0.1	0.1	0.01	0.1	0.1	-0.01
Primary malignant neoplasm of prostate	0.5	0.4	0.01	0.4	0.4	0.01
**Medication use**						
Agents acting on the renin-angiotensin system	11.9	8	0.13	10.7	9.5	0.04
Antibacterials for systemic use	39.7	25.1	0.32	36.7	38.8	-0.04
Antidepressants	12.7	9	0.12	12.1	13.8	-0.05
Antiepileptics	6.1	3.9	0.1	5.5	6.2	-0.03
Antiinflammatory and antirheumatic products	19.8	10.8	0.25	18.1	17.9	0.01
Antineoplastic agents	2.8	1.9	0.06	2.7	3	-0.02
Antipsoriatics	1.8	1.3	0.04	1.9	2	-0.01
Antithrombotic agents	4.9	2.5	0.13	4.1	4.2	0

Table S6.146. *Continued.* Selected baseline characteristics for US PharMetrics Plus for the short-term risk of alcohol misuse or dependence

Characteristic	Before propensity score matching			After propensity score matching		
	Targets, %	Comparators, %	Standardized difference	Targets, %	Comparators, %	Standardized difference
**Medication use**						
Beta blocking agents	7	4.5	0.11	6.3	6	0.01
Calcium channel blockers	5.3	3.5	0.09	4.7	4.3	0.02
Diuretics	8.9	5.5	0.13	7.9	7.4	0.02
Drugs for acid-related disorders	12.9	7.4	0.18	11.8	12.4	-0.02
Drugs for obstructive airway diseases	24.6	14.3	0.26	22.5	23.7	-0.03
Drugs used in diabetes	7.1	3.8	0.15	6.2	5.3	0.04
Immunosuppressants	1.7	1.1	0.05	1.5	1.7	-0.01
Lipid modifying agents	11.4	8	0.11	10.2	9.3	0.03
Opioids	13.1	7.7	0.18	12.1	12.9	-0.03
Psycholeptics	13.3	8.6	0.15	12.5	13.9	-0.04
Psychostimulants, agents used for ADHD and nootropics	3.6	2.7	0.05	3.5	4.2	-0.04

Table S6.147. Selected baseline characteristics for US PharMetrics Plus for the short-term risk of substance misuse or dependence

Characteristic	Before propensity score matching			After propensity score matching		
	**Targets, %**	**Comparators, %**	**Standardized difference**	**Targets, %**	**Comparators, %**	**Standardized difference**
**Age group (years)**						
0-4	0.7	3.7	-0.2	0.8	2.2	-0.11
5-9	1.2	5.5	-0.24	1.4	2.7	-0.09
10-14	2.3	6.1	-0.19	2.6	3.8	-0.06
15-19	6.1	6.6	-0.02	6.8	7.6	-0.03
20-24	9.1	7.6	0.06	9.8	10.2	-0.01
25-29	7.2	7	0.01	7.6	8.4	-0.03
30-34	7.7	7.2	0.02	8	8.7	-0.03
35-39	8.2	7.4	0.03	8.3	8.3	0
40-44	8.8	7.3	0.06	8.8	8	0.03
45-49	9.9	7.3	0.09	9.6	8.4	0.04
50-54	10.8	7.9	0.1	10.5	8.8	0.06
55-59	10.7	8.6	0.07	10.1	8.5	0.05
60-64	9.1	8.5	0.02	8.5	7.3	0.04
65-69	3.2	4.1	-0.05	2.9	2.7	0.01
75-79	1.1	1.3	-0.02	0.9	0.8	0.01
80-84	2.4	1.9	0.04	2.1	2.3	-0.01
**Sex**						
Female	52.5	50.2	0.05	53.3	55.4	-0.04

Table S6.147. *Continued.* Selected baseline characteristics for US PharMetrics Plus for the short-term risk of substance misuse or dependence

Characteristic	Before propensity score matching			After propensity score matching		
	Targets, %	Comparators, %	Standardized difference	Targets, %	Comparators, %	Standardized difference
**Medical history (general)**						
Acute respiratory disease	40.9	18.6	0.5	36.5	41.7	-0.1
Attention deficit hyperactivity disorder	3.2	2.9	0.02	3.2	4	-0.04
Chronic obstructive lung disease	2.3	1.2	0.09	1.5	1.6	-0.01
Crohn’s disease	0.3	0.2	0.01	0.3	0.4	-0.02
Dementia	1.4	0.4	0.11	1.1	1.6	-0.04
Depressive disorder	10.3	6.9	0.12	9.3	10.6	-0.05
Diabetes mellitus	12.2	5.9	0.22	10.1	8.9	0.04
Gastroesophageal reflux disease	11	5.8	0.19	9.6	10	-0.01
Gastrointestinal hemorrhage	1.6	0.9	0.06	1.3	1.5	-0.02
Human immunodeficiency virus infection	0.3	0.2	0.03	0.3	0.3	-0.01
Hyperlipidemia	22.7	14.9	0.2	20.3	18.5	0.04
Hypertensive disorder	27.4	16.3	0.27	23.9	21.8	0.05
Lesion of liver	0.5	0.3	0.04	0.3	0.3	0
Obesity	12.4	5.8	0.23	10.4	9.6	0.03
Osteoarthritis	11.5	7	0.16	10.3	9.9	0.01
Pneumonia	10.6	1.1	0.41	4.6	3.1	0.08
Psoriasis	1.1	0.8	0.03	1	1	0
Renal impairment	5	1.8	0.18	3.5	3.5	0
Rheumatoid arthritis	1.1	0.5	0.06	1	0.9	0.01
Schizophrenia	0.1	0.1	0.01	0.1	0.1	0
Ulcerative colitis	0.3	0.2	0.02	0.3	0.4	-0.01
Urinary tract infectious disease	5.5	2.5	0.15	4.9	5.2	-0.02
Viral hepatitis C	0.2	0.1	0.01	0.1	0.1	0

Table S6.147. *Continued.* Selected baseline characteristics for US PharMetrics Plus for the short-term risk of substance misuse or dependence

Characteristic	Before propensity score matching			After propensity score matching		
	Targets, %	Comparators, %	Standardized diff.	Targets, %	Comparators, %	Standardized diff.
**Medical history (cardiovascular disease)**						
Atrial fibrillation	2.4	1.3	0.08	2	2	0
Cerebrovascular disease	1.2	0.7	0.05	1	1.2	-0.02
Coronary arteriosclerosis	4	2.2	0.1	3.3	3.2	0
Heart disease	10.3	5.7	0.17	8.5	8.5	0
Heart failure	2.4	0.9	0.12	1.8	1.9	-0.01
Peripheral vascular disease	2.1	0.9	0.1	1.7	1.8	-0.01
Pulmonary embolism	0.6	0.2	0.07	0.4	0.4	-0.01
Venous thrombosis	0.8	0.4	0.05	0.6	0.7	-0.01
**Medical history (neoplasms)**						
Malignant lymphoma	0.3	0.2	0.02	0.3	0.3	-0.01
Malignant neoplasm of anorectum	0.1	0.1	0.01	0.1	0.1	-0.01
Malignant neoplastic disease	3.9	3	0.05	3.5	3.8	-0.02
Malignant tumor of breast	0.7	0.6	0.01	0.6	0.7	-0.01
Malignant tumor of lung	0.2	0.1	0.01	0.1	0.2	-0.01
Malignant tumor of urinary bladder	0.1	0.1	0.01	0.1	0.1	-0.01
Primary malignant neoplasm of prostate	0.5	0.4	0.01	0.4	0.4	0.01
**Medication use**						
Agents acting on the renin-angiotensin system	11.9	8	0.13	10.4	9	0.05
Antibacterials for systemic use	39.7	24.9	0.32	36.1	37.9	-0.04
Antidepressants	12.7	9.2	0.11	11.7	13.2	-0.05
Antiepileptics	6.1	3.9	0.1	5.2	5.6	-0.02
Antiinflammatory and antirheumatic products	19.8	10.7	0.25	17.5	16.9	0.02
Antineoplastic agents	2.8	1.9	0.06	2.6	2.9	-0.02
Antipsoriatics	1.8	1.2	0.05	1.9	2.1	-0.01
Antithrombotic agents	4.9	2.5	0.13	3.8	3.9	0
Beta blocking agents	7	4.5	0.1	6	5.7	0.01

Table S6.147. *Continued.* Selected baseline characteristics for US PharMetrics Plus for the short-term risk of substance misuse or dependence

Characteristic	Before propensity score matching			After propensity score matching		
	Targets, %	Comparators, %	Standardized difference	Targets, %	Comparators, %	Standardized difference
**Medication use**						
Calcium channel blockers	5.3	3.4	0.09	4.6	4.2	0.02
Diuretics	8.9	5.5	0.13	7.7	7	0.03
Drugs for acid-related disorders	12.9	7.5	0.18	11.3	11.6	-0.01
Drugs for obstructive airway diseases	24.6	14.2	0.26	22	22.9	-0.02
Drugs used in diabetes	7.1	3.8	0.14	6	5.2	0.04
Immunosuppressants	1.7	1	0.05	1.5	1.6	-0.01
Lipid modifying agents	11.4	8	0.12	10	8.8	0.04
Opioids	13.1	7.7	0.18	11.3	11.8	-0.02
Psycholeptics	13.3	8.6	0.15	11.8	12.9	-0.03
Psychostimulants, agents used for ADHD and nootropics	3.6	2.7	0.05	3.4	4.2	-0.04

Table S6.148. Selected baseline characteristics for US PharMetrics Plus for the short-term risk of bipolar disorders

**Characteristic**	**Before propensity score matching**			**After propensity score matching**		
	**Targets, %**	**Comparators, %**	**Standardized difference**	**Targets, %**	**Comparators, %**	**Standardized difference**
**Age group (years)**						
0-4	0.7	3.7	-0.2	0.8	2	-0.1
5-9	1.2	5.5	-0.24	1.4	2.7	-0.09
10-14	2.3	6.1	-0.19	2.5	3.5	-0.06
15-19	6.1	6.6	-0.02	6.6	7.3	-0.03
20-24	9.1	7.5	0.06	9.7	10	-0.01
25-29	7.2	6.9	0.01	7.5	8.3	-0.03
30-34	7.7	7.1	0.02	8	8.6	-0.02
35-39	8.2	7.4	0.03	8.4	8.2	0
40-44	8.8	7.4	0.05	8.8	8.3	0.02
45-49	9.9	7.4	0.09	9.6	8.5	0.04
50-54	10.8	8.1	0.1	10.5	9.1	0.05
55-59	10.7	8.6	0.07	10.2	8.7	0.05
60-64	9.1	8.5	0.02	8.6	7.6	0.04
65-69	3.2	4.2	-0.05	2.9	2.7	0.02
75-79	1.1	1.3	-0.02	0.9	0.8	0.01
80-84	2.4	1.8	0.04	2.1	2.2	-0.01
**Sex**						
Female	52.5	50	0.05	52.8	54.7	-0.04

Table S6.148. *Continued.* Selected baseline characteristics for US PharMetrics Plus for the short-term risk of bipolar disorders

Characteristic	Before propensity score matching			After propensity score matching		
	Targets, %	Comparators, %	Standardized difference	Targets, %	Comparators, %	Standardized difference
**Medical history (general)**						
Acute respiratory disease	40.9	18.4	0.51	37	41.9	-0.1
Attention deficit hyperactivity disorder	3.2	2.9	0.02	3.2	3.9	-0.04
Chronic liver disease	0.4	0.2	0.03	0.4	0.4	0
Chronic obstructive lung disease	2.3	1.2	0.08	1.9	2.3	-0.03
Crohn’s disease	0.3	0.2	0.01	0.3	0.4	-0.02
Dementia	1.4	0.4	0.11	1.2	1.6	-0.04
Depressive disorder	10.3	6.9	0.12	9.6	11.2	-0.05
Diabetes mellitus	12.2	5.9	0.22	10.4	9.2	0.04
Gastroesophageal reflux disease	11	5.9	0.19	10	10.6	-0.02
Gastrointestinal hemorrhage	1.6	0.8	0.06	1.4	1.6	-0.01
Human immunodeficiency virus infection	0.3	0.2	0.03	0.3	0.3	-0.01
Hyperlipidemia	22.7	14.9	0.2	20.7	19.1	0.04
Hypertensive disorder	27.4	16.4	0.27	24.7	23	0.04
Lesion of liver	0.5	0.2	0.04	0.4	0.5	-0.01
Obesity	12.4	5.9	0.23	10.7	10.1	0.02
Osteoarthritis	11.5	7	0.15	10.6	10.3	0.01
Pneumonia	10.6	1.1	0.41	4.9	3.4	0.08
Renal impairment	5	1.8	0.17	3.8	3.8	0
Rheumatoid arthritis	1.1	0.6	0.06	1	1	0
Schizophrenia	0.1	0.1	0.01	0.1	0.1	0
Ulcerative colitis	0.3	0.2	0.02	0.3	0.3	0
Urinary tract infectious disease	5.5	2.5	0.15	5	5.3	-0.01
Viral hepatitis C	0.2	0.1	0.02	0.2	0.2	-0.01

Table S6.148. *Continued.* Selected baseline characteristics for US PharMetrics Plus for the short-term risk of bipolar disorders

Characteristic	Before propensity score matching			After propensity score matching		
	Targets, %	Comparators, %	Standardized diff	Targets, %	Comparators, %	Standardized diff
**Medical history (cardiovascular disease)**						
Atrial fibrillation	2.4	1.2	0.09	2.1	2.1	0
Cerebrovascular disease	1.2	0.7	0.05	1.1	1.2	-0.01
Coronary arteriosclerosis	4	2.2	0.1	3.5	3.5	0
Heart disease	10.3	5.6	0.17	8.9	9.1	0
Heart failure	2.4	0.9	0.12	2	2.2	-0.01
Ischemic heart disease	2.5	1.1	0.11	2	2.1	0
Peripheral vascular disease	2.1	0.9	0.1	1.8	1.9	-0.01
Pulmonary embolism	0.6	0.2	0.06	0.4	0.5	-0.01
Venous thrombosis	0.8	0.4	0.05	0.7	0.8	-0.01
**Medical history (neoplasms)**						
Malignant lymphoma	0.3	0.2	0.03	0.3	0.3	0
Malignant neoplasm of anorectum	0.1	0.1	0.01	0.1	0.1	-0.01
Malignant neoplastic disease	3.9	3.1	0.04	3.6	3.9	-0.01
Malignant tumor of breast	0.7	0.6	0.01	0.6	0.7	-0.01
Malignant tumor of lung	0.2	0.1	0.02	0.2	0.3	-0.02
Malignant tumor of urinary bladder	0.1	0.1	0	0.1	0.1	-0.01
Primary malignant neoplasm of prostate	0.5	0.4	0.01	0.5	0.4	0.01
**Medication use**						
Agents acting on the renin-angiotensin system	11.9	8	0.13	10.7	9.7	0.04
Antibacterials for systemic use	39.7	25.1	0.32	36.6	38.7	-0.04
Antidepressants	12.7	9	0.12	12	13.7	-0.05
Antiepileptics	6.1	3.8	0.1	5.3	5.9	-0.03
Antiinflammatory and antirheumatic products	19.8	10.9	0.25	18.1	17.8	0.01
Antineoplastic agents	2.8	1.9	0.06	2.7	2.9	-0.01
Antipsoriatics	1.8	1.2	0.05	1.9	2	-0.01
Antithrombotic agents	4.9	2.5	0.12	4.2	4.3	-0.01

Table S6.148. *Continued.* Selected baseline characteristics for US PharMetrics Plus for the short-term risk of bipolar disorders

**Characteristic**	**Before propensity score matching**			**After propensity score matching**		
	**Targets, %**	**Comparators, %**	**Standardized difference**	**Targets, %**	**Comparators, %**	**Standardized difference**
**Medication use**						
Beta blocking agents	7	4.5	0.11	6.3	6.1	0.01
Calcium channel blockers	5.3	3.5	0.09	4.8	4.5	0.01
Diuretics	8.9	5.5	0.13	8	7.6	0.02
Drugs for acid-related disorders	12.9	7.5	0.18	11.8	12.4	-0.02
Drugs for obstructive airway diseases	24.6	14.1	0.27	22.4	23.6	-0.03
Drugs used in diabetes	7.1	3.8	0.15	6.2	5.4	0.03
Immunosuppressants	1.7	1.1	0.05	1.5	1.6	0
Lipid modifying agents	11.4	8	0.11	10.2	9.3	0.03
Opioids	13.1	7.8	0.17	12	12.9	-0.03
Psycholeptics	13.3	8.7	0.15	12.4	13.6	-0.04
Psychostimulants, agents used for ADHD and nootropics	3.6	2.7	0.05	3.5	4.1	-0.03

Table S6.149. Selected baseline characteristics for US PharMetrics Plus for the short-term risk of psychoses

Characteristic	Before propensity score matching			After propensity score matching		
	Targets, %	Comparators, %	Standardized difference	Targets, %	Comparators, %	Standardized difference
**Age group (years)**						
0-4	0.7	3.8	-0.21	0.8	2	-0.1
5-9	1.2	5.5	-0.24	1.4	2.6	-0.09
10-14	2.3	6.1	-0.19	2.5	3.5	-0.06
15-19	6.1	6.7	-0.03	6.6	7.2	-0.03
20-24	9.1	7.6	0.06	9.7	10.3	-0.02
25-29	7.2	6.9	0.01	7.6	8.3	-0.03
30-34	7.7	7.2	0.02	8	8.6	-0.02
35-39	8.2	7.4	0.03	8.4	8.4	0
40-44	8.8	7.3	0.05	8.8	8.3	0.02
45-49	9.9	7.4	0.09	9.7	8.5	0.04
50-54	10.8	8	0.1	10.5	8.9	0.06
55-59	10.7	8.5	0.07	10.2	8.7	0.05
60-64	9.1	8.4	0.02	8.6	7.5	0.04
65-69	3.2	4.1	-0.05	2.9	2.7	0.01
70-74	1.6	2	-0.03	1.4	1.4	0
75-79	1.1	1.3	-0.02	0.9	0.8	0.01
80-84	2.4	2	0.03	2	2.2	-0.01
**Sex**						
Female	52.5	50.1	0.05	52.9	54.9	-0.04

Table S6.149. *Continued.* Selected baseline characteristics for US PharMetrics Plus for the short-term risk of psychoses

Characteristic	Before propensity score matching			After propensity score matching		
	Targets, %	Comparators, %	Standardized difference	Targets, %	Comparators, %	Standardized difference
**Medical history (general)**						
Acute respiratory disease	40.9	18.5	0.5	37.1	42.4	-0.11
Attention deficit hyperactivity disorder	3.2	2.9	0.02	3.4	4.1	-0.04
Chronic liver disease	0.4	0.3	0.03	0.4	0.4	0
Chronic obstructive lung disease	2.3	1.2	0.09	1.9	2.3	-0.03
Crohn’s disease	0.3	0.2	0.02	0.3	0.4	-0.02
Dementia	1.4	0.4	0.11	1.1	1.5	-0.04
Depressive disorder	10.3	6.9	0.12	9.9	11.7	-0.06
Diabetes mellitus	12.2	5.9	0.22	10.4	9.2	0.04
Gastroesophageal reflux disease	11	5.8	0.19	10.1	10.7	-0.02
Gastrointestinal hemorrhage	1.6	0.8	0.07	1.4	1.6	-0.01
Human immunodeficiency virus infection	0.3	0.2	0.03	0.3	0.4	-0.01
Hyperlipidemia	22.7	14.6	0.21	20.8	19.2	0.04
Hypertensive disorder	27.4	16.3	0.27	24.7	23	0.04
Lesion of liver	0.5	0.3	0.03	0.4	0.5	-0.01
Obesity	12.4	5.7	0.23	10.8	10.2	0.02
Osteoarthritis	11.5	7	0.16	10.7	10.3	0.01
Pneumonia	10.6	1.1	0.41	5	3.5	0.07
Psoriasis	1.1	0.8	0.03	1	1	0
Renal impairment	5	1.9	0.17	3.8	3.9	-0.01
Rheumatoid arthritis	1.1	0.5	0.06	1	1	0
Ulcerative colitis	0.3	0.2	0.02	0.3	0.4	-0.01
Urinary tract infectious disease	5.5	2.5	0.15	5	5.4	-0.02
Viral hepatitis C	0.2	0.1	0.01	0.2	0.2	0

Table S6.149. *Continued.* Selected baseline characteristics for US PharMetrics Plus for the short-term risk of psychoses

Characteristic	Before propensity score matching			After propensity score matching		
	Targets, %	Comparators, %	Standardized difference	Targets, %	Comparators, %	Standardized difference
**Medical history (cardiovascular disease)**						
Atrial fibrillation	2.4	1.3	0.08	2.1	2.1	0
Cerebrovascular disease	1.2	0.7	0.06	1.1	1.3	-0.02
Coronary arteriosclerosis	4	2.2	0.1	3.5	3.5	0
Heart disease	10.3	5.6	0.17	8.9	9.1	-0.01
Heart failure	2.4	0.9	0.12	2	2.2	-0.01
Ischemic heart disease	2.5	1.1	0.1	2	2.1	-0.01
Peripheral vascular disease	2.1	0.9	0.1	1.7	1.9	-0.01
Pulmonary embolism	0.6	0.2	0.07	0.4	0.5	-0.01
Venous thrombosis	0.8	0.4	0.05	0.7	0.8	-0.01
**Medical history (neoplasms)**						
Malignant lymphoma	0.3	0.2	0.03	0.3	0.3	-0.01
Malignant neoplasm of anorectum	0.1	0.1	0.01	0.1	0.1	-0.01
Malignant neoplastic disease	3.9	3.1	0.04	3.6	4	-0.02
Malignant tumor of breast	0.7	0.6	0.01	0.6	0.7	-0.01
Malignant tumor of colon	0.2	0.1	0.01	0.2	0.2	0
Malignant tumor of lung	0.2	0.1	0.02	0.2	0.3	-0.02
Malignant tumor of urinary bladder	0.1	0.1	0	0.1	0.1	-0.01
Primary malignant neoplasm of prostate	0.5	0.4	0.02	0.4	0.4	0.01
**Medication use**						
Agents acting on the renin-angiotensin system	11.9	8	0.13	10.7	9.6	0.04
Antibacterials for systemic use	39.7	25.1	0.32	36.7	38.7	-0.04
Antidepressants	12.7	9.1	0.12	12.2	13.8	-0.05
Antiepileptics	6.1	3.8	0.1	5.5	6.2	-0.03
Antiinflammatory and antirheumatic products	19.8	10.9	0.25	18.2	17.9	0.01
Antineoplastic agents	2.8	1.9	0.06	2.7	3	-0.02

Table S6.149. *Continued.* Selected baseline characteristics for US PharMetrics Plus for the short-term risk of psychoses

Characteristic	Before propensity score matching			After propensity score matching		
	Targets, %	Comparators, %	Standardized difference	Targets, %	Comparators, %	Standardized difference
**Medication use**						
Antipsoriatics	1.8	1.2	0.05	1.9	2.1	-0.01
Antithrombotic agents	4.9	2.5	0.13	4.2	4.4	-0.01
Beta blocking agents	7	4.5	0.11	6.3	6.2	0.01
Calcium channel blockers	5.3	3.5	0.09	4.7	4.5	0.01
Diuretics	8.9	5.5	0.13	8	7.5	0.02
Drugs for acid-related disorders	12.9	7.4	0.18	11.9	12.4	-0.02
Drugs for obstructive airway diseases	24.6	14.2	0.26	22.5	23.7	-0.03
Drugs used in diabetes	7.1	3.7	0.15	6.2	5.4	0.03
Immunosuppressants	1.7	1	0.05	1.5	1.6	-0.01
Lipid modifying agents	11.4	7.9	0.12	10.3	9.3	0.03
Opioids	13.1	7.8	0.18	12.1	13	-0.03
Psycholeptics	13.3	8.6	0.15	12.6	13.9	-0.04
Psychostimulants, agents used for ADHD and nootropics	3.6	2.7	0.05	3.5	4.2	-0.04

Table S6.150. Selected baseline characteristics for US PharMetrics Plus for the short-term risk of personality disorders

Characteristic	Before propensity score matching			After propensity score matching		
	Targets, %	Comparators, %	Standardized difference	Targets, %	Comparators, %	Standardized difference
**Age group (years)**						
0-4	0.7	3.8	-0.21	0.8	1.9	-0.1
45421	1.2	5.4	-0.24	1.4	2.5	-0.08
45579	2.3	6.1	-0.19	2.5	3.5	-0.06
15-19	6.1	6.6	-0.02	6.6	7.2	-0.02
20-24	9.1	7.5	0.06	9.7	10	-0.01
25-29	7.2	7	0.01	7.5	8.4	-0.03
30-34	7.7	7.2	0.02	8	8.7	-0.03
35-39	8.2	7.5	0.03	8.4	8.5	0
40-44	8.8	7.4	0.05	8.8	8.4	0.02
45-49	9.9	7.4	0.09	9.7	8.5	0.04
50-54	10.8	7.9	0.1	10.5	8.9	0.05
55-59	10.7	8.6	0.07	10.2	8.8	0.05
60-64	9.1	8.4	0.02	8.6	7.6	0.04
65-69	3.2	4.1	-0.05	3	2.7	0.02
75-79	1.1	1.2	-0.02	0.9	0.8	0.01
80-84	2.4	1.9	0.04	2.1	2.3	-0.01
**Sex**						
Female	52.5	50.2	0.05	52.9	55.1	-0.04

Table S6.150. *Continued.* Selected baseline characteristics for US PharMetrics Plus for the short-term risk of personality disorders

Characteristic	Before propensity score matching			After propensity score matching		
	Targets, %	Comparators, %	Standardized difference	Targets, %	Comparators, %	Standardized difference
**Medical history (general)**						
Acute respiratory disease	40.9	18.5	0.5	37	42.2	-0.11
Attention deficit hyperactivity disorder	3.2	2.8	0.02	3.3	4.1	-0.04
Chronic liver disease	0.4	0.2	0.03	0.4	0.4	-0.01
Chronic obstructive lung disease	2.3	1.2	0.09	1.9	2.4	-0.03
Crohn's disease	0.3	0.2	0.01	0.3	0.4	-0.02
Dementia	1.4	0.4	0.11	1.2	1.7	-0.04
Depressive disorder	10.3	6.9	0.12	9.9	11.9	-0.06
Diabetes mellitus	12.2	5.9	0.22	10.5	9.2	0.04
Gastroesophageal reflux disease	11	5.7	0.19	10.1	10.8	-0.02
Gastrointestinal hemorrhage	1.6	0.8	0.07	1.4	1.7	-0.02
Human immunodeficiency virus infection	0.3	0.2	0.03	0.3	0.4	-0.01
Hyperlipidemia	22.7	14.7	0.21	20.8	19.3	0.04
Hypertensive disorder	27.4	16.4	0.27	24.8	23.1	0.04
Lesion of liver	0.5	0.2	0.04	0.4	0.5	-0.01
Obesity	12.4	5.8	0.23	10.8	10.2	0.02
Osteoarthritis	11.5	7	0.16	10.7	10.6	0
Pneumonia	10.6	1.1	0.41	5	3.5	0.07
Psoriasis	1.1	0.8	0.03	1	1.1	0
Renal impairment	5	1.8	0.17	3.8	3.9	0
Rheumatoid arthritis	1.1	0.6	0.06	1	1	0
Schizophrenia	0.1	0.1	0.01	0.1	0.1	-0.01
Ulcerative colitis	0.3	0.2	0.02	0.3	0.3	0
Urinary tract infectious disease	5.5	2.5	0.15	5.1	5.6	-0.02
Viral hepatitis C	0.2	0.1	0.02	0.2	0.2	-0.01

Table S6.150. *Continued.* Selected baseline characteristics for US PharMetrics Plus for the short-term risk of personality disorders

Characteristic	Before propensity score matching			After propensity score matching		
	Targets, %	Comparators, %	Standardized difference	Targets, %	Comparators, %	Standardized difference
**Medical history (cardiovascular disease)**						
Atrial fibrillation	2.4	1.2	0.09	2.1	2.1	0
Cerebrovascular disease	1.2	0.7	0.06	1.1	1.2	-0.01
Coronary arteriosclerosis	4	2.3	0.1	3.5	3.5	0
Heart disease	10.3	5.6	0.17	9	9.2	-0.01
Heart failure	2.4	0.9	0.12	2	2.2	-0.01
Ischemic heart disease	2.5	1.1	0.1	2	2.1	0
Peripheral vascular disease	2.1	0.9	0.1	1.8	1.9	-0.01
Pulmonary embolism	0.6	0.2	0.06	0.4	0.5	-0.01
Venous thrombosis	0.8	0.4	0.05	0.7	0.8	0
**Medical history (neoplasms)**						
Malignant lymphoma	0.3	0.2	0.02	0.3	0.3	-0.01
Malignant neoplasm of anorectum	0.1	0.1	0.01	0.1	0.1	-0.01
Malignant neoplastic disease	3.9	3.1	0.04	3.7	3.9	-0.02
Malignant tumor of breast	0.7	0.6	0.01	0.6	0.7	-0.01
Malignant tumor of colon	0.2	0.1	0.02	0.2	0.2	0
Malignant tumor of lung	0.2	0.1	0.02	0.2	0.3	-0.02
Malignant tumor of urinary bladder	0.1	0.1	0	0.1	0.1	-0.01
Primary malignant neoplasm of prostate	0.5	0.4	0.02	0.5	0.4	0.01
**Medication use**						
Agents acting on the renin-angiotensin system	11.9	8.1	0.13	10.7	9.6	0.04
Antibacterials for systemic use	39.7	25.1	0.31	36.7	38.7	-0.04
Antidepressants	12.7	9.1	0.12	12.2	14.2	-0.06
Antiepileptics	6.1	3.9	0.1	5.6	6.2	-0.03
Antiinflammatory and antirheumatic products	19.8	10.9	0.25	18.2	17.9	0.01
Antineoplastic agents	2.8	1.9	0.06	2.7	3	-0.02

Table S6.150. *Continued.* Selected baseline characteristics for US PharMetrics Plus for the short-term risk of personality disorders

Characteristic	Before propensity score matching			After propensity score matching		
	Targets, %	Comparators, %	Standardized difference	Targets, %	Comparators, %	Standardized difference
**Medication use**						
Antipsoriatics	1.8	1.3	0.05	1.9	2	-0.01
Antithrombotic agents	4.9	2.6	0.12	4.2	4.3	0
Beta blocking agents	7	4.6	0.1	6.3	6.2	0
Calcium channel blockers	5.3	3.5	0.09	4.8	4.5	0.01
Diuretics	8.9	5.5	0.13	8	7.5	0.02
Drugs for acid-related disorders	12.9	7.5	0.18	11.9	12.5	-0.02
Drugs for obstructive airway diseases	24.6	14.2	0.26	22.5	23.7	-0.03
Drugs used in diabetes	7.1	3.9	0.14	6.2	5.4	0.03
Immunosuppressants	1.7	1.1	0.05	1.5	1.6	0
Lipid modifying agents	11.4	8	0.12	10.3	9.4	0.03
Opioids	13.1	7.8	0.17	12.1	13	-0.03
Psycholeptics	13.3	8.6	0.15	12.7	14.1	-0.04
Psychostimulants, agents used for ADHD and nootropics	3.6	2.6	0.05	3.5	4.3	-0.04

Table S6.151. Selected baseline characteristics for US PharMetrics Plus for the short-term risk of self-harm and suicide

Characteristic	Before propensity score matching			After propensity score matching		
	Targets, %	Comparators, %	Standardized difference	Targets, %	Comparators, %	Standardized difference
**Age group (years)**						
0-4	0.7	3.8	-0.21	0.8	2	-0.1
5-9	1.2	5.5	-0.24	1.4	2.6	-0.09
10-14	2.3	6	-0.19	2.5	3.5	-0.06
15-19	6.1	6.5	-0.02	6.6	7.2	-0.03
20-24	9.1	7.6	0.06	9.7	10.2	-0.01
25-29	7.2	6.9	0.01	7.5	8.4	-0.03
30-34	7.7	7.2	0.02	8	8.5	-0.02
35-39	8.2	7.5	0.03	8.4	8.3	0
40-44	8.8	7.3	0.06	8.8	8.5	0.01
45-49	9.9	7.4	0.09	9.7	8.3	0.05
50-54	10.8	8.1	0.09	10.5	8.9	0.05
55-59	10.7	8.5	0.07	10.2	8.8	0.05
60-64	9.1	8.5	0.02	8.6	7.4	0.04
65-69	3.2	4.2	-0.06	3	2.8	0.01
70-74	1.6	1.9	-0.03	1.4	1.4	0
75-79	1.1	1.3	-0.02	0.9	0.8	0.01
80-84	2.4	1.9	0.03	2.1	2.3	-0.01
**Sex**						
Female	52.5	50	0.05	52.9	54.8	-0.04

Table S6.151. *Continued.* Selected baseline characteristics for US PharMetrics Plus for the short-term risk of self-harm and suicide

Characteristic	Before propensity score matching			After propensity score matching		
	Targets, %	Comparators, %	Standardized difference	Targets, %	Comparators, %	Standardized difference
**Medical history (general)**						
Acute respiratory disease	40.9	18.5	0.51	37.1	42.3	-0.11
Attention deficit hyperactivity disorder	3.2	2.8	0.02	3.4	4	-0.04
Chronic liver disease	0.4	0.2	0.04	0.4	0.4	-0.01
Chronic obstructive lung disease	2.3	1.2	0.09	1.9	2.4	-0.03
Crohn’s disease	0.3	0.2	0.02	0.3	0.4	-0.02
Dementia	1.4	0.4	0.11	1.2	1.6	-0.04
Depressive disorder	10.3	6.8	0.12	10	11.7	-0.06
Diabetes mellitus	12.2	5.9	0.22	10.5	9.3	0.04
Gastroesophageal reflux disease	11	5.7	0.19	10.2	10.6	-0.01
Gastrointestinal hemorrhage	1.6	0.8	0.07	1.5	1.6	-0.01
Human immunodeficiency virus infection	0.3	0.2	0.03	0.3	0.4	-0.01
Hyperlipidemia	22.7	14.9	0.2	20.8	19.4	0.04
Hypertensive disorder	27.4	16.4	0.27	24.8	23.2	0.04
Lesion of liver	0.5	0.2	0.04	0.4	0.5	-0.01
Obesity	12.4	5.8	0.23	10.8	10.3	0.02
Osteoarthritis	11.5	7	0.16	10.7	10.5	0.01
Pneumonia	10.6	1.1	0.41	5	3.5	0.08
Psoriasis	1.1	0.8	0.03	1	1	0
Renal impairment	5	1.9	0.17	3.8	3.9	0
Rheumatoid arthritis	1.1	0.5	0.06	1	0.9	0.01
Ulcerative colitis	0.3	0.2	0.02	0.3	0.4	-0.01
Urinary tract infectious disease	5.5	2.5	0.15	5.1	5.6	-0.02
Viral hepatitis C	0.2	0.1	0.02	0.2	0.2	-0.01

Table S6.151. *Continued.* Selected baseline characteristics for US PharMetrics Plus for the short-term risk of self-harm and suicide

Characteristic	Before propensity score matching			After propensity score matching		
	Targets, %	Comparators, %	Standardized difference	Targets, %	Comparators, %	Standardized difference
**Medical history (cardiovascular disease)**						
Atrial fibrillation	2.4	1.2	0.09	2.1	2.1	0
Cerebrovascular disease	1.2	0.7	0.06	1.1	1.2	-0.01
Coronary arteriosclerosis	4	2.3	0.1	3.5	3.6	0
Heart disease	10.3	5.7	0.17	9	9.2	0
Heart failure	2.4	0.9	0.12	2	2.2	-0.01
Ischemic heart disease	2.5	1.1	0.1	2	2.1	-0.01
Peripheral vascular disease	2.1	0.9	0.1	1.8	2	-0.01
Pulmonary embolism	0.6	0.2	0.07	0.4	0.5	0
Venous thrombosis	0.8	0.4	0.06	0.7	0.7	0
**Medical history (neoplasms)**						
Malignant lymphoma	0.3	0.2	0.03	0.3	0.3	-0.01
Malignant neoplasm of anorectum	0.1	0.1	0	0.1	0.1	-0.01
Malignant neoplastic disease	3.9	3.1	0.04	3.7	4	-0.02
Malignant tumor of breast	0.7	0.6	0.01	0.6	0.8	-0.01
Malignant tumor of colon	0.2	0.1	0.01	0.2	0.2	0
Malignant tumor of lung	0.2	0.1	0.01	0.2	0.3	-0.02
Malignant tumor of urinary bladder	0.1	0.1	0.01	0.1	0.1	-0.01
Primary malignant neoplasm of prostate	0.5	0.4	0.01	0.5	0.4	0.01
**Medication use**						
Agents acting on the renin-angiotensin system	11.9	8.1	0.13	10.7	9.8	0.03
Antibacterials for systemic use	39.7	25.2	0.31	36.7	38.8	-0.04
Antidepressants	12.7	9.1	0.12	12.3	14	-0.05
Antiepileptics	6.1	3.9	0.1	5.6	6.3	-0.03
Antiinflammatory and antirheumatic products	19.8	10.9	0.25	18.2	17.9	0.01
Antineoplastic agents	2.8	1.9	0.06	2.7	2.9	-0.01

Table S6.151. *Continued.* Selected baseline characteristics for US PharMetrics Plus for the short-term risk of self-harm and suicide

Characteristic	Before propensity score matching			After propensity score matching		
	Targets, %	Comparators, %	Standardized difference	Targets, %	Comparators, %	Standardized difference
**Medication use**						
Antipsoriatics	1.8	1.2	0.05	1.9	2	-0.01
Antithrombotic agents	4.9	2.5	0.12	4.2	4.3	0
Beta blocking agents	7	4.6	0.1	6.4	6.2	0
Calcium channel blockers	5.3	3.5	0.09	4.8	4.5	0.01
Diuretics	8.9	5.6	0.13	8	7.5	0.02
Drugs for acid-related disorders	12.9	7.4	0.18	11.9	12.5	-0.02
Drugs for obstructive airway diseases	24.6	14.2	0.26	22.5	23.7	-0.03
Drugs used in diabetes	7.1	3.8	0.15	6.2	5.4	0.03
Immunosuppressants	1.7	1	0.06	1.5	1.6	-0.01
Lipid modifying agents	11.4	8	0.12	10.3	9.4	0.03
Opioids	13.1	7.8	0.18	12.2	13	-0.03
Psycholeptics	13.3	8.7	0.15	12.7	14	-0.04
Psychostimulants, agents used for ADHD and nootropics	3.6	2.6	0.05	3.6	4.3	-0.04

Table S6.152. Selected baseline characteristics for US PharMetrics Plus for the short-term risk of sleep disorders

**Characteristic**	**Before propensity score matching**			**After propensity score matching**		
	**Targets, %**	**Comparators, %**	**Standardized difference**	**Targets, %**	**Comparators, %**	**Standardized difference**
**Age group (years)**						
0-4	0.7	3.7	-0.2	0.9	2.1	-0.1
5-9	1.2	5.5	-0.24	1.5	2.8	-0.09
10-14	2.3	6.2	-0.19	2.7	3.8	-0.06
15-19	6.1	6.6	-0.02	7.1	7.7	-0.02
20-24	9.1	7.5	0.06	10.4	10.9	-0.01
25-29	7.2	7.1	0	8	8.9	-0.03
30-34	7.7	7.1	0.02	8.3	8.9	-0.02
35-39	8.2	7.5	0.03	8.6	8.4	0.01
40-44	8.8	7.4	0.05	8.8	8.3	0.02
45-49	9.9	7.3	0.09	9.3	8.1	0.04
50-54	10.8	7.9	0.1	10.1	8.6	0.05
55-59	10.7	8.5	0.07	9.7	8.3	0.05
60-64	9.1	8.4	0.03	8	6.9	0.04
65-69	3.2	4.2	-0.06	2.7	2.4	0.02
70-74	1.6	2	-0.03	1.3	1.3	0
75-79	1.1	1.3	-0.02	0.8	0.7	0.01
80-84	2.4	1.9	0.04	1.8	1.9	-0.01
**Sex**						
Female	52.5	50.2	0.04	53.1	55.3	-0.05

Table S6.152. *Continued.* Selected baseline characteristics for US PharMetrics Plus for the short-term risk of sleep disorders

**Characteristic**	**Before propensity score matching**			**After propensity score matching**		
	**Targets, %**	**Comparators, %**	**Standardized difference**	**Targets, %**	**Comparators, %**	**Standardized difference**
**Medical history (general)**						
Acute respiratory disease	40.9	18.6	0.5	36.2	41.4	-0.11
Attention deficit hyperactivity disorder	3.2	2.9	0.02	3.2	3.8	-0.03
Chronic liver disease	0.4	0.2	0.03	0.3	0.3	0
Chronic obstructive lung disease	2.3	1.2	0.09	1.5	1.8	-0.03
Crohn’s disease	0.3	0.2	0.01	0.3	0.4	-0.02
Dementia	1.4	0.4	0.11	1	1.4	-0.04
Depressive disorder	10.3	6.8	0.12	8.3	9.8	-0.05
Diabetes mellitus	12.2	5.8	0.22	9.3	7.9	0.05
Gastroesophageal reflux disease	11	5.8	0.19	8.6	9	-0.02
Gastrointestinal hemorrhage	1.6	0.9	0.06	1.2	1.4	-0.02
Human immunodeficiency virus infection	0.3	0.2	0.03	0.3	0.4	-0.01
Hyperlipidemia	22.7	14.6	0.21	18.6	16.9	0.04
Hypertensive disorder	27.4	16.3	0.27	22	20.1	0.05
Lesion of liver	0.5	0.2	0.04	0.4	0.4	-0.01
Obesity	12.4	5.8	0.23	8.8	8.1	0.03
Osteoarthritis	11.5	7.1	0.15	9.1	8.9	0.01
Pneumonia	10.6	1.1	0.41	4.5	3.1	0.08
Psoriasis	1.1	0.8	0.02	0.9	1	0
Renal impairment	5	1.8	0.17	3.2	3.1	0
Rheumatoid arthritis	1.1	0.5	0.06	0.9	0.9	0
Schizophrenia	0.1	0.1	0.01	0.1	0.1	0
Ulcerative colitis	0.3	0.2	0.02	0.3	0.3	0
Urinary tract infectious disease	5.5	2.5	0.16	4.7	5	-0.01
Viral hepatitis C	0.2	0.1	0.02	0.1	0.2	0

Table S6.152. *Continued.* Selected baseline characteristics for US PharMetrics Plus for the short-term risk of sleep disorders

Characteristic	Before propensity score matching			After propensity score matching		
	Targets, %	Comparators, %	Standardized difference	Targets, %	Comparators, %	Standardized difference
**Medical history (cardiovascular disease)**						
Atrial fibrillation	2.4	1.2	0.09	1.6	1.6	0
Cerebrovascular disease	1.2	0.7	0.05	0.8	1	-0.02
Coronary arteriosclerosis	4	2.2	0.1	2.8	2.8	0
Heart disease	10.3	5.6	0.18	7.5	7.5	0
Heart failure	2.4	0.8	0.13	1.5	1.6	-0.01
Ischemic heart disease	2.5	1.1	0.11	1.6	1.7	0
Peripheral vascular disease	2.1	0.9	0.1	1.5	1.6	-0.01
Pulmonary embolism	0.6	0.2	0.07	0.3	0.4	-0.01
Venous thrombosis	0.8	0.4	0.06	0.6	0.6	0
**Medical history (neoplasms)**						
Malignant lymphoma	0.3	0.2	0.02	0.3	0.2	0
Malignant neoplasm of anorectum	0.1	0.1	0.01	0.1	0.1	-0.01
Malignant neoplastic disease	3.9	3.1	0.04	3.3	3.5	-0.01
Malignant tumor of breast	0.7	0.6	0.01	0.6	0.7	-0.01
Malignant tumor of colon	0.2	0.1	0.01	0.1	0.2	0
Malignant tumor of lung	0.2	0.1	0.02	0.1	0.2	-0.02
Malignant tumor of urinary bladder	0.1	0.1	0.01	0.1	0.1	0
Primary malignant neoplasm of prostate	0.5	0.4	0.01	0.4	0.3	0.01
**Medication use**						
Agents acting on the renin-angiotensin system	11.9	7.9	0.13	9.7	8.6	0.04
Antibacterials for systemic use	39.7	25.1	0.32	35.6	37.9	-0.05
Antidepressants	12.7	9.1	0.11	10.6	12.2	-0.05
Antiepileptics	6.1	3.9	0.1	4.6	5.2	-0.03
Antiinflammatory and antirheumatic products	19.8	11	0.24	17.1	16.9	0.01
Antineoplastic agents	2.8	1.9	0.06	2.4	2.7	-0.02

Table S6.152. *Continued.* Selected baseline characteristics for US PharMetrics Plus for the short-term risk of sleep disorders

Characteristic	Before propensity score matching			After propensity score matching		
	Targets, %	Comparators, %	Standardized difference	Targets, %	Comparators, %	Standardized difference
**Medication use**						
Antipsoriatics	1.8	1.3	0.04	1.9	2.1	-0.01
Antithrombotic agents	4.9	2.5	0.13	3.4	3.6	-0.01
Beta blocking agents	7	4.6	0.1	5.5	5.3	0.01
Calcium channel blockers	5.3	3.5	0.09	4.2	3.9	0.01
Diuretics	8.9	5.4	0.13	7.1	6.5	0.02
Drugs for acid-related disorders	12.9	7.4	0.18	10.5	11.2	-0.02
Drugs for obstructive airway diseases	24.6	14.3	0.26	21.1	22.3	-0.03
Drugs used in diabetes	7.1	3.8	0.14	5.6	4.8	0.04
Immunosuppressants	1.7	1.1	0.05	1.4	1.4	-0.01
Lipid modifying agents	11.4	7.9	0.12	9.3	8.3	0.04
Opioids	13.1	7.8	0.17	10.8	11.5	-0.03
Psycholeptics	13.3	8.6	0.15	10.6	11.8	-0.04
Psychostimulants, agents used for ADHD and nootropics	3.6	2.7	0.05	3.3	3.8	-0.03

Table S6.153. Selected baseline characteristics for US PharMetrics Plus for the short-term risk of dementia

Characteristic	Before propensity score matching			After propensity score matching		
	Targets, %	Comparators, %	Standardized difference	Targets, %	Comparators, %	Standardized difference
**Age group (years)**						
0-4	0.7	3.7	-0.2	0.8	2	-0.1
5-9	1.2	5.5	-0.24	1.4	2.6	-0.09
10-14	2.3	6.1	-0.19	2.6	3.4	-0.05
15-19	6.1	6.6	-0.02	6.6	7.3	-0.03
20-24	9.1	7.5	0.06	9.8	10.3	-0.01
25-29	7.2	6.9	0.01	7.6	8.5	-0.03
30-34	7.7	7.2	0.02	8	8.8	-0.03
35-39	8.2	7.4	0.03	8.4	8.4	0
40-44	8.8	7.2	0.06	8.9	8.5	0.01
45-49	9.9	7.4	0.09	9.8	8.6	0.04
50-54	10.8	8	0.1	10.6	9.2	0.05
55-59	10.7	8.6	0.07	10.3	8.9	0.05
60-64	9.1	8.5	0.02	8.6	7.5	0.04
65-69	3.2	4.2	-0.06	2.9	2.7	0.02
75-79	1.1	1.3	-0.03	0.8	0.7	0.01
80-84	2.4	1.9	0.03	1.4	1.3	0
**Sex**						
Female	52.5	50.2	0.05	52.8	55	-0.04

Table S6.153. *Continued.* Selected baseline characteristics for US PharMetrics Plus for the short-term risk of dementia

Characteristic	Before propensity score matching			After propensity score matching		
	Targets, %	Comparators, %	Standardized difference	Targets, %	Comparators, %	Standardized difference
**Medical history (general)**						
Acute respiratory disease	40.9	18.5	0.5	37.1	42.4	-0.11
Attention deficit hyperactivity disorder	3.2	2.9	0.02	3.4	4.1	-0.04
Chronic liver disease	0.4	0.2	0.03	0.4	0.5	-0.01
Chronic obstructive lung disease	2.3	1.2	0.08	1.8	2.1	-0.02
Crohn’s disease	0.3	0.2	0.02	0.3	0.4	-0.02
Depressive disorder	10.3	7	0.12	9.7	11.4	-0.06
Diabetes mellitus	12.2	5.9	0.22	10.2	8.9	0.05
Gastroesophageal reflux disease	11	5.8	0.19	10	10.5	-0.02
Gastrointestinal hemorrhage	1.6	0.8	0.07	1.4	1.6	-0.02
Human immunodeficiency virus infection	0.3	0.2	0.03	0.3	0.3	-0.01
Hyperlipidemia	22.7	14.8	0.2	20.5	18.7	0.04
Hypertensive disorder	27.4	16.4	0.27	24.2	22.3	0.04
Lesion of liver	0.5	0.2	0.04	0.4	0.5	-0.01
Obesity	12.4	5.8	0.23	10.8	10.2	0.02
Osteoarthritis	11.5	7	0.16	10.4	9.9	0.01
Pneumonia	10.6	1.1	0.41	4.8	3.2	0.08
Psoriasis	1.1	0.8	0.03	1	1.1	0
Renal impairment	5	1.8	0.17	3.4	3.4	0
Rheumatoid arthritis	1.1	0.6	0.06	1	0.9	0
Schizophrenia	0.1	0.1	0.01	0.1	0.1	-0.01
Ulcerative colitis	0.3	0.2	0.02	0.3	0.4	-0.01
Urinary tract infectious disease	5.5	2.4	0.16	4.8	5	-0.01
Viral hepatitis C	0.2	0.1	0.01	0.2	0.2	-0.01

Table S6.153. *Continued.* Selected baseline characteristics for US PharMetrics Plus for the short-term risk of dementia

**Characteristic**	**Before propensity score matching**			**After propensity score matching**		
	**Targets, %**	**Comparators, %**	**Standardized difference**	**Targets, %**	**Comparators, %**	**Standardized difference**
**Medical history (cardiovascular disease)**						
Atrial fibrillation	2.4	1.3	0.08	1.8	1.8	0.01
Cerebrovascular disease	1.2	0.7	0.05	0.9	1	-0.01
Coronary arteriosclerosis	4	2.3	0.1	3.3	3.1	0.01
Heart disease	10.3	5.7	0.17	8.5	8.4	0
Heart failure	2.4	0.9	0.12	1.8	1.8	0
Ischemic heart disease	2.5	1.1	0.1	1.9	2	0
Peripheral vascular disease	2.1	0.9	0.1	1.4	1.6	-0.01
Pulmonary embolism	0.6	0.2	0.06	0.4	0.5	-0.01
Venous thrombosis	0.8	0.4	0.06	0.7	0.7	0
**Medical history (neoplasms)**						
Malignant lymphoma	0.3	0.2	0.03	0.3	0.3	-0.01
Malignant neoplasm of anorectum	0.1	0.1	0.01	0.1	0.1	-0.01
Malignant neoplastic disease	3.9	3.1	0.04	3.5	3.8	-0.02
Malignant tumor of breast	0.7	0.6	0.01	0.6	0.7	-0.02
Malignant tumor of colon	0.2	0.1	0.02	0.1	0.1	0
Malignant tumor of lung	0.2	0.1	0.01	0.2	0.3	-0.02
Malignant tumor of urinary bladder	0.1	0.1	0	0.1	0.1	0
Primary malignant neoplasm of prostate	0.5	0.4	0.01	0.4	0.3	0.01
**Medication use**						
Agents acting on the renin-angiotensin system	11.9	8	0.13	10.7	9.5	0.04
Antibacterials for systemic use	39.7	25.2	0.31	36.8	39	-0.04
Antidepressants	12.7	9.1	0.11	12.2	14	-0.05
Antiepileptics	6.1	3.9	0.1	5.5	6.2	-0.03
Antiinflammatory and antirheumatic products	19.8	10.8	0.25	18.3	18	0.01
Antineoplastic agents	2.8	2	0.06	2.7	2.9	-0.01

Table S6.153. *Continued.* Selected baseline characteristics for US PharMetrics Plus for the short-term risk of dementia

Characteristic	Before propensity score matching			After propensity score matching		
	Targets, %	Comparators, %	Standardized difference	Targets, %	Comparators, %	Standardized difference
**Medication use**						
Antipsoriatics	1.8	1.2	0.05	1.9	2.1	-0.01
Antithrombotic agents	4.9	2.5	0.12	4.1	4.2	-0.01
Beta blocking agents	7	4.6	0.1	6.2	6.1	0
Calcium channel blockers	5.3	3.5	0.09	4.7	4.4	0.01
Diuretics	8.9	5.4	0.14	7.9	7.4	0.02
Drugs for acid-related disorders	12.9	7.5	0.18	11.8	12.4	-0.02
Drugs for obstructive airway diseases	24.6	14.4	0.26	22.5	23.8	-0.03
Drugs used in diabetes	7.1	3.8	0.15	6.1	5.3	0.04
Immunosuppressants	1.7	1.1	0.05	1.5	1.6	-0.01
Lipid modifying agents	11.4	7.9	0.12	10.2	9.2	0.03
Opioids	13.1	7.7	0.18	12.1	13.1	-0.03
Psycholeptics	13.3	8.6	0.15	12.6	14	-0.04
Psychostimulants, agents used for ADHD and nootropics	3.6	2.7	0.05	3.6	4.3	-0.04

Table S6.154. Selected baseline characteristics for US PharMetrics Plus for the short-term risk of neurodevelopmental disorders

**Characteristic**	**Before propensity score matching**			**After propensity score matching**		
	**Targets, %**	**Comparators, %**	**Standardized difference**	**Targets, %**	**Comparators, %**	**Standardized difference**
**Age group (years)**						
0-4	0.7	3.7	-0.2	0.8	2	-0.1
5-9	1.2	5.5	-0.24	1.3	2.5	-0.09
10-14	2.3	6.1	-0.19	2.4	3.2	-0.05
15-19	6.1	6.7	-0.03	6.3	6.9	-0.02
20-24	9.1	7.5	0.06	9.5	9.8	-0.01
25-29	7.2	7	0.01	7.4	8.2	-0.03
30-34	7.7	7.3	0.02	7.9	8.6	-0.02
35-39	8.2	7.4	0.03	8.4	8.4	0
40-44	8.8	7.3	0.06	8.9	8.5	0.01
45-49	9.9	7.4	0.09	9.7	8.7	0.04
50-54	10.8	8	0.1	10.7	9.1	0.05
55-59	10.7	8.6	0.07	10.4	9	0.05
60-64	9.1	8.4	0.03	8.8	7.7	0.04
65-69	3.2	4.1	-0.05	3	2.8	0.01
70-74	1.6	2	-0.03	1.4	1.5	0
75-79	1.1	1.3	-0.02	0.9	0.9	0.01
80-84	2.4	1.8	0.04	2.1	2.3	-0.01
**Sex**						
Female	52.5	49.9	0.05	52.9	55.1	-0.04

Table S6.154. *Continued.* Selected baseline characteristics for US PharMetrics Plus for the short-term risk of neurodevelopmental disorders

Characteristic	Before propensity score matching			After propensity score matching		
	Targets, %	Comparators, %	Standardized difference	Targets, %	Comparators, %	Standardized difference
**Medical history (general)**						
Acute respiratory disease	40.9	18.4	0.51	36.8	42	-0.11
Chronic liver disease	0.4	0.2	0.03	0.4	0.4	0
Chronic obstructive lung disease	2.3	1.3	0.08	2	2.5	-0.03
Crohn’s disease	0.3	0.2	0.01	0.3	0.4	-0.02
Dementia	1.4	0.4	0.11	1.2	1.6	-0.04
Depressive disorder	10.3	6.9	0.12	9.4	10.9	-0.05
Diabetes mellitus	12.2	5.9	0.22	10.6	9.4	0.04
Gastroesophageal reflux disease	11	5.8	0.19	10.2	10.8	-0.02
Gastrointestinal hemorrhage	1.6	0.8	0.07	1.4	1.7	-0.02
Human immunodeficiency virus infection	0.3	0.2	0.03	0.3	0.4	-0.01
Hyperlipidemia	22.7	14.9	0.2	21.1	19.6	0.04
Hypertensive disorder	27.4	16.3	0.27	25.1	23.4	0.04
Lesion of liver	0.5	0.3	0.03	0.4	0.5	-0.01
Obesity	12.4	5.8	0.23	10.8	10.2	0.02
Osteoarthritis	11.5	7	0.15	10.8	10.6	0.01
Pneumonia	10.6	1.1	0.41	5	3.5	0.08
Psoriasis	1.1	0.8	0.03	1	1	0
Renal impairment	5	1.9	0.17	3.9	3.9	0
Rheumatoid arthritis	1.1	0.6	0.06	1	1	0
Schizophrenia	0.1	0.1	0.01	0.1	0.1	0
Ulcerative colitis	0.3	0.2	0.02	0.3	0.3	0
Urinary tract infectious disease	5.5	2.4	0.16	5	5.4	-0.02
Viral hepatitis C	0.2	0.1	0.02	0.2	0.2	-0.01

Table S6.154. *Continued.* Selected baseline characteristics for US PharMetrics Plus for the short-term risk of neurodevelopmental disorders

Characteristic	Before propensity score matching			After propensity score matching		
	Targets, %	Comparators, %	Standardized difference	Targets, %	Comparators, %	Standardized difference
**Medical history (cardiovascular disease)**						
Atrial fibrillation	2.4	1.2	0.09	2.1	2.1	0
Cerebrovascular disease	1.2	0.7	0.06	1	1.2	-0.02
Coronary arteriosclerosis	4	2.3	0.1	3.6	3.6	0
Heart disease	10.3	5.7	0.17	9.1	9.3	-0.01
Heart failure	2.4	0.9	0.12	2	2.2	-0.01
Ischemic heart disease	2.5	1.1	0.1	2.1	2.2	-0.01
Peripheral vascular disease	2.1	0.9	0.1	1.8	2	-0.01
Pulmonary embolism	0.6	0.2	0.06	0.4	0.4	0
Venous thrombosis	0.8	0.4	0.05	0.7	0.8	0
**Medical history (neoplasms)**						
Malignant lymphoma	0.3	0.2	0.02	0.3	0.3	-0.01
Malignant neoplasm of anorectum	0.1	0.1	0.01	0.1	0.1	-0.01
Malignant neoplastic disease	3.9	3.1	0.05	3.7	4.1	-0.02
Malignant tumor of breast	0.7	0.5	0.01	0.6	0.8	-0.01
Malignant tumor of colon	0.2	0.1	0.02	0.2	0.2	0
Malignant tumor of lung	0.2	0.1	0.01	0.2	0.2	-0.01
Malignant tumor of urinary bladder	0.1	0.1	0.01	0.1	0.1	-0.01
Primary malignant neoplasm of prostate	0.5	0.4	0.01	0.5	0.4	0.01
**Medication use**						
Agents acting on the renin-angiotensin system	11.9	8.1	0.13	10.9	9.8	0.04
Antibacterials for systemic use	39.7	25.1	0.32	36.4	38.4	-0.04
Antidepressants	12.7	9	0.12	11.7	13.5	-0.05
Antiepileptics	6.1	3.9	0.1	5.4	6.1	-0.03
Antiinflammatory and antirheumatic products	19.8	10.9	0.25	18.2	18	0
Antineoplastic agents	2.8	1.9	0.06	2.7	3	-0.02

Table S6.154. *Continued.* Selected baseline characteristics for US PharMetrics Plus for the short-term risk of neurodevelopmental disorders

**Characteristic**	**Before propensity score matching**			**After propensity score matching**		
	**Targets, %**	**Comparators, %**	**Standardized difference**	**Targets, %**	**Comparators, %**	**Standardized difference**
**Medication use**						
Antipsoriatics	1.8	1.2	0.05	1.8	2	-0.01
Antithrombotic agents	4.9	2.5	0.13	4.2	4.4	-0.01
Beta blocking agents	7	4.6	0.1	6.4	6.2	0
Calcium channel blockers	5.3	3.6	0.09	4.8	4.6	0.01
Diuretics	8.9	5.5	0.13	8.1	7.6	0.02
Drugs for acid-related disorders	12.9	7.5	0.18	11.9	12.5	-0.02
Drugs for obstructive airway diseases	24.6	14.2	0.26	22.4	23.6	-0.03
Drugs used in diabetes	7.1	3.8	0.14	6.3	5.5	0.03
Immunosuppressants	1.7	1.1	0.05	1.5	1.6	-0.01
Lipid modifying agents	11.4	8	0.11	10.5	9.6	0.03
Opioids	13.1	7.7	0.18	12.1	13	-0.03
Psycholeptics	13.3	8.6	0.15	12.3	13.7	-0.04
Psychostimulants, agents used for ADHD and nootropics	3.6	2.7	0.05	1.8	2.1	-0.02

Table S6.155. Selected baseline characteristics for US PharMetrics Plus for the short-term risk of any of psychiatric and neuropsychiatric disorder

**Characteristic**	**Before propensity score matching**			**After propensity score matching**		
	**Targets, %**	**Comparators, %**	**Standardized difference**	**Targets, %**	**Comparators, %**	**Standardized difference**
**Age group (years)**						
0-4	0.7	3.8	-0.21	1.1	2.8	-0.12
5-9	1.2	5.4	-0.24	1.7	3.3	-0.1
10-14	2.3	6.1	-0.19	3	4.1	-0.06
15-19	6.1	6.6	-0.02	7.2	8	-0.03
20-24	9.1	7.6	0.05	10.2	10.5	-0.01
25-29	7.2	7	0.01	7.9	8.8	-0.03
30-34	7.7	7.2	0.02	8.2	8.8	-0.02
35-39	8.2	7.5	0.03	8.4	8.2	0.01
40-44	8.8	7.3	0.06	8.6	8.1	0.02
45-49	9.9	7.3	0.1	9.4	8.1	0.04
50-54	10.8	8	0.1	10.3	8.6	0.06
55-59	10.7	8.5	0.07	10	8.3	0.06
60-64	9.1	8.5	0.02	8.2	7.1	0.04
65-69	3.2	4.1	-0.05	2.7	2.4	0.02
70-74	1.6	2	-0.03	1.2	1.1	0
75-79	1.1	1.3	-0.02	0.7	0.6	0.02
80-84	2.4	1.8	0.04	1.2	1.2	0
**Sex**						
Female	52.5	50.1	0.05	50	52.1	-0.04

Table S6.155. *Continued.* Selected baseline characteristics for US PharMetrics Plus for the short-term risk of any of psychiatric and neuropsychiatric disorder

Characteristic	Before propensity score matching			After propensity score matching		
	Targets, %	Comparators, %	Standardized difference	Targets, %	Comparators, %	Standardized difference
**Medical history (general)**						
Acute respiratory disease	40.9	18.6	0.5	34	39	-0.1
Chronic liver disease	0.4	0.2	0.03	0.2	0.2	0
Chronic obstructive lung disease	2.3	1.2	0.09	0.9	0.9	-0.01
Crohn’s disease	0.3	0.2	0.02	0.2	0.3	-0.02
Depressive disorder	10.3	6.9	0.12	0.3	0.1	0.05
Diabetes mellitus	12.2	6	0.22	8.9	7.3	0.06
Gastroesophageal reflux disease	11	5.8	0.19	6.7	6.8	-0.01
Gastrointestinal hemorrhage	1.6	0.8	0.07	0.9	1	-0.01
Human immunodeficiency virus infection	0.3	0.2	0.03	0.2	0.3	-0.01
Hyperlipidemia	22.7	14.8	0.2	17.1	15	0.06
Hypertensive disorder	27.4	16.3	0.27	20	17.3	0.07
Lesion of liver	0.5	0.3	0.04	0.2	0.3	0
Obesity	12.4	5.8	0.23	7.6	6.7	0.04
Osteoarthritis	11.5	7	0.15	7.8	7.4	0.02
Pneumonia	10.6	1.1	0.41	3.9	2.3	0.1
Psoriasis	1.1	0.8	0.03	0.9	0.9	0
Renal impairment	5	1.9	0.17	2.6	2.3	0.02
Rheumatoid arthritis	1.1	0.5	0.06	0.7	0.7	0
Ulcerative colitis	0.3	0.2	0.03	0.3	0.3	0
Urinary tract infectious disease	5.5	2.5	0.15	3.7	3.9	-0.01

Table S6.155. *Continued.* Selected baseline characteristics for US PharMetrics Plus for the short-term risk of any of psychiatric and neuropsychiatric disorder

Characteristic	Before propensity score matching			After propensity score matching		
	Targets, %	Comparators, %	Standardized diff	Targets, %	Comparators, %	Standardized diff
**Medical history (cardiovascular disease)**						
Atrial fibrillation	2.4	1.3	0.08	1.2	1.2	0
Cerebrovascular disease	1.2	0.7	0.05	0.6	0.6	0
Coronary arteriosclerosis	4	2.2	0.1	2.3	2.2	0.01
Heart disease	10.3	5.7	0.17	6.2	5.9	0.01
Heart failure	2.4	0.9	0.12	1	1	0
Ischemic heart disease	2.5	1.1	0.11	1.3	1.2	0.01
Pulmonary embolism	0.6	0.2	0.07	0.3	0.2	0
Venous thrombosis	0.8	0.4	0.05	0.5	0.5	0
**Medical history (neoplasms)**						
Malignant lymphoma	0.3	0.2	0.03	0.2	0.2	0
Malignant neoplasm of anorectum	0.1	0.1	0	0.1	0.1	-0.01
Malignant neoplastic disease	3.9	3.1	0.04	2.9	3.1	-0.01
Malignant tumor of breast	0.7	0.6	0.01	0.5	0.6	-0.01
Malignant tumor of colon	0.2	0.1	0.01	0.1	0.1	0
Malignant tumor of lung	0.2	0.1	0.02	0.1	0.2	-0.01
Malignant tumor of urinary bladder	0.1	0.1	0	0.1	0.1	0
Primary malignant neoplasm of prostate	0.5	0.4	0.02	0.4	0.3	0.01
**Medication use**						
Agents acting on the renin-angiotensin system	11.9	8.1	0.13	9.3	7.9	0.05
Antibacterials for systemic use	39.7	25.2	0.31	33.3	34.9	-0.03
Antidepressants	12.7	9.1	0.12	4.6	4.9	-0.01
Antiepileptics	6.1	3.8	0.1	2.9	2.9	0
Antiinflammatory and antirheumatic products	19.8	10.8	0.25	15.3	14.5	0.02
Antineoplastic agents	2.8	1.9	0.06	2.1	2.3	-0.01
Antipsoriatics	1.8	1.2	0.05	1.7	1.9	-0.01

Table S6.155. *Continued.* Selected baseline characteristics for US PharMetrics Plus for the short-term risk of any of psychiatric and neuropsychiatric disorder

**Characteristic**	**Before propensity score matching**			**After propensity score matching**		
	**Targets, %**	**Comparators, %**	**Standardized difference**	**Targets, %**	**Comparators, %**	**Standardized difference**
**Medical history (cardiovascular disease)**						
Antithrombotic agents	4.9	2.5	0.13	2.8	2.7	0.01
Beta blocking agents	7	4.7	0.1	4.6	4.2	0.02
Calcium channel blockers	5.3	3.5	0.09	3.9	3.4	0.03
Diuretics	8.9	5.5	0.13	6.5	5.7	0.03
Drugs for acid-related disorders	12.9	7.5	0.18	8.8	9	0
Drugs for obstructive airway diseases	24.6	14.1	0.27	19.3	20	-0.02
Drugs used in diabetes	7.1	3.9	0.14	5.3	4.4	0.04
Immunosuppressants	1.7	1	0.05	1.2	1.3	-0.01
Lipid modifying agents	11.4	8	0.12	8.8	7.5	0.05
Opioids	13.1	7.8	0.18	9	9.1	0
Psycholeptics	13.3	8.6	0.15	6.7	6.8	0
Psychostimulants, agents used for ADHD and nootropics	3.6	2.7	0.05	1.3	1.4	-0.01

Table S6.156. Selected baseline characteristics for US PharMetrics Plus for the medium-term risk of depression

Characteristic	Before propensity score matching			After propensity score matching		
	**Targets, %**	**Comparators, %**	**Standardized difference**	**Targets, %**	**Comparators, %**	**Standardized difference**
**Age group (years)**						
0-4	0.7	3.7	-0.2	0.9	2.4	-0.11
5-9	1.2	5.5	-0.24	1.6	3.1	-0.1
10-14	2.3	6	-0.19	2.9	4	-0.06
15-19	6.1	6.6	-0.02	7	7.5	-0.02
20-24	9.1	7.5	0.06	10	10.2	-0.01
25-29	7.2	6.9	0.01	7	7.4	-0.02
30-34	7.7	7.2	0.02	7.9	8.4	-0.02
35-39	8.2	7.4	0.03	8.4	8.4	0
40-44	8.8	7.4	0.05	8.8	8.4	0.01
45-49	9.9	7.5	0.09	9.8	8.3	0.05
50-54	10.8	8	0.1	10.9	9.2	0.06
55-59	10.7	8.6	0.07	10.5	9.3	0.04
60-64	9.1	8.4	0.03	8.6	7.4	0.04
65-69	3.2	4.2	-0.05	2.6	2.3	0.02
70-74	1.6	2	-0.03	1.2	1.3	0
75-79	1.1	1.3	-0.02	0.7	0.7	0
80-84	2.4	1.9	0.04	1.3	1.6	-0.02
**Sex**						
Female	52.5	50.3	0.04	50.2	51.7	-0.03

Table S6.156. *Continued.* Selected baseline characteristics for US PharMetrics Plus for the medium-term risk of depression

Characteristic	Before propensity score matching			After propensity score matching		
	Targets, %	Comparators, %	Standardized difference	Targets, %	Comparators, %	Standardized difference
**Medical history (general)**						
Acute respiratory disease	40.9	18.6	0.5	35.3	40.4	-0.1
Attention deficit hyperactivity disorder	3.2	3	0.01	2.7	3.1	-0.03
Chronic liver disease	0.4	0.2	0.03	0.3	0.3	0
Chronic obstructive lung disease	2.3	1.2	0.09	1.3	1.6	-0.03
Crohn’s disease	0.3	0.2	0.01	0.2	0.4	-0.02
Dementia	1.4	0.4	0.11	0.5	0.7	-0.03
Diabetes mellitus	12.2	5.9	0.22	9.4	8.1	0.04
Gastroesophageal reflux disease	11	5.9	0.19	8.6	8.9	-0.01
Gastrointestinal hemorrhage	1.6	0.8	0.07	1.2	1.3	-0.01
Human immunodeficiency virus infection	0.3	0.2	0.02	0.3	0.3	-0.01
Hyperlipidemia	22.7	14.8	0.2	19.3	17.7	0.04
Hypertensive disorder	27.4	16.3	0.27	22.7	21.1	0.04
Lesion of liver	0.5	0.2	0.04	0.3	0.3	0
Obesity	12.4	5.8	0.23	9.5	8.6	0.03
Osteoarthritis	11.5	7	0.15	9.4	9.1	0.01
Pneumonia	10.6	1.1	0.41	3.9	2.7	0.07
Psoriasis	1.1	0.8	0.03	1	0.9	0.01
Renal impairment	5	1.8	0.18	2.8	2.7	0
Rheumatoid arthritis	1.1	0.6	0.06	0.9	0.8	0.01
Ulcerative colitis	0.3	0.2	0.02	0.3	0.3	0
Urinary tract infectious disease	5.5	2.5	0.15	4	4.4	-0.02
Viral hepatitis C	0.2	0.1	0.01	0.1	0.1	0

Table S6.156. *Continued.* Selected baseline characteristics for US PharMetrics Plus for the medium-term risk of depression

Characteristic	Before propensity score matching			After propensity score matching		
	Targets, %	Comparators, %	Standardized diff	Targets, %	Comparators, %	Standardized diff
**Medical history (cardiovascular disease)**						
Atrial fibrillation	2.4	1.3	0.08	1.6	1.6	0
Cerebrovascular disease	1.2	0.7	0.05	0.8	0.9	0
Coronary arteriosclerosis	4	2.2	0.1	2.9	2.9	0
Heart disease	10.3	5.7	0.17	7.5	7.7	-0.01
Heart failure	2.4	0.9	0.12	1.3	1.5	-0.02
Ischemic heart disease	2.5	1.1	0.11	1.6	1.6	0
Peripheral vascular disease	2.1	0.9	0.1	1.2	1.3	-0.01
Pulmonary embolism	0.6	0.2	0.06	0.3	0.3	-0.01
Venous thrombosis	0.8	0.4	0.05	0.6	0.6	0
**Medical history (neoplasms)**						
Malignant lymphoma	0.3	0.2	0.02	0.2	0.3	0
Malignant neoplasm of anorectum	0.1	0.1	0	0.1	0.1	-0.01
Malignant neoplastic disease	3.9	3.1	0.04	3.2	3.4	-0.01
Malignant tumor of breast	0.7	0.6	0.01	0.5	0.6	-0.01
Malignant tumor of colon	0.2	0.1	0.01	0.1	0.1	0
Malignant tumor of lung	0.2	0.1	0.02	0.1	0.2	-0.01
Malignant tumor of urinary bladder	0.1	0.1	0.01	0.1	0.1	-0.01
Primary malignant neoplasm of prostate	0.5	0.4	0.01	0.4	0.4	0.01
**Medication use**						
Agents acting on the renin-angiotensin system	11.9	8	0.13	10	9	0.03
Antibacterials for systemic use	39.7	25.2	0.31	34.6	36.2	-0.03
Antidepressants	12.7	9.1	0.11	7.5	8.1	-0.02
Antiepileptics	6.1	3.9	0.1	4	4.2	-0.01
Antiinflammatory and antirheumatic products	19.8	10.8	0.25	16.5	15.8	0.02
Antineoplastic agents	2.8	1.9	0.06	2.3	2.5	-0.01
Antipsoriatics	1.8	1.2	0.05	1.8	1.8	0

Table S6.156. *Continued.* Selected baseline characteristics for US PharMetrics Plus for the medium-term risk of depression

Characteristic	Before propensity score matching			After propensity score matching		
	Targets, %	Comparators, %	Standardized difference	Targets, %	Comparators, %	Standardized difference
**Medication use**						
Antithrombotic agents	4.9	2.4	0.13	3.5	3.5	0
Beta blocking agents	7	4.6	0.1	5.4	5.1	0.01
Calcium channel blockers	5.3	3.5	0.09	4.3	4	0.01
Diuretics	8.9	5.5	0.13	7.2	6.7	0.02
Drugs for acid-related disorders	12.9	7.5	0.18	10.3	10.7	-0.01
Drugs for obstructive airway diseases	24.6	14.1	0.27	20.9	21.6	-0.02
Drugs used in diabetes	7.1	3.8	0.15	5.5	4.7	0.04
Immunosuppressants	1.7	1.1	0.05	1.4	1.3	0
Lipid modifying agents	11.4	7.9	0.12	9.5	8.6	0.03
Opioids	13.1	7.7	0.18	10.6	11	-0.01
Psycholeptics	13.3	8.7	0.15	10	10.9	-0.03
Psychostimulants, agents used for ADHD and nootropics	3.6	2.7	0.05	3	3.3	-0.02

Table S6.157. Selected baseline characteristics for US PharMetrics Plus for the medium-term risk of anxiety disorders

Characteristic	Before propensity score matching			After propensity score matching		
	**Targets, %**	**Comparators, %**	**Standardized difference**	**Targets, %**	**Comparators, %**	**Standardized difference**
**Age group (years)**						
0-4	0.7	3.7	-0.2	1	2.6	-0.12
5-9	1.2	5.5	-0.24	1.7	3.2	-0.1
10-14	2.3	6.1	-0.19	2.9	4.1	-0.06
15-19	6.1	6.7	-0.03	7.1	7.5	-0.02
20-24	9.1	7.5	0.06	9.8	10	-0.01
25-29	7.2	7.1	0.01	6.9	7.2	-0.01
30-34	7.7	7.3	0.02	7.7	8.1	-0.02
35-39	8.2	7.5	0.03	8.1	8	0
40-44	8.8	7.3	0.06	8.5	8.2	0.01
45-49	9.9	7.2	0.1	9.7	8.5	0.04
50-54	10.8	7.8	0.1	10.9	9.3	0.05
55-59	10.7	8.6	0.07	10.6	9.4	0.04
60-64	9.1	8.5	0.02	8.8	7.7	0.04
65-69	3.2	4.1	-0.05	2.7	2.5	0.01
70-74	1.6	1.9	-0.03	1.3	1.3	0
75-79	1.1	1.3	-0.02	0.8	0.7	0
80-84	2.4	1.9	0.03	1.5	1.7	-0.02
**Sex**						
Female	52.5	50.1	0.05	48.9	50.4	-0.03

Table S6.157. *Continued.* Selected baseline characteristics for US PharMetrics Plus for the medium-term risk of anxiety disorders

Characteristic	Before propensity score matching			After propensity score matching		
	**Targets, %**	**Comparators, %**	**Standardized difference**	**Targets, %**	**Comparators, %**	**Standardized difference**
**Medical history (general)**						
Acute respiratory disease	40.9	18.6	0.5	34.4	39.6	-0.11
Attention deficit hyperactivity disorder	3.2	2.9	0.02	2.3	2.6	-0.03
Chronic liver disease	0.4	0.2	0.03	0.3	0.3	0
Chronic obstructive lung disease	2.3	1.2	0.09	1.3	1.7	-0.03
Crohn’s disease	0.3	0.2	0.02	0.2	0.3	-0.02
Dementia	1.4	0.4	0.11	0.6	0.9	-0.03
Depressive disorder	10.3	6.9	0.12	4.3	4.8	-0.02
Diabetes mellitus	12.2	5.9	0.22	9.8	8.8	0.03
Gastroesophageal reflux disease	11	5.8	0.19	8	8.3	-0.01
Gastrointestinal hemorrhage	1.6	0.8	0.07	1.1	1.2	-0.01
Human immunodeficiency virus infection	0.3	0.2	0.03	0.3	0.3	-0.02
Hyperlipidemia	22.7	14.9	0.2	19.3	18	0.04
Hypertensive disorder	27.4	16.3	0.27	22.6	21.1	0.04
Lesion of liver	0.5	0.3	0.03	0.3	0.3	0
Obesity	12.4	5.8	0.23	9.4	8.5	0.03
Osteoarthritis	11.5	7	0.15	9.4	9.1	0.01
Pneumonia	10.6	1.1	0.41	3.9	2.8	0.06
Psoriasis	1.1	0.8	0.03	0.9	0.9	0
Renal impairment	5	1.8	0.17	3	2.9	0
Rheumatoid arthritis	1.1	0.5	0.06	0.8	0.8	0.01
Urinary tract infectious disease	5.5	2.5	0.16	3.9	4.2	-0.01
Viral hepatitis C	0.2	0.1	0.01	0.1	0.1	0

Table S6.157. *Continued.* Selected baseline characteristics for US PharMetrics Plus for the medium-term risk of anxiety disorders

Characteristic	Before propensity score matching			After propensity score matching		
	Targets, %	Comparators, %	Standardized diff	Targets, %	Comparators, %	Standardized diff
**Medical history (cardiovascular disease)**						
Atrial fibrillation	2.4	1.2	0.09	1.6	1.7	-0.01
Cerebrovascular disease	1.2	0.7	0.05	0.9	0.9	0
Coronary arteriosclerosis	4	2.2	0.1	3	3.1	0
Heart disease	10.3	5.6	0.17	7.6	7.7	0
Heart failure	2.4	0.9	0.12	1.4	1.6	-0.01
Ischemic heart disease	2.5	1.1	0.1	1.6	1.6	0
Peripheral vascular disease	2.1	0.9	0.1	1.4	1.5	-0.01
Pulmonary embolism	0.6	0.2	0.07	0.3	0.3	0
Venous thrombosis	0.8	0.4	0.06	0.6	0.6	0
**Medical history (neoplasms)**						
Malignant lymphoma	0.3	0.2	0.03	0.2	0.2	0
Malignant neoplasm of anorectum	0.1	0.1	0	0.1	0.1	-0.01
Malignant neoplastic disease	3.9	3.1	0.04	3.2	3.4	-0.01
Malignant tumor of breast	0.7	0.6	0.01	0.5	0.6	0
Malignant tumor of colon	0.2	0.1	0.02	0.1	0.1	0
Malignant tumor of lung	0.2	0.1	0.01	0.1	0.1	-0.01
Malignant tumor of urinary bladder	0.1	0.1	0.01	0.1	0.1	-0.01
Primary malignant neoplasm of prostate	0.5	0.4	0.01	0.5	0.4	0.01
**Medication use**						
Agents acting on the renin-angiotensin system	11.9	8	0.13	10.1	9.1	0.03
Antibacterials for systemic use	39.7	25.2	0.31	33.7	35.6	-0.04
Antidepressants	12.7	9	0.12	6.4	6.9	-0.02
Antiepileptics	6.1	3.9	0.1	3.6	3.8	-0.01
Antiinflammatory and antirheumatic products	19.8	10.8	0.25	15.9	15.2	0.02
Antineoplastic agents	2.8	1.9	0.06	2.3	2.4	-0.01
Antipsoriatics	1.8	1.3	0.05	1.7	1.7	-0.01

Table S6.157. *Continued.* Selected baseline characteristics for US PharMetrics Plus for the medium-term risk of anxiety disorders

Characteristic	Before propensity score matching			After propensity score matching		
	Targets, %	Comparators, %	Standardized difference	Targets, %	Comparators, %	Standardized difference
**Medication use**						
Antithrombotic agents	4.9	2.5	0.13	3.5	3.5	0
Beta blocking agents	7	4.6	0.1	5.3	4.9	0.01
Calcium channel blockers	5.3	3.5	0.09	4.3	4	0.02
Diuretics	8.9	5.4	0.13	7.2	6.7	0.02
Drugs for acid-related disorders	12.9	7.5	0.18	9.8	10.2	-0.01
Drugs for obstructive airway diseases	24.6	14.2	0.26	20.2	21	-0.02
Drugs used in diabetes	7.1	3.8	0.15	5.7	5	0.03
Immunosuppressants	1.7	1	0.05	1.4	1.3	0.01
Lipid modifying agents	11.4	8	0.11	9.7	8.8	0.03
Opioids	13.1	7.8	0.18	10.2	10.6	-0.01
Psycholeptics	13.3	8.6	0.15	8.1	8.8	-0.03
Psychostimulants, agents used for ADHD and nootropics	3.6	2.7	0.05	2.6	3	-0.02

Table S6.158. Selected baseline characteristics for US PharMetrics Plus for the medium-term risk of alcohol misuse or dependence

Characteristic	Before propensity score matching			After propensity score matching		
	Targets, %	Comparators, %	Standardized difference	Targets, %	Comparators, %	Standardized difference
**Age group (years)**						
0-4	0.7	3.7	-0.2	0.8	2.1	-0.1
5-9	1.2	5.5	-0.24	1.4	2.7	-0.09
10-14	2.3	6.1	-0.19	2.7	3.7	-0.06
15-19	6.1	6.6	-0.02	6.9	7.9	-0.04
20-24	9.1	7.5	0.06	10	10.4	-0.01
25-29	7.2	6.9	0.01	7	7.3	-0.01
30-34	7.7	7.1	0.02	7.9	8.3	-0.02
35-39	8.2	7.4	0.03	8.4	8.4	0
40-44	8.8	7.3	0.06	8.8	8.5	0.01
45-49	9.9	7.4	0.09	9.8	8.6	0.04
50-54	10.8	8.1	0.1	10.8	9.2	0.06
55-59	10.7	8.6	0.07	10.4	9.2	0.04
60-64	9.1	8.5	0.02	8.5	7.5	0.04
65-69	3.2	4.1	-0.05	2.6	2.4	0.02
75-79	1.1	1.3	-0.02	0.8	0.7	0.01
80-84	2.4	1.9	0.04	1.7	1.8	-0.01
**Sex**						
Female	52.5	50.1	0.05	53.1	54.7	-0.03

Table S6.158. *Continued.* Selected baseline characteristics for US PharMetrics Plus for the medium-term risk of alcohol misuse or dependence

Characteristic	Before propensity score matching			After propensity score matching		
	**Targets, %**	**Comparators, %**	**Standardized difference**	**Targets, %**	**Comparators, %**	**Standardized difference**
**Medical history (general)**						
Acute respiratory disease	40.9	18.6	0.5	36.5	42	-0.11
Attention deficit hyperactivity disorder	3.2	2.9	0.02	3.4	4	-0.03
Chronic liver disease	0.4	0.2	0.03	0.3	0.3	0
Chronic obstructive lung disease	2.3	1.2	0.09	1.6	1.9	-0.02
Crohn's disease	0.3	0.2	0.02	0.3	0.4	-0.02
Dementia	1.4	0.4	0.11	0.9	1.1	-0.03
Depressive disorder	10.3	6.9	0.12	9.5	10.8	-0.04
Diabetes mellitus	12.2	6	0.22	9.9	8.7	0.04
Gastroesophageal reflux disease	11	5.8	0.19	9.8	10.1	-0.01
Gastrointestinal hemorrhage	1.6	0.8	0.07	1.3	1.4	-0.01
Human immunodeficiency virus infection	0.3	0.2	0.03	0.3	0.4	-0.01
Hyperlipidemia	22.7	14.7	0.2	20.2	18.6	0.04
Hypertensive disorder	27.4	16.3	0.27	23.8	22	0.04
Lesion of liver	0.5	0.2	0.04	0.3	0.3	0
Obesity	12.4	5.9	0.23	10.5	9.7	0.03
Osteoarthritis	11.5	7	0.16	10.5	10.2	0.01
Pneumonia	10.6	1.1	0.41	4.2	2.9	0.07
Psoriasis	1.1	0.8	0.03	1	1	0
Renal impairment	5	1.9	0.17	3.1	3	0
Rheumatoid arthritis	1.1	0.5	0.06	1	0.9	0.01
Schizophrenia	0.1	0.1	0.01	0.1	0.1	-0.01
Ulcerative colitis	0.3	0.2	0.03	0.3	0.3	0
Urinary tract infectious disease	5.5	2.4	0.16	4.7	5.1	-0.02
Viral hepatitis C	0.2	0.1	0.02	0.1	0.2	-0.01

Table S6.158. *Continued.* Selected baseline characteristics for US PharMetrics Plus for the medium-term risk of alcohol misuse or dependence

Characteristic	Before propensity score matching			After propensity score matching		
	Targets, %	Comparators, %	Standardized difference	Targets, %	Comparators, %	Standardized difference
**Medical history (cardiovascular disease)**						
Atrial fibrillation	2.4	1.3	0.08	1.8	1.7	0
Cerebrovascular disease	1.2	0.7	0.05	0.9	1	0
Coronary arteriosclerosis	4	2.3	0.1	3.2	3.1	0
Heart disease	10.3	5.6	0.17	8.2	8.3	0
Heart failure	2.4	0.9	0.12	1.6	1.7	-0.01
Ischemic heart disease	2.5	1.1	0.1	1.8	1.7	0
Peripheral vascular disease	2.1	0.9	0.1	1.5	1.6	-0.01
Pulmonary embolism	0.6	0.2	0.06	0.4	0.4	0
Venous thrombosis	0.8	0.4	0.06	0.6	0.7	0
**Medical history (neoplasms)**						
Malignant lymphoma	0.3	0.2	0.02	0.3	0.3	0
Malignant neoplasm of anorectum	0.1	0.1	0.01	0.1	0.1	-0.01
Malignant neoplastic disease	3.9	3.1	0.04	3.5	3.6	0
Malignant tumor of breast	0.7	0.6	0.01	0.6	0.6	0
Malignant tumor of colon	0.2	0.1	0.01	0.1	0.1	0
Malignant tumor of lung	0.2	0.1	0.02	0.1	0.2	-0.01
Malignant tumor of urinary bladder	0.1	0.1	0	0.1	0.1	-0.01
Primary malignant neoplasm of prostate	0.5	0.4	0.01	0.4	0.3	0.01
**Medication use**						
Agents acting on the renin-angiotensin system	11.9	8	0.13	10.2	9.2	0.03
Antibacterials for systemic use	39.7	25.1	0.31	35.7	37.9	-0.04
Antidepressants	12.7	9	0.12	11.7	13	-0.04
Antiepileptics	6.1	3.9	0.1	5.2	5.7	-0.02
Antiinflammatory and antirheumatic products	19.8	10.8	0.25	17.5	17.2	0.01
Antineoplastic agents	2.8	1.8	0.07	2.6	2.8	-0.01

Table S6.158. *Continued.* Selected baseline characteristics for US PharMetrics Plus for the medium-term risk of alcohol misuse or dependence

Characteristic	Before propensity score matching			After propensity score matching		
	Targets, %	Comparators, %	Standardized difference	Targets, %	Comparators, %	Standardized difference
**Medication use**						
Antipsoriatics	1.8	1.3	0.05	1.9	2	-0.01
Antithrombotic agents	4.9	2.5	0.13	3.9	3.9	0
Beta blocking agents	7	4.5	0.11	5.9	5.6	0.01
Calcium channel blockers	5.3	3.4	0.1	4.4	4.1	0.02
Diuretics	8.9	5.4	0.13	7.6	7.1	0.02
Drugs for acid-related disorders	12.9	7.4	0.18	11.3	11.9	-0.02
Drugs for obstructive airway diseases	24.6	14.2	0.26	21.9	23	-0.03
Drugs used in diabetes	7.1	3.8	0.15	5.8	5.2	0.03
Lipid modifying agents	11.4	7.9	0.12	9.8	9	0.03
Opioids	13.1	7.7	0.18	11.7	12.4	-0.02
Psycholeptics	13.3	8.6	0.15	12.1	13.5	-0.04
Psychostimulants, agents used for ADHD and nootropics	3.6	2.7	0.05	3.5	4	-0.02

Table S6.159. Selected baseline characteristics for US PharMetrics Plus for the medium-term risk of substance misuse or dependence

Characteristic	Before propensity score matching			After propensity score matching		
	**Targets, %**	**Comparators, %**	**Standardized difference**	**Targets, %**	**Comparators, %**	**Standardized difference**
**Age group (years)**						
0-4	0.7	3.7	-0.21	0.9	2.2	-0.11
5-9	1.2	5.5	-0.24	1.5	2.9	-0.09
10-14	2.3	6.1	-0.19	2.8	3.9	-0.06
15-19	6.1	6.6	-0.02	7.1	8	-0.03
20-24	9.1	7.6	0.06	9.9	10.5	-0.02
25-29	7.2	6.9	0.01	7	7.5	-0.02
30-34	7.7	7.2	0.02	7.9	8.4	-0.02
35-39	8.2	7.5	0.03	8.3	8.3	0
40-44	8.8	7.4	0.05	8.8	8.3	0.02
45-49	9.9	7.4	0.09	9.7	8.5	0.04
50-54	10.8	8	0.1	10.8	9	0.06
55-59	10.7	8.5	0.07	10.3	8.8	0.05
60-64	9.1	8.5	0.02	8.4	7.3	0.04
65-69	3.2	4.2	-0.05	2.6	2.3	0.02
70-74	1.6	2	-0.03	1.3	1.3	0
75-79	1.1	1.3	-0.02	0.8	0.7	0.01
80-84	2.4	1.9	0.04	1.7	1.9	-0.01
**Sex**						
Female	52.5	49.9	0.05	53.5	55	-0.03

Table S6.159. *Continued.* Selected baseline characteristics for US PharMetrics Plus for the medium-term risk of substance misuse or dependence

**Characteristic**	**Before propensity score matching**			**After propensity score matching**		
	**Targets, %**	**Comparators, %**	**Standardized difference**	**Targets, %**	**Comparators, %**	**Standardized difference**
**Medical history (general)**						
Acute respiratory disease	40.9	18.5	0.5	36.1	41.1	-0.1
Attention deficit hyperactivity disorder	3.2	2.9	0.02	3.2	3.9	-0.03
Chronic liver disease	0.4	0.3	0.03	0.2	0.2	0
Chronic obstructive lung disease	2.3	1.2	0.08	1.3	1.3	-0.01
Crohn’s disease	0.3	0.2	0.01	0.3	0.4	-0.02
Dementia	1.4	0.4	0.11	0.9	1.1	-0.03
Depressive disorder	10.3	6.9	0.12	8.8	10.1	-0.04
Diabetes mellitus	12.2	5.9	0.22	9.6	8.5	0.04
Gastroesophageal reflux disease	11	5.9	0.19	9.3	9.6	-0.01
Gastrointestinal hemorrhage	1.6	0.8	0.07	1.2	1.3	-0.01
Human immunodeficiency virus infection	0.3	0.2	0.03	0.2	0.3	-0.01
Hyperlipidemia	22.7	14.8	0.2	19.7	18	0.04
Hypertensive disorder	27.4	16.5	0.27	23.1	21.1	0.05
Lesion of liver	0.5	0.3	0.03	0.3	0.3	0
Obesity	12.4	5.8	0.23	10.1	9.3	0.03
Osteoarthritis	11.5	7.1	0.15	10.1	9.5	0.02
Pneumonia	10.6	1.1	0.41	3.9	2.7	0.07
Psoriasis	1.1	0.8	0.03	1	0.9	0.01
Renal impairment	5	1.9	0.17	2.9	2.8	0.01
Rheumatoid arthritis	1.1	0.6	0.06	0.9	0.8	0.02
Ulcerative colitis	0.3	0.2	0.02	0.3	0.3	0
Urinary tract infectious disease	5.5	2.5	0.15	4.5	4.9	-0.01
Viral hepatitis C	0.2	0.1	0.01	0.1	0.1	0

Table S6.159. *Continued.* Selected baseline characteristics for US PharMetrics Plus for the medium-term risk of substance misuse or dependence

**Characteristic**	**Before propensity score matching**			**After propensity score matching**		
	**Targets, %**	**Comparators, %**	**Standardized difference**	**Targets, %**	**Comparators, %**	**Standardized difference**
**Medical history (cardiovascular disease)**						
Atrial fibrillation	2.4	1.3	0.08	1.7	1.7	0
Cerebrovascular disease	1.2	0.7	0.05	0.9	0.9	-0.01
Coronary arteriosclerosis	4	2.2	0.1	2.9	2.9	0
Heart failure	2.4	0.9	0.12	1.4	1.5	-0.01
Ischemic heart disease	2.5	1.1	0.1	1.6	1.6	0
Peripheral vascular disease	2.1	0.9	0.1	1.4	1.4	0
Pulmonary embolism	0.6	0.2	0.07	0.3	0.3	0
Venous thrombosis	0.8	0.4	0.05	0.6	0.6	0
**Medical history (neoplasms)**						
Malignant lymphoma	0.3	0.2	0.03	0.2	0.2	0
Malignant neoplasm of anorectum	0.1	0.1	0.01	0.1	0.1	-0.01
Malignant neoplastic disease	3.9	3	0.05	3.4	3.5	0
Malignant tumor of breast	0.7	0.5	0.01	0.6	0.7	0
Malignant tumor of colon	0.2	0.1	0.02	0.1	0.1	0
Malignant tumor of lung	0.2	0.1	0.01	0.1	0.1	-0.01
Malignant tumor of urinary bladder	0.1	0.1	0.01	0.1	0.1	-0.01
Primary malignant neoplasm of prostate	0.5	0.4	0.01	0.4	0.4	0.01
**Medication use**						
Agents acting on the renin-angiotensin system	11.9	8.1	0.12	9.9	8.9	0.03
Antibacterials for systemic use	39.7	25.2	0.31	35.2	36.7	-0.03
Antidepressants	12.7	9.1	0.11	11.2	12.3	-0.04
Antiepileptics	6.1	3.9	0.1	4.8	5.1	-0.01
Antiinflammatory and antirheumatic products	19.8	10.9	0.25	16.8	16	0.02
Antineoplastic agents	2.8	1.9	0.06	2.5	2.7	-0.01
Antipsoriatics	1.8	1.3	0.05	1.9	2	-0.01

Table S6.159. *Continued.* Selected baseline characteristics for US PharMetrics Plus for the medium-term risk of substance misuse or dependence

Characteristic	Before propensity score matching			After propensity score matching		
	Targets, %	Comparators, %	Standardized difference	Targets, %	Comparators, %	Standardized difference
**Medication use**						
Antithrombotic agents	4.9	2.5	0.13	3.6	3.5	0
Beta blocking agents	7	4.6	0.1	5.6	5.3	0.01
Calcium channel blockers	5.3	3.5	0.09	4.3	3.9	0.02
Diuretics	8.9	5.5	0.13	7.3	6.8	0.02
Drugs for acid-related disorders	12.9	7.5	0.18	10.8	11.2	-0.01
Drugs for obstructive airway diseases	24.6	14.3	0.26	21.5	22.1	-0.01
Drugs used in diabetes	7.1	3.8	0.14	5.6	5	0.03
Immunosuppressants	1.7	1.1	0.05	1.4	1.4	0
Lipid modifying agents	11.4	8.1	0.11	9.6	8.5	0.04
Opioids	13.1	7.8	0.17	10.8	11.1	-0.01
Psycholeptics	13.3	8.7	0.15	11.4	12.4	-0.03
Psychostimulants, agents used for ADHD and nootropics	3.6	2.7	0.05	3.4	3.8	-0.02

Table S6.160. Selected baseline characteristics for US PharMetrics Plus for the medium-term risk of bipolar disorders

Characteristic	Before propensity score matching			After propensity score matching		
	Targets, %	Comparators, %	Standardized difference	Targets, %	Comparators, %	Standardized difference
**Age group (years)**						
0-4	0.7	3.7	-0.2	0.8	2	-0.1
5-9	1.2	5.5	-0.24	1.4	2.6	-0.08
10-14	2.3	6.1	-0.19	2.7	3.7	-0.06
15-19	6.1	6.6	-0.02	6.9	7.6	-0.03
20-24	9.1	7.5	0.06	9.9	10.2	-0.01
25-29	7.2	6.9	0.01	6.9	7.4	-0.02
30-34	7.7	7.2	0.02	7.9	8.4	-0.02
35-39	8.2	7.5	0.03	8.4	8.4	0
40-44	8.8	7.4	0.05	8.8	8.6	0.01
45-49	9.9	7.4	0.09	9.8	8.6	0.04
50-54	10.8	8	0.1	10.8	9.2	0.05
55-59	10.7	8.6	0.07	10.5	9.2	0.04
60-64	9.1	8.4	0.03	8.6	7.6	0.04
65-69	3.2	4.1	-0.05	2.6	2.4	0.01
75-79	1.1	1.3	-0.02	0.8	0.8	0.01
80-84	2.4	1.9	0.03	1.7	1.8	-0.01
**Sex**						
Female	52.5	50	0.05	52.7	54.2	-0.03

Table S6.160. *Continued.* Selected baseline characteristics for US PharMetrics Plus for the medium-term risk of bipolar disorders

Characteristic	Before propensity score matching			After propensity score matching		
	Targets, %	Comparators, %	Standardized difference	Targets, %	Comparators, %	Standardized difference
**Medical history (general)**						
Acute respiratory disease	40.9	18.6	0.5	36.4	41.2	-0.1
Attention deficit hyperactivity disorder	3.2	2.9	0.02	3.3	3.9	-0.03
Chronic liver disease	0.4	0.2	0.03	0.3	0.3	0
Chronic obstructive lung disease	2.3	1.2	0.08	1.6	2	-0.02
Crohn’s disease	0.3	0.2	0.01	0.3	0.4	-0.02
Dementia	1.4	0.4	0.11	0.9	1.1	-0.02
Depressive disorder	10.3	6.9	0.12	9.3	10.6	-0.04
Diabetes mellitus	12.2	6	0.22	9.9	8.8	0.04
Gastroesophageal reflux disease	11	5.8	0.19	9.8	10.2	-0.02
Gastrointestinal hemorrhage	1.6	0.8	0.07	1.3	1.5	-0.01
Human immunodeficiency virus infection	0.3	0.2	0.03	0.3	0.3	-0.01
Hyperlipidemia	22.7	14.9	0.2	20.2	18.8	0.04
Hypertensive disorder	27.4	16.5	0.27	23.9	22.4	0.04
Lesion of liver	0.5	0.3	0.03	0.4	0.4	-0.01
Obesity	12.4	5.8	0.23	10.4	9.8	0.02
Osteoarthritis	11.5	7.1	0.15	10.4	10.2	0.01
Pneumonia	10.6	1.1	0.41	4.2	3	0.07
Psoriasis	1.1	0.8	0.03	1	1	0
Renal impairment	5	1.9	0.17	3.2	3.1	0
Rheumatoid arthritis	1.1	0.6	0.06	1	0.9	0.01
Urinary tract infectious disease	5.5	2.5	0.16	4.6	5	-0.02
Viral hepatitis C	0.2	0.1	0.01	0.1	0.1	0

Table S6.160. *Continued.* Selected baseline characteristics for US PharMetrics Plus for the medium-term risk of bipolar disorders

Characteristic	Before propensity score matching			After propensity score matching		
	Targets, %	Comparators, %	Standardized difference	Targets, %	Comparators, %	Standardized difference
**Medical history (cardiovascular disease)**						
Atrial fibrillation	2.4	1.3	0.08	1.8	1.9	0
Cerebrovascular disease	1.2	0.7	0.05	1	1	-0.01
Coronary arteriosclerosis	4	2.3	0.1	3.2	3.2	0
Heart disease	10.3	5.7	0.17	8.3	8.5	-0.01
Heart failure	2.4	0.9	0.12	1.6	1.7	-0.01
Ischemic heart disease	2.5	1.1	0.1	1.8	1.9	-0.01
Peripheral vascular disease	2.1	0.9	0.1	1.5	1.6	-0.01
Pulmonary embolism	0.6	0.2	0.06	0.4	0.4	-0.01
Venous thrombosis	0.8	0.4	0.05	0.6	0.7	0
**Medical history (neoplasms)**						
Malignant lymphoma	0.3	0.2	0.03	0.3	0.3	0
Malignant neoplasm of anorectum	0.1	0.1	0.01	0.1	0.1	-0.01
Malignant neoplastic disease	3.9	3.1	0.04	3.5	3.6	-0.01
Malignant tumor of breast	0.7	0.6	0.01	0.6	0.7	-0.01
Malignant tumor of lung	0.2	0.1	0.02	0.1	0.2	-0.01
Malignant tumor of urinary bladder	0.1	0.1	0.01	0.1	0.1	-0.01
Primary malignant neoplasm of prostate	0.5	0.4	0.01	0.4	0.3	0.01
**Medication use**						
Agents acting on the renin-angiotensin system	11.9	8.1	0.13	10.2	9.4	0.03
Antibacterials for systemic use	39.7	25.1	0.32	35.7	37.7	-0.04
Antidepressants	12.7	9.2	0.11	11.6	13	-0.04
Antiepileptics	6.1	3.9	0.1	4.9	5.5	-0.02
Antiinflammatory and antirheumatic products	19.8	11	0.25	17.5	17	0.01
Antineoplastic agents	2.8	1.9	0.06	2.6	2.8	-0.01

Table S6.160. *Continued.* Selected baseline characteristics for US PharMetrics Plus for the medium-term risk of bipolar disorders

Characteristic	Before propensity score matching			After propensity score matching		
	Targets, %	Comparators, %	Standardized difference	Targets, %	Comparators, %	Standardized difference
**Medication use**						
Antipsoriatics	1.8	1.2	0.05	1.8	2	-0.01
Antithrombotic agents	4.9	2.5	0.13	3.9	4	0
Beta blocking agents	7	4.5	0.11	5.9	5.6	0.01
Calcium channel blockers	5.3	3.5	0.09	4.5	4.3	0.01
Diuretics	8.9	5.4	0.13	7.6	7.2	0.01
Drugs for acid-related disorders	12.9	7.5	0.18	11.3	11.9	-0.02
Drugs for obstructive airway diseases	24.6	14.1	0.27	21.9	22.9	-0.02
Drugs used in diabetes	7.1	3.8	0.15	5.8	5.2	0.03
Immunosuppressants	1.7	1.1	0.05	1.5	1.5	0
Lipid modifying agents	11.4	8	0.11	9.9	9	0.03
Opioids	13.1	7.8	0.17	11.6	12.3	-0.02
Psycholeptics	13.3	8.6	0.15	12	13.3	-0.04
Psychostimulants, agents used for ADHD and nootropics	3.6	2.7	0.05	3.5	3.9	-0.03

Table S6.161. Selected baseline characteristics for US PharMetrics Plus for the medium-term risk of psychoses

**Characteristic**	**Before propensity score matching**			**After propensity score matching**		
	**Targets, %**	**Comparators, %**	**Standardized difference**	**Targets, %**	**Comparators, %**	**Standardized difference**
**Age group (years)**						
0-4	0.7	3.7	-0.2	0.8	2.1	-0.1
5-9	1.2	5.5	-0.24	1.4	2.7	-0.09
10-14	2.3	6.1	-0.19	2.7	3.6	-0.06
15-19	6.1	6.6	-0.02	6.9	7.7	-0.03
20-24	9.1	7.5	0.06	10	10.4	-0.01
25-29	7.2	7	0.01	7	7.3	-0.01
30-34	7.7	7.2	0.02	7.9	8.4	-0.02
35-39	8.2	7.4	0.03	8.4	8.4	0
40-44	8.8	7.4	0.05	8.9	8.5	0.01
45-49	9.9	7.4	0.09	9.9	8.5	0.05
50-54	10.8	8	0.1	10.8	9.3	0.05
55-59	10.7	8.6	0.07	10.5	9.3	0.04
60-64	9.1	8.5	0.02	8.6	7.6	0.04
65-69	3.2	4	-0.05	2.6	2.5	0.01
75-79	1.1	1.3	-0.02	0.8	0.7	0.01
80-84	2.4	1.8	0.04	1.6	1.7	-0.01
**Sex**						
Female	52.5	50.1	0.05	52.8	54.5	-0.03

Table S6.161. *Continued.* Selected baseline characteristics for US PharMetrics Plus for the medium-term risk of psychoses

Characteristic	Before propensity score matching			After propensity score matching		
	Targets, %	Comparators, %	Standardized difference	Targets, %	Comparators, %	Standardized difference
**Medical history (general)**						
Acute respiratory disease	40.9	18.7	0.5	36.5	41.9	-0.11
Attention deficit hyperactivity disorder	3.2	2.9	0.02	3.4	4	-0.03
Chronic liver disease	0.4	0.2	0.03	0.3	0.3	0
Chronic obstructive lung disease	2.3	1.2	0.08	1.6	2	-0.03
Crohn’s disease	0.3	0.2	0.01	0.3	0.4	-0.02
Dementia	1.4	0.4	0.11	0.8	1	-0.02
Depressive disorder	10.3	6.9	0.12	9.6	11.1	-0.05
Diabetes mellitus	12.2	5.9	0.22	9.9	8.8	0.04
Gastroesophageal reflux disease	11	5.8	0.19	9.8	10.3	-0.02
Gastrointestinal hemorrhage	1.6	0.8	0.07	1.3	1.5	-0.01
Human immunodeficiency virus infection	0.3	0.2	0.03	0.3	0.3	-0.01
Hyperlipidemia	22.7	14.8	0.2	20.3	18.9	0.04
Hypertensive disorder	27.4	16.3	0.27	23.9	22.4	0.04
Lesion of liver	0.5	0.3	0.03	0.4	0.4	0
Obesity	12.4	5.7	0.23	10.5	9.9	0.02
Osteoarthritis	11.5	7	0.16	10.5	10.2	0.01
Pneumonia	10.6	1.2	0.41	4.2	3	0.07
Renal impairment	5	1.8	0.17	3.1	3.1	0
Rheumatoid arthritis	1.1	0.5	0.06	1	0.9	0.01
Urinary tract infectious disease	5.5	2.4	0.16	4.6	5	-0.02
Viral hepatitis C	0.2	0.1	0.02	0.1	0.2	-0.01

Table S6.161. *Continued.* Selected baseline characteristics for US PharMetrics Plus for the medium-term risk of psychoses

Characteristic	Before propensity score matching			After propensity score matching		
	Targets, %	Comparators, %	Standardized difference	Targets, %	Comparators, %	Standardized difference
**Medical history (cardiovascular disease)**						
Atrial fibrillation	2.4	1.2	0.09	1.8	1.7	0
Cerebrovascular disease	1.2	0.7	0.06	0.9	1	0
Coronary arteriosclerosis	4	2.3	0.1	3.2	3.2	0
Heart disease	10.3	5.7	0.17	8.3	8.4	0
Heart failure	2.4	0.9	0.12	1.6	1.7	-0.01
Ischemic heart disease	2.5	1.1	0.11	1.8	1.8	0
Peripheral vascular disease	2.1	0.9	0.1	1.5	1.5	0
Pulmonary embolism	0.6	0.2	0.06	0.4	0.4	-0.01
Venous thrombosis	0.8	0.4	0.05	0.6	0.7	0
**Medical history (neoplasms)**						
Malignant lymphoma	0.3	0.2	0.02	0.3	0.3	0
Malignant neoplasm of anorectum	0.1	0.1	0	0.1	0.1	-0.01
Malignant neoplastic disease	3.9	3.1	0.04	3.5	3.6	-0.01
Malignant tumor of breast	0.7	0.6	0.01	0.6	0.7	-0.01
Malignant tumor of colon	0.2	0.1	0.01	0.1	0.1	0
Malignant tumor of lung	0.2	0.1	0.02	0.1	0.2	-0.01
Malignant tumor of urinary bladder	0.1	0.1	0.01	0.1	0.1	0
Primary malignant neoplasm of prostate	0.5	0.4	0.02	0.4	0.3	0.01
**Medication use**						
Agents acting on the renin-angiotensin system	11.9	8	0.13	10.2	9.5	0.02
Antibacterials for systemic use	39.7	25	0.32	35.8	37.8	-0.04
Antidepressants	12.7	9.1	0.12	11.9	13.5	-0.05
Antiepileptics	6.1	3.8	0.1	5.2	5.8	-0.03
Antiinflammatory and antirheumatic products	19.8	10.8	0.25	17.6	17.2	0.01
Antineoplastic agents	2.8	2	0.06	2.6	2.8	-0.01

Table S6.161. *Continued.* Selected baseline characteristics for US PharMetrics Plus for the medium-term risk of psychoses

Sex	Before propensity score matching			After propensity score matching		
	**Targets, %**	**Comparators, %**	**Standardized difference**	**Targets, %**	**Comparators, %**	**Standardized difference**
**Medication use**						
Antipsoriatics	1.8	1.3	0.05	1.9	2	-0.01
Antithrombotic agents	4.9	2.5	0.13	3.9	3.9	0
Beta blocking agents	7	4.5	0.11	5.9	5.7	0.01
Calcium channel blockers	5.3	3.4	0.1	4.5	4.2	0.02
Diuretics	8.9	5.4	0.14	7.6	7.3	0.01
Drugs for acid-related disorders	12.9	7.4	0.18	11.4	12	-0.02
Drugs for obstructive airway diseases	24.6	14.2	0.26	22	23.1	-0.03
Drugs used in diabetes	7.1	3.8	0.15	5.8	5.2	0.02
Immunosuppressants	1.7	1.1	0.05	1.5	1.4	0
Lipid modifying agents	11.4	7.9	0.12	9.9	9.1	0.03
Opioids	13.1	7.8	0.18	11.7	12.5	-0.02
Psycholeptics	13.3	8.6	0.15	12.2	13.7	-0.04
Psychostimulants, agents used for ADHD and nootropics	3.6	2.6	0.06	3.6	4	-0.03

Table S6.162. Selected baseline characteristics for US PharMetrics Plus for the medium-term risk of personality disorders

Characteristic	Before propensity score matching			After propensity score matching		
	Targets, %	Comparators, %	Standardized difference	Targets, %	Comparators, %	Standardized difference
**Age group (years)**						
0-4	0.7	3.7	-0.2	0.8	2	-0.1
5-9	1.2	5.5	-0.24	1.4	2.7	-0.09
10-14	2.3	6.2	-0.2	2.6	3.6	-0.05
15-19	6.1	6.6	-0.02	6.9	7.7	-0.03
20-24	9.1	7.6	0.06	9.9	10.4	-0.01
25-29	7.2	6.9	0.01	6.9	7.3	-0.01
30-34	7.7	7.2	0.02	7.9	8.4	-0.02
40-44	8.8	7.4	0.05	8.8	8.5	0.01
45-49	9.9	7.3	0.09	9.9	8.6	0.04
50-54	10.8	8	0.1	10.8	9.3	0.05
55-59	10.7	8.6	0.07	10.5	9.3	0.04
60-64	9.1	8.4	0.02	8.6	7.5	0.04
65-69	3.2	4.1	-0.05	2.6	2.4	0.01
70-74	1.6	2	-0.03	1.4	1.3	0
75-79	1.1	1.3	-0.02	0.8	0.8	0.01
80-84	2.4	1.9	0.04	1.7	1.8	-0.01
**Sex**						
Female	52.5	50.1	0.05	52.8	54.5	-0.03

Table S6.162. *Continued.* Selected baseline characteristics for US PharMetrics Plus for the medium-term risk of personality disorders

Characteristic	Before propensity score matching			After propensity score matching		
	Targets, %	Comparators, %	Standardized difference	Targets, %	Comparators, %	Standardized difference
**Medical history (general)**						
Medical history: General						
Acute respiratory disease	40.9	18.4	0.51	36.5	41.8	-0.11
Attention deficit hyperactivity disorder	3.2	2.9	0.02	3.4	3.9	-0.03
Chronic liver disease	0.4	0.2	0.03	0.3	0.3	0
Chronic obstructive lung disease	2.3	1.2	0.09	1.7	2	-0.03
Crohn’s disease	0.3	0.2	0.02	0.3	0.4	-0.02
Dementia	1.4	0.4	0.11	0.9	1.1	-0.02
Depressive disorder	10.3	6.8	0.13	9.6	11.1	-0.05
Diabetes mellitus	12.2	5.9	0.22	9.9	8.8	0.04
Gastroesophageal reflux disease	11	5.8	0.19	9.9	10.3	-0.01
Gastrointestinal hemorrhage	1.6	0.9	0.06	1.3	1.5	-0.01
Human immunodeficiency virus infection	0.3	0.2	0.03	0.3	0.3	-0.01
Hyperlipidemia	22.7	14.8	0.2	20.3	18.9	0.04
Hypertensive disorder	27.4	16.3	0.27	24	22.5	0.04
Lesion of liver	0.5	0.2	0.04	0.4	0.4	-0.01
Obesity	12.4	5.8	0.23	10.5	9.9	0.02
Osteoarthritis	11.5	6.9	0.16	10.5	10.2	0.01
Pneumonia	10.6	1.1	0.41	4.3	3.1	0.06
Psoriasis	1.1	0.8	0.03	1	1	0
Renal impairment	5	1.9	0.17	3.2	3.2	0
Rheumatoid arthritis	1.1	0.6	0.06	1	0.9	0.01
Schizophrenia	0.1	0.1	0.01	0.1	0.1	-0.01
Ulcerative colitis	0.3	0.2	0.02	0.3	0.3	0
Urinary tract infectious disease	5.5	2.4	0.16	4.7	5	-0.01
Viral hepatitis C	0.2	0.1	0.02	0.1	0.2	-0.01

Table S6.162. *Continued.* Selected baseline characteristics for US PharMetrics Plus for the medium-term risk of personality disorders

Characteristic	Before propensity score matching			After propensity score matching		
	Targets, %	Comparators, %	Standardized difference	Targets, %	Comparators, %	Standardized difference
**Medical history (cardiovascular disease)**						
Atrial fibrillation	2.4	1.2	0.09	1.8	1.8	0
Cerebrovascular disease	1.2	0.7	0.05	1	1	0
Coronary arteriosclerosis	4	2.2	0.1	3.2	3.2	0
Heart disease	10.3	5.6	0.17	8.3	8.5	0
Heart failure	2.4	0.9	0.12	1.6	1.8	-0.01
Ischemic heart disease	2.5	1.1	0.11	1.8	1.9	0
Peripheral vascular disease	2.1	0.9	0.1	1.5	1.6	0
Pulmonary embolism	0.6	0.2	0.07	0.4	0.4	0
Venous thrombosis	0.8	0.4	0.05	0.6	0.7	0
**Medical history (neoplasms)**						
Malignant lymphoma	0.3	0.2	0.03	0.3	0.3	0
Malignant neoplasm of anorectum	0.1	0.1	0	0.1	0.1	0
Malignant neoplastic disease	3.9	3.1	0.05	3.5	3.6	0
Malignant tumor of breast	0.7	0.5	0.01	0.6	0.7	0
Malignant tumor of lung	0.2	0.1	0.01	0.1	0.2	-0.01
Malignant tumor of urinary bladder	0.1	0.1	0	0.1	0.1	-0.01
Primary malignant neoplasm of prostate	0.5	0.4	0.02	0.4	0.3	0.01
**Medication use**						
Agents acting on the renin-angiotensin system	11.9	7.9	0.13	10.2	9.4	0.03
Antibacterials for systemic use	39.7	25.2	0.31	35.8	37.6	-0.04
Antidepressants	12.7	9	0.12	11.9	13.3	-0.04
Antiepileptics	6.1	3.8	0.11	5.2	5.7	-0.02

Table S6.162. *Continued.* Selected baseline characteristics for US PharMetrics Plus for the medium-term risk of personality disorders

Characteristic	Before propensity score matching			After propensity score matching		
	Targets, %	Comparators, %	Standardized difference	Targets, %	Comparators, %	Standardized difference
**Medication use**						
Antiinflammatory and antirheumatic products	19.8	10.7	0.25	17.6	17.2	0.01
Antineoplastic agents	2.8	1.9	0.06	2.6	2.8	-0.01
Antipsoriatics	1.8	1.2	0.05	1.8	2	-0.01
Antithrombotic agents	4.9	2.5	0.13	3.9	4	0
Beta blocking agents	7	4.6	0.1	6	5.8	0.01
Calcium channel blockers	5.3	3.5	0.09	4.5	4.2	0.01
Diuretics	8.9	5.4	0.13	7.6	7.2	0.02
Drugs for acid-related disorders	12.9	7.4	0.18	11.4	12	-0.02
Drugs for obstructive airway diseases	24.6	14.1	0.27	22	22.9	-0.02
Drugs used in diabetes	7.1	3.8	0.15	5.8	5.2	0.03
Immunosuppressants	1.7	1	0.06	1.5	1.4	0
Lipid modifying agents	11.4	7.9	0.12	9.9	9.1	0.03
Opioids	13.1	7.6	0.18	11.7	12.3	-0.02
Psycholeptics	13.3	8.5	0.16	12.3	13.6	-0.04
Psychostimulants, agents used for ADHD and nootropics	3.6	2.7	0.05	3.5	3.9	-0.02

Table S6.163. Selected baseline characteristics for US PharMetrics Plus for the medium-term risk of self-harm and suicide

**Characteristic**	**Before propensity score matching**			**After propensity score matching**		
	**Targets, %**	**Comparators, %**	**Standardized difference**	**Targets, %**	**Comparators, %**	**Standardized difference**
**Age group (years)**						
0-4	0.7	3.7	-0.2	0.8	2	-0.1
5-9	1.2	5.5	-0.24	1.4	2.6	-0.09
10-14	2.3	6.1	-0.19	2.6	3.6	-0.06
15-19	6.1	6.6	-0.02	6.9	7.6	-0.03
20-24	9.1	7.5	0.06	10	10.4	-0.01
25-29	7.2	7	0.01	6.9	7.4	-0.02
30-34	7.7	7.2	0.02	7.9	8.5	-0.02
35-39	8.2	7.5	0.03	8.4	8.5	0
40-44	8.8	7.3	0.05	8.9	8.4	0.02
45-49	9.9	7.3	0.09	9.9	8.6	0.04
50-54	10.8	8	0.1	10.8	9.2	0.05
55-59	10.7	8.5	0.07	10.5	9.3	0.04
60-64	9.1	8.5	0.02	8.6	7.5	0.04
65-69	3.2	4.1	-0.05	2.6	2.4	0.02
70-74	1.6	1.9	-0.03	1.4	1.3	0
75-79	1.1	1.3	-0.02	0.8	0.7	0.01
80-84	2.4	1.9	0.03	1.7	1.9	-0.01
**Sex**						
Female	52.5	50.4	0.04	52.8	54.5	-0.03

Table S6.163. *Continued.* Selected baseline characteristics for US PharMetrics Plus for the medium-term risk of self-harm and suicide

Characteristic	Before propensity score matching			After propensity score matching		
	Targets, %	Comparators, %	Standardized difference	Targets, %	Comparators, %	Standardized difference
**Medical history (general)**						
Acute respiratory disease	40.9	18.5	0.51	36.5	41.8	-0.11
Attention deficit hyperactivity disorder	3.2	2.9	0.02	3.4	4	-0.03
Chronic liver disease	0.4	0.2	0.04	0.3	0.4	0
Chronic obstructive lung disease	2.3	1.2	0.09	1.7	2	-0.02
Crohn’s disease	0.3	0.2	0.01	0.3	0.4	-0.02
Dementia	1.4	0.4	0.11	0.9	1.1	-0.02
Depressive disorder	10.3	6.8	0.12	9.7	11.2	-0.05
Diabetes mellitus	12.2	5.9	0.22	9.9	8.8	0.04
Gastroesophageal reflux disease	11	5.8	0.19	9.9	10.3	-0.01
Gastrointestinal hemorrhage	1.6	0.8	0.07	1.3	1.5	-0.01
Human immunodeficiency virus infection	0.3	0.2	0.03	0.3	0.4	-0.01
Hyperlipidemia	22.7	14.8	0.2	20.3	18.9	0.04
Hypertensive disorder	27.4	16.4	0.27	24	22.4	0.04
Lesion of liver	0.5	0.2	0.04	0.4	0.4	-0.01
Obesity	12.4	5.8	0.23	10.5	9.8	0.02
Osteoarthritis	11.5	7	0.16	10.5	10.2	0.01
Pneumonia	10.6	1.1	0.41	4.3	3	0.07
Psoriasis	1.1	0.8	0.03	1	1	0.01
Renal impairment	5	1.9	0.17	3.2	3.2	0
Rheumatoid arthritis	1.1	0.6	0.06	1	0.9	0.01
Schizophrenia	0.1	0.1	0.01	0.1	0.1	-0.01
Ulcerative colitis	0.3	0.2	0.02	0.3	0.3	0
Urinary tract infectious disease	5.5	2.5	0.16	4.7	5	-0.01
Viral hepatitis C	0.2	0.1	0.02	0.1	0.2	-0.01

Table S6.163. *Continued.* Selected baseline characteristics for US PharMetrics Plus for the medium-term risk of self-harm and suicide

Characteristic	Before propensity score matching			After propensity score matching		
	**Targets, %**	**Comparators, %**	**Standardized difference**	**Targets, %**	**Comparators, %**	**Standardized difference**
**Medical history (cardiovascular disease)**						
Atrial fibrillation	2.4	1.3	0.08	1.8	1.8	0
Cerebrovascular disease	1.2	0.7	0.05	1	1	0
Coronary arteriosclerosis	4	2.3	0.1	3.2	3.2	0
Heart disease	10.3	5.7	0.17	8.4	8.5	0
Heart failure	2.4	0.9	0.12	1.6	1.7	-0.01
Ischemic heart disease	2.5	1.1	0.11	1.8	1.9	0
Peripheral vascular disease	2.1	1	0.1	1.5	1.6	0
Venous thrombosis	0.8	0.4	0.06	0.6	0.7	0
Medical history: Neoplasms						
Malignant lymphoma	0.3	0.2	0.02	0.3	0.3	0
Malignant neoplasm of anorectum	0.1	0.1	0	0.1	0.1	-0.01
Malignant neoplastic disease	3.9	3.2	0.04	3.5	3.6	-0.01
Malignant tumor of breast	0.7	0.6	0	0.6	0.7	0
Malignant tumor of colon	0.2	0.1	0.01	0.1	0.1	0
Malignant tumor of lung	0.2	0.1	0.01	0.1	0.2	-0.01
Malignant tumor of urinary bladder	0.1	0.1	0.01	0.1	0.1	-0.01
Primary malignant neoplasm of prostate	0.5	0.4	0.01	0.4	0.4	0.01
Medication use						
Agents acting on the renin-angiotensin system	11.9	8	0.13	10.2	9.4	0.03
Antibacterials for systemic use	39.7	25.1	0.32	35.8	37.8	-0.04
Antidepressants	12.7	9.1	0.11	11.9	13.3	-0.04
Antiepileptics	6.1	3.9	0.1	5.3	5.8	-0.02
Antiinflammatory and antirheumatic products	19.8	10.9	0.25	17.6	17.3	0.01
Antineoplastic agents	2.8	2	0.06	2.6	2.7	-0.01
Antipsoriatics	1.8	1.2	0.05	1.8	2	-0.01

Table S6.163. *Continued.* Selected baseline characteristics for US PharMetrics Plus for the medium-term risk of self-harm and suicide

Characteristic	Before propensity score matching			After propensity score matching		
	**Targets, %**	**Comparators, %**	**Standardized difference**	**Targets, %**	**Comparators, %**	**Standardized difference**
**Medication use**						
Antithrombotic agents	4.9	2.5	0.13	3.9	4	0
Beta blocking agents	7	4.6	0.1	6	5.7	0.01
Calcium channel blockers	5.3	3.5	0.09	4.5	4.2	0.02
Diuretics	8.9	5.5	0.13	7.6	7.2	0.01
Drugs for acid-related disorders	12.9	7.5	0.18	11.4	11.9	-0.01
Drugs for obstructive airway diseases	24.6	14.2	0.26	22	23	-0.02
Drugs used in diabetes	7.1	3.8	0.15	5.8	5.1	0.03
Immunosuppressants	1.7	1.1	0.05	1.5	1.4	0.01
Lipid modifying agents	11.4	7.9	0.12	9.9	9	0.03
Opioids	13.1	7.8	0.18	11.8	12.4	-0.02
Psycholeptics	13.3	8.6	0.15	12.3	13.7	-0.04
Psychostimulants, agents used for ADHD and nootropics	3.6	2.7	0.05	3.6	4	-0.03

Table S6.164. Selected baseline characteristics for US PharMetrics Plus for the medium-term risk of sleep disorders

Characteristic	Before propensity score matching			After propensity score matching		
	Targets, %	Comparators, %	Standardized difference	Targets, %	Comparators, %	Standardized difference
**Age group (years)**						
0-4	0.7	3.7	-0.21	0.9	2.3	-0.11
45421	1.2	5.4	-0.24	1.6	2.9	-0.09
45579	2.3	6.1	-0.19	2.9	4	-0.06
15-19	6.1	6.5	-0.02	7.7	8.4	-0.03
20-24	9.1	7.5	0.06	11	11.4	-0.01
25-29	7.2	6.9	0.01	7.5	7.8	-0.01
30-34	7.7	7.2	0.02	8.3	8.9	-0.02
35-39	8.2	7.5	0.03	8.6	8.5	0
40-44	8.8	7.3	0.05	8.8	8.5	0.01
45-49	9.9	7.4	0.09	9.4	8.1	0.05
50-54	10.8	8.1	0.09	10.2	8.5	0.06
55-59	10.7	8.5	0.07	9.7	8.6	0.04
60-64	9.1	8.5	0.02	7.8	6.8	0.04
65-69	3.2	4.1	-0.05	2.4	2.1	0.02
70-74	1.6	2	-0.03	1.2	1.1	0
75-79	1.1	1.3	-0.02	0.7	0.6	0.01
80-84	2.4	1.9	0.03	1.5	1.6	-0.01
**Sex**						
Female	52.5	50	0.05	53.1	54.7	-0.03

Table S6.164. *Continued.* Selected baseline characteristics for US PharMetrics Plus for the medium-term risk of sleep disorders

Characteristic	Before propensity score matching			After propensity score matching		
	Targets, %	Comparators, %	Standardized difference	Targets, %	Comparators, %	Standardized difference
**Medical history (general)**						
Acute respiratory disease	40.9	18.4	0.51	35.6	40.8	-0.11
Attention deficit hyperactivity disorder	3.2	2.9	0.02	3.2	3.7	-0.03
Chronic liver disease	0.4	0.2	0.03	0.3	0.3	0
Chronic obstructive lung disease	2.3	1.2	0.09	1.2	1.5	-0.02
Crohn’s disease	0.3	0.2	0.01	0.2	0.4	-0.02
Dementia	1.4	0.4	0.11	0.7	0.9	-0.02
Depressive disorder	10.3	6.9	0.12	7.7	9	-0.04
Diabetes mellitus	12.2	5.9	0.22	8.5	7.4	0.04
Gastroesophageal reflux disease	11	5.9	0.19	8	8.4	-0.01
Gastrointestinal hemorrhage	1.6	0.8	0.07	1.1	1.3	-0.01
Human immunodeficiency virus infection	0.3	0.2	0.03	0.3	0.3	-0.01
Hyperlipidemia	22.7	14.7	0.2	17.5	16.1	0.04
Hypertensive disorder	27.4	16.4	0.27	20.5	19.1	0.03
Lesion of liver	0.5	0.2	0.04	0.3	0.3	0
Obesity	12.4	5.9	0.23	8.1	7.4	0.03
Osteoarthritis	11.5	7	0.16	8.6	8.4	0.01
Pneumonia	10.6	1.1	0.41	3.7	2.5	0.07
Psoriasis	1.1	0.8	0.03	0.9	0.9	0
Renal impairment	5	1.9	0.17	2.5	2.4	0.01
Rheumatoid arthritis	1.1	0.6	0.06	0.8	0.7	0.01
Schizophrenia	0.1	0.1	0.01	0.1	0.1	-0.01
Ulcerative colitis	0.3	0.2	0.02	0.3	0.3	0
Urinary tract infectious disease	5.5	2.5	0.16	4.3	4.6	-0.01
Viral hepatitis C	0.2	0.1	0.01	0.1	0.1	-0.01

Table S6.164. *Continued.* Selected baseline characteristics for US PharMetrics Plus for the medium-term risk of sleep disorders

**Characteristic**	**Before propensity score matching**			**After propensity score matching**		
	**Targets, %**	**Comparators, %**	**Standardized difference**	**Targets, %**	**Comparators, %**	**Standardized difference**
**Medical history (cardiovascular disease)**						
Atrial fibrillation	2.4	1.2	0.09	1.2	1.3	0
Cerebrovascular disease	1.2	0.7	0.05	0.7	0.8	-0.01
Coronary arteriosclerosis	4	2.2	0.1	2.4	2.4	0
Heart disease	10.3	5.6	0.18	6.5	6.7	-0.01
Heart failure	2.4	0.9	0.12	1.1	1.2	-0.01
Ischemic heart disease	2.5	1.1	0.11	1.3	1.4	0
Peripheral vascular disease	2.1	0.9	0.1	1.2	1.2	0
Pulmonary embolism	0.6	0.2	0.07	0.3	0.3	0
Venous thrombosis	0.8	0.4	0.05	0.5	0.5	0
**Medical history (neoplasms)**						
Malignant lymphoma	0.3	0.2	0.03	0.2	0.2	0
Malignant neoplasm of anorectum	0.1	0.1	0.01	0.1	0.1	-0.01
Malignant neoplastic disease	3.9	3.1	0.04	3	3.1	-0.01
Malignant tumor of breast	0.7	0.6	0.01	0.6	0.6	0
Malignant tumor of colon	0.2	0.1	0.01	0.1	0.1	0
Malignant tumor of lung	0.2	0.1	0.02	0.1	0.2	-0.01
Malignant tumor of urinary bladder	0.1	0.1	0.01	0.1	0.1	-0.01
Primary malignant neoplasm of prostate	0.5	0.4	0.01	0.4	0.3	0.01
**Medication use**						
Agents acting on the renin-angiotensin system	11.9	8.1	0.13	8.9	8.1	0.03
Antibacterials for systemic use	39.7	25.2	0.31	34.5	36.5	-0.04
Antidepressants	12.7	9.1	0.11	9.9	11.2	-0.04
Antiepileptics	6.1	3.8	0.1	4.1	4.6	-0.02
Antiinflammatory and antirheumatic products	19.8	11	0.25	16.2	15.9	0.01
Antineoplastic agents	2.8	1.9	0.06	2.3	2.5	-0.01

Table S6.164. *Continued.* Selected baseline characteristics for US PharMetrics Plus for the medium-term risk of sleep disorders

Characteristic	Before propensity score matching			After propensity score matching		
	**Targets, %**	**Comparators, %**	**Standardized difference**	**Targets, %**	**Comparators, %**	**Standardized difference**
**Medication use**						
Antipsoriatics	1.8	1.2	0.05	1.9	2	-0.01
Antithrombotic agents	4.9	2.5	0.13	3	3.1	0
Beta blocking agents	7	4.6	0.1	5	4.7	0.01
Calcium channel blockers	5.3	3.5	0.09	3.8	3.5	0.02
Diuretics	8.9	5.5	0.13	6.5	6.1	0.02
Drugs for acid-related disorders	12.9	7.5	0.18	9.8	10.4	-0.02
Drugs for obstructive airway diseases	24.6	14.2	0.26	20.2	21.2	-0.03
Drugs used in diabetes	7.1	3.8	0.15	5.1	4.4	0.03
Immunosuppressants	1.7	1.1	0.05	1.3	1.3	0
Lipid modifying agents	11.4	7.9	0.12	8.6	7.8	0.03
Opioids	13.1	7.8	0.18	10.1	10.8	-0.02
Psycholeptics	13.3	8.7	0.15	10	11.1	-0.04
Psychostimulants, agents used for ADHD and nootropics	3.6	2.7	0.05	3.2	3.7	-0.02

Table S6.165. Selected baseline characteristics for US PharMetrics Plus for the medium-term risk of dementia

Characteristic	Before propensity score matching			After propensity score matching		
	Targets, %	Comparators, %	Standardized difference	Targets, %	Comparators, %	Standardized difference
**Age group (years)**						
0-4	0.7	3.7	-0.2	0.8	2	-0.1
5-9	1.2	5.5	-0.24	1.4	2.7	-0.09
10-14	2.3	6.2	-0.19	2.7	3.6	-0.05
15-19	6.1	6.6	-0.02	7	7.7	-0.03
20-24	9.1	7.5	0.06	10	10.5	-0.02
25-29	7.2	6.9	0.01	7	7.4	-0.02
30-34	7.7	7.1	0.02	7.9	8.7	-0.03
35-39	8.2	7.4	0.03	8.4	8.4	0
40-44	8.8	7.4	0.05	8.9	8.6	0.01
45-49	9.9	7.3	0.09	9.9	8.7	0.04
50-54	10.8	8	0.1	10.9	9.2	0.06
55-59	10.7	8.6	0.07	10.5	9.3	0.04
60-64	9.1	8.4	0.02	8.6	7.6	0.04
65-69	3.2	4.2	-0.05	2.6	2.4	0.02
70-74	1.6	2	-0.03	1.3	1.3	0
75-79	1.1	1.3	-0.02	0.7	0.7	0.01
80-84	2.4	1.9	0.03	1.1	1.2	-0.01
**Sex**						
Female	52.5	50.1	0.05	52.7	54.6	-0.04

Table S6.165. *Continued.* Selected baseline characteristics for US PharMetrics Plus for the medium-term risk of dementia

Characteristic	Before propensity score matching			After propensity score matching		
	Targets, %	Comparators, %	Standardized difference	Targets, %	Comparators, %	Standardized difference
**Medical history (general)**						
Acute respiratory disease	40.9	18.6	0.5	36.6	41.8	-0.11
Attention deficit hyperactivity disorder	3.2	2.9	0.02	3.5	4	-0.03
Chronic liver disease	0.4	0.2	0.03	0.3	0.3	0
Chronic obstructive lung disease	2.3	1.2	0.08	1.5	1.9	-0.03
Crohn's disease	0.3	0.2	0.01	0.3	0.4	-0.02
Depressive disorder	10.3	6.9	0.12	9.4	11.1	-0.06
Diabetes mellitus	12.2	5.9	0.22	9.7	8.6	0.04
Gastroesophageal reflux disease	11	5.9	0.19	9.7	10.1	-0.01
Gastrointestinal hemorrhage	1.6	0.8	0.07	1.3	1.4	-0.01
Human immunodeficiency virus infection	0.3	0.2	0.03	0.3	0.3	-0.01
Hyperlipidemia	22.7	14.9	0.2	20	18.6	0.04
Hypertensive disorder	27.4	16.5	0.26	23.5	22	0.04
Lesion of liver	0.5	0.3	0.04	0.4	0.4	0
Obesity	12.4	5.8	0.23	10.5	9.9	0.02
Osteoarthritis	11.5	7	0.16	10.3	9.9	0.01
Pneumonia	10.6	1.1	0.41	4.1	2.8	0.07
Psoriasis	1.1	0.8	0.03	1	1	0
Renal impairment	5	1.9	0.17	2.9	2.8	0
Rheumatoid arthritis	1.1	0.5	0.06	1	0.9	0.01
Ulcerative colitis	0.3	0.2	0.02	0.3	0.3	0
Urinary tract infectious disease	5.5	2.4	0.16	4.4	4.8	-0.02
Viral hepatitis C	0.2	0.1	0.02	0.1	0.2	-0.01

Table S6.165. *Continued.* Selected baseline characteristics for US PharMetrics Plus for the medium-term risk of dementia

Characteristic	Before propensity score matching			After propensity score matching		
	Targets, %	Comparators, %	Standardized difference	Targets, %	Comparators, %	Standardized difference
**Medical history (cardiovascular disease)**						
Atrial fibrillation	2.4	1.2	0.09	1.6	1.6	0
Cerebrovascular disease	1.2	0.7	0.05	0.9	0.9	0
Coronary arteriosclerosis	4	2.2	0.1	3	3	0
Heart disease	10.3	5.6	0.17	7.9	8.1	-0.01
Heart failure	2.4	0.9	0.12	1.4	1.6	-0.01
Ischemic heart disease	2.5	1.1	0.1	1.7	1.7	0
Peripheral vascular disease	2.1	1	0.1	1.2	1.3	-0.01
Pulmonary embolism	0.6	0.2	0.07	0.4	0.4	0
**Medical history (neoplasms)**						
Malignant lymphoma	0.3	0.2	0.02	0.2	0.3	0
Malignant neoplasm of anorectum	0.1	0.1	0	0.1	0.1	-0.01
Malignant neoplastic disease	3.9	3.1	0.04	3.4	3.4	0
Malignant tumor of breast	0.7	0.6	0.01	0.6	0.6	0
Malignant tumor of colon	0.2	0.1	0.01	0.1	0.1	0
Malignant tumor of lung	0.2	0.1	0.01	0.1	0.2	-0.02
Malignant tumor of urinary bladder	0.1	0.1	0.01	0.1	0.1	-0.01
Primary malignant neoplasm of prostate	0.5	0.4	0.01	0.4	0.3	0.01
**Medication use**						
Agents acting on the renin-angiotensin system	11.9	8.1	0.13	10.2	9.3	0.03
Antibacterials for systemic use	39.7	25.1	0.31	35.8	37.8	-0.04
Antidepressants	12.7	9.1	0.12	11.8	13.4	-0.05
Antiepileptics	6.1	3.8	0.1	5.2	5.7	-0.02
Antiinflammatory and antirheumatic products	19.8	10.8	0.25	17.6	17.3	0.01
Antineoplastic agents	2.8	1.9	0.06	2.6	2.7	-0.01
Antipsoriatics	1.8	1.2	0.05	1.9	2	-0.01

Table S6.165. *Continued.* Selected baseline characteristics for US PharMetrics Plus for the medium-term risk of dementia

Characteristic	Before propensity score matching			After propensity score matching		
	**Targets, %**	**Comparators, %**	**Standardized difference**	**Targets, %**	**Comparators, %**	**Standardized difference**
**Medication use**						
Antithrombotic agents	4.9	2.5	0.13	3.8	3.9	0
Beta blocking agents	7	4.6	0.1	5.8	5.6	0.01
Calcium channel blockers	5.3	3.5	0.09	4.4	4	0.02
Diuretics	8.9	5.5	0.13	7.5	7.2	0.01
Drugs for acid-related disorders	12.9	7.5	0.18	11.4	11.8	-0.01
Drugs for obstructive airway diseases	24.6	14.3	0.26	22	23.1	-0.03
Drugs used in diabetes	7.1	3.8	0.14	5.8	5.2	0.03
Immunosuppressants	1.7	1.1	0.05	1.5	1.4	0.01
Lipid modifying agents	11.4	8.1	0.11	9.8	8.9	0.03
Opioids	13.1	7.8	0.17	11.7	12.4	-0.02
Psycholeptics	13.3	8.6	0.15	12.2	13.7	-0.04
Psychostimulants, agents used for ADHD and nootropics	3.6	2.6	0.06	3.6	4.1	-0.03

Table S6.166. Selected baseline characteristics for US PharMetrics Plus for the medium-term risk of neurodevelopmental disorders

Characteristic	Before propensity score matching			After propensity score matching		
	Targets, %	Comparators, %	Standardized difference	Targets, %	Comparators, %	Standardized difference
**Age group (years)**						
0-4	0.7	3.7	-0.21	0.8	2.1	-0.1
5-9	1.2	5.5	-0.24	1.3	2.6	-0.09
10-14	2.3	6.1	-0.19	2.4	3.2	-0.05
15-19	6.1	6.6	-0.02	6.5	7.3	-0.03
20-24	9.1	7.5	0.06	9.6	10.1	-0.02
25-29	7.2	7	0.01	6.8	7.3	-0.02
30-34	7.7	7.2	0.02	7.8	8.4	-0.02
35-39	8.2	7.4	0.03	8.3	8.3	0
40-44	8.8	7.3	0.05	8.9	8.6	0.01
45-49	9.9	7.4	0.09	10	8.8	0.04
50-54	10.8	8	0.1	11.1	9.4	0.06
55-59	10.7	8.6	0.07	10.8	9.6	0.04
60-64	9.1	8.4	0.02	8.8	7.7	0.04
65-69	3.2	4.2	-0.05	2.7	2.5	0.02
70-74	1.6	1.9	-0.03	1.4	1.4	0
75-79	1.1	1.3	-0.02	0.8	0.8	0
80-84	2.4	1.9	0.03	1.7	1.8	-0.01
**Sex**						
Female	52.5	50.1	0.05	52.8	54.7	-0.04

Table S6.166. *Continued.* Selected baseline characteristics for US PharMetrics Plus for the medium-term risk of neurodevelopmental disorders

Characteristic	Before propensity score matching			After propensity score matching		
	Targets, %	Comparators, %	Standardized difference	Targets, %	Comparators, %	Standardized difference
**Medical history (general)**						
Acute respiratory disease	40.9	18.5	0.51	36.1	41.5	-0.11
Chronic liver disease	0.4	0.2	0.03	0.3	0.4	0
Chronic obstructive lung disease	2.3	1.2	0.08	1.7	2	-0.03
Crohn’s disease	0.3	0.2	0.02	0.3	0.4	-0.02
Dementia	1.4	0.4	0.1	0.9	1.1	-0.02
Depressive disorder	10.3	6.9	0.12	9	10.3	-0.04
Diabetes mellitus	12.2	6	0.22	10.1	9	0.04
Gastroesophageal reflux disease	11	5.8	0.19	9.9	10.2	-0.01
Gastrointestinal hemorrhage	1.6	0.8	0.07	1.3	1.4	-0.01
Human immunodeficiency virus infection	0.3	0.2	0.03	0.3	0.4	-0.02
Hyperlipidemia	22.7	14.9	0.2	20.7	19.2	0.04
Hypertensive disorder	27.4	16.5	0.27	24.4	23	0.03
Lesion of liver	0.5	0.2	0.04	0.4	0.4	0
Obesity	12.4	5.8	0.23	10.6	10	0.02
Osteoarthritis	11.5	7	0.16	10.6	10.3	0.01
Pneumonia	10.6	1.1	0.41	4.3	3.1	0.06
Psoriasis	1.1	0.8	0.03	1	0.9	0.01
Renal impairment	5	1.9	0.17	3.2	3.2	0
Rheumatoid arthritis	1.1	0.6	0.06	1	0.9	0.01
Schizophrenia	0.1	0.1	0.01	0.1	0.1	-0.01
Ulcerative colitis	0.3	0.2	0.03	0.3	0.3	0
Urinary tract infectious disease	5.5	2.5	0.16	4.6	5	-0.02
Viral hepatitis C	0.2	0.1	0.02	0.1	0.2	-0.01

Table S6.166. *Continued.* Selected baseline characteristics for US PharMetrics Plus for the medium-term risk of neurodevelopmental disorders

Characteristic	Before propensity score matching			After propensity score matching		
	Targets, %	Comparators, %	Standardized difference	Targets, %	Comparators, %	Standardized difference
**Medical history (cardiovascular disease)**						
Atrial fibrillation	2.4	1.2	0.09	1.8	1.9	-0.01
Cerebrovascular disease	1.2	0.7	0.05	0.9	1	-0.01
Coronary arteriosclerosis	4	2.3	0.1	3.3	3.3	0
Heart disease	10.3	5.7	0.17	8.4	8.7	-0.01
Heart failure	2.4	0.9	0.12	1.6	1.8	-0.01
Ischemic heart disease	2.5	1.1	0.1	1.8	1.9	-0.01
Peripheral vascular disease	2.1	1	0.1	1.5	1.7	-0.01
Pulmonary embolism	0.6	0.2	0.06	0.4	0.4	0
Venous thrombosis	0.8	0.4	0.05	0.6	0.7	0
**Medical history (neoplasms)**						
Malignant lymphoma	0.3	0.2	0.02	0.3	0.3	0
Malignant neoplasm of anorectum	0.1	0.1	0.01	0.1	0.1	-0.01
Malignant neoplastic disease	3.9	3	0.05	3.6	3.7	-0.01
Malignant tumor of breast	0.7	0.6	0.01	0.6	0.7	0
Malignant tumor of colon	0.2	0.1	0.01	0.1	0.2	0
Malignant tumor of lung	0.2	0.1	0.02	0.1	0.2	-0.01
Malignant tumor of urinary bladder	0.1	0.1	0.01	0.1	0.1	-0.01
Primary malignant neoplasm of prostate	0.5	0.4	0.02	0.4	0.4	0.01
**Medication use**						
Agents acting on the renin-angiotensin system	11.9	8.1	0.12	10.5	9.7	0.03
Antibacterials for systemic use	39.7	25.2	0.31	35.5	37.5	-0.04
Antidepressants	12.7	9.1	0.12	11.3	12.7	-0.04
Antiepileptics	6.1	3.8	0.11	5	5.6	-0.02
Antiinflammatory and antirheumatic products	19.8	10.8	0.25	17.6	17.1	0.01
Antineoplastic agents	2.8	1.9	0.06	2.5	2.7	-0.01

Table S6.166. *Continued.* Selected baseline characteristics for US PharMetrics Plus for the medium-term risk of neurodevelopmental disorders

Characteristic	Before propensity score matching			After propensity score matching		
	Targets, %	Comparators, %	Standardized difference	Targets, %	Comparators, %	Standardized difference
**Medication use**						
Antipsoriatics	1.8	1.3	0.05	1.8	1.9	-0.01
Antithrombotic agents	4.9	2.5	0.13	3.9	4.1	-0.01
Beta blocking agents	7	4.6	0.1	6	5.9	0
Calcium channel blockers	5.3	3.5	0.09	4.6	4.4	0.01
Diuretics	8.9	5.5	0.13	7.7	7.4	0.01
Drugs for acid-related disorders	12.9	7.4	0.18	11.4	11.9	-0.02
Drugs for obstructive airway diseases	24.6	14	0.27	21.8	22.8	-0.03
Drugs used in diabetes	7.1	3.8	0.14	5.9	5.3	0.03
Immunosuppressants	1.7	1.1	0.05	1.5	1.4	0.01
Lipid modifying agents	11.4	8	0.11	10.1	9.3	0.03
Opioids	13.1	7.8	0.18	11.6	12.3	-0.02
Psycholeptics	13.3	8.6	0.15	11.9	13.3	-0.04
Psychostimulants, agents used for ADHD and nootropics	3.6	2.7	0.05	1.6	1.7	-0.01

Table S6.167. Selected baseline characteristics for US PharMetrics Plus for the medium-term risk of any of psychiatric and neuropsychiatric disorders

Characteristic	Before propensity score matching			After propensity score matching		
	Targets, %	Comparators, %	Standardized difference	Targets, %	Comparators, %	Standardized difference
**Age group (years)**						
0-4	0.7	3.7	-0.2	1.2	3	-0.13
5-9	1.2	5.5	-0.24	1.8	3.7	-0.11
10-14	2.3	6.1	-0.19	3.2	4.4	-0.06
15-19	6.1	6.6	-0.02	7.8	8.2	-0.02
20-24	9.1	7.5	0.06	10.5	10.8	-0.01
25-29	7.2	7	0.01	7.4	7.7	-0.01
30-34	7.7	7.1	0.02	8.1	8.7	-0.02
35-39	8.2	7.5	0.03	8.4	8.1	0.01
40-44	8.8	7.3	0.06	8.6	8.2	0.02
45-49	9.9	7.5	0.09	9.3	8	0.05
50-54	10.8	8.1	0.1	10.4	8.8	0.06
55-59	10.7	8.4	0.08	10	8.7	0.04
60-64	9.1	8.4	0.02	8.2	6.9	0.05
65-69	3.2	4.1	-0.05	2.4	2.2	0.01
70-74	1.6	2	-0.03	1.1	1.1	0
75-79	1.1	1.3	-0.02	0.6	0.5	0.01
80-84	2.4	1.9	0.04	0.9	1.1	-0.02
**Sex**						
Female	52.5	50.1	0.05	49.3	50.9	-0.03

Table S6.167. *Continued.* Selected baseline characteristics for US PharMetrics Plus for the medium-term risk of any of psychiatric and neuropsychiatric disorders

Characteristic	Before propensity score matching			After propensity score matching		
	Targets, %	Comparators, %	Standardized difference	Targets, %	Comparators, %	Standardized difference
**Medical history (general)**						
Acute respiratory disease	40.9	18.5	0.5	33	38.4	-0.11
Chronic liver disease	0.4	0.2	0.03	0.2	0.2	0
Chronic obstructive lung disease	2.3	1.2	0.08	0.7	0.7	-0.01
Crohn’s disease	0.3	0.2	0.01	0.2	0.3	-0.02
Diabetes mellitus	12.2	5.9	0.22	8.1	6.8	0.05
Gastroesophageal reflux disease	11	5.8	0.19	6.1	6.4	-0.01
Gastrointestinal hemorrhage	1.6	0.8	0.06	0.9	1	-0.01
Human immunodeficiency virus infection	0.3	0.2	0.03	0.2	0.3	-0.01
Hyperlipidemia	22.7	14.8	0.2	16.2	14.7	0.04
Hypertensive disorder	27.4	16.3	0.27	18.6	16.7	0.05
Lesion of liver	0.5	0.3	0.04	0.2	0.2	0
Obesity	12.4	5.8	0.23	6.9	6.1	0.03
Osteoarthritis	11.5	7	0.15	7.3	6.9	0.01
Pneumonia	10.6	1.2	0.41	3.1	2	0.07
Psoriasis	1.1	0.8	0.03	0.8	0.8	0.01
Renal impairment	5	1.9	0.17	2	1.8	0.01
Rheumatoid arthritis	1.1	0.6	0.06	0.7	0.6	0.01
Ulcerative colitis	0.3	0.2	0.02	0.3	0.2	0
Urinary tract infectious disease	5.5	2.5	0.15	3.3	3.5	-0.01
Viral hepatitis C	0.2	0.1	0.01	0.1	0.1	0

Table S6.167. *Continued.* Selected baseline characteristics for US PharMetrics Plus for the medium-term risk of any of psychiatric and neuropsychiatric disorders

Characteristic	Before propensity score matching			After propensity score matching		
	Targets, %	Comparators, %	Standardized diff	Targets, %	Comparators, %	Standardized diff
**Medical history (cardiovascular disease)**						
Atrial fibrillation	2.4	1.2	0.09	0.9	1	0
Cerebrovascular disease	1.2	0.7	0.05	0.5	0.5	0
Coronary arteriosclerosis	4	2.3	0.1	2	2	0
Heart disease	10.3	5.7	0.17	5.4	5.4	0
Heart failure	2.4	0.9	0.12	0.7	0.8	-0.01
Ischemic heart disease	2.5	1.1	0.1	1	1	0
Peripheral vascular disease	2.1	0.9	0.1	0.8	0.8	-0.01
Venous thrombosis	0.8	0.4	0.05	0.4	0.4	0
**Medical history (neoplasms)**						
Malignant lymphoma	0.3	0.2	0.03	0.2	0.2	0
Malignant neoplastic disease	3.9	3.1	0.04	2.7	2.8	-0.01
Malignant tumor of breast	0.7	0.6	0.01	0.5	0.5	0
Malignant tumor of colon	0.2	0.1	0.01	0.1	0.1	0
Malignant tumor of lung	0.2	0.1	0.01	0.1	0.1	-0.01
Primary malignant neoplasm of prostate	0.5	0.4	0.01	0.4	0.3	0.01
**Medication use**						
Agents acting on the renin-angiotensin system	11.9	8	0.13	8.6	7.6	0.04
Antibacterials for systemic use	39.7	25.1	0.31	31.9	33.6	-0.04
Antidepressants	12.7	9.1	0.11	3.5	3.8	-0.01
Antiepileptics	6.1	3.9	0.1	2.4	2.5	-0.01
Antiinflammatory and antirheumatic products	19.8	10.8	0.25	14.2	13.3	0.03
Antineoplastic agents	2.8	1.9	0.06	1.9	2.1	-0.01
Antipsoriatics	1.8	1.3	0.05	1.7	1.8	-0.01
Antithrombotic agents	4.9	2.5	0.12	2.4	2.4	0
Beta blocking agents	7	4.5	0.11	4.1	3.7	0.02

Table S6.167. *Continued.* Selected baseline characteristics for US PharMetrics Plus for the medium-term risk of any of psychiatric and neuropsychiatric disorders

Characteristic	Before propensity score matching			After propensity score matching		
	**Targets, %**	**Comparators, %**	**Standardized difference**	**Targets, %**	**Comparators, %**	**Standardized difference**
**Medication use**						
Calcium channel blockers	5.3	3.4	0.09	3.5	3.1	0.02
Diuretics	8.9	5.5	0.13	6	5.3	0.03
Drugs for acid-related disorders	12.9	7.5	0.18	8	8.2	-0.01
Drugs for obstructive airway diseases	24.6	14.2	0.27	18.2	18.8	-0.02
Drugs used in diabetes	7.1	3.9	0.14	4.8	4.1	0.04
Immunosuppressants	1.7	1	0.06	1.1	1.1	0
Lipid modifying agents	11.4	8	0.12	8.2	7.3	0.03
Opioids	13.1	7.8	0.18	8.3	8.6	-0.01
Psycholeptics	13.3	8.6	0.15	5.9	6.4	-0.02
Psychostimulants, agents used for ADHD and nootropics	3.6	2.7	0.05	1.1	1.1	-0.01

Table S6.168. Selected baseline characteristics for US PharMetrics Plus for the long-term risk of depression

**Characteristic**	**Before propensity score matching**			**After propensity score matching**		
	**Targets, %**	**Comparators, %**	**Standardized difference**	**Targets, %**	**Comparators, %**	**Standardized difference**
**Age group (years)**						
0-4	0.7	3.7	-0.2	1	2.4	-0.11
5-9	1.2	5.5	-0.24	1.7	3.3	-0.1
10-14	2.3	6.1	-0.19	3	4.3	-0.07
15-19	6.1	6.6	-0.02	7.1	7.9	-0.03
20-24	9.1	7.6	0.06	10	10.2	-0.01
25-29	7.2	6.9	0.01	6.2	6.6	-0.02
30-34	7.7	7.2	0.02	7.7	8.1	-0.01
35-39	8.2	7.4	0.03	8.3	8.4	0
40-44	8.8	7.3	0.05	8.9	8.6	0.01
45-49	9.9	7.4	0.09	9.9	8.5	0.05
50-54	10.8	8	0.1	11.1	9.6	0.05
55-59	10.7	8.6	0.07	10.9	9.2	0.06
60-64	9.1	8.4	0.02	8.5	7.4	0.04
65-69	3.2	4.1	-0.05	2.5	2.2	0.02
70-74	1.6	1.9	-0.03	1.2	1.2	0
75-79	1.1	1.3	-0.02	0.7	0.7	0
80-84	2.4	1.9	0.03	1.3	1.4	-0.01
**Sex**						
Female	52.5	50	0.05	49.6	50.9	-0.03

Table S6.168. *Continued.* Selected baseline characteristics for US PharMetrics Plus for the long-term risk of depression

Characteristic	Before propensity score matching			After propensity score matching		
	Targets, %	Comparators, %	Standardized difference	Targets, %	Comparators, %	Standardized difference
**Medical history (general)**						
Medical history: General						
Acute respiratory disease	40.9	18.6	0.5	35	40	-0.1
Attention deficit hyperactivity disorder	3.2	2.8	0.02	2.6	3.2	-0.03
Chronic obstructive lung disease	2.3	1.2	0.08	1.3	1.5	-0.02
Crohn’s disease	0.3	0.2	0.01	0.3	0.3	-0.01
Dementia	1.4	0.4	0.11	0.4	0.6	-0.03
Diabetes mellitus	12.2	5.9	0.22	9.4	8	0.05
Gastroesophageal reflux disease	11	5.9	0.19	8.5	8.6	0
Gastrointestinal hemorrhage	1.6	0.8	0.07	1.2	1.2	0
Human immunodeficiency virus infection	0.3	0.2	0.03	0.2	0.3	-0.02
Hyperlipidemia	22.7	14.8	0.2	19.3	17.7	0.04
Hypertensive disorder	27.4	16.4	0.27	22.5	20.5	0.05
Lesion of liver	0.5	0.2	0.04	0.3	0.3	0
Obesity	12.4	5.8	0.23	9.3	8.6	0.02
Osteoarthritis	11.5	7.1	0.15	9.3	9	0.01
Pneumonia	10.6	1.1	0.41	3.8	2.5	0.08
Psoriasis	1.1	0.8	0.03	1	1	0
Renal impairment	5	1.9	0.17	2.7	2.6	0
Rheumatoid arthritis	1.1	0.6	0.06	0.8	0.8	0
Ulcerative colitis	0.3	0.2	0.02	0.3	0.3	0
Urinary tract infectious disease	5.5	2.4	0.16	3.9	4	0
Viral hepatitis C	0.2	0.1	0.02	0.1	0.1	0

Table S6.168. *Continued.* Selected baseline characteristics for US PharMetrics Plus for the long-term risk of depression

Characteristic	Before propensity score matching			After propensity score matching		
	**Targets, %**	**Comparators, %**	**Standardized diff**	**Targets, %**	**Comparators, %**	**Standardized diff**
**Medical history (cardiovascular disease)**						
Atrial fibrillation	2.4	1.3	0.08	1.5	1.6	0
Cerebrovascular disease	1.2	0.7	0.05	0.8	0.9	-0.01
Coronary arteriosclerosis	4	2.2	0.1	2.8	2.8	0
Heart failure	2.4	0.9	0.12	1.2	1.4	-0.01
Ischemic heart disease	2.5	1.1	0.11	1.5	1.5	0
Peripheral vascular disease	2.1	0.9	0.1	1.1	1.3	-0.01
Pulmonary embolism	0.6	0.2	0.07	0.3	0.3	0
Venous thrombosis	0.8	0.4	0.05	0.6	0.5	0.01
**Medical history (neoplasms)**						
Malignant lymphoma	0.3	0.2	0.03	0.2	0.2	0
Malignant neoplasm of anorectum	0.1	0.1	0.01	0.1	0.1	-0.01
Malignant neoplastic disease	3.9	3.1	0.04	3.1	3.3	-0.01
Malignant tumor of breast	0.7	0.6	0.01	0.5	0.6	-0.01
Malignant tumor of lung	0.2	0.1	0.02	0.1	0.1	-0.01
Malignant tumor of urinary bladder	0.1	0.1	0.01	0.1	0.1	-0.01
Primary malignant neoplasm of prostate	0.5	0.4	0.01	0.4	0.4	0
**Medication use**						
Agents acting on the renin-angiotensin system	11.9	8	0.13	9.9	8.7	0.04
Antibacterials for systemic use	39.7	25.1	0.31	34.1	35.4	-0.03
Antidepressants	12.7	9.1	0.12	6.9	7.6	-0.03
Antiepileptics	6.1	3.9	0.1	3.8	4	-0.01
Antiinflammatory and antirheumatic products	19.8	10.9	0.25	16.1	15.6	0.01
Antineoplastic agents	2.8	2	0.06	2.3	2.5	-0.02
Antipsoriatics	1.8	1.2	0.05	1.7	1.8	-0.01
Antithrombotic agents	4.9	2.5	0.13	3.4	3.4	0
Beta blocking agents	7	4.6	0.1	5.3	5	0.01

Table S6.168. *Continued.* Selected baseline characteristics for US PharMetrics Plus for the long-term risk of depression

Characteristic	Before propensity score matching			After propensity score matching		
	**Targets, %**	**Comparators, %**	**Standardized difference**	**Targets, %**	**Comparators, %**	**Standardized difference**
**Medication use**						
Calcium channel blockers	5.3	3.5	0.09	4.2	3.8	0.02
Diuretics	8.9	5.5	0.13	7.1	6.4	0.03
Drugs for acid-related disorders	12.9	7.5	0.18	10.1	10.3	0
Drugs for obstructive airway diseases	24.6	14.1	0.27	20.6	21.2	-0.02
Drugs used in diabetes	7.1	3.8	0.15	5.4	4.6	0.04
Immunosuppressants	1.7	1.1	0.05	1.3	1.4	-0.01
Lipid modifying agents	11.4	8	0.11	9.4	8.4	0.04
Opioids	13.1	7.7	0.18	10.3	10.6	-0.01
Psycholeptics	13.3	8.5	0.16	9.8	10.4	-0.02
Psychostimulants, agents used for ADHD and nootropics	3.6	2.6	0.06	2.8	3.3	-0.03

Table S6.169. Selected baseline characteristics for US PharMetrics Plus for the long-term risk of anxiety disorders

Characteristic	Before propensity score matching			After propensity score matching		
	Targets, %	Comparators, %	Standardized difference	Targets, %	Comparators, %	Standardized difference
**Age group (years)**						
0-4	0.7	3.8	-0.21	1.1	2.7	-0.12
5-9	1.2	5.5	-0.24	1.7	3.4	-0.11
10-14	2.3	6	-0.19	3.1	4.4	-0.07
15-19	6.1	6.6	-0.02	7.2	7.9	-0.03
20-24	9.1	7.5	0.06	9.8	9.9	0
25-29	7.2	7	0.01	6.1	6.3	-0.01
30-34	7.7	7.1	0.02	7.4	7.8	-0.02
35-39	8.2	7.5	0.03	8	8.1	0
40-44	8.8	7.3	0.05	8.6	8.3	0.01
45-49	9.9	7.4	0.09	9.9	8.5	0.05
50-54	10.8	7.9	0.1	11.1	9.7	0.05
55-59	10.7	8.5	0.07	11	9.5	0.05
60-64	9.1	8.5	0.02	8.8	7.6	0.04
65-69	3.2	4.2	-0.05	2.6	2.4	0.02
75-79	1.1	1.3	-0.02	0.8	0.7	0.01
80-84	2.4	1.9	0.03	1.5	1.5	0
**Sex**						
Female	52.5	50	0.05	48	49.1	-0.02

Table S6.169. *Continued.* Selected baseline characteristics for US PharMetrics Plus for the long-term risk of anxiety disorders

Characteristic	Before propensity score matching			After propensity score matching		
	Targets, %	Comparators, %	Standardized difference	Targets, %	Comparators, %	Standardized difference
**Medical history (general)**						
Acute respiratory disease	40.9	18.5	0.51	34.1	39.2	-0.11
Attention deficit hyperactivity disorder	3.2	2.9	0.02	2.2	2.6	-0.03
Chronic liver disease	0.4	0.2	0.04	0.3	0.3	0
Chronic obstructive lung disease	2.3	1.2	0.09	1.3	1.5	-0.02
Crohn's disease	0.3	0.2	0.02	0.2	0.3	-0.01
Dementia	1.4	0.4	0.11	0.6	0.7	-0.02
Depressive disorder	10.3	6.9	0.12	4	4.3	-0.02
Diabetes mellitus	12.2	5.9	0.22	9.9	8.6	0.04
Gastroesophageal reflux disease	11	5.8	0.19	7.9	8	0
Gastrointestinal hemorrhage	1.6	0.9	0.06	1.1	1.2	-0.01
Human immunodeficiency virus infection	0.3	0.2	0.03	0.2	0.4	-0.02
Hyperlipidemia	22.7	14.8	0.2	19.5	17.9	0.04
Hypertensive disorder	27.4	16.3	0.27	22.6	20.5	0.05
Lesion of liver	0.5	0.2	0.04	0.3	0.3	0
Obesity	12.4	5.8	0.23	9.3	8.5	0.03
Osteoarthritis	11.5	7	0.15	9.4	8.9	0.01
Pneumonia	10.6	1.1	0.41	3.8	2.5	0.07
Psoriasis	1.1	0.8	0.03	0.9	1	0
Renal impairment	5	1.9	0.17	2.8	2.8	0
Rheumatoid arthritis	1.1	0.6	0.06	0.8	0.8	0
Ulcerative colitis	0.3	0.2	0.02	0.3	0.3	0
Urinary tract infectious disease	5.5	2.4	0.16	3.8	3.9	-0.01
Viral hepatitis C	0.2	0.1	0.02	0.1	0.1	-0.01

Table S6.169. *Continued.* Selected baseline characteristics for US PharMetrics Plus for the long-term risk of anxiety disorders

Characteristic	Before propensity score matching			After propensity score matching		
	Targets, %	Comparators, %	Standardized diff	Targets, %	Comparators, %	Standardized diff
**Medical history (cardiovascular disease)**						
Atrial fibrillation	2.4	1.2	0.09	1.6	1.6	0
Cerebrovascular disease	1.2	0.7	0.05	0.8	0.9	-0.01
Coronary arteriosclerosis	4	2.3	0.1	3	2.9	0
Heart disease	10.3	5.7	0.17	7.5	7.5	0
Heart failure	2.4	0.9	0.12	1.4	1.5	-0.01
Ischemic heart disease	2.5	1.1	0.11	1.6	1.6	0
Peripheral vascular disease	2.1	0.9	0.1	1.3	1.4	-0.01
Pulmonary embolism	0.6	0.2	0.07	0.3	0.3	0.01
Venous thrombosis	0.8	0.4	0.05	0.6	0.5	0.01
**Medical history (neoplasms)**						
Malignant lymphoma	0.3	0.2	0.02	0.2	0.2	0
Malignant neoplasm of anorectum	0.1	0.1	0	0.1	0.1	-0.01
Malignant neoplastic disease	3.9	3.1	0.04	3.2	3.3	0
Malignant tumor of breast	0.7	0.6	0.01	0.5	0.6	-0.01
Malignant tumor of colon	0.2	0.1	0.01	0.1	0.1	0
Malignant tumor of lung	0.2	0.1	0.02	0.1	0.1	-0.01
Malignant tumor of urinary bladder	0.1	0.1	0.01	0.1	0.1	-0.01
Primary malignant neoplasm of prostate	0.5	0.4	0.01	0.4	0.4	0.01
**Medication use**						
Agents acting on the renin-angiotensin system	11.9	8	0.13	10	8.9	0.04
Antibacterials for systemic use	39.7	25	0.32	33.1	34.7	-0.03
Antidepressants	12.7	9	0.12	5.7	6.3	-0.02
Antiepileptics	6.1	3.8	0.1	3.5	3.5	0
Antiinflammatory and antirheumatic products	19.8	10.8	0.25	15.5	15.1	0.01
Antineoplastic agents	2.8	1.9	0.06	2.2	2.3	0
Antipsoriatics	1.8	1.2	0.05	1.6	1.7	-0.01

Table S6.169. *Continued.* Selected baseline characteristics for US PharMetrics Plus for the long-term risk of anxiety disorders

Characteristic	Before propensity score matching			After propensity score matching		
	Targets, %	Comparators, %	Standardized difference	Targets, %	Comparators, %	Standardized difference
**Medication use**						
Antithrombotic agents	4.9	2.5	0.13	3.4	3.4	0
Beta blocking agents	7	4.6	0.1	5.1	4.8	0.02
Calcium channel blockers	5.3	3.5	0.09	4.2	3.8	0.02
Diuretics	8.9	5.5	0.13	7.1	6.3	0.03
Drugs for acid-related disorders	12.9	7.5	0.18	9.6	9.8	0
Drugs for obstructive airway diseases	24.6	14.2	0.27	19.8	20.4	-0.02
Drugs used in diabetes	7.1	3.8	0.15	5.7	4.8	0.04
Immunosuppressants	1.7	1.1	0.05	1.3	1.3	0
Lipid modifying agents	11.4	7.9	0.12	9.7	8.6	0.04
Opioids	13.1	7.8	0.17	9.9	10.2	-0.01
Psycholeptics	13.3	8.5	0.16	7.8	8.3	-0.02
Psychostimulants, agents used for ADHD and nootropics	3.6	2.7	0.05	2.5	2.9	-0.03

Table S6.170. Selected baseline characteristics for US PharMetrics Plus for the long-term risk of alcohol misuse or dependence

Characteristic	Before propensity score matching			After propensity score matching		
	Targets, %	Comparators, %	Standardized difference	Targets, %	Comparators, %	Standardized difference
**Age group (years)**						
0-4	0.7	3.7	-0.21	0.9	2	-0.1
5-9	1.2	5.5	-0.24	1.5	2.8	-0.09
10-14	2.3	6.1	-0.19	2.8	3.9	-0.06
15-19	6.1	6.6	-0.02	7.2	8	-0.03
20-24	9.1	7.6	0.06	10	10.8	-0.03
25-29	7.2	6.8	0.01	6.2	6.6	-0.02
30-34	7.7	7.2	0.02	7.7	8.1	-0.02
35-39	8.2	7.4	0.03	8.3	8.4	0
40-44	8.8	7.3	0.05	9	8.7	0.01
45-49	9.9	7.3	0.09	9.9	8.6	0.05
50-54	10.8	8	0.1	11	9.5	0.05
55-59	10.7	8.6	0.07	10.7	9.2	0.05
60-64	9.1	8.5	0.02	8.4	7.4	0.04
65-69	3.2	4.1	-0.05	2.5	2.2	0.02
70-74	1.6	2	-0.03	1.4	1.3	0.01
75-79	1.1	1.3	-0.02	0.8	0.8	0.01
80-84	2.4	1.9	0.04	1.7	1.6	0
**Sex**						
Female	52.5	50.2	0.05	52.9	54.4	-0.03

Table S6.170. *Continued.* Selected baseline characteristics for US PharMetrics Plus for the long-term risk of alcohol misuse or dependence

Characteristic	Before propensity score matching			After propensity score matching		
	Targets, %	Comparators, %	Standardized difference	Targets, %	Comparators, %	Standardized difference
**Medical history (general)**						
Acute respiratory disease	40.9	18.4	0.51	36.5	41.2	-0.1
Attention deficit hyperactivity disorder	3.2	2.8	0.02	3.4	4	-0.03
Chronic liver disease	0.4	0.2	0.03	0.3	0.3	0
Chronic obstructive lung disease	2.3	1.2	0.09	1.6	1.8	-0.01
Crohn’s disease	0.3	0.2	0.02	0.3	0.4	-0.02
Dementia	1.4	0.4	0.11	0.8	0.9	-0.01
Depressive disorder	10.3	6.9	0.12	9.2	10.6	-0.05
Diabetes mellitus	12.2	5.9	0.22	9.9	8.6	0.04
Gastroesophageal reflux disease	11	5.8	0.19	9.8	10	-0.01
Gastrointestinal hemorrhage	1.6	0.8	0.07	1.3	1.5	-0.01
Human immunodeficiency virus infection	0.3	0.1	0.04	0.3	0.4	-0.02
Hyperlipidemia	22.7	14.8	0.2	20.3	18.5	0.04
Hypertensive disorder	27.4	16.3	0.27	23.8	21.6	0.05
Lesion of liver	0.5	0.3	0.03	0.3	0.3	-0.01
Obesity	12.4	5.8	0.23	10.4	9.8	0.02
Osteoarthritis	11.5	7	0.15	10.5	10.2	0.01
Pneumonia	10.6	1.1	0.41	4.2	2.7	0.08
Psoriasis	1.1	0.8	0.03	1	1.1	0
Renal impairment	5	1.9	0.17	3	2.9	0.01
Rheumatoid arthritis	1.1	0.5	0.06	1	0.9	0.01
Ulcerative colitis	0.3	0.2	0.02	0.3	0.3	0
Urinary tract infectious disease	5.5	2.5	0.15	4.6	4.7	0
Viral hepatitis C	0.2	0.1	0.02	0.1	0.1	0

Table S6.170. *Continued.* Selected baseline characteristics for US PharMetrics Plus for the long-term risk of alcohol misuse or dependence

Characteristic	Before propensity score matching			After propensity score matching		
	Targets, %	Comparators, %	Standardized difference	Targets, %	Comparators, %	Standardized difference
**Medical history (cardiovascular disease)**						
Atrial fibrillation	2.4	1.2	0.09	1.7	1.7	0
Cerebrovascular disease	1.2	0.7	0.05	0.9	1.1	-0.02
Coronary arteriosclerosis	4	2.3	0.1	3.1	3	0.01
Heart disease	10.3	5.7	0.17	8.2	8	0.01
Heart failure	2.4	0.9	0.12	1.5	1.6	-0.01
Ischemic heart disease	2.5	1.1	0.1	1.7	1.7	0
Peripheral vascular disease	2.1	0.9	0.1	1.5	1.5	0
Pulmonary embolism	0.6	0.2	0.07	0.4	0.4	0
Venous thrombosis	0.8	0.4	0.06	0.6	0.6	0.01
**Medical history (neoplasms)**						
Malignant neoplasm of anorectum	0.1	0.1	0	0.1	0.1	-0.01
Malignant tumor of breast	0.7	0.6	0.01	0.6	0.7	0
Malignant tumor of colon	0.2	0.1	0.01	0.1	0.1	0
Malignant tumor of lung	0.2	0.1	0.01	0.1	0.1	-0.01
Malignant tumor of urinary bladder	0.1	0.1	0	0.1	0.1	0
Primary malignant neoplasm of prostate	0.5	0.4	0.01	0.4	0.4	0
**Medication use**						
Agents acting on the renin-angiotensin system	11.9	8	0.13	10.2	8.9	0.04
Antibacterials for systemic use	39.7	25.1	0.32	35.5	36.9	-0.03
Antidepressants	12.7	9.1	0.12	11.5	12.8	-0.04
Antiepileptics	6.1	3.9	0.1	5	5.4	-0.02
Antiinflammatory and antirheumatic products	19.8	10.9	0.25	17.3	17.1	0.01
Antineoplastic agents	2.8	1.9	0.06	2.5	2.7	-0.01
Antipsoriatics	1.8	1.3	0.05	1.8	1.9	-0.01
Antithrombotic agents	4.9	2.5	0.13	3.8	3.9	-0.01

Table S6.170. *Continued.* Selected baseline characteristics for US PharMetrics Plus for the long-term risk of alcohol misuse or dependence

Characteristic	Before propensity score matching			After propensity score matching		
	Targets, %	Comparators, %	Standardized difference	Targets, %	Comparators, %	Standardized difference
**Medication use**						
Beta blocking agents	7	4.6	0.1	5.8	5.4	0.02
Calcium channel blockers	5.3	3.5	0.09	4.3	3.9	0.02
Diuretics	8.9	5.5	0.13	7.5	6.7	0.03
Drugs for acid-related disorders	12.9	7.5	0.18	11.3	11.6	-0.01
Drugs for obstructive airway diseases	24.6	14.2	0.27	21.8	22.5	-0.02
Drugs used in diabetes	7.1	3.8	0.14	5.8	5	0.04
Immunosuppressants	1.7	1.1	0.05	1.5	1.5	-0.01
Lipid modifying agents	11.4	8	0.12	9.8	8.8	0.04
Opioids	13.1	7.8	0.17	11.5	11.8	-0.01
Psycholeptics	13.3	8.6	0.15	12	12.9	-0.03
Psychostimulants, agents used for ADHD and nootropics	3.6	2.6	0.06	3.5	4.1	-0.03

Table S6.171. Selected baseline characteristics for US PharMetrics Plus for the long-term risk of substance misuse or dependence

**Characteristic**	**Before propensity score matching**			**After propensity score matching**		
	**Targets, %**	**Comparators, %**	**Standardized difference**	**Targets, %**	**Comparators, %**	**Standardized difference**
**Age group (years)**						
0-4	0.7	3.7	-0.2	0.9	2.3	-0.11
5-9	1.2	5.5	-0.24	1.6	3	-0.1
10-14	2.3	6.1	-0.19	3	4.2	-0.07
15-19	6.1	6.6	-0.02	7.3	8.4	-0.04
20-24	9.1	7.6	0.06	10	10.6	-0.02
25-29	7.2	7	0.01	6.2	6.6	-0.02
30-34	7.7	7.2	0.02	7.7	8.3	-0.02
35-39	8.2	7.4	0.03	8.3	8.3	0
40-44	8.8	7.4	0.05	8.9	8.3	0.02
45-49	9.9	7.5	0.09	9.8	8.5	0.04
50-54	10.8	7.9	0.1	11	9.4	0.05
55-59	10.7	8.6	0.07	10.6	9	0.06
60-64	9.1	8.4	0.02	8.3	7.2	0.04
65-69	3.2	4.1	-0.05	2.5	2.2	0.02
70-74	1.6	1.9	-0.03	1.4	1.3	0.01
75-79	1.1	1.3	-0.02	0.8	0.7	0.01
80-84	2.4	1.9	0.03	1.7	1.6	0
**Sex**						
Female	52.5	50.1	0.05	53.4	54.8	-0.03

Table S6.171. *Continued.* Selected baseline characteristics for US PharMetrics Plus for the long-term risk of substance misuse or dependence

Characteristic	Before propensity score matching			After propensity score matching		
	Targets, %	Comparators, %	Standardized difference	Targets, %	Comparators, %	Standardized difference
**Medical history (general)**						
Acute respiratory disease	40.9	18.7	0.5	36	40.8	-0.1
Attention deficit hyperactivity disorder	3.2	3	0.02	3.2	3.9	-0.04
Chronic liver disease	0.4	0.2	0.03	0.2	0.2	0
Chronic obstructive lung disease	2.3	1.2	0.09	1.2	1.1	0
Crohn’s disease	0.3	0.2	0.01	0.3	0.4	-0.01
Dementia	1.4	0.4	0.11	0.8	0.9	-0.02
Depressive disorder	10.3	6.8	0.12	8.6	9.6	-0.04
Diabetes mellitus	12.2	5.9	0.22	9.6	8.2	0.05
Gastroesophageal reflux disease	11	5.9	0.19	9.3	9.4	0
Gastrointestinal hemorrhage	1.6	0.8	0.07	1.2	1.3	-0.01
Human immunodeficiency virus infection	0.3	0.2	0.03	0.2	0.3	-0.02
Hyperlipidemia	22.7	14.8	0.2	19.8	17.8	0.05
Hypertensive disorder	27.4	16.4	0.27	23.1	20.4	0.07
Lesion of liver	0.5	0.2	0.04	0.2	0.2	0
Obesity	12.4	5.8	0.23	10	9.3	0.02
Osteoarthritis	11.5	7	0.16	10.1	9.5	0.02
Pneumonia	10.6	1.1	0.41	3.9	2.4	0.09
Psoriasis	1.1	0.8	0.03	1	1.1	-0.01
Renal impairment	5	1.9	0.17	2.8	2.7	0.01
Rheumatoid arthritis	1.1	0.6	0.06	0.9	0.9	0
Urinary tract infectious disease	5.5	2.5	0.15	4.4	4.4	0
Viral hepatitis C	0.2	0.1	0.01	0.1	0.1	0

Table S6.171. *Continued.* Selected baseline characteristics for US PharMetrics Plus for the long-term risk of substance misuse or dependence

Characteristic	Before propensity score matching			After propensity score matching		
	Targets, %	Comparators, %	Standardized diff	Targets, %	Comparators, %	Standardized diff
**Medical history (cardiovascular disease)**						
Atrial fibrillation	2.4	1.2	0.09	1.6	1.6	0
Cerebrovascular disease	1.2	0.7	0.05	0.8	0.9	-0.01
Coronary arteriosclerosis	4	2.2	0.1	2.9	2.6	0.01
Heart disease	10.3	5.6	0.17	7.7	7.4	0.01
Heart failure	2.4	0.9	0.12	1.4	1.4	0
Ischemic heart disease	2.5	1.1	0.11	1.5	1.4	0.01
Peripheral vascular disease	2.1	0.9	0.1	1.4	1.3	0.01
Pulmonary embolism	0.6	0.2	0.06	0.3	0.3	0.01
Venous thrombosis	0.8	0.4	0.06	0.6	0.5	0.01
**Medical history (neoplasms)**						
Malignant lymphoma	0.3	0.2	0.02	0.2	0.2	0
Malignant neoplasm of anorectum	0.1	0.1	0.01	0.1	0.1	-0.01
Malignant neoplastic disease	3.9	3.1	0.04	3.3	3.3	0
Malignant tumor of breast	0.7	0.6	0.01	0.6	0.7	0
Malignant tumor of colon	0.2	0.1	0.01	0.1	0.1	0
Malignant tumor of lung	0.2	0.1	0.02	0.1	0.1	-0.01
Malignant tumor of urinary bladder	0.1	0.1	0.01	0.1	0.1	-0.01
Primary malignant neoplasm of prostate	0.5	0.4	0.01	0.4	0.4	0.01
**Medication use**						
Agents acting on the renin-angiotensin system	11.9	8	0.13	9.9	8.4	0.05
Antibacterials for systemic use	39.7	25.1	0.32	34.8	35.9	-0.02
Antidepressants	12.7	9.1	0.12	10.9	11.9	-0.03
Antiepileptics	6.1	3.9	0.1	4.7	4.8	-0.01
Antiinflammatory and antirheumatic products	19.8	10.8	0.25	16.5	15.9	0.02
Antineoplastic agents	2.8	1.9	0.06	2.5	2.7	-0.01
Antipsoriatics	1.8	1.3	0.05	1.9	2	-0.01

Table S6.171. *Continued.* Selected baseline characteristics for US PharMetrics Plus for the long-term risk of substance misuse or dependence

Characteristic	Before propensity score matching			After propensity score matching		
	Targets, %	Comparators, %	Standardized difference	Targets, %	Comparators, %	Standardized difference
**Medication use**						
Antithrombotic agents	4.9	2.5	0.13	3.4	3.4	0
Beta blocking agents	7	4.6	0.1	5.5	5.1	0.02
Calcium channel blockers	5.3	3.5	0.09	4.2	3.7	0.03
Diuretics	8.9	5.5	0.13	7.3	6.3	0.04
Drugs for acid-related disorders	12.9	7.5	0.18	10.7	10.7	0
Drugs for obstructive airway diseases	24.6	14.2	0.26	21.3	21.9	-0.01
Drugs used in diabetes	7.1	3.7	0.15	5.6	4.7	0.04
Immunosuppressants	1.7	1.1	0.05	1.4	1.5	-0.01
Lipid modifying agents	11.4	7.9	0.12	9.5	8.3	0.04
Opioids	13.1	7.8	0.18	10.7	10.8	0
Psycholeptics	13.3	8.6	0.15	11.3	12	-0.02
Psychostimulants, agents used for ADHD and nootropics	3.6	2.7	0.05	3.3	4	-0.04

Table S6.172. Selected baseline characteristics for US PharMetrics Plus for the long-term risk of bipolar disorders

Characteristic	Before propensity score matching			After propensity score matching		
	**Targets, %**	**Comparators, %**	**Standardized difference**	**Targets, %**	**Comparators, %**	**Standardized difference**
**Age group (years)**						
0-4	0.7	3.7	-0.21	0.9	2	-0.1
5-9	1.2	5.5	-0.24	1.5	2.8	-0.09
10-14	2.3	6.1	-0.19	2.8	4	-0.07
15-19	6.1	6.6	-0.02	7.1	8	-0.03
20-24	9.1	7.5	0.06	10	10.6	-0.02
25-29	7.2	7	0.01	6.2	6.6	-0.02
30-34	7.7	7.3	0.02	7.6	8.1	-0.02
35-39	8.2	7.4	0.03	8.3	8.4	0
40-44	8.8	7.4	0.05	9	8.6	0.01
45-49	9.9	7.4	0.09	10	8.6	0.05
50-54	10.8	8	0.1	11	9.6	0.05
55-59	10.7	8.6	0.07	10.8	9.2	0.05
60-64	9.1	8.3	0.03	8.5	7.4	0.04
65-69	3.2	4.1	-0.05	2.5	2.3	0.02
70-74	1.6	2	-0.03	1.4	1.3	0
75-79	1.1	1.3	-0.02	0.8	0.8	0.01
80-84	2.4	1.9	0.03	1.7	1.6	0
**Sex**						
Female	52.5	50.2	0.05	52.5	53.9	-0.03

Table S6.172. *Continued.* Selected baseline characteristics for US PharMetrics Plus for the long-term risk of bipolar disorders

Characteristic	Before propensity score matching			After propensity score matching		
	Targets, %	Comparators, %	Standardized difference	Targets, %	Comparators, %	Standardized difference
**Medical history (general)**						
Acute respiratory disease	40.9	18.5	0.5	36.3	41.2	-0.1
Attention deficit hyperactivity disorder	3.2	2.9	0.02	3.3	3.9	-0.04
Chronic liver disease	0.4	0.2	0.04	0.3	0.3	0
Chronic obstructive lung disease	2.3	1.2	0.09	1.6	1.8	-0.02
Crohn’s disease	0.3	0.2	0.01	0.3	0.4	-0.02
Dementia	1.4	0.4	0.11	0.8	0.9	-0.02
Depressive disorder	10.3	6.9	0.12	9	10.4	-0.05
Diabetes mellitus	12.2	5.9	0.22	9.9	8.5	0.05
Gastroesophageal reflux disease	11	5.8	0.19	9.8	9.9	0
Gastrointestinal hemorrhage	1.6	0.8	0.07	1.3	1.5	-0.02
Human immunodeficiency virus infection	0.3	0.2	0.03	0.3	0.4	-0.02
Hyperlipidemia	22.7	14.7	0.2	20.3	18.4	0.05
Hypertensive disorder	27.4	16.3	0.27	23.9	21.9	0.05
Lesion of liver	0.5	0.2	0.04	0.3	0.4	-0.01
Obesity	12.4	5.8	0.23	10.3	9.6	0.02
Osteoarthritis	11.5	7	0.16	10.5	10.1	0.01
Pneumonia	10.6	1.1	0.41	4.1	2.7	0.08
Psoriasis	1.1	0.8	0.03	1	1.1	0
Renal impairment	5	1.9	0.17	3	3	0
Rheumatoid arthritis	1.1	0.5	0.06	1	0.9	0.01
Ulcerative colitis	0.3	0.2	0.03	0.3	0.3	0
Urinary tract infectious disease	5.5	2.5	0.15	4.5	4.6	0
Viral hepatitis C	0.2	0.1	0.02	0.1	0.1	-0.01

Table S6.172. *Continued.* Selected baseline characteristics for US PharMetrics Plus for the long-term risk of bipolar disorders

Characteristic	Before propensity score matching			After propensity score matching		
	Targets, %	Comparators, %	Standardized difference	Targets, %	Comparators, %	Standardized difference
**Medical history (cardiovascular disease)**						
Atrial fibrillation	2.4	1.2	0.09	1.7	1.7	0
Cerebrovascular disease	1.2	0.7	0.06	0.9	1.1	-0.01
Coronary arteriosclerosis	4	2.2	0.1	3.1	3	0.01
Heart disease	10.3	5.7	0.17	8.2	8.1	0
Heart failure	2.4	0.9	0.12	1.5	1.6	0
Peripheral vascular disease	2.1	0.9	0.1	1.4	1.5	0
Pulmonary embolism	0.6	0.2	0.06	0.4	0.4	0
Venous thrombosis	0.8	0.4	0.06	0.7	0.6	0
**Medical history (neoplasms)**						
Malignant neoplasm of anorectum	0.1	0.1	0.01	0.1	0.1	-0.01
Malignant neoplastic disease	3.9	3.1	0.04	3.4	3.5	0
Malignant tumor of breast	0.7	0.6	0.01	0.6	0.7	-0.01
Malignant tumor of colon	0.2	0.1	0.01	0.1	0.1	0
Malignant tumor of lung	0.2	0.1	0.02	0.1	0.1	-0.01
Malignant tumor of urinary bladder	0.1	0.1	0.01	0.1	0.1	-0.01
Primary malignant neoplasm of prostate	0.5	0.4	0.01	0.4	0.4	0.01
**Medication use**						
Agents acting on the renin-angiotensin system	11.9	8	0.13	10.3	9	0.04
Antibacterials for systemic use	39.7	25.1	0.31	35.4	36.7	-0.03
Antidepressants	12.7	9	0.12	11.4	12.5	-0.04
Antiepileptics	6.1	3.8	0.11	4.8	5.2	-0.02
Antiinflammatory and antirheumatic products	19.8	10.9	0.25	17.3	17	0.01
Antineoplastic agents	2.8	1.9	0.06	2.5	2.7	-0.01
Antipsoriatics	1.8	1.2	0.05	1.8	2	-0.01
Antithrombotic agents	4.9	2.5	0.13	3.8	3.9	-0.01

Table S6.172. *Continued.* Selected baseline characteristics for US PharMetrics Plus for the long-term risk of bipolar disorders

Characteristic	Before propensity score matching			After propensity score matching		
	**Targets, %**	**Comparators, %**	**Standardized difference**	**Targets, %**	**Comparators, %**	**Standardized difference**
**Medication use**						
Beta blocking agents	7	4.5	0.11	5.8	5.5	0.01
Calcium channel blockers	5.3	3.5	0.09	4.4	4	0.02
Diuretics	8.9	5.4	0.13	7.5	6.8	0.03
Drugs for acid-related disorders	12.9	7.5	0.18	11.3	11.4	0
Drugs for obstructive airway diseases	24.6	14.2	0.27	21.8	22.5	-0.02
Drugs used in diabetes	7.1	3.8	0.15	5.8	4.9	0.04
Immunosuppressants	1.7	1	0.05	1.5	1.5	-0.01
Lipid modifying agents	11.4	7.9	0.12	9.8	8.8	0.03
Opioids	13.1	7.7	0.18	11.5	11.9	-0.01
Psycholeptics	13.3	8.5	0.15	11.8	12.8	-0.03
Psychostimulants, agents used for ADHD and nootropics	3.6	2.7	0.05	3.4	4	-0.03

Table S6.173. Selected baseline characteristics for US PharMetrics Plus for the long-term risk of psychoses

Characteristic	Before propensity score matching			After propensity score matching		
	Targets, %	Comparators, %	Standardized difference	Targets, %	Comparators, %	Standardized difference
**Age group (years)**						
0-4	0.7	3.7	-0.2	0.9	2	-0.1
5-9	1.2	5.5	-0.24	1.5	2.8	-0.09
10-14	2.3	6.1	-0.19	2.8	3.8	-0.06
15-19	6.1	6.7	-0.03	7.2	8	-0.03
20-24	9.1	7.6	0.06	10.1	10.6	-0.02
25-29	7.2	6.9	0.01	6.2	6.6	-0.02
30-34	7.7	7.2	0.02	7.7	8.2	-0.02
35-39	8.2	7.4	0.03	8.3	8.4	0
40-44	8.8	7.3	0.06	9	8.6	0.01
45-49	9.9	7.4	0.09	10	8.7	0.05
50-54	10.8	7.9	0.1	11	9.6	0.05
55-59	10.7	8.6	0.07	10.8	9.2	0.05
60-64	9.1	8.5	0.02	8.4	7.4	0.04
65-69	3.2	4.1	-0.05	2.5	2.3	0.02
70-74	1.6	2	-0.03	1.4	1.3	0
75-79	1.1	1.2	-0.02	0.8	0.8	0
80-84	2.4	1.9	0.03	1.6	1.6	0
**Sex**						
Female	52.5	50.1	0.05	52.6	54.2	-0.03

Table S6.173. *Continued.* Selected baseline characteristics for US PharMetrics Plus for the long-term risk of psychoses

Characteristic	Before propensity score matching			After propensity score matching		
	Targets, %	Comparators, %	Standardized difference	Targets, %	Comparators, %	Standardized difference
**Medical history (general)**						
Acute respiratory disease	40.9	18.5	0.5	36.5	41.5	-0.1
Attention deficit hyperactivity disorder	3.2	2.9	0.02	3.4	4.1	-0.04
Chronic liver disease	0.4	0.2	0.04	0.3	0.3	0
Chronic obstructive lung disease	2.3	1.2	0.09	1.6	1.9	-0.02
Crohn’s disease	0.3	0.2	0.01	0.3	0.4	-0.01
Dementia	1.4	0.4	0.1	0.7	0.9	-0.02
Depressive disorder	10.3	6.9	0.12	9.4	10.8	-0.05
Diabetes mellitus	12.2	5.9	0.22	9.9	8.6	0.05
Gastroesophageal reflux disease	11	5.8	0.19	9.8	10.1	-0.01
Gastrointestinal hemorrhage	1.6	0.8	0.07	1.3	1.5	-0.02
Human immunodeficiency virus infection	0.3	0.2	0.03	0.3	0.4	-0.02
Hyperlipidemia	22.7	14.8	0.2	20.3	18.6	0.04
Hypertensive disorder	27.4	16.3	0.27	23.9	21.9	0.05
Lesion of liver	0.5	0.2	0.04	0.3	0.4	-0.01
Obesity	12.4	5.8	0.23	10.4	9.9	0.02
Osteoarthritis	11.5	7	0.15	10.5	10.2	0.01
Pneumonia	10.6	1.1	0.41	4.2	2.8	0.07
Psoriasis	1.1	0.8	0.03	1	1.1	-0.01
Renal impairment	5	1.8	0.18	3	2.9	0.01
Rheumatoid arthritis	1.1	0.5	0.06	1	0.9	0.01
Ulcerative colitis	0.3	0.2	0.02	0.3	0.4	0
Urinary tract infectious disease	5.5	2.5	0.16	4.5	4.6	0
Viral hepatitis C	0.2	0.1	0.02	0.1	0.2	-0.01

Table S6.173. *Continued.* Selected baseline characteristics for US PharMetrics Plus for the long-term risk of psychoses

**Characteristic**	**Before propensity score matching**			**After propensity score matching**		
	**Targets, %**	**Comparators, %**	**Standardized difference**	**Targets, %**	**Comparators, %**	**Standardized difference**
**Medical history (cardiovascular disease)**						
Medical history: Cardiovascular disease						
Atrial fibrillation	2.4	1.3	0.08	1.7	1.7	0
Cerebrovascular disease	1.2	0.7	0.05	0.9	1	-0.01
Coronary arteriosclerosis	4	2.3	0.1	3.1	3	0.01
Heart disease	10.3	5.7	0.17	8.2	8.2	0
Heart failure	2.4	0.9	0.12	1.5	1.7	-0.01
Peripheral vascular disease	2.1	0.9	0.1	1.4	1.4	0
Pulmonary embolism	0.6	0.2	0.07	0.4	0.4	0
Venous thrombosis	0.8	0.4	0.06	0.7	0.6	0
**Medical history (neoplasms)**						
Malignant lymphoma	0.3	0.2	0.03	0.3	0.2	0
Malignant neoplasm of anorectum	0.1	0.1	0	0.1	0.1	-0.01
Malignant tumor of breast	0.7	0.5	0.01	0.6	0.7	-0.01
Malignant tumor of colon	0.2	0.1	0.01	0.1	0.1	0
Malignant tumor of lung	0.2	0.1	0.02	0.1	0.1	-0.01
Malignant tumor of urinary bladder	0.1	0.1	0.01	0.1	0.1	-0.01
Primary malignant neoplasm of prostate	0.5	0.4	0.02	0.4	0.4	0.01
**Medication use**						
Agents acting on the renin-angiotensin system	11.9	8	0.13	10.2	9.1	0.04
Antibacterials for systemic use	39.7	25.1	0.31	35.5	37	-0.03
Antidepressants	12.7	9	0.12	11.6	13	-0.04
Antiepileptics	6.1	3.8	0.1	5.1	5.5	-0.02
Antiinflammatory and antirheumatic products	19.8	10.8	0.25	17.4	17.1	0.01
Antineoplastic agents	2.8	1.9	0.06	2.5	2.7	-0.01
Antipsoriatics	1.8	1.3	0.05	1.8	2	-0.01

Table S6.173. *Continued.* Selected baseline characteristics for US PharMetrics Plus for the long-term risk of psychoses

Characteristic	Before propensity score matching			After propensity score matching		
	**Targets, %**	**Comparators, %**	**Standardized difference**	**Targets, %**	**Comparators, %**	**Standardized difference**
**Medication use**						
Antithrombotic agents	4.9	2.5	0.13	3.8	3.9	0
Beta blocking agents	7	4.6	0.1	5.8	5.5	0.01
Calcium channel blockers	5.3	3.4	0.09	4.4	3.9	0.03
Diuretics	8.9	5.4	0.14	7.5	6.8	0.03
Drugs for acid-related disorders	12.9	7.5	0.18	11.4	11.7	-0.01
Drugs for obstructive airway diseases	24.6	14	0.27	21.9	22.8	-0.02
Drugs used in diabetes	7.1	3.7	0.15	5.8	4.9	0.04
Immunosuppressants	1.7	1	0.06	1.5	1.6	-0.01
Lipid modifying agents	11.4	7.9	0.12	9.8	8.7	0.04
Opioids	13.1	7.6	0.18	11.6	12.1	-0.01
Psycholeptics	13.3	8.6	0.15	12.1	13.1	-0.03
Psychostimulants, agents used for ADHD and nootropics	3.6	2.7	0.05	3.5	4.2	-0.04

Table S6.174. Selected baseline characteristics for US PharMetrics Plus for the long-term risk of personality disorders

Characteristic	Before propensity score matching			After propensity score matching		
	**Targets, %**	**Comparators, %**	**Standardized difference**	**Targets, %**	**Comparators, %**	**Standardized difference**
**Age group (years)**						
0-4	0.7	3.7	-0.2	0.9	2.1	-0.1
5-9	1.2	5.5	-0.24	1.4	2.8	-0.09
10-14	2.3	6.2	-0.19	2.8	4	-0.07
15-19	6.1	6.6	-0.02	7.1	8.1	-0.04
20-24	9.1	7.6	0.06	10	10.5	-0.02
25-29	7.2	7	0.01	6.2	6.6	-0.02
30-34	7.7	7.2	0.02	7.7	8.1	-0.02
35-39	8.2	7.4	0.03	8.3	8.4	0
40-44	8.8	7.3	0.06	9	8.6	0.01
45-49	9.9	7.4	0.09	10	8.6	0.05
50-54	10.8	8	0.1	11.1	9.6	0.05
55-59	10.7	8.6	0.07	10.8	9.2	0.05
60-64	9.1	8.4	0.03	8.4	7.4	0.04
65-69	3.2	4.1	-0.05	2.5	2.3	0.01
70-74	1.6	1.9	-0.03	1.4	1.3	0.01
75-79	1.1	1.3	-0.02	0.8	0.8	0.01
80-84	2.4	1.8	0.04	1.7	1.6	0
**Sex**						
Female	52.5	49.9	0.05	52.6	53.9	-0.03

Table S6.174. *Continued.* Selected baseline characteristics for US PharMetrics Plus for the long-term risk of personality disorders

Characteristic	Before propensity score matching			After propensity score matching		
	**Targets, %**	**Comparators, %**	**Standardized difference**	**Targets, %**	**Comparators, %**	**Standardized difference**
**Medical history (general)**						
Acute respiratory disease	40.9	18.5	0.5	36.4	41.3	-0.1
Attention deficit hyperactivity disorder	3.2	2.9	0.02	3.4	4	-0.03
Chronic liver disease	0.4	0.3	0.03	0.3	0.3	0
Chronic obstructive lung disease	2.3	1.2	0.08	1.6	1.9	-0.02
Crohn’s disease	0.3	0.2	0.02	0.3	0.4	-0.02
Dementia	1.4	0.4	0.11	0.8	1	-0.02
Depressive disorder	10.3	7	0.12	9.4	10.8	-0.05
Diabetes mellitus	12.2	5.9	0.22	10	8.5	0.05
Gastroesophageal reflux disease	11	5.8	0.19	9.9	10	0
Gastrointestinal hemorrhage	1.6	0.8	0.07	1.3	1.4	-0.01
Human immunodeficiency virus infection	0.3	0.2	0.03	0.3	0.4	-0.02
Hyperlipidemia	22.7	14.7	0.2	20.4	18.5	0.05
Hypertensive disorder	27.4	16.4	0.27	24	21.7	0.05
Lesion of liver	0.5	0.3	0.03	0.3	0.4	0
Obesity	12.4	5.8	0.23	10.4	9.8	0.02
Osteoarthritis	11.5	7	0.16	10.6	10.1	0.01
Pneumonia	10.6	1.1	0.41	4.2	2.8	0.07
Psoriasis	1.1	0.8	0.03	1	1	0
Renal impairment	5	1.9	0.17	3.1	3	0
Rheumatoid arthritis	1.1	0.6	0.06	1	0.9	0
Ulcerative colitis	0.3	0.2	0.02	0.3	0.3	0
Urinary tract infectious disease	5.5	2.4	0.16	4.6	4.7	-0.01
Viral hepatitis C	0.2	0.1	0.01	0.1	0.2	-0.01

Table S6.174. *Continued.* Selected baseline characteristics for US PharMetrics Plus for the long-term risk of personality disorders

Characteristic	Before propensity score matching			After propensity score matching		
	Targets, %	Comparators, %	Standardized difference	Targets, %	Comparators, %	Standardized difference
**Medical history (cardiovascular disease)**						
Atrial fibrillation	2.4	1.2	0.09	1.7	1.7	0
Cerebrovascular disease	1.2	0.7	0.05	0.9	1.1	-0.01
Coronary arteriosclerosis	4	2.2	0.1	3.2	3	0.01
Heart disease	10.3	5.6	0.17	8.3	8.1	0.01
Heart failure	2.4	0.9	0.12	1.6	1.7	-0.01
Peripheral vascular disease	2.1	0.9	0.1	1.5	1.5	0
Pulmonary embolism	0.6	0.2	0.07	0.4	0.3	0
Venous thrombosis	0.8	0.4	0.06	0.7	0.6	0.01
**Medical history (neoplasms)**						
Malignant lymphoma	0.3	0.2	0.03	0.3	0.2	0
Malignant neoplasm of anorectum	0.1	0.1	0	0.1	0.1	-0.01
Malignant tumor of breast	0.7	0.6	0.01	0.6	0.7	0
Malignant tumor of colon	0.2	0.1	0.01	0.1	0.1	0
Malignant tumor of lung	0.2	0.1	0.01	0.1	0.1	-0.01
Malignant tumor of urinary bladder	0.1	0.1	0.01	0.1	0.1	-0.01
Primary malignant neoplasm of prostate	0.5	0.4	0.01	0.4	0.4	0.01
**Medication use**						
Agents acting on the renin-angiotensin system	11.9	8	0.13	10.2	9	0.04
Antibacterials for systemic use	39.7	25.1	0.31	35.5	36.7	-0.03
Antidepressants	12.7	9.1	0.12	11.6	12.9	-0.04
Antiepileptics	6.1	3.8	0.1	5.1	5.4	-0.01
Antiinflammatory and antirheumatic products	19.8	10.8	0.25	17.4	17	0.01
Antineoplastic agents	2.8	1.9	0.06	2.5	2.7	-0.01
Antipsoriatics	1.8	1.3	0.05	1.8	2	-0.01
Antithrombotic agents	4.9	2.5	0.13	3.8	3.9	0

Table S6.174. *Continued.* Selected baseline characteristics for US PharMetrics Plus for the long-term risk of personality disorders

Characteristic	Before propensity score matching			After propensity score matching		
	**Targets, %**	**Comparators, %**	**Standardized difference**	**Targets, %**	**Comparators, %**	**Standardized difference**
**Medication use**						
Beta blocking agents	7	4.6	0.1	5.9	5.5	0.01
Calcium channel blockers	5.3	3.4	0.09	4.4	3.9	0.03
Diuretics	8.9	5.5	0.13	7.5	6.7	0.03
Drugs for acid-related disorders	12.9	7.5	0.18	11.4	11.6	-0.01
Drugs for obstructive airway diseases	24.6	14.3	0.26	21.8	22.5	-0.02
Drugs used in diabetes	7.1	3.8	0.15	5.8	4.9	0.04
Immunosuppressants	1.7	1	0.05	1.5	1.5	-0.01
Lipid modifying agents	11.4	7.9	0.12	9.8	8.7	0.04
Opioids	13.1	7.8	0.18	11.6	12	-0.01
Psycholeptics	13.3	8.6	0.15	12.2	13.2	-0.03
Psychostimulants, agents used for ADHD and nootropics	3.6	2.7	0.05	3.5	4.1	-0.03

Table S6.175. Selected baseline characteristics for US PharMetrics Plus for the long-term risk of self-harm and suicide

Characteristic	Before propensity score matching			After propensity score matching		
	Targets, %	Comparators, %	Standardized difference	Targets, %	Comparators, %	Standardized difference
**Age group (years)**						
0-4	0.7	3.7	-0.2	0.9	2	-0.1
5-9	1.2	5.5	-0.24	1.5	2.8	-0.09
10-14	2.3	6.1	-0.19	2.7	3.9	-0.06
15-19	6.1	6.6	-0.02	7.1	8	-0.03
20-24	9.1	7.5	0.06	10.1	10.6	-0.02
25-29	7.2	7	0.01	6.2	6.7	-0.02
30-34	7.7	7.1	0.02	7.7	8.2	-0.02
35-39	8.2	7.5	0.03	8.3	8.6	-0.01
40-44	8.8	7.3	0.06	9	8.6	0.01
45-49	9.9	7.3	0.09	10	8.6	0.05
50-54	10.8	7.9	0.1	11	9.6	0.05
55-59	10.7	8.6	0.07	10.8	9.2	0.05
60-64	9.1	8.5	0.02	8.4	7.4	0.04
65-69	3.2	4.1	-0.05	2.5	2.3	0.02
70-74	1.6	2	-0.03	1.4	1.3	0
75-79	1.1	1.3	-0.02	0.8	0.8	0.01
80-84	2.4	1.9	0.03	1.7	1.6	0.01
**Sex**						
Female	52.5	50.1	0.05	52.6	53.8	-0.02

Table S6.175. *Continued.* Selected baseline characteristics for US PharMetrics Plus for the long-term risk of self-harm and suicide

Characteristic	Before propensity score matching			After propensity score matching		
	Targets, %	Comparators, %	Standardized difference	Targets, %	Comparators, %	Standardized difference
**Medical history (general)**						
Acute respiratory disease	40.9	18.5	0.5	36.4	41.4	-0.1
Attention deficit hyperactivity disorder	3.2	2.9	0.02	3.4	4.1	-0.04
Chronic liver disease	0.4	0.2	0.03	0.3	0.3	0
Chronic obstructive lung disease	2.3	1.2	0.09	1.6	1.9	-0.02
Crohn’s disease	0.3	0.2	0.01	0.3	0.4	-0.02
Dementia	1.4	0.4	0.1	0.8	1	-0.01
Depressive disorder	10.3	6.9	0.12	9.5	10.9	-0.05
Diabetes mellitus	12.2	5.9	0.22	10	8.6	0.05
Gastroesophageal reflux disease	11	5.8	0.19	9.9	10.1	-0.01
Gastrointestinal hemorrhage	1.6	0.8	0.07	1.3	1.5	-0.01
Human immunodeficiency virus infection	0.3	0.2	0.03	0.3	0.4	-0.02
Hyperlipidemia	22.7	14.8	0.2	20.4	18.6	0.04
Hypertensive disorder	27.4	16.3	0.27	24	21.9	0.05
Lesion of liver	0.5	0.3	0.03	0.3	0.4	-0.01
Obesity	12.4	5.8	0.23	10.4	9.8	0.02
Osteoarthritis	11.5	7.1	0.15	10.6	10.1	0.01
Pneumonia	10.6	1.1	0.41	4.2	2.8	0.08
Psoriasis	1.1	0.8	0.03	1	1	0
Renal impairment	5	1.9	0.17	3.1	3	0.01
Rheumatoid arthritis	1.1	0.6	0.06	1	0.9	0.01
Schizophrenia	0.1	0.1	0.01	0.1	0.1	-0.01
Urinary tract infectious disease	5.5	2.4	0.16	4.6	4.7	0
Viral hepatitis C	0.2	0.1	0.01	0.1	0.2	-0.01

Table S6.175. *Continued.* Selected baseline characteristics for US PharMetrics Plus for the long-term risk of self-harm and suicide

Characteristic	Before propensity score matching			After propensity score matching		
	Targets, %	Comparators, %	Standardized difference	Targets, %	Comparators, %	Standardized difference
**Medical history (cardiovascular disease)**						
Atrial fibrillation	2.4	1.2	0.09	1.7	1.7	0.01
Cerebrovascular disease	1.2	0.7	0.05	0.9	1	-0.01
Coronary arteriosclerosis	4	2.3	0.1	3.2	3	0.01
Heart disease	10.3	5.7	0.17	8.3	8.1	0
Heart failure	2.4	0.9	0.12	1.6	1.6	-0.01
Ischemic heart disease	2.5	1.1	0.1	1.8	1.7	0
Peripheral vascular disease	2.1	1	0.09	1.5	1.5	0
Pulmonary embolism	0.6	0.2	0.06	0.4	0.4	0
Venous thrombosis	0.8	0.4	0.05	0.7	0.6	0.01
**Medical history (neoplasms)**						
Malignant neoplasm of anorectum	0.1	0.1	0.01	0.1	0.1	-0.01
Malignant neoplastic disease	3.9	3.1	0.04	3.5	3.5	0
Malignant tumor of breast	0.7	0.6	0.01	0.6	0.7	-0.01
Malignant tumor of colon	0.2	0.1	0.01	0.1	0.1	0
Malignant tumor of lung	0.2	0.1	0.02	0.1	0.1	-0.01
Primary malignant neoplasm of prostate	0.5	0.4	0.01	0.4	0.4	0
**Medication use**						
Agents acting on the renin-angiotensin system	11.9	8	0.13	10.3	9	0.04
Antibacterials for systemic use	39.7	25.1	0.32	35.5	36.8	-0.03
Antidepressants	12.7	9.1	0.12	11.7	13	-0.04
Antiepileptics	6.1	3.8	0.1	5.2	5.7	-0.02
Antiinflammatory and antirheumatic products	19.8	10.9	0.25	17.4	17.2	0.01
Antineoplastic agents	2.8	1.9	0.06	2.5	2.7	-0.01
Antipsoriatics	1.8	1.3	0.05	1.8	2	-0.01
Antithrombotic agents	4.9	2.5	0.13	3.8	3.9	0

Table S6.175. *Continued.* Selected baseline characteristics for US PharMetrics Plus for the long-term risk of self-harm and suicide

	Before propensity score matching			After propensity score matching		
	**Targets, %**	**Comparators, %**	**Standardized difference**	**Targets, %**	**Comparators, %**	**Standardized difference**
**Medication use**						
Beta blocking agents	7	4.6	0.1	5.9	5.5	0.01
Calcium channel blockers	5.3	3.5	0.09	4.4	3.9	0.02
Diuretics	8.9	5.4	0.13	7.5	6.8	0.03
Drugs for acid-related disorders	12.9	7.4	0.18	11.4	11.7	-0.01
Drugs for obstructive airway diseases	24.6	14.3	0.26	21.9	22.7	-0.02
Drugs used in diabetes	7.1	3.8	0.15	5.8	4.9	0.04
Immunosuppressants	1.7	1	0.05	1.5	1.6	-0.01
Lipid modifying agents	11.4	7.9	0.12	9.8	8.8	0.04
Opioids	13.1	7.7	0.18	11.6	12	-0.01
Psycholeptics	13.3	8.6	0.15	12.2	13.4	-0.03
Psychostimulants, agents used for ADHD and nootropics	3.6	2.7	0.05	3.5	4.1	-0.03

Table S6.176. Selected baseline characteristics for US PharMetrics Plus for the long-term risk of sleep disorders

Characteristic	Before propensity score matching			After propensity score matching		
	Targets, %	Comparators, %	Standardized difference	Targets, %	Comparators, %	Standardized difference
**Age group (years)**						
0-4	0.7	3.8	-0.21	1	2.2	-0.1
5-9	1.2	5.5	-0.24	1.6	3.1	-0.1
10-14	2.3	6.1	-0.19	3.1	4.4	-0.07
15-19	6.1	6.6	-0.02	8	8.8	-0.03
20-24	9.1	7.5	0.06	11.2	11.7	-0.01
25-29	7.2	7	0.01	6.8	7.1	-0.01
30-34	7.7	7.3	0.01	8.1	8.6	-0.02
35-39	8.2	7.4	0.03	8.6	8.7	0
40-44	8.8	7.3	0.06	8.9	8.4	0.02
45-49	9.9	7.3	0.09	9.4	8	0.05
50-54	10.8	8	0.1	10.2	8.9	0.04
55-59	10.7	8.6	0.07	9.9	8.5	0.05
60-64	9.1	8.4	0.03	7.6	6.6	0.04
65-69	3.2	4.2	-0.05	2.3	1.9	0.02
70-74	1.6	1.9	-0.03	1.2	1.1	0
75-79	1.1	1.3	-0.02	0.7	0.6	0.01
80-84	2.4	1.8	0.04	1.4	1.3	0.01
**Sex**						
Female	52.5	50.2	0.05	52.9	54.3	-0.03

Table S6.176. *Continued.* Selected baseline characteristics for US PharMetrics Plus for the long-term risk of sleep disorders

Characteristic	Before propensity score matching			After propensity score matching		
	Targets, %	Comparators, %	Standardized difference	Targets, %	Comparators, %	Standardized difference
**Medical history (general)**						
Acute respiratory disease	40.9	18.6	0.5	35.4	40.2	-0.1
Attention deficit hyperactivity disorder	3.2	2.9	0.02	3.2	3.8	-0.03
Chronic obstructive lung disease	2.3	1.2	0.09	1.1	1.3	-0.02
Crohn’s disease	0.3	0.2	0.01	0.3	0.3	-0.02
Dementia	1.4	0.4	0.11	0.6	0.7	-0.01
Depressive disorder	10.3	6.9	0.12	7.4	8.6	-0.05
Diabetes mellitus	12.2	5.9	0.22	8.4	7.1	0.05
Gastroesophageal reflux disease	11	5.8	0.19	7.8	8	-0.01
Gastrointestinal hemorrhage	1.6	0.8	0.07	1.1	1.3	-0.01
Human immunodeficiency virus infection	0.3	0.2	0.03	0.2	0.3	-0.02
Hyperlipidemia	22.7	14.8	0.2	17.3	15.5	0.05
Hypertensive disorder	27.4	16.3	0.27	20.1	18	0.05
Lesion of liver	0.5	0.2	0.04	0.3	0.3	0
Obesity	12.4	5.8	0.23	7.8	7.3	0.02
Osteoarthritis	11.5	6.9	0.16	8.4	8.2	0.01
Pneumonia	10.6	1.2	0.41	3.6	2.3	0.08
Psoriasis	1.1	0.8	0.03	0.9	1	-0.01
Renal impairment	5	1.8	0.17	2.4	2.3	0.01
Rheumatoid arthritis	1.1	0.5	0.06	0.8	0.8	0
Ulcerative colitis	0.3	0.2	0.02	0.3	0.3	0
Urinary tract infectious disease	5.5	2.4	0.16	4.2	4.2	0
Viral hepatitis C	0.2	0.1	0.01	0.1	0.1	-0.01

Table S6.176. *Continued.* Selected baseline characteristics for US PharMetrics Plus for the long-term risk of sleep disorders

Characteristic	Before propensity score matching			After propensity score matching		
	Targets, %	Comparators, %	Standardized difference	Targets, %	Comparators, %	Standardized difference
**Medical history (cardiovascular disease)**						
Atrial fibrillation	2.4	1.2	0.09	1.2	1.1	0.01
Cerebrovascular disease	1.2	0.6	0.06	0.7	0.8	-0.01
Coronary arteriosclerosis	4	2.3	0.1	2.3	2.2	0
Heart disease	10.3	5.6	0.17	6.3	6.2	0.01
Heart failure	2.4	0.9	0.12	1	1	0
Ischemic heart disease	2.5	1.1	0.1	1.3	1.2	0
Peripheral vascular disease	2.1	0.9	0.1	1.1	1.1	0
Pulmonary embolism	0.6	0.2	0.06	0.3	0.2	0
Venous thrombosis	0.8	0.4	0.06	0.5	0.5	0.01
**Medical history (neoplasms)**						
Malignant lymphoma	0.3	0.2	0.02	0.2	0.2	0.01
Malignant neoplasm of anorectum	0.1	0.1	0.01	0.1	0.1	-0.01
Malignant neoplastic disease	3.9	3.1	0.04	3	3	0
Malignant tumor of breast	0.7	0.6	0.01	0.6	0.6	0
Malignant tumor of colon	0.2	0.1	0.02	0.1	0.1	0
Malignant tumor of lung	0.2	0.1	0.02	0.1	0.1	0
Malignant tumor of urinary bladder	0.1	0.1	0.01	0.1	0.1	-0.01
Primary malignant neoplasm of prostate	0.5	0.4	0.01	0.3	0.3	0
**Medication use**						
Agents acting on the renin-angiotensin system	11.9	8	0.13	8.8	7.6	0.04
Antibacterials for systemic use	39.7	25.2	0.31	34.1	35.4	-0.03
Antidepressants	12.7	9.1	0.12	9.4	10.6	-0.04
Antiepileptics	6.1	3.8	0.1	3.9	4.2	-0.01
Antiinflammatory and antirheumatic products	19.8	10.9	0.25	15.8	15.6	0.01
Antineoplastic agents	2.8	1.9	0.06	2.2	2.4	-0.01

Table S6.176. *Continued.* Selected baseline characteristics for US PharMetrics Plus for the long-term risk of sleep disorders

Characteristic	Before propensity score matching			After propensity score matching		
	Targets, %	Comparators, %	Standardized difference	Targets, %	Comparators, %	Standardized difference
**Medication use**						
Antipsoriatics	1.8	1.2	0.05	1.9	2.1	-0.01
Antithrombotic agents	4.9	2.5	0.13	2.9	3	0
Beta blocking agents	7	4.6	0.1	4.8	4.4	0.02
Calcium channel blockers	5.3	3.5	0.09	3.7	3.2	0.02
Diuretics	8.9	5.5	0.13	6.3	5.6	0.03
Drugs for acid-related disorders	12.9	7.5	0.18	9.6	9.9	-0.01
Drugs for obstructive airway diseases	24.6	14.2	0.27	19.8	20.5	-0.02
Drugs used in diabetes	7.1	3.7	0.15	5	4.1	0.04
Immunosuppressants	1.7	1	0.06	1.2	1.4	-0.01
Lipid modifying agents	11.4	7.9	0.12	8.4	7.4	0.04
Opioids	13.1	7.8	0.17	9.9	10.2	-0.01
Psycholeptics	13.3	8.6	0.15	9.7	10.5	-0.03
Psychostimulants, agents used for ADHD and nootropics	3.6	2.7	0.05	3.2	3.7	-0.03

Table S6.177. Selected baseline characteristics for US PharMetrics Plus for the long-term risk of dementia

Characteristic	Before propensity score matching			After propensity score matching		
	Targets, %	Comparators, %	Standardized difference	Targets, %	Comparators, %	Standardized difference
**Age group (years)**						
0-4	0.7	3.7	-0.2	0.9	2	-0.1
5-9	1.2	5.5	-0.24	1.5	2.8	-0.1
10-14	2.3	6.1	-0.19	2.8	3.9	-0.06
15-19	6.1	6.6	-0.02	7.2	8.1	-0.04
20-24	9.1	7.5	0.06	10.1	10.7	-0.02
25-29	7.2	6.9	0.01	6.2	6.6	-0.02
30-34	7.7	7.1	0.02	7.7	8.2	-0.02
35-39	8.2	7.5	0.03	8.4	8.7	-0.01
40-44	8.8	7.3	0.06	9.1	8.7	0.01
45-49	9.9	7.4	0.09	10.1	8.7	0.05
50-54	10.8	8	0.1	11.1	9.6	0.05
55-59	10.7	8.6	0.07	10.8	9.3	0.05
60-64	9.1	8.5	0.02	8.4	7.3	0.04
65-69	3.2	4.1	-0.05	2.5	2.3	0.02
70-74	1.6	2	-0.03	1.3	1.3	0
75-79	1.1	1.3	-0.02	0.7	0.7	0.01
80-84	2.4	1.9	0.03	1.1	1.1	0
**Sex**						
Female	52.5	50.1	0.05	52.6	54	-0.03

Table S6.177. *Continued.* Selected baseline characteristics for US PharMetrics Plus for the long-term risk of dementia

Characteristic	Before propensity score matching			After propensity score matching		
	Targets, %	Comparators, %	Standardized difference	Targets, %	Comparators, %	Standardized difference
**Medical history (general)**						
Acute respiratory disease	40.9	18.6	0.5	36.5	41.4	-0.1
Attention deficit hyperactivity disorder	3.2	2.9	0.02	3.4	4.1	-0.04
Chronic liver disease	0.4	0.2	0.04	0.3	0.3	0
Chronic obstructive lung disease	2.3	1.2	0.09	1.5	1.8	-0.03
Crohn’s disease	0.3	0.2	0.01	0.3	0.4	-0.01
Depressive disorder	10.3	6.9	0.12	9.2	10.7	-0.05
Diabetes mellitus	12.2	5.9	0.22	9.8	8.4	0.05
Gastroesophageal reflux disease	11	5.9	0.19	9.7	10	-0.01
Gastrointestinal hemorrhage	1.6	0.8	0.07	1.3	1.5	-0.01
Human immunodeficiency virus infection	0.3	0.2	0.03	0.3	0.4	-0.02
Hyperlipidemia	22.7	14.8	0.2	20.1	18.1	0.05
Hypertensive disorder	27.4	16.4	0.27	23.5	21.3	0.05
Lesion of liver	0.5	0.2	0.04	0.3	0.4	-0.01
Obesity	12.4	5.8	0.23	10.4	9.8	0.02
Osteoarthritis	11.5	7.1	0.15	10.3	9.8	0.02
Pneumonia	10.6	1.1	0.41	4	2.7	0.07
Psoriasis	1.1	0.8	0.03	1	1.1	0
Renal impairment	5	1.9	0.17	2.8	2.7	0
Rheumatoid arthritis	1.1	0.6	0.06	1	0.9	0
Urinary tract infectious disease	5.5	2.4	0.16	4.4	4.5	-0.01
Viral hepatitis C	0.2	0.1	0.01	0.1	0.2	-0.01

Table S6.177. *Continued.* Selected baseline characteristics for US PharMetrics Plus for the long-term risk of dementia

Characteristic	Before propensity score matching			After propensity score matching		
	Targets, %	Comparators, %	Standardized difference	Targets, %	Comparators, %	Standardized difference
**Medical history (cardiovascular disease)**						
Atrial fibrillation	2.4	1.3	0.08	1.5	1.5	0
Cerebrovascular disease	1.2	0.7	0.05	0.8	0.9	-0.01
Coronary arteriosclerosis	4	2.3	0.1	2.9	2.8	0.01
Heart disease	10.3	5.7	0.17	7.8	7.7	0.01
Heart failure	2.4	0.9	0.12	1.4	1.5	-0.01
Ischemic heart disease	2.5	1.1	0.11	1.6	1.6	0
Peripheral vascular disease	2.1	0.9	0.1	1.2	1.3	-0.01
Pulmonary embolism	0.6	0.2	0.07	0.4	0.4	0
Venous thrombosis	0.8	0.4	0.05	0.6	0.6	0.01
**Medical history (neoplasms)**						
Malignant lymphoma	0.3	0.2	0.03	0.2	0.2	0
Malignant neoplasm of anorectum	0.1	0.1	0	0.1	0.1	-0.01
Malignant tumor of breast	0.7	0.6	0.01	0.6	0.6	0
Malignant tumor of colon	0.2	0.1	0.01	0.1	0.1	0
Malignant tumor of lung	0.2	0.1	0.01	0.1	0.1	-0.01
Malignant tumor of urinary bladder	0.1	0.1	0.01	0.1	0.1	0
Primary malignant neoplasm of prostate	0.5	0.4	0.01	0.4	0.4	0.01
**Medication use**						
Agents acting on the renin-angiotensin system	11.9	8	0.13	10.2	8.9	0.04
Antibacterials for systemic use	39.7	25.1	0.32	35.6	36.8	-0.03
Antidepressants	12.7	9.2	0.11	11.6	12.9	-0.04
Antiepileptics	6.1	3.8	0.1	5.1	5.5	-0.02
Antiinflammatory and antirheumatic products	19.8	10.8	0.25	17.4	17.1	0.01
Antineoplastic agents	2.8	1.9	0.06	2.5	2.7	-0.01
Antipsoriatics	1.8	1.3	0.05	1.8	2	-0.01

Table S6.177. *Continued.* Selected baseline characteristics for US PharMetrics Plus for the long-term risk of dementia

Characteristic	Before propensity score matching			After propensity score matching		
	Targets, %	Comparators, %	Standardized difference	Targets, %	Comparators, %	Standardized difference
**Medication use**						
Antithrombotic agents	4.9	2.5	0.13	3.7	3.8	-0.01
Beta blocking agents	7	4.6	0.1	5.8	5.4	0.02
Calcium channel blockers	5.3	3.5	0.09	4.3	3.8	0.03
Diuretics	8.9	5.5	0.13	7.5	6.6	0.03
Drugs for acid-related disorders	12.9	7.5	0.18	11.4	11.6	-0.01
Drugs for obstructive airway diseases	24.6	14.4	0.26	21.9	22.6	-0.02
Drugs used in diabetes	7.1	3.8	0.15	5.7	4.8	0.04
Immunosuppressants	1.7	1	0.05	1.5	1.5	-0.01
Lipid modifying agents	11.4	8	0.12	9.7	8.5	0.04
Opioids	13.1	7.8	0.17	11.6	11.9	-0.01
Psycholeptics	13.3	8.7	0.15	12.2	13.2	-0.03
Psychostimulants, agents used for ADHD and nootropics	3.6	2.7	0.05	3.5	4.2	-0.03

Table S6.178. Selected baseline characteristics for US PharMetrics Plus for the long-term risk of neurodevelopmental disorders

Characteristic	Before propensity score matching			After propensity score matching		
	Targets, %	Comparators, %	Standardized difference	Targets, %	Comparators, %	Standardized difference
**Age group (years)**						
0-4	0.7	3.7	-0.2	0.8	2	-0.1
5-9	1.2	5.5	-0.24	1.3	2.6	-0.09
10-14	2.3	6.1	-0.19	2.5	3.6	-0.06
15-19	6.1	6.6	-0.02	6.7	7.5	-0.03
20-24	9.1	7.6	0.06	9.6	10.3	-0.02
25-29	7.2	7	0.01	6	6.5	-0.02
30-34	7.7	7.2	0.02	7.6	8.1	-0.02
35-39	8.2	7.4	0.03	8.3	8.5	-0.01
40-44	8.8	7.2	0.06	9.1	8.8	0.01
45-49	9.9	7.4	0.09	10.1	8.7	0.05
50-54	10.8	8	0.1	11.3	9.9	0.05
55-59	10.7	8.6	0.07	11.1	9.6	0.05
60-64	9.1	8.4	0.02	8.7	7.8	0.04
65-69	3.2	4.1	-0.05	2.6	2.4	0.01
70-74	1.6	1.9	-0.03	1.4	1.4	0
75-79	1.1	1.3	-0.02	0.8	0.8	0.01
80-84	2.4	1.9	0.03	1.7	1.7	0
**Sex**						
Female	52.5	50	0.05	52.6	54.2	-0.03

Table S6.178. *Continued.* Selected baseline characteristics for US PharMetrics Plus for the long-term risk of neurodevelopmental disorders

Characteristic	Before propensity score matching			After propensity score matching		
	Targets, %	Comparators, %	Standardized difference	Targets, %	Comparators, %	Standardized difference
**Medical history (general)**						
Acute respiratory disease	40.9	18.6	0.5	36	40.8	-0.1
Chronic obstructive lung disease	2.3	1.2	0.09	1.6	1.9	-0.02
Crohn’s disease	0.3	0.2	0.02	0.3	0.4	-0.02
Dementia	1.4	0.4	0.11	0.8	0.9	-0.01
Depressive disorder	10.3	7	0.12	8.7	10	-0.04
Diabetes mellitus	12.2	6	0.22	10.2	8.9	0.04
Gastroesophageal reflux disease	11	5.9	0.19	9.9	10.1	-0.01
Gastrointestinal hemorrhage	1.6	0.8	0.07	1.3	1.5	-0.01
Human immunodeficiency virus infection	0.3	0.2	0.03	0.3	0.4	-0.02
Hyperlipidemia	22.7	14.9	0.2	20.9	19	0.05
Hypertensive disorder	27.4	16.3	0.27	24.5	22.5	0.05
Lesion of liver	0.5	0.3	0.03	0.3	0.4	-0.01
Obesity	12.4	5.8	0.23	10.5	10	0.02
Osteoarthritis	11.5	6.9	0.16	10.7	10.3	0.01
Pneumonia	10.6	1.1	0.41	4.2	2.9	0.07
Psoriasis	1.1	0.8	0.03	1	1.1	-0.01
Renal impairment	5	1.8	0.17	3.1	3.1	0
Rheumatoid arthritis	1.1	0.6	0.06	1	0.9	0.01
Ulcerative colitis	0.3	0.2	0.02	0.3	0.3	0
Urinary tract infectious disease	5.5	2.5	0.16	4.6	4.6	0
Viral hepatitis C	0.2	0.1	0.01	0.1	0.2	-0.01

Table S6.178. *Continued.* Selected baseline characteristics for US PharMetrics Plus for the long-term risk of neurodevelopmental disorders

Characteristic	Before propensity score matching			After propensity score matching		
	Targets, %	Comparators, %	Standardized difference	Targets, %	Comparators, %	Standardized difference
**Medical history (cardiovascular disease)**						
Atrial fibrillation	2.4	1.2	0.09	1.8	1.7	0
Cerebrovascular disease	1.2	0.7	0.05	0.9	1	-0.01
Coronary arteriosclerosis	4	2.3	0.1	3.2	3.1	0
Heart disease	10.3	5.7	0.17	8.4	8.2	0.01
Heart failure	2.4	0.9	0.12	1.6	1.7	-0.01
Ischemic heart disease	2.5	1.1	0.1	1.8	1.8	0
Peripheral vascular disease	2.1	0.9	0.1	1.5	1.5	0
Pulmonary embolism	0.6	0.2	0.07	0.4	0.4	0
Venous thrombosis	0.8	0.4	0.05	0.7	0.6	0
**Medical history (neoplasms)**						
Malignant lymphoma	0.3	0.2	0.03	0.3	0.3	0
Malignant neoplasm of anorectum	0.1	0.1	0	0.1	0.1	-0.01
Malignant neoplastic disease	3.9	3.1	0.04	3.5	3.6	0
Malignant tumor of breast	0.7	0.6	0.01	0.6	0.7	-0.01
Malignant tumor of colon	0.2	0.1	0.01	0.1	0.1	0
Malignant tumor of lung	0.2	0.1	0.02	0.1	0.1	-0.01
Malignant tumor of urinary bladder	0.1	0.1	0.01	0.1	0.1	-0.01
Primary malignant neoplasm of prostate	0.5	0.4	0.01	0.4	0.4	0.01
**Medication use**						
Agents acting on the renin-angiotensin system	11.9	8	0.13	10.5	9.4	0.04
Antibacterials for systemic use	39.7	25.2	0.31	35.1	36.5	-0.03
Antidepressants	12.7	9.2	0.11	11	12.3	-0.04
Antiepileptics	6.1	3.8	0.1	4.9	5.3	-0.01
Antiinflammatory and antirheumatic products	19.8	10.9	0.25	17.4	17.1	0.01
Antineoplastic agents	2.8	1.9	0.06	2.5	2.6	-0.01

Table S6.178. *Continued.* Selected baseline characteristics for US PharMetrics Plus for the long-term risk of neurodevelopmental disorders

Characteristic	Before propensity score matching			After propensity score matching		
	Targets, %	Comparators, %	Standardized difference	Targets, %	Comparators, %	Standardized difference
**Medication use**						
Antipsoriatics	1.8	1.3	0.05	1.8	1.9	-0.01
Antithrombotic agents	4.9	2.5	0.13	3.9	4.1	-0.01
Beta blocking agents	7	4.6	0.1	5.9	5.7	0.01
Calcium channel blockers	5.3	3.5	0.09	4.5	4.1	0.02
Diuretics	8.9	5.4	0.13	7.6	7	0.02
Drugs for acid-related disorders	12.9	7.5	0.18	11.4	11.6	-0.01
Drugs for obstructive airway diseases	24.6	14.2	0.26	21.7	22.3	-0.02
Drugs used in diabetes	7.1	3.8	0.14	5.9	5.1	0.04
Immunosuppressants	1.7	1	0.06	1.5	1.5	-0.01
Lipid modifying agents	11.4	8	0.12	10.1	9.1	0.04
Opioids	13.1	7.8	0.17	11.5	11.9	-0.01
Psycholeptics	13.3	8.6	0.15	11.8	12.7	-0.03
Psychostimulants, agents used for ADHD and nootropics	3.6	2.7	0.05	1.5	1.7	-0.02

Table S6.179. Selected baseline characteristics for US PharMetrics Plus for the long-term risk of any of psychiatric and neuropsychiatric disorders

Characteristic	Before propensity score matching			After propensity score matching		
	Targets, %	Comparators, %	Standardized difference	Targets, %	Comparators, %	Standardized difference
**Age group (years)**						
0-4	0.7	3.7	-0.2	1.3	3.1	-0.13
5-9	1.2	5.5	-0.24	1.9	3.8	-0.11
10-14	2.3	6	-0.19	3.4	4.8	-0.07
15-19	6.1	6.6	-0.02	8	8.6	-0.02
20-24	9.1	7.6	0.06	10.7	10.8	0
25-29	7.2	7	0.01	6.5	6.8	-0.01
30-34	7.7	7.2	0.02	7.9	8.3	-0.02
35-39	8.2	7.4	0.03	8.4	8.3	0
40-44	8.8	7.3	0.05	8.7	8.3	0.01
45-49	9.9	7.4	0.09	9.5	8.2	0.05
50-54	10.8	8	0.1	10.6	9.1	0.05
55-59	10.7	8.6	0.07	10.3	8.6	0.06
60-64	9.1	8.5	0.02	8	6.8	0.05
65-69	3.2	4.2	-0.05	2.3	2	0.02
70-74	1.6	2	-0.03	1.1	1.1	0
75-79	1.1	1.3	-0.02	0.6	0.5	0.01
80-84	2.4	1.9	0.03	0.9	1	-0.01
**Sex**						
Female	52.5	50	0.05	48.6	49.8	-0.03

Table S6.179. *Continued.* Selected baseline characteristics for US PharMetrics Plus for the long-term risk of any of psychiatric and neuropsychiatric disorders

Characteristic	Before propensity score matching			After propensity score matching		
	**Targets, %**	**Comparators, %**	**Standardized difference**	**Targets, %**	**Comparators, %**	**Standardized difference**
**Medical history (general)**						
Medical history: General						
Acute respiratory disease	40.9	18.5	0.5	32.6	37.4	-0.1
Chronic liver disease	0.4	0.3	0.03	0.2	0.2	0
Chronic obstructive lung disease	2.3	1.2	0.09	0.6	0.6	-0.01
Crohn's disease	0.3	0.2	0.01	0.2	0.3	-0.01
Diabetes mellitus	12.2	6	0.22	8	6.7	0.05
Gastroesophageal reflux disease	11	5.8	0.19	5.9	6	-0.01
Gastrointestinal hemorrhage	1.6	0.8	0.07	0.9	1	-0.01
Human immunodeficiency virus infection	0.3	0.2	0.03	0.2	0.3	-0.02
Hyperlipidemia	22.7	14.9	0.2	16.1	14.4	0.05
Hypertensive disorder	27.4	16.5	0.27	18.1	16	0.06
Lesion of liver	0.5	0.3	0.03	0.2	0.2	0
Obesity	12.4	5.7	0.23	6.7	6	0.03
Osteoarthritis	11.5	7.1	0.15	7.2	6.8	0.01
Pneumonia	10.6	1.1	0.41	3.1	1.9	0.08
Psoriasis	1.1	0.8	0.03	0.8	0.9	-0.01
Renal impairment	5	1.9	0.17	1.9	1.8	0.01
Rheumatoid arthritis	1.1	0.6	0.06	0.7	0.6	0
Ulcerative colitis	0.3	0.2	0.02	0.3	0.3	0
Urinary tract infectious disease	5.5	2.4	0.16	3.2	3.3	0
Viral hepatitis C	0.2	0.1	0.01	0.1	0.1	-0.01

Table S6.179. *Continued.* Selected baseline characteristics for US PharMetrics Plus for the long-term risk of any of psychiatric and neuropsychiatric disorders

Characteristic	Before propensity score matching			After propensity score matching		
	Targets, %	Comparators, %	Standardized diff	Targets, %	Comparators, %	Standardized diff
**Medical history (cardiovascular disease)**						
Atrial fibrillation	2.4	1.3	0.08	0.9	0.9	0
Cerebrovascular disease	1.2	0.7	0.05	0.5	0.5	0
Coronary arteriosclerosis	4	2.3	0.1	1.9	1.9	0
Heart disease	10.3	5.7	0.17	5.2	5.2	0
Heart failure	2.4	0.9	0.12	0.6	0.7	-0.01
Ischemic heart disease	2.5	1.1	0.11	1	1	0
Peripheral vascular disease	2.1	1	0.1	0.7	0.8	-0.01
Pulmonary embolism	0.6	0.2	0.07	0.2	0.2	0
Venous thrombosis	0.8	0.4	0.06	0.4	0.4	0
**Medical history (neoplasms)**						
Malignant lymphoma	0.3	0.2	0.02	0.2	0.2	0
Malignant neoplastic disease	3.9	3.1	0.04	2.6	2.7	0
Malignant tumor of breast	0.7	0.6	0.01	0.5	0.5	-0.01
Malignant tumor of lung	0.2	0.1	0.01	0.1	0.1	-0.01
Malignant tumor of urinary bladder	0.1	0.1	0	0.1	0.1	-0.01
Primary malignant neoplasm of prostate	0.5	0.4	0.01	0.3	0.3	0
**Medication use**						
Agents acting on the renin-angiotensin system	11.9	8.1	0.12	8.3	7.2	0.04
Antibacterials for systemic use	39.7	25.2	0.31	31.3	32.7	-0.03
Antidepressants	12.7	9.1	0.11	3	3.2	-0.01
Antiepileptics	6.1	3.9	0.1	2.3	2.2	0
Antiinflammatory and antirheumatic products	19.8	10.9	0.25	13.8	13.1	0.02
Antineoplastic agents	2.8	1.9	0.06	1.9	2.1	-0.01
Antipsoriatics	1.8	1.2	0.05	1.6	1.7	-0.01
Antithrombotic agents	4.9	2.5	0.13	2.4	2.4	0

Table S6.179. *Continued.* Selected baseline characteristics for US PharMetrics Plus for the long-term risk of any of psychiatric and neuropsychiatric disorders

Characteristic	Before propensity score matching			After propensity score matching		
	Targets, %	Comparators, %	Standardized difference	Targets, %	Comparators, %	Standardized difference
**Medication use**						
Beta blocking agents	7	4.6	0.1	3.9	3.6	0.02
Calcium channel blockers	5.3	3.5	0.09	3.4	2.9	0.03
Diuretics	8.9	5.6	0.13	5.7	4.9	0.04
Drugs for acid-related disorders	12.9	7.5	0.18	7.8	7.9	0
Drugs for obstructive airway diseases	24.6	14.2	0.26	17.6	18.2	-0.02
Drugs used in diabetes	7.1	3.9	0.14	4.7	3.9	0.04
Immunosuppressants	1.7	1.1	0.05	1.1	1.2	0
Lipid modifying agents	11.4	8.1	0.11	8.1	7	0.04
Opioids	13.1	7.7	0.18	8.1	8.1	0
Psycholeptics	13.3	8.5	0.15	5.7	6	-0.01
Psychostimulants, agents used for ADHD and nootropics	3.6	2.6	0.06	0.9	1.1	-0.02

Table S7. Risk of developing outcome events among individuals with COVID-19 for the whole study sample

**Outcome**		**Database**				
		**France IQVIA**	**Germany IQVIA**	**Italy IQVIA**	**UK IMRD**	**US PharMetrics Plus**
**Depression**	Short-term	1.47 (1.37-1.58)	1.25 (1.14-1.37)	1.90 (1.55-2.31)	1.07 (0.92-1.23)	1.05 (1.01-1.09)
	Medium-term	1.29 (1.18-1.41)	1.04 (0.93-1.16)	1.21 (0.93-1.56)	0.91 (0.76-1.08)	0.89 (0.84-0.93)
	Long-term	1.36 (1.25-1.47)	0.88 (0.78-0.98)	0.97 (0.73-1.27)	0.98 (0.83-1.14)	0.88 (0.84-0.93)
**Anxiety disorders**	Short-term	1.50 (1.42-1.59)	1.01 (0.91-1.11)	2.43 (1.61-3.61)	1.26 (1.10-1.43)	1.18 (1.14-1.21)
	Medium-term	1.29 (1.21-1.38)	0.90 (0.79-1.01)	1.05 (0.61-1.74)	0.91 (0.77-1.06)	0.89 (0.85-0.92)
	Long-term	1.37 (1.29-1.47)	1.00 (0.90-1.12)	0.87 (0.50-1.44)	0.88 (0.74-1.02)	0.89 (0.85-0.92)
**Alcohol misuse or dependence**	Short-term	1.06 (0.76-1.45)	0.49 (0.29-0.78)	1.52 (0.56-3.61)	0.83 (0.49-1.33)	0.97 (0.87-1.08)
	Medium-term	0.84 (0.54-1.25)	0.74 (0.43-1.19)	0.46 (0.07-1.67)	0.35 (0.14-0.72)	0.85 (0.75-0.96)
	Long-term	1.17 (0.83-1.63)	0.74 (0.46-1.13)	1.35 (0.49-3.26)	0.69 (0.38-1.15)	0.97 (0.86-1.09)
**Substance misuse or dependence**	Short-term	1.07 (0.87-1.29)	0.74 (0.59-0.92)	1.30 (0.63-2.48)	0.78 (0.51-1.15)	1.14 (1.08-1.19)
	Medium-term	1.01 (0.79-1.27)	0.56 (0.42-0.74)	0.67 (0.25-1.51)	0.34 (0.17-0.60)	0.78 (0.74-0.84)
	Long-term	1.12 (0.91-1.36)	0.73 (0.58-0.90)	1.09 (0.50-2.15)	0.54 (0.34-0.83)	0.82 (0.77-0.88)
**Bipolar disorders**	Short-term	0.90 (0.51-1.51)	0.41 (0.10-1.18)	0.72 (0.11-2.87)	1.49 (0.64-3.07)	0.92 (0.81-1.06)
	Medium-term	1.41 (0.78-2.41)	1.36 (0.44-3.47)	0.75 (0.04-3.95)	0.58 (0.09-2.01)	0.73 (0.61-0.86)
	Long-term	0.71 (0.35-1.31)	-	0.64 (0.03-3.28)	0.53 (0.13-1.52)	0.95 (0.81-1.11)
**Psychoses**	Short-term	0.94 (0.60-1.41)	0.40 (0.20-0.72)	2.39 (1.09-4.85)	0.81 (0.41-1.45)	1.41 (1.17-1.70)
	Medium-term	1.11 (0.65-1.79)	0.98 (0.56-1.62)	0.82 (0.18-2.55)	0.76 (0.33-1.50)	0.88 (0.67-1.14)
	Long-term	1.49 (0.91-2.34)	1.07 (0.66-1.68)	0.32 (0.05-1.13)	0.69 (0.26-1.47)	0.85 (0.65-1.09)

Table S7. *Continued.* Risk of developing outcome events among individuals with COVID-19 for the whole study sample

Outcome		Database				
		France IQVIA	Germany IQVIA	Italy IQVIA	UK IMRD	US PharMetrics Plus
**Personality disorders**	Short-term	1.23 (0.61-2.28)	0.76 (0.51-1.09)	1.55 (1.04-2.25)	1.48 (0.81-2.53)	0.54 (0.42-0.69)
	Medium-term	0.81 (0.30-1.81)	0.94 (0.59-1.43)	0.86 (0.53-1.34)	0.38 (0.12-0.92)	0.69 (0.53-0.89)
	Long-term	1.27 (0.63-2.39)	0.44 (0.24-0.73)	1.13 (0.73-1.68)	1.49 (0.79-2.59)	0.82 (0.63-1.05)
**Self-harm and suicide**	Short-term	1.00 (0.42-2.15)	-	-	0.77 (0.42-1.31)	1.17 (0.87-1.56)
	Medium-term	1.28 (0.54-2.75)	-	-	0.84 (0.49-1.36)	0.77 (0.52-1.11)
	Long-term	1.15 (0.47-2.48)	-	-	0.38 (0.16-0.77)	0.80 (0.55-1.15)
**Sleep disorders**	Short-term	1.34 (1.25-1.44)	1.03 (0.92-1.16)	1.73 (1.39-2.14)	1.11 (0.92-1.34)	1.21 (1.16-1.25)
	Medium-term	1.10 (1.01-1.21)	1.02 (0.89-1.16)	1.12 (0.84-1.47)	0.80 (0.62-1.02)	1.03 (0.98-1.08)
	Long-term	1.14 (1.05-1.24)	0.93 (0.82-1.06)	1.23 (0.96-1.57)	0.92 (0.73-1.16)	1.04 (0.99-1.09)
**Dementia**	Short-term	1.84 (1.12-2.91)	0.99 (0.73-1.33)	0.80 (0.34-1.68)	1.86 (1.50-2.28)	1.43 (1.26-1.63)
	Medium-term	0.64 (0.30-1.21)	1.22 (0.82-1.76)	1.80 (0.81-3.72)	1.65 (1.28-2.10)	0.84 (0.69-1.02)
	Long-term	1.48 (0.94-2.26)	1.22 (0.84-1.71)	0.53 (0.18-1.26)	0.97 (0.68-1.36)	0.86 (0.72-1.03)
**Neurodevelopmental disorders**	Short-term	2.20 (1.65-2.91)	0.70 (0.54-0.90)	1.17 (0.39-3.01)	0.72 (0.49-1.01)	0.93 (0.87-1.00)
	Medium-term	1.45 (0.99-2.09)	0.76 (0.56-1.01)	1.07 (0.23-3.54)	0.78 (0.53-1.11)	0.88 (0.81-0.96)
	Long-term	1.32 (0.94-1.82)	0.68 (0.51-0.88)	0.97 (0.22-2.97)	0.89 (0.64-1.22)	0.84 (0.77-0.91)
**Overall**	Short-term	1.51 (1.45-1.58)	1.14 (1.07-1.22)	1.89 (1.64-2.17)	1.15 (1.05-1.25)	1.22 (1.19-1.25)
	Medium-term	1.26 (1.19-1.34)	0.94 (0.87-1.02)	1.19 (0.99-1.42)	0.91 (0.82-1.01)	0.93 (0.90-0.96)
	Long-term	1.34 (1.27-1.41)	0.96 (0.88-1.03)	1.05 (0.88-1.25)	0.92 (0.82-1.02)	0.92 (0.89-0.95)

Table S8. Risk of developing outcome events among individuals with COVID-19 for males

Outcome		Database				
		**France IQVIA**	**Germany IQVIA**	**Italy IQVIA**	**UK IMRD**	**US PharMetrics Plus**
**Depression**	Short-term	1.55 (1.36-1.77)	1.21 (1.04-1.41)	2.29 (1.54-3.35)	1.14 (0.87-1.47)	1.05 (0.98-1.13)
	Medium-term	1.23 (1.05-1.45)	1.00 (0.82-1.20)	1.28 (0.78-2.01)	0.93 (0.68-1.24)	0.87 (0.80-0.95)
	Long-term	1.20 (1.02-1.40)	0.84 (0.70-1.00)	1.05 (0.59-1.77)	1.23 (0.93-1.61)	0.83 (0.75-0.90)
**Anxiety disorders**	Short-term	1.55 (1.40-1.70)	1.02 (0.86-1.20)	4.04 (2.02-7.75)	1.59 (1.24-2.01)	1.22 (1.16-1.29)
	Medium-term	1.34 (1.19-1.51)	0.69 (0.56-0.86)	1.32 (0.52-3.07)	0.81 (0.58-1.10)	0.89 (0.83-0.95)
	Long-term	1.24 (1.10-1.39)	0.94 (0.78-1.13)	0.92 (0.30-2.32)	1.16 (0.88-1.52)	0.92 (0.86-0.99)
**Alcohol misuse or dependence**	Short-term	1.05 (0.67-1.57)	0.80 (0.45-1.32)	1.63 (0.40-5.36)	0.99 (0.50-1.77)	1.07 (0.94-1.22)
	Medium-term	0.66 (0.34-1.17)	0.58 (0.27-1.12)	0.40 (0.02-2.16)	0.33 (0.08-0.90)	0.87 (0.74-1.02)
	Long-term	1.09 (0.69-1.66)	0.89 (0.52-1.44)	0.79 (0.17-2.61)	0.81 (0.37-1.56)	0.99 (0.85-1.15)
**Substance misuse or dependence**	Short-term	0.96 (0.72-1.26)	0.93 (0.70-1.22)	1.79 (0.70-4.11)	0.87 (0.51-1.40)	1.26 (1.18-1.34)
	Medium-term	0.94 (0.66-1.30)	0.57 (0.39-0.82)	1.01 (0.28-2.82)	0.34 (0.13-0.72)	0.76 (0.70-0.83)
	Long-term	1.12 (0.84-1.45)	0.79 (0.59-1.05)	0.68 (0.19-1.80)	0.80 (0.45-1.32)	0.88 (0.81-0.96)
**Bipolar disorders**	Short-term	1.07 (0.40-2.37)	0.34 (0.02-1.78)	1.48 (0.20-6.96)	5.15 (1.35-17.09)	1.14 (0.89-1.44)
	Medium-term	1.47 (0.50-3.46)	0.54 (0.03-3.02)	1.33 (0.07-7.93)	-	0.73 (0.53-0.97)
	Long-term	1.17 (0.42-2.78)	-	-	-	0.73 (0.54-0.98)
**Psychoses**	Short-term	0.76 (0.36-1.41)	0.33 (0.10-0.80)	2.39 (0.64-7.02)	0.59 (0.18-1.45)	1.39 (0.98-1.80)
	Medium-term	1.10 (0.50-2.14)	0.93 (0.41-1.88)	0.33 (0.02-1.95)	0.59 (0.14-1.67)	0.86 (0.54-1.33)
	Long-term	1.00 (0.49-1.85)	1.43 (0.74-2.59)	0.65 (0.03-4.03)	0.36 (0.06-1.23)	0.80 (0.54-1.17)

Table S8. *Continued.* Risk of developing outcome events among individuals with COVID-19 for males

Outcome		Database				
		**France IQVIA**	**Germany IQVIA**	**Italy IQVIA**	**UK IMRD**	**US PharMetrics Plus**
**Personality disorders**	Short-term	1.25 (0.35-3.44)	1.02 (0.54-1.79)	0.64 (0.24-1.40)	1.05 (0.16-3.74)	0.51 (0.31-0.70)
	Medium-term	0.57 (0.09-2.09)	0.96 (0.46-1.80)	1.12 (0.45-2.47)	0.89 (0.13-3.29)	0.82 (0.52-1.25)
	Long-term	1.23 (0.39-3.26)	0.30 (0.09-0.74)	1.28 (0.61-2.47)	0.99 (0.16-3.50)	0.80 (0.50-1.22)
**Self-harm and suicide**	Short-term	0.82 (0.12-3.62)	-	-	0.76 (0.23-1.91)	1.90 (1.08-3.23)
	Medium-term	0.78 (0.04-4.23)	-	-	0.87 (0.33-1.91)	0.97 (0.41-2.06)
	Long-term	0.94 (0.13-4.17)	-	-	0.15 (0.01-0.73)	0.33 (0.12-0.74)
**Sleep disorders**	Short-term	1.31 (1.17-1.48)	1.05 (0.88-1.25)	1.68 (1.14-2.43)	1.10 (0.78-1.51)	1.28 (1.22-1.35)
	Medium-term	1.08 (0.92-1.25)	0.97 (0.79-1.18)	1.21 (0.74-1.89)	0.71 (0.45-1.06)	1.06 (0.99-1.13)
	Long-term	1.15 (1.00-1.32)	0.98 (0.81-1.17)	1.10 (0.72-1.62)	0.86 (0.58-1.22)	1.07 (1.00-1.14)
**Dementia**	Short-term	1.02 (0.38-2.30)	1.09 (0.64-1.77)	0.80 (0.12-3.20)	1.74 (1.21-2.43)	1.67 (1.37-2.04)
	Medium-term	0.69 (0.20-1.83)	0.93 (0.43-1.77)	0.52 (0.03-3.01)	1.35 (0.85-2.05)	1.01 (0.74-1.36)
	Long-term	1.71 (0.75-3.49)	1.64 (0.89-2.84)	-	1.01 (0.57-1.69)	0.79 (0.58-1.05)
**Neurodevelopmental disorders**	Short-term	3.28 (2.22-4.77)	0.60 (0.41-0.85)	0.28 (0.01-1.69)	0.73 (0.45-1.13)	0.90 (0.81-1.01)
	Medium-term	1.44 (0.78-2.51)	0.93 (0.65-1.30)	0.69 (0.03-4.84)	0.88 (0.49-1.48)	0.81 (0.71-0.92)
	Long-term	1.27 (0.76-2.03)	0.93 (0.66-1.29)	1.98 (0.41-7.13)	0.80 (0.49-1.25)	0.83 (0.73-0.94)
**Overall**	Short-term	1.48 (1.38-1.59)	1.13 (1.02-1.24)	2.05 (1.61-2.58)	1.19 (1.03-1.38)	1.25 (1.20-1.30)
	Medium-term	1.27 (1.16-1.40)	0.86 (0.76-0.98)	1.08 (0.78-1.47)	0.80 (0.66-0.96)	0.92 (0.87-0.97)
	Long-term	1.23 (1.12-1.34)	0.97 (0.86-1.09)	1.07 (0.79-1.43)	1.00 (0.84-1.19)	0.97 (0.92-1.02)

Table S9. Risk of developing outcome events among individuals with COVID-19 for females

**Outcome**		**Database**				
		**France IQVIA**	**Germany IQVIA**	**Italy IQVIA**	**UK IMRD**	**US PharMetrics Plus**
**Depression**	Short-term	1.50 (1.37-1.64)	1.20 (1.08-1.35)	1.52 (1.17-1.97)	1.02 (0.84-1.21)	1.08 (1.03-1.13)
	Medium-term	1.29 (1.16-1.43)	1.03 (0.89-1.18)	1.13 (0.81-1.54)	0.84 (0.67-1.03)	0.92 (0.87-0.97)
	Long-term	1.46 (1.33-1.60)	0.86 (0.75-1.00)	1.05 (0.76-1.44)	1.01 (0.82-1.22)	0.93 (0.88-0.99)
**Anxiety disorders**	Short-term	1.52 (1.42-1.63)	0.99 (0.87-1.12)	1.70 (0.95-2.92)	1.16 (0.98-1.36)	1.16 (1.12-1.21)
	Medium-term	1.27 (1.16-1.38)	0.97 (0.84-1.12)	1.29 (0.64-2.41)	0.93 (0.76-1.11)	0.90 (0.86-0.95)
	Long-term	1.51 (1.40-1.63)	1.04 (0.91-1.19)	0.82 (0.41-1.51)	0.83 (0.68-1.01)	0.92 (0.87-0.96)
**Alcohol misuse or dependence**	Short-term	1.55 (0.92-2.52)	0.16 (0.03-0.51)	1.77 (0.09-12.61)	0.74 (0.28-1.61)	0.79 (0.65-0.95)
	Medium-term	0.93 (0.45-1.76)	1.18 (0.52-2.36)	-	0.46 (0.11-1.26)	0.81 (0.65-0.99)
	Long-term	1.29 (0.71-2.21)	0.39 (0.11-0.99)	4.83 (0.22-50.39)	0.74 (0.28-1.63)	1.04 (0.85-1.26)
**Substance misuse or dependence**	Short-term	1.19 (0.89-1.58)	0.57 (0.38-0.82)	0.34 (0.02-1.74)	0.67 (0.31-1.27)	0.97 (0.90-1.04)
	Medium-term	0.99 (0.69-1.39)	0.58 (0.37-0.87)	0.40 (0.02-2.13)	0.37 (0.13-0.82)	0.76 (0.69-0.83)
	Long-term	1.28 (0.93-1.72)	0.82 (0.57-1.14)	1.90 (0.43-6.11)	0.28 (0.10-0.63)	0.81 (0.73-0.89)
**Bipolar disorders**	Short-term	0.86 (0.39-1.66)	0.47 (0.07-1.70)	-	0.86 (0.25-2.28)	0.90 (0.76-1.07)
	Medium-term	1.46 (0.70-2.85)	1.51 (0.42-4.36)	-	0.53 (0.08-1.75)	0.77 (0.63-0.95)
	Long-term	0.49 (0.18-1.07)	-	0.88 (0.05-5.08)	0.75 (0.17-2.18)	1.01 (0.83-1.22)
**Psychoses**	Short-term	1.33 (0.73-2.27)	0.41 (0.17-0.83)	2.98 (1.12-7.33)	1.17 (0.48-2.45)	1.57 (1.23-1.99)
	Medium-term	1.25 (0.62-2.33)	1.06 (0.48-2.10)	3.13 (0.62-11.99)	0.83 (0.28-1.99)	0.87 (0.61-1.22)
	Long-term	1.21 (0.54-2.42)	0.80 (0.36-1.57)	0.50 (0.03-2.91)	1.02 (0.30-2.62)	0.86 (0.61-1.20)

Table S9. *Continued.* Risk of developing outcome events among individuals with COVID-19 for females

**Outcome**		**Database**				
		**France IQVIA**	**Germany IQVIA**	**Italy IQVIA**	**UK IMRD**	**US PharMetrics Plus**
**Personality disorders**	Short-term	1.13 (0.45-2.50)	0.61 (0.35-0.99)	1.78 (1.09-2.81)	1.64 (0.85-2.91)	0.65 (0.47-0.89)
	Medium-term	0.99 (0.22-3.05)	0.83 (0.44-1.44)	0.85 (0.46-1.47)	0.18 (0.03-0.58)	0.67 (0.49-0.91)
	Long-term	1.92 (0.78-4.26)	0.55 (0.27-1.02)	1.38 (0.78-2.31)	1.25 (0.64-2.27)	0.92 (0.66-1.25)
**Self-harm and suicide**	Short-term	1.33 (0.51-3.08)	-	-	0.82 (0.39-1.52)	0.94 (0.66-1.33)
	Medium-term	1.15 (0.45-2.56)	-	-	0.78 (0.40-1.40)	0.82 (0.52-1.26)
	Long-term	1.62 (0.57-3.98)	-	-	0.40 (0.14-0.94)	0.97 (0.63-1.45)
**Sleep disorders**	Short-term	1.34 (1.22-1.47)	1.04 (0.88-1.21)	2.04 (1.51-2.72)	1.17 (0.92-1.47)	1.15 (1.10-1.21)
	Medium-term	1.13 (1.01-1.27)	1.06 (0.89-1.27)	1.42 (0.98-2.00)	0.95 (0.69-1.29)	1.01 (0.95-1.07)
	Long-term	1.14 (1.02-1.26)	0.90 (0.75-1.07)	1.49 (1.03-2.09)	0.86 (0.61-1.17)	1.00 (0.94-1.07)
**Dementia**	Short-term	2.30 (1.27-3.95)	0.95 (0.64-1.35)	1.47 (0.54-3.56)	1.77 (1.36-2.28)	1.46 (1.23-1.73)
	Medium-term	0.49 (0.17-1.16)	1.22 (0.76-1.87)	1.52 (0.49-3.85)	1.86 (1.36-2.50)	0.76 (0.58-0.97)
	Long-term	1.81 (1.00-3.16)	1.20 (0.76-1.82)	1.50 (0.49-3.71)	0.97 (0.62-1.46)	0.90 (0.71-1.14)
**Neurodevelopmental disorders**	Short-term	1.80 (1.15-2.74)	1.02 (0.70-1.45)	4.16 (0.71-21.06)	0.62 (0.34-1.04)	0.97 (0.88-1.06)
	Medium-term	1.67 (1.02-2.64)	0.59 (0.34-0.96)	1.62 (0.22-7.05)	0.92 (0.54-1.48)	0.93 (0.84-1.04)
	Long-term	1.33 (0.84-2.05)	0.52 (0.30-0.82)	-	0.87 (0.55-1.33)	0.91 (0.82-1.01)
**Overall**	Short-term	1.56 (1.47-1.65)	1.17 (1.07-1.27)	1.86 (1.53-2.24)	1.14 (1.02-1.27)	1.19 (1.15-1.23)
	Medium-term	1.31 (1.22-1.41)	0.98 (0.88-1.09)	1.22 (0.97-1.53)	0.99 (0.87-1.12)	0.95 (0.91-0.99)
	Long-term	1.44 (1.34-1.53)	0.93 (0.84-1.04)	1.20 (0.95-1.51)	0.93 (0.81-1.06)	0.91 (0.87-0.95)

Table S10. Risk of developing outcome events among individuals with COVID-19 for individuals aged below 18 years

**Outcome**		**Database**				
		**France IQVIA**	**Germany IQVIA**	**Italy IQVIA**	**UK IMRD**	**US PharMetrics Plus**
**Depression**	Short-term	0.70 (0.30-1.40)	1.04 (0.55-1.82)	-	2.33 (1.08-4.58)	0.96 (0.82-1.12)
	Medium-term	1.71 (0.82-3.27)	1.02 (0.47-1.97)	-	0.42 (0.07-1.36)	0.89 (0.73-1.07)
	Long-term	2.32 (1.50-3.49)	1.26 (0.75-2.00)	-	1.34 (0.55-2.79)	0.88 (0.74-1.04)
**Anxiety disorders**	Short-term	1.06 (0.76-1.44)	1.63 (1.12-2.48)	-	1.98 (1.15-3.21)	1.20 (1.07-1.34)
	Medium-term	1.09 (0.74-1.57)	1.05 (0.63-1.66)	-	1.25 (0.63-2.26)	0.94 (0.81-1.07)
	Long-term	1.03 (0.74-1.39)	0.93 (0.56-1.46)	-	1.24 (0.65-2.17)	0.78 (0.68-0.89)
**Alcohol misuse or dependence**	Short-term	-	-	-	-	0.98 (0.46-1.88)
	Medium-term	-	-	-	-	0.70 (0.28-1.53)
	Long-term	-	-	-	4.66 (0.22-48.65)	0.82 (0.38-1.59)
**Substance misuse or dependence**	Short-term	-	1.20 (0.19-4.30)	-	1.45 (0.07-9.43)	1.11 (0.79-1.51)
	Medium-term	3.24 (0.16-25.35)	5.39 (0.69-33.33)	-	-	0.77 (0.51-1.14)
	Long-term	1.29 (0.20-4.80)	0.36 (0.02-1.79)	-	-	0.99 (0.71-1.34)
**Bipolar disorders**	Short-term	-	-	-	-	0.81 (0.41-1.45)
	Medium-term	-	-	-	-	0.78 (0.41-1.38)
	Long-term	-	-	-	-	0.63 (0.32-1.14)
**Psychoses**	Short-term	1.60 (0.21-7.89)	-	-	-	1.07 (0.39-2.50)
	Medium-term	1.16 (0.05-9.65)	-	2.83 (0.09-90.96)	-	0.63 (0.15-1.84)
	Long-term	3.32 (0.38-22.38)	-	-	-	0.86 (0.28-2.14)

Table S10. *Continued.* Risk of developing outcome events among individuals with COVID-19 for individuals aged below 18 years

**Outcome**		**Database**				
		**France IQVIA**	**Germany IQVIA**	**Italy IQVIA**	**UK IMRD**	**US PharMetrics Plus**
**Personality disorders**	Short-term	-	0.73 (0.12-2.51)	-	-	1.05 (0.49-2.03)
	Medium-term	1.66 (0.09-10.31)	0.87 (0.13-3.22)	-	-	0.65 (0.26-1.37)
	Long-term	-	0.90 (0.20-2.78)	-	-	0.70 (0.32-1.37)
**Self-harm and suicide**	Short-term	3.06 (0.64-11.27)	-	-	0.67 (0.11-2.35)	1.52 (0.86-2.55)
	Medium-term	2.72 (0.41-11.28)	0.85 (0.56-1.24)	-	0.96 (0.54-1.59)	0.90 (0.74-1.09)
	Long-term	-	0.86 (0.59-1.22)	1.58 (0.08-9.95)	0.90 (0.49-1.51)	0.87 (0.73-1.03)
**Sleep disorders**	Short-term	1.40 (0.92-2.08)	1.11 (0.55-2.02)	-	0.50 (0.12-1.39)	0.98 (0.80-1.20)
	Medium-term	1.18 (0.65-2.01)	1.00 (0.46-1.91)	-	0.63 (0.15-1.83)	0.82 (0.64-1.05)
	Long-term	0.48 (0.25-0.85)	1.12 (0.53-2.13)	-	0.87 (0.21-2.50)	0.86 (0.69-1.07)
**Dementia**	Short-term	-	-	-	-	1.06 (0.48-2.08)
	Medium-term	-	-	-	-	0.57 (0.23-1.19)
	Long-term	-	-	-	-	1.16 (0.59-2.10)
**Neurodevelopmental disorders**	Short-term	2.65 (1.74-3.94)	0.77 (0.53-1.09)	-	0.56 (0.25-1.07)	0.93 (0.79-1.09)
	Medium-term	1.29 (0.65-2.35)	0.85 (0.56-1.24)	-	0.96 (0.54-1.59)	0.90 (0.74-1.09)
	Long-term	1.76 (1.05-2.83)	0.86 (0.59-1.22)	1.58 (0.08-9.95)	0.90 (0.49-1.51)	0.87 (0.73-1.03)
**Overall**	Short-term	1.37 (1.10-1.69)	1.01 (0.77-1.29)	-	0.92 (0.61-1.34)	1.07 (0.97-1.18)
	Medium-term	1.06 (0.79-1.40)	0.84 (0.61-1.13)	-	0.96 (0.64-1.38)	0.89 (0.79-1.00)
	Long-term	1.29 (1.02-1.61)	1.02 (0.78-1.32)	0.31 (0.02-1.71)	0.81 (0.53-1.19)	0.80 (0.72-0.90)

Table S11. Risk of developing outcome events among individuals with COVID-19 for individuals aged between 18 and 24 years

Outcome		Database				
		France IQVIA	Germany IQVIA	Italy IQVIA	UK IMRD	US PharMetrics Plus
**Depression**	Short-term	1.11 (0.76-1.57)	1.11 (0.78-1.56)	2.70 (0.58-9.62)	0.95 (0.63-1.37)	0.86 (0.78-0.96)
	Medium-term	1.49 (1.03-2.10)	0.87 (0.57-1.28)	3.51 (0.85-12.45)	0.68 (0.41-1.08)	0.84 (0.74-0.95)
	Long-term	1.92 (1.43-2.54)	0.81 (0.52-1.21)	-	0.89 (0.54-1.39)	0.82 (0.73-0.93)
**Anxiety disorders**	Short-term	1.37 (1.10-1.69)	0.57 (0.37-0.84)	2.69 (0.49-11.67)	1.13 (0.78-1.58)	0.96 (0.88-1.04)
	Medium-term	1.27 (0.98-1.62)	0.93 (0.63-1.33)	3.09 (1.00-8.49)	0.98 (0.64-1.44)	0.96 (0.87-1.06)
	Long-term	1.41 (1.13-1.74)	1.06 (0.74-1.49)	0.49 (0.07-2.04)	0.71 (0.44-1.10)	0.86 (0.78-0.95)
**Alcohol misuse or dependence**	Short-term	1.26 (0.07-7.65)	0.69 (0.04-3.91)	-	0.69 (0.04-3.75)	1.19 (0.90-1.54)
	Medium-term	-	-	-	-	1.07 (0.79-1.43)
	Long-term	-	-	-	3.11 (0.46-13.52)	1.01 (0.76-1.33)
**Substance misuse or dependence**	Short-term	1.56 (0.66-3.30)	1.00 (0.41-2.10)	-	0.36 (0.02-1.77)	1.36 (1.20-1.54)
	Medium-term	1.23 (0.35-3.32)	0.21 (0.01-0.98)	1.11 (0.05-9.12)	0.20 (0.01-0.96)	0.89 (0.75-1.04)
	Long-term	1.42 (0.58-3.12)	1.05 (0.42-2.23)	-	1.99 (0.56-5.58)	0.92 (0.79-1.08)
**Bipolar disorders**	Short-term	1.42 (0.08-8.36)	-	-	1.14 (0.06-6.42)	1.10 (0.83-1.43)
	Medium-term	1.64 (0.22-7.48)	-	-	-	0.71 (0.50-0.99)
	Long-term	2.60 (0.13-20.70)	-	-	-	0.97 (0.72-1.28)
**Psychoses**	Short-term	-	0.40 (0.02-2.19)	-	1.20 (0.06-6.79)	1.47 (0.90-2.31)
	Medium-term	0.42 (0.02-2.61)	3.39 (0.67-14.40)	-	-	0.75 (0.34-1.47)
	Long-term	3.82 (0.86-14.70)	2.00 (0.50-6.63)	-	4.83 (0.22-50.39)	0.67 (0.35-1.21)

Table S11. *Continued.* Risk of developing outcome events among individuals with COVID-19 for individuals aged between 18 and 24 years

Outcome		Database				
		France IQVIA	Germany IQVIA	Italy IQVIA	UK IMRD	US PharMetrics Plus
**Personality disorders**	Short-term	2.34 (0.12-15.81)	1.22 (0.43-3.01)	-	1.32 (0.31-3.90)	0.50 (0.29-0.81)
	Medium-term	1.80 (0.07-24.07)	0.39 (0.06-1.31)	5.58 (0.55-52.03)	0.25 (0.01-1.18)	0.80 (0.50-1.25)
	Long-term	1.33 (0.06-12.93)	0.37 (0.02-1.88)	-	1.17 (0.27-3.54)	0.86 (0.55-1.28)
**Self-harm and suicide**	Short-term	3.81 (0.43-33.75)	-	-	0.51 (0.08-1.73)	1.53 (0.91-2.51)
	Medium-term	1.24 (0.06-9.08)	-	-	0.20 (0.01-1.00)	0.86 (0.37-1.82)
	Long-term	-	-	-	0.65 (0.10-2.40)	1.04 (0.54-1.87)
**Sleep disorders**	Short-term	1.36 (0.97-1.87)	0.45 (0.23-0.79)	1.01 (0.15-4.14)	1.30 (0.63-2.44)	0.92 (0.78-1.07)
	Medium-term	0.67 (0.41-1.04)	0.72 (0.38-1.25)	0.69 (0.04-3.75)	0.78 (0.23-1.92)	0.87 (0.72-1.04)
	Long-term	1.15 (0.80-1.61)	0.98 (0.58-1.58)	2.61 (0.35-14.06)	0.33 (0.05-1.10)	1.04 (0.88-1.23)
**Dementia**	Short-term	-	-	-	-	0.73 (0.39-1.31)
	Medium-term	-	-	-	-	0.97 (0.50-1.75)
	Long-term	2.11 (0.09-23.85)	-	-	-	0.50 (0.24-0.94)
**Neurodevelopmental disorders**	Short-term	2.46 (1.02-5.45)	0.40 (0.06-1.38)	1.43 (0.07-9.25)	0.98 (0.38-2.13)	1.03 (0.90-1.19)
	Medium-term	2.06 (0.69-5.03)	1.41 (0.56-3.10)	-	0.71 (0.21-1.76)	0.93 (0.79-1.09)
	Long-term	1.12 (0.17-4.19)	0.74 (0.24-1.82)	-	0.58 (0.17-1.45)	1.09 (0.93-1.27)
**Overall**	Short-term	1.51 (1.27-1.78)	0.83 (0.64-1.07)	1.63 (0.69-3.43)	1.01 (0.76-1.31)	1.04 (0.97-1.12)
	Medium-term	1.17 (0.95-1.44)	0.84 (0.63-1.11)	2.09 (1.00-4.05)	0.75 (0.53-1.04)	0.92 (0.84-1.00)
	Long-term	1.45 (1.21-1.73)	1.10 (0.84-1.42)	0.38 (0.09-1.17)	0.80 (0.56-1.11)	0.90 (0.82-0.98)

Table S12. Risk of developing outcome events among individuals with COVID-19 for individuals aged between 25 and 44 years

Outcome		Database				
		**France IQVIA**	**Germany IQVIA**	**Italy IQVIA**	**UK IMRD**	**US PharMetrics Plus**
**Depression**	Short-term	1.38 (1.20-1.58)	1.18 (1.00-1.39)	2.18 (1.31-3.51)	0.89 (0.70-1.12)	1.01 (0.95-1.09)
	Medium-term	1.35 (1.16-1.57)	0.94 (0.76-1.16)	1.10 (0.56-2.05)	1.01 (0.78-1.28)	0.86 (0.79-0.94)
	Long-term	1.34 (1.16-1.54)	0.96 (0.78-1.16)	0.99 (0.52-1.77)	1.16 (0.91-1.45)	0.91 (0.84-1.00)
**Anxiety disorders**	Short-term	1.63 (1.48-1.79)	1.03 (0.86-1.22)	1.66 (0.86-3.02)	1.00 (0.80-1.22)	1.15 (1.09-1.21)
	Medium-term	1.38 (1.23-1.55)	0.90 (0.73-1.11)	1.21 (0.46-2.77)	0.89 (0.69-1.13)	0.90 (0.84-0.97)
	Long-term	1.44 (1.29-1.61)	1.06 (0.88-1.28)	1.52 (0.62-3.34)	0.90 (0.70-1.14)	0.92 (0.86-0.99)
**Alcohol misuse or dependence**	Short-term	1.07 (0.60-1.82)	0.67 (0.22-1.60)	-	0.53 (0.16-1.31)	0.82 (0.67-1.00)
	Medium-term	0.84 (0.34-1.79)	0.59 (0.17-1.52)	3.09 (0.14-34.77)	0.41 (0.07-1.32)	0.77 (0.61-0.95)
	Long-term	1.38 (0.79-2.30)	0.69 (0.26-1.50)	-	0.77 (0.22-2.03)	0.92 (0.73-1.14)
**Substance misuse or dependence**	Short-term	1.02 (0.73-1.41)	0.54 (0.32-0.84)	6.55 (2.02-20.38)	0.37 (0.13-0.83)	1.10 (1.02-1.20)
	Medium-term	0.99 (0.62-1.51)	0.49 (0.28-0.81)	0.63 (0.03-4.22)	0.39 (0.12-0.97)	0.76 (0.68-0.85)
	Long-term	1.09 (0.76-1.53)	0.54 (0.33-0.83)	6.42 (1.53-24.97)	0.40 (0.14-0.92)	0.81 (0.71-0.91)
**Bipolar disorders**	Short-term	1.66 (0.72-3.46)	-	-	0.37 (0.02-1.86)	0.93 (0.74-1.17)
	Medium-term	1.15 (0.37-2.88)	0.85 (0.04-4.88)	-	-	0.97 (0.73-1.28)
	Long-term	-	-	-	1.60 (0.24-6.44)	1.02 (0.77-1.33)
**Psychoses**	Short-term	0.81 (0.34-1.65)	0.34 (0.05-1.14)	2.27 (0.60-6.82)	0.70 (0.17-2.03)	1.41 (0.95-2.07)
	Medium-term	1.09 (0.46-2.26)	0.85 (0.25-2.23)	0.83 (0.04-6.11)	0.42 (0.07-1.44)	0.98 (0.59-1.58)
	Long-term	0.83 (0.33-1.83)	0.56 (0.13-1.62)	-	0.37 (0.02-1.74)	0.77 (0.41-1.34)

Table S12. *Continued.* Risk of developing outcome events among individuals with COVID-19 for individuals aged between 25 and 44 years

**Outcome**		**Database**				
		**France IQVIA**	**Germany IQVIA**	**Italy IQVIA**	**UK IMRD**	**US PharMetrics Plus**
**Personality disorders**	Short-term	2.18 (0.80-5.30)	0.27 (0.08-0.67)	0.91 (0.05-5.79)	1.64 (0.77-3.16)	0.67 (0.44-1.00)
	Medium-term	0.39 (0.02-1.89)	0.86 (0.35-1.81)	0.75 (0.03-7.73)	0.25 (0.01-1.17)	0.90 (0.57-1.36)
	Long-term	0.92 (0.13-3.80)	0.40 (0.10-1.10)	-	1.40 (0.61-2.84)	0.75 (0.45-1.21)
**Self-harm and suicide**	Short-term	0.94 (0.14-3.62)	-	-	0.75 (0.22-1.90)	1.66 (0.85-3.08)
	Medium-term	0.72 (0.04-4.93)	-	-	0.87 (0.32-1.98)	1.47 (0.64-3.13)
	Long-term	0.33 (0.02-1.95)	-	-	0.14 (0.01-0.72)	1.08 (0.35-2.70)
**Sleep disorders**	Short-term	1.29 (1.12-1.49)	0.86 (0.67-1.09)	1.76 (0.91-3.18)	1.14 (0.79-1.60)	1.15 (1.07-1.23)
	Medium-term	1.10 (0.91-1.32)	0.94 (0.71-1.23)	1.05 (0.50-2.04)	0.69 (0.41-1.10)	1.00 (0.92-1.09)
	Long-term	1.21 (1.03-1.42)	0.96 (0.74-1.24)	0.75 (0.30-1.60)	0.87 (0.55-1.31)	1.06 (0.97-1.16)
**Dementia**	Short-term	0.90 (0.04-8.40)	7.45 (2.30-24.08)	-	-	1.03 (0.60-1.69)
	Medium-term	2.83 (0.09-90.96)	-	-	0.82 (0.04-4.81)	1.08 (0.60-1.84)
	Long-term	0.87 (0.13-3.63)	1.07 (0.05-8.72)	-	2.42 (0.10-28.45)	1.07 (0.61-1.81)
**Neurodevelopmental disorders**	Short-term	1.62 (0.73-3.28)	0.39 (0.12-0.97)	0.85 (0.04-7.12)	0.60 (0.25-1.21)	0.98 (0.86-1.11)
	Medium-term	2.42 (0.99-5.33)	0.86 (0.35-1.81)	4.04 (0.44-27.44)	0.61 (0.24-1.28)	0.88 (0.75-1.02)
	Long-term	0.92 (0.33-2.15)	0.29 (0.07-0.78)	1.58 (0.22-6.90)	1.19 (0.68-1.97)	0.91 (0.79-1.04)
**Overall**	Short-term	1.50 (1.38-1.62)	1.13 (1.00-1.27)	2.44 (1.75-3.35)	0.92 (0.79-1.08)	1.19 (1.13-1.24)
	Medium-term	1.39 (1.26-1.53)	0.84 (0.71-0.98)	1.08 (0.68-1.67)	0.79 (0.65-0.95)	0.91 (0.86-0.97)
	Long-term	1.35 (1.23-1.49)	1.00 (0.86-1.15)	1.09 (0.70-1.65)	1.00 (0.83-1.19)	0.93 (0.87-0.99)

Table S13. Risk of developing outcome events among individuals with COVID-19 for individuals aged between 45 and 64 years

Outcome		Database				
		**France IQVIA**	**Germany IQVIA**	**Italy IQVIA**	**UK IMRD**	**US PharMetrics Plus**
**Depression**	Short-term	1.72 (1.54-1.91)	1.07 (0.92-1.23)	1.64 (1.19-2.24)	1.15 (0.87-1.50)	1.13 (1.06-1.20)
	Medium-term	1.28 (1.11-1.46)	1.02 (0.86-1.20)	1.32 (0.90-1.88)	0.92 (0.67-1.24)	0.93 (0.86-1.02)
	Long-term	1.41 (1.25-1.59)	0.70 (0.58-0.84)	0.95 (0.63-1.40)	1.01 (0.74-1.36)	0.91 (0.83-0.99)
**Anxiety disorders**	Short-term	1.57 (1.44-1.71)	0.98 (0.83-1.16)	3.29 (1.72-6.14)	1.50 (1.17-1.91)	1.28 (1.22-1.35)
	Medium-term	1.36 (1.22-1.52)	0.86 (0.71-1.05)	0.67 (0.26-1.48)	0.80 (0.58-1.09)	0.85 (0.79-0.92)
	Long-term	1.46 (1.32-1.62)	0.96 (0.80-1.13)	0.59 (0.22-1.34)	1.09 (0.81-1.44)	0.93 (0.87-1.00)
**Alcohol misuse or dependence**	Short-term	1.47 (0.94-2.21)	0.21 (0.77-0.48)	-	1.14 (0.55-2.14)	0.97 (0.82-1.15)
	Medium-term	0.74 (0.39-1.30)	0.58 (0.25-1.16)	0.68 (0.03-4.43)	0.35 (0.06-1.15)	0.85 (0.69-1.05)
	Long-term	1.25 (0.73-2.03)	0.72 (0.36-1.32)	0.48 (0.02-3.16)	0.78 (0.34-1.59)	1.03 (0.85-1.25)
**Substance misuse or dependence**	Short-term	1.17 (0.87-1.54)	0.65 (0.47-0.90)	0.25 (0.01-1.35)	1.14 (0.62-1.96)	1.02 (0.95-1.10)
	Medium-term	1.00 (0.70-1.38)	0.51 (0.32-0.75)	1.06 (0.30-2.92)	0.26 (0.04-0.85)	0.73 (0.65-0.80)
	Long-term	1.38 (1.02-1.83)	0.68 (0.48-0.93)	0.68 (0.48-0.93)	0.74 (0.37-1.36)	0.82 (0.73-0.90)
**Bipolar disorders**	Short-term	0.43 (0.13-1.11)	0.80 (0.12-2.91)	1.13 (0.06-7.14)	0.67 (0.04-3.47)	0.92 (0.68-1.22)
	Medium-term	1.82 (0.80-3.71)	1.28 (0.19-5.11)	1.48 (0.08-9.62)	0.81 (0.04-4.57)	0.67 (0.46-0.95)
	Long-term	0.49 (0.15-1.22)	-	1.20 (0.05-10.11）	-	0.93 (0.65-1.30)
**Psychoses**	Short-term	0.93 (0.46-1.72)	0.80 (0.30-1.81)	1.56 (0.23-6.15)	0.47 (0.08-1.56)	1.41 (0.93-2.09)
	Medium-term	0.84 (0.31-1.93)	0.64 (0.19-1.65)	0.66 (0.03-4.20)	2.40 (0.64-7.05)	1.10 (0.65-1.79)
	Long-term	1.21 (0.46-2.64)	0.67 (0.23-1.60)	0.65 (0.03-4.03)	0.86 (0.14-2.95)	1.32 (0.77-2.19)

Table S13. *Continued.* Risk of developing outcome events among individuals with COVID-19 for individuals aged between 45 and 64 years

Outcome		Database				
		**France IQVIA**	**Germany IQVIA**	**Italy IQVIA**	**UK IMRD**	**US PharMetrics Plus**
**Personality disorders**	Short-term	1.35 (0.37-3.89)	0.82 (0.40-1.53)	3.17 (1.40-6.72)	1.18 (0.18-4.36)	0.54 (0.25-1.03)
	Medium-term	1.13 (0.18-4.13)	0.84 (0.31-1.88)	1.22 (0.33-3.58)	1.29 (0.18-5.29)	0.69 (0.39-1.18)
	Long-term	1.30 (0.37-3.57)	0.72 (0.29-1.53)	1.10 (0.25-3.47)	0.64 (0.03-3.93)	0.84 (0.44-1.52)
**Self-harm and suicide**	Short-term	0.29 (0.02-1.59)	-	-	2.10 (0.68-5.45)	1.09 (0.47-2.28)
	Medium-term	0.43 (0.02-2.38)	-	-	0.56 (0.08-2.12)	0.57 (0.22-1.28)
	Long-term	2.81 (0.68-9.75)	-	-	1.09 (0.23-3.67)	0.70 (0.25-1.70)
**Sleep disorders**	Short-term	1.45 (1.29-1.62)	1.16 (0.97-1.38)	1.48 (1.04-2.06)	1.18 (0.84-1.61)	1.24 (1.18-1.31)
	Medium-term	1.25 (1.09-1.43)	1.03 (0.84-1.26)	0.99 (0.65-1.46)	0.96 (0.64-1.38)	1.03 (0.97-1.10)
	Long-term	1.19 (1.05-1.35)	0.85 (0.70-1.03)	1.43 (0.99-2.01)	1.00 (0.68-1.42)	1.03 (0.96-1.10)
**Dementia**	Short-term	1.85 (0.38-6.74)	2.90 (1.31-5.95)	1.92 (0.10-11.94)	0.93 (0.21-2.95)	0.97 (0.70-1.33)
	Medium-term	0.43 (0.02-2.31)	2.52 (0.94-6.06)	-	0.23 (0.01-1.15)	0.89 (0.57-1.35)
	Long-term	0.57 (0.03-2.96)	0.88 (0.29-2.18)	1.54 (0.08-9.79)	0.63 (0.10-2.26)	0.83 (0.53-1.25)
**Neurodevelopmental disorders**	Short-term	2.29 (0.88-5.21)	1.63 (0.87-2.88)	-	0.90 (0.34-1.99)	1.07 (0.90-1.27)
	Medium-term	2.14 (0.88-4.77)	0.47 (0.11-1.35)	2.00 (0.09-17.68)	0.87 (0.26-2.21)	1.16 (0.94-1.42)
	Long-term	1.31 (0.57-2.70)	0.55 (0.23-1.15)	-	1.60 (0.70-3.30)	0.85 (0.69-1.05)
**Overall**	Short-term	1.61 (1.50-1.73)	1.11 (1.00-1.23)	1.79 (1.44-2.23)	1.23 (1.04-1.46)	1.26 (1.21-1.31)
	Medium-term	1.32 (1.20-1.44)	0.95 (0.84-1.08)	0.95 (0.71-1.27)	0.91 (0.74-1.12)	0.94 (0.89-1.00)
	Long-term	1.37 (1.25-1.49)	0.83 (0.73-0.95)	1.10 (0.83-1.43)	1.00 (0.82-1.21)	0.96 (0.91-1.02)

Table S14. Risk of developing outcome events among individuals with COVID-19 for individuals aged between 65 years or older

Outcome		Database				
		**France IQVIA**	**Germany IQVIA**	**Italy IQVIA**	**UK IMRD**	**US PharMetrics Plus**
**Depression**	Short-term	1.46 (1.20-1.77)	1.47 (1.19-1.80)	2.01 (1.40-2.85)	1.18 (0.68-1.94)	1.46 (1.28-1.67)
	Medium-term	1.19 (0.93-1.50)	0.99 (0.71-1.35)	1.24 (0.74-1.98)	0.64 (0.26-1.32)	0.96 (0.79-1.16)
	Long-term	1.00 (0.77-1.28)	0.90 (0.65-1.22)	1.10 (0.66-1.76)	0.88 (0.46-1.57)	0.95 (0.77-1.16)
**Anxiety disorders**	Short-term	1.36 (1.16-1.60)	1.05 (0.76-1.43)	2.21 (0.46-7.67)	1.71 (0.97-2.84)	1.37 (1.21-1.54)
	Medium-term	1.01 (0.81-1.25)	0.89 (0.56-1.35)	0.42 (0.02-2.33)	0.66 (0.25-1.44)	0.81 (0.67-0.98)
	Long-term	1.28 (1.05-1.55)	0.86 (0.55-1.29)	1.55 (0.33-5.23)	0.68 (0.31-1.34)	1.03 (0.86-1.24)
**Alcohol misuse or dependence**	Short-term	0.62 (0.15-1.79)	1.41 (0.56-3.09)	2.17 (0.28-11.25)	0.59 (0.09-2.19)	1.62 (1.10-2.35)
	Medium-term	0.34 (0.02-1.63)	4.60 (1.55-12.26)	-	1.28 (0.20-4.78)	0.89 (0.49-1.50)
	Long-term	0.64 (0.15-1.84)	1.17 (0.43-2.68)	-	1.04 (0.15-4.04)	1.12 (0.65-1.84)
**Substance misuse or dependence**	Short-term	0.82 (0.39-1.56)	1.44 (0.86-2.30)	1.17 (0.17-4.72)	0.93 (0.26-2.57)	1.32 (1.09-1.58)
	Medium-term	0.93 (0.40-1.91)	1.32 (0.69-2.34)	-	1.83 (0.50-5.29)	0.71 (0.53-0.94)
	Long-term	0.54 (0.21-1.18)	1.62 (0.97-2.59)	-	0.29 (0.02-1.46)	0.66 (0.48-0.88)
**Bipolar disorders**	Short-term	1.19 (0.28-3.45)	1.17 (0.06-6.41)	-	5.30 (1.14-24.05)	1.33 (0.76-2.26)
	Medium-term	-	2.44 (0.43-12.53)	-	1.46 (0.07-9.45)	0.93 (0.36-2.07)
	Long-term	1.23 (0.27-3.99)	-	2.28 (0.12-15.44)	-	1.16 (0.52-2.38)
**Psychoses**	Short-term	2.27 (0.79-5.61)	0.21 (0.03-0.67)	2.40 (0.66-7.79)	1.30 (0.42-3.35)	2.01 (1.40-2.86)
	Medium-term	2.40 (0.66-6.94)	1.21 (0.40-2.93)	1.26 (0.17-6.17)	0.57 (0.09-2.06)	1.12 (0.60-1.98)
	Long-term	1.49 (0.35-4.52)	2.44 (1.06-5.13)	0.79 (0.04-5.15)	1.02 (0.15-4.08)	1.28 (0.73-2.16)

Table S14. *Continued.* Risk of developing outcome events among individuals with COVID-19 for individuals aged between 65 years or older

**Outcome**		**Database**				
		**France IQVIA**	**Germany IQVIA**	**Italy IQVIA**	**UK IMRD**	**US PharMetrics Plus**
**Personality disorders**	Short-term	0.99 (0.05-5.71)	1.30 (0.52-2.84)	1.13 (0.69-1.77)	-	0.59 (0.17-1.62)
	Medium-term	-	1.64 (0.56-4.23)	0.85 (0.47-1.45)	-	0.57 (0.13-1.85)
	Long-term	0.53 (0.03-3.13)	0.43 (0.02-2.15)	1.20 (0.73-1.90)	-	0.92 (0.25-2.65)
**Self-harm and suicide**	Short-term	-	-	-	2.15 (0.10-17.27)	0.39 (0.02-2.46)
	Medium-term	2.94 (0.38-16.46)	-	-	2.45 (0.13-16.54)	-
	Long-term	2.49 (0.33-13.56)	-	-	-	1.80 (0.34-7.76)
**Sleep disorders**	Short-term	1.51 (1.29-1.77)	1.18 (0.92-1.51)	1.95 (1.36-2.76)	1.19 (0.77-1.79）	1.13 (1.01-1.28)
	Medium-term	1.10 (0.89-1.34)	0.99 (0.71-1.35)	1.38 (0.86-2.13)	0.85 (0.46-1.43)	0.92 (0.78-1.08)
	Long-term	1.35 (1.12-1.62)	0.95 (0.69-1.30)	1.16 (0.72-1.80)	0.76 (0.41-1.31)	1.12 (0.95-1.32)
**Dementia**	Short-term	1.65 (0.96-2.71)	0.64 (0.44-0.91)	1.10 (0.48-2.27)	1.62 (1.29-2.02)	1.94 (1.63-2.31)
	Medium-term	0.67 (0.29-1.34)	0.97 (0.61-1.47)	1.34 (0.54-2.91)	1.47 (1.12-1.89)	0.66 (0.48-0.90)
	Long-term	1.97 (1.17-3.19)	1.13 (0.76-1.64)	0.53 (0.16-1.37)	0.83 (0.57-1.18)	0.98 (0.73-1.30)
**Neurodevelopmental disorders**	Short-term	5.58 (1.82-17.04)	1.29 (0.65-2.36)	-	2.11 (0.79-5.06)	1.30 (0.93-1.79)
	Medium-term	1.24 (0.28-3.87)	1.36 (0.55-2.89)	-	0.91 (0.14-3.42)	0.76 (0.47-1.19)
	Long-term	2.03 (0.75-4.90)	2.10 (1.03-3.98)	-	-	1.74 (1.15-2.58)
**Overall**	Short-term	1.54 (1.37-1.73)	1.34 (1.15-1.55)	1.76 (1.37-2.23)	1.57 (1.31-1.86)	1.48 (1.34-1.62)
	Medium-term	1.10 (0.94-1.28)	1.07 (0.86-1.33)	1.63 (1.19-2.20)	1.17 (0.93-1.46)	0.83 (0.71-0.96)
	Long-term	1.30 (1.11-1.50)	1.16 (0.93-1.44)	1.07 (0.77-1.46)	0.85 (0.64-1.10)	0.94 (0.80-1.10)
